# Supplementary material for: How to account for the uncertainty from standard toxicity tests in species sensitivity distributions: An example in non-target plants
Source: PLoS One. 2021 Jan 7;16(1):e0245071. doi: 10.1371/journal.pone.0245071 (PMC7790375; doi:10.1371/journal.pone.0245071)
Supplement: S1 Table — (PDF) [file pone.0245071.s001.pdf]

How to account for the uncertainty from standard toxicity tests in species sensitivity distributions: an example in non-target plants

Sandrine Charles Dan Wu Virginie Ducrot

29 June 2020

Contact: sandrine.charles@univ-lyon1.fr

S1 Table: Corresponding species and common names to the EPPO code

EPPO code	Species name	Common name
ALLCE	Allium cepa	Onion
AVESA	Avena sativa	Oat
BEAVA	Beta vulgaris	Sugar beet
BRSNW	Brassica napus winter	Oilseed rape
BRSOL	Brassica oleracea	Cabbage
CUMSA	Cucumis sativus	Cucumber
FAGES	Fagopyrum esculentum	Buckwheat
GLXMA	Glycine max	Soybean
HELAN	Helianthus annuus	Sunflower
LOLPE	Lolium perenne	Perennial ryegrass
LYPES	Solanum lycopersicum	Tomato
RAPSR	Raphanus sativus	Radish
TRZAW	Triticum aestivum winter	Winter wheat
ZEAMA	Zea mays subs. Amylacea	Corn
