# Supplementary material for: How to account for the uncertainty from standard toxicity tests in species sensitivity distributions: An example in non-target plants
Source: PLoS One. 2021 Jan 7;16(1):e0245071. doi: 10.1371/journal.pone.0245071 (PMC7790375; doi:10.1371/journal.pone.0245071)
Supplement: S2 Table — (PDF) [file pone.0245071.s002.pdf]

How to account for the uncertainty from standard toxicity tests in species sensitivity distributions: an example in non-target plants

Sandrine Charles   Dan Wu   Virginie Ducrot

29 June 2020

Contact: sandrine.charles@univ-lyon1.fr

S2 Table: Formulation of active substances in seven products

Product number	Chemical family	Mode of action
1	Diphenylethers + Oxyacetamides + Pyridinecarboxamides	Inhibition of carotenoid synthesis + Inhibition of cell division
2	Pyridinecarboxamides	Inhibition of carotenoid synthesis
3	Benzofuran + Uracil + Carbamate	Inhibition of lipid synthesis + Inhibition of photosynthesis at photosystem II
4	Sulfonylureas + Phenoxy + Sulfonly-amino-carbonyl-triazolinones	Synthetic auxin + Inhibition of acetolactate synthase
5	Triazines	Inhibition of cellulose synthesis
6	Sulfonylureas + Sulfonly-amino-carbonyl-triazolinones	Inhibition of acetolactate synthase
7	Sulfonylureas + Sulfonly-amino-carbonyl-triazolinones	Inhibition of acetolactate synthase

Note: The proportion of active substances is different between products 6 and 7.
