# Supplementary material for: How to account for the uncertainty from standard toxicity tests in species sensitivity distributions: An example in non-target plants
Source: PLoS One. 2021 Jan 7;16(1):e0245071. doi: 10.1371/journal.pone.0245071 (PMC7790375; doi:10.1371/journal.pone.0245071)
Supplement: S1 Archive — It is a zip file containing seven folders (one folder per case study). Each folder contains five files report_xxx.pdf with detailed results of the dose-response analyses, one file corresponding to does-response analysis per endpoint. It also contains one file ER50_censoring.pdf for censored ER50 and one file SSD_analyses.pdf for results of SSD analyses. (ZIP) [file pone.0245071.s004.zip › S1_archive/Study1/ER50_censoring.pdf]

ER_{50} censoring after dose-response analyses

Study 1

25 June 2020

Contact: sandrine.charles@univ-lyon1.fr

This document provides the ER_{50} censoring after dose-response analyses for study 1, including four parts:

- Raw data description (test item rates);
- Summary on ER_{50} from a Bayesian dose-response fit;
- Censoring on ER_{50} according to two criteria;
  - Criterion 1 (C_1) based on the ratio of the probablities, that is the ratio of the probability that the ER_{50} lies within the range of the LCI95 and the maximum tested rate over the probability that the ER_{50} lies in the CI95;
  - Criterion 2 (C_2) based on the ratio of distances, that is the ratio of the distance between the LCI95 of ER_{50} and the maximum tested rate over the extend of the CI95 of ER_{50};
- Graphical presentation of censored ER_{50} per species per endpoint.

Meaning of symbols used in the document

- min_rate: minimum tested rate;
- max_rate: maximum tested rate;
- ER_{50}: 50% effective rate;
- CI95: 95% credible interval;
- ER_{50}_median: median of ER_{50};
- ER_{50}_LCI95: lower bound of the CI95 of ER_{50};
- ER_{50}_UCI95: upper bound of the CI95 of ER_{50};
- C_1: criterion based on the ratio of probabilities;
- C_2: criterion based on the ratio of distances;
- T: decision threshold;
- Censored ER_{50} [lower bound; upper bound]:
  - C1T0.0: censored ER_{50} according to C_1 with T = 0.0;
  - C1T0.2: censored ER_{50} according to C_1 with T = 0.2;

- C1T0.4: censored ER_{50} according to C_1 with T = 0.4;
- C1T0.5: censored ER_{50} according to C_1 with T = 0.5;
- C1T0.6: censored ER_{50} according to C_1 with T = 0.6;
- C1T0.8: censored ER_{50} according to C_1 with T = 0.8;
- C1T1.0: censored ER_{50} according to C_1 with T = 1.0;
- C2T0.0: censored ER_{50} according to C_2 with T = 0.0;
- C2T0.2: censored ER_{50} according to C_2 with T = 0.2;
- C2T0.4: censored ER_{50} according to C_2 with T = 0.4;
- C2T0.5: censored ER_{50} according to C_2 with T = 0.5;
- C2T0.6: censored ER_{50} according to C_2 with T = 0.6;
- C2T0.8: censored ER_{50} according to C_2 with T = 0.8;
- C2T1.0: censored ER_{50} according to C_2 with T = 1.0.

Seeding emergence (SE) test of study 1

Raw data description (test item rates) for the SE test

```
Number of species:  10
EPPO code:  ALLCE AVESA BEAVA BRSNW CUMSA GLXMA HELAN LYPES TRZAW ZEAMA
```

Table 1: Correspondance between species and common names according to the EPPO code

EPPO code	Species name	Common name
ALLCE	*Allium cepa*	Onion
AVESA	*Avena sativa*	Oat
BEAVA	*Beta vulgaris*	Sugar beet
BRSNW	*Brassica napus winter*	Oilseed rape
CUMSA	*Cucumis sativus*	Cucumber
GLXMA	*Glycine max*	Soybean
HELAN	*Helianthus annuus*	Sunflower
LYPES	*Solanum lycopersicum*	Tomato
TRZAW	*Triticum aestivum winter*	Winter wheat
ZEAMA	*Zea mays subs. Amylacea*	Corn

Table 2: Tested rates per species (ml product/ha) for the SE test of study 1

ALLCE	AVESA	BEAVA	BRSNW	CUMSA	GLXMA	HELAN	LYPES	TRZAW	ZEAMA
0.00	0.00	0.00	0.00	0.00	0.00	0.00	0.00	0.00	0.00
31.07	71.47	31.07	31.07	31.07	31.07	31.07	31.07	71.47	71.47
71.47	164.38	71.47	71.47	71.47	71.47	71.47	71.47	164.38	164.38
164.38	378.07	164.38	164.38	164.38	164.38	164.38	164.38	378.07	378.07
378.07	869.57	378.07	378.07	378.07	378.07	378.07	378.07	869.57	869.57
869.57	2000.00	869.57	869.57	869.57	869.57	869.57	869.57	2000.00	2000.00

SE test - emergence endpoint

Table 3: Minimum and maximum tested rates, median, quantile 2.5% and quantile 97.5% of the ER_{50} posterior distribution - emergence endpoint of the SE test of study 1

species	min_rate	max_rate	ER_{50}_median	ER_{50}_LCI95	ER_{50}_UCI95
ALLCE	31.07	869.57	1420.196	692.4289	3756.931
AVESA	71.47	2000.00	1506.596	1053.5823	2425.402
BEAVA	31.07	869.57	1203.154	908.4504	2777.003
BRSNW	31.07	869.57	1262.485	932.6021	2937.444
CUMSA	31.07	869.57	2674.779	1416.8116	6583.219
GLXMA	31.07	869.57	1270.655	933.6909	3001.201
HELAN	31.07	869.57	2543.192	1395.4354	6113.227
LYPES	31.07	869.57	1246.794	924.3375	2886.373
TRZAW	71.47	2000.00	2093.930	1879.2971	2909.464
ZEAMA	71.47	2000.00	5813.628	3111.8055	14090.879

Table 4: Censored ER_{50} according to criterion 1 and the seven decision thresholds - emergence endpoint of the SE test of study 1

species	C1T0.0	C1T0.2	C1T0.4	C1T0.5	C1T0.6	C1T0.8	C1T1.0
ALLCE	[692.43; 3756.93]	[692.43; Inf)	[692.43; Inf)	[692.43; Inf)	[692.43; Inf)	[692.43; Inf)	[692.43; Inf)
AVESA	[1053.58; 2425.4]	[1053.58; 2425.4]	[1053.58; 2425.4]	[1053.58; 2425.4]	[1053.58; 2425.4]	[1053.58; 2425.4]	[1053.58; Inf)
BEAVA	[869.57; Inf)	[869.57; Inf)	[869.57; Inf)	[869.57; Inf)	[869.57; Inf)	[869.57; Inf)	[869.57; Inf)
BRSNW	[869.57; Inf)	[869.57; Inf)	[869.57; Inf)	[869.57; Inf)	[869.57; Inf)	[869.57; Inf)	[869.57; Inf)
CUMSA	[869.57; Inf)	[869.57; Inf)	[869.57; Inf)	[869.57; Inf)	[869.57; Inf)	[869.57; Inf)	[869.57; Inf)
GLXMA	[869.57; Inf)	[869.57; Inf)	[869.57; Inf)	[869.57; Inf)	[869.57; Inf)	[869.57; Inf)	[869.57; Inf)
HELAN	[869.57; Inf)	[869.57; Inf)	[869.57; Inf)	[869.57; Inf)	[869.57; Inf)	[869.57; Inf)	[869.57; Inf)
LYPES	[869.57; Inf)	[869.57; Inf)	[869.57; Inf)	[869.57; Inf)	[869.57; Inf)	[869.57; Inf)	[869.57; Inf)
TRZAW	[1879.3; 2909.46]	[1879.3; Inf)	[1879.3; Inf)	[1879.3; Inf)	[1879.3; Inf)	[1879.3; Inf)	[1879.3; Inf)
ZEAMA	[2000; Inf)	[2000; Inf)	[2000; Inf)	[2000; Inf)	[2000; Inf)	[2000; Inf)	[2000; Inf)

Table 5: Censored ER_{50} according to criterion 2 and the seven decision thresholds - emergence endpoint of the SE test of study 1

species	C2T0.0	C2T0.2	C2T0.4	C2T0.5	C2T0.6	C2T0.8	C2T1.0
ALLCE	[692.43; 3756.93]	[692.43; Inf)	[692.43; Inf)	[692.43; Inf)	[692.43; Inf)	[692.43; Inf)	[692.43; Inf)
AVESA	[1053.58; 2425.4]	[1053.58; 2425.4]	[1053.58; 2425.4]	[1053.58; 2425.4]	[1053.58; 2425.4]	[1053.58; Inf)	[1053.58; Inf)
BEAVA	[869.57; Inf)	[869.57; Inf)	[869.57; Inf)	[869.57; Inf)	[869.57; Inf)	[869.57; Inf)	[869.57; Inf)
BRSNW	[869.57; Inf)	[869.57; Inf)	[869.57; Inf)	[869.57; Inf)	[869.57; Inf)	[869.57; Inf)	[869.57; Inf)
CUMSA	[869.57; Inf)	[869.57; Inf)	[869.57; Inf)	[869.57; Inf)	[869.57; Inf)	[869.57; Inf)	[869.57; Inf)
GLXMA	[869.57; Inf)	[869.57; Inf)	[869.57; Inf)	[869.57; Inf)	[869.57; Inf)	[869.57; Inf)	[869.57; Inf)
HELAN	[869.57; Inf)	[869.57; Inf)	[869.57; Inf)	[869.57; Inf)	[869.57; Inf)	[869.57; Inf)	[869.57; Inf)
LYPES	[869.57; Inf)	[869.57; Inf)	[869.57; Inf)	[869.57; Inf)	[869.57; Inf)	[869.57; Inf)	[869.57; Inf)
TRZAW	[1879.3; 2909.46]	[1879.3; Inf)	[1879.3; Inf)	[1879.3; Inf)	[1879.3; Inf)	[1879.3; Inf)	[1879.3; Inf)
ZEAMA	[2000; Inf)	[2000; Inf)	[2000; Inf)	[2000; Inf)	[2000; Inf)	[2000; Inf)	[2000; Inf)

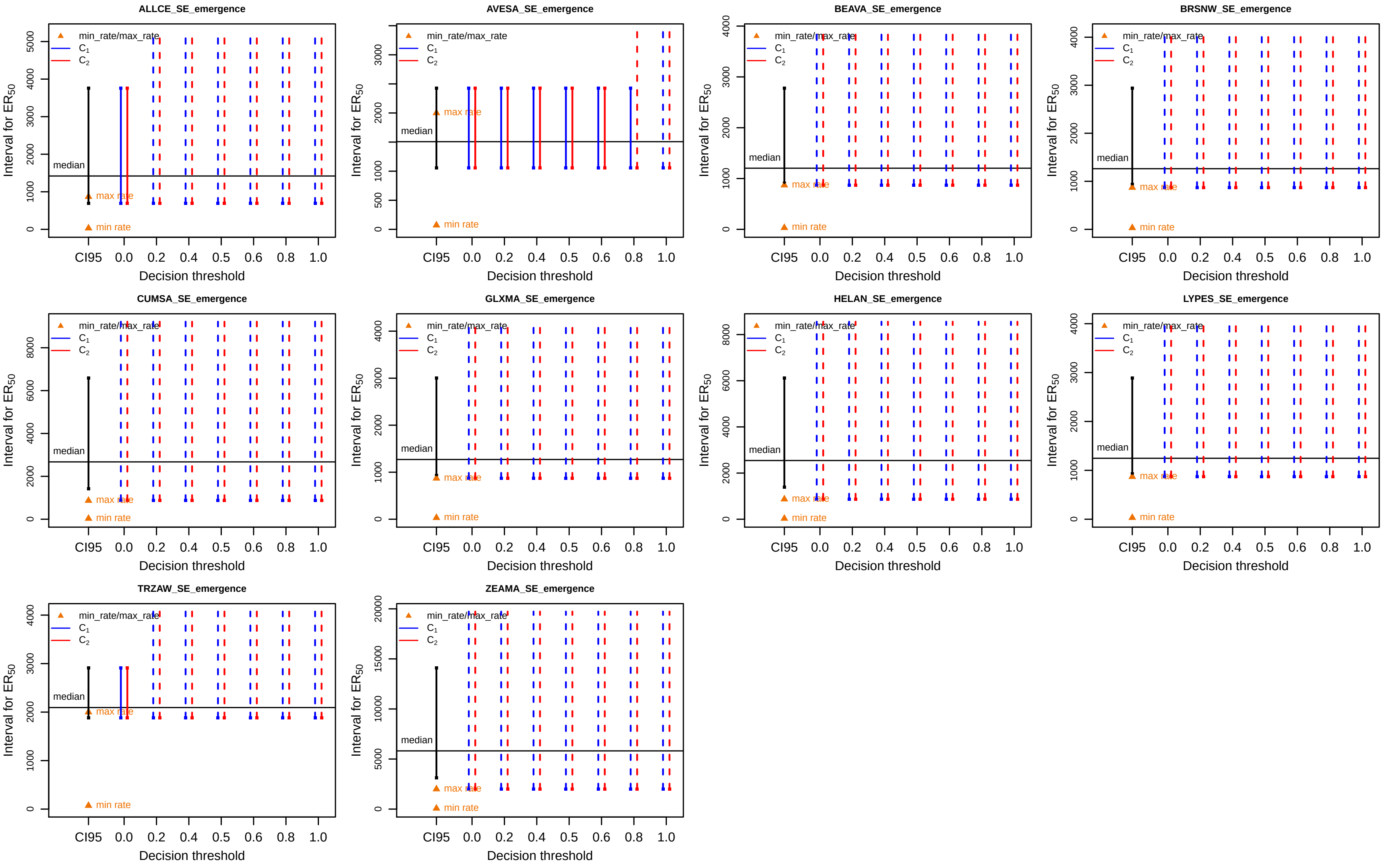

Figure 1: CI95 of ER_{50} and censored ER_{50} according to both criteria and the seven decision thresholds for all species - emergence endpoint of the SE test of study 1. The two orange triangles stand for the lowest and the highest tested rates. Vertical segments of three different colors (black, blue, red) represent the CI95 of ER_{50}, the censored ER_{50} according to C_1 and the censored ER_{50} according to C_2, respectively; solid vertical segments are for bounded intervals while dotted vertical segments stand for right-unbounded intervals; the black horizontal line represents the median of the ER_{50}.

SE test - survival endpoint

Table 6: Minimum and maximum tested rates, median, quantile 2.5% and quantile 97.5% of the ER_{50} posterior distribution - survival endpoint of the SE test of study 1

species	min_rate	max_rate	ER_{50}_median	ER_{50}_LCI95	ER_{50}_UCI95
ALLCE	31.07	869.57	163.0892	128.5010	206.3729
AVESA	71.47	2000.00	264.4067	201.1559	352.9132
BEAVA	31.07	869.57	368.2296	300.3761	448.5027
BRSNW	31.07	869.57	539.8981	444.0571	661.1070
CUMSA	31.07	869.57	555.7471	430.3477	754.0101
GLXMA	31.07	869.57	1270.8044	931.2418	2845.2630
HELAN	31.07	869.57	1269.0585	930.7878	2936.6626
LYPES	31.07	869.57	1108.0447	901.0288	2353.7261
TRZAW	71.47	2000.00	1938.4264	1369.9977	3381.9892
ZEAMA	71.47	2000.00	2794.5687	2085.5955	6353.9845

Table 7: Censored ER_{50} according to criterion 1 and the seven decision thresholds - survival endpoint of the SE test of study 1

species	C1T0.0	C1T0.2	C1T0.4	C1T0.5	C1T0.6	C1T0.8	C1T1.0
ALLCE	[128.5; 206.37]	[128.5; 206.37]	[128.5; 206.37]	[128.5; 206.37]	[128.5; 206.37]	[128.5; 206.37]	[128.5; 206.37]
AVESA	[201.16; 352.91]	[201.16; 352.91]	[201.16; 352.91]	[201.16; 352.91]	[201.16; 352.91]	[201.16; 352.91]	[201.16; 352.91]
BEAVA	[300.38; 448.5]	[300.38; 448.5]	[300.38; 448.5]	[300.38; 448.5]	[300.38; 448.5]	[300.38; 448.5]	[300.38; 448.5]
BRSNW	[444.06; 661.11]	[444.06; 661.11]	[444.06; 661.11]	[444.06; 661.11]	[444.06; 661.11]	[444.06; 661.11]	[444.06; 661.11]
CUMSA	[430.35; 754.01]	[430.35; 754.01]	[430.35; 754.01]	[430.35; 754.01]	[430.35; 754.01]	[430.35; 754.01]	[430.35; 754.01]
GLXMA	[869.57; Inf)	[869.57; Inf)	[869.57; Inf)	[869.57; Inf)	[869.57; Inf)	[869.57; Inf)	[869.57; Inf)
HELAN	[869.57; Inf)	[869.57; Inf)	[869.57; Inf)	[869.57; Inf)	[869.57; Inf)	[869.57; Inf)	[869.57; Inf)
LYPES	[869.57; Inf)	[869.57; Inf)	[869.57; Inf)	[869.57; Inf)	[869.57; Inf)	[869.57; Inf)	[869.57; Inf)
TRZAW	[1370; 3381.99]	[1370; 3381.99]	[1370; 3381.99]	[1370; 3381.99]	[1370; Inf)	[1370; Inf)	[1370; Inf)
ZEAMA	[2000; Inf)	[2000; Inf)	[2000; Inf)	[2000; Inf)	[2000; Inf)	[2000; Inf)	[2000; Inf)

Table 8: Censored ER_{50} according to criterion 2 and the seven decision thresholds - survival endpoint of the SE test of study 1

species	C2T0.0	C2T0.2	C2T0.4	C2T0.5	C2T0.6	C2T0.8	C2T1.0
ALLCE	[128.5; 206.37]	[128.5; 206.37]	[128.5; 206.37]	[128.5; 206.37]	[128.5; 206.37]	[128.5; 206.37]	[128.5; 206.37]
AVESA	[201.16; 352.91]	[201.16; 352.91]	[201.16; 352.91]	[201.16; 352.91]	[201.16; 352.91]	[201.16; 352.91]	[201.16; 352.91]
BEAVA	[300.38; 448.5]	[300.38; 448.5]	[300.38; 448.5]	[300.38; 448.5]	[300.38; 448.5]	[300.38; 448.5]	[300.38; 448.5]
BRSNW	[444.06; 661.11]	[444.06; 661.11]	[444.06; 661.11]	[444.06; 661.11]	[444.06; 661.11]	[444.06; 661.11]	[444.06; 661.11]
CUMSA	[430.35; 754.01]	[430.35; 754.01]	[430.35; 754.01]	[430.35; 754.01]	[430.35; 754.01]	[430.35; 754.01]	[430.35; 754.01]
GLXMA	[869.57; Inf)	[869.57; Inf)	[869.57; Inf)	[869.57; Inf)	[869.57; Inf)	[869.57; Inf)	[869.57; Inf)
HELAN	[869.57; Inf)	[869.57; Inf)	[869.57; Inf)	[869.57; Inf)	[869.57; Inf)	[869.57; Inf)	[869.57; Inf)
LYPES	[869.57; Inf)	[869.57; Inf)	[869.57; Inf)	[869.57; Inf)	[869.57; Inf)	[869.57; Inf)	[869.57; Inf)
TRZAW	[1370; 3381.99]	[1370; 3381.99]	[1370; Inf)	[1370; Inf)	[1370; Inf)	[1370; Inf)	[1370; Inf)
ZEAMA	[2000; Inf)	[2000; Inf)	[2000; Inf)	[2000; Inf)	[2000; Inf)	[2000; Inf)	[2000; Inf)

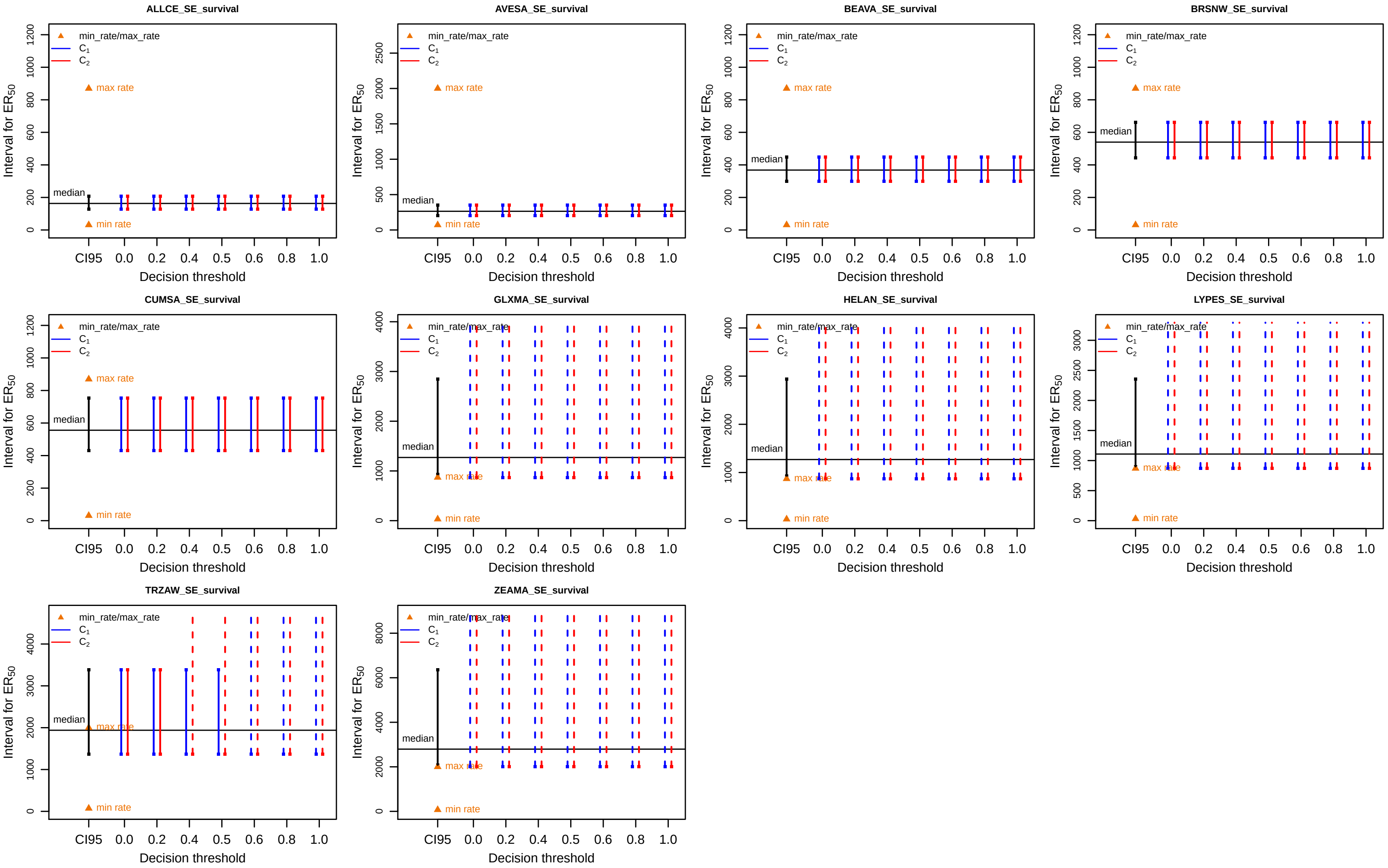

Figure 2: CI95 of ER_{50} and censored ER_{50} according to both criteria and the seven decision thresholds for all species - survival endpoint of the SE test of study 1. The two orange triangles stand for the lowest and the highest tested rates. Vertical segments of three different colors (black, blue, red) represent the CI95 of ER_{50}, the censored ER_{50} according to C_1 and the censored ER_{50} according to C_2, respectively; solid vertical segments are for bounded intervals while dotted vertical segments stand for right-unbounded intervals; the black horizontal line represents the median of the ER_{50}.

SE test - shoot dry weight endpoint

Table 9: Minimum and maximum tested rates, median, quantile 2.5% and quantile 97.5% of the ER_{50} posterior distribution - shoot dry weight endpoint of the SE test of study 1

species	min_rate	max_rate	ER_{50}_median	ER_{50}_LCI95	ER_{50}_UCI95
ALLCE	31.07	378.07	155.8689	106.6271	222.6088
AVESA	71.47	869.57	183.3126	142.1188	244.2603
BEAVA	31.07	378.07	283.2377	230.0457	351.8027
BRSNW	31.07	869.57	321.0205	272.8069	372.1631
CUMSA	31.07	869.57	275.3910	236.3495	321.3627
GLXMA	31.07	869.57	1215.5481	913.7874	2761.6941
HELAN	31.07	869.57	1209.7758	911.7498	2903.6297
LYPES	31.07	869.57	597.0532	507.2770	701.1788
TRZAW	71.47	2000.00	670.0644	532.6734	841.1321
ZEAMA	71.47	2000.00	1992.5124	1312.3398	3350.1970

Table 10: Censored ER_{50} according to criterion 1 and the seven decision thresholds - shoot dry weight endpoint of the SE test of study 1

species	C1T0.0	C1T0.2	C1T0.4	C1T0.5	C1T0.6	C1T0.8	C1T1.0
ALLCE	[106.63; 222.61]	[106.63; 222.61]	[106.63; 222.61]	[106.63; 222.61]	[106.63; 222.61]	[106.63; 222.61]	[106.63; 222.61]
AVESA	[142.12; 244.26]	[142.12; 244.26]	[142.12; 244.26]	[142.12; 244.26]	[142.12; 244.26]	[142.12; 244.26]	[142.12; 244.26]
BEAVA	[230.05; 351.8]	[230.05; 351.8]	[230.05; 351.8]	[230.05; 351.8]	[230.05; 351.8]	[230.05; 351.8]	[230.05; 351.8]
BRSNW	[272.81; 372.16]	[272.81; 372.16]	[272.81; 372.16]	[272.81; 372.16]	[272.81; 372.16]	[272.81; 372.16]	[272.81; 372.16]
CUMSA	[236.35; 321.36]	[236.35; 321.36]	[236.35; 321.36]	[236.35; 321.36]	[236.35; 321.36]	[236.35; 321.36]	[236.35; 321.36]
GLXMA	[869.57; Inf)	[869.57; Inf)	[869.57; Inf)	[869.57; Inf)	[869.57; Inf)	[869.57; Inf)	[869.57; Inf)
HELAN	[869.57; Inf)	[869.57; Inf)	[869.57; Inf)	[869.57; Inf)	[869.57; Inf)	[869.57; Inf)	[869.57; Inf)
LYPES	[507.28; 701.18]	[507.28; 701.18]	[507.28; 701.18]	[507.28; 701.18]	[507.28; 701.18]	[507.28; 701.18]	[507.28; 701.18]
TRZAW	[532.67; 841.13]	[532.67; 841.13]	[532.67; 841.13]	[532.67; 841.13]	[532.67; 841.13]	[532.67; 841.13]	[532.67; 841.13]
ZEAMA	[1312.34; 3350.2]	[1312.34; 3350.2]	[1312.34; 3350.2]	[1312.34; 3350.2]	[1312.34; Inf)	[1312.34; Inf)	[1312.34; Inf)

Table 11: Censored ER_{50} according to criterion 2 and the seven decision thresholds - shoot dry weight endpoint of the SE test of study 1

species	C2T0.0	C2T0.2	C2T0.4	C2T0.5	C2T0.6	C2T0.8	C2T1.0
ALLCE	[106.63; 222.61]	[106.63; 222.61]	[106.63; 222.61]	[106.63; 222.61]	[106.63; 222.61]	[106.63; 222.61]	[106.63; 222.61]
AVESA	[142.12; 244.26]	[142.12; 244.26]	[142.12; 244.26]	[142.12; 244.26]	[142.12; 244.26]	[142.12; 244.26]	[142.12; 244.26]
BEAVA	[230.05; 351.8]	[230.05; 351.8]	[230.05; 351.8]	[230.05; 351.8]	[230.05; 351.8]	[230.05; 351.8]	[230.05; 351.8]
BRSNW	[272.81; 372.16]	[272.81; 372.16]	[272.81; 372.16]	[272.81; 372.16]	[272.81; 372.16]	[272.81; 372.16]	[272.81; 372.16]
CUMSA	[236.35; 321.36]	[236.35; 321.36]	[236.35; 321.36]	[236.35; 321.36]	[236.35; 321.36]	[236.35; 321.36]	[236.35; 321.36]
GLXMA	[869.57; Inf)	[869.57; Inf)	[869.57; Inf)	[869.57; Inf)	[869.57; Inf)	[869.57; Inf)	[869.57; Inf)
HELAN	[869.57; Inf)	[869.57; Inf)	[869.57; Inf)	[869.57; Inf)	[869.57; Inf)	[869.57; Inf)	[869.57; Inf)
LYPES	[507.28; 701.18]	[507.28; 701.18]	[507.28; 701.18]	[507.28; 701.18]	[507.28; 701.18]	[507.28; 701.18]	[507.28; 701.18]
TRZAW	[532.67; 841.13]	[532.67; 841.13]	[532.67; 841.13]	[532.67; 841.13]	[532.67; 841.13]	[532.67; 841.13]	[532.67; 841.13]
ZEAMA	[1312.34; 3350.2]	[1312.34; 3350.2]	[1312.34; Inf)	[1312.34; Inf)	[1312.34; Inf)	[1312.34; Inf)	[1312.34; Inf)

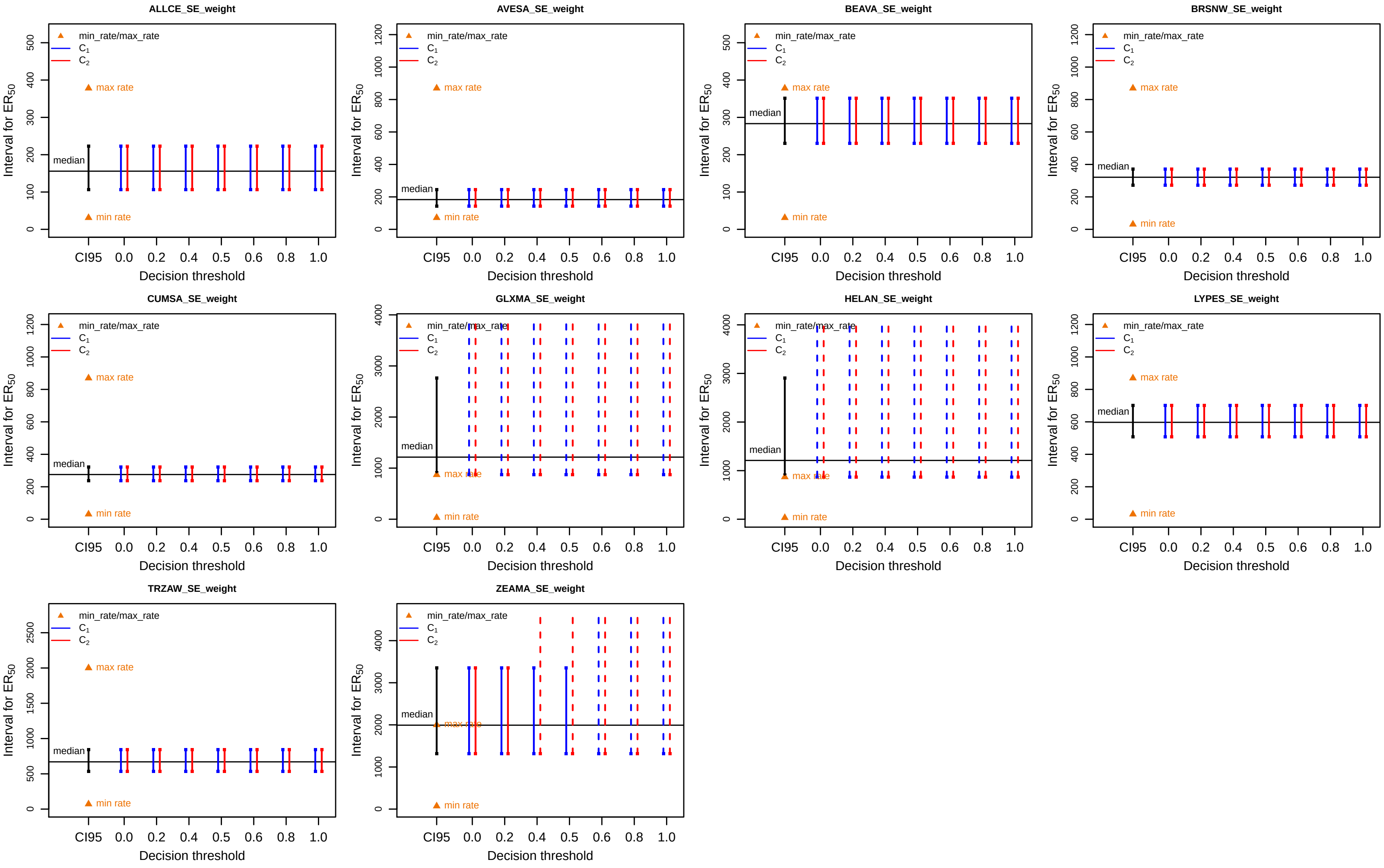

Figure 3: CI95 of ER_{50} and censored ER_{50} according to both criteria and the seven decision thresholds for all species - shoot dry weight endpoint of the SE test of study 1. The two orange triangles stand for the lowest and the highest tested rates. Vertical segments of three different colors (black, blue, red) represent the CI95 of ER_{50}, the censored ER_{50} according to C_1 and the censored ER_{50} according to C_2, respectively; solid vertical segments are for bounded intervals while dotted vertical segments stand for right-unbounded intervals; the black horizontal line represents the median of the ER_{50}.

Vegatative vigor (VV) test of study 1

Raw data description (test item rates) for VV test

```
Number of species:  10
EPPO code:  ALLCE AVESA BEAVA BRSNW CUMSA GLXMA HELAN LYPES TRZAW ZEAMA
```

Table 12: Corresponding species and common names to the EPPO code

EPPO code	Species name	Common name
ALLCE	*Allium cepa*	Onion
AVESA	*Avena sativa*	Oat
BEAVA	*Beta vulgaris*	Sugar beet
BRSNW	*Brassica napus winter*	Oilseed rape
CUMSA	*Cucumis sativus*	Cucumber
GLXMA	*Glycine max*	Soybean
HELAN	*Helianthus annuus*	Sunflower
LYPES	*Solanum lycopersicum*	Tomato
TRZAW	*Triticum aestivum winter*	Winter wheat
ZEAMA	*Zea mays subs. Amylacea*	Corn

Table 13: Tested rates per species (ml product/ha) for the VV test of study 1

ALLCE	AVESA	BEAVA	BRSNW	CUMSA	GLXMA	HELAN	LYPES	TRZAW	ZEAMA
0.00	0.00	0.00	0.00	0.00	0.00	0.00	0.00	0.00	0.00
31.07	71.47	31.07	31.07	31.07	31.07	31.07	31.07	71.47	71.47
71.47	164.38	71.47	71.47	71.47	71.47	71.47	71.47	164.38	164.38
164.38	378.07	164.38	164.38	164.38	164.38	164.38	164.38	378.07	378.07
378.07	869.57	378.07	378.07	378.07	378.07	378.07	378.07	869.57	869.57
869.57	2000.00	869.57	869.57	869.57	869.57	869.57	869.57	2000.00	2000.00

VV test - survival endpoint

Table 14: Minimum and maximum tested rates, median, quantile 2.5% and quantile 97.5% of the ER_{50} posterior distribution - survival endpoint of the VV test of study 1

species	min_rate	max_rate	ER_{50}_median	ER_{50}_LCI95	ER_{50}_UCI95
ALLCE	31.07	869.57	1268.5992	931.0119	2901.631
AVESA	71.47	2000.00	2920.8134	2147.1338	6425.718
BEAVA	31.07	869.57	1267.0010	933.1524	2950.169
BRSNW	31.07	869.57	956.0488	862.9716	1475.258
CUMSA	31.07	869.57	1113.0925	901.9258	2351.503
GLXMA	31.07	869.57	1272.9332	934.5864	2917.581
HELAN	31.07	869.57	1271.9763	934.9826	2842.151
LYPES	31.07	869.57	1268.7299	933.6295	2893.262
TRZAW	71.47	2000.00	2930.0900	2147.2277	6668.731
ZEAMA	71.47	2000.00	2901.5010	2144.8387	6692.480

Table 15: Censored ER_{50} according to criterion 1 and the seven decision thresholds - survival endpoint of the VV test of study 1

species	C1T0.0	C1T0.2	C1T0.4	C1T0.5	C1T0.6	C1T0.8	C1T1.0
ALLCE	[869.57; Inf)	[869.57; Inf)	[869.57; Inf)	[869.57; Inf)	[869.57; Inf)	[869.57; Inf)	[869.57; Inf)
AVESA	[2000; Inf)	[2000; Inf)	[2000; Inf)	[2000; Inf)	[2000; Inf)	[2000; Inf)	[2000; Inf)
BEAVA	[869.57; Inf)	[869.57; Inf)	[869.57; Inf)	[869.57; Inf)	[869.57; Inf)	[869.57; Inf)	[869.57; Inf)
BRSNW	[862.97; 1475.26]	[862.97; Inf)	[862.97; Inf)	[862.97; Inf)	[862.97; Inf)	[862.97; Inf)	[862.97; Inf)
CUMSA	[869.57; Inf)	[869.57; Inf)	[869.57; Inf)	[869.57; Inf)	[869.57; Inf)	[869.57; Inf)	[869.57; Inf)
GLXMA	[869.57; Inf)	[869.57; Inf)	[869.57; Inf)	[869.57; Inf)	[869.57; Inf)	[869.57; Inf)	[869.57; Inf)
HELAN	[869.57; Inf)	[869.57; Inf)	[869.57; Inf)	[869.57; Inf)	[869.57; Inf)	[869.57; Inf)	[869.57; Inf)
LYPES	[869.57; Inf)	[869.57; Inf)	[869.57; Inf)	[869.57; Inf)	[869.57; Inf)	[869.57; Inf)	[869.57; Inf)
TRZAW	[2000; Inf)	[2000; Inf)	[2000; Inf)	[2000; Inf)	[2000; Inf)	[2000; Inf)	[2000; Inf)
ZEAMA	[2000; Inf)	[2000; Inf)	[2000; Inf)	[2000; Inf)	[2000; Inf)	[2000; Inf)	[2000; Inf)

Table 16: Censored ER_{50} according to criterion 2 and the seven decision thresholds - survival endpoint of the VV test of study 1

species	C2T0.0	C2T0.2	C2T0.4	C2T0.5	C2T0.6	C2T0.8	C2T1.0
ALLCE	[869.57; Inf)	[869.57; Inf)	[869.57; Inf)	[869.57; Inf)	[869.57; Inf)	[869.57; Inf)	[869.57; Inf)
AVESA	[2000; Inf)	[2000; Inf)	[2000; Inf)	[2000; Inf)	[2000; Inf)	[2000; Inf)	[2000; Inf)
BEAVA	[869.57; Inf)	[869.57; Inf)	[869.57; Inf)	[869.57; Inf)	[869.57; Inf)	[869.57; Inf)	[869.57; Inf)
BRSNW	[862.97; 1475.26]	[862.97; Inf)	[862.97; Inf)	[862.97; Inf)	[862.97; Inf)	[862.97; Inf)	[862.97; Inf)
CUMSA	[869.57; Inf)	[869.57; Inf)	[869.57; Inf)	[869.57; Inf)	[869.57; Inf)	[869.57; Inf)	[869.57; Inf)
GLXMA	[869.57; Inf)	[869.57; Inf)	[869.57; Inf)	[869.57; Inf)	[869.57; Inf)	[869.57; Inf)	[869.57; Inf)
HELAN	[869.57; Inf)	[869.57; Inf)	[869.57; Inf)	[869.57; Inf)	[869.57; Inf)	[869.57; Inf)	[869.57; Inf)
LYPES	[869.57; Inf)	[869.57; Inf)	[869.57; Inf)	[869.57; Inf)	[869.57; Inf)	[869.57; Inf)	[869.57; Inf)
TRZAW	[2000; Inf)	[2000; Inf)	[2000; Inf)	[2000; Inf)	[2000; Inf)	[2000; Inf)	[2000; Inf)
ZEAMA	[2000; Inf)	[2000; Inf)	[2000; Inf)	[2000; Inf)	[2000; Inf)	[2000; Inf)	[2000; Inf)

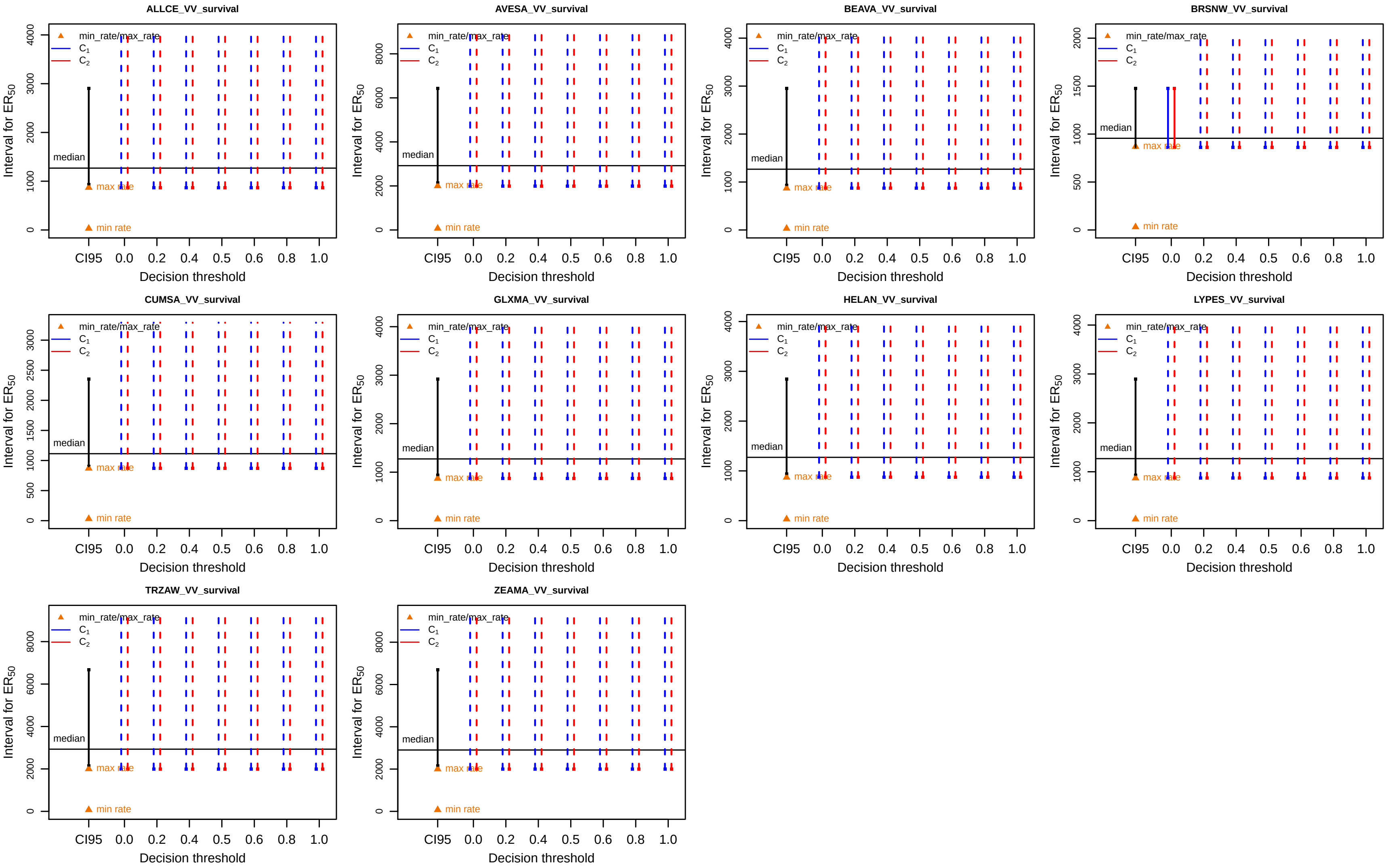

Figure 4: CI95 of ER_{50} and censored ER_{50} according to both criteria and the seven decision thresholds for all species - survival endpoint of the VV test of study 1. The two orange triangles stand for the lowest and the highest tested rates. Vertical segments of three different colors (black, blue, red) represent the CI95 of ER_{50}, the censored ER_{50} according to C_1 and the censored ER_{50} according to C_2, respectively; solid vertical segments are for bounded intervals while dotted vertical segments stand for right-unbounded intervals; the black horizontal line represents the median of the ER_{50}.

VV test - shoot dry weight endpoint

Table 17: Minimum and maximum tested rates, median, quantile 2.5% and quantile 97.5% of the ER_{50} posterior distribution - shoot dry weight endpoint of the VV test of study 1

species	min_rate	max_rate	ER_{50}_median	ER_{50}_LCI95	ER_{50}_UCI95
ALLCE	31.07	869.57	656.8173	76.04318	1945.3813
AVESA	71.47	2000.00	2235.4933	1330.00639	3967.5197
BEAVA	31.07	869.57	538.5002	426.64664	681.0293
BRSNW	31.07	869.57	166.3894	134.76212	204.4930
CUMSA	31.07	869.57	281.4183	177.97705	460.0662
GLXMA	31.07	869.57	443.8683	346.20412	577.4326
HELAN	31.07	869.57	685.7885	510.86219	955.9358
LYPES	31.07	869.57	1316.7886	958.54475	1924.9520
TRZAW	71.47	2000.00	3874.8076	2129.18665	7560.3578
ZEAMA	71.47	2000.00	6788.7400	4142.47676	13203.0575

Table 18: Censored ER_{50} according to criterion 1 and the seven decision thresholds - shoot dry weight endpoint of the VV test of study 1

species	C1T0.0	C1T0.2	C1T0.4	C1T0.5	C1T0.6	C1T0.8	C1T1.0
ALLCE	[76.04; 1945.38]	[76.04; 1945.38]	[76.04; 1945.38]	[76.04; 1945.38]	[76.04; 1945.38]	[76.04; Inf)	[76.04; Inf)
AVESA	[1330.01; 3967.52]	[1330.01; 3967.52]	[1330.01; Inf)	[1330.01; Inf)	[1330.01; Inf)	[1330.01; Inf)	[1330.01; Inf)
BEAVA	[426.65; 681.03]	[426.65; 681.03]	[426.65; 681.03]	[426.65; 681.03]	[426.65; 681.03]	[426.65; 681.03]	[426.65; 681.03]
BRSNW	[134.76; 204.49]	[134.76; 204.49]	[134.76; 204.49]	[134.76; 204.49]	[134.76; 204.49]	[134.76; 204.49]	[134.76; 204.49]
CUMSA	[177.98; 460.07]	[177.98; 460.07]	[177.98; 460.07]	[177.98; 460.07]	[177.98; 460.07]	[177.98; 460.07]	[177.98; 460.07]
GLXMA	[346.2; 577.43]	[346.2; 577.43]	[346.2; 577.43]	[346.2; 577.43]	[346.2; 577.43]	[346.2; 577.43]	[346.2; 577.43]
HELAN	[510.86; 955.94]	[510.86; 955.94]	[510.86; 955.94]	[510.86; 955.94]	[510.86; 955.94]	[510.86; 955.94]	[510.86; Inf)
LYPES	[869.57; Inf)	[869.57; Inf)	[869.57; Inf)	[869.57; Inf)	[869.57; Inf)	[869.57; Inf)	[869.57; Inf)
TRZAW	[2000; Inf)	[2000; Inf)	[2000; Inf)	[2000; Inf)	[2000; Inf)	[2000; Inf)	[2000; Inf)
ZEAMA	[2000; Inf)	[2000; Inf)	[2000; Inf)	[2000; Inf)	[2000; Inf)	[2000; Inf)	[2000; Inf)

Table 19: Censored ER_{50} according to criterion 2 and the seven decision thresholds - shoot dry weight endpoint of the VV test of study 1

species	C2T0.0	C2T0.2	C2T0.4	C2T0.5	C2T0.6	C2T0.8	C2T1.0
ALLCE	[76.04; 1945.38]	[76.04; 1945.38]	[76.04; 1945.38]	[76.04; Inf)	[76.04; Inf)	[76.04; Inf)	[76.04; Inf)
AVESA	[1330.01; 3967.52]	[1330.01; 3967.52]	[1330.01; Inf)	[1330.01; Inf)	[1330.01; Inf)	[1330.01; Inf)	[1330.01; Inf)
BEAVA	[426.65; 681.03]	[426.65; 681.03]	[426.65; 681.03]	[426.65; 681.03]	[426.65; 681.03]	[426.65; 681.03]	[426.65; 681.03]
BRSNW	[134.76; 204.49]	[134.76; 204.49]	[134.76; 204.49]	[134.76; 204.49]	[134.76; 204.49]	[134.76; 204.49]	[134.76; 204.49]
CUMSA	[177.98; 460.07]	[177.98; 460.07]	[177.98; 460.07]	[177.98; 460.07]	[177.98; 460.07]	[177.98; 460.07]	[177.98; 460.07]
GLXMA	[346.2; 577.43]	[346.2; 577.43]	[346.2; 577.43]	[346.2; 577.43]	[346.2; 577.43]	[346.2; 577.43]	[346.2; 577.43]
HELAN	[510.86; 955.94]	[510.86; 955.94]	[510.86; 955.94]	[510.86; 955.94]	[510.86; 955.94]	[510.86; 955.94]	[510.86; Inf)
LYPES	[869.57; Inf)	[869.57; Inf)	[869.57; Inf)	[869.57; Inf)	[869.57; Inf)	[869.57; Inf)	[869.57; Inf)
TRZAW	[2000; Inf)	[2000; Inf)	[2000; Inf)	[2000; Inf)	[2000; Inf)	[2000; Inf)	[2000; Inf)
ZEAMA	[2000; Inf)	[2000; Inf)	[2000; Inf)	[2000; Inf)	[2000; Inf)	[2000; Inf)	[2000; Inf)

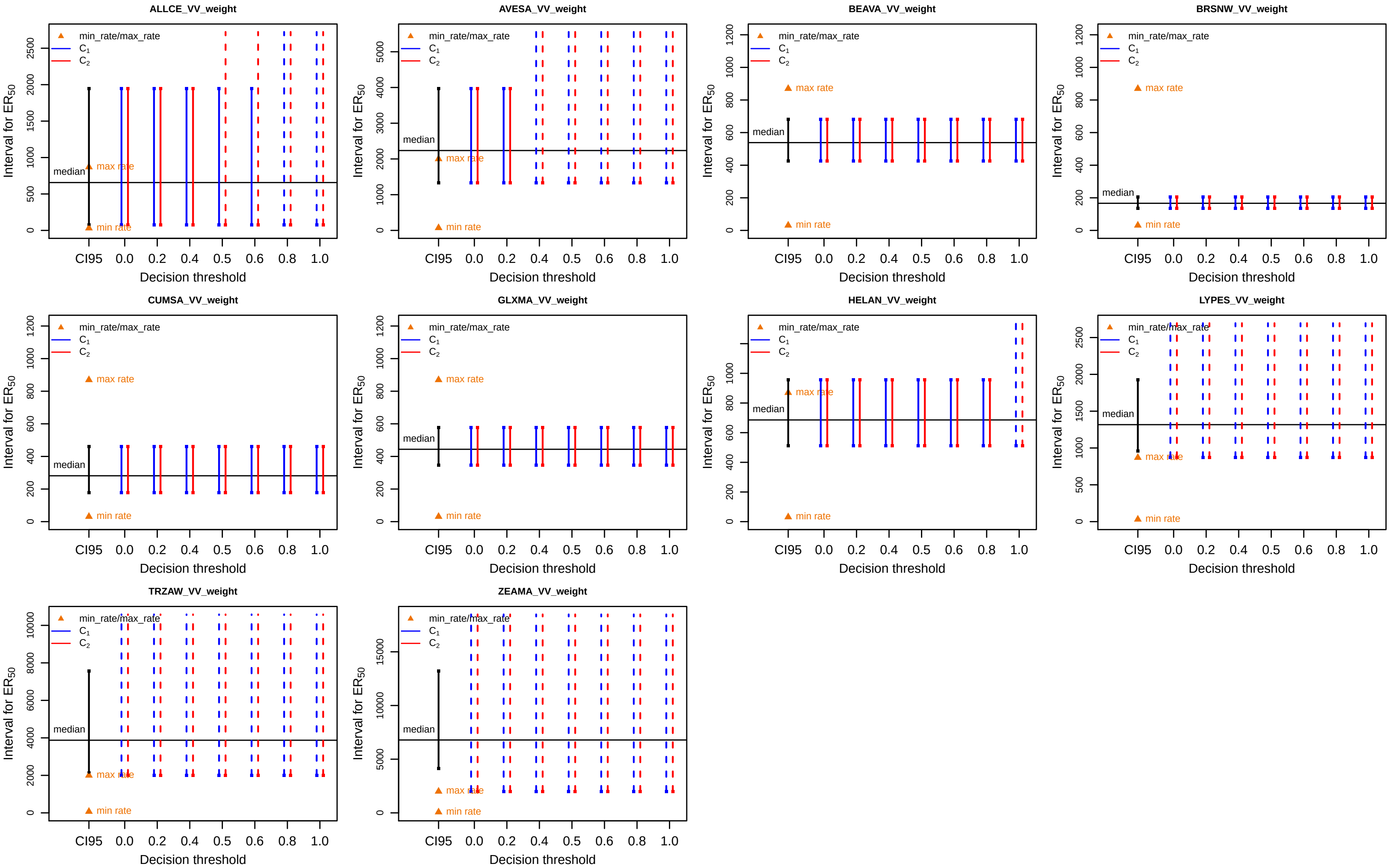

Figure 5: CI95 of ER_{50} and censored ER_{50} according to both criteria and the seven decision thresholds for all species - shoot dry weight endpoint of the VV test of study 1. The two orange triangles stand for the lowest and the highest tested rates. Vertical segments of three different colors (black, blue, red) represent the CI95 of ER_{50}, the censored ER_{50} according to C_1 and the censored ER_{50} according to C_2, respectively; solid vertical segments are for bounded intervals while dotted vertical segments stand for right-unbounded intervals; the black horizontal line represents the median of the ER_{50}.
