# Supplementary material for: How to account for the uncertainty from standard toxicity tests in species sensitivity distributions: An example in non-target plants
Source: PLoS One. 2021 Jan 7;16(1):e0245071. doi: 10.1371/journal.pone.0245071 (PMC7790375; doi:10.1371/journal.pone.0245071)
Supplement: S1 Archive — It is a zip file containing seven folders (one folder per case study). Each folder contains five files report_xxx.pdf with detailed results of the dose-response analyses, one file corresponding to does-response analysis per endpoint. It also contains one file ER50_censoring.pdf for censored ER50 and one file SSD_analyses.pdf for results of SSD analyses. (ZIP) [file pone.0245071.s004.zip › S1_archive/Study1/report_SE_emergence.pdf]

Dose-response analyses

Study 1

Seedling Emergence test - emergence endpoint

25 June 2020

Contact: sandrine.charles@univ-lyon1.fr

This is a report which provides results on all performed dose-response analyses for the emergence endpoint of the Seedling Emergence test for study 1.

Contents

Data set: ALLCE_SE_emergence . . . 2
Data set: AVESA_SE_emergence . . . 3
Data set: BEAVA_SE_emergence . . . 4
Data set: BRSNW_SE_emergence . . . 5
Data set: CUMSA_SE_emergence . . . 6
Data set: GLXMA_SE_emergence . . . 7
Data set: HELAN_SE_emergence . . . 8
Data set: LYPES_SE_emergence . . . 9
Data set: TRZAW_SE_emergence . . . 10
Data set: ZEAMA_SE_emergence . . . 11

Data set: ALLCE_SE_emergence

Table 1: Summary of parameter estimates (parameter d is set to 1) for ALLCE_SE_emergence data set

Parameter	median	Q2.5	Q97.5
b	0.627	0.380	0.969
e	1420.196	692.429	3756.931

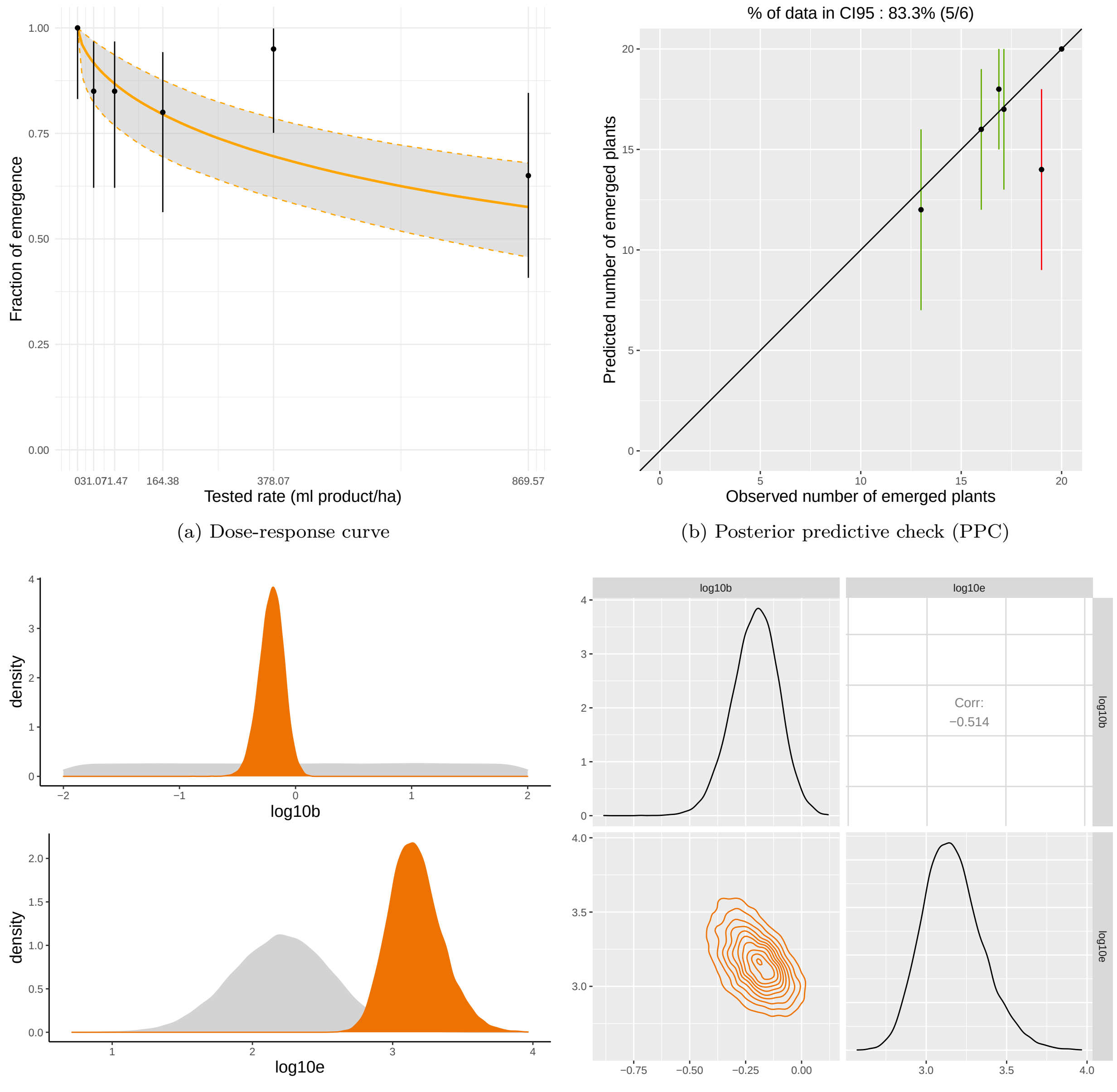

(a) Dose-response curve

(b) Posterior predictive check (PPC)

(c) Priors and posteriors

(d) Correlations between parameters

Figure 1: Dose-response curve (a), PPC (b), prior and posterior distributions (c) and correlations between parameters (d).

Data set: AVESA_SE_emergence

Table 2: Summary of parameter estimates for AVESA_SE_emergence data set

Parameter	median	Q2.5	Q97.5
b	1.753	1.041	3.069
d	0.968	0.903	0.996
e	1506.596	1053.582	2425.402

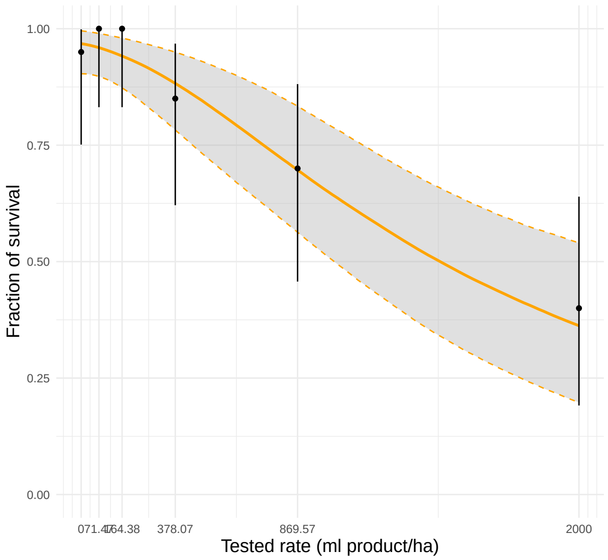

(a) Dose-response curve

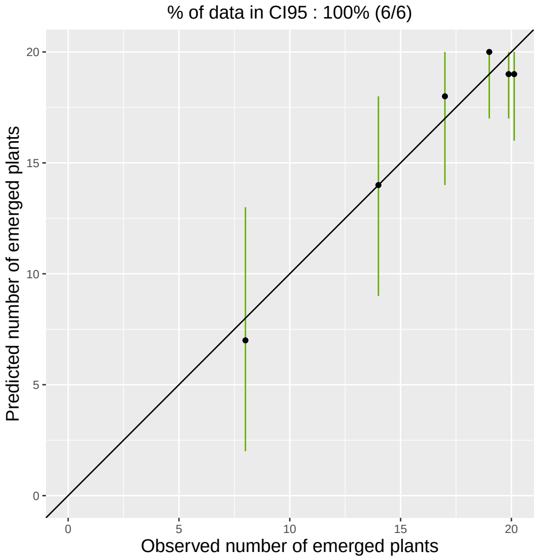

(b) Posterior predictive check (PPC)

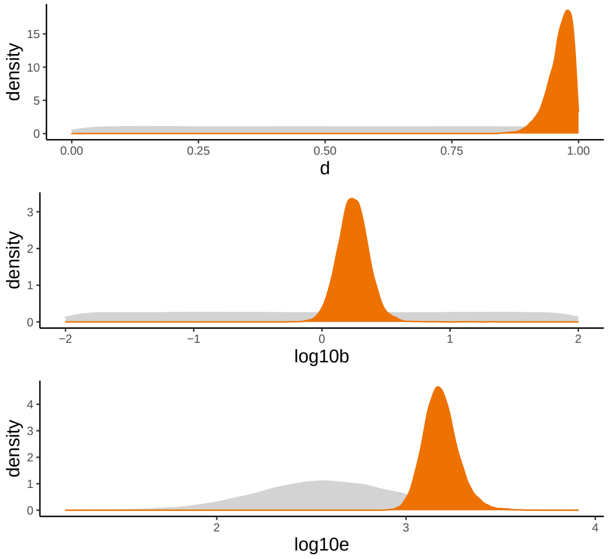

(c) Priors and posteriors

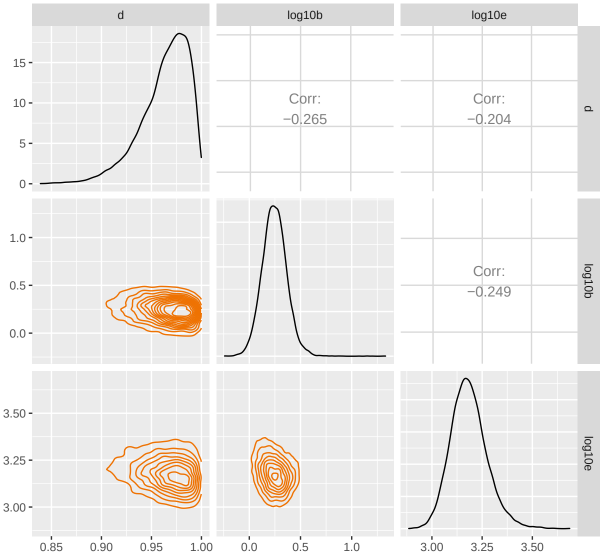

(d) Correlations between parameters

Figure 2: Dose-response curve (a), PPC (b), prior and posterior distributions (c) and correlations between parameters (d).

Data set: BEAVA_SE_emergence

Table 3: Summary of parameter estimates for BEAVA_SE_emergence data set

Parameter	median	Q2.5	Q97.5
b	21.306	2.437	92.630
d	0.950	0.899	0.982
e	1203.154	908.450	2777.003

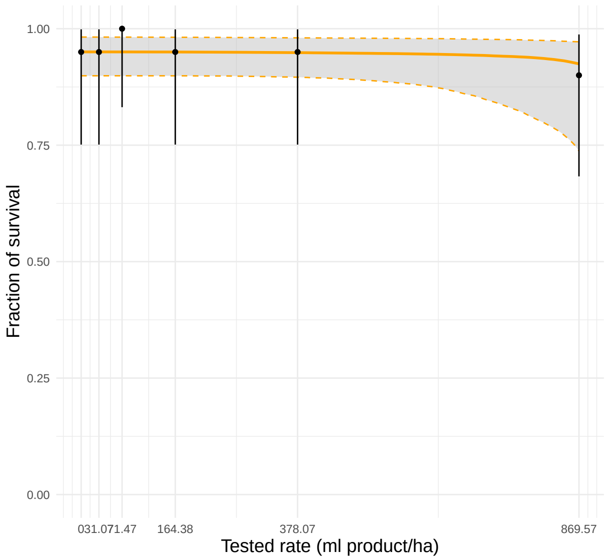

(a) Dose-response curve

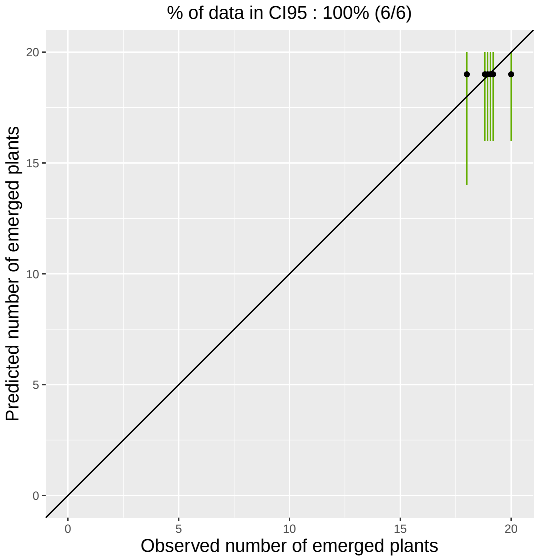

(b) Posterior predictive check (PPC)

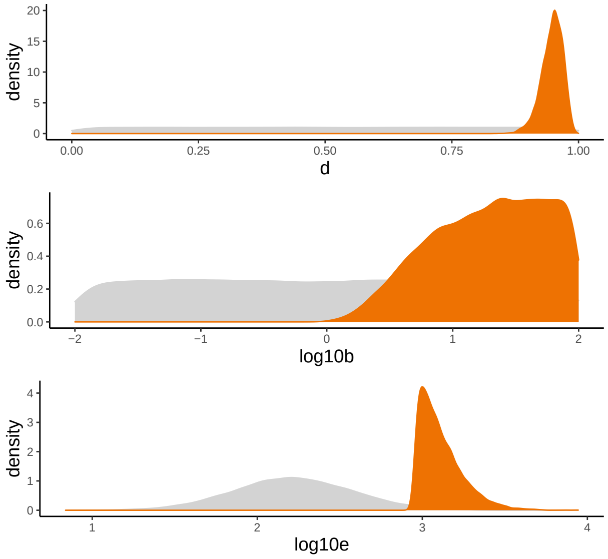

(c) Priors and posteriors

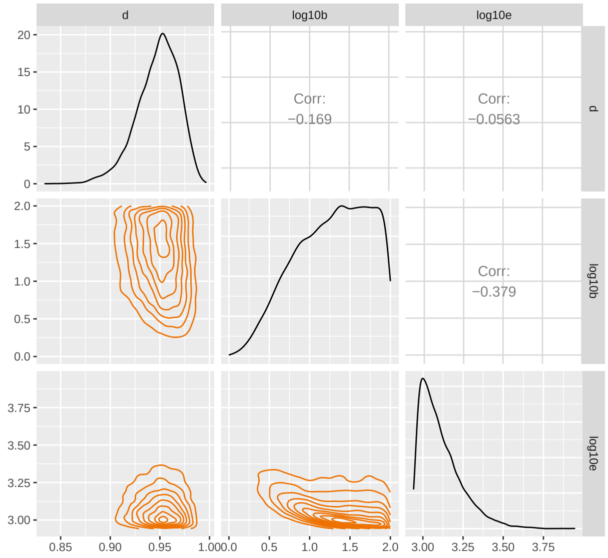

(d) Correlations between parameters

Figure 3: Dose-response curve (a), PPC (b), prior and posterior distributions (c) and correlations between parameters (d).

Data set: BRSNW_SE_emergence

Table 4: Summary of parameter estimates for BRSNW_SE_emergence data set

Parameter	median	Q2.5	Q97.5
b	34.011	5.055	94.975
d	0.970	0.929	0.991
e	1262.485	932.602	2937.444

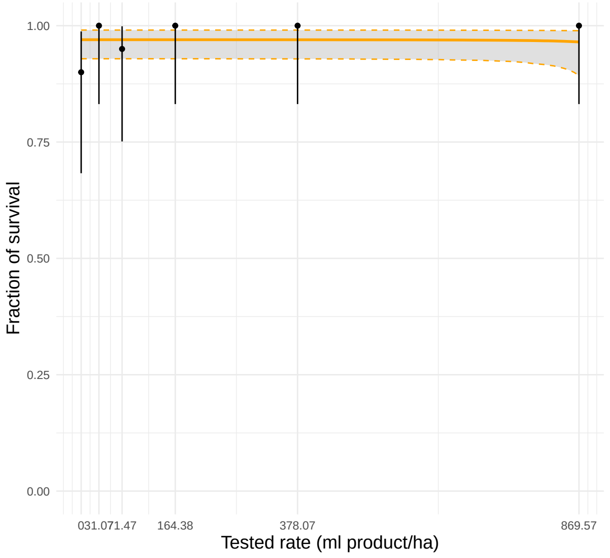

(a) Dose-response curve

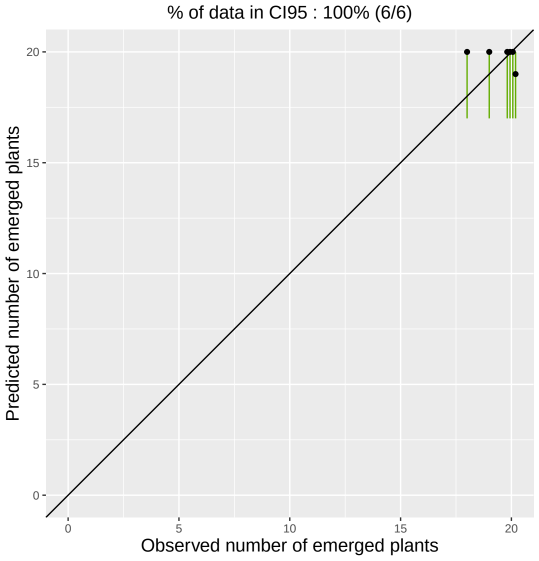

(b) Posterior predictive check (PPC)

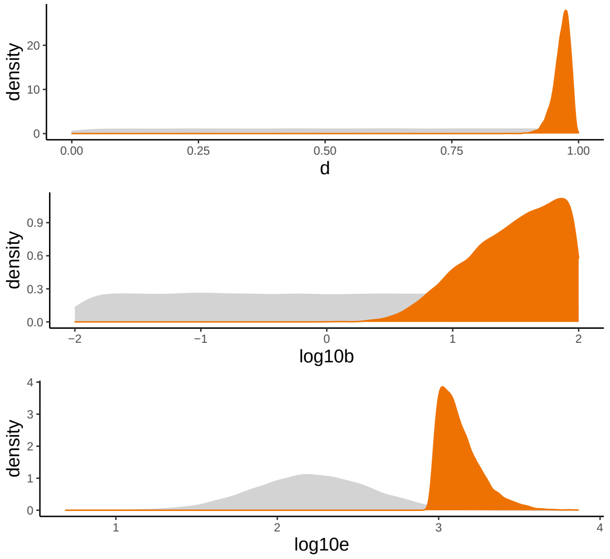

(c) Priors and posteriors

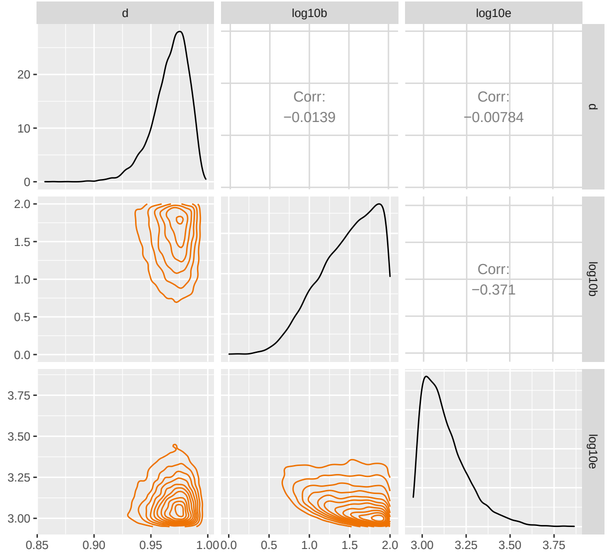

(d) Correlations between parameters

Figure 4: Dose-response curve (a), PPC (b), prior and posterior distributions (c) and correlations between parameters (d).

Data set: CUMSA_SE_emergence

Table 5: Summary of parameter estimates (parameter d is set to 1) for CUMSA_SE_emergence data set

Parameter	median	Q2.5	Q97.5
b	1.520	0.929	2.518
e	2674.779	1416.812	6583.219

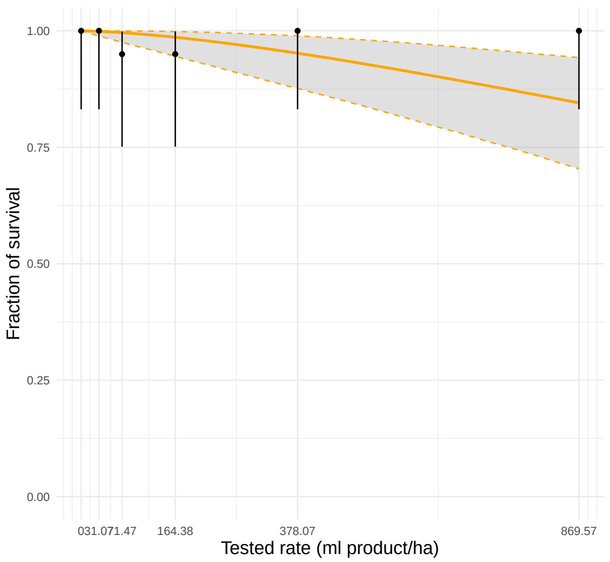

(a) Dose-response curve

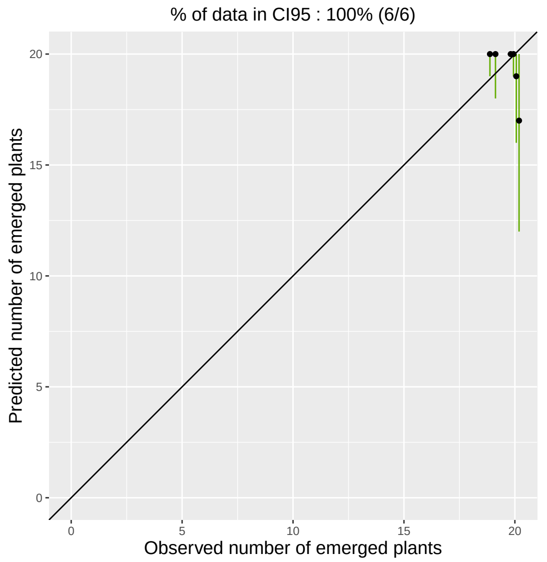

(b) Posterior predictive check (PPC)

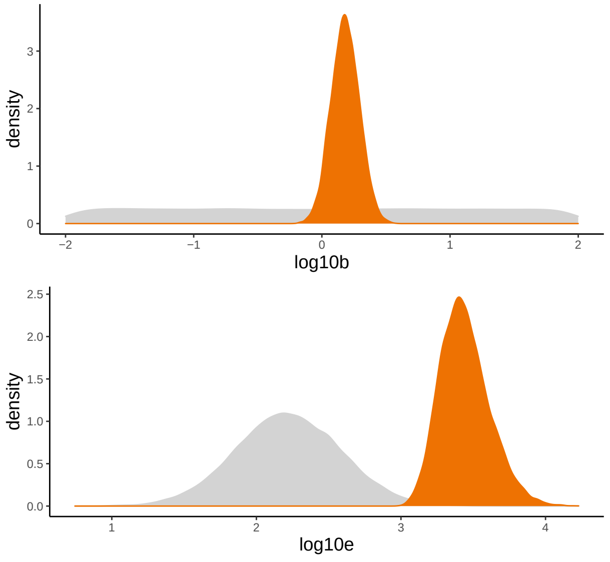

(c) Priors and posteriors

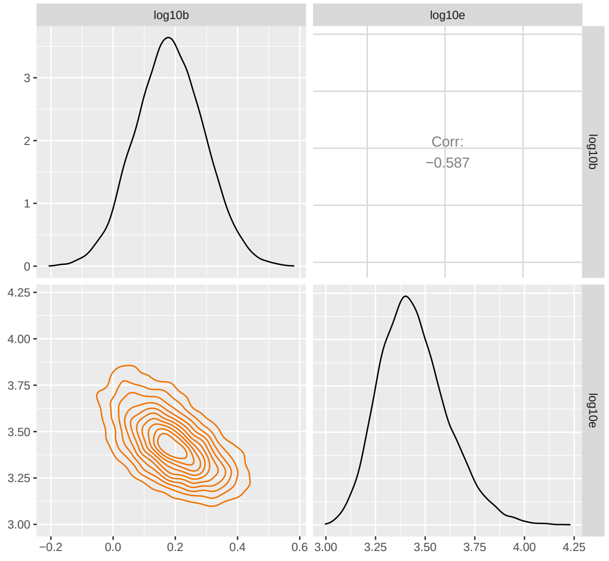

(d) Correlations between parameters

Figure 5: Dose-response curve (a), PPC (b), prior and posterior distributions (c) and correlations between parameters (d).

Data set: GLXMA_SE_emergence

Table 6: Summary of parameter estimates (parameter d is set to 1) for GLXMA_SE_emergence data set

Parameter	median	Q2.5	Q97.5
b	34.273	5.004	95.574
e	1270.655	933.691	3001.201

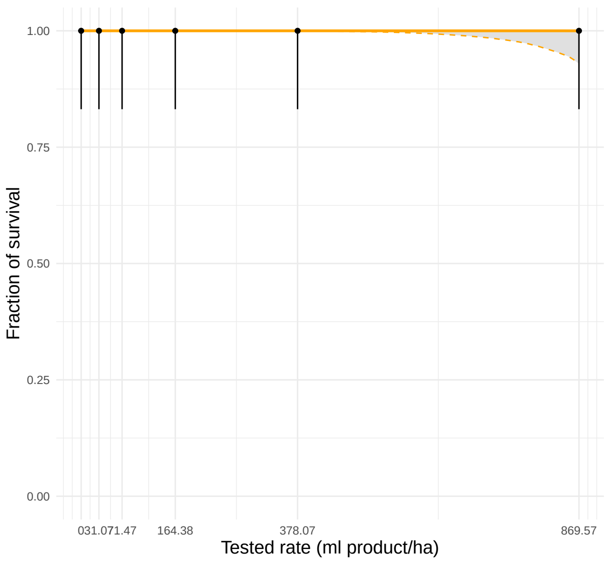

(a) Dose-response curve

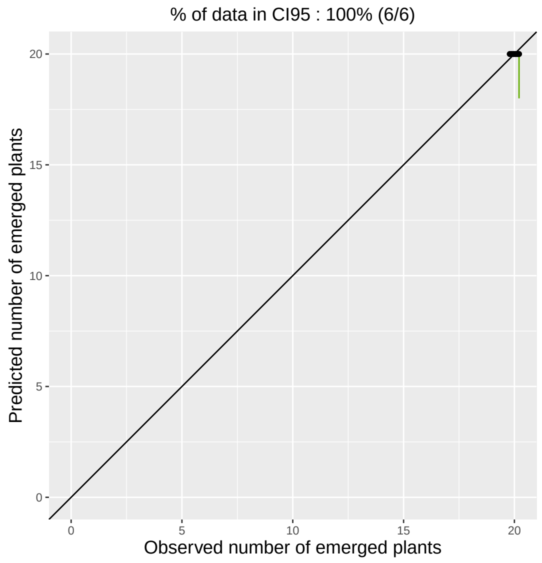

(b) Posterior predictive check (PPC)

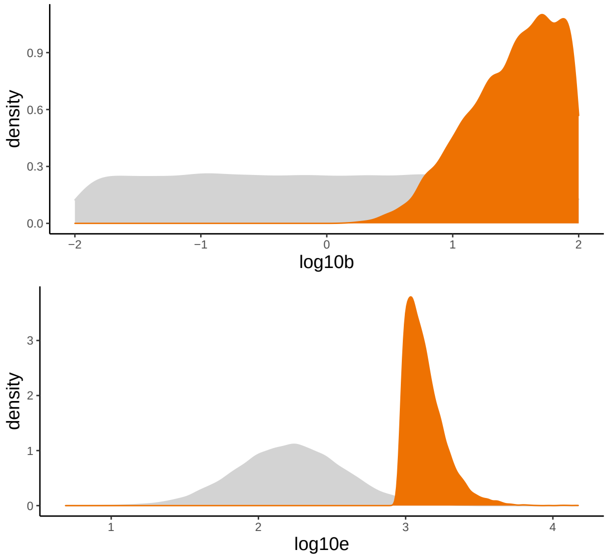

(c) Priors and posteriors

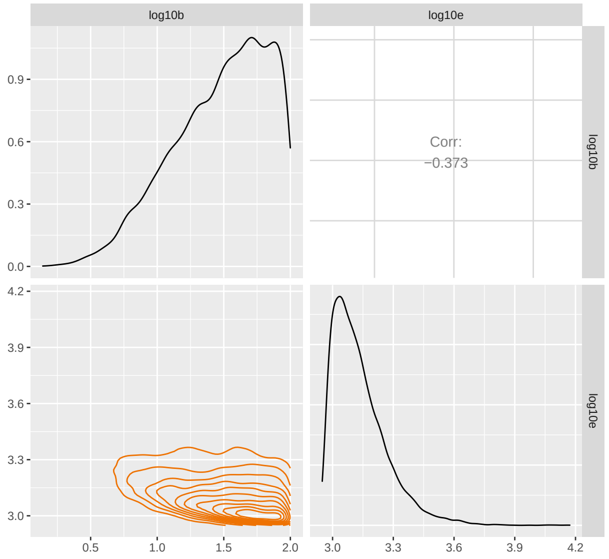

(d) Correlations between parameters

Figure 6: Dose-response curve (a), PPC (b), prior and posterior distributions (c) and correlations between parameters (d).

Data set: HELAN_SE_emergence

Table 7: Summary of parameter estimates (parameter d is set to 1) for HELAN_SE_emergence data set

Parameter	median	Q2.5	Q97.5
b	1.964	1.122	3.446
e	2543.192	1395.435	6113.227

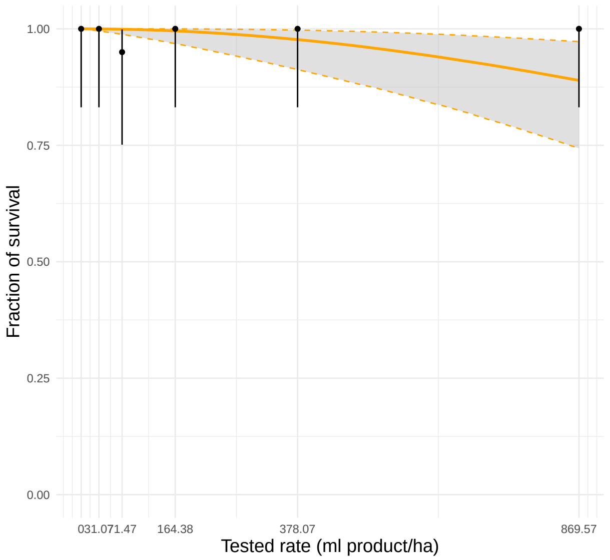

(a) Dose-response curve

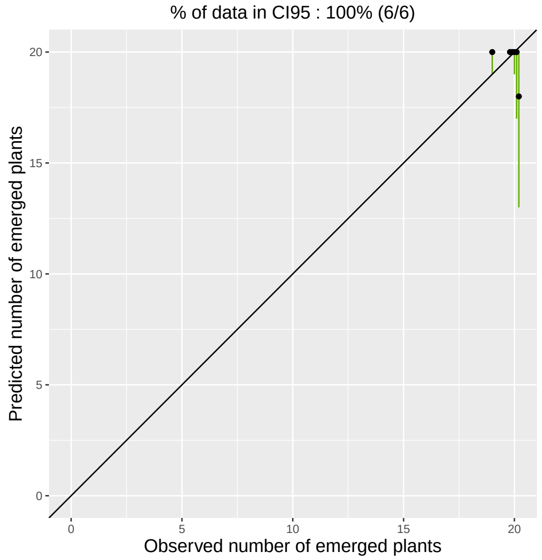

(b) Posterior predictive check (PPC)

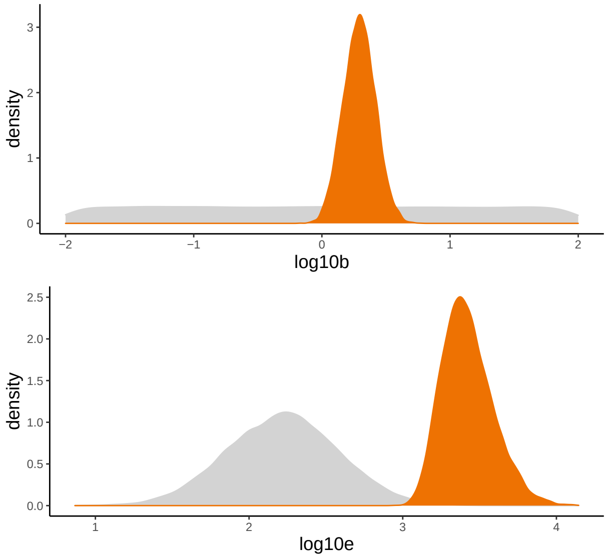

(c) Priors and posteriors

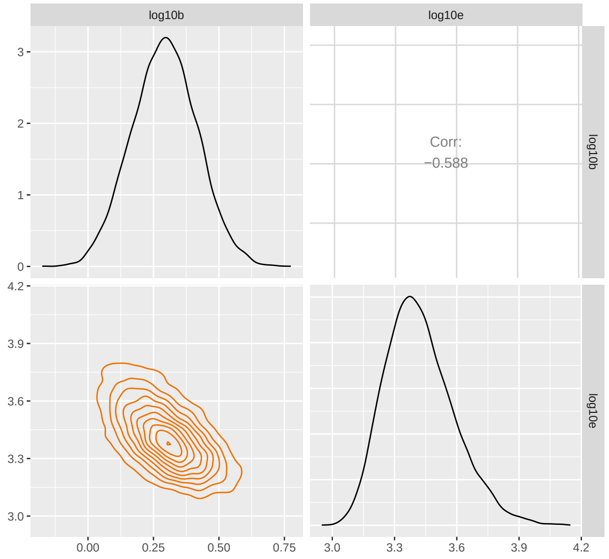

(d) Correlations between parameters

Figure 7: Dose-response curve (a), PPC (b), prior and posterior distributions (c) and correlations between parameters (d).

Data set: LYPES_SE_emergence

Table 8: Summary of parameter estimates for LYPES_SE_emergence data set

Parameter	median	Q2.5	Q97.5
b	29.884	3.041	94.884
d	0.938	0.885	0.973
e	1246.794	924.337	2886.373

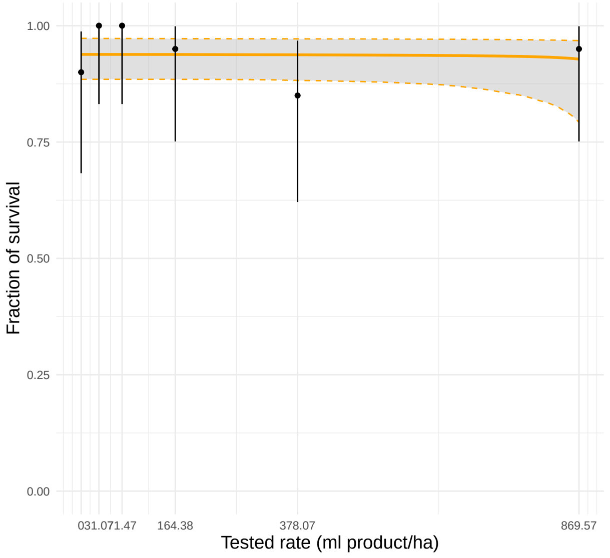

(a) Dose-response curve

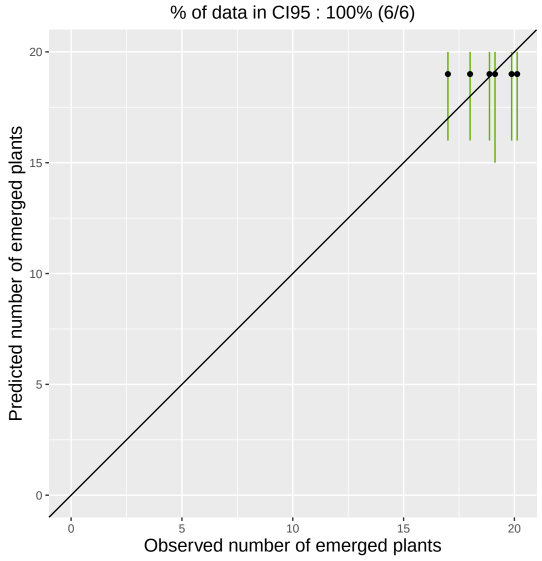

(b) Posterior predictive check (PPC)

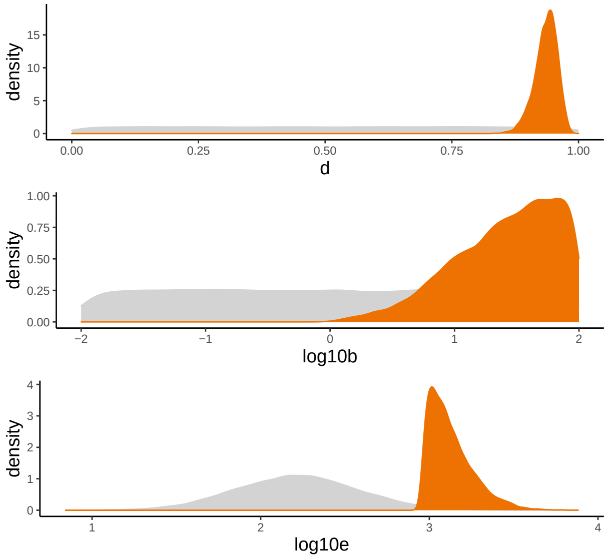

(c) Priors and posteriors

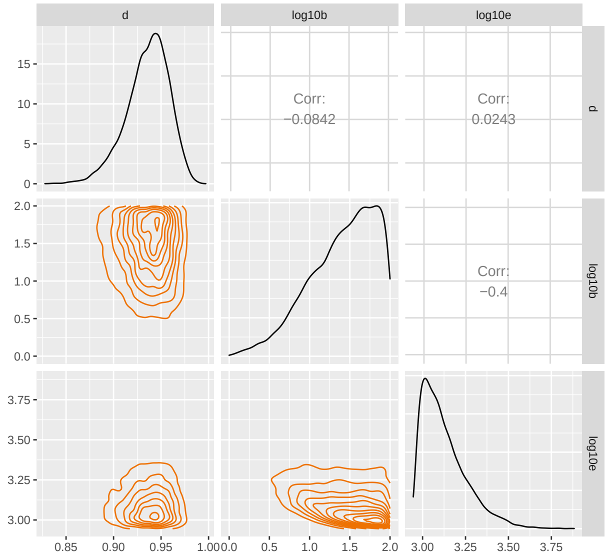

(d) Correlations between parameters

Figure 8: Dose-response curve (a), PPC (b), prior and posterior distributions (c) and correlations between parameters (d).

Data set: TRZAW_SE_emergence

Table 9: Summary of parameter estimates for TRZAW_SE_emergence data set

Parameter	median	Q2.5	Q97.5
b	7.238	2.312	60.868
d	0.983	0.945	0.997
e	2093.930	1879.297	2909.464

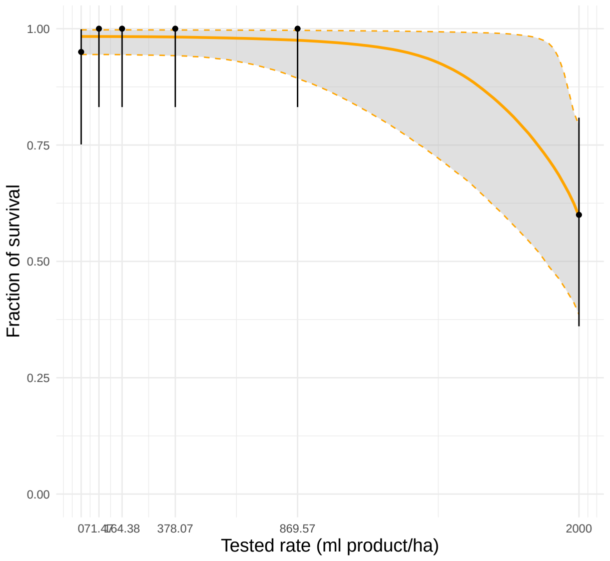

(a) Dose-response curve

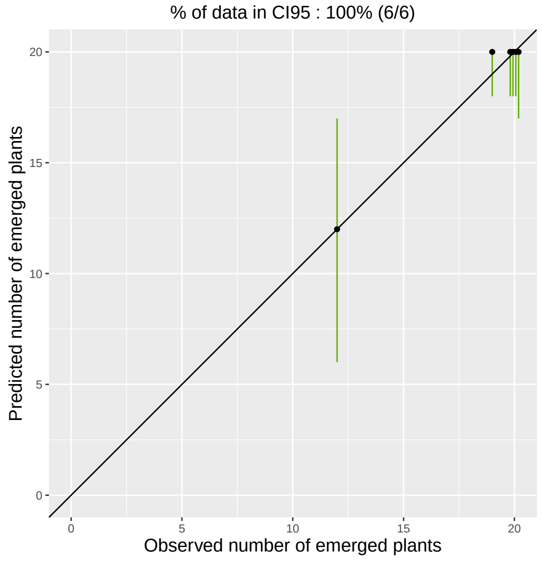

(b) Posterior predictive check (PPC)

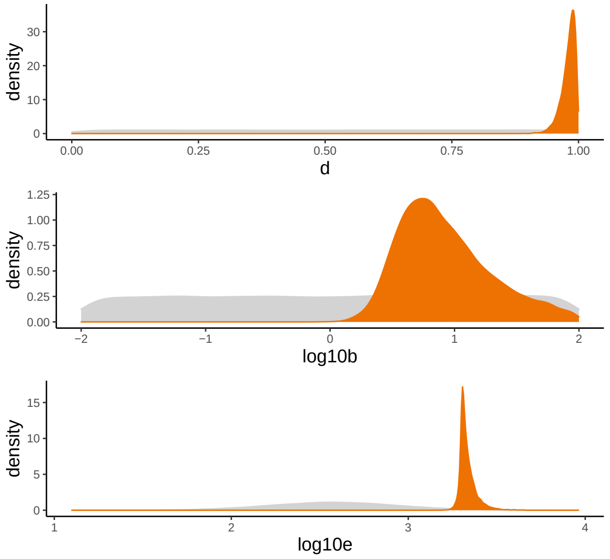

(c) Priors and posteriors

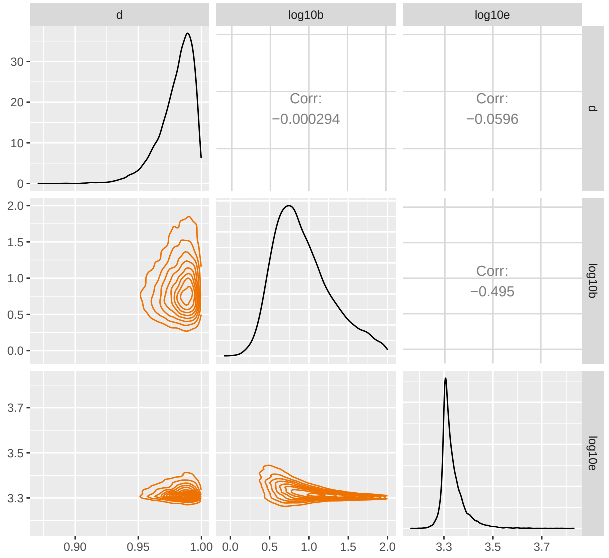

(d) Correlations between parameters

Figure 9: Dose-response curve (a), PPC (b), prior and posterior distributions (c) and correlations between parameters (d).

Data set: ZEAMA_SE_emergence

Table 10: Summary of parameter estimates (parameter d is set to 1) for ZEAMA_SE_emergence data set

Parameter	median	Q2.5	Q97.5
b	1.385	0.859	2.256
e	5813.628	3111.806	14090.879

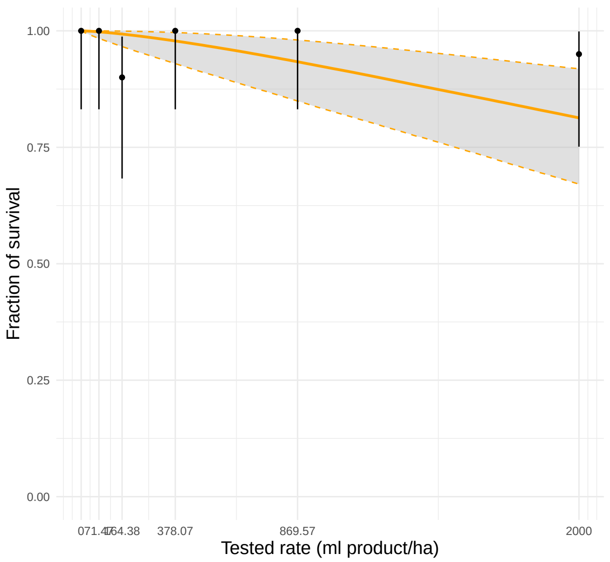

(a) Dose-response curve

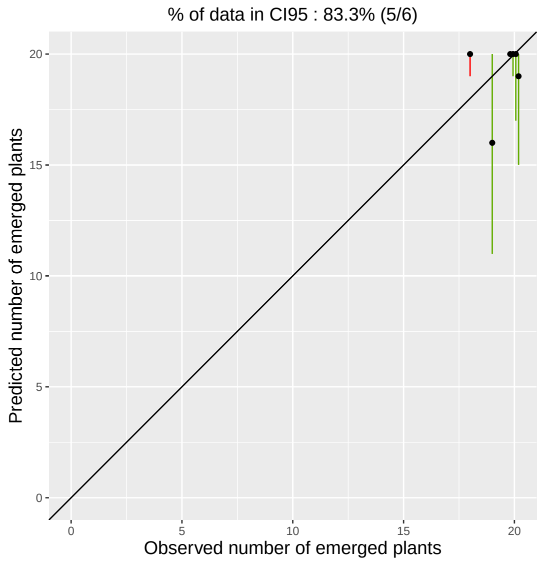

(b) Posterior predictive check (PPC)

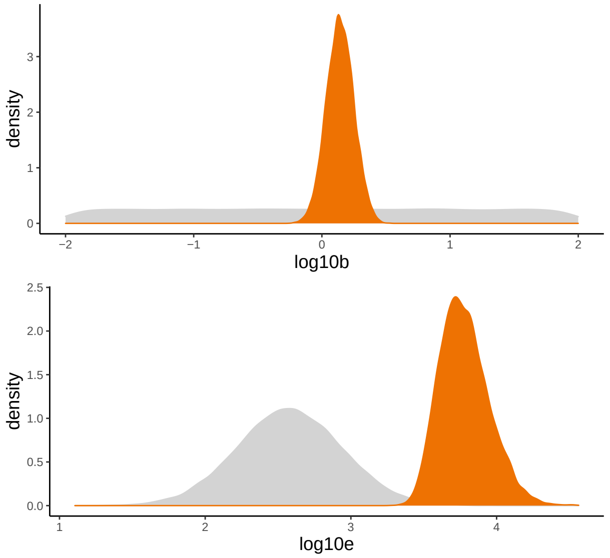

(c) Priors and posteriors

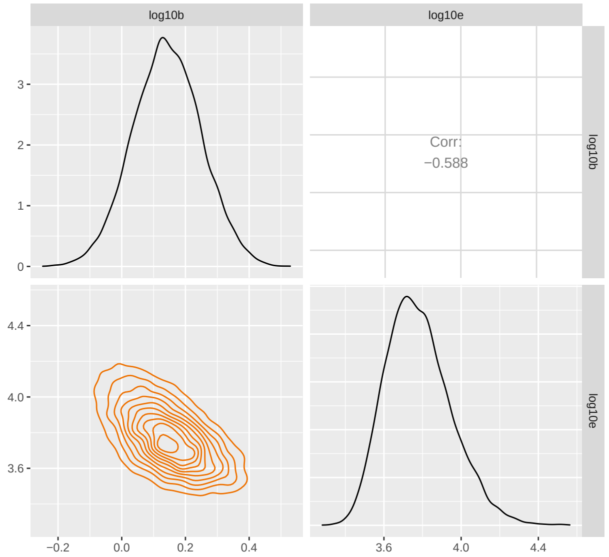

(d) Correlations between parameters

Figure 10: Dose-response curve (a), PPC (b), prior and posterior distributions (c) and correlations between parameters (d).
