# Supplementary material for: How to account for the uncertainty from standard toxicity tests in species sensitivity distributions: An example in non-target plants
Source: PLoS One. 2021 Jan 7;16(1):e0245071. doi: 10.1371/journal.pone.0245071 (PMC7790375; doi:10.1371/journal.pone.0245071)
Supplement: S1 Archive — It is a zip file containing seven folders (one folder per case study). Each folder contains five files report_xxx.pdf with detailed results of the dose-response analyses, one file corresponding to does-response analysis per endpoint. It also contains one file ER50_censoring.pdf for censored ER50 and one file SSD_analyses.pdf for results of SSD analyses. (ZIP) [file pone.0245071.s004.zip › S1_archive/Study2/report_SE_emergence.pdf]

Dose-response analyses

Study 2

Seedling Emergence test - emergence endpoint

25 June 2020

Contact: sandrine.charles@univ-lyon1.fr

This is a report which provides results on all performed dose-response analyses for the emergence endpoint of the Seedling Emergence test for study 2.

Contents

Data set: ALLCE_SE_emergence . . . 2
Data set: AVESA_SE_emergence . . . 3
Data set: BEAVA_SE_emergence . . . 4
Data set: BRSNW_SE_emergence . . . 5
Data set: CUMSA_SE_emergence . . . 6
Data set: GLXMA_SE_emergence . . . 7
Data set: HELAN_SE_emergence . . . 8
Data set: LOLPE_SE_emergence . . . 9
Data set: LYPES_SE_emergence . . . 10
Data set: ZEAMA_SE_emergence . . . 11

Data set: ALLCE_SE_emergence

Table 1: Summary of parameter estimates for ALLCE_SE_emergence data set

Parameter	median	Q2.5	Q97.5
b	18.855	2.070	92.244
d	0.883	0.835	0.924
e	41.298	30.148	105.057

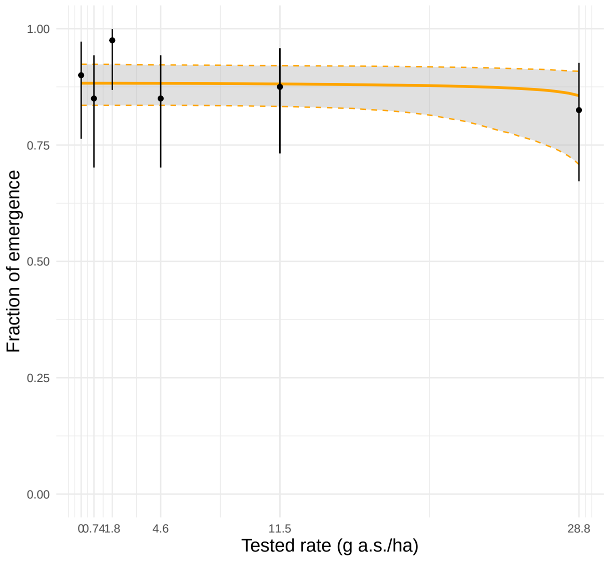

(a) Dose-response curve

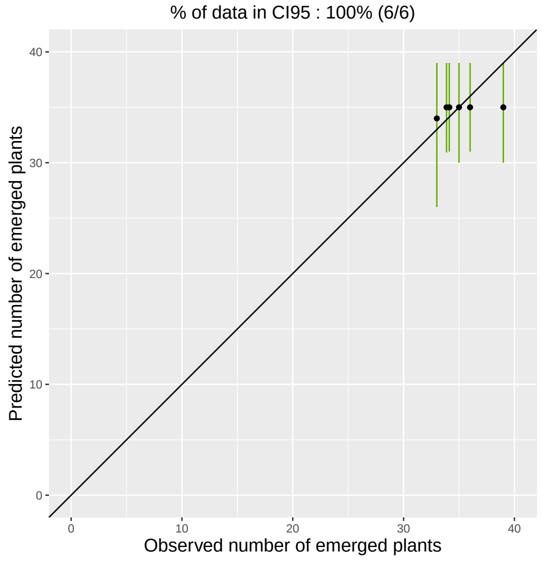

(b) Posterior predictive check (PPC)

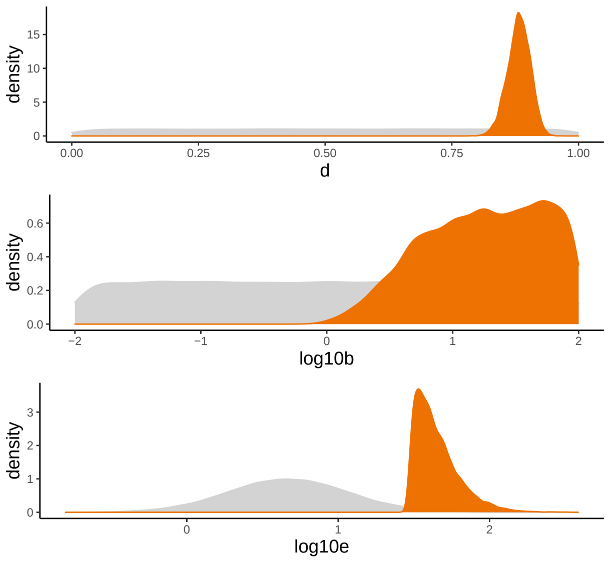

(c) Priors and posteriors

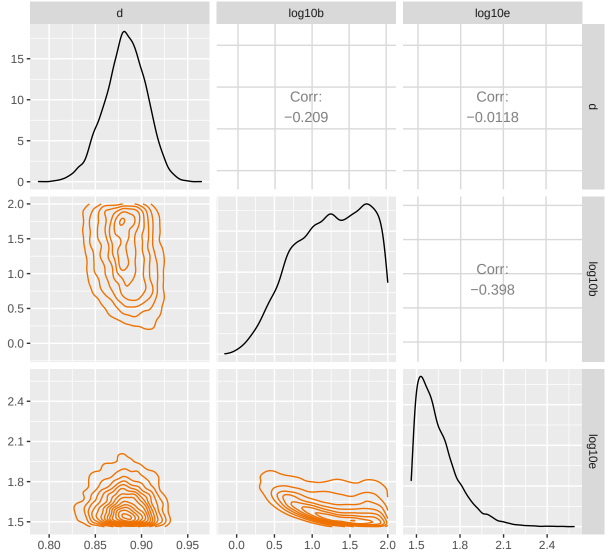

(d) Correlations between parameters

Figure 1: Dose-response curve (a), PPC (b), prior and posterior distributions (c) and correlations between parameters (d).

Data set: AVESA_SE_emergence

Table 2: Summary of parameter estimates for AVESA_SE_emergence data set

Parameter	median	Q2.5	Q97.5
b	32.577	4.714	94.702
d	0.969	0.942	0.986
e	268.672	193.990	661.026

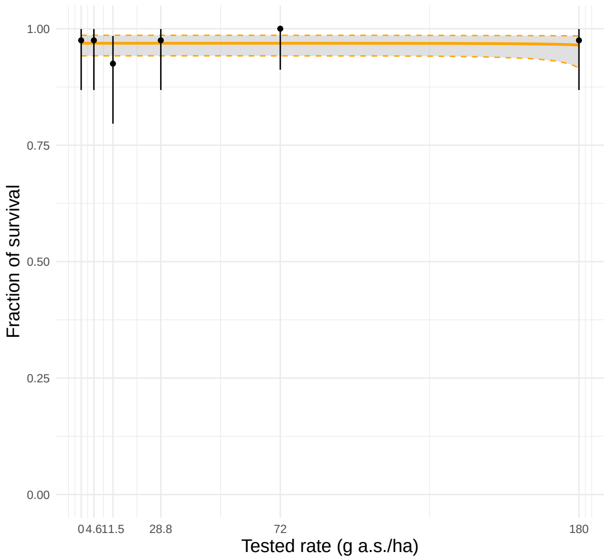

(a) Dose-response curve

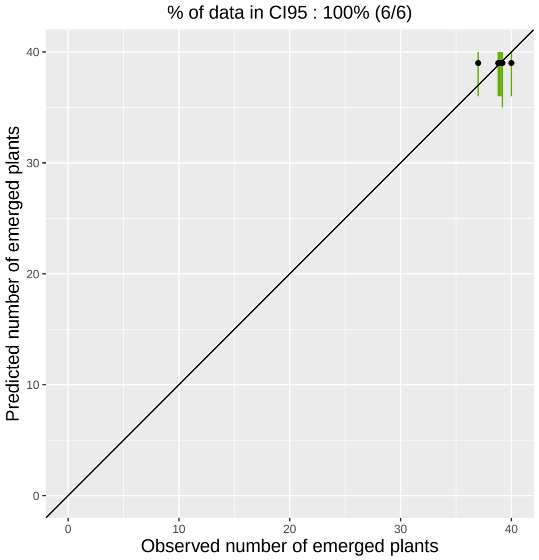

(b) Posterior predictive check (PPC)

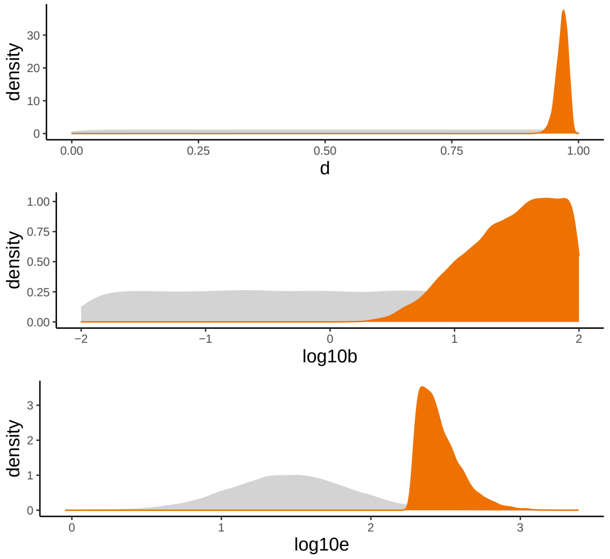

(c) Priors and posteriors

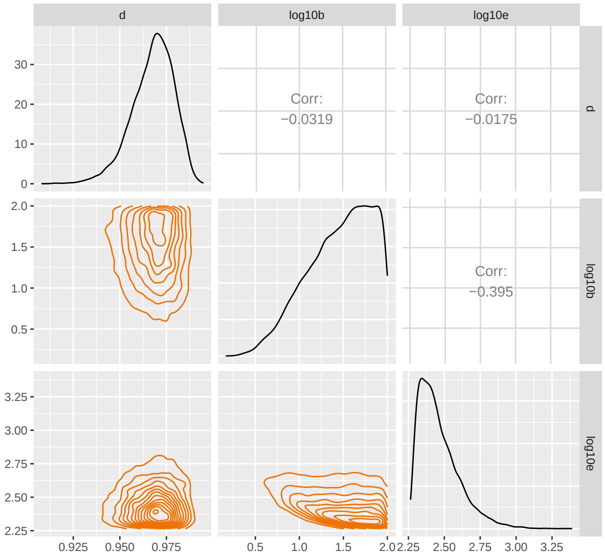

(d) Correlations between parameters

Figure 2: Dose-response curve (a), PPC (b), prior and posterior distributions (c) and correlations between parameters (d).

Data set: BEAVA_SE_emergence

Table 3: Summary of parameter estimates for BEAVA_SE_emergence data set

Parameter	median	Q2.5	Q97.5
b	29.112	3.458	94.438
d	0.861	0.816	0.902
e	42.933	30.786	107.747

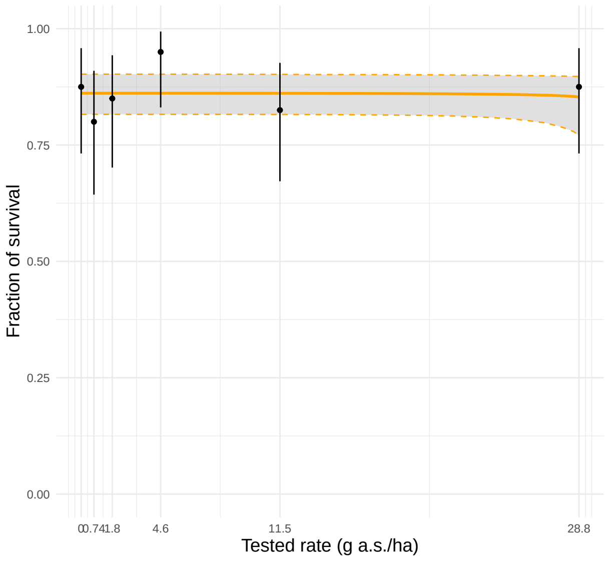

(a) Dose-response curve

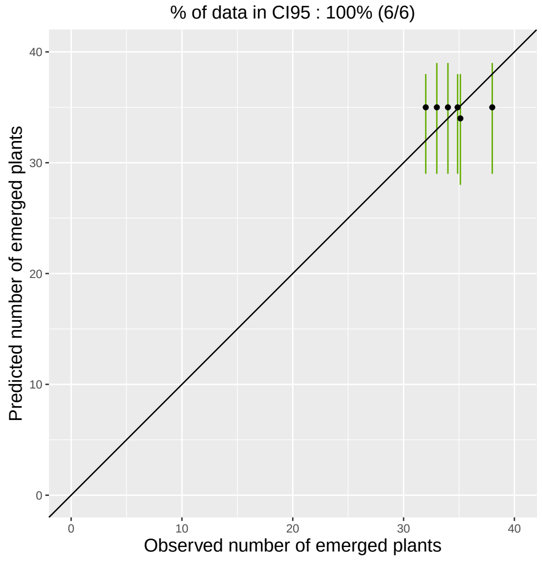

(b) Posterior predictive check (PPC)

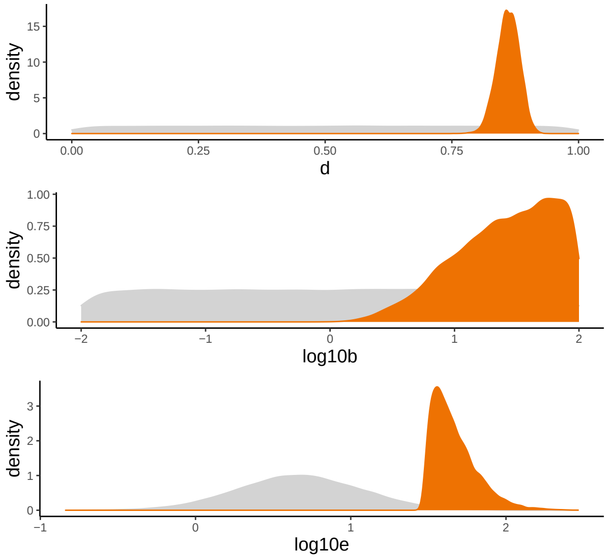

(c) Priors and posteriors

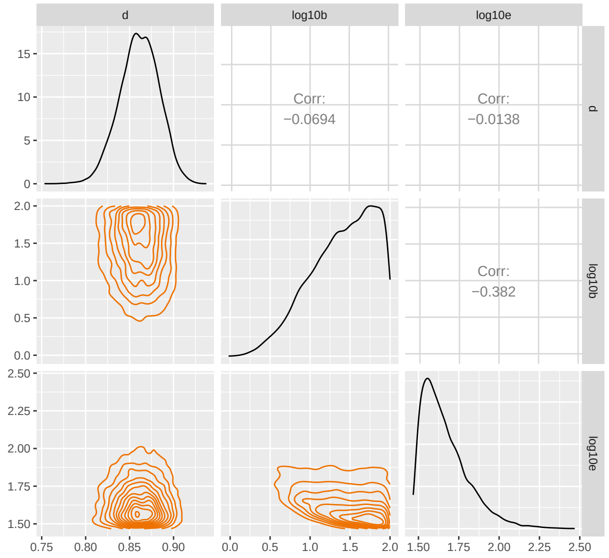

(d) Correlations between parameters

Figure 3: Dose-response curve (a), PPC (b), prior and posterior distributions (c) and correlations between parameters (d).

Data set: BRSNW_SE_emergence

Table 4: Summary of parameter estimates for BRSNW_SE_emergence data set

Parameter	median	Q2.5	Q97.5
b	29.073	3.879	94.291
d	0.928	0.891	0.956
e	268.648	192.273	673.321

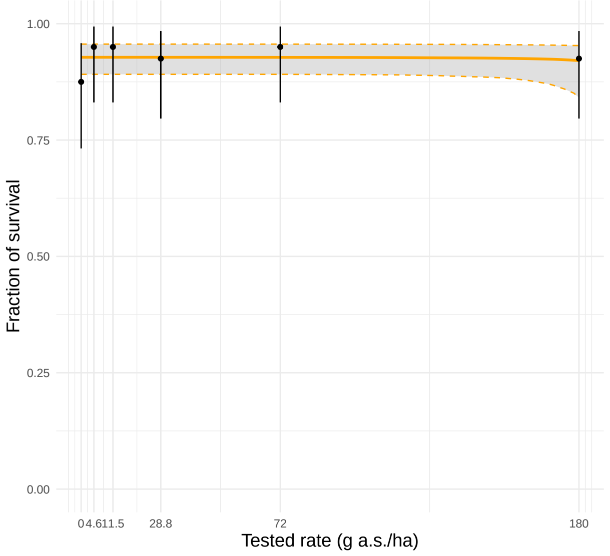

(a) Dose-response curve

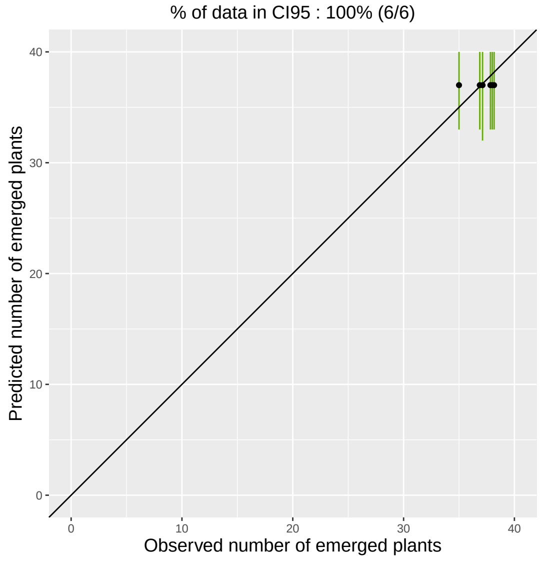

(b) Posterior predictive check (PPC)

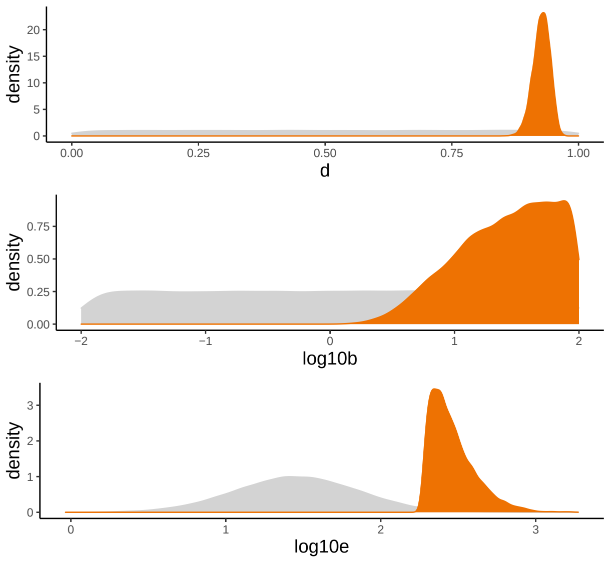

(c) Priors and posteriors

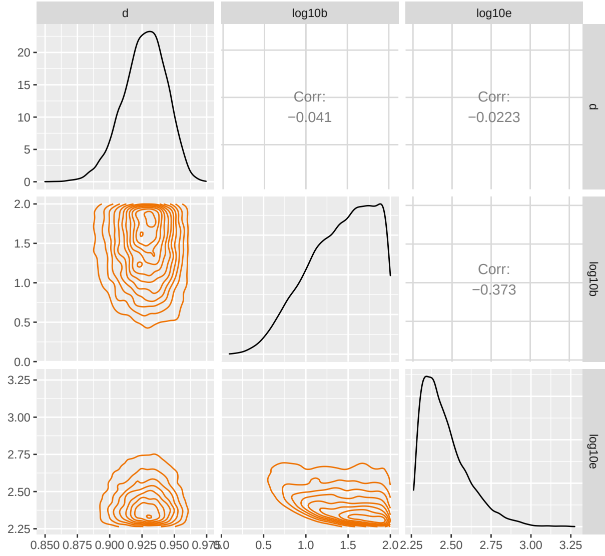

(d) Correlations between parameters

Figure 4: Dose-response curve (a), PPC (b), prior and posterior distributions (c) and correlations between parameters (d).

Data set: CUMSA_SE_emergence

Table 5: Summary of parameter estimates for CUMSA_SE_emergence data set

Parameter	median	Q2.5	Q97.5
b	26.365	3.297	93.365
d	0.920	0.882	0.951
e	262.856	190.861	656.414

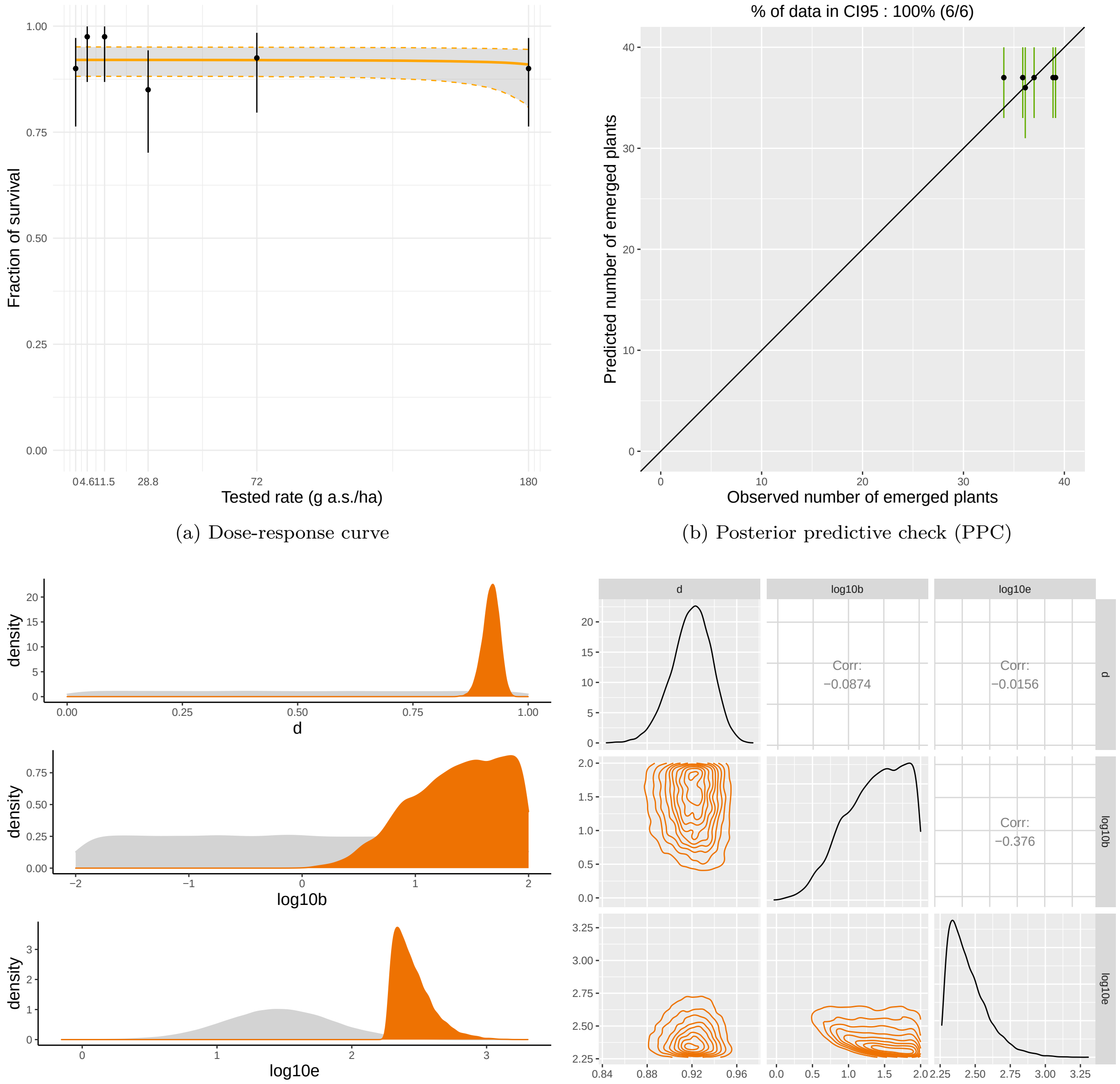

(a) Dose-response curve

(b) Posterior predictive check (PPC)

(c) Priors and posteriors

(d) Correlations between parameters

Figure 5: Dose-response curve (a), PPC (b), prior and posterior distributions (c) and correlations between parameters (d).

Data set: GLXMA_SE_emergence

Table 6: Summary of parameter estimates for GLXMA_SE_emergence data set

Parameter	median	Q2.5	Q97.5
b	21.972	2.247	92.993
d	0.918	0.878	0.951
e	262.148	189.786	661.344

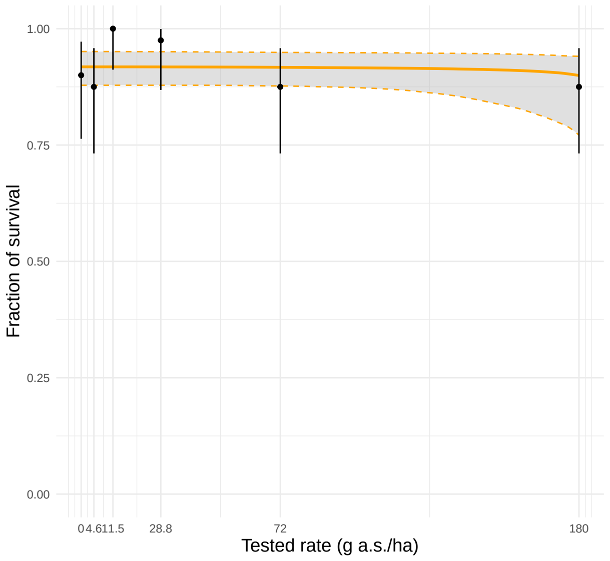

(a) Dose-response curve

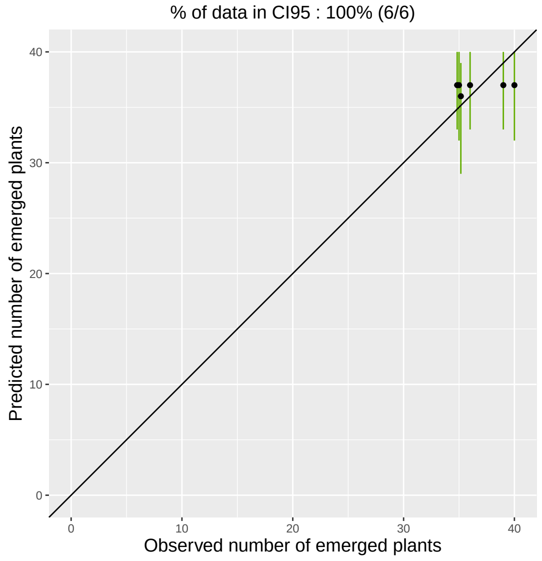

(b) Posterior predictive check (PPC)

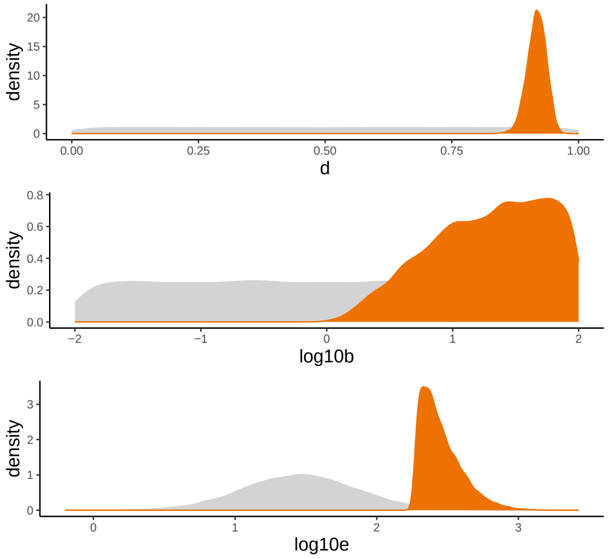

(c) Priors and posteriors

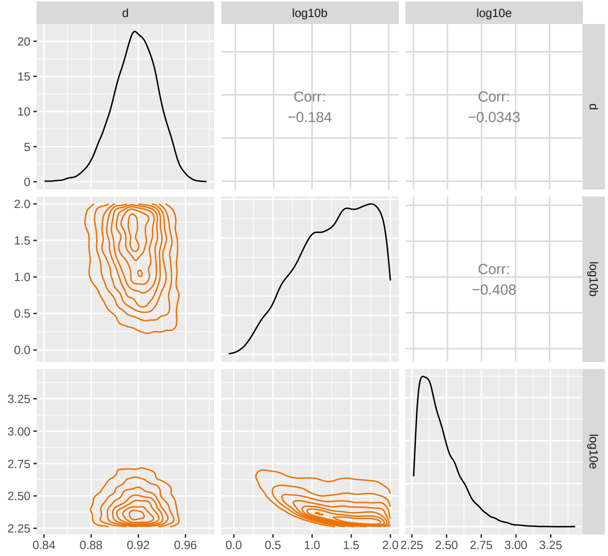

(d) Correlations between parameters

Figure 6: Dose-response curve (a), PPC (b), prior and posterior distributions (c) and correlations between parameters (d).

Data set: HELAN_SE_emergence

Table 7: Summary of parameter estimates for HELAN_SE_emergence data set

Parameter	median	Q2.5	Q97.5
b	35.784	5.436	94.713
d	0.960	0.931	0.980
e	274.842	195.480	668.667

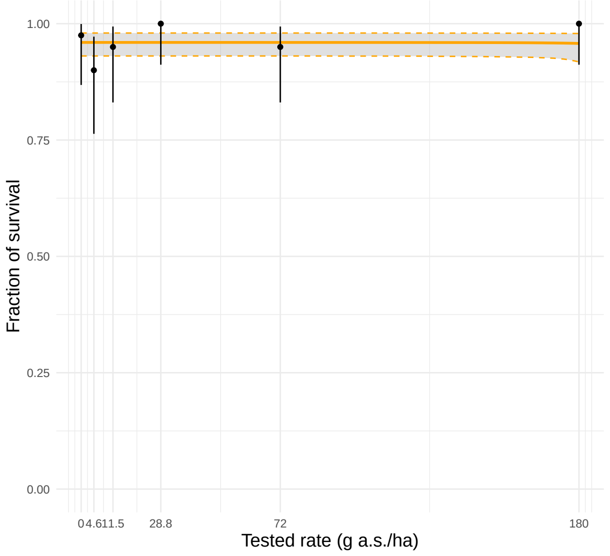

(a) Dose-response curve

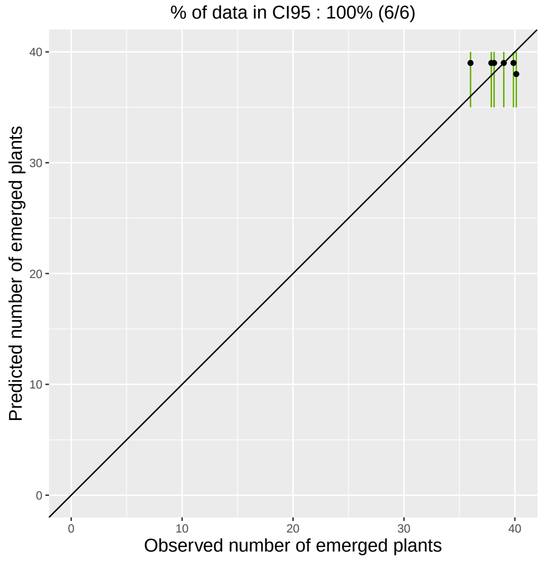

(b) Posterior predictive check (PPC)

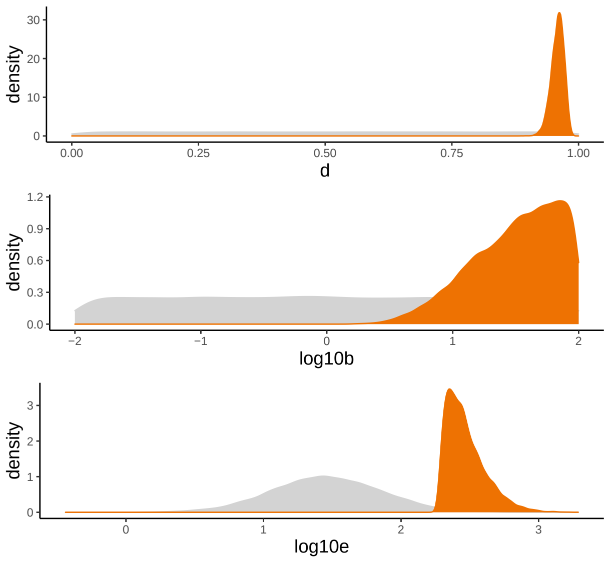

(c) Priors and posteriors

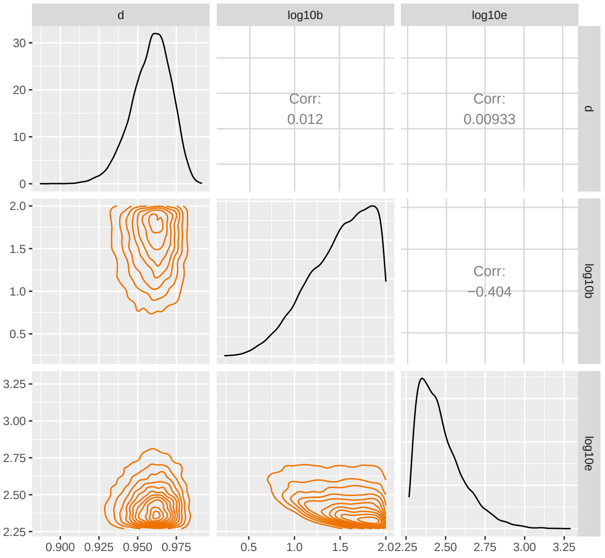

(d) Correlations between parameters

Figure 7: Dose-response curve (a), PPC (b), prior and posterior distributions (c) and correlations between parameters (d).

Data set: LOLPE_SE_emergence

Table 8: Summary of parameter estimates for LOLPE_SE_emergence data set

Parameter	median	Q2.5	Q97.5
b	31.536	4.408	94.940
d	0.890	0.847	0.925
e	107.631	77.598	267.661

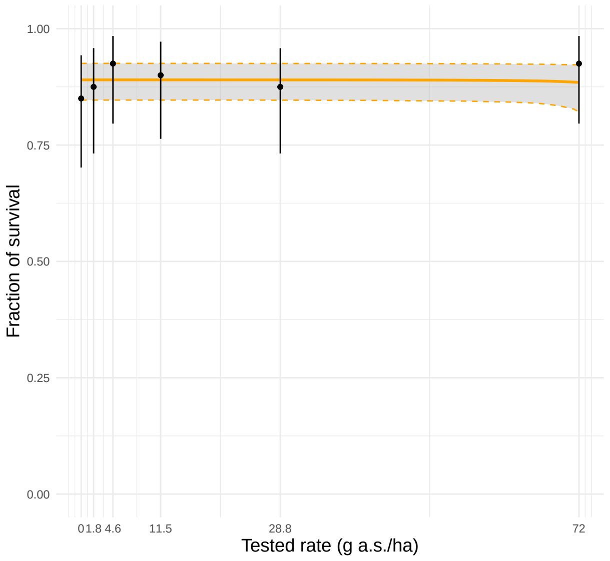

(a) Dose-response curve

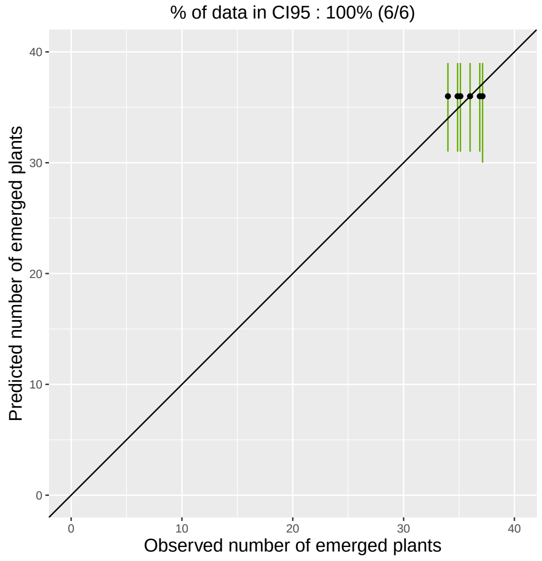

(b) Posterior predictive check (PPC)

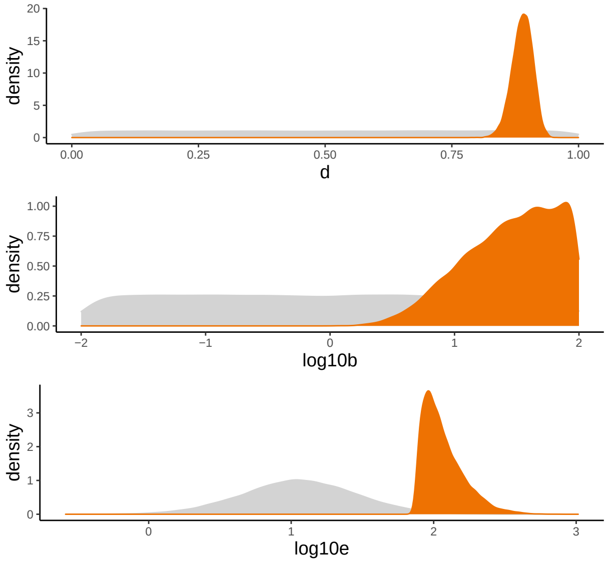

(c) Priors and posteriors

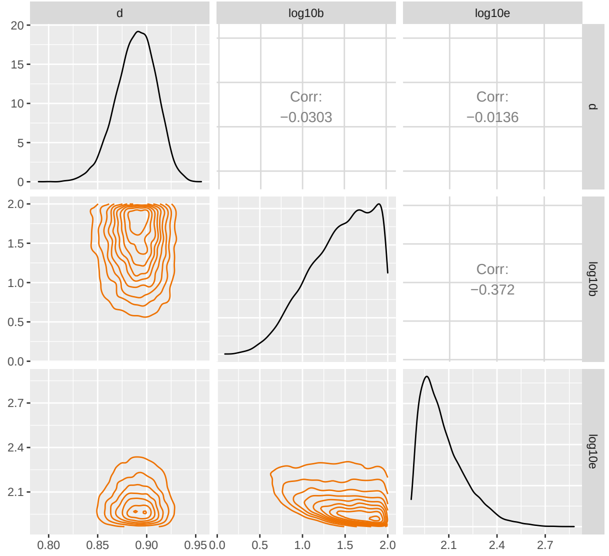

(d) Correlations between parameters

Figure 8: Dose-response curve (a), PPC (b), prior and posterior distributions (c) and correlations between parameters (d).

Data set: LYPES_SE_emergence

Table 9: Summary of parameter estimates (parameter d is set to 1) for LYPES_SE_emergence data set

Parameter	median	Q2.5	Q97.5
b	2.326	1.318	4.176
e	543.278	311.511	1338.246

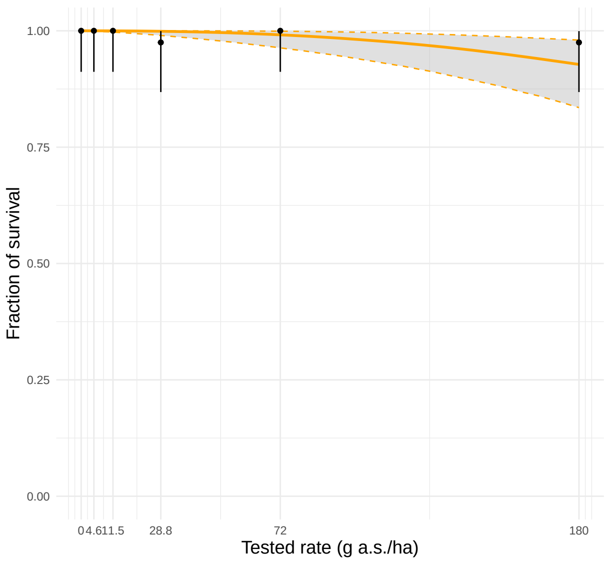

(a) Dose-response curve

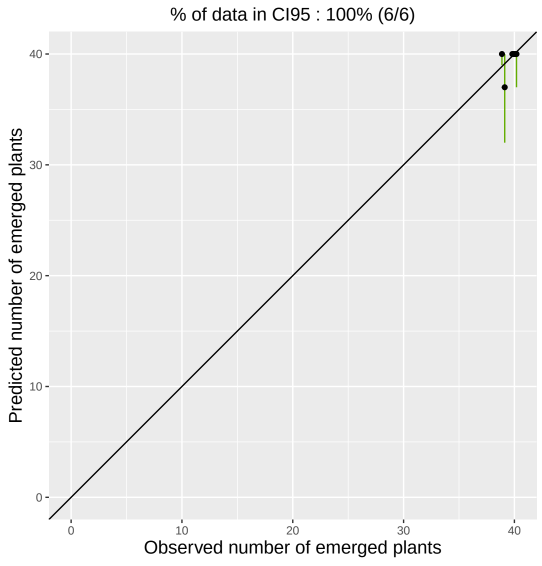

(b) Posterior predictive check (PPC)

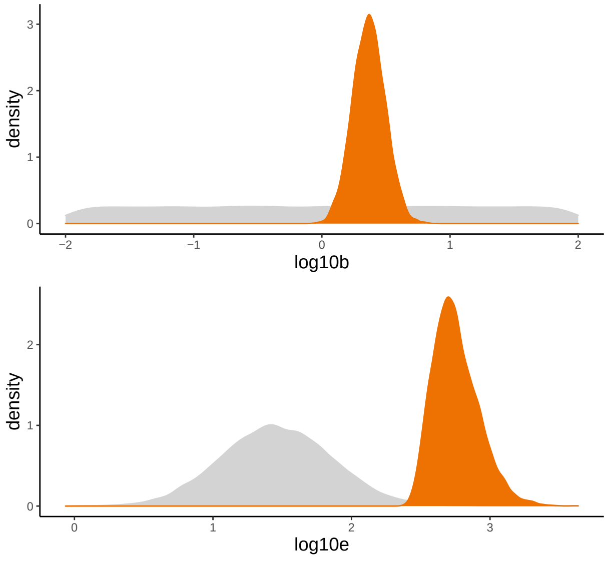

(c) Priors and posteriors

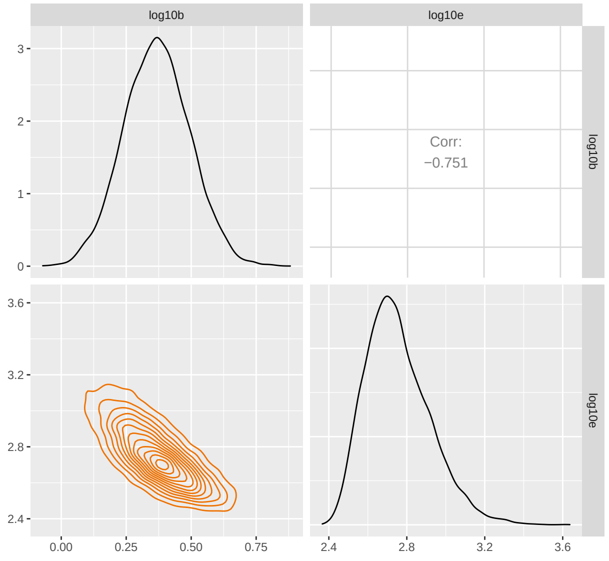

(d) Correlations between parameters

Figure 9: Dose-response curve (a), PPC (b), prior and posterior distributions (c) and correlations between parameters (d).

Data set: ZEAMA_SE_emergence

Table 10: Summary of parameter estimates (parameter d is set to 1) for ZEAMA_SE_emergence data set

Parameter	median	Q2.5	Q97.5
b	1.039	0.721	1.498
e	1096.913	516.603	2938.759

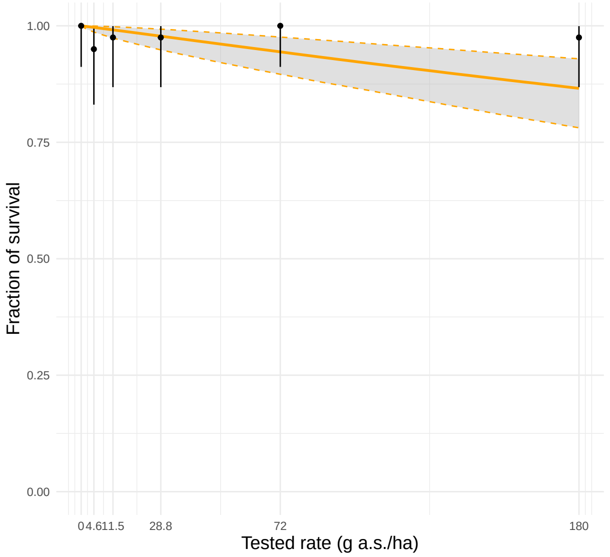

(a) Dose-response curve

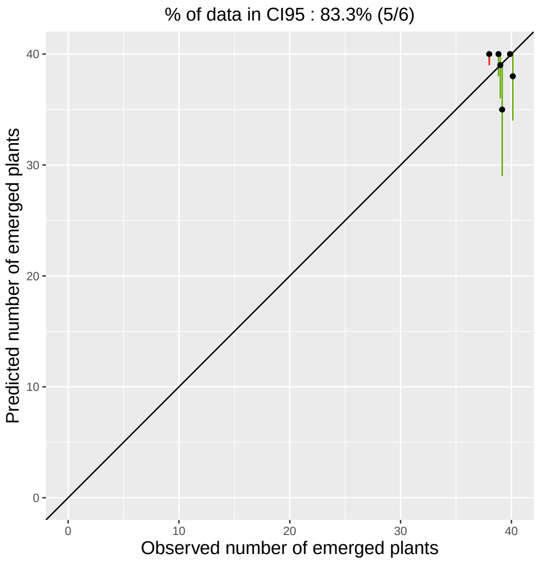

(b) Posterior predictive check (PPC)

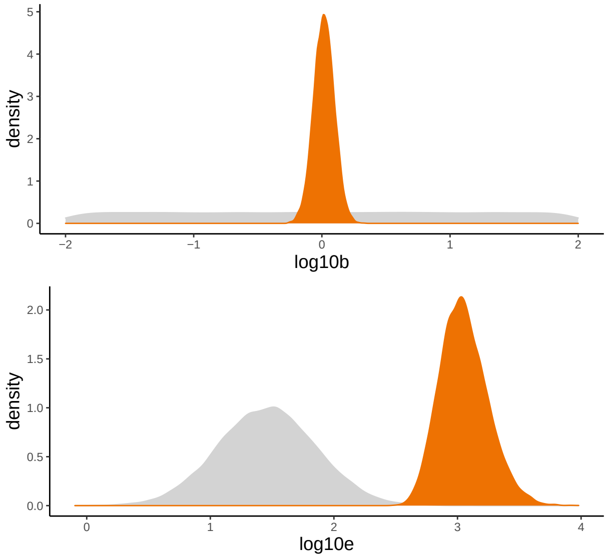

(c) Priors and posteriors

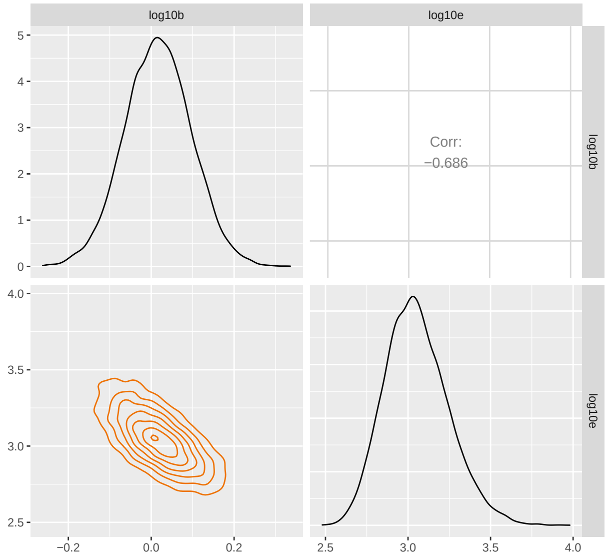

(d) Correlations between parameters

Figure 10: Dose-response curve (a), PPC (b), prior and posterior distributions (c) and correlations between parameters (d).
