# Supplementary material for: How to account for the uncertainty from standard toxicity tests in species sensitivity distributions: An example in non-target plants
Source: PLoS One. 2021 Jan 7;16(1):e0245071. doi: 10.1371/journal.pone.0245071 (PMC7790375; doi:10.1371/journal.pone.0245071)
Supplement: S1 Archive — It is a zip file containing seven folders (one folder per case study). Each folder contains five files report_xxx.pdf with detailed results of the dose-response analyses, one file corresponding to does-response analysis per endpoint. It also contains one file ER50_censoring.pdf for censored ER50 and one file SSD_analyses.pdf for results of SSD analyses. (ZIP) [file pone.0245071.s004.zip › S1_archive/Study2/report_SE_survival.pdf]

Dose-response analyses

Study 2

Seedling Emergence test - survival endpoint

25 June 2020

Contact: sandrine.charles@univ-lyon1.fr

This is a report which provides results on all performed dose-response analyses for the survival endpoint of the Seedling Emergence test for study 2.

Contents

Data set: ALLCE_SE_survival 2
Data set: AVESA_SE_survival 3
Data set: BEAVA_SE_survival 4
Data set: BRSNW_SE_survival 5
Data set: CUMSA_SE_survival 6
Data set: GLXMA_SE_survival 7
Data set: HELAN_SE_survival 8
Data set: LOLPE_SE_survival 9
Data set: LYPES_SE_survival 10
Data set: ZEAMA_SE_survival 11

Data set: ALLCE_SE_survival

Table 1: Summary of parameter estimates for ALLCE_SE_survival data set

Parameter	median	Q2.5	Q97.5
b	8.118	3.409	61.721
d	0.913	0.859	0.953
e	10.909	9.148	11.764

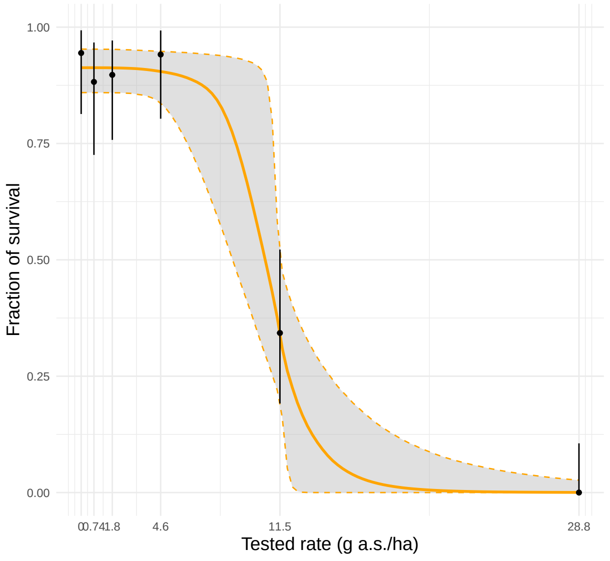

(a) Dose-response curve

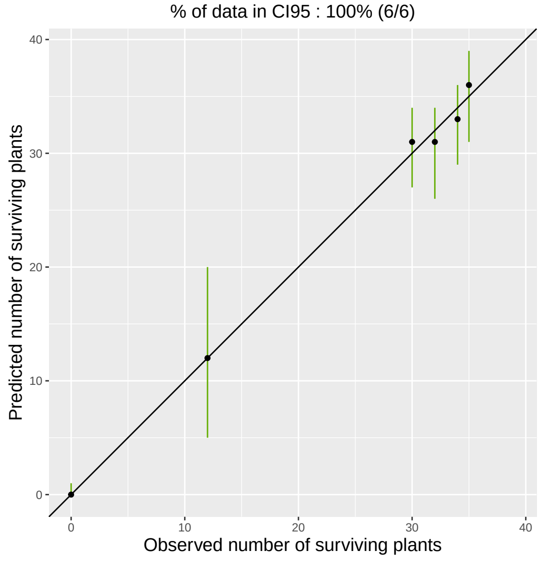

(b) Posterior predictive check (PPC)

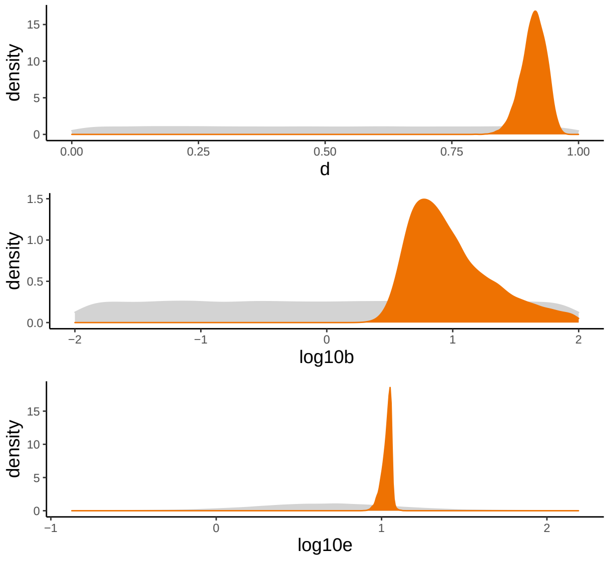

(c) Priors and posteriors

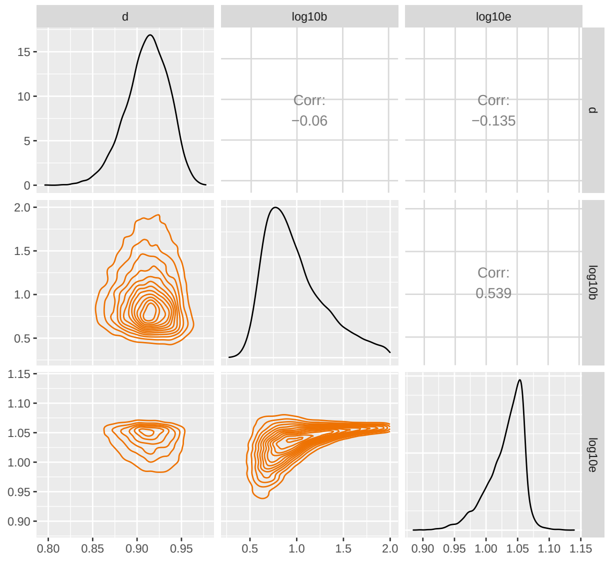

(d) Correlations between parameters

Figure 1: Dose-response curve (a), PPC (b), prior and posterior distributions (c) and correlations between parameters (d).

Data set: AVESA_SE_survival

Table 2: Summary of parameter estimates (parameter d is set to 1) for AVESA_SE_survival data set

Parameter	median	Q2.5	Q97.5
b	1.980	1.192	3.373
e	634.104	347.301	1630.038

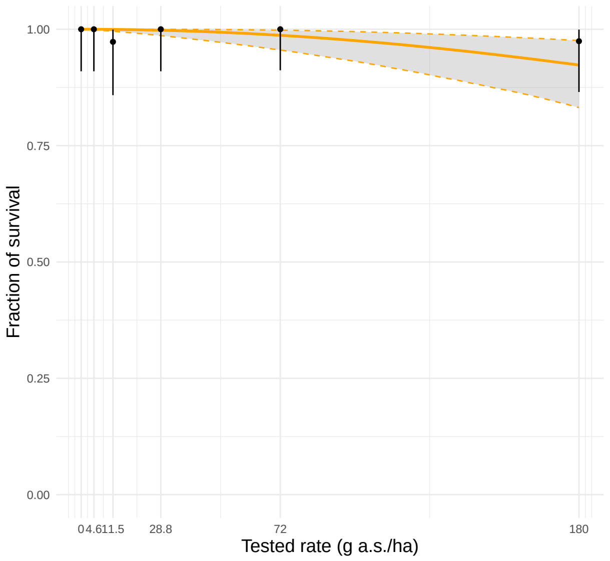

(a) Dose-response curve

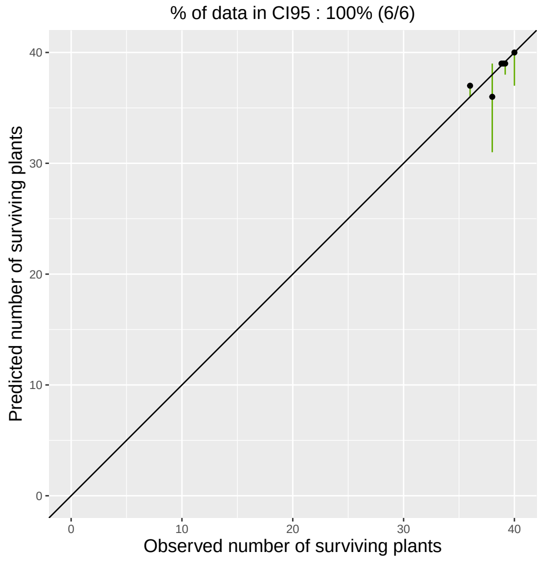

(b) Posterior predictive check (PPC)

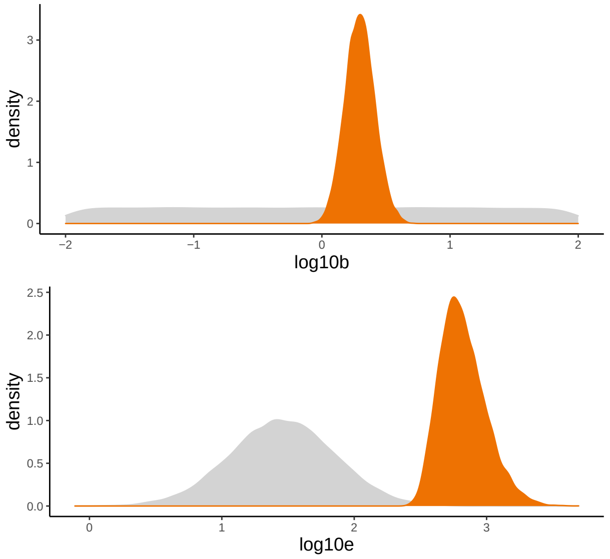

(c) Priors and posteriors

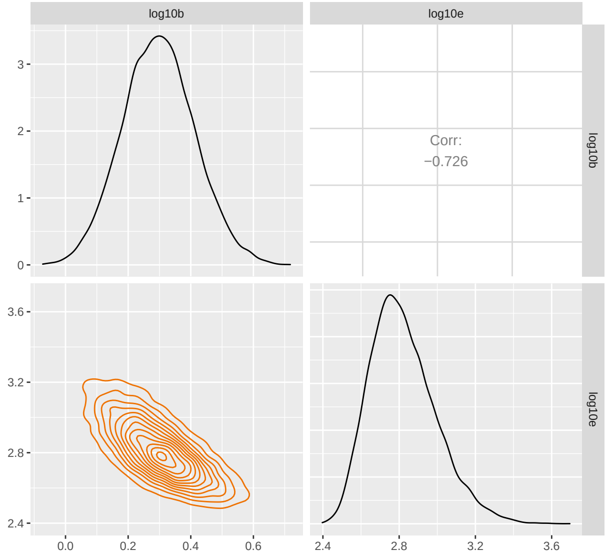

(d) Correlations between parameters

Figure 2: Dose-response curve (a), PPC (b), prior and posterior distributions (c) and correlations between parameters (d).

Data set: BEAVA_SE_survival

Table 3: Summary of parameter estimates (parameter d is set to 1) for BEAVA_SE_survival data set

Parameter	median	Q2.5	Q97.5
b	12.706	2.921	80.197
e	38.173	30.097	88.307

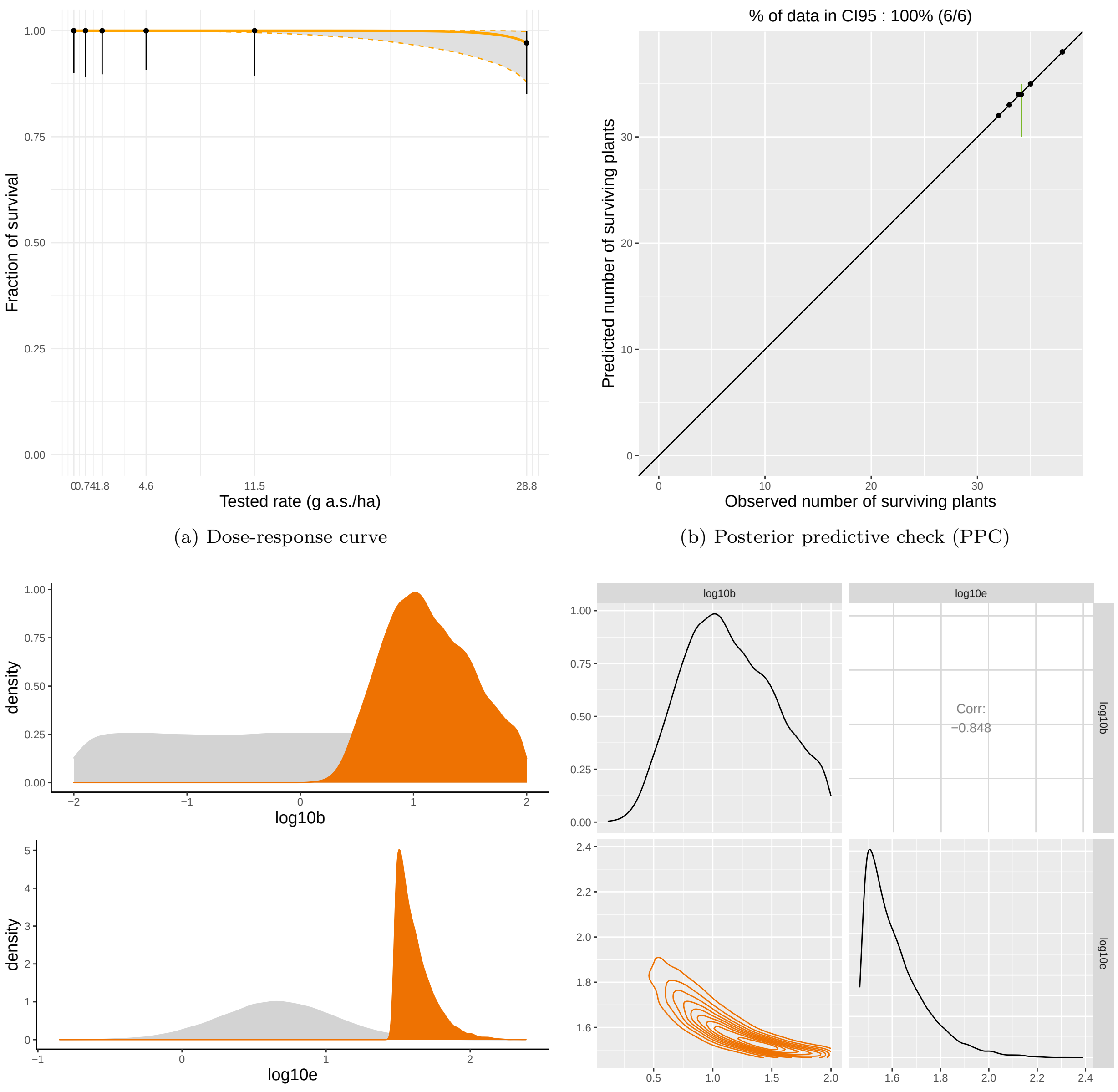

(a) Dose-response curve

(b) Posterior predictive check (PPC)

(c) Priors and posteriors

(d) Correlations between parameters

Figure 3: Dose-response curve (a), PPC (b), prior and posterior distributions (c) and correlations between parameters (d).

Data set: BRSNW_SE_survival

Table 4: Summary of parameter estimates (parameter d is set to 1) for BRSNW_SE_survival data set

Parameter	median	Q2.5	Q97.5
b	1.979	1.204	3.204
e	262.479	189.098	463.606

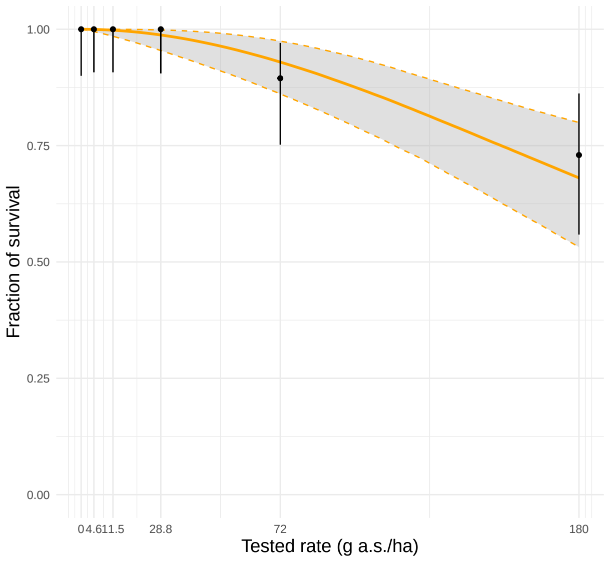

(a) Dose-response curve

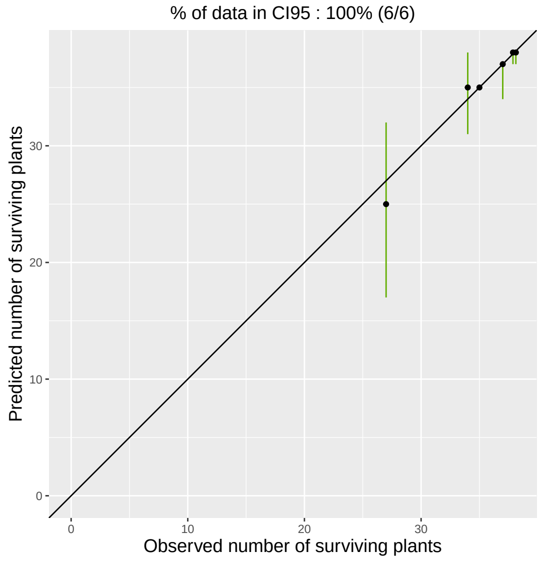

(b) Posterior predictive check (PPC)

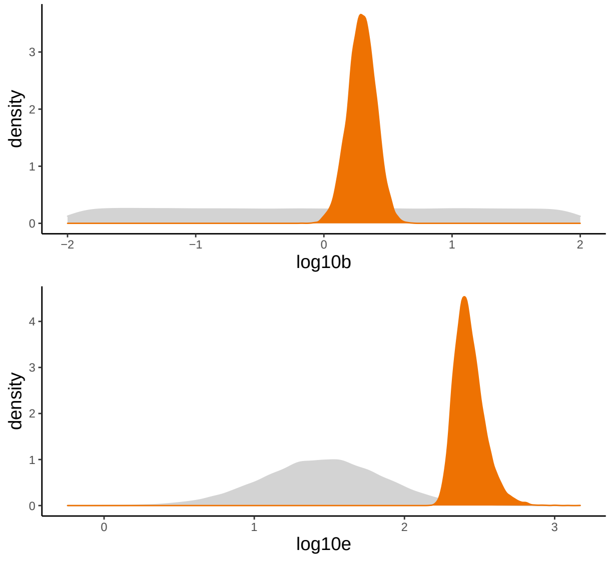

(c) Priors and posteriors

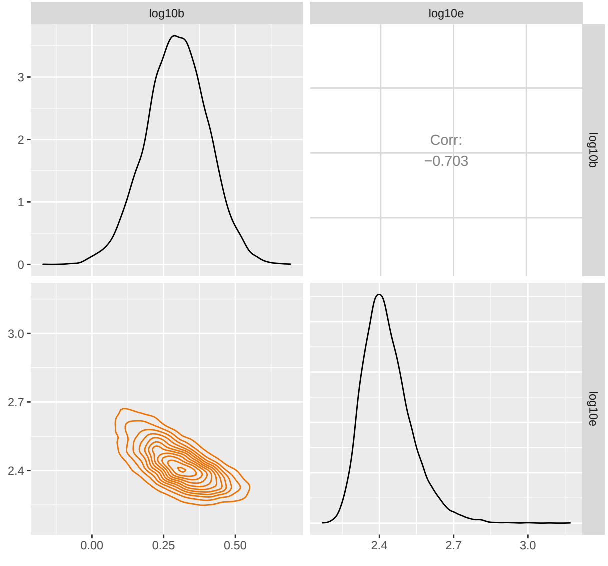

(d) Correlations between parameters

Figure 4: Dose-response curve (a), PPC (b), prior and posterior distributions (c) and correlations between parameters (d).

Data set: CUMSA_SE_survival

Table 5: Summary of parameter estimates (parameter d is set to 1) for CUMSA_SE_survival data set

Parameter	median	Q2.5	Q97.5
b	1.384	0.903	2.021
e	232.752	158.330	424.670

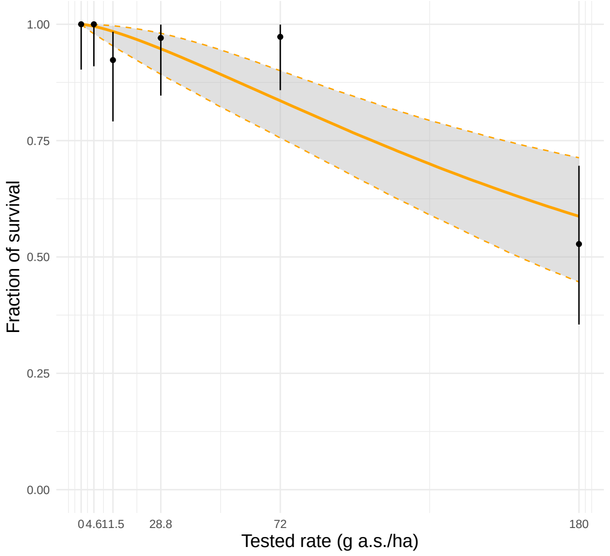

(a) Dose-response curve

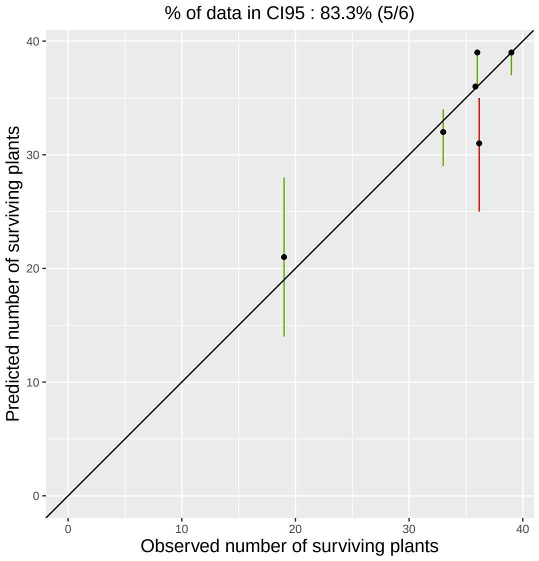

(b) Posterior predictive check (PPC)

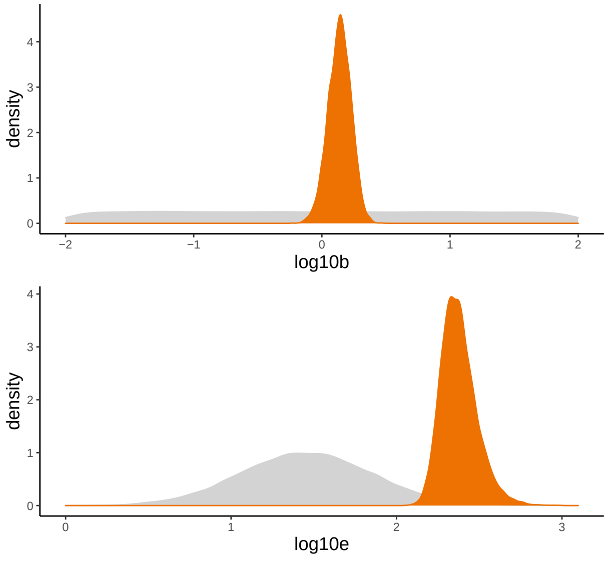

(c) Priors and posteriors

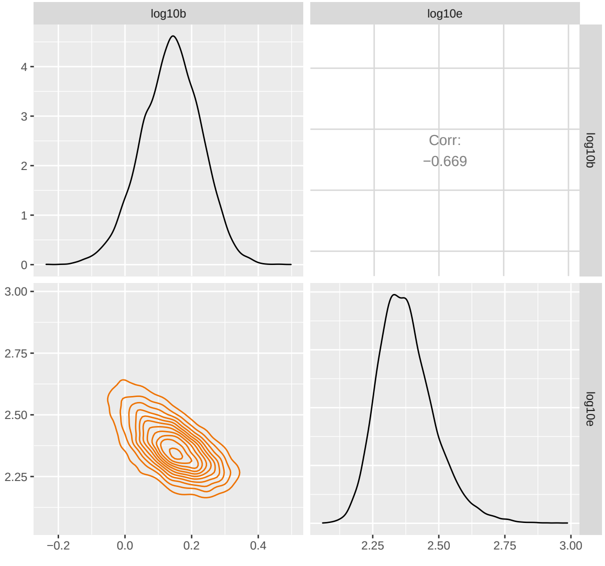

(d) Correlations between parameters

Figure 5: Dose-response curve (a), PPC (b), prior and posterior distributions (c) and correlations between parameters (d).

Data set: GLXMA_SE_survival

Table 6: Summary of parameter estimates (parameter d is set to 1) for GLXMA_SE_survival data set

Parameter	median	Q2.5	Q97.5
b	35.518	5.564	95.150
e	272.223	194.915	689.205

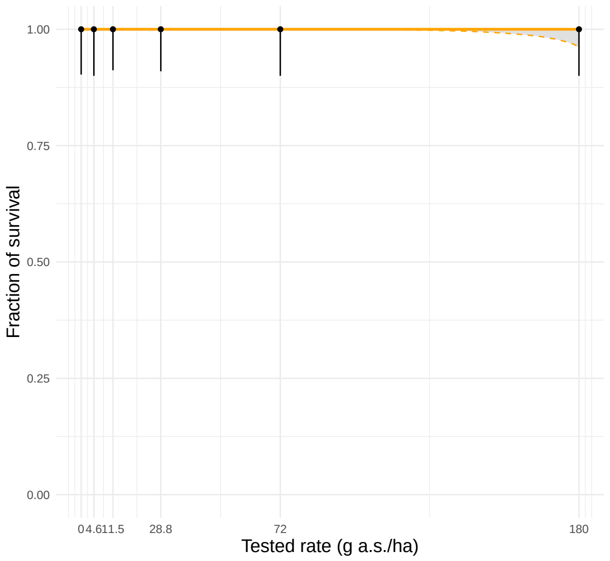

(a) Dose-response curve

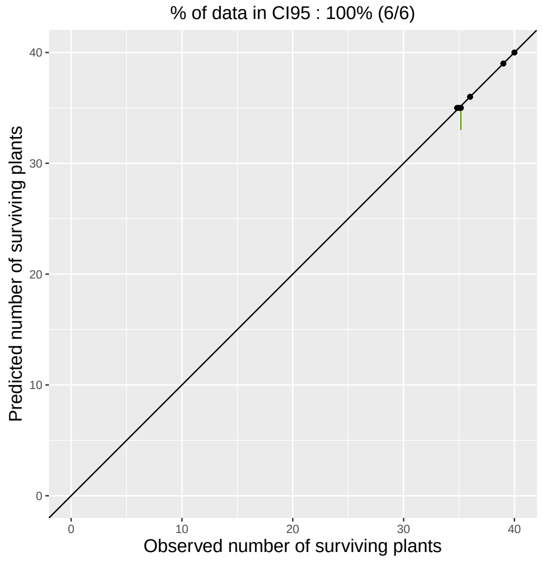

(b) Posterior predictive check (PPC)

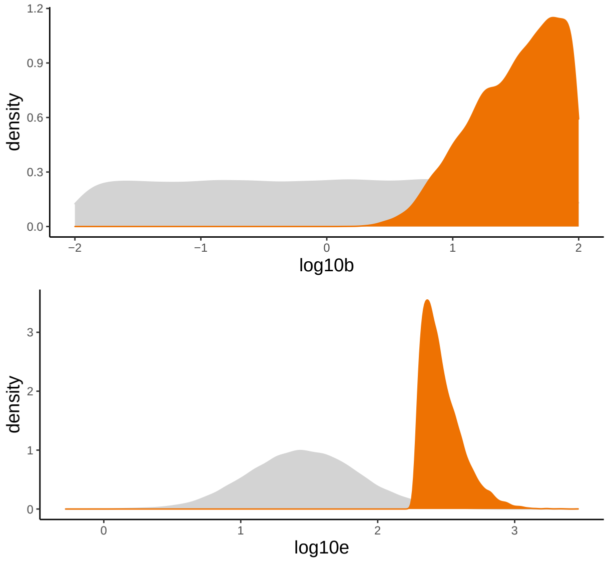

(c) Priors and posteriors

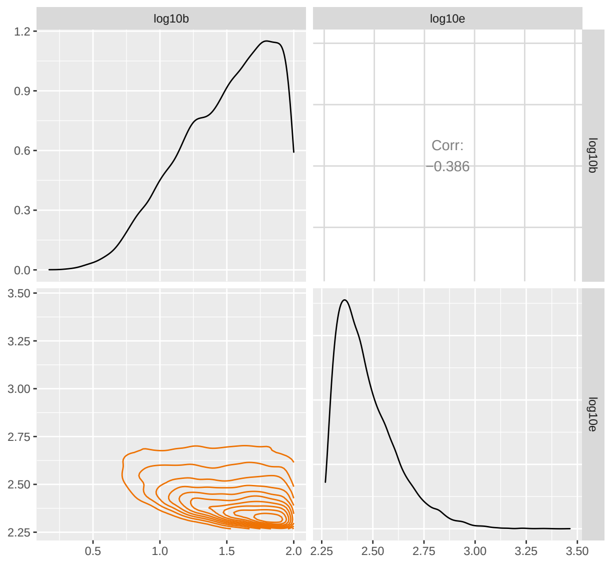

(d) Correlations between parameters

Figure 6: Dose-response curve (a), PPC (b), prior and posterior distributions (c) and correlations between parameters (d).

Data set: HELAN_SE_survival

Table 7: Summary of parameter estimates (parameter d is set to 1) for HELAN_SE_survival data set

Parameter	median	Q2.5	Q97.5
b	34.792	5.485	95.020
e	275.965	196.376	683.402

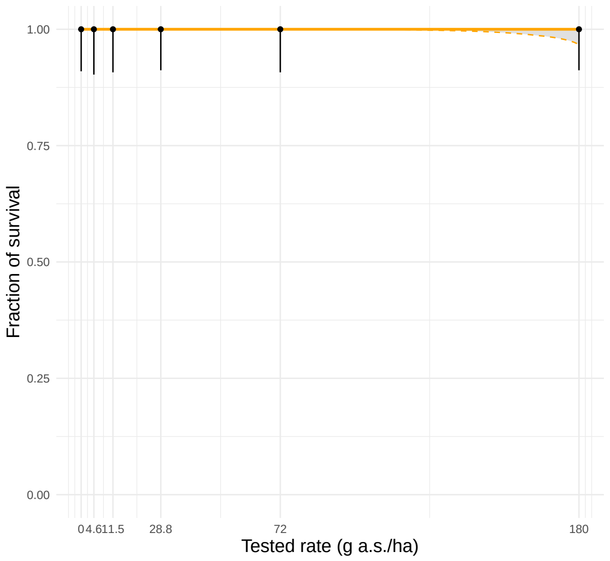

(a) Dose-response curve

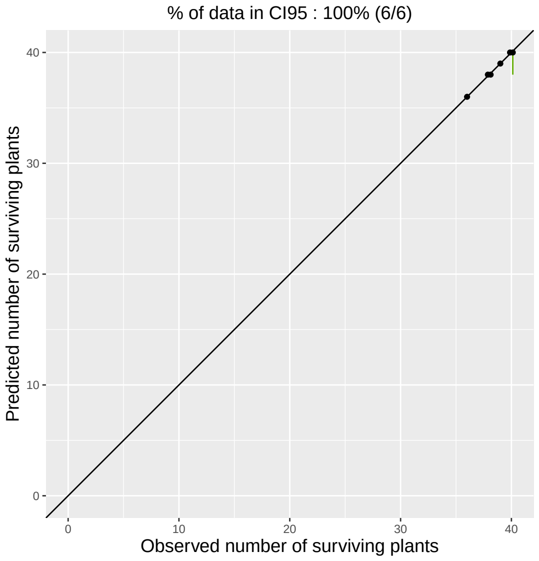

(b) Posterior predictive check (PPC)

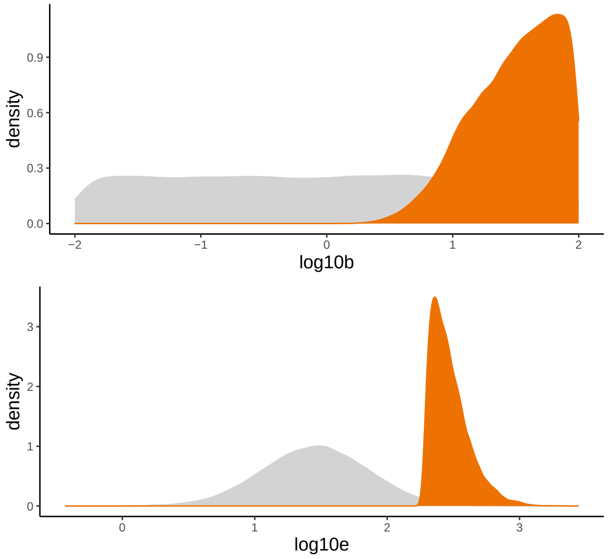

(c) Priors and posteriors

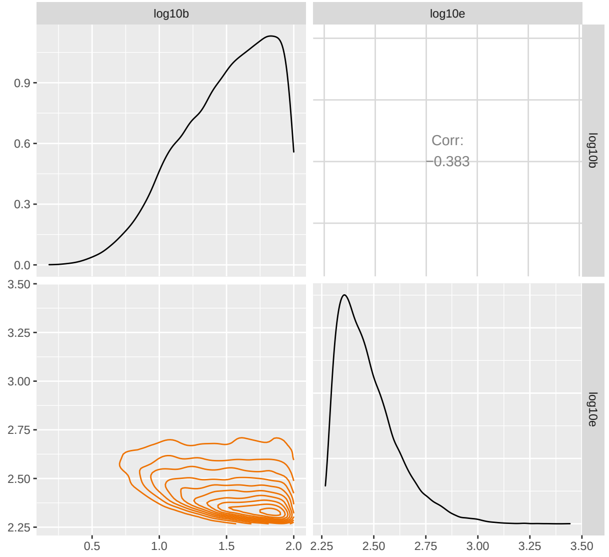

(d) Correlations between parameters

Figure 7: Dose-response curve (a), PPC (b), prior and posterior distributions (c) and correlations between parameters (d).

Data set: LOLPE_SE_survival

Table 8: Summary of parameter estimates (parameter d is set to 1) for LOLPE_SE_survival data set

Parameter	median	Q2.5	Q97.5
b	2.396	1.745	3.238
e	30.701	25.012	37.963

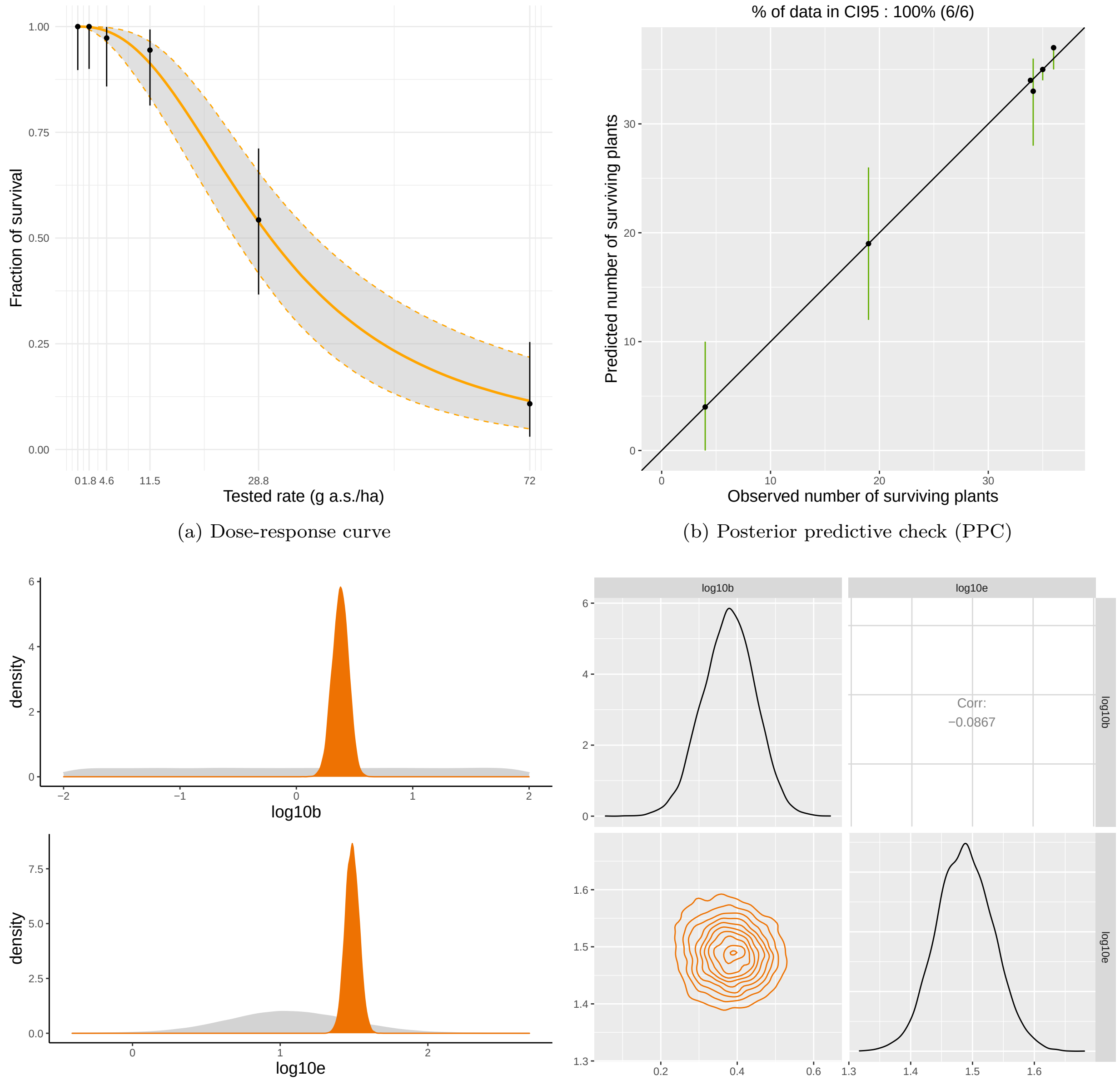

(a) Dose-response curve

(b) Posterior predictive check (PPC)

(c) Priors and posteriors

(d) Correlations between parameters

Figure 8: Dose-response curve (a), PPC (b), prior and posterior distributions (c) and correlations between parameters (d).

Data set: LYPES_SE_survival

Table 9: Summary of parameter estimates (parameter d is set to 1) for LYPES_SE_survival data set

Parameter	median	Q2.5	Q97.5
b	9.574	2.477	70.945
e	232.654	186.275	491.065

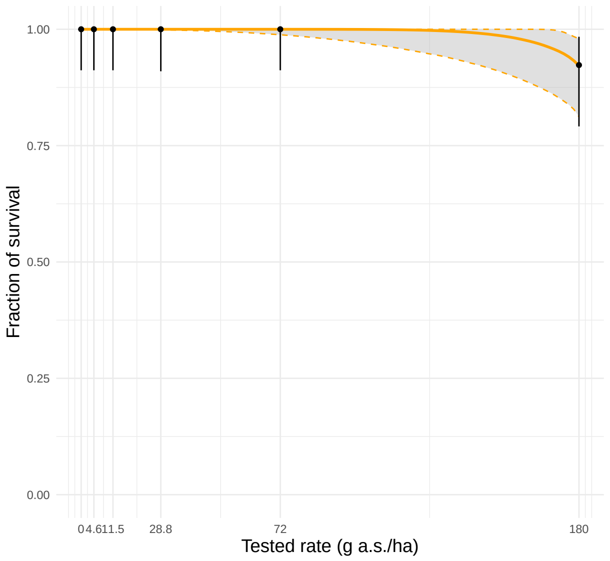

(a) Dose-response curve

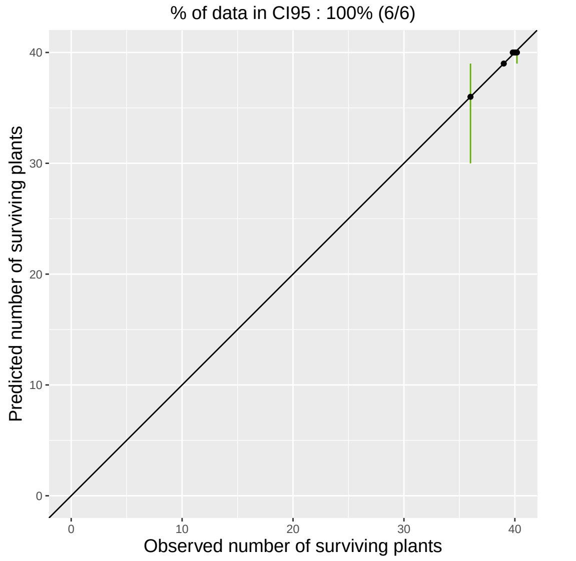

(b) Posterior predictive check (PPC)

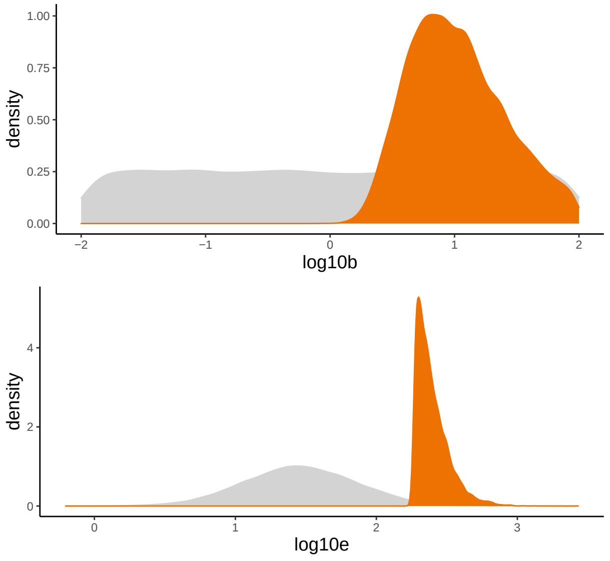

(c) Priors and posteriors

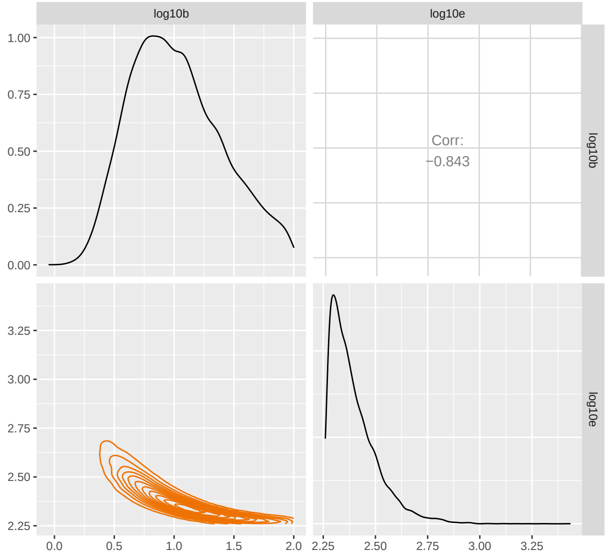

(d) Correlations between parameters

Figure 9: Dose-response curve (a), PPC (b), prior and posterior distributions (c) and correlations between parameters (d).

Data set: ZEAMA_SE_survival

Table 10: Summary of parameter estimates (parameter d is set to 1) for ZEAMA_SE_survival data set

Parameter	median	Q2.5	Q97.5
b	35.654	5.445	95.026
e	274.483	195.217	695.887

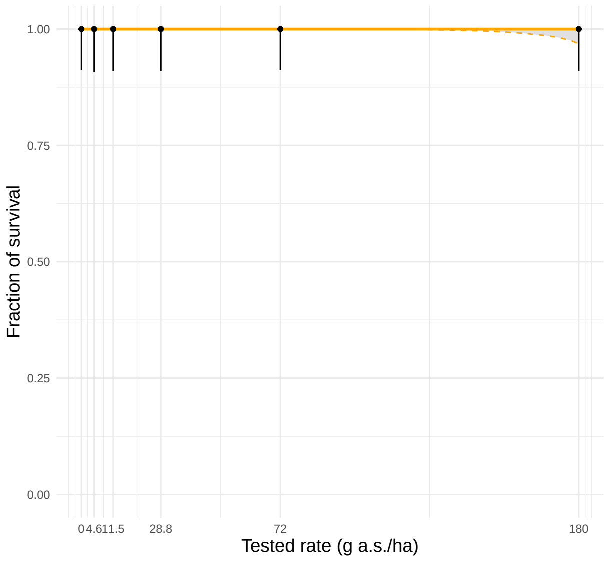

(a) Dose-response curve

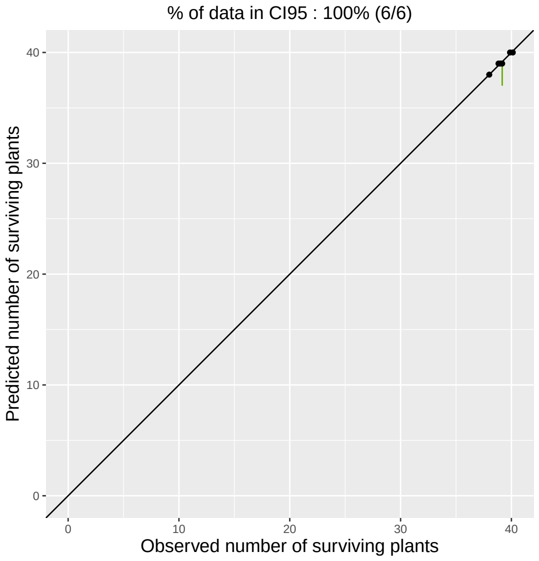

(b) Posterior predictive check (PPC)

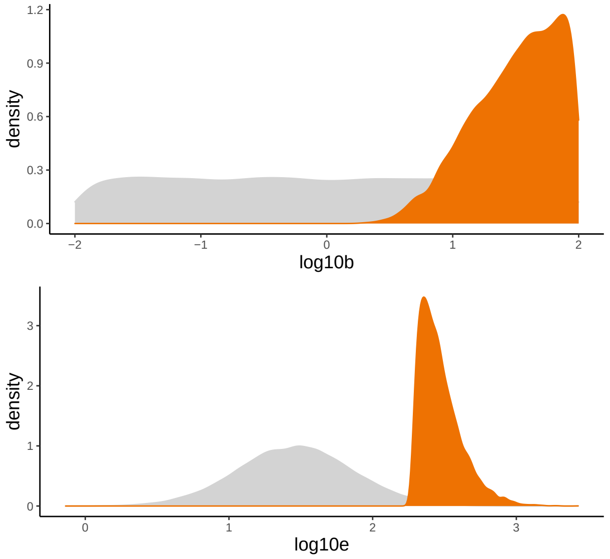

(c) Priors and posteriors

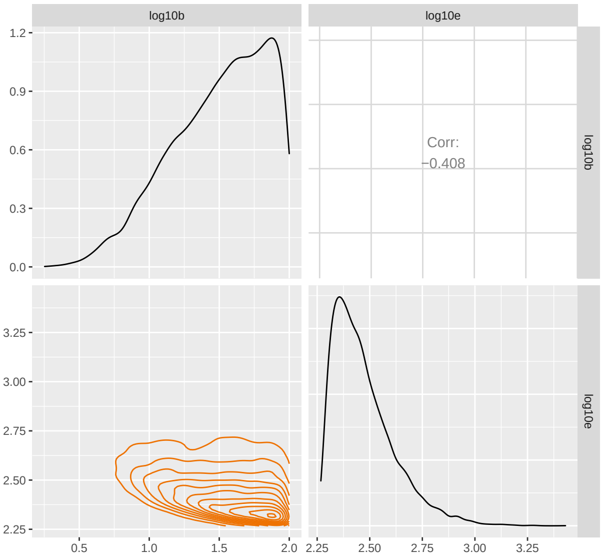

(d) Correlations between parameters

Figure 10: Dose-response curve (a), PPC (b), prior and posterior distributions (c) and correlations between parameters (d).
