# Supplementary material for: How to account for the uncertainty from standard toxicity tests in species sensitivity distributions: An example in non-target plants
Source: PLoS One. 2021 Jan 7;16(1):e0245071. doi: 10.1371/journal.pone.0245071 (PMC7790375; doi:10.1371/journal.pone.0245071)
Supplement: S1 Archive — It is a zip file containing seven folders (one folder per case study). Each folder contains five files report_xxx.pdf with detailed results of the dose-response analyses, one file corresponding to does-response analysis per endpoint. It also contains one file ER50_censoring.pdf for censored ER50 and one file SSD_analyses.pdf for results of SSD analyses. (ZIP) [file pone.0245071.s004.zip › S1_archive/Study2/report_SE_weight.pdf]

Dose-response analysis

Study 2

Seedling Emergence test - shoot dry SE_weight endpoint

25 June 2020

Contact: sandrine.charles@univ-lyon1.fr

This is a report which provides results on all performed dose-response analyses for the shoot dry SE_weight endpoint of the Seedling Emergence test for study 2.

Contents

Data set: ALLCE_SE_weight 2
Data set: AVESA_SE_weight 3
Data set: BEAVA_SE_weight 4
Data set: BRSNW_SE_weight 5
Data set: CUMSA_SE_weight 6
Data set: GLXMA_SE_weight 7
Data set: HELAN_SE_weight 8
Data set: LOLPE_SE_weight 9
Data set: LYPES_SE_weight 10
Data set: ZEAMA_SE_weight 11

Data set: ALLCE_SE_weight

Table 1: Summary of parameter estimates for ALLCE_SE_weight data set

Parameter	median	Q2.5	Q97.5
b	1.964	1.055	4.073
d	0.045	0.041	0.050
e	7.954	5.785	10.746
sigma	0.010	0.009	0.013

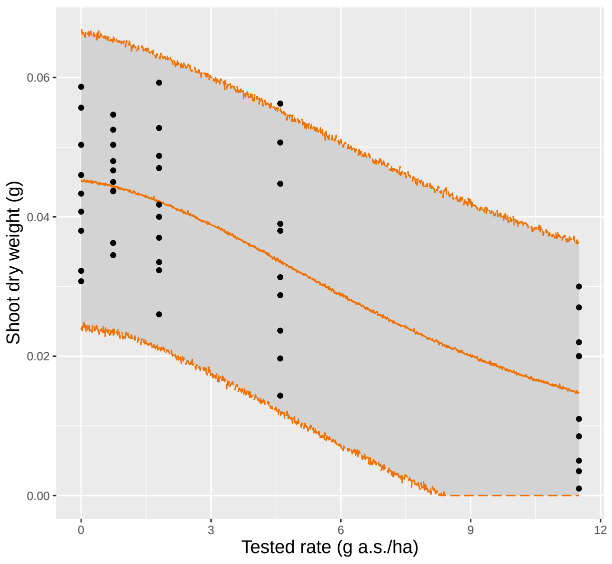

(a) Dose-response curve

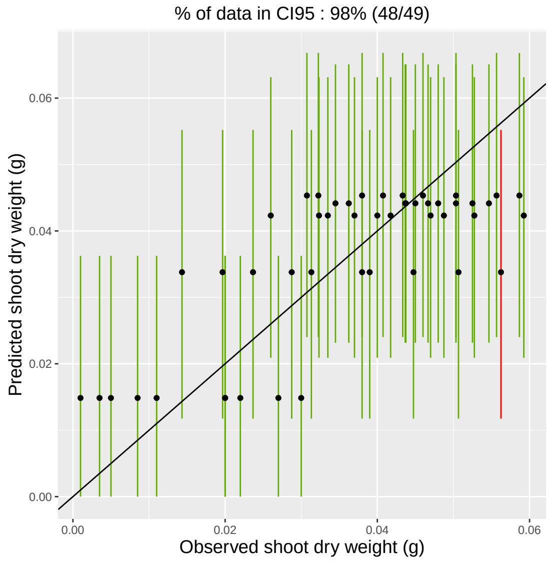

(b) Posterior predictive check (PPC)

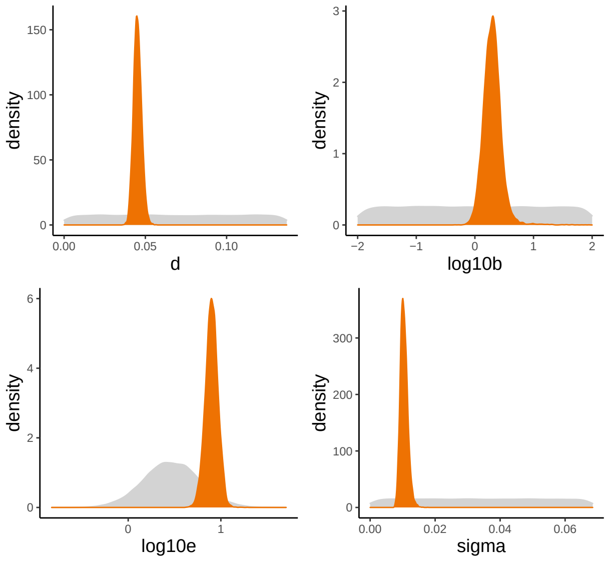

(c) Priors and posteriors

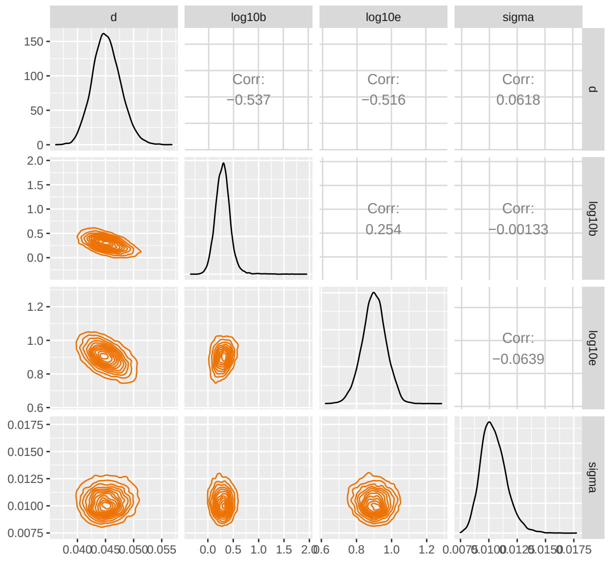

(d) Correlations between parameters

Figure 1: Dose-response curve (a), PPC (b), prior and posterior distributions (c) and correlations between parameters (d).

Data set: AVESA_SE_weight

Table 2: Summary of parameter estimates for AVESA_SE_weight data set

Parameter	median	Q2.5	Q97.5
b	0.971	0.548	1.707
d	0.599	0.552	0.660
e	217.238	147.926	374.312
sigma	0.102	0.086	0.125

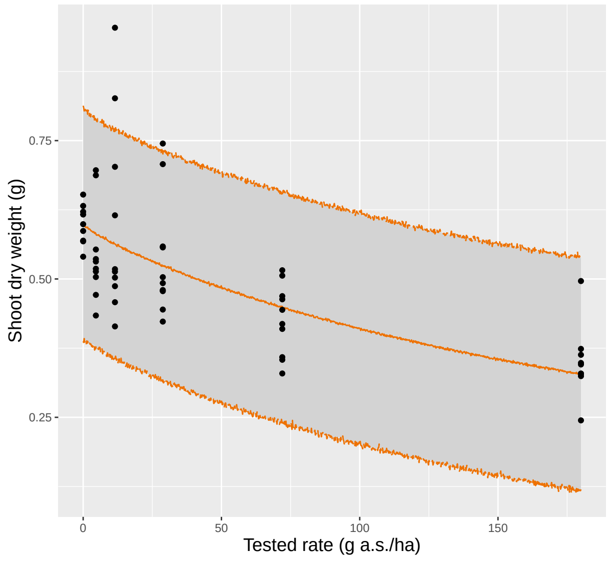

(a) Dose-response curve

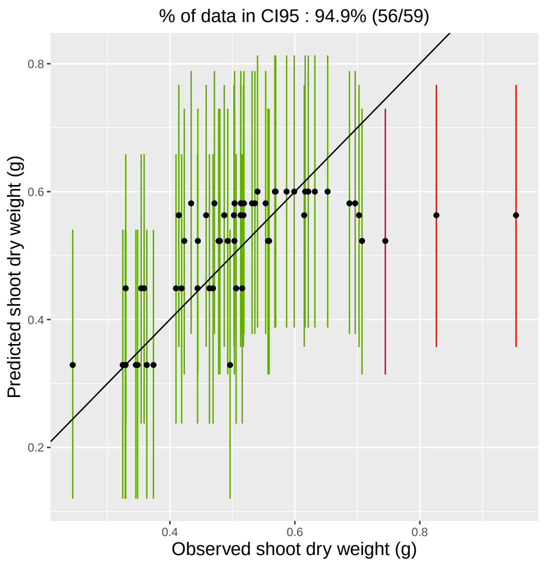

(b) Posterior predictive check (PPC)

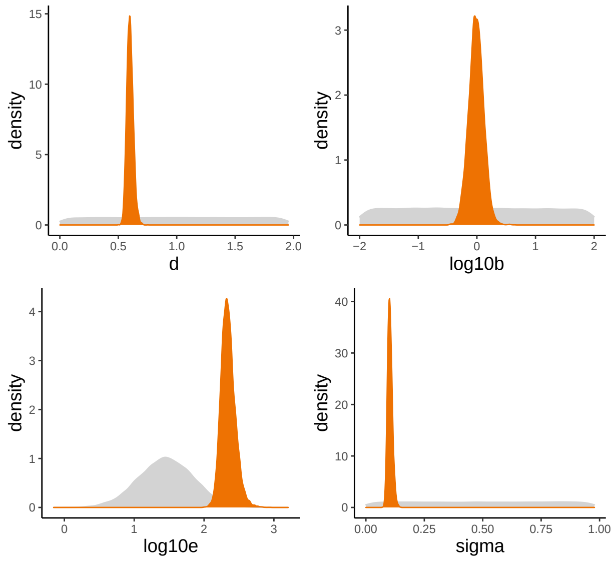

(c) Priors and posteriors

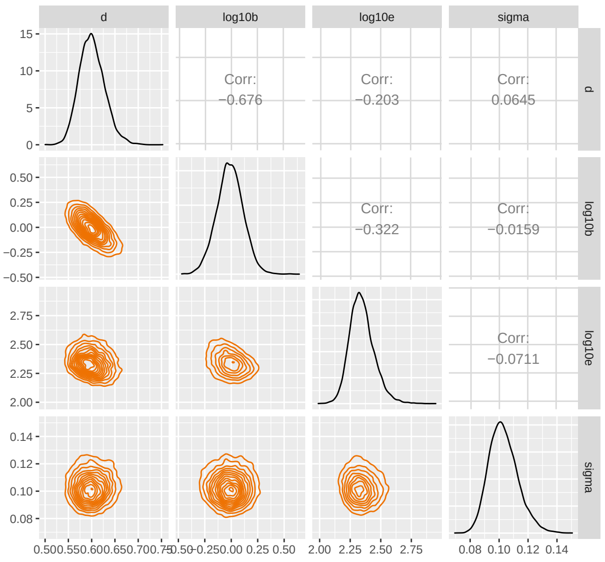

(d) Correlations between parameters

Figure 2: Dose-response curve (a), PPC (b), prior and posterior distributions (c) and correlations between parameters (d).

Data set: BEAVA_SE_weight

Table 3: Summary of parameter estimates for BEAVA_SE_weight data set

Parameter	median	Q2.5	Q97.5
b	5.248	1.150	64.725
d	1.014	0.957	1.073
e	38.374	29.507	88.484
sigma	0.256	0.226	0.293

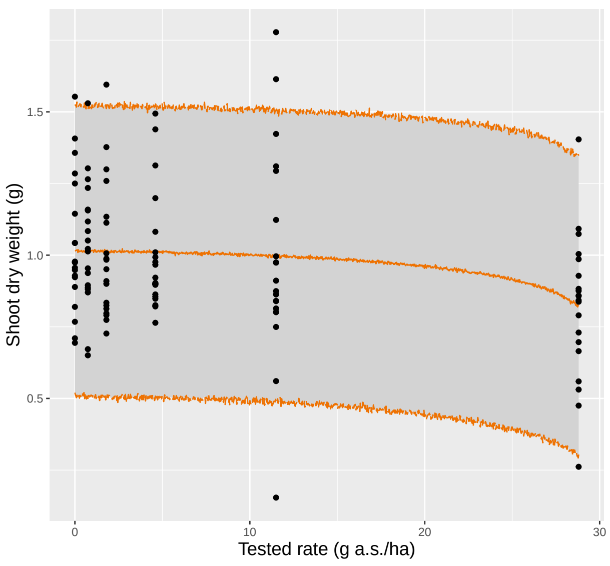

(a) Dose-response curve

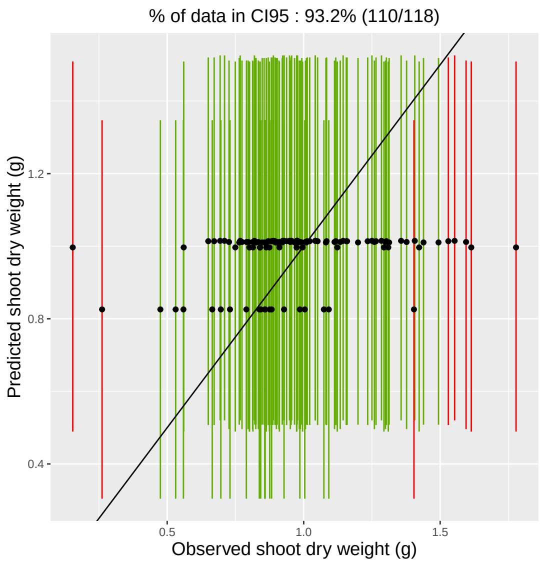

(b) Posterior predictive check (PPC)

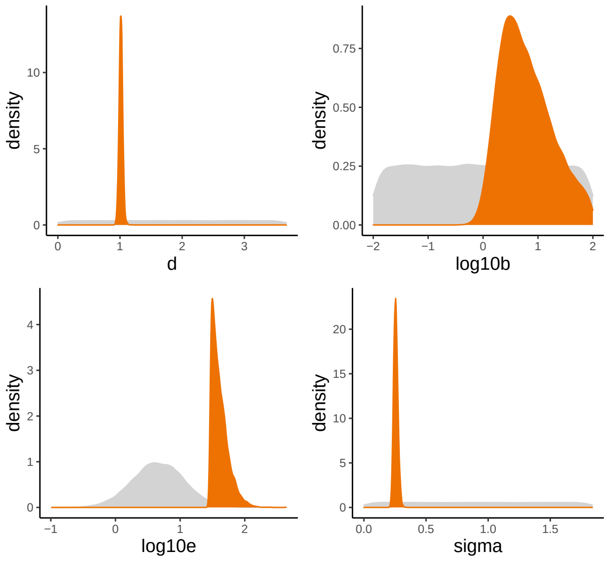

(c) Priors and posteriors

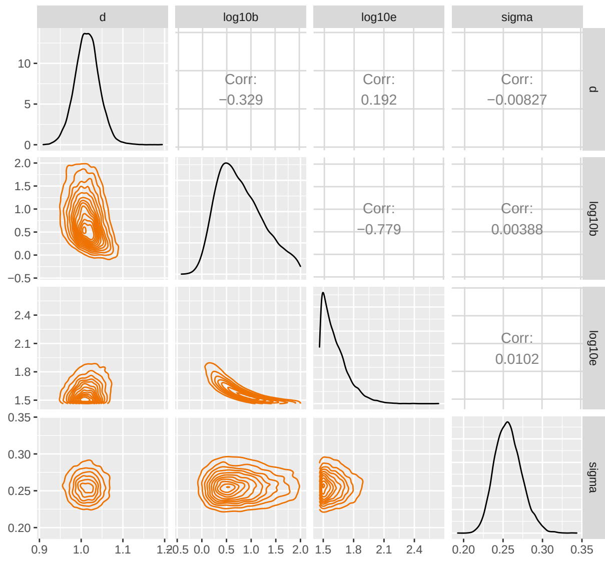

(d) Correlations between parameters

Figure 3: Dose-response curve (a), PPC (b), prior and posterior distributions (c) and correlations between parameters (d).

Data set: BRSNW_SE_weight

Table 4: Summary of parameter estimates for BRSNW_SE_weight data set

Parameter	median	Q2.5	Q97.5
b	0.965	0.586	1.770
d	1.321	1.220	1.443
e	208.450	143.197	357.522
sigma	0.314	0.276	0.358

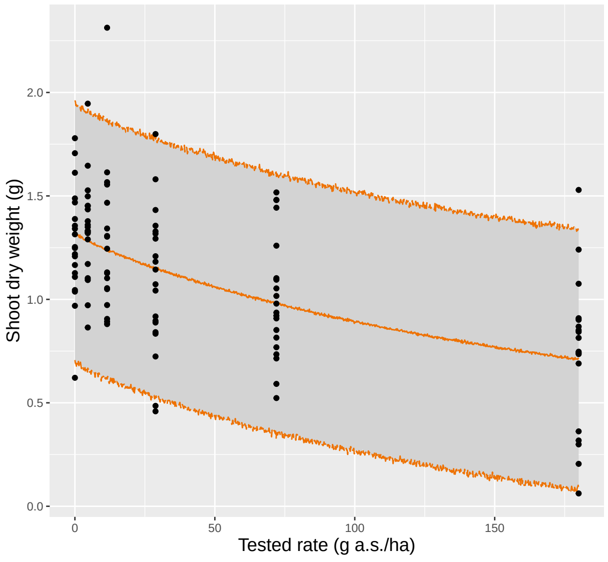

(a) Dose-response curve

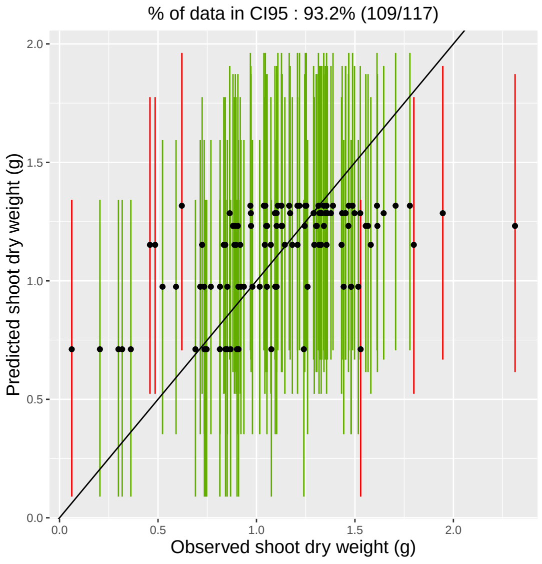

(b) Posterior predictive check (PPC)

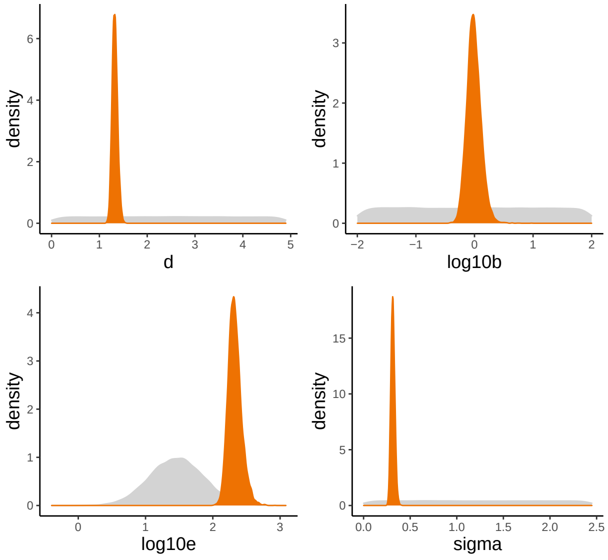

(c) Priors and posteriors

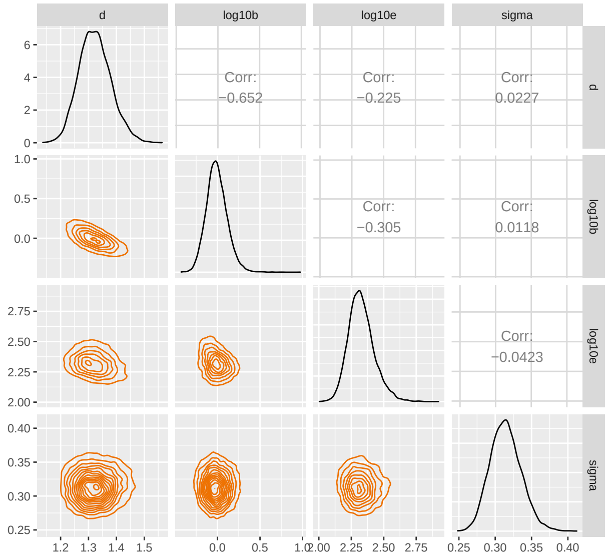

(d) Correlations between parameters

Figure 4: Dose-response curve (a), PPC (b), prior and posterior distributions (c) and correlations between parameters (d).

Data set: CUMSA_SE_weight

Table 5: Summary of parameter estimates for CUMSA_SE_weight data set

Parameter	median	Q2.5	Q97.5
b	0.904	0.628	1.315
d	2.871	2.614	3.137
e	51.999	36.541	75.689
sigma	0.654	0.576	0.750

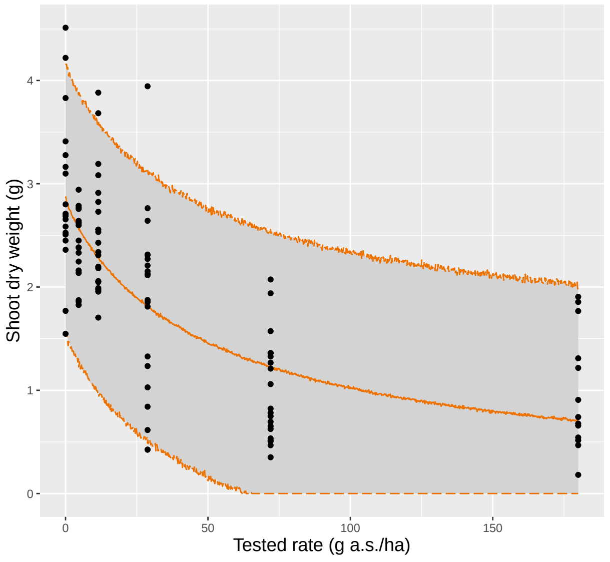

(a) Dose-response curve

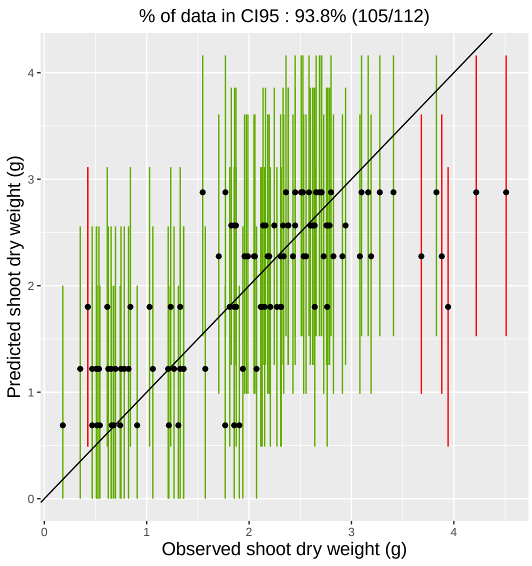

(b) Posterior predictive check (PPC)

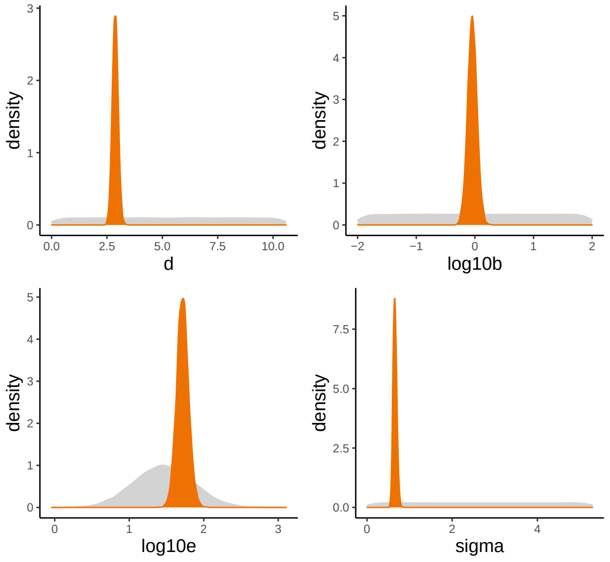

(c) Priors and posteriors

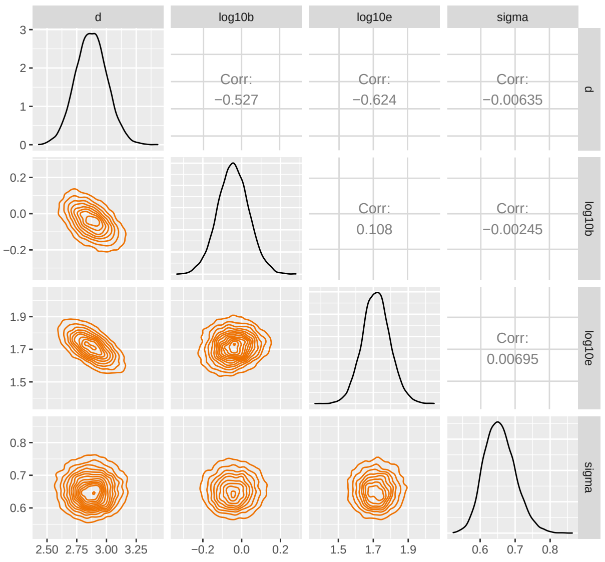

(d) Correlations between parameters

Figure 5: Dose-response curve (a), PPC (b), prior and posterior distributions (c) and correlations between parameters (d).

Data set: GLXMA_SE_weight

Table 6: Summary of parameter estimates for GLXMA_SE_weight data set

Parameter	median	Q2.5	Q97.5
b	25.033	3.336	93.096
d	2.197	2.116	2.284
e	259.458	189.967	632.591
sigma	0.437	0.386	0.501

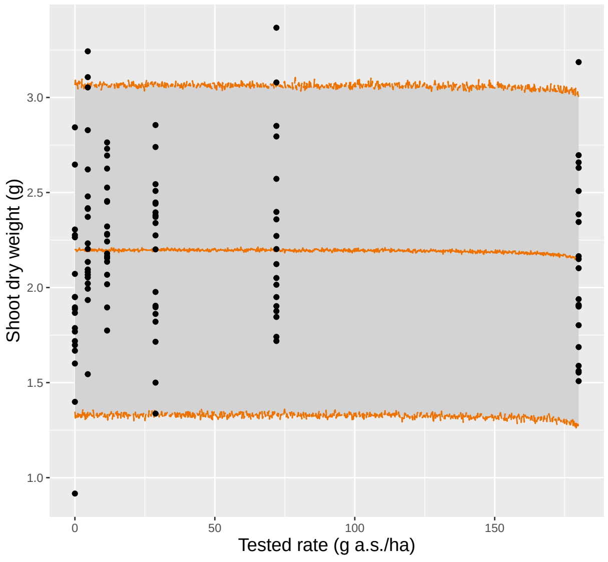

(a) Dose-response curve

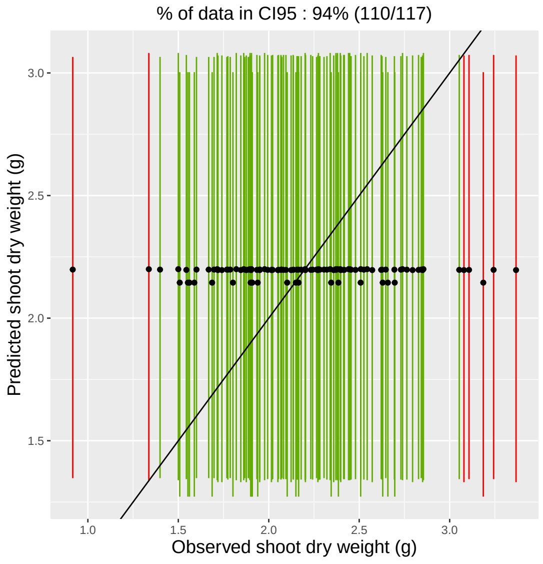

(b) Posterior predictive check (PPC)

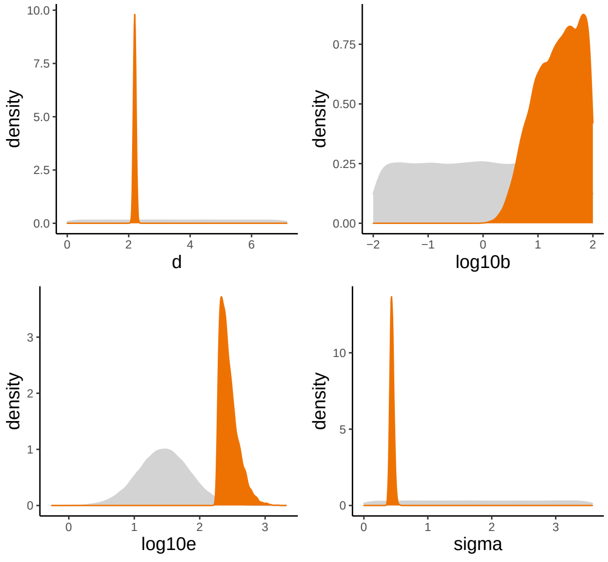

(c) Priors and posteriors

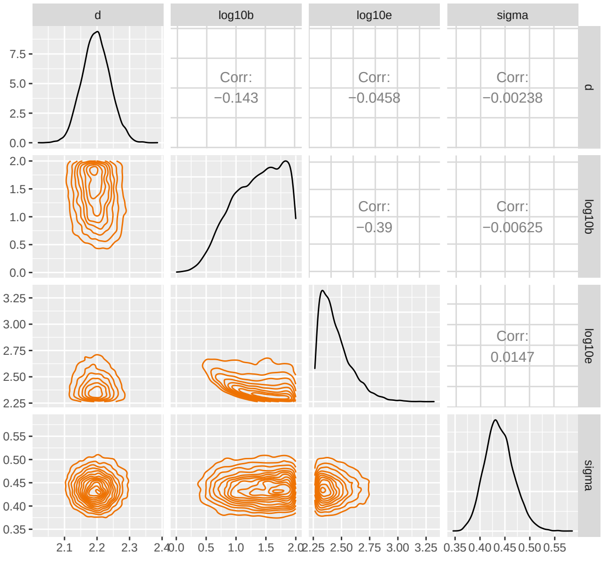

(d) Correlations between parameters

Figure 6: Dose-response curve (a), PPC (b), prior and posterior distributions (c) and correlations between parameters (d).

Data set: HELAN_SE_weight

Table 7: Summary of parameter estimates for HELAN_SE_weight data set

Parameter	median	Q2.5	Q97.5
b	33.124	4.954	94.387
d	0.551	0.530	0.570
e	272.888	195.420	674.027
sigma	0.109	0.097	0.125

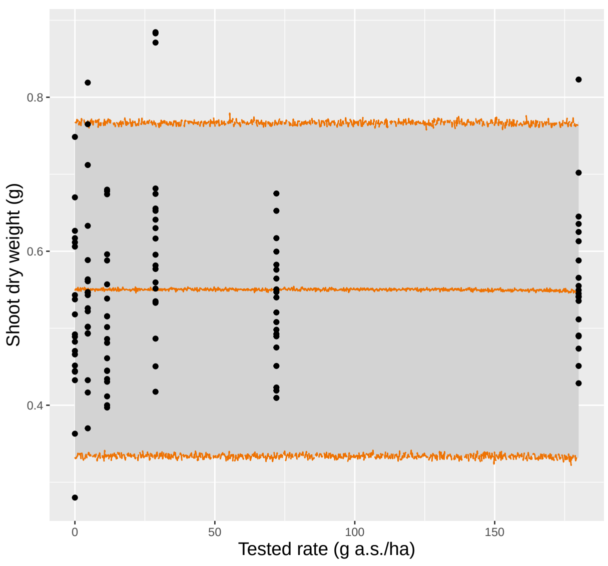

(a) Dose-response curve

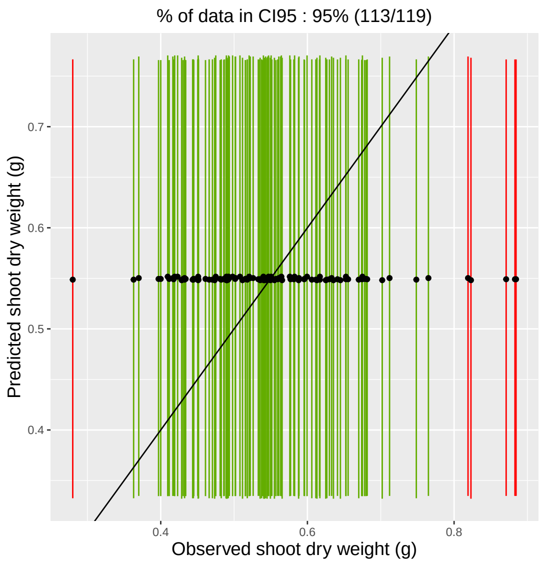

(b) Posterior predictive check (PPC)

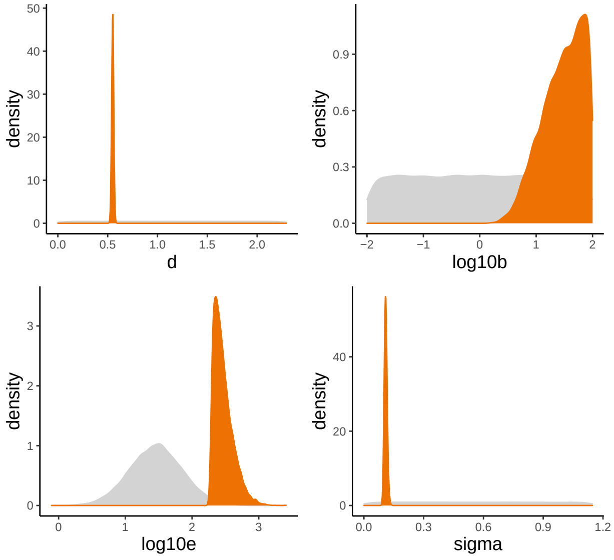

(c) Priors and posteriors

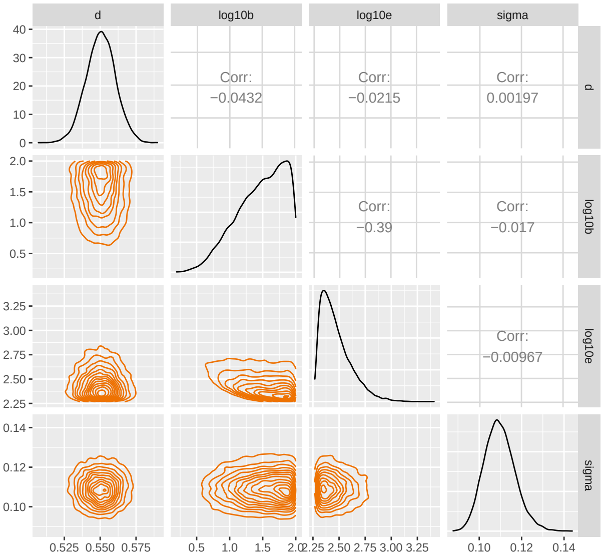

(d) Correlations between parameters

Figure 7: Dose-response curve (a), PPC (b), prior and posterior distributions (c) and correlations between parameters (d).

Data set: LOLPE_SE_weight

Table 8: Summary of parameter estimates for LOLPE_SE_weight data set

Parameter	median	Q2.5	Q97.5
b	4.431	1.384	56.887
d	0.104	0.094	0.114
e	74.133	51.281	137.118
sigma	0.031	0.026	0.039

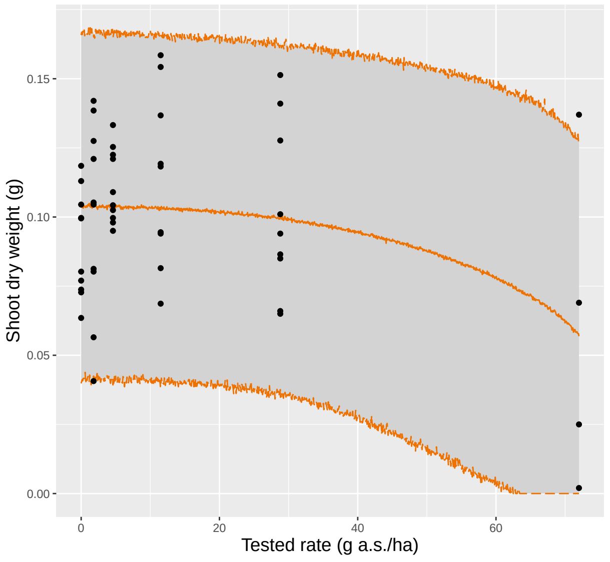

(a) Dose-response curve

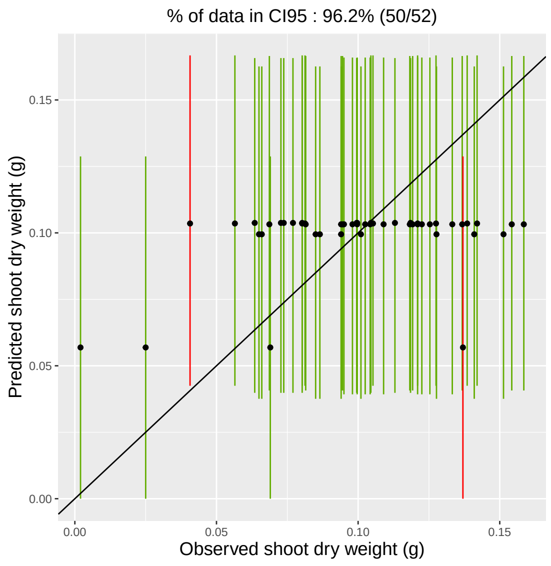

(b) Posterior predictive check (PPC)

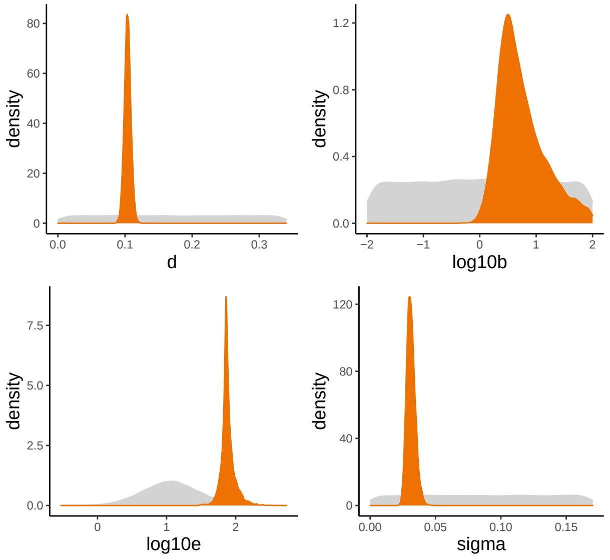

(c) Priors and posteriors

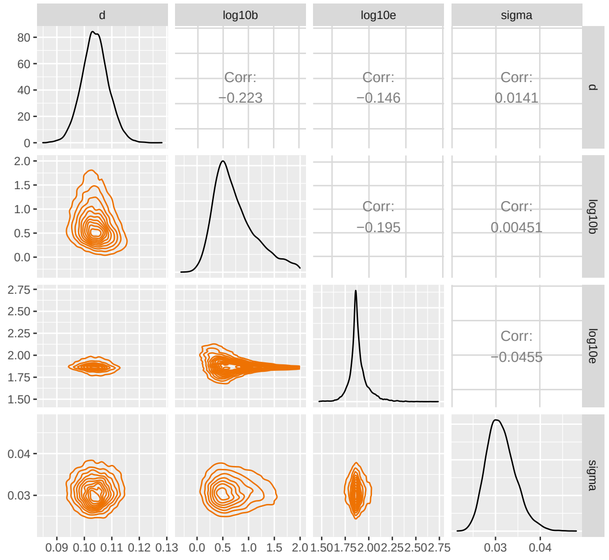

(d) Correlations between parameters

Figure 8: Dose-response curve (a), PPC (b), prior and posterior distributions (c) and correlations between parameters (d).

Data set: LYPES_SE_weight

Table 9: Summary of parameter estimates for LYPES_SE_weight data set

Parameter	median	Q2.5	Q97.5
b	0.889	0.620	1.321
d	1.286	1.187	1.396
e	103.170	74.600	144.647
sigma	0.258	0.228	0.296

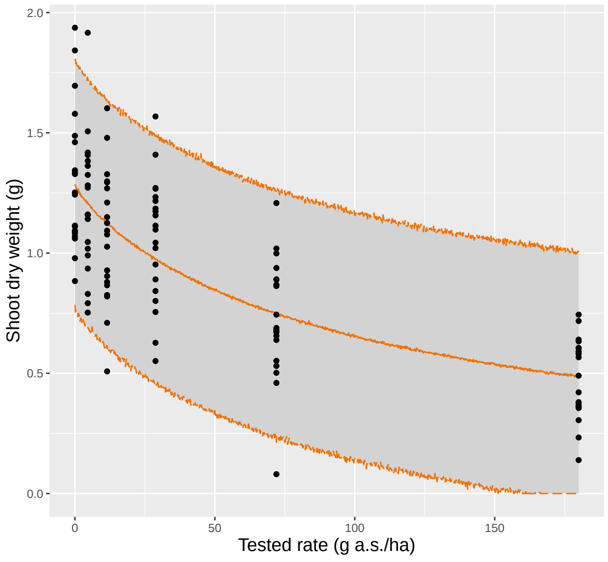

(a) Dose-response curve

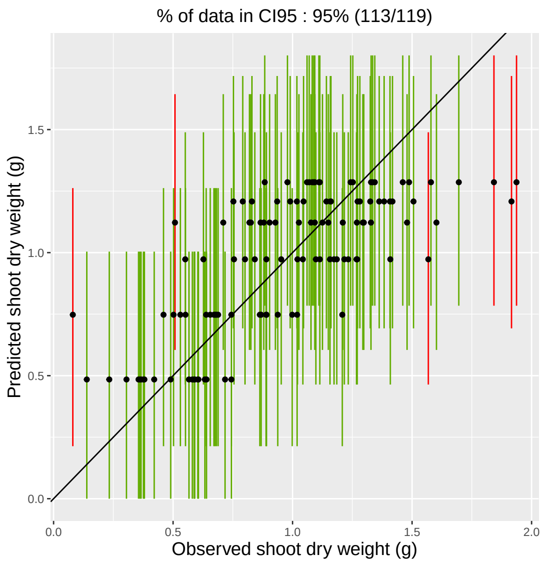

(b) Posterior predictive check (PPC)

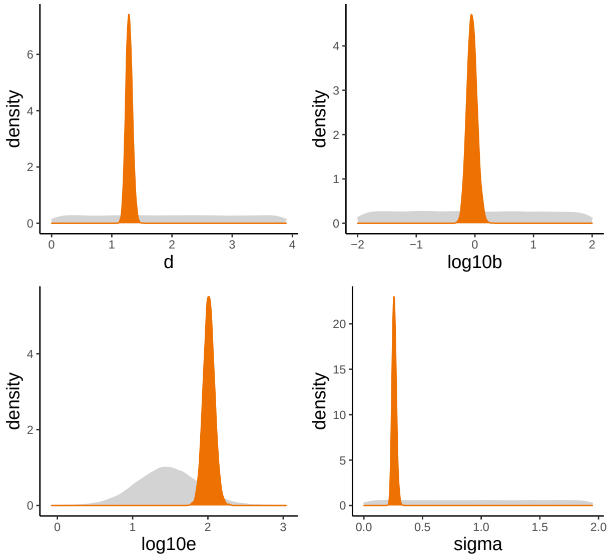

(c) Priors and posteriors

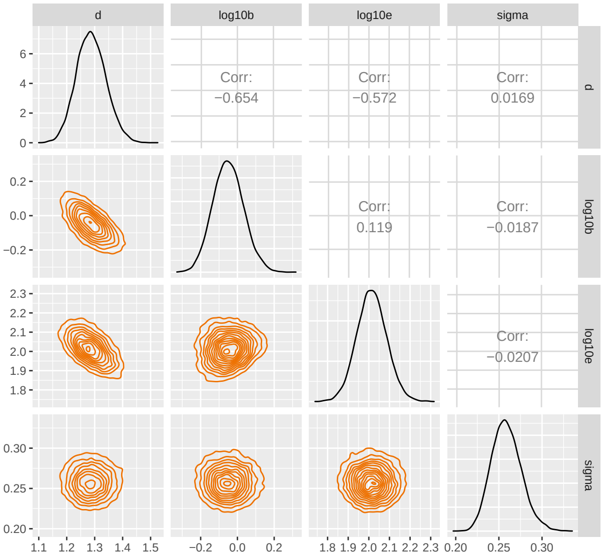

(d) Correlations between parameters

Figure 9: Dose-response curve (a), PPC (b), prior and posterior distributions (c) and correlations between parameters (d).

Data set: ZEAMA_SE_weight

Table 10: Summary of parameter estimates for ZEAMA_SE_weight data set

Parameter	median	Q2.5	Q97.5
b	17.004	2.717	91.516
d	3.783	3.674	3.895
e	252.786	188.393	622.364
sigma	0.554	0.489	0.633

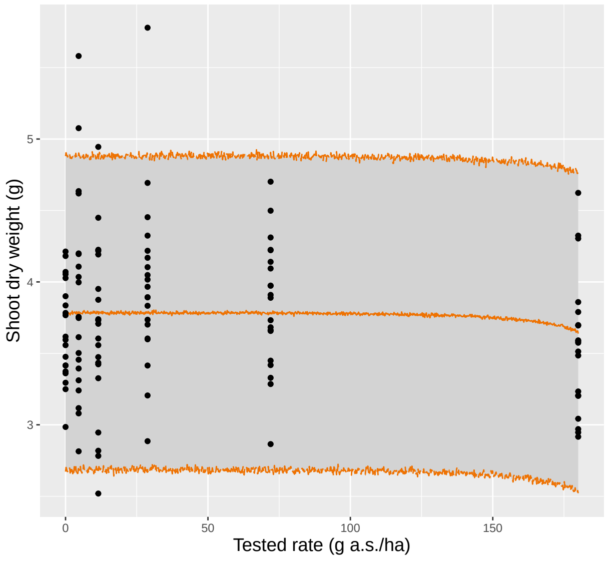

(a) Dose-response curve

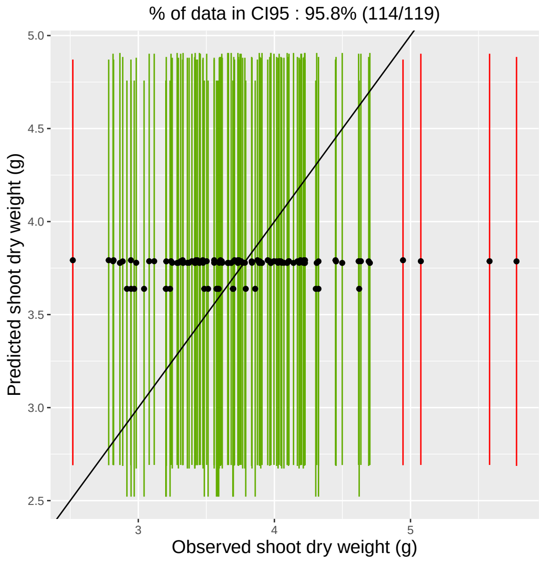

(b) Posterior predictive check (PPC)

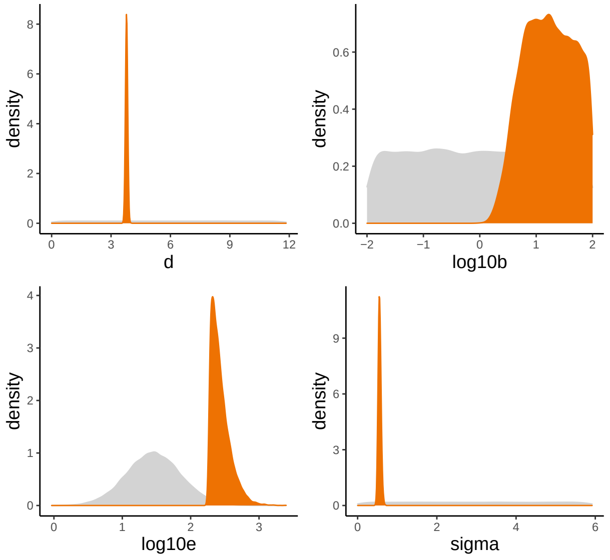

(c) Priors and posteriors

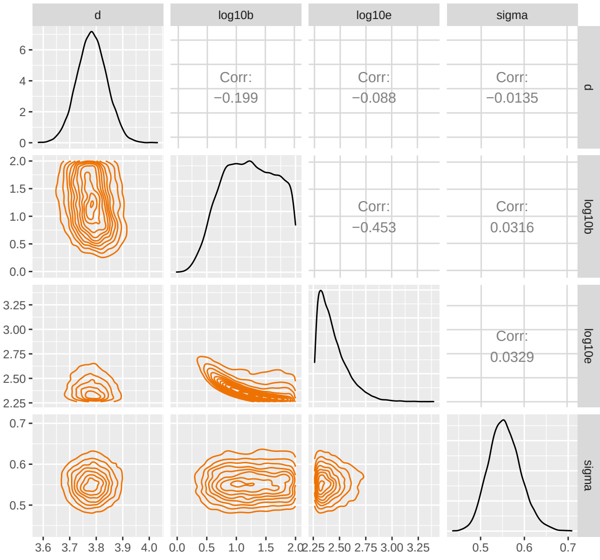

(d) Correlations between parameters

Figure 10: Dose-response curve (a), PPC (b), prior and posterior distributions (c) and correlations between parameters (d).
