# Supplementary material for: How to account for the uncertainty from standard toxicity tests in species sensitivity distributions: An example in non-target plants
Source: PLoS One. 2021 Jan 7;16(1):e0245071. doi: 10.1371/journal.pone.0245071 (PMC7790375; doi:10.1371/journal.pone.0245071)
Supplement: S1 Archive — It is a zip file containing seven folders (one folder per case study). Each folder contains five files report_xxx.pdf with detailed results of the dose-response analyses, one file corresponding to does-response analysis per endpoint. It also contains one file ER50_censoring.pdf for censored ER50 and one file SSD_analyses.pdf for results of SSD analyses. (ZIP) [file pone.0245071.s004.zip › S1_archive/Study2/report_VV_survival.pdf]

Dose-response analyses
Study 2
Vegetative Vigour test - survival endpoint

25 June 2020

Contact: sandrine.charles@univ-lyon1.fr

This is a report which provides results on all performed dose-response analyses for the survival endpoint of the Vegetative Vigour test for study 2.

Contents

Data set: ALLCE_VV_survival . . . 2
Data set: AVESA_VV_survival . . . 3
Data set: BEAVA_VV_survival . . . 4
Data set: BRSNW_VV_survival . . . 5
Data set: CUMSA_VV_survival . . . 6
Data set: GLXMA_VV_survival . . . 7
Data set: HELAN_VV_survival . . . 8
Data set: LOLPE_VV_survival . . . 9
Data set: LYPES_VV_survival . . . 10
Data set: ZEAMA_VV_survival . . . 11

Data set: ALLCE_VV_survival

Table 1: Summary of parameter estimates for ALLCE_VV_survival data set

Parameter	median	Q2.5	Q97.5
b	34.476	5.178	95.414
d	0.976	0.948	0.991
e	272.080	195.030	704.008

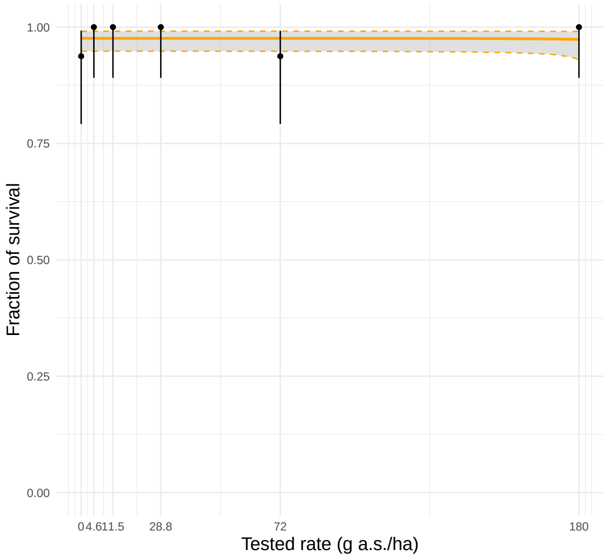

(a) Dose-response curve

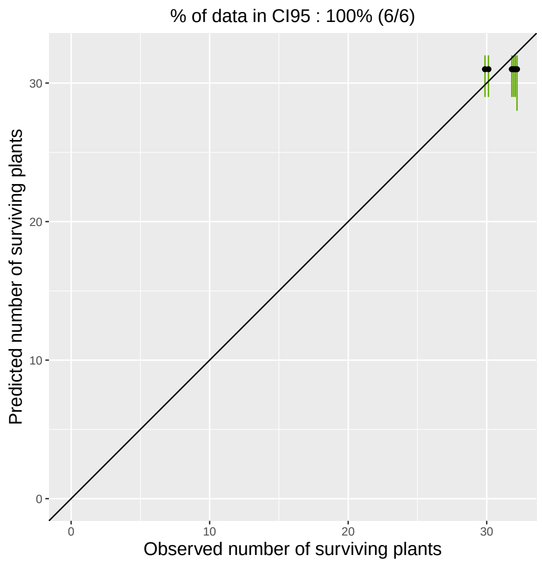

(b) Posterior predictive check (PPC)

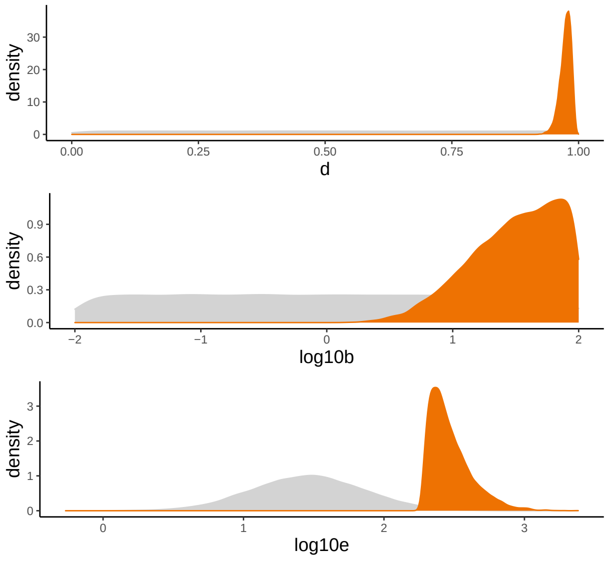

(c) Priors and posteriors

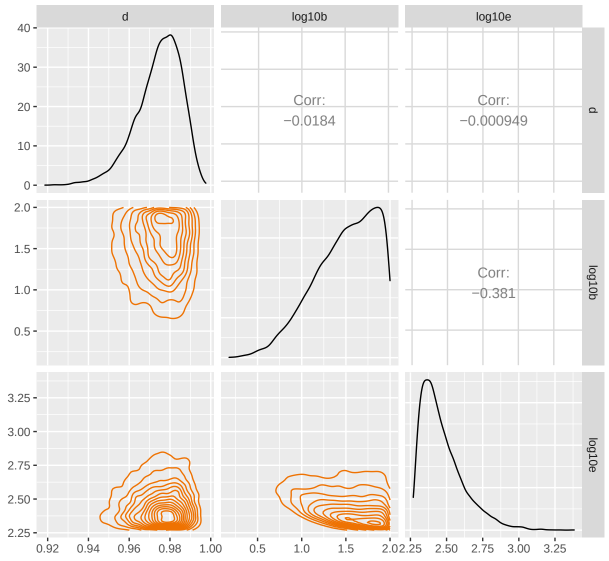

(d) Correlations between parameters

Figure 1: Dose-response curve (a), PPC (b), prior and posterior distributions (c) and correlations between parameters (d).

Data set: AVESA_VV_survival

Table 2: Summary of parameter estimates (parameter d is set to 1) for AVESA_VV_survival data set

Parameter	median	Q2.5	Q97.5
b	1.750	1.082	2.917
e	765.482	384.854	1993.331

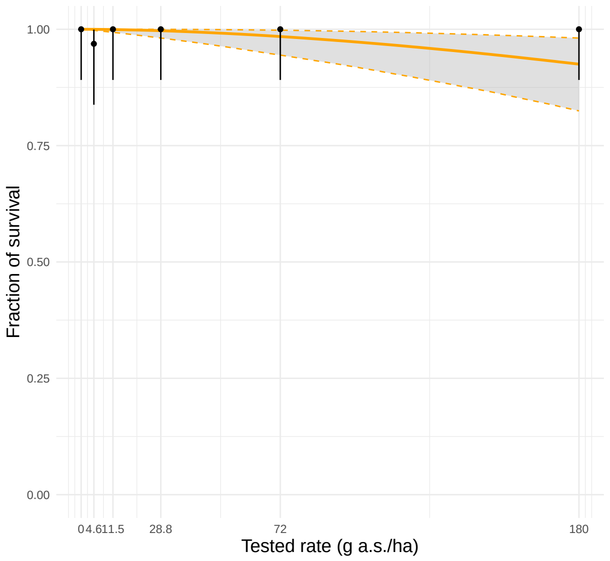

(a) Dose-response curve

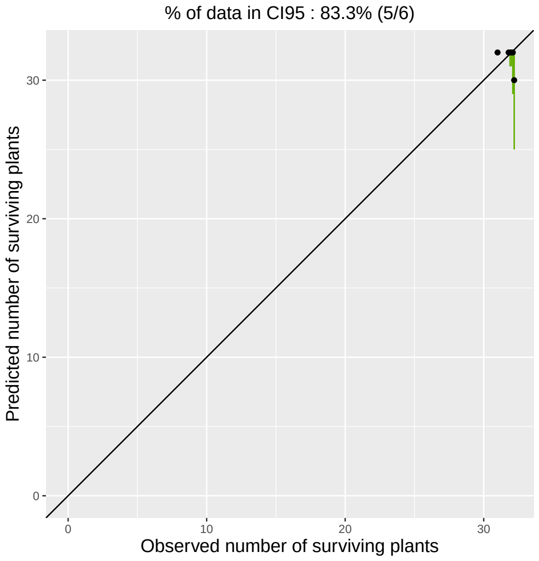

(b) Posterior predictive check (PPC)

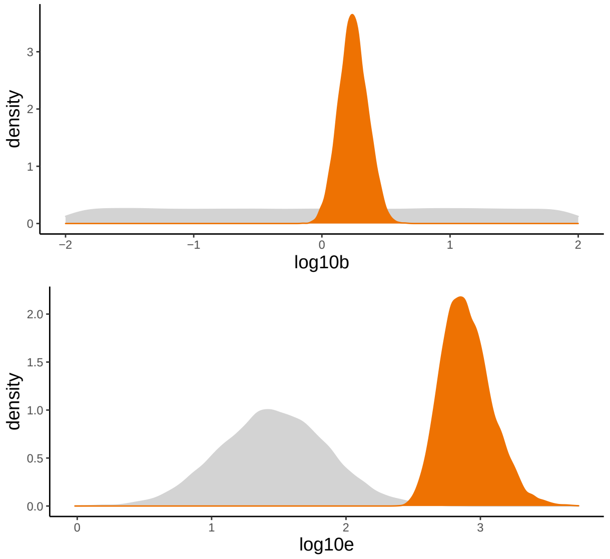

(c) Priors and posteriors

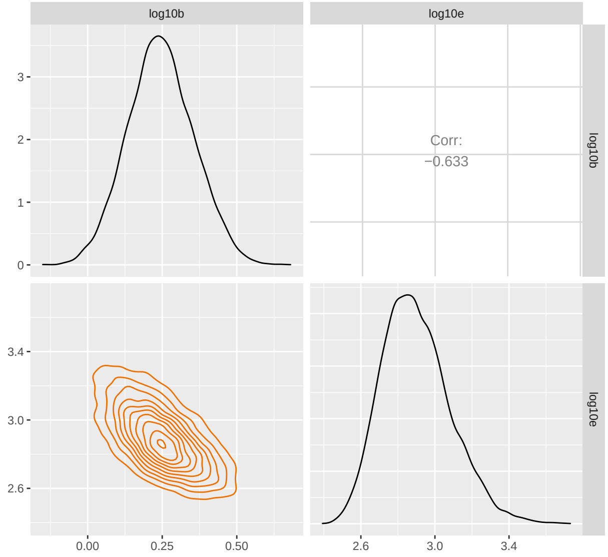

(d) Correlations between parameters

Figure 2: Dose-response curve (a), PPC (b), prior and posterior distributions (c) and correlations between parameters (d).

Data set: BEAVA_VV_survival

Table 3: Summary of parameter estimates (parameter d is set to 1) for BEAVA_VV_survival data set

Parameter	median	Q2.5	Q97.5
b	34.179	5.143	95.256
e	273.887	195.183	695.467

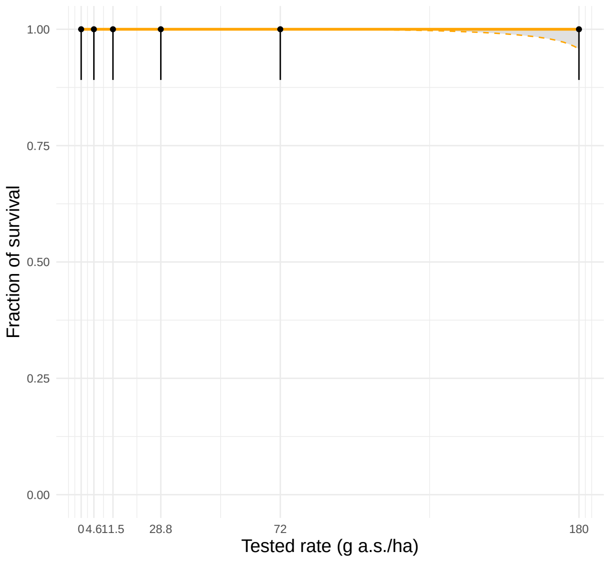

(a) Dose-response curve

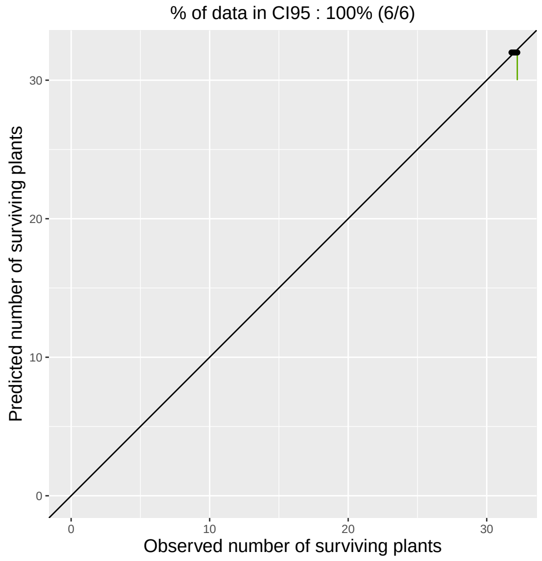

(b) Posterior predictive check (PPC)

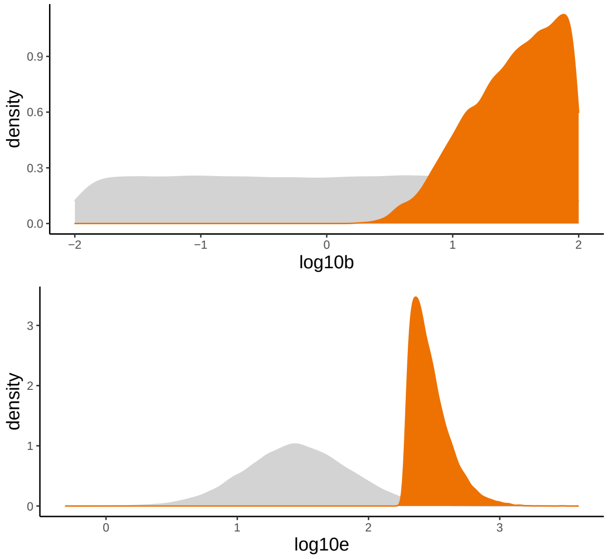

(c) Priors and posteriors

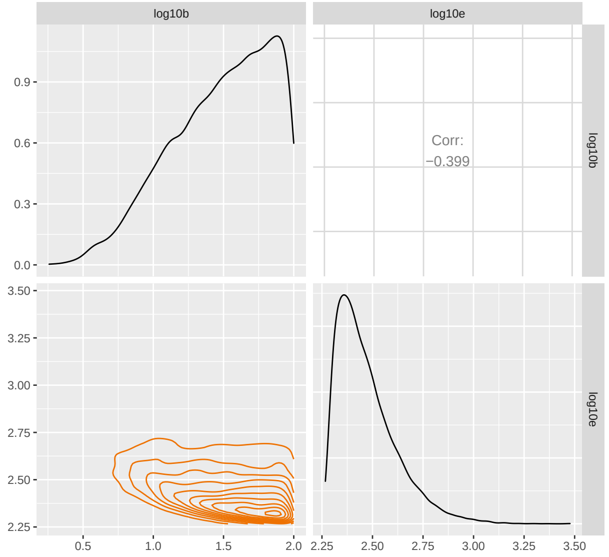

(d) Correlations between parameters

Figure 3: Dose-response curve (a), PPC (b), prior and posterior distributions (c) and correlations between parameters (d).

Data set: BRSNW_VV_survival

Table 4: Summary of parameter estimates (parameter d is set to 1) for BRSNW_VV_survival data set

Parameter	median	Q2.5	Q97.5
b	34.834	5.390	94.880
e	272.166	195.255	674.817

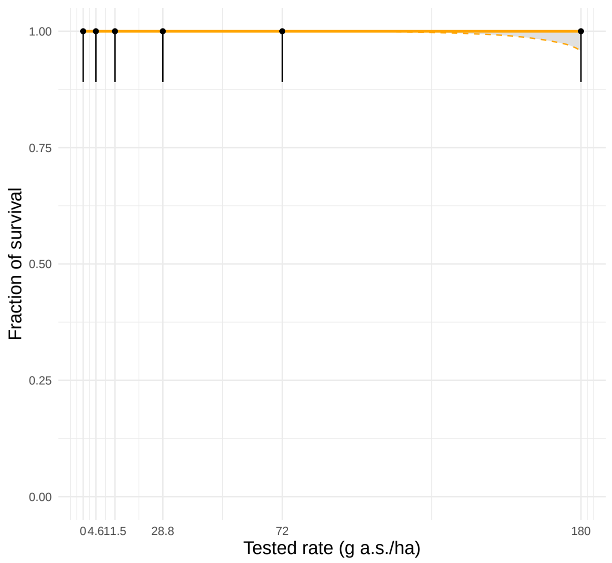

(a) Dose-response curve

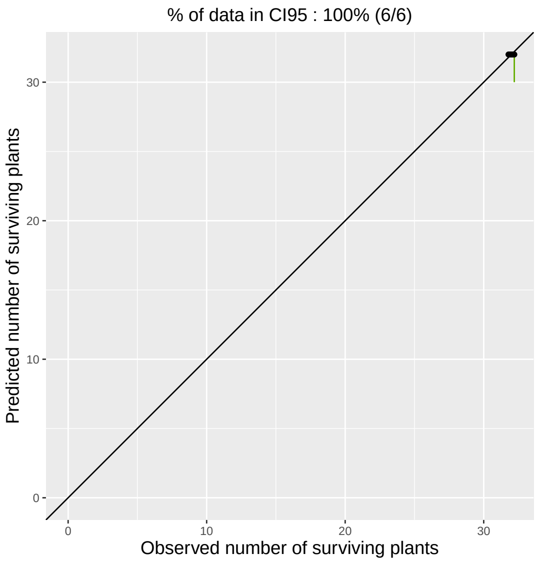

(b) Posterior predictive check (PPC)

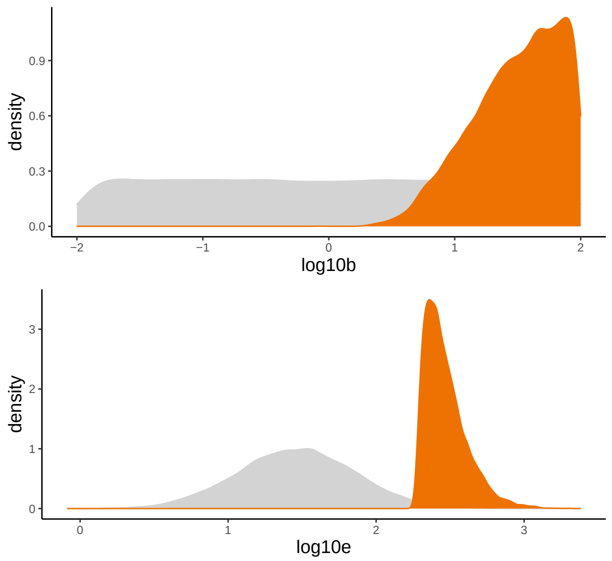

(c) Priors and posteriors

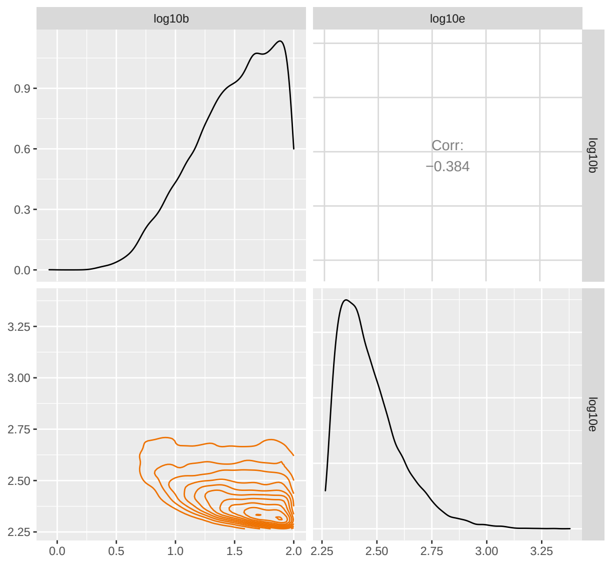

(d) Correlations between parameters

Figure 4: Dose-response curve (a), PPC (b), prior and posterior distributions (c) and correlations between parameters (d).

Data set: CUMSA_VV_survival

Table 5: Summary of parameter estimates (parameter d is set to 1) for CUMSA_VV_survival data set

Parameter	median	Q2.5	Q97.5
b	35.228	5.294	94.847
e	273.302	194.944	667.065

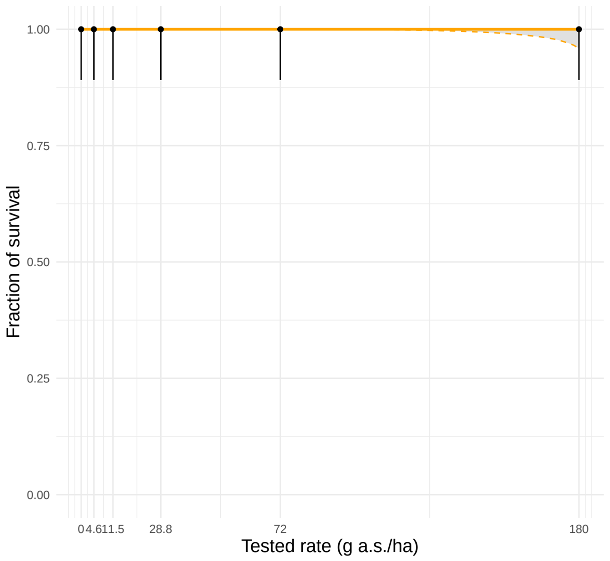

(a) Dose-response curve

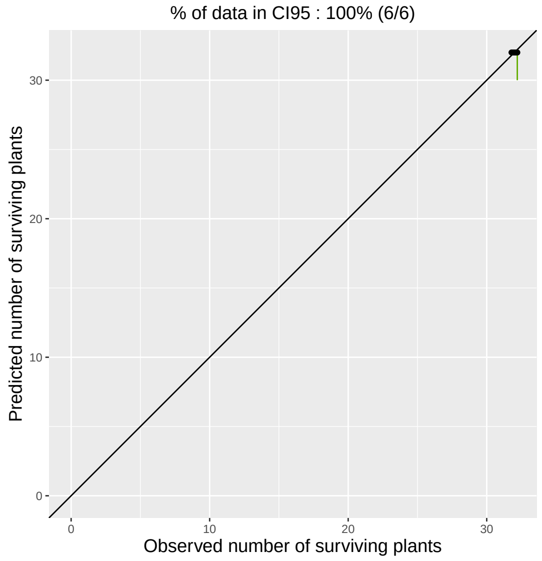

(b) Posterior predictive check (PPC)

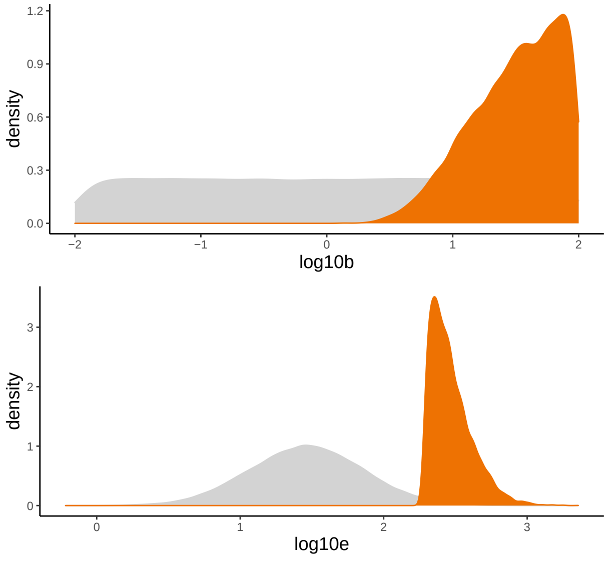

(c) Priors and posteriors

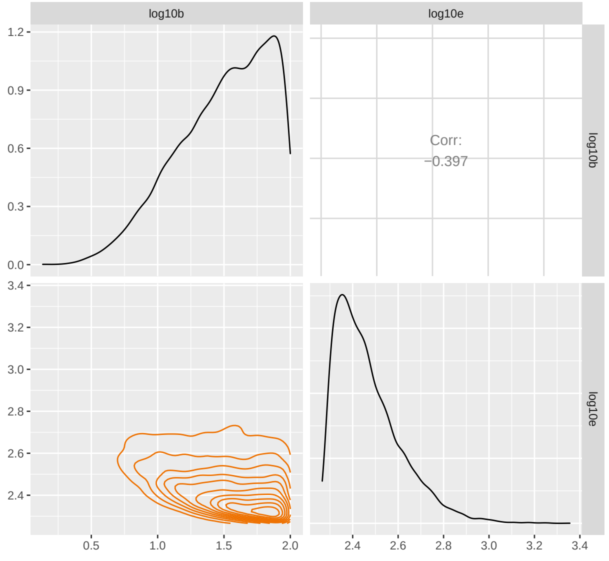

(d) Correlations between parameters

Figure 5: Dose-response curve (a), PPC (b), prior and posterior distributions (c) and correlations between parameters (d).

Data set: GLXMA_VV_survival

Table 6: Summary of parameter estimates (parameter d is set to 1) for GLXMA_VV_survival data set

Parameter	median	Q2.5	Q97.5
b	34.929	5.344	95.055
e	271.871	195.023	678.067

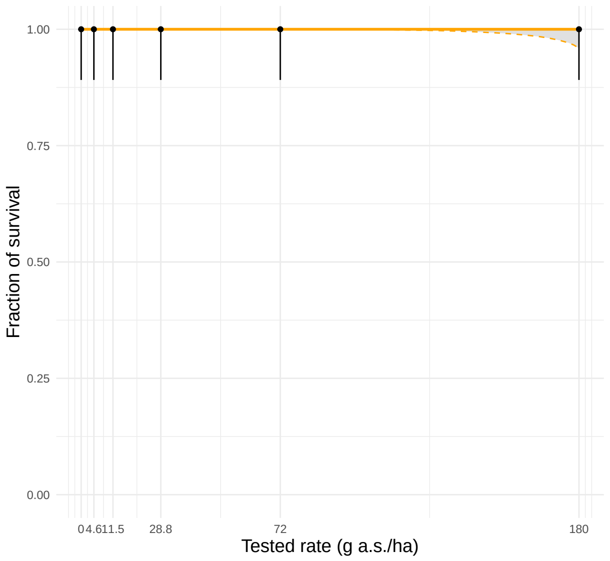

(a) Dose-response curve

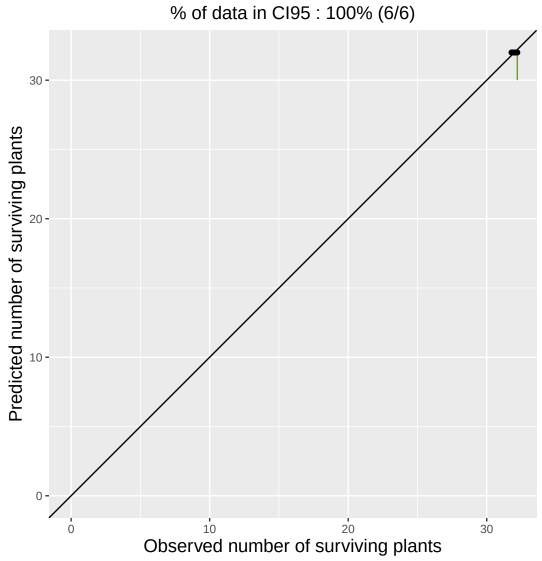

(b) Posterior predictive check (PPC)

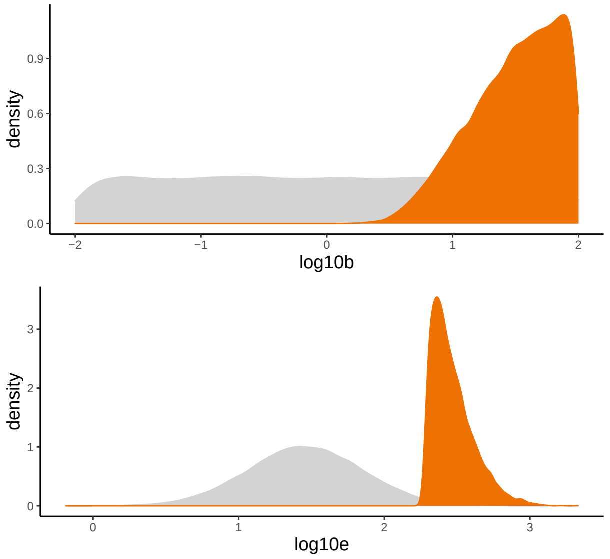

(c) Priors and posteriors

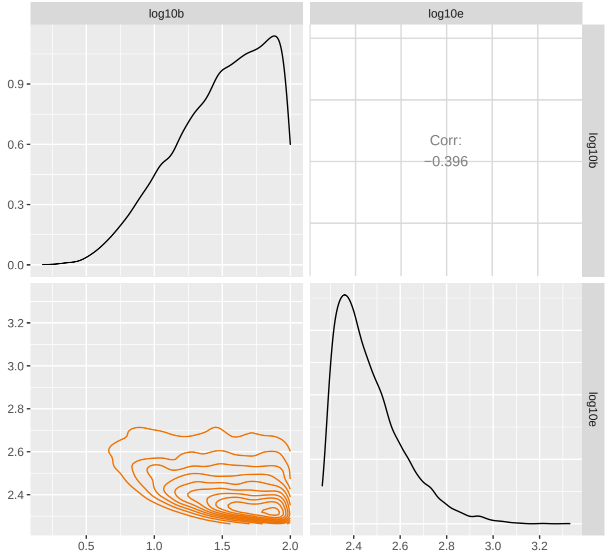

(d) Correlations between parameters

Figure 6: Dose-response curve (a), PPC (b), prior and posterior distributions (c) and correlations between parameters (d).

Data set: HELAN_VV_survival

Table 7: Summary of parameter estimates (parameter d is set to 1) for HELAN_VV_survival data set

Parameter	median	Q2.5	Q97.5
b	1.746	1.088	2.886
e	771.668	393.115	2033.204

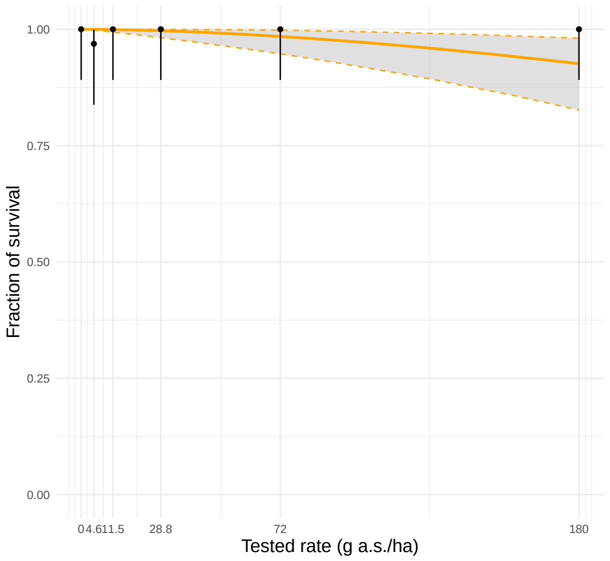

(a) Dose-response curve

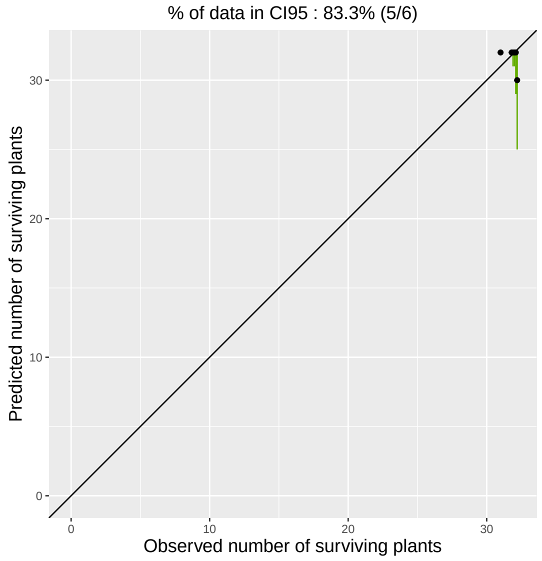

(b) Posterior predictive check (PPC)

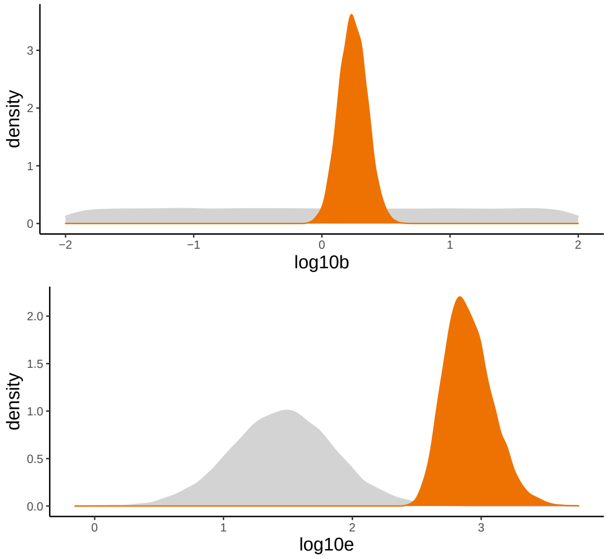

(c) Priors and posteriors

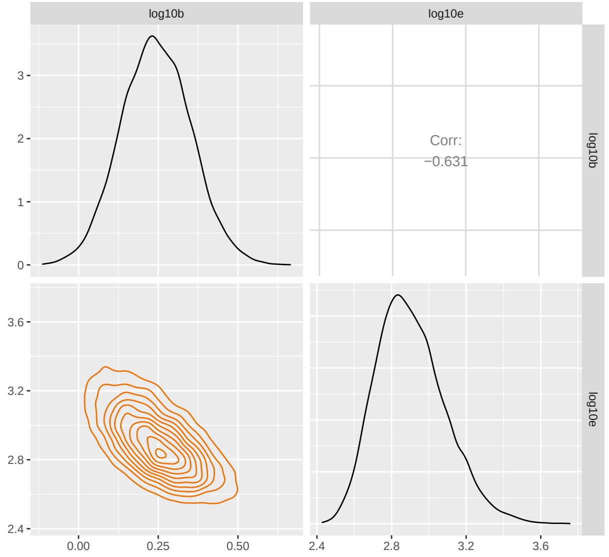

(d) Correlations between parameters

Figure 7: Dose-response curve (a), PPC (b), prior and posterior distributions (c) and correlations between parameters (d).

Data set: LOLPE_VV_survival

Table 8: Summary of parameter estimates (parameter d is set to 1) for LOLPE_VV_survival data set

Parameter	median	Q2.5	Q97.5
b	34.540	5.346	94.954
e	271.511	195.454	664.165

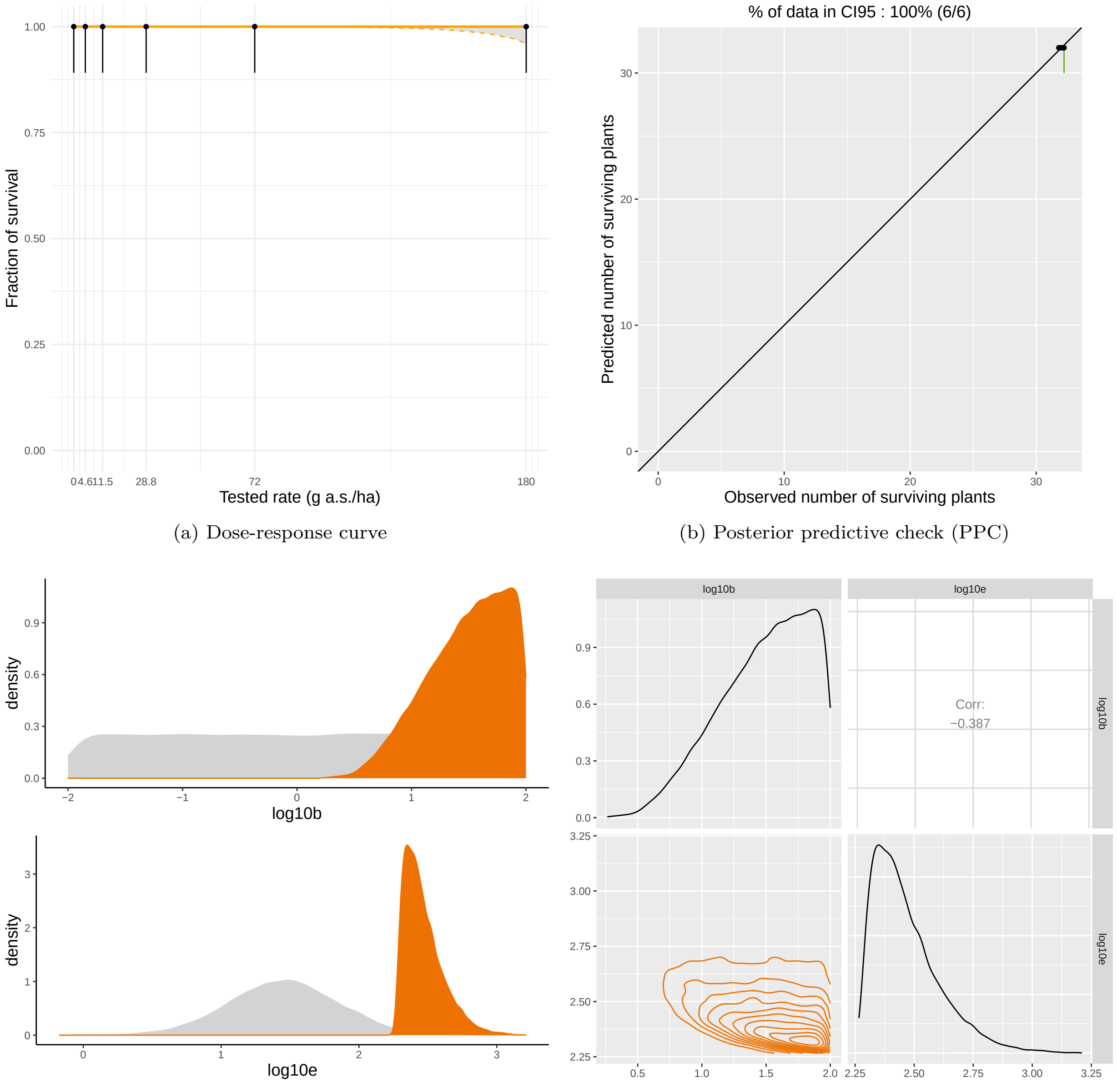

(a) Dose-response curve

(b) Posterior predictive check (PPC)

(c) Priors and posteriors

(d) Correlations between parameters

Figure 8: Dose-response curve (a), PPC (b), prior and posterior distributions (c) and correlations between parameters (d).

Data set: LYPES_VV_survival

Table 9: Summary of parameter estimates (parameter d is set to 1) for LYPES_VV_survival data set

Parameter	median	Q2.5	Q97.5
b	35.129	5.084	95.399
e	273.151	195.041	671.923

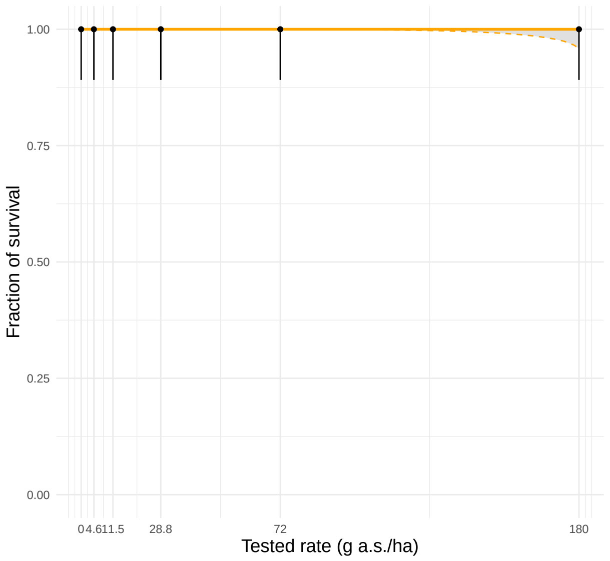

(a) Dose-response curve

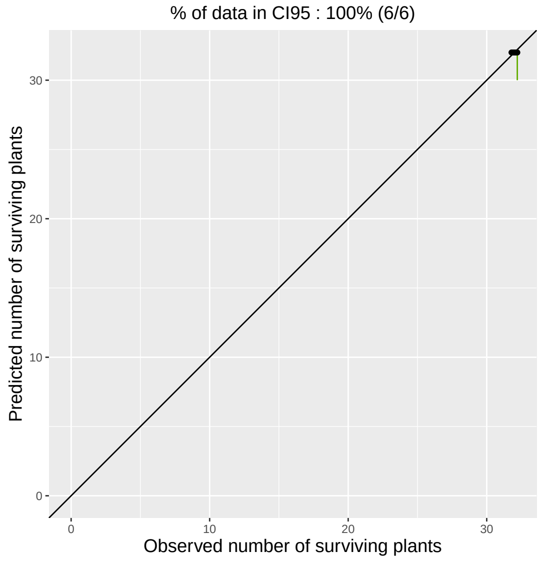

(b) Posterior predictive check (PPC)

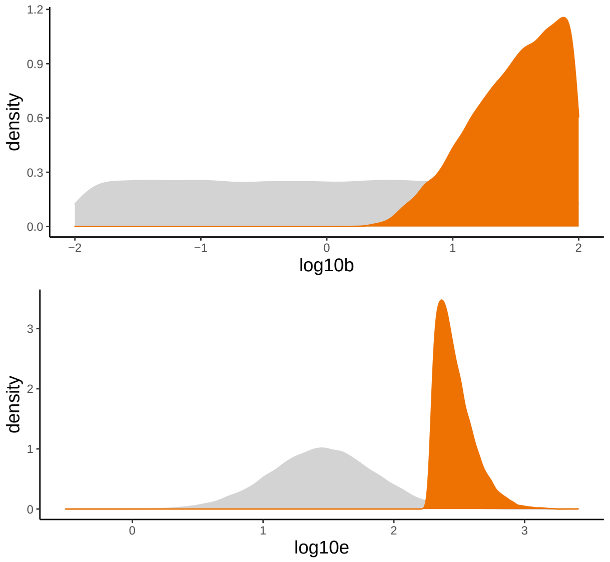

(c) Priors and posteriors

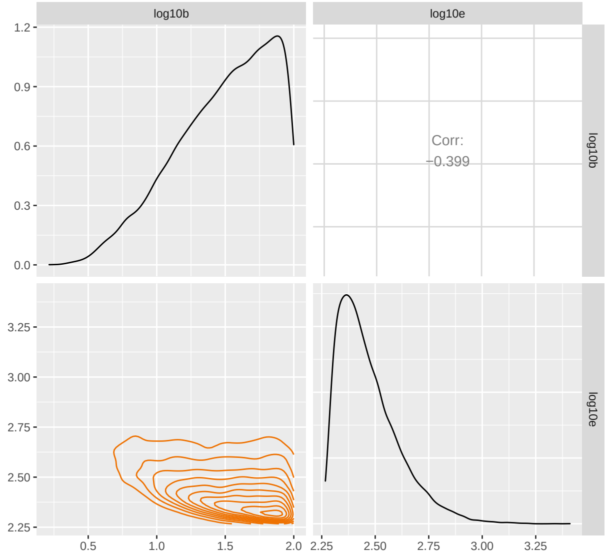

(d) Correlations between parameters

Figure 9: Dose-response curve (a), PPC (b), prior and posterior distributions (c) and correlations between parameters (d).

Data set: ZEAMA_VV_survival

Table 10: Summary of parameter estimates (parameter d is set to 1) for ZEAMA_VV_survival data set

Parameter	median	Q2.5	Q97.5
b	34.766	5.198	95.195
e	272.189	194.775	668.061

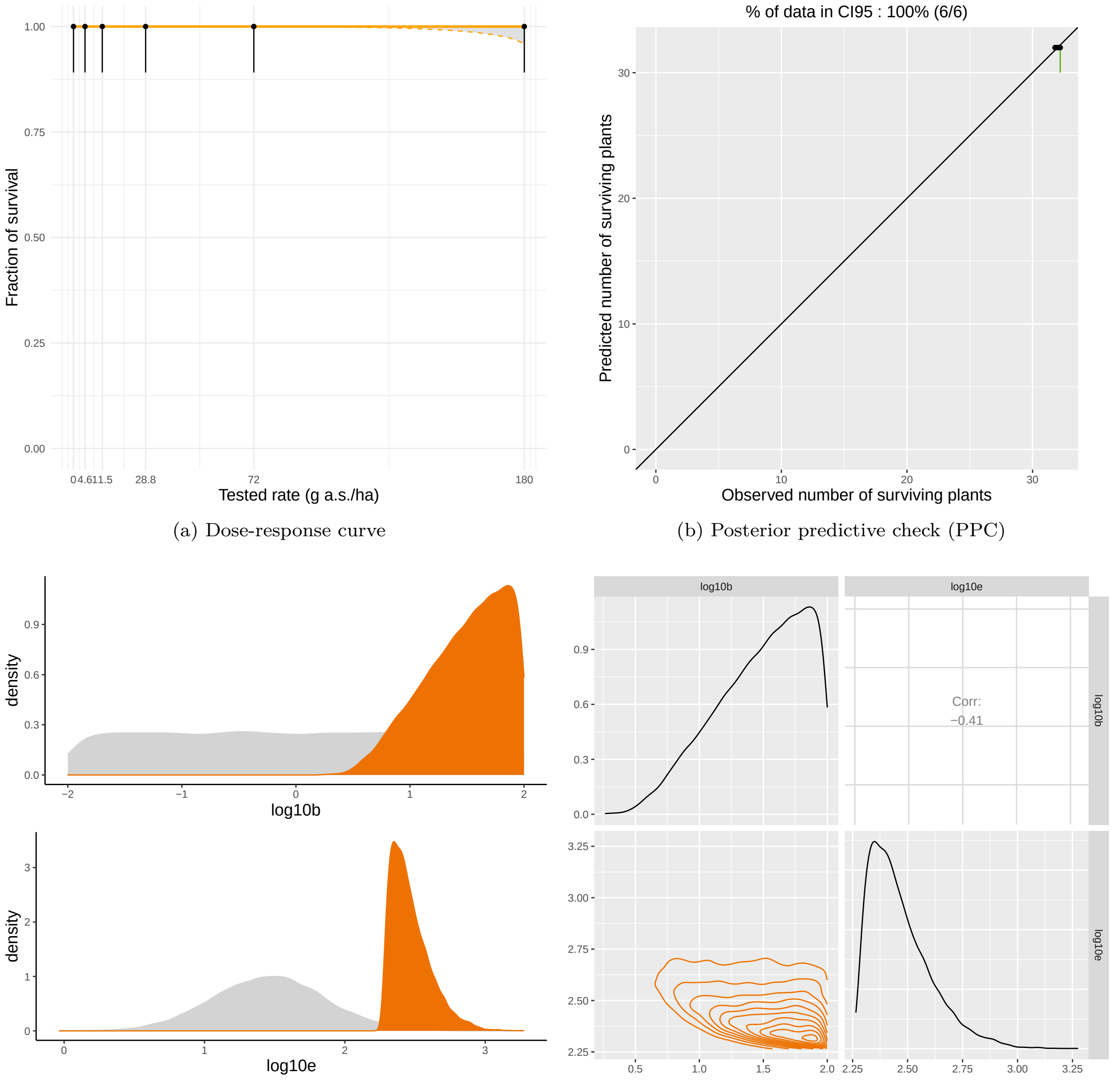

(a) Dose-response curve

(b) Posterior predictive check (PPC)

(c) Priors and posteriors

(d) Correlations between parameters

Figure 10: Dose-response curve (a), PPC (b), prior and posterior distributions (c) and correlations between parameters (d).
