# Supplementary material for: How to account for the uncertainty from standard toxicity tests in species sensitivity distributions: An example in non-target plants
Source: PLoS One. 2021 Jan 7;16(1):e0245071. doi: 10.1371/journal.pone.0245071 (PMC7790375; doi:10.1371/journal.pone.0245071)
Supplement: S1 Archive — It is a zip file containing seven folders (one folder per case study). Each folder contains five files report_xxx.pdf with detailed results of the dose-response analyses, one file corresponding to does-response analysis per endpoint. It also contains one file ER50_censoring.pdf for censored ER50 and one file SSD_analyses.pdf for results of SSD analyses. (ZIP) [file pone.0245071.s004.zip › S1_archive/Study2/report_VV_weight.pdf]

Dose-response analysis

Study 2

Vegetative Vigour test - shoot dry VV_weight endpoint

25 June 2020

Contact: sandrine.charles@univ-lyon1.fr

This is a report which provides results on all performed dose-response analyses for the shoot dry VV_weight endpoint of the Vegetative Vigour test for study 2.

Contents

Data set: ALLCE_VV_weight 2
Data set: AVESA_VV_weight 3
Data set: BEAVA_VV_weight 4
Data set: BRSNW_VV_weight 5
Data set: CUMSA_VV_weight 6
Data set: GLXMA_VV_weight 7
Data set: HELAN_VV_weight 8
Data set: LOLPE_VV_weight 9
Data set: LYPES_VV_weight 10
Data set: ZEAMA_VV_weight 11

Data set: ALLCE_VV_weight

Table 1: Summary of parameter estimates for ALLCE_VV_weight data set

Parameter	median	Q2.5	Q97.5
b	27.377	3.206	94.450
d	0.069	0.064	0.073
e	267.731	191.643	702.042
sigma	0.015	0.012	0.019

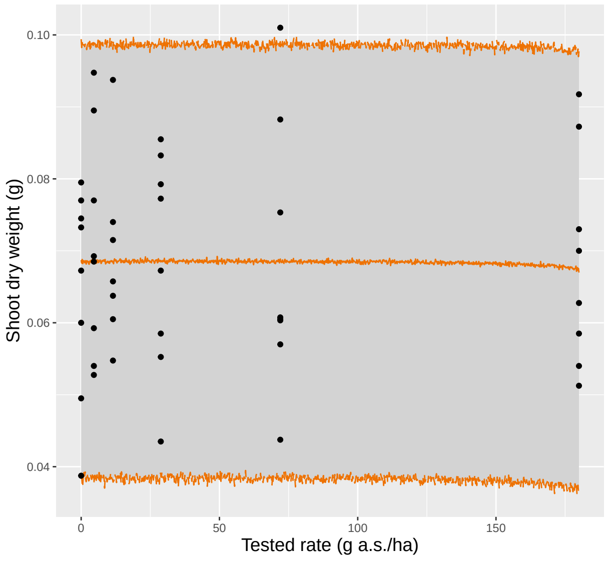

(a) Dose-response curve

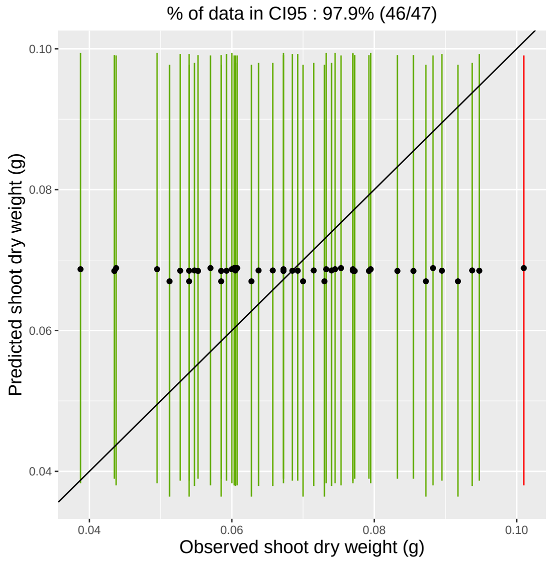

(b) Posterior predictive check (PPC)

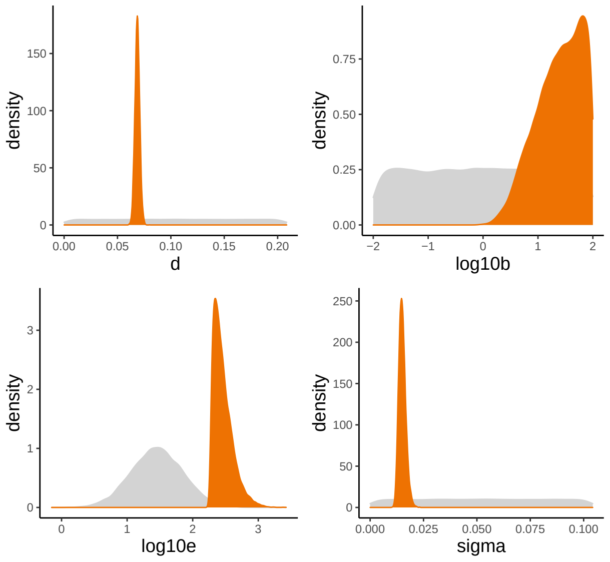

(c) Priors and posteriors

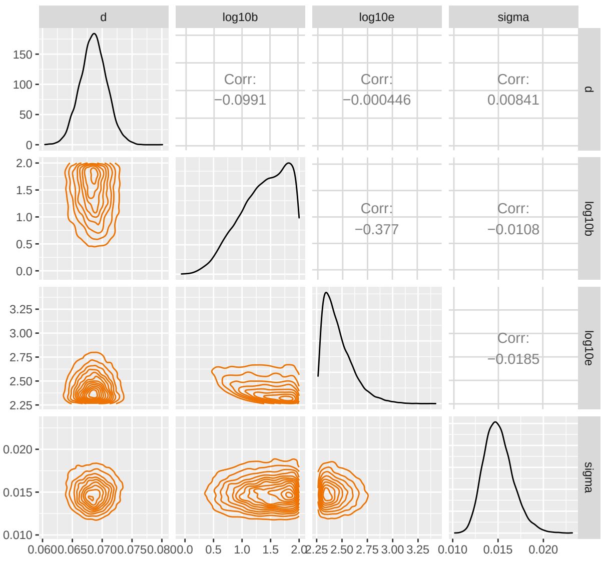

(d) Correlations between parameters

Figure 1: Dose-response curve (a), PPC (b), prior and posterior distributions (c) and correlations between parameters (d).

Data set: AVESA_VV_weight

Table 2: Summary of parameter estimates for AVESA_VV_weight data set

Parameter	median	Q2.5	Q97.5
b	36.267	5.761	95.815
d	2.781	2.685	2.881
e	275.835	195.680	719.176
sigma	0.329	0.271	0.413

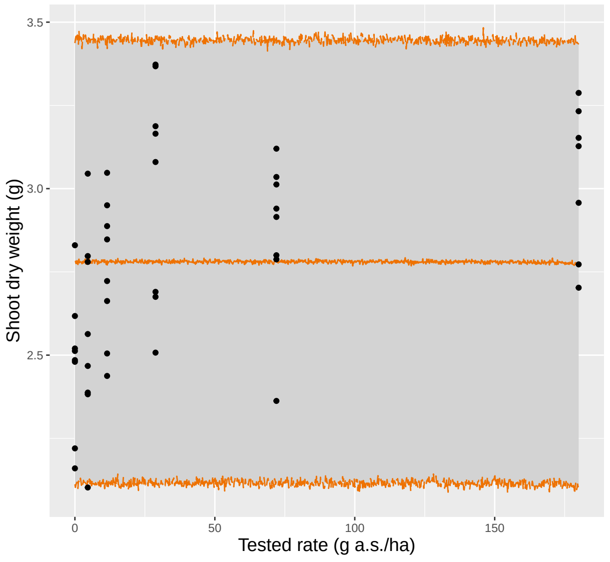

(a) Dose-response curve

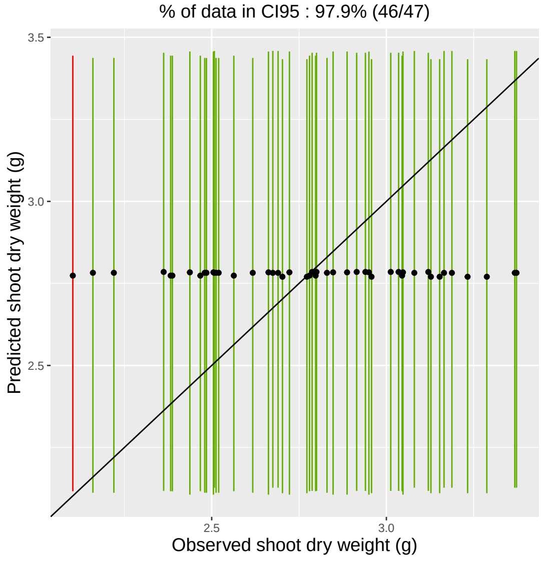

(b) Posterior predictive check (PPC)

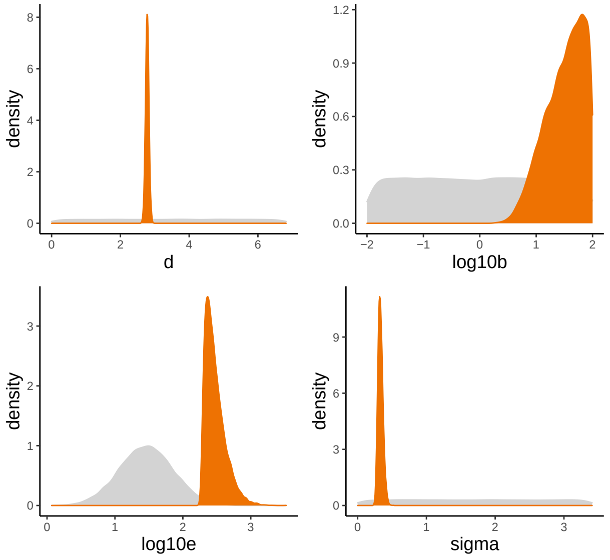

(c) Priors and posteriors

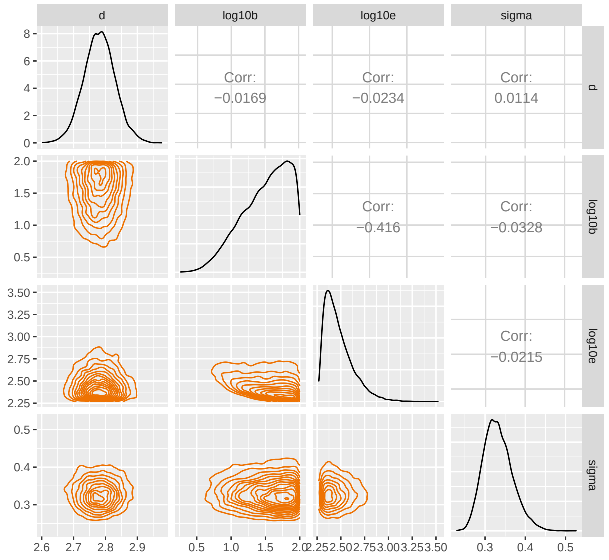

(d) Correlations between parameters

Figure 2: Dose-response curve (a), PPC (b), prior and posterior distributions (c) and correlations between parameters (d).

Data set: BEAVA_VV_weight

Table 3: Summary of parameter estimates for BEAVA_VV_weight data set

Parameter	median	Q2.5	Q97.5
b	4.309	1.092	54.930
d	4.464	4.361	4.600
e	260.558	185.483	711.614
sigma	0.433	0.377	0.506

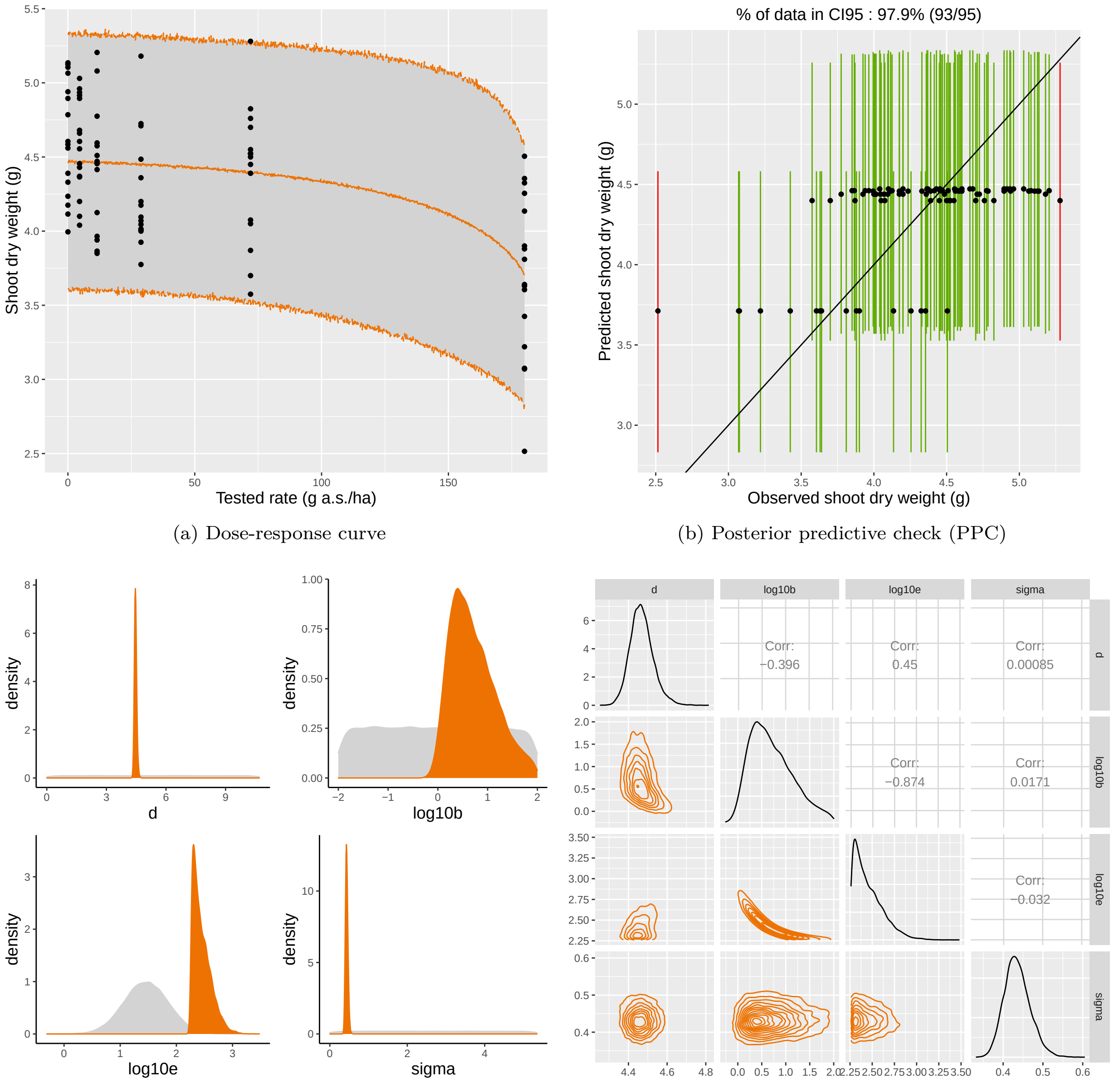

(a) Dose-response curve

(b) Posterior predictive check (PPC)

(c) Priors and posteriors

(d) Correlations between parameters

Figure 3: Dose-response curve (a), PPC (b), prior and posterior distributions (c) and correlations between parameters (d).

Data set: BRSNW_VV_weight

Table 4: Summary of parameter estimates for BRSNW_VV_weight data set

Parameter	median	Q2.5	Q97.5
b	0.828	0.566	1.256
d	6.786	6.440	7.187
e	373.208	257.356	643.918
sigma	0.906	0.787	1.056

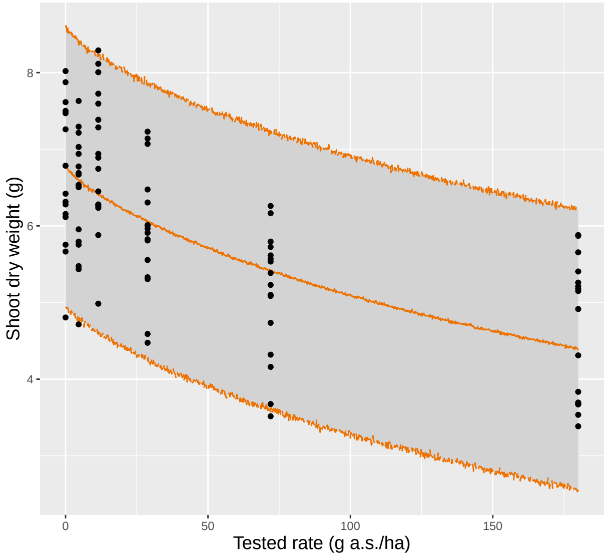

(a) Dose-response curve

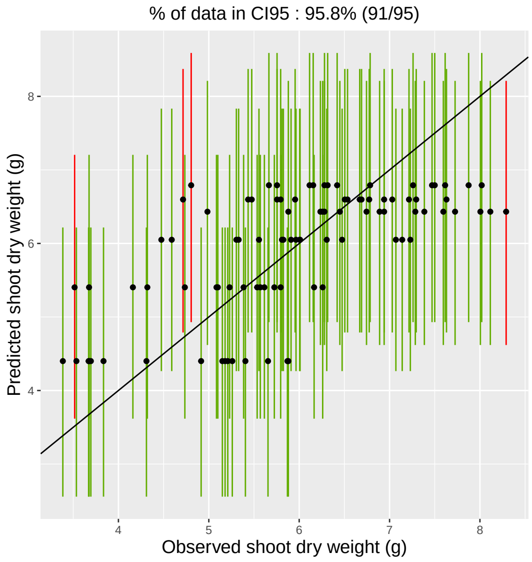

(b) Posterior predictive check (PPC)

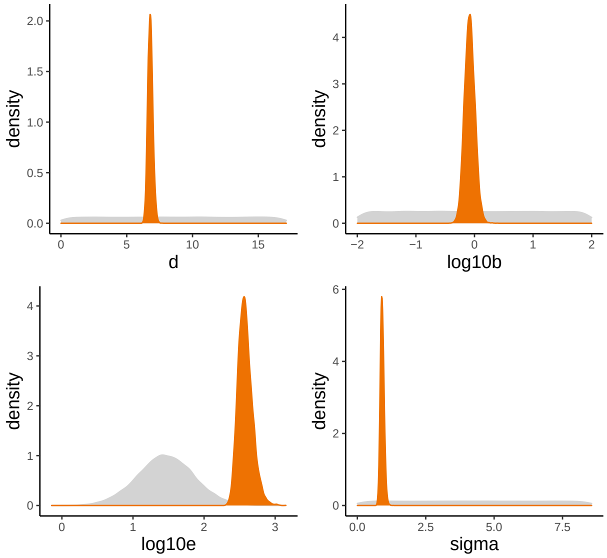

(c) Priors and posteriors

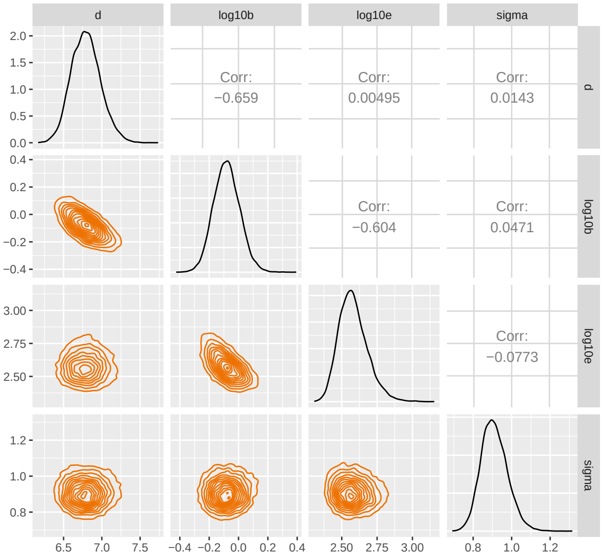

(d) Correlations between parameters

Figure 4: Dose-response curve (a), PPC (b), prior and posterior distributions (c) and correlations between parameters (d).

Data set: CUMSA_VV_weight

Table 5: Summary of parameter estimates for CUMSA_VV_weight data set

Parameter	median	Q2.5	Q97.5
b	0.509	0.380	0.686
d	14.056	13.292	14.866
e	422.594	267.973	776.848
sigma	1.609	1.398	1.879

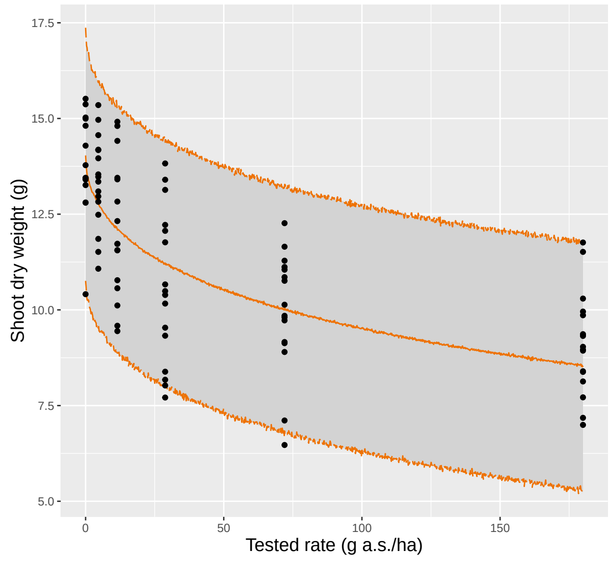

(a) Dose-response curve

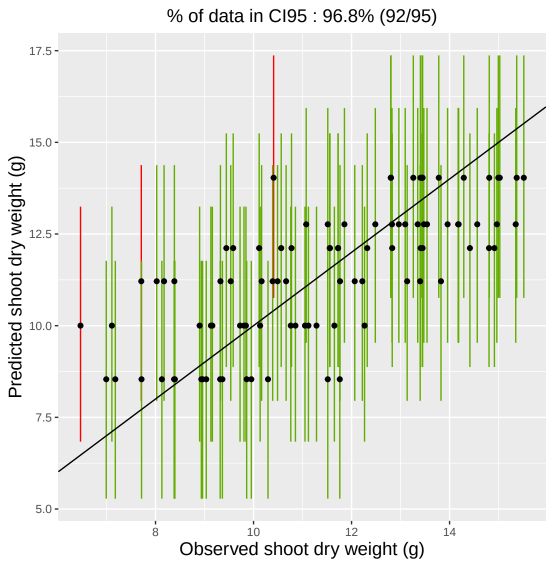

(b) Posterior predictive check (PPC)

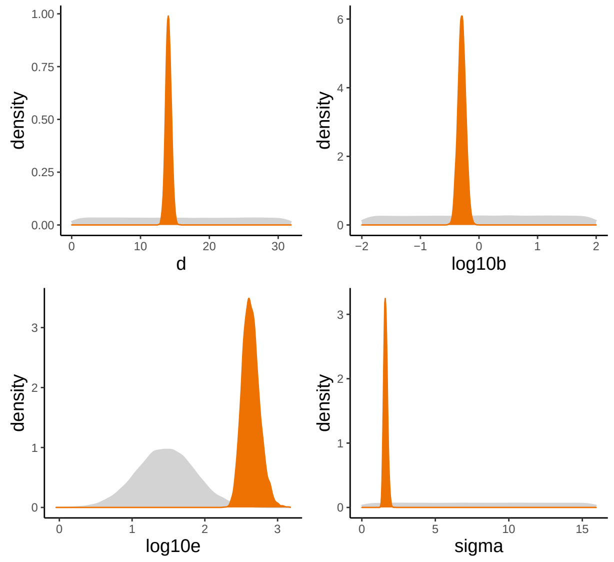

(c) Priors and posteriors

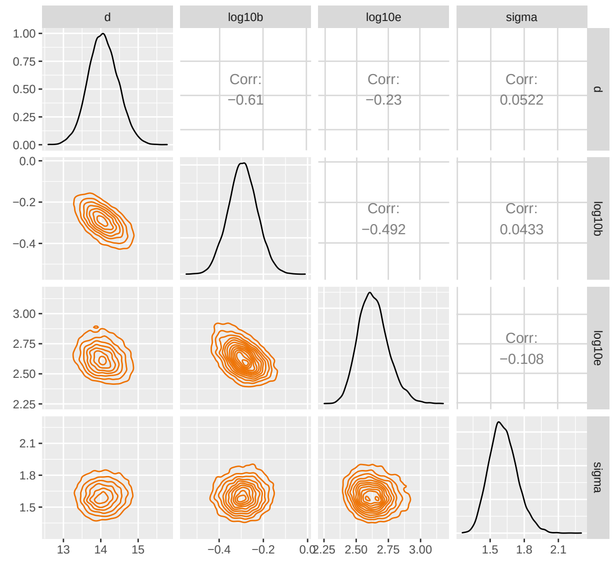

(d) Correlations between parameters

Figure 5: Dose-response curve (a), PPC (b), prior and posterior distributions (c) and correlations between parameters (d).

Data set: GLXMA_VV_weight

Table 6: Summary of parameter estimates for GLXMA_VV_weight data set

Parameter	median	Q2.5	Q97.5
b	31.159	3.593	94.501
d	5.780	5.645	5.923
e	270.189	193.516	720.077
sigma	0.658	0.576	0.765

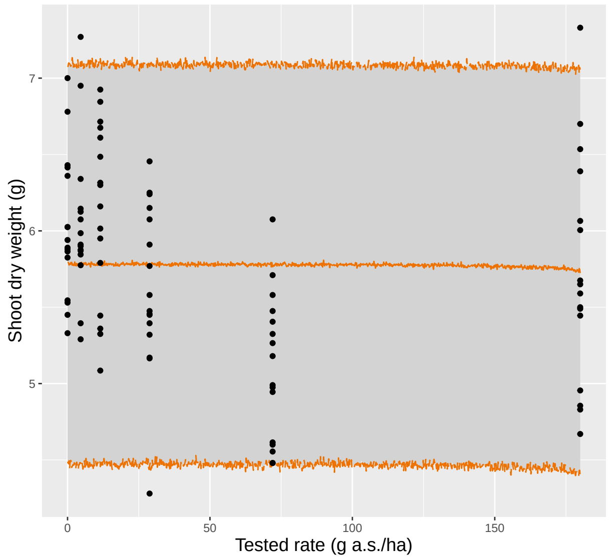

(a) Dose-response curve

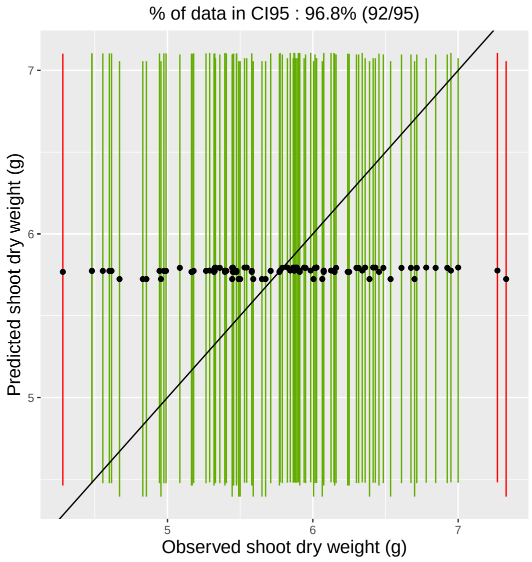

(b) Posterior predictive check (PPC)

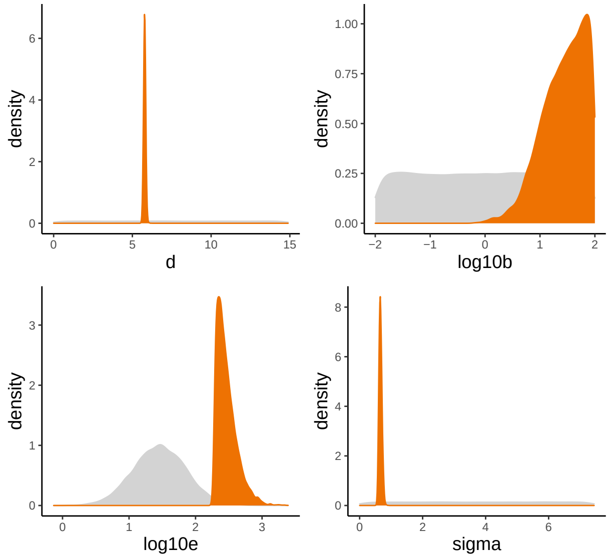

(c) Priors and posteriors

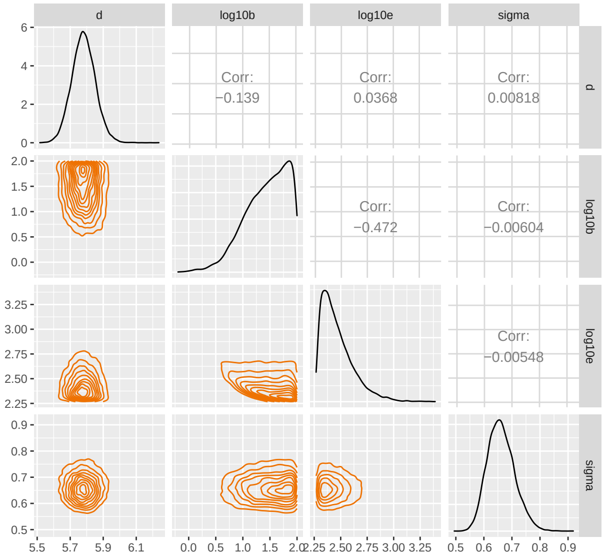

(d) Correlations between parameters

Figure 6: Dose-response curve (a), PPC (b), prior and posterior distributions (c) and correlations between parameters (d).

Data set: HELAN_VV_weight

Table 7: Summary of parameter estimates for HELAN_VV_weight data set

Parameter	median	Q2.5	Q97.5
b	21.936	1.979	92.641
d	2.759	2.690	2.837
e	265.261	190.155	816.097
sigma	0.310	0.270	0.361

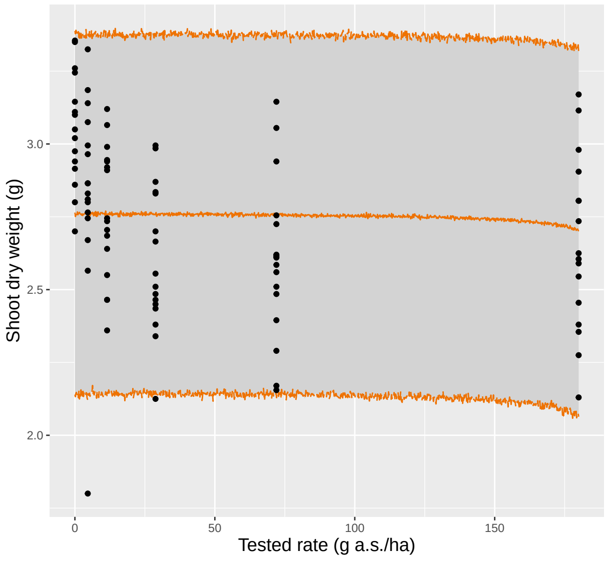

(a) Dose-response curve

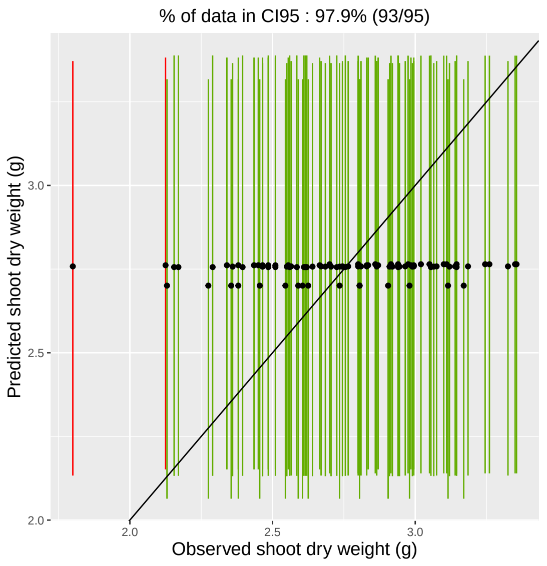

(b) Posterior predictive check (PPC)

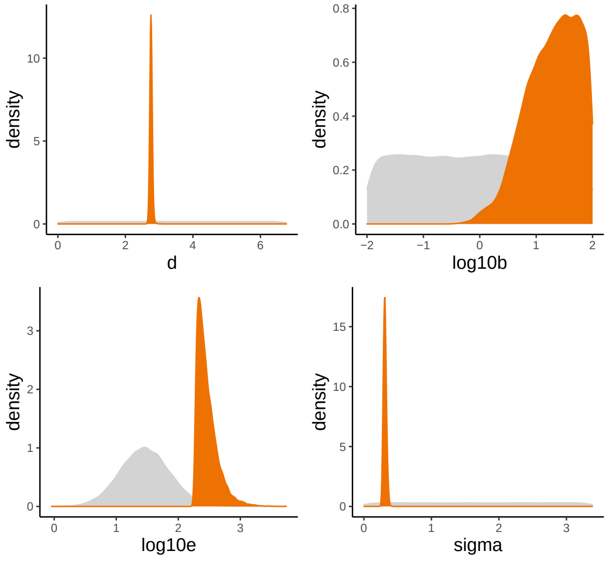

(c) Priors and posteriors

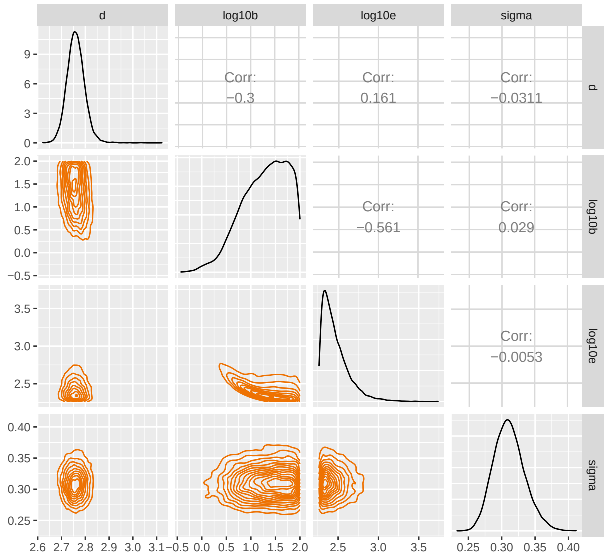

(d) Correlations between parameters

Figure 7: Dose-response curve (a), PPC (b), prior and posterior distributions (c) and correlations between parameters (d).

Data set: LOLPE_VV_weight

Table 8: Summary of parameter estimates for LOLPE_VV_weight data set

Parameter	median	Q2.5	Q97.5
b	30.217	4.185	94.400
d	0.253	0.239	0.267
e	266.256	192.719	665.884
sigma	0.049	0.041	0.062

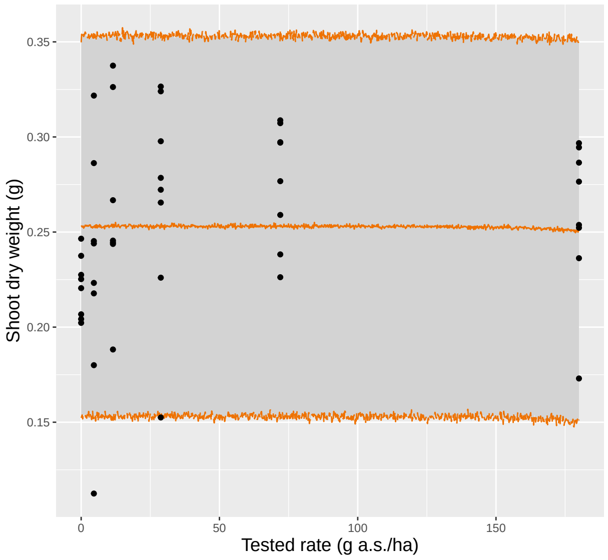

(a) Dose-response curve

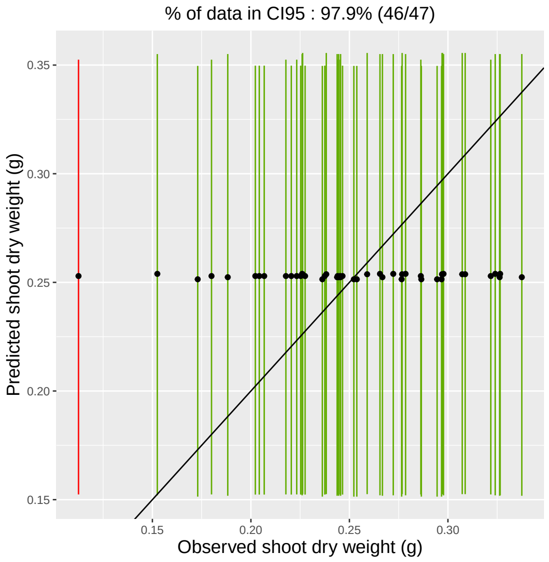

(b) Posterior predictive check (PPC)

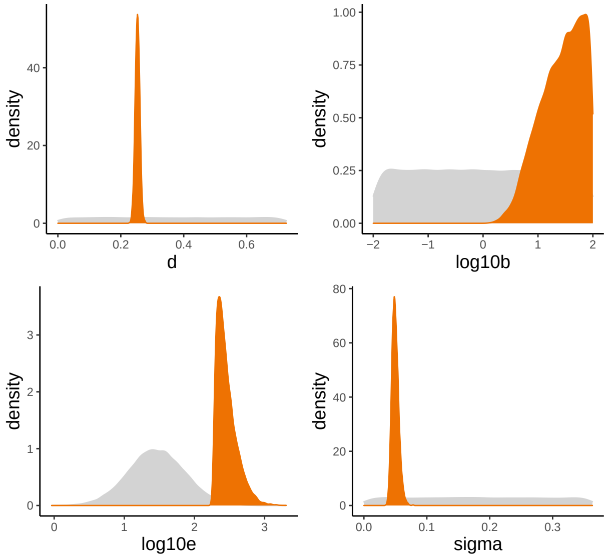

(c) Priors and posteriors

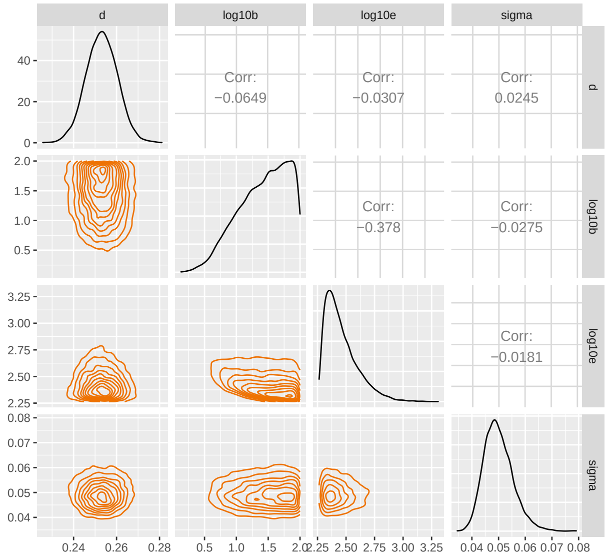

(d) Correlations between parameters

Figure 8: Dose-response curve (a), PPC (b), prior and posterior distributions (c) and correlations between parameters (d).

Data set: LYPES_VV_weight

Table 9: Summary of parameter estimates for LYPES_VV_weight data set

Parameter	median	Q2.5	Q97.5
b	0.812	0.651	1.020
d	6.851	6.553	7.163
e	176.247	141.137	229.857
sigma	0.732	0.639	0.852

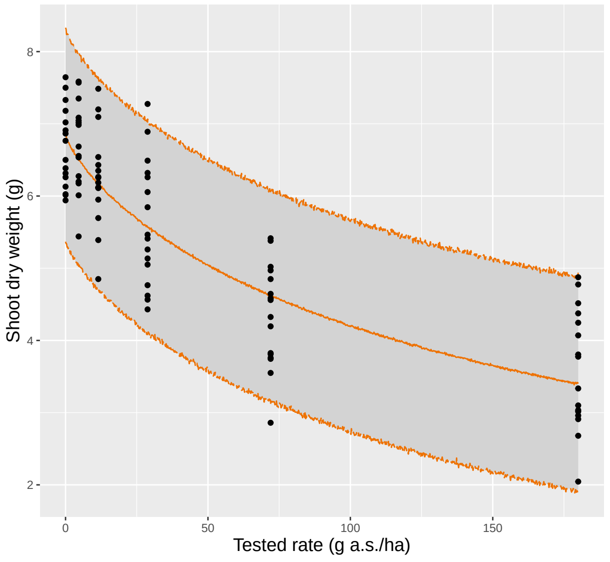

(a) Dose-response curve

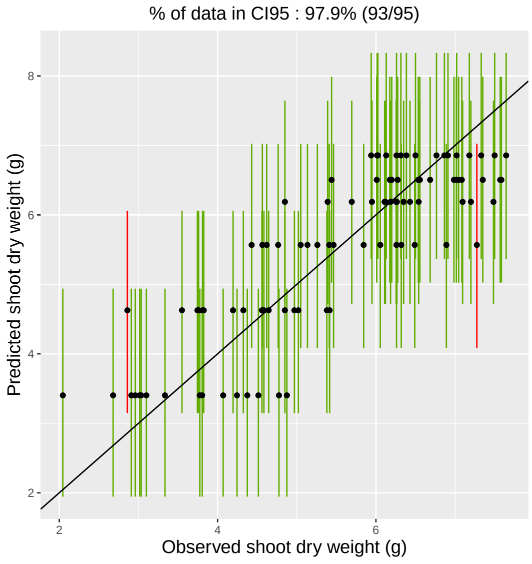

(b) Posterior predictive check (PPC)

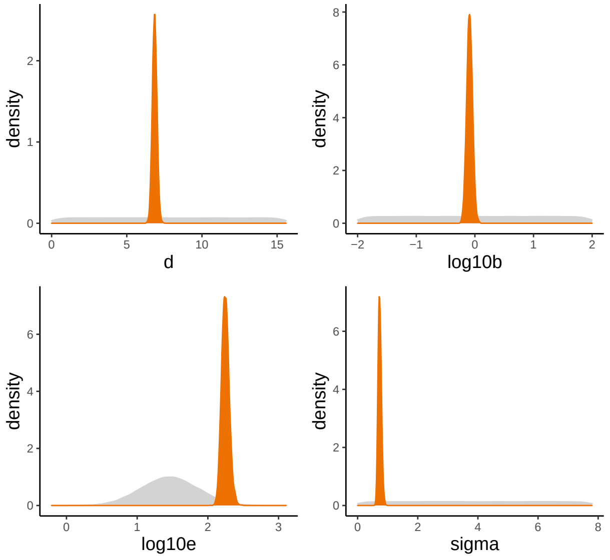

(c) Priors and posteriors

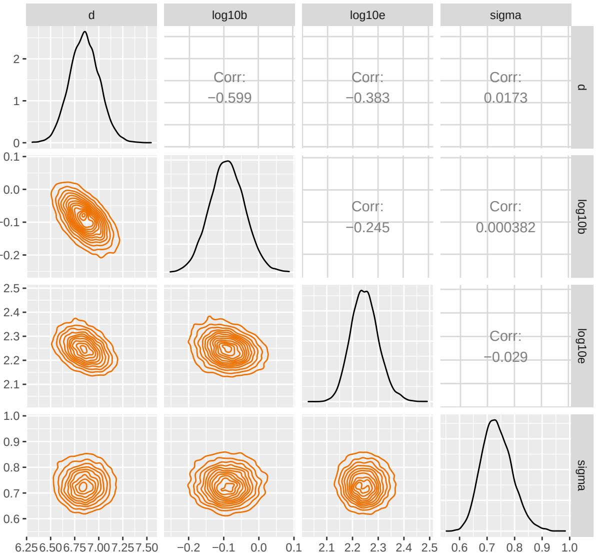

(d) Correlations between parameters

Figure 9: Dose-response curve (a), PPC (b), prior and posterior distributions (c) and correlations between parameters (d).

Data set: ZEAMA_VV_weight

Table 10: Summary of parameter estimates for ZEAMA_VV_weight data set

Parameter	median	Q2.5	Q97.5
b	22.991	1.864	92.923
d	7.636	7.365	7.947
e	264.931	190.651	729.949
sigma	1.298	1.131	1.510

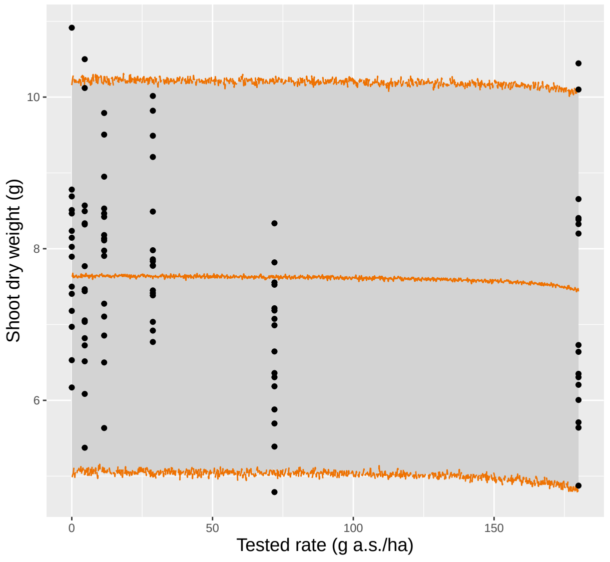

(a) Dose-response curve

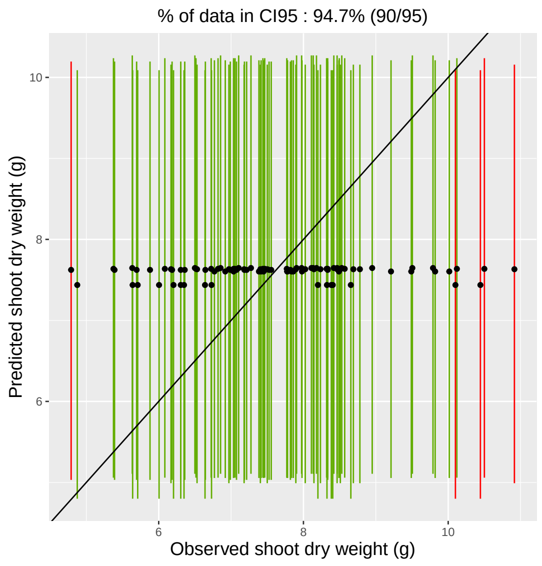

(b) Posterior predictive check (PPC)

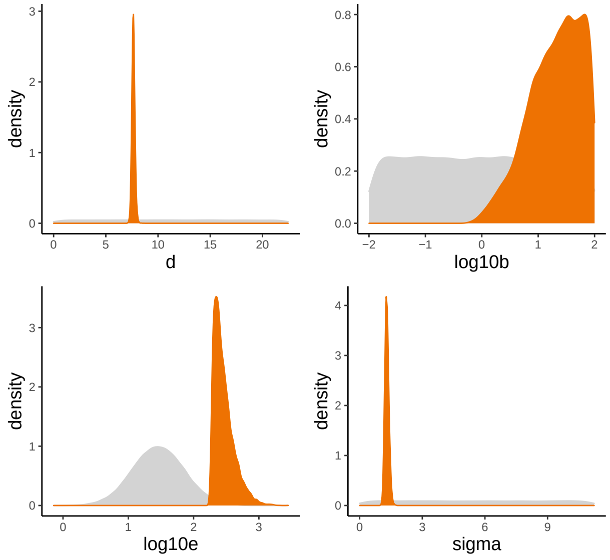

(c) Priors and posteriors

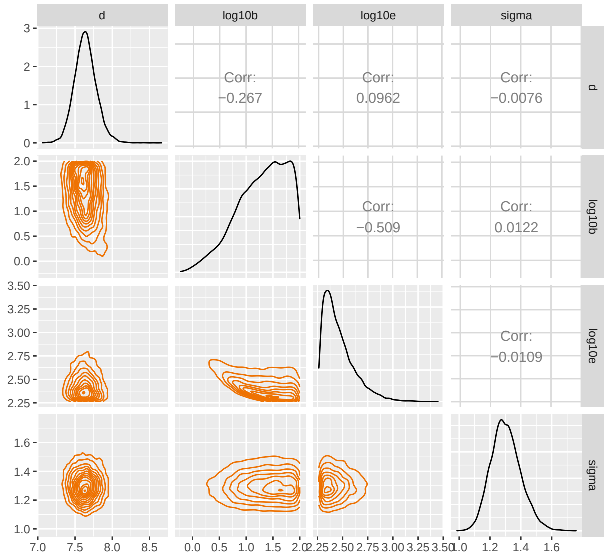

(d) Correlations between parameters

Figure 10: Dose-response curve (a), PPC (b), prior and posterior distributions (c) and correlations between parameters (d).
