# Supplementary material for: How to account for the uncertainty from standard toxicity tests in species sensitivity distributions: An example in non-target plants
Source: PLoS One. 2021 Jan 7;16(1):e0245071. doi: 10.1371/journal.pone.0245071 (PMC7790375; doi:10.1371/journal.pone.0245071)
Supplement: S1 Archive — It is a zip file containing seven folders (one folder per case study). Each folder contains five files report_xxx.pdf with detailed results of the dose-response analyses, one file corresponding to does-response analysis per endpoint. It also contains one file ER50_censoring.pdf for censored ER50 and one file SSD_analyses.pdf for results of SSD analyses. (ZIP) [file pone.0245071.s004.zip › S1_archive/Study3/ER50_censoring.pdf]

ER_{50} censoring after dose-response analyses

Study 3

25 June 2020

Contact: sandrine.charles@univ-lyon1.fr

This document provides the ER_{50} censoring after dose-response analyses for study 3, including four parts:

- Raw data description (test item rates);
- Summary on ER_{50} from a Bayesian dose-response fit;
- Censoring on ER_{50} according to two criteria;
  - Criterion 1 (C_1) based on the ratio of the probablities, that is the ratio of the probability that the ER_{50} lies within the range of the LCI95 and the maximum tested rate over the probability that the ER_{50} lies in the CI95;
  - Criterion 2 (C_2) based on the ratio of distances, that is the ratio of the distance between the LCI95 of ER_{50} and the maximum tested rate over the extend of the CI95 of ER_{50};
- Graphical presentation of censored ER_{50} per species per endpoint.

Meaning of symbols used in the document

- min_rate: minimum tested rate;
- max_rate: maximum tested rate;
- ER_{50}: 50% effective rate;
- CI95: 95% credible interval;
- ER_{50}_median: median of ER_{50};
- ER_{50}_LCI95: lower bound of the CI95 of ER_{50};
- ER_{50}_UCI95: upper bound of the CI95 of ER_{50};
- C_1: criterion based on the ratio of probabilities;
- C_2: criterion based on the ratio of distances;
- T: decision threshold;
- Censored ER_{50} [lower bound; upper bound]:
  - C1T0.0: censored ER_{50} according to C_1 with T = 0.0;
  - C1T0.2: censored ER_{50} according to C_1 with T = 0.2;

- C1T0.4: censored ER_{50} according to C_1 with T = 0.4;
- C1T0.5: censored ER_{50} according to C_1 with T = 0.5;
- C1T0.6: censored ER_{50} according to C_1 with T = 0.6;
- C1T0.8: censored ER_{50} according to C_1 with T = 0.8;
- C1T1.0: censored ER_{50} according to C_1 with T = 1.0;
- C2T0.0: censored ER_{50} according to C_2 with T = 0.0;
- C2T0.2: censored ER_{50} according to C_2 with T = 0.2;
- C2T0.4: censored ER_{50} according to C_2 with T = 0.4;
- C2T0.5: censored ER_{50} according to C_2 with T = 0.5;
- C2T0.6: censored ER_{50} according to C_2 with T = 0.6;
- C2T0.8: censored ER_{50} according to C_2 with T = 0.8;
- C2T1.0: censored ER_{50} according to C_2 with T = 1.0.

Seeding emergence (SE) test of study 3

Raw data description (test item rates) for the SE test

```
Number of species:  10
EPPO code:  ALLCE BEAVA BRSNW CUMSA FAGES GLXMA LOLPE LYPES TRZAW ZEAMA
```

Table 1: Correspondance between species and common names according to the EPPO code

EPPO code	Species name	Common name
ALLCE	*Allium cepa*	Onion
BEAVA	*Beta vulgaris*	Sugar beet
BRSNW	*Brassica napus winter*	Oilseed rape
CUMSA	*Cucumis sativus*	Cucumber
FAGES	*Fagopyrum esculentum*	Buckwheat
GLXMA	*Glycine max*	Soybean
LOLPE	*Lolium perenne*	Perennial ryegrass
LYPES	*Solanum lycopersicum*	Tomato
TRZAW	*Triticum aestivum winter*	Winter wheat
ZEAMA	*Zea mays subs. Amylacea*	Corn

Table 2: Tested rates per species (ml product/ha) for the SE test of study 3

ALLCE	BEAVA	BRSNW	CUMSA	FAGES	GLXMA	LOLPE	LYPES	TRZAW	ZEAMA
0.0	0.0	0.0	0.0	0.0	0.0	0.0	0.0	0.0	0.0
30.0	30.0	30.0	30.0	30.0	30.0	30.0	30.0	30.0	30.0
79.8	79.8	79.8	79.8	79.8	79.8	79.8	79.8	79.8	79.8
212.0	212.0	212.0	212.0	212.0	212.0	212.0	212.0	212.0	212.0
564.0	564.0	564.0	564.0	564.0	564.0	564.0	564.0	564.0	564.0
1500.0	1500.0	1500.0	1500.0	1500.0	1500.0	1500.0	1500.0	1500.0	1500.0

SE test - emergence endpoint

Table 3: Minimum and maximum tested rates, median, quantile 2.5% and quantile 97.5% of the ER_{50} posterior distribution - emergence endpoint of the SE test of study 3

species	min_rate	max_rate	ER_{50}_median	ER_{50}_LCI95	ER_{50}_UCI95
ALLCE	30	1500	1742.724	1386.898	4285.237
BEAVA	30	1500	2314.562	1618.871	6004.500
BRSNW	30	1500	2218.620	1583.885	5925.315
CUMSA	30	1500	2289.370	1605.898	6129.611
FAGES	30	1500	2269.224	1600.636	6043.838
GLXMA	30	1500	5292.275	2475.910	15515.520
LOLPE	30	1500	2250.291	1589.317	5903.808
LYPES	30	1500	2229.507	1585.341	5793.903
TRZAW	30	1500	2257.608	1595.494	6090.512
ZEAMA	30	1500	4389.401	2291.687	11578.225

Table 4: Censored ER_{50} according to criterion 1 and the seven decision thresholds - emergence endpoint of the SE test of study 3

species	C1T0.0	C1T0.2	C1T0.4	C1T0.5	C1T0.6	C1T0.8	C1T1.0
ALLCE	[1386.9; 4285.24]	[1386.9; Inf)	[1386.9; Inf)	[1386.9; Inf)	[1386.9; Inf)	[1386.9; Inf)	[1386.9; Inf)
BEAVA	[1500; Inf)	[1500; Inf)	[1500; Inf)	[1500; Inf)	[1500; Inf)	[1500; Inf)	[1500; Inf)
BRSNW	[1500; Inf)	[1500; Inf)	[1500; Inf)	[1500; Inf)	[1500; Inf)	[1500; Inf)	[1500; Inf)
CUMSA	[1500; Inf)	[1500; Inf)	[1500; Inf)	[1500; Inf)	[1500; Inf)	[1500; Inf)	[1500; Inf)
FAGES	[1500; Inf)	[1500; Inf)	[1500; Inf)	[1500; Inf)	[1500; Inf)	[1500; Inf)	[1500; Inf)
GLXMA	[1500; Inf)	[1500; Inf)	[1500; Inf)	[1500; Inf)	[1500; Inf)	[1500; Inf)	[1500; Inf)
LOLPE	[1500; Inf)	[1500; Inf)	[1500; Inf)	[1500; Inf)	[1500; Inf)	[1500; Inf)	[1500; Inf)
LYPES	[1500; Inf)	[1500; Inf)	[1500; Inf)	[1500; Inf)	[1500; Inf)	[1500; Inf)	[1500; Inf)
TRZAW	[1500; Inf)	[1500; Inf)	[1500; Inf)	[1500; Inf)	[1500; Inf)	[1500; Inf)	[1500; Inf)
ZEAMA	[1500; Inf)	[1500; Inf)	[1500; Inf)	[1500; Inf)	[1500; Inf)	[1500; Inf)	[1500; Inf)

Table 5: Censored ER_{50} according to criterion 2 and the seven decision thresholds - emergence endpoint of the SE test of study 3

species	C2T0.0	C2T0.2	C2T0.4	C2T0.5	C2T0.6	C2T0.8	C2T1.0
ALLCE	[1386.9; 4285.24]	[1386.9; Inf)	[1386.9; Inf)	[1386.9; Inf)	[1386.9; Inf)	[1386.9; Inf)	[1386.9; Inf)
BEAVA	[1500; Inf)	[1500; Inf)	[1500; Inf)	[1500; Inf)	[1500; Inf)	[1500; Inf)	[1500; Inf)
BRSNW	[1500; Inf)	[1500; Inf)	[1500; Inf)	[1500; Inf)	[1500; Inf)	[1500; Inf)	[1500; Inf)
CUMSA	[1500; Inf)	[1500; Inf)	[1500; Inf)	[1500; Inf)	[1500; Inf)	[1500; Inf)	[1500; Inf)
FAGES	[1500; Inf)	[1500; Inf)	[1500; Inf)	[1500; Inf)	[1500; Inf)	[1500; Inf)	[1500; Inf)
GLXMA	[1500; Inf)	[1500; Inf)	[1500; Inf)	[1500; Inf)	[1500; Inf)	[1500; Inf)	[1500; Inf)
LOLPE	[1500; Inf)	[1500; Inf)	[1500; Inf)	[1500; Inf)	[1500; Inf)	[1500; Inf)	[1500; Inf)
LYPES	[1500; Inf)	[1500; Inf)	[1500; Inf)	[1500; Inf)	[1500; Inf)	[1500; Inf)	[1500; Inf)
TRZAW	[1500; Inf)	[1500; Inf)	[1500; Inf)	[1500; Inf)	[1500; Inf)	[1500; Inf)	[1500; Inf)
ZEAMA	[1500; Inf)	[1500; Inf)	[1500; Inf)	[1500; Inf)	[1500; Inf)	[1500; Inf)	[1500; Inf)

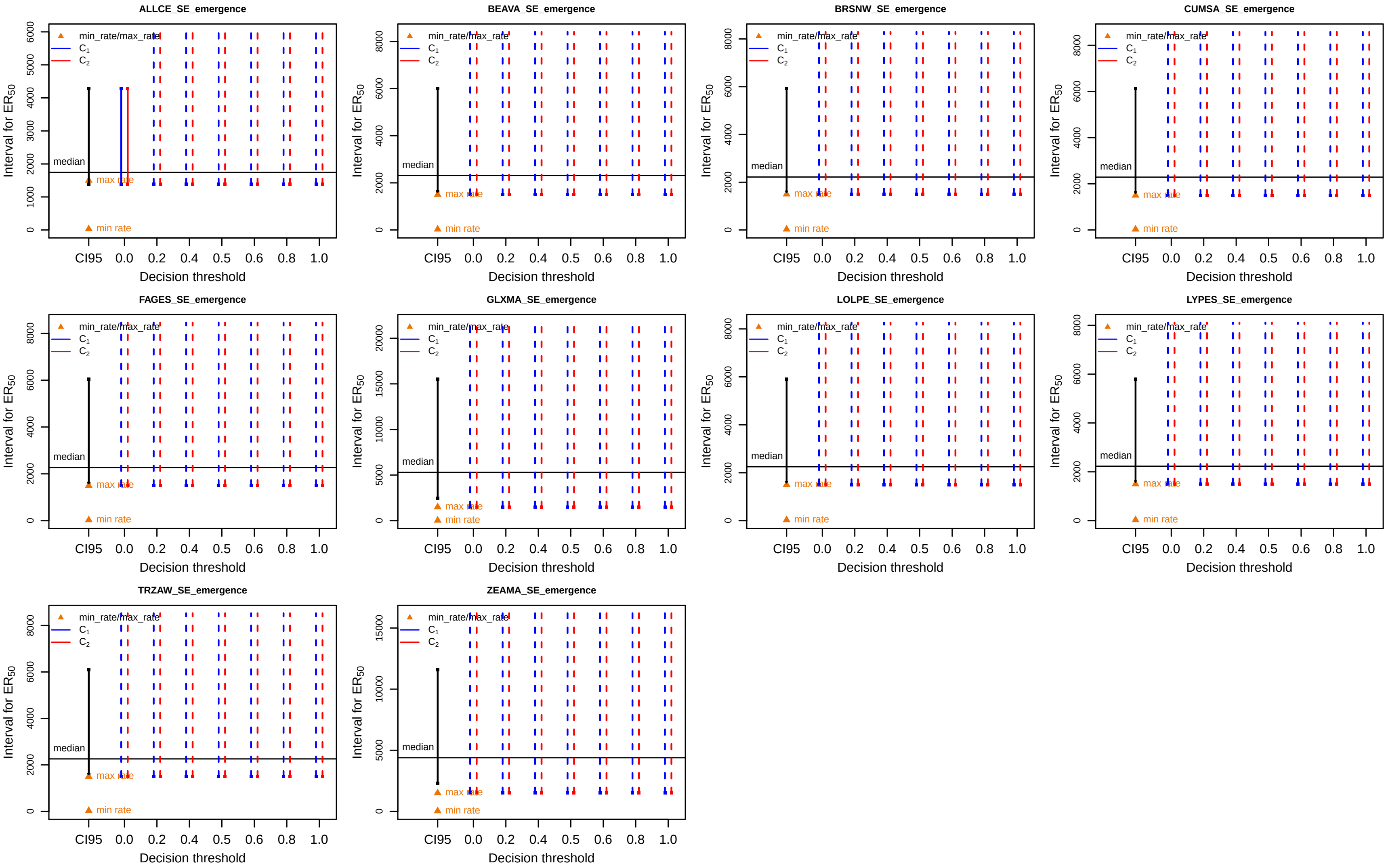

Figure 1: CI95 of ER_{50} and censored ER_{50} according to both criteria and the seven decision thresholds for all species - emergence endpoint of the SE test of study 3. The two orange triangles stand for the lowest and the highest tested rates. Vertical segments of three different colors (black, blue, red) represent the CI95 of ER_{50}, the censored ER_{50} according to C_1 and the censored ER_{50} according to C_2, respectively; solid vertical segments are for bounded intervals while dotted vertical segments stand for right-unbounded intervals; the black horizontal line represents the median of the ER_{50}.

SE test - survival endpoint

Table 6: Minimum and maximum tested rates, median, quantile 2.5% and quantile 97.5% of the ER_{50} posterior distribution - survival endpoint of the SE test of study 3

species	min_rate	max_rate	ER_{50}_median	ER_{50}_LCI95	ER_{50}_UCI95
ALLCE	30	1500	2272.1158	1603.5634	6155.935
BEAVA	30	1500	2311.8684	1620.4007	5999.744
BRSNW	30	1500	1377.0157	1073.6710	1557.188
CUMSA	30	1500	1024.0139	796.9740	1279.489
FAGES	30	1500	1870.3143	1532.0435	3884.872
GLXMA	30	1500	2318.1280	1618.9881	6272.430
LOLPE	30	1500	2461.8786	1537.6805	5801.728
LYPES	30	1500	1857.5730	1530.8646	3901.110
TRZAW	30	1500	869.5895	675.9331	1143.524
ZEAMA	30	1500	2303.7013	1622.0544	5941.655

Table 7: Censored ER_{50} according to criterion 1 and the seven decision thresholds - survival endpoint of the SE test of study 3

species	C1T0.0	C1T0.2	C1T0.4	C1T0.5	C1T0.6	C1T0.8	C1T1.0
ALLCE	[1500; Inf)	[1500; Inf)	[1500; Inf)	[1500; Inf)	[1500; Inf)	[1500; Inf)	[1500; Inf)
BEAVA	[1500; Inf)	[1500; Inf)	[1500; Inf)	[1500; Inf)	[1500; Inf)	[1500; Inf)	[1500; Inf)
BRSNW	[1073.67; 1557.19]	[1073.67; 1557.19]	[1073.67; 1557.19]	[1073.67; 1557.19]	[1073.67; 1557.19]	[1073.67; 1557.19]	[1073.67; Inf)
CUMSA	[796.97; 1279.49]	[796.97; 1279.49]	[796.97; 1279.49]	[796.97; 1279.49]	[796.97; 1279.49]	[796.97; 1279.49]	[796.97; 1279.49]
FAGES	[1500; Inf)	[1500; Inf)	[1500; Inf)	[1500; Inf)	[1500; Inf)	[1500; Inf)	[1500; Inf)
GLXMA	[1500; Inf)	[1500; Inf)	[1500; Inf)	[1500; Inf)	[1500; Inf)	[1500; Inf)	[1500; Inf)
LOLPE	[1500; Inf)	[1500; Inf)	[1500; Inf)	[1500; Inf)	[1500; Inf)	[1500; Inf)	[1500; Inf)
LYPES	[1500; Inf)	[1500; Inf)	[1500; Inf)	[1500; Inf)	[1500; Inf)	[1500; Inf)	[1500; Inf)
TRZAW	[675.93; 1143.52]	[675.93; 1143.52]	[675.93; 1143.52]	[675.93; 1143.52]	[675.93; 1143.52]	[675.93; 1143.52]	[675.93; 1143.52]
ZEAMA	[1500; Inf)	[1500; Inf)	[1500; Inf)	[1500; Inf)	[1500; Inf)	[1500; Inf)	[1500; Inf)

Table 8: Censored ER_{50} according to criterion 2 and the seven decision thresholds - survival endpoint of the SE test of study 3

species	C2T0.0	C2T0.2	C2T0.4	C2T0.5	C2T0.6	C2T0.8	C2T1.0
ALLCE	[1500; Inf)	[1500; Inf)	[1500; Inf)	[1500; Inf)	[1500; Inf)	[1500; Inf)	[1500; Inf)
BEAVA	[1500; Inf)	[1500; Inf)	[1500; Inf)	[1500; Inf)	[1500; Inf)	[1500; Inf)	[1500; Inf)
BRSNW	[1073.67; 1557.19]	[1073.67; 1557.19]	[1073.67; 1557.19]	[1073.67; 1557.19]	[1073.67; 1557.19]	[1073.67; 1557.19]	[1073.67; Inf)
CUMSA	[796.97; 1279.49]	[796.97; 1279.49]	[796.97; 1279.49]	[796.97; 1279.49]	[796.97; 1279.49]	[796.97; 1279.49]	[796.97; 1279.49]
FAGES	[1500; Inf)	[1500; Inf)	[1500; Inf)	[1500; Inf)	[1500; Inf)	[1500; Inf)	[1500; Inf)
GLXMA	[1500; Inf)	[1500; Inf)	[1500; Inf)	[1500; Inf)	[1500; Inf)	[1500; Inf)	[1500; Inf)
LOLPE	[1500; Inf)	[1500; Inf)	[1500; Inf)	[1500; Inf)	[1500; Inf)	[1500; Inf)	[1500; Inf)
LYPES	[1500; Inf)	[1500; Inf)	[1500; Inf)	[1500; Inf)	[1500; Inf)	[1500; Inf)	[1500; Inf)
TRZAW	[675.93; 1143.52]	[675.93; 1143.52]	[675.93; 1143.52]	[675.93; 1143.52]	[675.93; 1143.52]	[675.93; 1143.52]	[675.93; 1143.52]
ZEAMA	[1500; Inf)	[1500; Inf)	[1500; Inf)	[1500; Inf)	[1500; Inf)	[1500; Inf)	[1500; Inf)

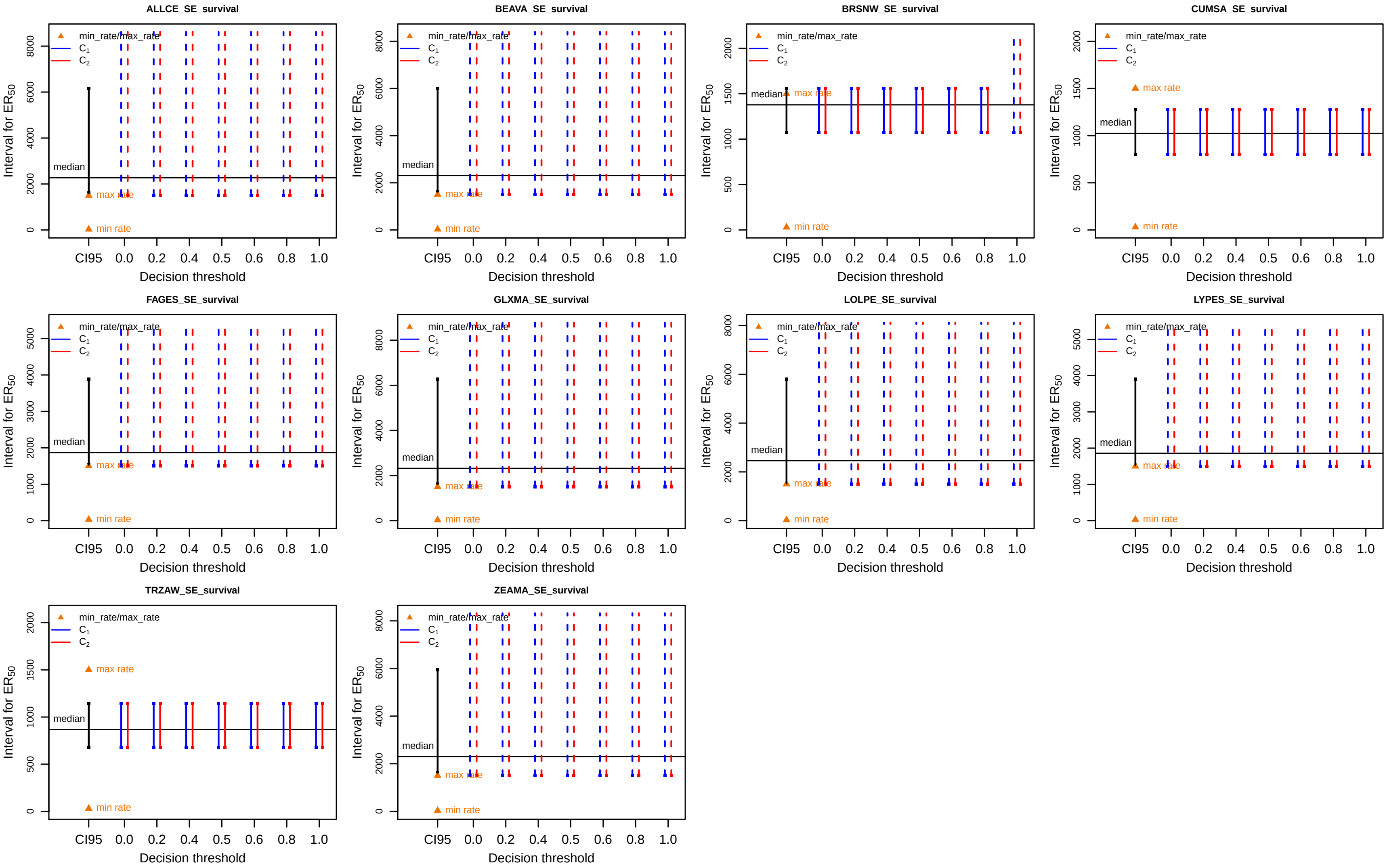

Figure 2: CI95 of ER_{50} and censored ER_{50} according to both criteria and the seven decision thresholds for all species - survival endpoint of the SE test of study 3. The two orange triangles stand for the lowest and the highest tested rates. Vertical segments of three different colors (black, blue, red) represent the CI95 of ER_{50}, the censored ER_{50} according to C_1 and the censored ER_{50} according to C_2, respectively; solid vertical segments are for bounded intervals while dotted vertical segments stand for right-unbounded intervals; the black horizontal line represents the median of the ER_{50}.

SE test - shoot dry weight endpoint

Table 9: Minimum and maximum tested rates, median, quantile 2.5% and quantile 97.5% of the ER_{50} posterior distribution - shoot dry weight endpoint of the SE test of study 3

species	min_rate	max_rate	ER_{50}_median	ER_{50}_LCI95	ER_{50}_UCI95
ALLCE	30	1500	1996.7535	1532.3159	4942.2224
BEAVA	30	1500	2034.0804	1544.7514	4912.1098
BRSNW	30	1500	839.9267	606.8249	1093.0591
CUMSA	30	1500	284.8633	238.8869	337.3320
FAGES	30	1500	1248.4139	730.3133	2214.8972
GLXMA	30	1500	1742.0393	1523.0436	2651.2352
LOLPE	30	1500	1420.2839	959.0803	1924.4711
LYPES	30	1500	772.6126	583.5074	970.8323
TRZAW	30	1500	213.8782	198.5682	234.7686
ZEAMA	30	1500	2149.6690	1573.5262	5601.5766

Table 10: Censored ER_{50} according to criterion 1 and the seven decision thresholds - shoot dry weight endpoint of the SE test of study 3

species	C1T0.0	C1T0.2	C1T0.4	C1T0.5	C1T0.6	C1T0.8	C1T1.0
ALLCE	[1500; Inf)	[1500; Inf)	[1500; Inf)	[1500; Inf)	[1500; Inf)	[1500; Inf)	[1500; Inf)
BEAVA	[1500; Inf)	[1500; Inf)	[1500; Inf)	[1500; Inf)	[1500; Inf)	[1500; Inf)	[1500; Inf)
BRSNW	[606.82; 1093.06]	[606.82; 1093.06]	[606.82; 1093.06]	[606.82; 1093.06]	[606.82; 1093.06]	[606.82; 1093.06]	[606.82; 1093.06]
CUMSA	[238.89; 337.33]	[238.89; 337.33]	[238.89; 337.33]	[238.89; 337.33]	[238.89; 337.33]	[238.89; 337.33]	[238.89; 337.33]
FAGES	[730.31; 2214.9]	[730.31; 2214.9]	[730.31; 2214.9]	[730.31; 2214.9]	[730.31; 2214.9]	[730.31; Inf)	[730.31; Inf)
GLXMA	[1500; Inf)	[1500; Inf)	[1500; Inf)	[1500; Inf)	[1500; Inf)	[1500; Inf)	[1500; Inf)
LOLPE	[959.08; 1924.47]	[959.08; 1924.47]	[959.08; 1924.47]	[959.08; 1924.47]	[959.08; 1924.47]	[959.08; Inf)	[959.08; Inf)
LYPES	[583.51; 970.83]	[583.51; 970.83]	[583.51; 970.83]	[583.51; 970.83]	[583.51; 970.83]	[583.51; 970.83]	[583.51; 970.83]
TRZAW	[198.57; 234.77]	[198.57; 234.77]	[198.57; 234.77]	[198.57; 234.77]	[198.57; 234.77]	[198.57; 234.77]	[198.57; 234.77]
ZEAMA	[1500; Inf)	[1500; Inf)	[1500; Inf)	[1500; Inf)	[1500; Inf)	[1500; Inf)	[1500; Inf)

Table 11: Censored ER_{50} according to criterion 2 and the seven decision thresholds - shoot dry weight endpoint of the SE test of study 3

species	C2T0.0	C2T0.2	C2T0.4	C2T0.5	C2T0.6	C2T0.8	C2T1.0
ALLCE	[1500; Inf)	[1500; Inf)	[1500; Inf)	[1500; Inf)	[1500; Inf)	[1500; Inf)	[1500; Inf)
BEAVA	[1500; Inf)	[1500; Inf)	[1500; Inf)	[1500; Inf)	[1500; Inf)	[1500; Inf)	[1500; Inf)
BRSNW	[606.82; 1093.06]	[606.82; 1093.06]	[606.82; 1093.06]	[606.82; 1093.06]	[606.82; 1093.06]	[606.82; 1093.06]	[606.82; 1093.06]
CUMSA	[238.89; 337.33]	[238.89; 337.33]	[238.89; 337.33]	[238.89; 337.33]	[238.89; 337.33]	[238.89; 337.33]	[238.89; 337.33]
FAGES	[730.31; 2214.9]	[730.31; 2214.9]	[730.31; 2214.9]	[730.31; 2214.9]	[730.31; Inf)	[730.31; Inf)	[730.31; Inf)
GLXMA	[1500; Inf)	[1500; Inf)	[1500; Inf)	[1500; Inf)	[1500; Inf)	[1500; Inf)	[1500; Inf)
LOLPE	[959.08; 1924.47]	[959.08; 1924.47]	[959.08; 1924.47]	[959.08; 1924.47]	[959.08; Inf)	[959.08; Inf)	[959.08; Inf)
LYPES	[583.51; 970.83]	[583.51; 970.83]	[583.51; 970.83]	[583.51; 970.83]	[583.51; 970.83]	[583.51; 970.83]	[583.51; 970.83]
TRZAW	[198.57; 234.77]	[198.57; 234.77]	[198.57; 234.77]	[198.57; 234.77]	[198.57; 234.77]	[198.57; 234.77]	[198.57; 234.77]
ZEAMA	[1500; Inf)	[1500; Inf)	[1500; Inf)	[1500; Inf)	[1500; Inf)	[1500; Inf)	[1500; Inf)

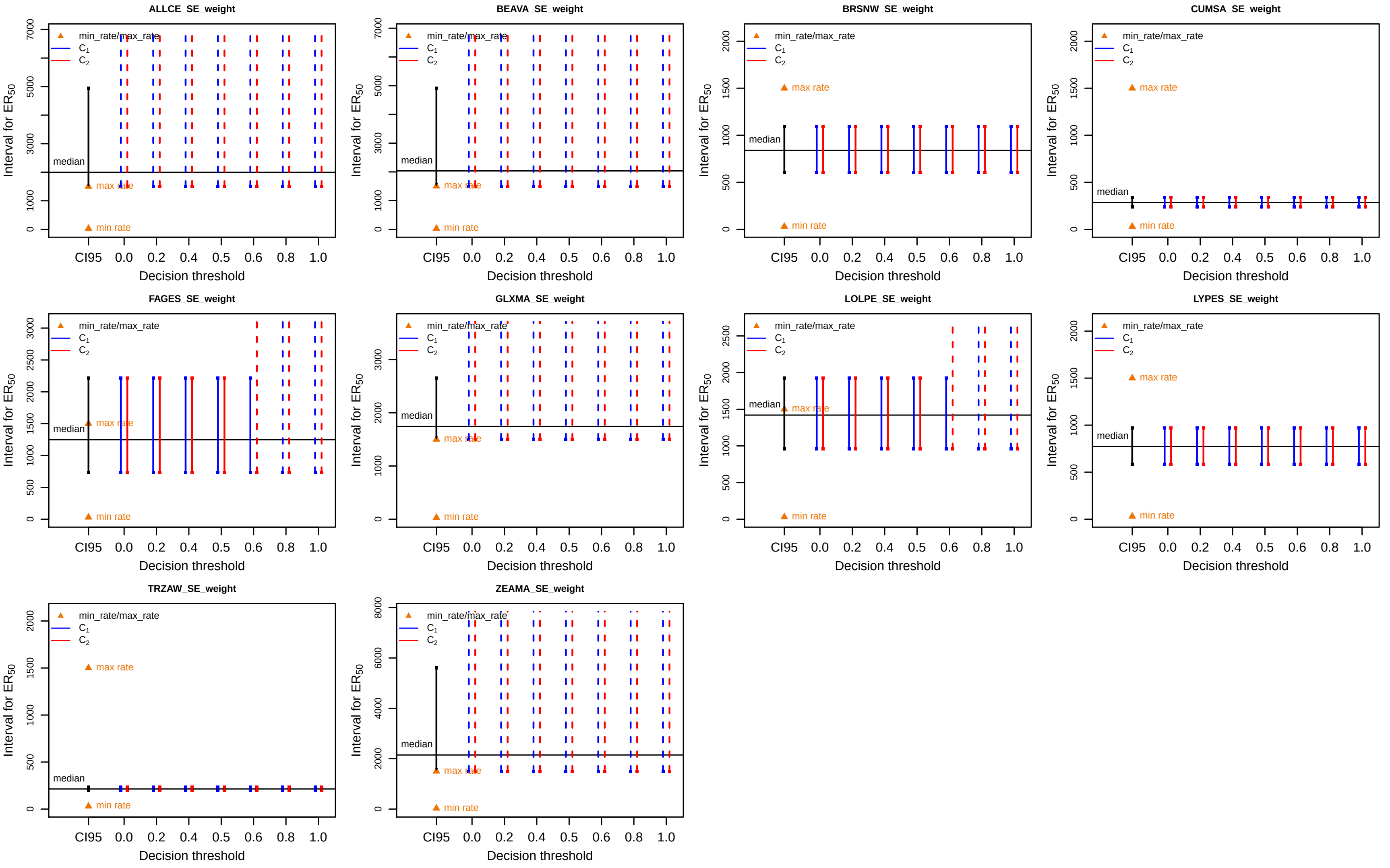

Figure 3: CI95 of ER_{50} and censored ER_{50} according to both criteria and the seven decision thresholds for all species - shoot dry weight endpoint of the SE test of study 3. The two orange triangles stand for the lowest and the highest tested rates. Vertical segments of three different colors (black, blue, red) represent the CI95 of ER_{50}, the censored ER_{50} according to C_1 and the censored ER_{50} according to C_2, respectively; solid vertical segments are for bounded intervals while dotted vertical segments stand for right-unbounded intervals; the black horizontal line represents the median of the ER_{50}.

Vegatative vigor (VV) test of study 3

Raw data description (test item rates) for VV test

```
Number of species:  10
EPPO code:  ALLCE BEAVA BRSNW CUMSA FAGES GLXMA LOLPE LYPES TRZAW ZEAMA
```

Table 12: Corresponding species and common names to the EPPO code

EPPO code	Species name	Common name
ALLCE	*Allium cepa*	Onion
BEAVA	*Beta vulgaris*	Sugar beet
BRSNW	*Brassica napus winter*	Oilseed rape
CUMSA	*Cucumis sativus*	Cucumber
FAGES	*Fagopyrum esculentum*	Buckwheat
GLXMA	*Glycine max*	Soybean
LOLPE	*Lolium perenne*	Perennial ryegrass
LYPES	*Solanum lycopersicum*	Tomato
TRZAW	*Triticum aestivum winter*	Winter wheat
ZEAMA	*Zea mays subs. Amylacea*	Corn

Table 13: Tested rates per species (ml product/ha) for the VV test of study 3

ALLCE	BEAVA	BRSNW	CUMSA	FAGES	GLXMA	LOLPE	LYPES	TRZAW	ZEAMA
0.0	0.0	0.0	0.0	0.0	0.0	0.0	0.0	0.0	0.0
30.0	30.0	30.0	30.0	30.0	30.0	30.0	30.0	30.0	30.0
79.8	79.8	79.8	79.8	79.8	79.8	79.8	79.8	79.8	79.8
212.0	212.0	212.0	212.0	212.0	212.0	212.0	212.0	212.0	212.0
564.0	564.0	564.0	564.0	564.0	564.0	564.0	564.0	564.0	564.0
1500.0	1500.0	1500.0	1500.0	1500.0	1500.0	1500.0	1500.0	1500.0	1500.0

VV test - survival endpoint

Table 14: Minimum and maximum tested rates, median, quantile 2.5% and quantile 97.5% of the ER_{50} posterior distribution - survival endpoint of the VV test of study 3

species	min_rate	max_rate	ER_{50}_median	ER_{50}_LCI95	ER_{50}_UCI95
ALLCE	30	1500	2307.7611	1616.7257	6305.021
BEAVA	30	1500	2328.1836	1626.1866	6317.185
BRSNW	30	1500	2307.5438	1619.2497	6248.358
CUMSA	30	1500	1190.1398	936.2344	1509.734
FAGES	30	1500	1094.0954	816.1594	1583.717
GLXMA	30	1500	2294.9298	1620.0415	6148.954
LOLPE	30	1500	1869.6714	1536.0866	3938.123
LYPES	30	1500	911.0478	712.4892	1174.539
TRZAW	30	1500	2292.9270	1617.4557	6114.726
ZEAMA	30	1500	2306.6144	1622.2776	6210.018

Table 15: Censored ER_{50} according to criterion 1 and the seven decision thresholds - survival endpoint of the VV test of study 3

species	C1T0.0	C1T0.2	C1T0.4	C1T0.5	C1T0.6	C1T0.8	C1T1.0
ALLCE	[1500; Inf)	[1500; Inf)	[1500; Inf)	[1500; Inf)	[1500; Inf)	[1500; Inf)	[1500; Inf)
BEAVA	[1500; Inf)	[1500; Inf)	[1500; Inf)	[1500; Inf)	[1500; Inf)	[1500; Inf)	[1500; Inf)
BRSNW	[1500; Inf)	[1500; Inf)	[1500; Inf)	[1500; Inf)	[1500; Inf)	[1500; Inf)	[1500; Inf)
CUMSA	[936.23; 1509.73]	[936.23; 1509.73]	[936.23; 1509.73]	[936.23; 1509.73]	[936.23; 1509.73]	[936.23; 1509.73]	[936.23; Inf)
FAGES	[816.16; 1583.72]	[816.16; 1583.72]	[816.16; 1583.72]	[816.16; 1583.72]	[816.16; 1583.72]	[816.16; 1583.72]	[816.16; Inf)
GLXMA	[1500; Inf)	[1500; Inf)	[1500; Inf)	[1500; Inf)	[1500; Inf)	[1500; Inf)	[1500; Inf)
LOLPE	[1500; Inf)	[1500; Inf)	[1500; Inf)	[1500; Inf)	[1500; Inf)	[1500; Inf)	[1500; Inf)
LYPES	[712.49; 1174.54]	[712.49; 1174.54]	[712.49; 1174.54]	[712.49; 1174.54]	[712.49; 1174.54]	[712.49; 1174.54]	[712.49; 1174.54]
TRZAW	[1500; Inf)	[1500; Inf)	[1500; Inf)	[1500; Inf)	[1500; Inf)	[1500; Inf)	[1500; Inf)
ZEAMA	[1500; Inf)	[1500; Inf)	[1500; Inf)	[1500; Inf)	[1500; Inf)	[1500; Inf)	[1500; Inf)

Table 16: Censored ER_{50} according to criterion 2 and the seven decision thresholds - survival endpoint of the VV test of study 3

species	C2T0.0	C2T0.2	C2T0.4	C2T0.5	C2T0.6	C2T0.8	C2T1.0
ALLCE	[1500; Inf)	[1500; Inf)	[1500; Inf)	[1500; Inf)	[1500; Inf)	[1500; Inf)	[1500; Inf)
BEAVA	[1500; Inf)	[1500; Inf)	[1500; Inf)	[1500; Inf)	[1500; Inf)	[1500; Inf)	[1500; Inf)
BRSNW	[1500; Inf)	[1500; Inf)	[1500; Inf)	[1500; Inf)	[1500; Inf)	[1500; Inf)	[1500; Inf)
CUMSA	[936.23; 1509.73]	[936.23; 1509.73]	[936.23; 1509.73]	[936.23; 1509.73]	[936.23; 1509.73]	[936.23; 1509.73]	[936.23; Inf)
FAGES	[816.16; 1583.72]	[816.16; 1583.72]	[816.16; 1583.72]	[816.16; 1583.72]	[816.16; 1583.72]	[816.16; 1583.72]	[816.16; Inf)
GLXMA	[1500; Inf)	[1500; Inf)	[1500; Inf)	[1500; Inf)	[1500; Inf)	[1500; Inf)	[1500; Inf)
LOLPE	[1500; Inf)	[1500; Inf)	[1500; Inf)	[1500; Inf)	[1500; Inf)	[1500; Inf)	[1500; Inf)
LYPES	[712.49; 1174.54]	[712.49; 1174.54]	[712.49; 1174.54]	[712.49; 1174.54]	[712.49; 1174.54]	[712.49; 1174.54]	[712.49; 1174.54]
TRZAW	[1500; Inf)	[1500; Inf)	[1500; Inf)	[1500; Inf)	[1500; Inf)	[1500; Inf)	[1500; Inf)
ZEAMA	[1500; Inf)	[1500; Inf)	[1500; Inf)	[1500; Inf)	[1500; Inf)	[1500; Inf)	[1500; Inf)

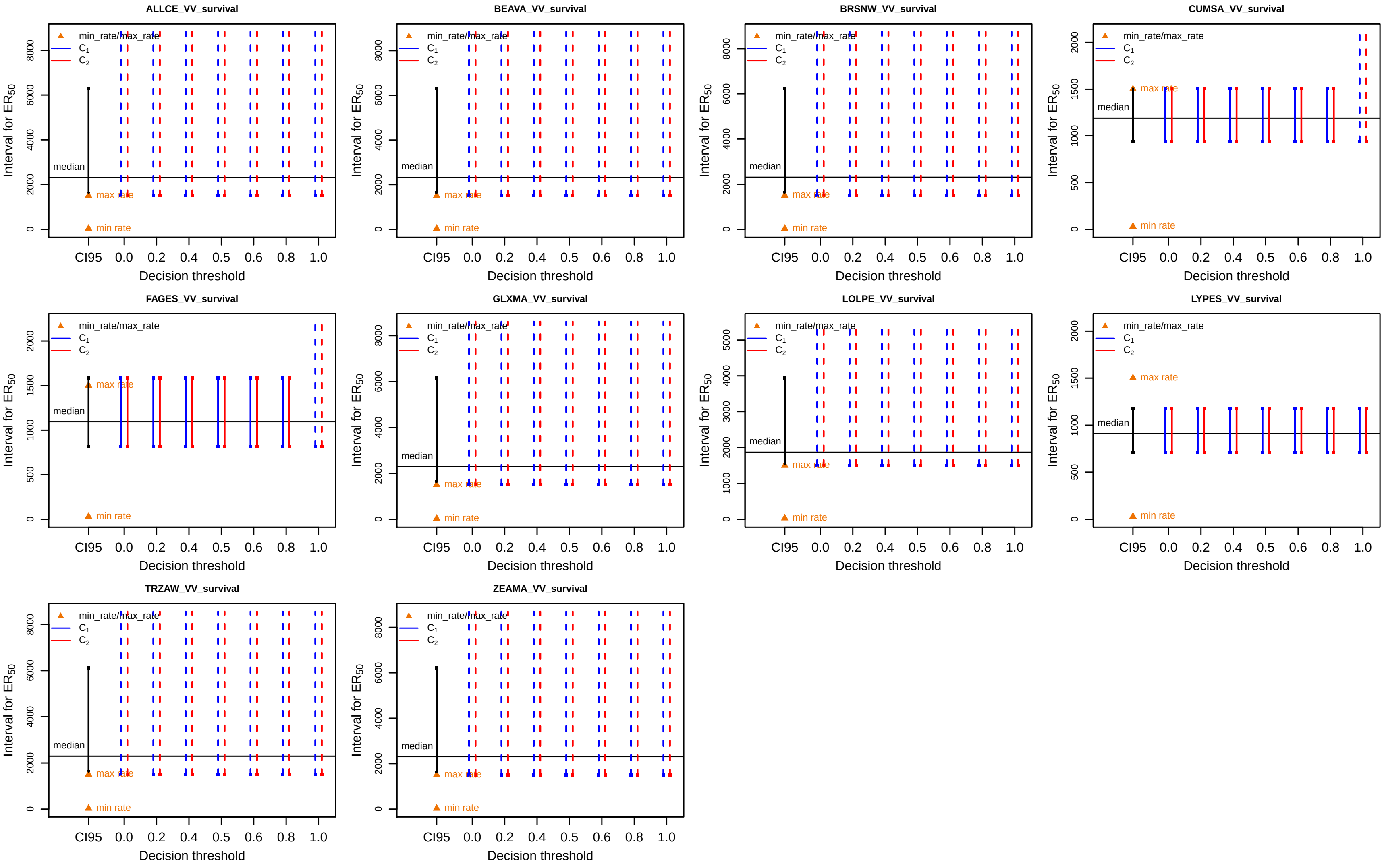

Figure 4: CI95 of ER_{50} and censored ER_{50} according to both criteria and the seven decision thresholds for all species - survival endpoint of the VV test of study 3. The two orange triangles stand for the lowest and the highest tested rates. Vertical segments of three different colors (black, blue, red) represent the CI95 of ER_{50}, the censored ER_{50} according to C_1 and the censored ER_{50} according to C_2, respectively; solid vertical segments are for bounded intervals while dotted vertical segments stand for right-unbounded intervals; the black horizontal line represents the median of the ER_{50}.

VV test - shoot dry weight endpoint

Table 17: Minimum and maximum tested rates, median, quantile 2.5% and quantile 97.5% of the ER_{50} posterior distribution - shoot dry weight endpoint of the VV test of study 3

species	min_rate	max_rate	ER_{50}_median	ER_{50}_LCI95	ER_{50}_UCI95
ALLCE	30	1500	1558.3999	1196.4205	2439.7662
BEAVA	30	1500	2377.9721	1609.3460	4221.2905
BRSNW	30	1500	838.5991	699.3952	1011.0980
CUMSA	30	1500	302.9751	245.5429	372.0404
FAGES	30	1500	262.0846	205.3369	333.3763
GLXMA	30	1500	1590.3538	1396.2621	1865.4753
LOLPE	30	1500	1038.1307	759.7044	1420.9466
LYPES	30	1500	147.9906	109.4036	199.4987
TRZAW	30	1500	342.3082	285.4423	409.9936
ZEAMA	30	1500	682.8282	586.5291	792.4331

Table 18: Censored ER_{50} according to criterion 1 and the seven decision thresholds - shoot dry weight endpoint of the VV test of study 3

species	C1T0.0	C1T0.2	C1T0.4	C1T0.5	C1T0.6	C1T0.8	C1T1.0
ALLCE	[1196.42; 2439.77]	[1196.42; 2439.77]	[1196.42; Inf)	[1196.42; Inf)	[1196.42; Inf)	[1196.42; Inf)	[1196.42; Inf)
BEAVA	[1500; Inf)	[1500; Inf)	[1500; Inf)	[1500; Inf)	[1500; Inf)	[1500; Inf)	[1500; Inf)
BRSNW	[699.4; 1011.1]	[699.4; 1011.1]	[699.4; 1011.1]	[699.4; 1011.1]	[699.4; 1011.1]	[699.4; 1011.1]	[699.4; 1011.1]
CUMSA	[245.54; 372.04]	[245.54; 372.04]	[245.54; 372.04]	[245.54; 372.04]	[245.54; 372.04]	[245.54; 372.04]	[245.54; 372.04]
FAGES	[205.34; 333.38]	[205.34; 333.38]	[205.34; 333.38]	[205.34; 333.38]	[205.34; 333.38]	[205.34; 333.38]	[205.34; 333.38]
GLXMA	[1396.26; 1865.48]	[1396.26; Inf)	[1396.26; Inf)	[1396.26; Inf)	[1396.26; Inf)	[1396.26; Inf)	[1396.26; Inf)
LOLPE	[759.7; 1420.95]	[759.7; 1420.95]	[759.7; 1420.95]	[759.7; 1420.95]	[759.7; 1420.95]	[759.7; 1420.95]	[759.7; 1420.95]
LYPES	[109.4; 199.5]	[109.4; 199.5]	[109.4; 199.5]	[109.4; 199.5]	[109.4; 199.5]	[109.4; 199.5]	[109.4; 199.5]
TRZAW	[285.44; 409.99]	[285.44; 409.99]	[285.44; 409.99]	[285.44; 409.99]	[285.44; 409.99]	[285.44; 409.99]	[285.44; 409.99]
ZEAMA	[586.53; 792.43]	[586.53; 792.43]	[586.53; 792.43]	[586.53; 792.43]	[586.53; 792.43]	[586.53; 792.43]	[586.53; 792.43]

Table 19: Censored ER_{50} according to criterion 2 and the seven decision thresholds - shoot dry weight endpoint of the VV test of study 3

species	C2T0.0	C2T0.2	C2T0.4	C2T0.5	C2T0.6	C2T0.8	C2T1.0
ALLCE	[1196.42; 2439.77]	[1196.42; 2439.77]	[1196.42; Inf)	[1196.42; Inf)	[1196.42; Inf)	[1196.42; Inf)	[1196.42; Inf)
BEAVA	[1500; Inf)	[1500; Inf)	[1500; Inf)	[1500; Inf)	[1500; Inf)	[1500; Inf)	[1500; Inf)
BRSNW	[699.4; 1011.1]	[699.4; 1011.1]	[699.4; 1011.1]	[699.4; 1011.1]	[699.4; 1011.1]	[699.4; 1011.1]	[699.4; 1011.1]
CUMSA	[245.54; 372.04]	[245.54; 372.04]	[245.54; 372.04]	[245.54; 372.04]	[245.54; 372.04]	[245.54; 372.04]	[245.54; 372.04]
FAGES	[205.34; 333.38]	[205.34; 333.38]	[205.34; 333.38]	[205.34; 333.38]	[205.34; 333.38]	[205.34; 333.38]	[205.34; 333.38]
GLXMA	[1396.26; 1865.48]	[1396.26; 1865.48]	[1396.26; Inf)	[1396.26; Inf)	[1396.26; Inf)	[1396.26; Inf)	[1396.26; Inf)
LOLPE	[759.7; 1420.95]	[759.7; 1420.95]	[759.7; 1420.95]	[759.7; 1420.95]	[759.7; 1420.95]	[759.7; 1420.95]	[759.7; 1420.95]
LYPES	[109.4; 199.5]	[109.4; 199.5]	[109.4; 199.5]	[109.4; 199.5]	[109.4; 199.5]	[109.4; 199.5]	[109.4; 199.5]
TRZAW	[285.44; 409.99]	[285.44; 409.99]	[285.44; 409.99]	[285.44; 409.99]	[285.44; 409.99]	[285.44; 409.99]	[285.44; 409.99]
ZEAMA	[586.53; 792.43]	[586.53; 792.43]	[586.53; 792.43]	[586.53; 792.43]	[586.53; 792.43]	[586.53; 792.43]	[586.53; 792.43]

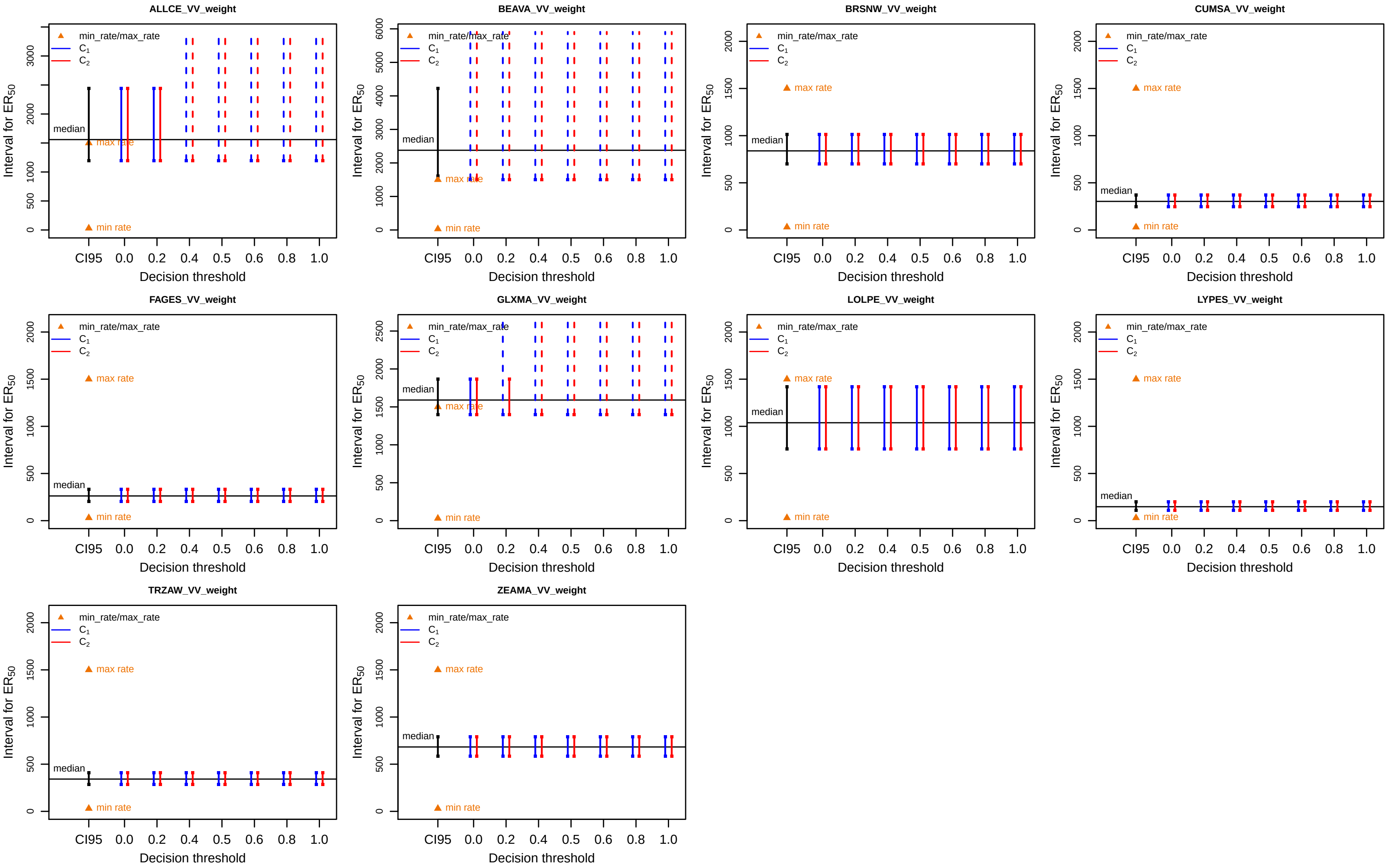

Figure 5: CI95 of ER_{50} and censored ER_{50} according to both criteria and the seven decision thresholds for all species - shoot dry weight endpoint of the VV test of study 3. The two orange triangles stand for the lowest and the highest tested rates. Vertical segments of three different colors (black, blue, red) represent the CI95 of ER_{50}, the censored ER_{50} according to C_1 and the censored ER_{50} according to C_2, respectively; solid vertical segments are for bounded intervals while dotted vertical segments stand for right-unbounded intervals; the black horizontal line represents the median of the ER_{50}.
