# Supplementary material for: How to account for the uncertainty from standard toxicity tests in species sensitivity distributions: An example in non-target plants
Source: PLoS One. 2021 Jan 7;16(1):e0245071. doi: 10.1371/journal.pone.0245071 (PMC7790375; doi:10.1371/journal.pone.0245071)
Supplement: S1 Archive — It is a zip file containing seven folders (one folder per case study). Each folder contains five files report_xxx.pdf with detailed results of the dose-response analyses, one file corresponding to does-response analysis per endpoint. It also contains one file ER50_censoring.pdf for censored ER50 and one file SSD_analyses.pdf for results of SSD analyses. (ZIP) [file pone.0245071.s004.zip › S1_archive/Study3/report_SE_emergence.pdf]

Dose-response analyses

Study 3

Seedling Emergence test - emergence endpoint

25 June 2020

Contact: sandrine.charles@univ-lyon1.fr

This is a report which provides results on all performed dose-response analyses for the emergence endpoint of the Seedling Emergence test for study 3.

Contents

Data set: ALLCE_SE_emergence 2
Data set: BEAVA_SE_emergence 3
Data set: BRSNW_SE_emergence 4
Data set: CUMSA_SE_emergence 5
Data set: FAGES_SE_emergence 6
Data set: GLXMA_SE_emergence 7
Data set: LOLPE_SE_emergence 8
Data set: LYPES_SE_emergence 9
Data set: TRZAW_SE_emergence 10
Data set: ZEAMA_SE_emergence 11

Data set: ALLCE_SE_emergence

Table 1: Summary of parameter estimates for ALLCE_SE_emergence data set

Parameter	median	Q2.5	Q97.5
b	6.208	0.404	75.086
d	0.793	0.704	0.936
e	1742.724	1386.898	4285.237

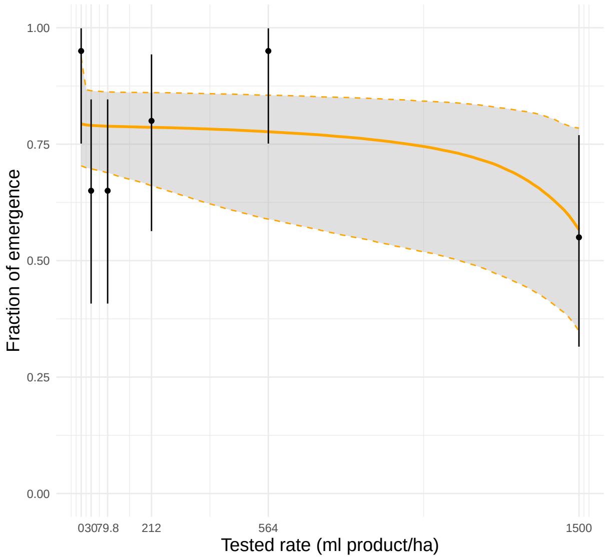

(a) Dose-response curve

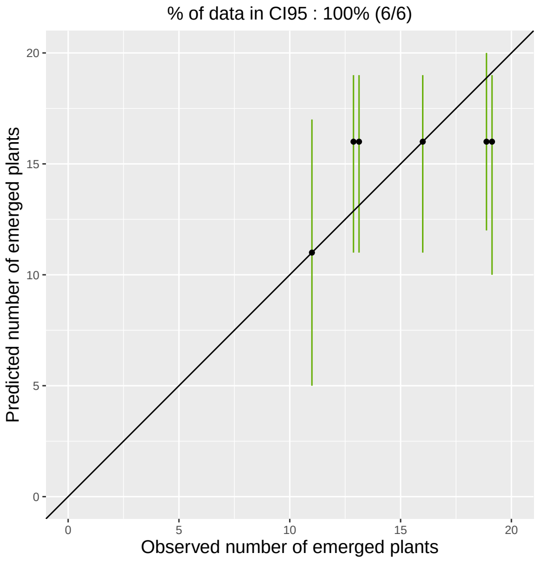

(b) Posterior predictive check (PPC)

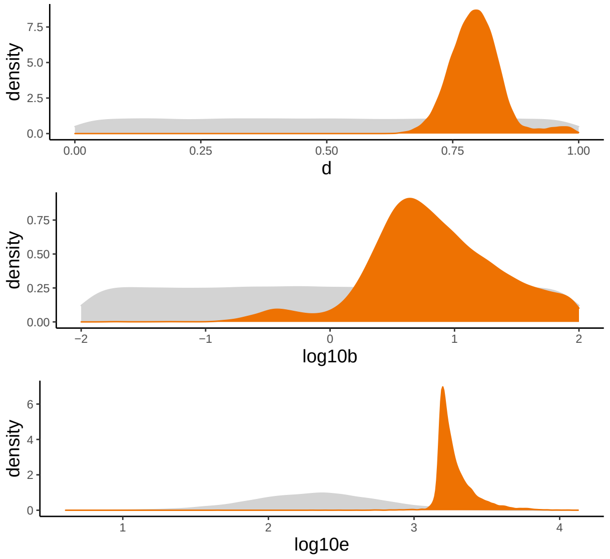

(c) Priors and posteriors

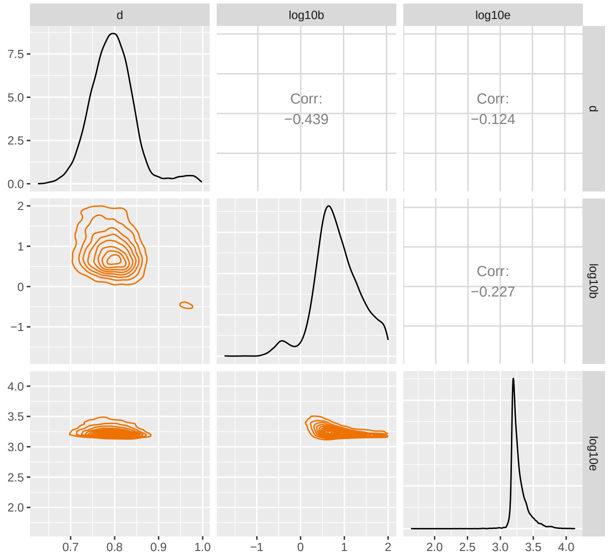

(d) Correlations between parameters

Figure 1: Dose-response curve (a), PPC (b), prior and posterior distributions (c) and correlations between parameters (d).

Data set: BEAVA_SE_emergence

Table 2: Summary of parameter estimates for BEAVA_SE_emergence data set

Parameter	median	Q2.5	Q97.5
b	31.797	4.206	94.386
d	0.962	0.918	0.986
e	2314.562	1618.871	6004.500

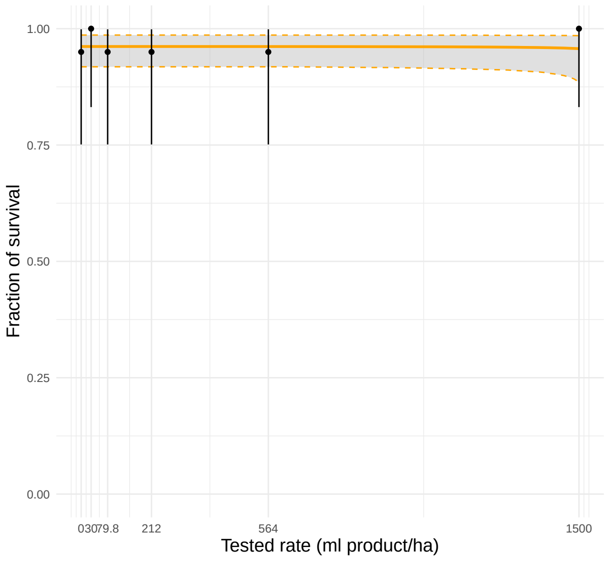

(a) Dose-response curve

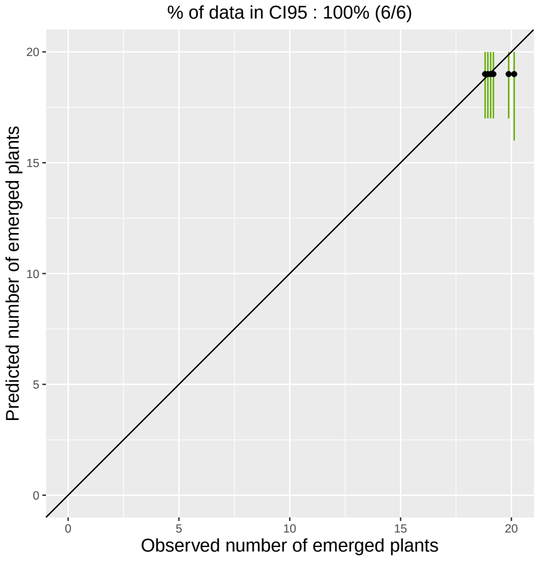

(b) Posterior predictive check (PPC)

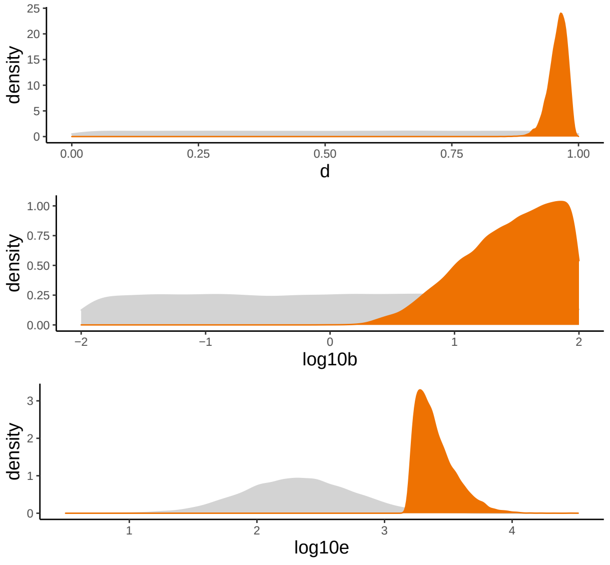

(c) Priors and posteriors

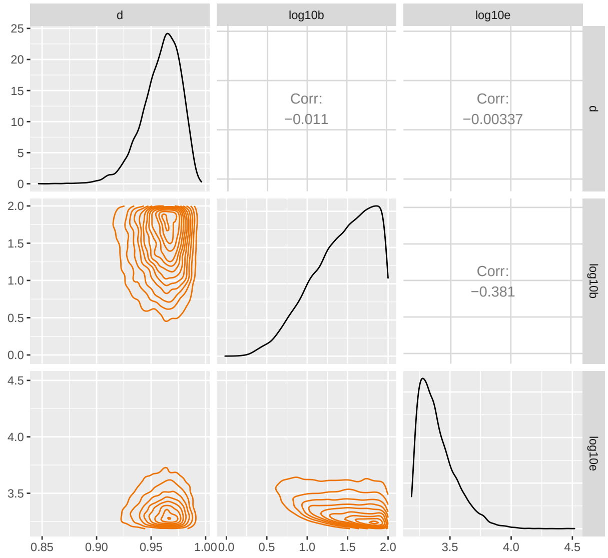

(d) Correlations between parameters

Figure 2: Dose-response curve (a), PPC (b), prior and posterior distributions (c) and correlations between parameters (d).

Data set: BRSNW_SE_emergence

Table 3: Summary of parameter estimates for BRSNW_SE_emergence data set

Parameter	median	Q2.5	Q97.5
b	19.929	1.788	92.748
d	0.942	0.887	0.978
e	2218.620	1583.885	5925.315

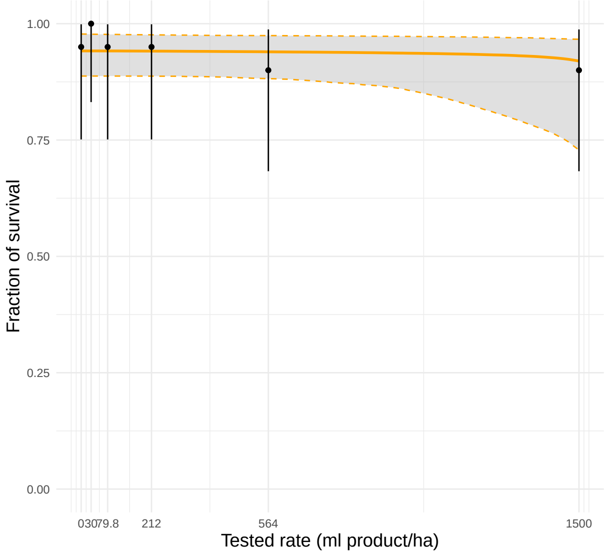

(a) Dose-response curve

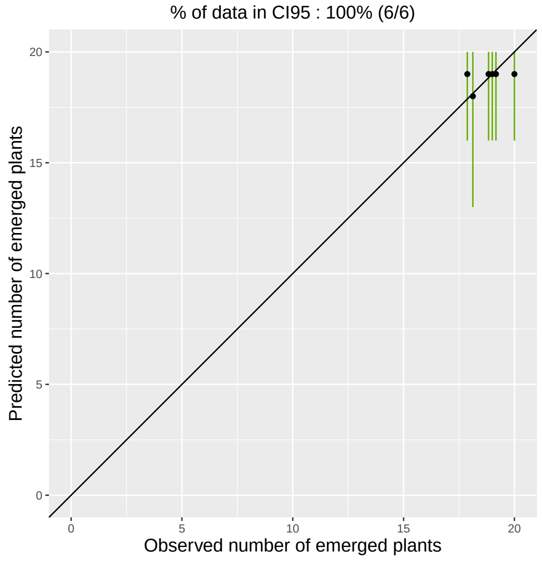

(b) Posterior predictive check (PPC)

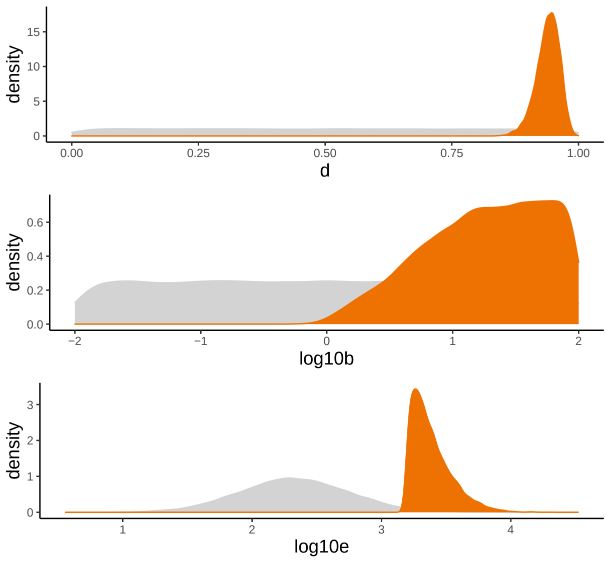

(c) Priors and posteriors

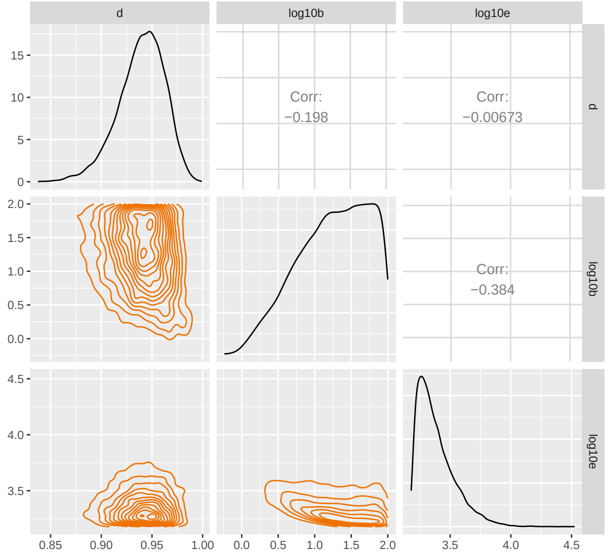

(d) Correlations between parameters

Figure 3: Dose-response curve (a), PPC (b), prior and posterior distributions (c) and correlations between parameters (d).

Data set: CUMSA_SE_emergence

Table 4: Summary of parameter estimates for CUMSA_SE_emergence data set

Parameter	median	Q2.5	Q97.5
b	28.851	3.342	94.485
d	0.905	0.845	0.948
e	2289.370	1605.898	6129.611

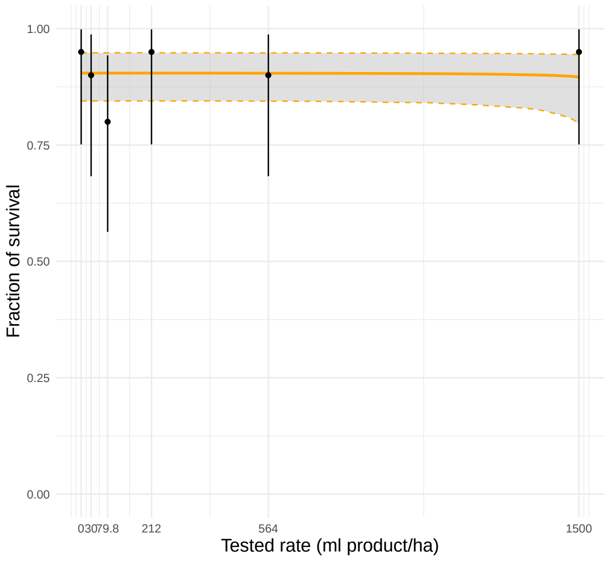

(a) Dose-response curve

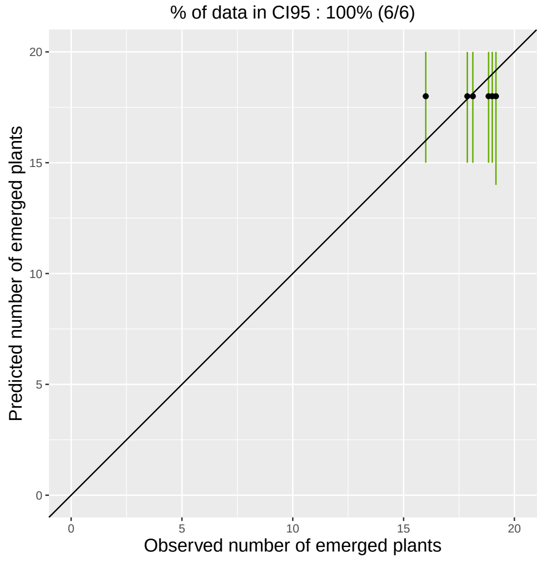

(b) Posterior predictive check (PPC)

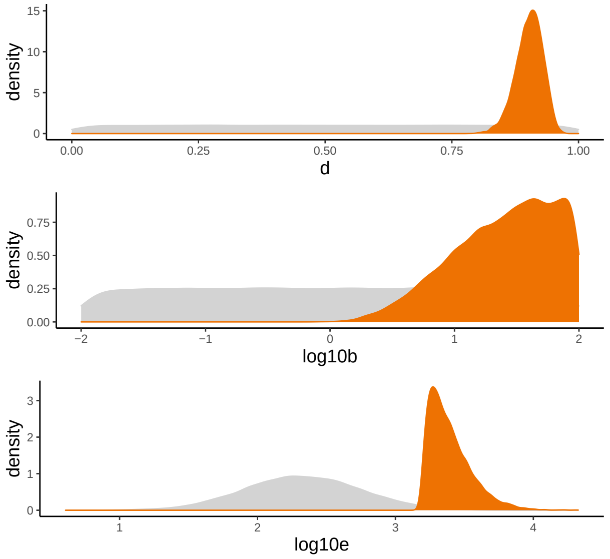

(c) Priors and posteriors

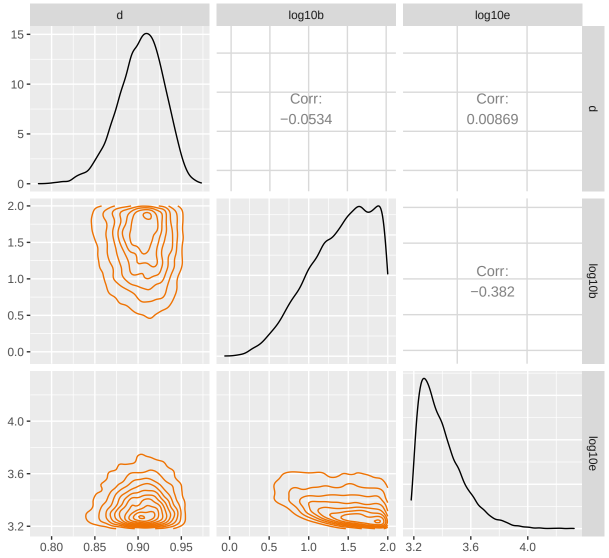

(d) Correlations between parameters

Figure 4: Dose-response curve (a), PPC (b), prior and posterior distributions (c) and correlations between parameters (d).

Data set: FAGES_SE_emergence

Table 5: Summary of parameter estimates for FAGES_SE_emergence data set

Parameter	median	Q2.5	Q97.5
b	27.233	2.658	94.217
d	0.864	0.798	0.918
e	2269.224	1600.636	6043.838

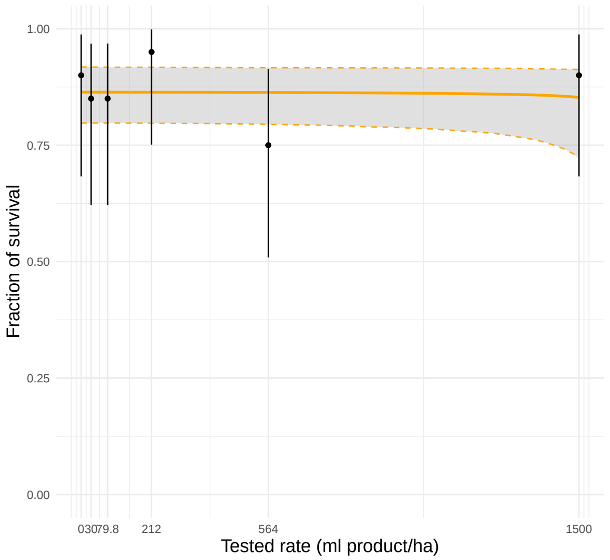

(a) Dose-response curve

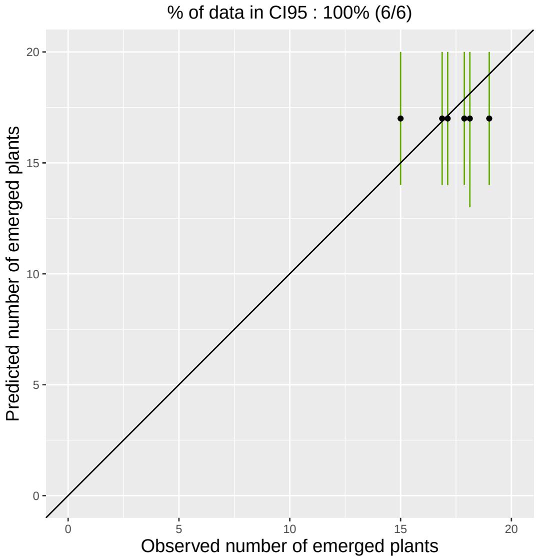

(b) Posterior predictive check (PPC)

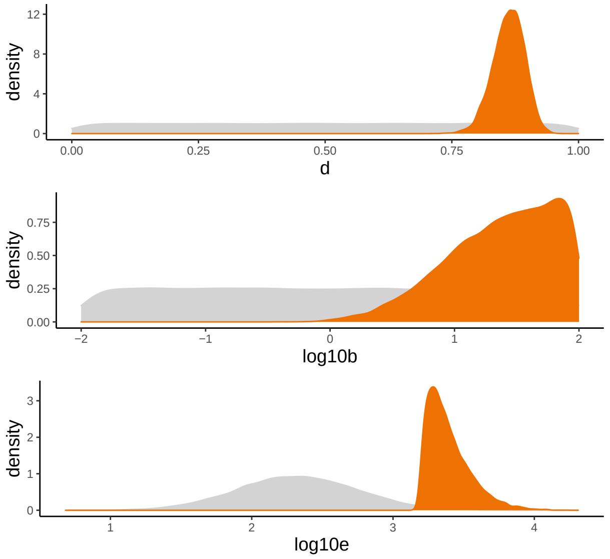

(c) Priors and posteriors

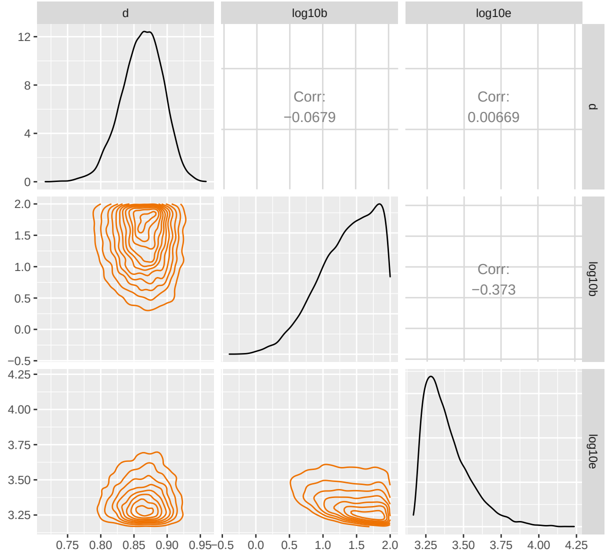

(d) Correlations between parameters

Figure 5: Dose-response curve (a), PPC (b), prior and posterior distributions (c) and correlations between parameters (d).

Data set: GLXMA_SE_emergence

Table 6: Summary of parameter estimates (parameter d is set to 1) for GLXMA_SE_emergence data set

Parameter	median	Q2.5	Q97.5
b	1.035	0.65	1.636
e	5292.275	2475.91	15515.520

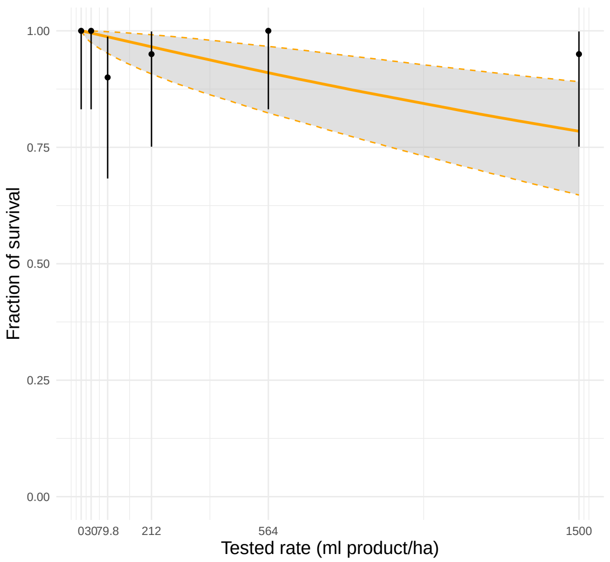

(a) Dose-response curve

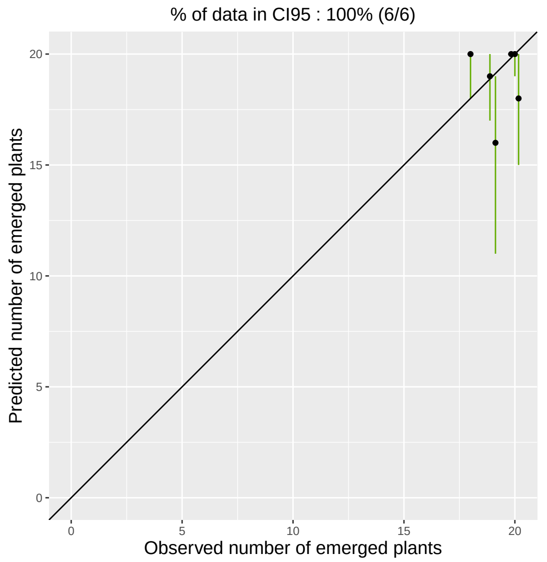

(b) Posterior predictive check (PPC)

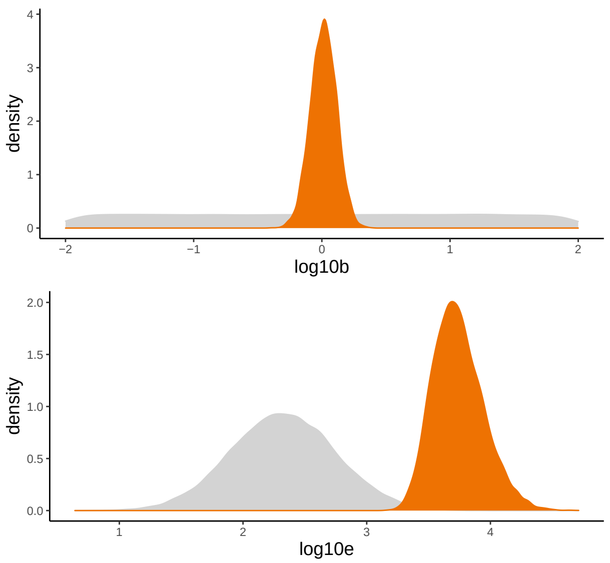

(c) Priors and posteriors

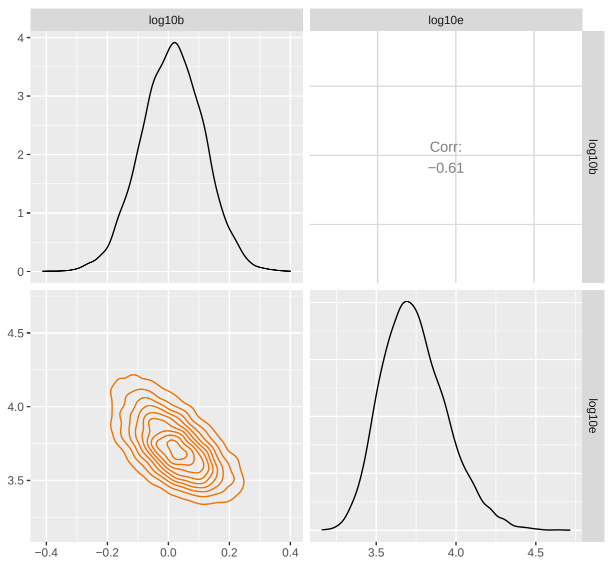

(d) Correlations between parameters

Figure 6: Dose-response curve (a), PPC (b), prior and posterior distributions (c) and correlations between parameters (d).

Data set: LOLPE_SE_emergence

Table 7: Summary of parameter estimates for LOLPE_SE_emergence data set

Parameter	median	Q2.5	Q97.5
b	24.548	2.401	93.117
d	0.849	0.779	0.906
e	2250.291	1589.317	5903.808

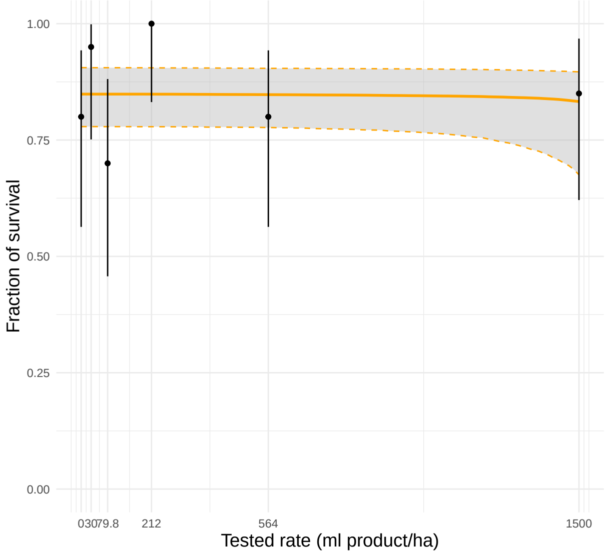

(a) Dose-response curve

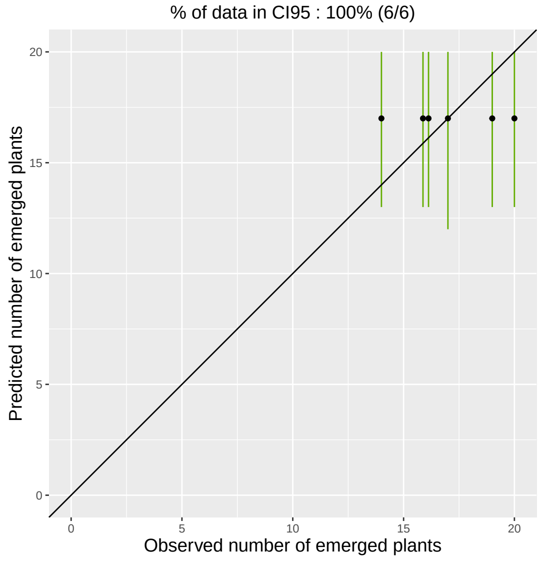

(b) Posterior predictive check (PPC)

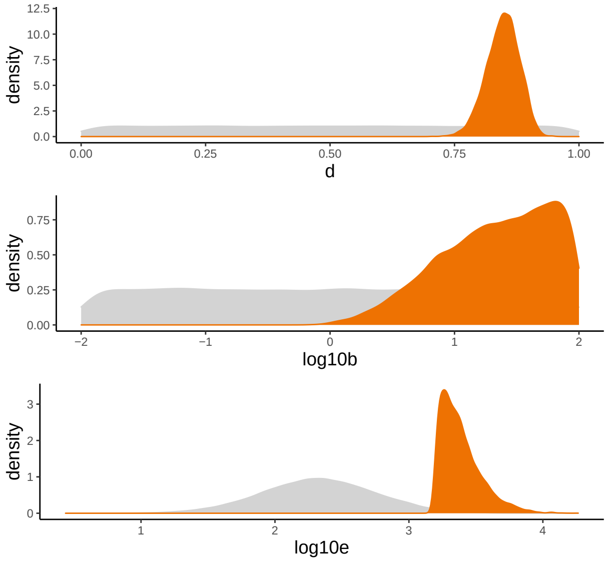

(c) Priors and posteriors

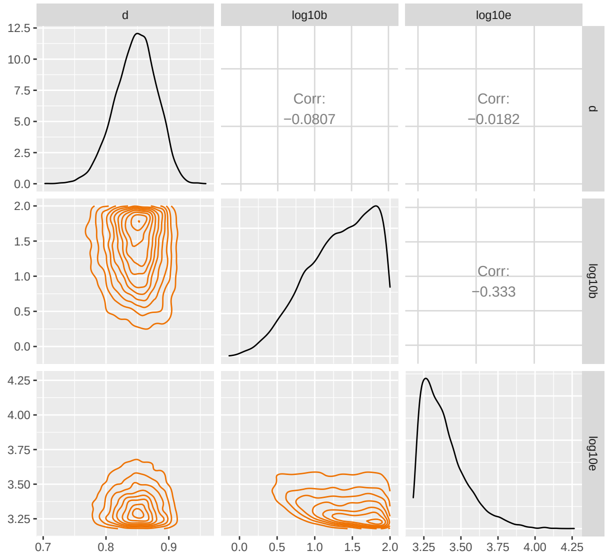

(d) Correlations between parameters

Figure 7: Dose-response curve (a), PPC (b), prior and posterior distributions (c) and correlations between parameters (d).

Data set: LYPES_SE_emergence

Table 8: Summary of parameter estimates for LYPES_SE_emergence data set

Parameter	median	Q2.5	Q97.5
b	22.348	2.289	93.094
d	0.932	0.876	0.970
e	2229.507	1585.341	5793.903

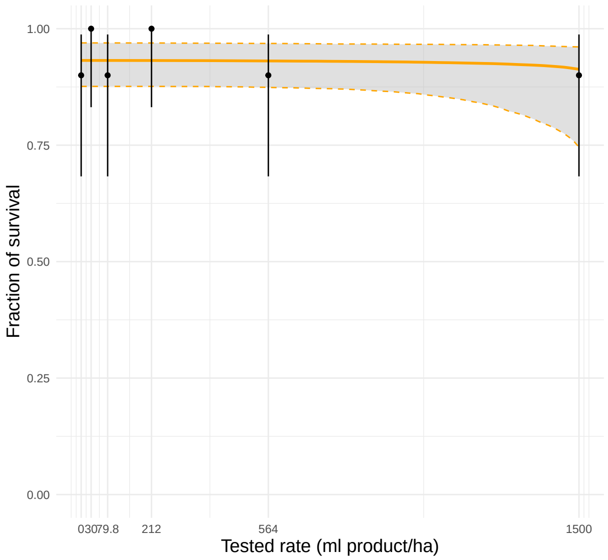

(a) Dose-response curve

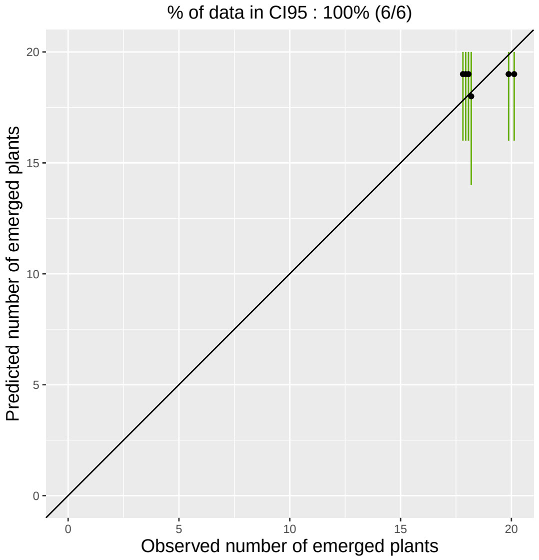

(b) Posterior predictive check (PPC)

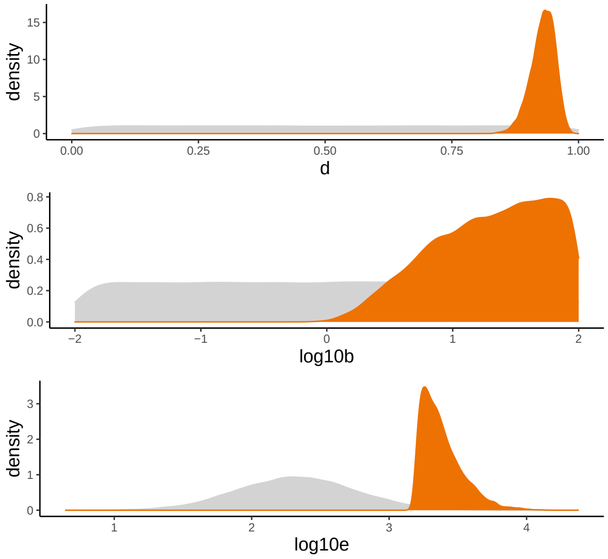

(c) Priors and posteriors

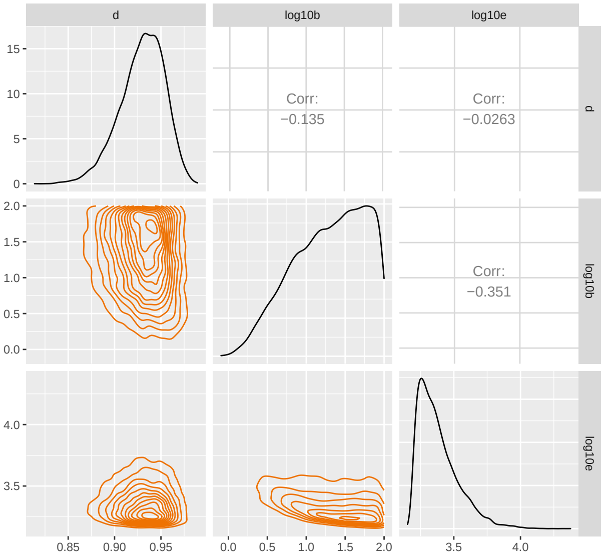

(d) Correlations between parameters

Figure 8: Dose-response curve (a), PPC (b), prior and posterior distributions (c) and correlations between parameters (d).

Data set: TRZAW_SE_emergence

Table 9: Summary of parameter estimates for TRZAW_SE_emergence data set

Parameter	median	Q2.5	Q97.5
b	25.690	3.213	93.378
d	0.963	0.918	0.988
e	2257.608	1595.494	6090.512

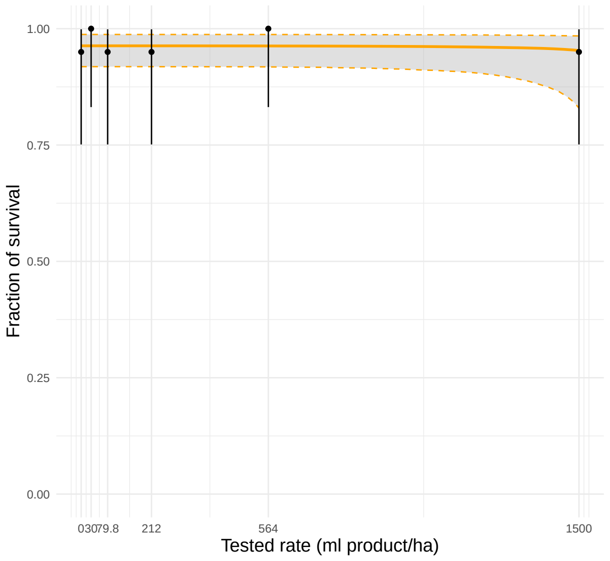

(a) Dose-response curve

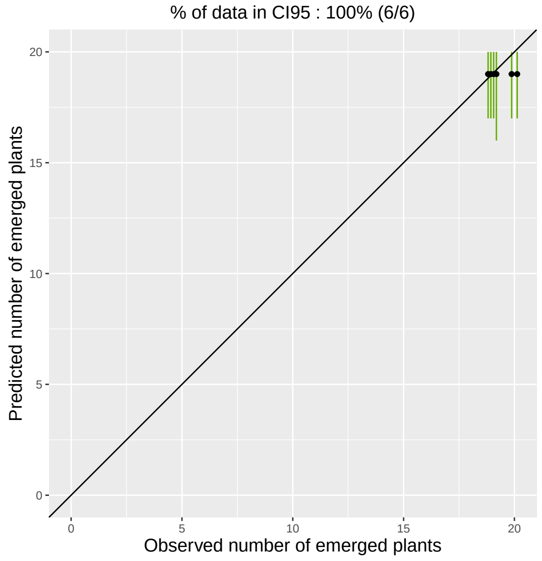

(b) Posterior predictive check (PPC)

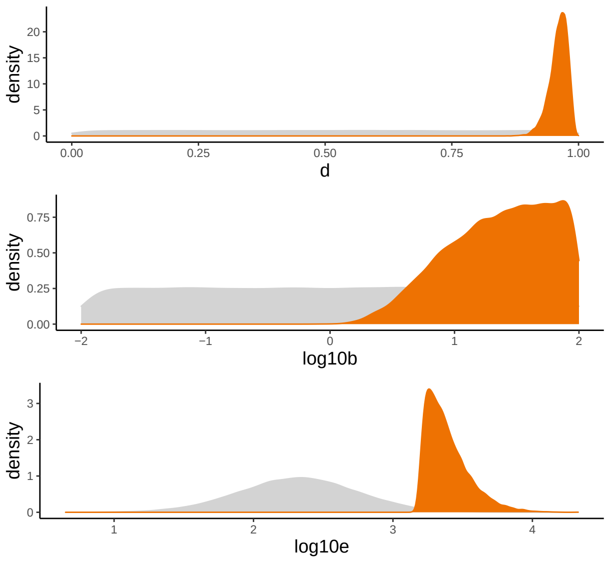

(c) Priors and posteriors

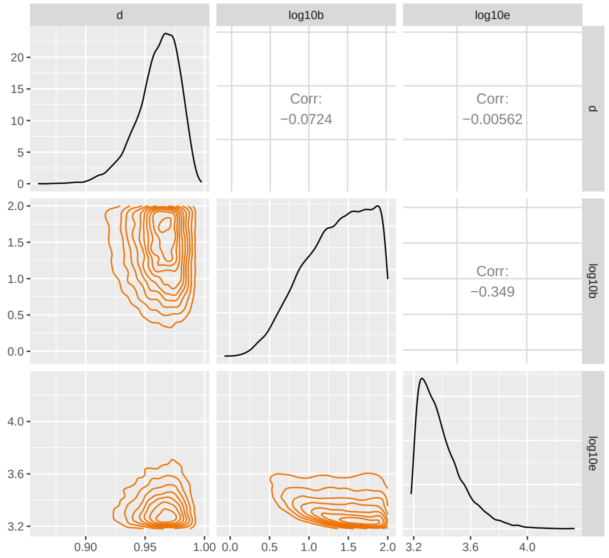

(d) Correlations between parameters

Figure 9: Dose-response curve (a), PPC (b), prior and posterior distributions (c) and correlations between parameters (d).

Data set: ZEAMA_SE_emergence

Table 10: Summary of parameter estimates (parameter d is set to 1) for ZEAMA_SE_emergence data set

Parameter	median	Q2.5	Q97.5
b	1.400	0.842	2.366
e	4389.401	2291.687	11578.225

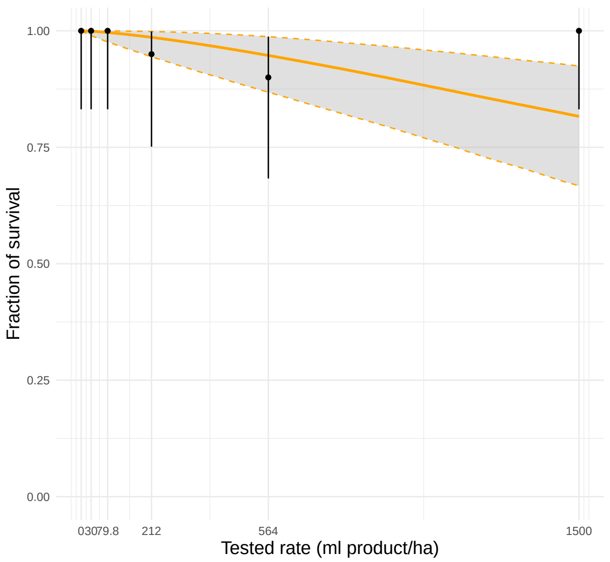

(a) Dose-response curve

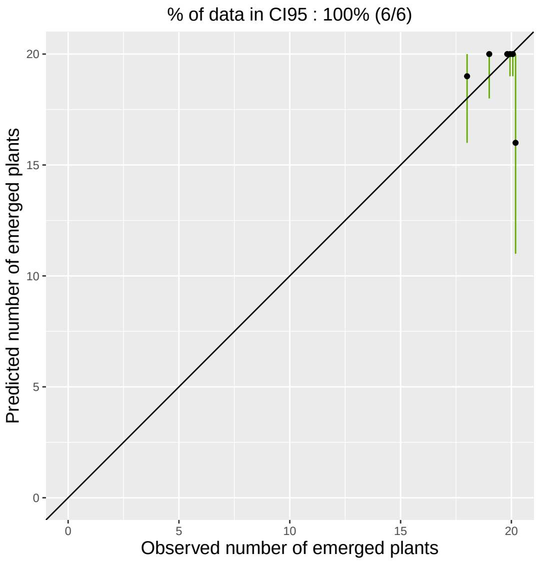

(b) Posterior predictive check (PPC)

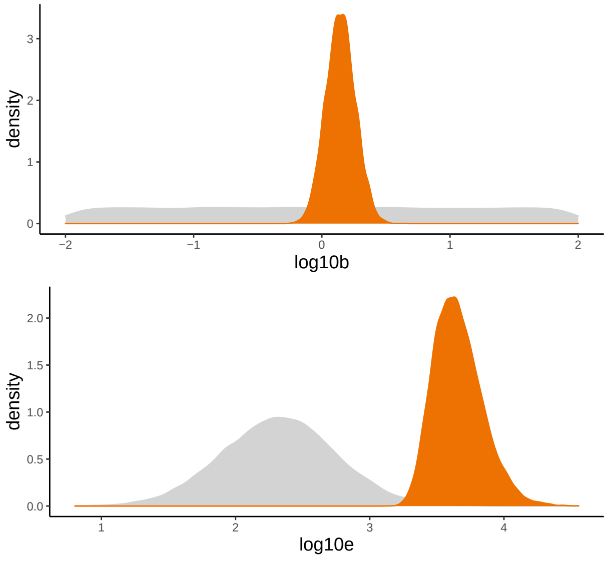

(c) Priors and posteriors

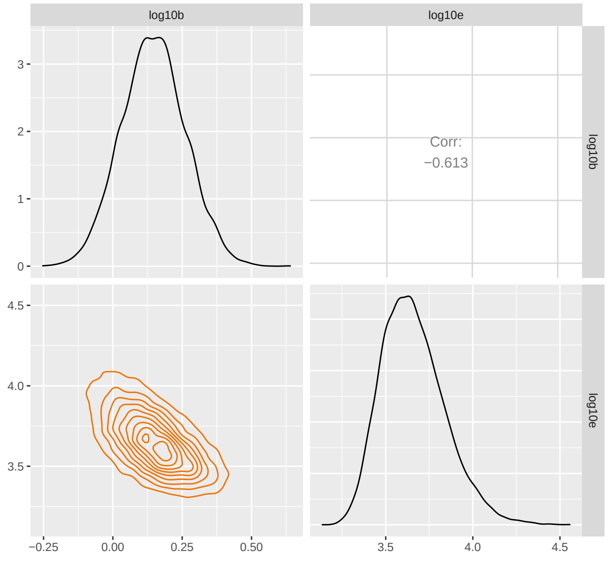

(d) Correlations between parameters

Figure 10: Dose-response curve (a), PPC (b), prior and posterior distributions (c) and correlations between parameters (d).
