# Supplementary material for: How to account for the uncertainty from standard toxicity tests in species sensitivity distributions: An example in non-target plants
Source: PLoS One. 2021 Jan 7;16(1):e0245071. doi: 10.1371/journal.pone.0245071 (PMC7790375; doi:10.1371/journal.pone.0245071)
Supplement: S1 Archive — It is a zip file containing seven folders (one folder per case study). Each folder contains five files report_xxx.pdf with detailed results of the dose-response analyses, one file corresponding to does-response analysis per endpoint. It also contains one file ER50_censoring.pdf for censored ER50 and one file SSD_analyses.pdf for results of SSD analyses. (ZIP) [file pone.0245071.s004.zip › S1_archive/Study3/report_SE_survival.pdf]

Dose-response analyses

Study 3

Seedling Emergence test - survival endpoint

25 June 2020

Contact: sandrine.charles@univ-lyon1.fr

This is a report which provides results on all performed dose-response analyses for the survival endpoint of the Seedling Emergence test for study 3.

Contents

Data set: ALLCE_SE_survival . . . 2
Data set: BEAVA_SE_survival . . . 3
Data set: BRSNW_SE_survival . . . 4
Data set: CUMSA_SE_survival . . . 5
Data set: FAGES_SE_survival . . . 6
Data set: GLXMA_SE_survival . . . 7
Data set: LOLPE_SE_survival . . . 8
Data set: LYPES_SE_survival . . . 9
Data set: TRZAW_SE_survival . . . 10
Data set: ZEAMA_SE_survival . . . 11

Data set: ALLCE_SE_survival

Table 1: Summary of parameter estimates (parameter d is set to 1) for ALLCE_SE_survival data set

Parameter	median	Q2.5	Q97.5
b	29.526	3.697	95.007
e	2272.116	1603.563	6155.935

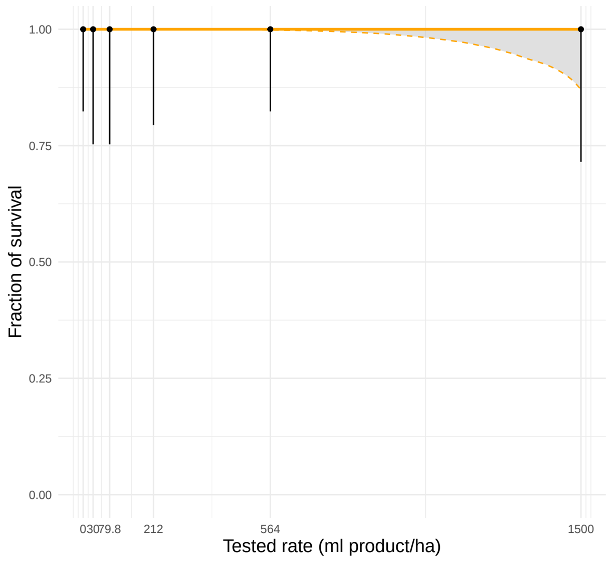

(a) Dose-response curve

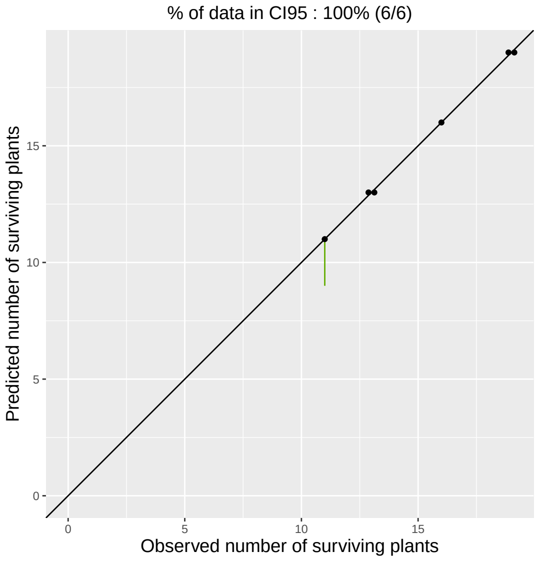

(b) Posterior predictive check (PPC)

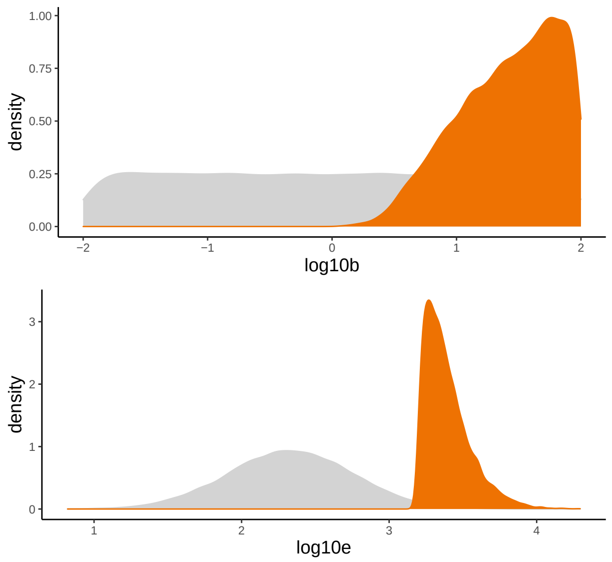

(c) Priors and posteriors

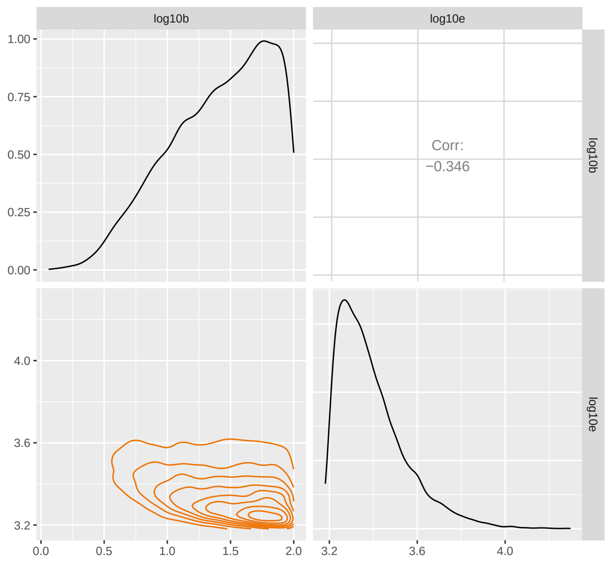

(d) Correlations between parameters

Figure 1: Dose-response curve (a), PPC (b), prior and posterior distributions (c) and correlations between parameters (d).

Data set: BEAVA_SE_survival

Table 2: Summary of parameter estimates (parameter d is set to 1) for BEAVA_SE_survival data set

Parameter	median	Q2.5	Q97.5
b	31.338	4.181	95.024
e	2311.868	1620.401	5999.744

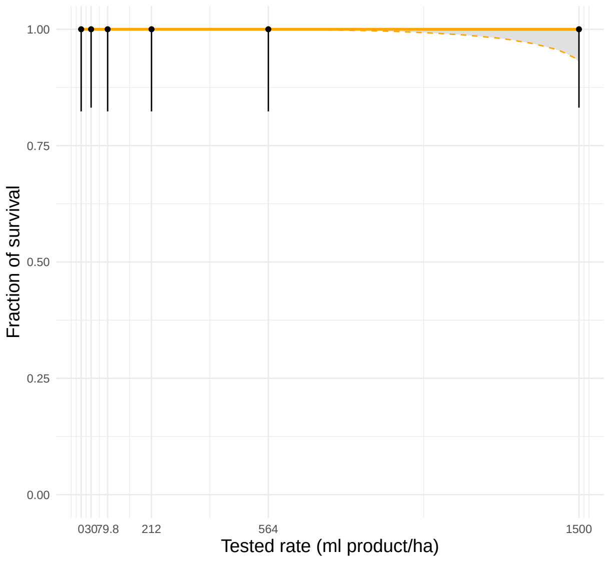

(a) Dose-response curve

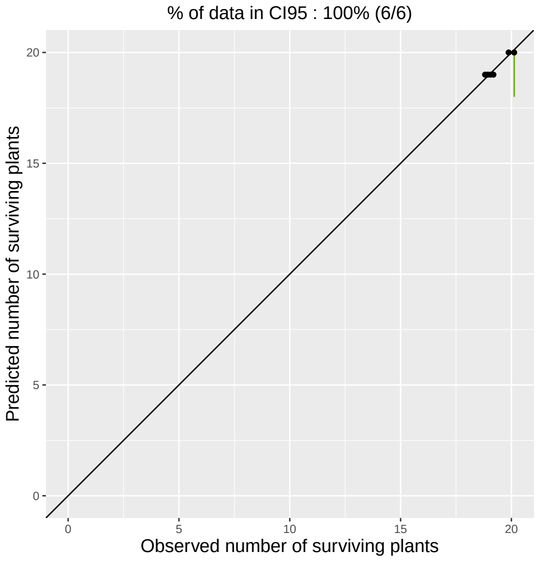

(b) Posterior predictive check (PPC)

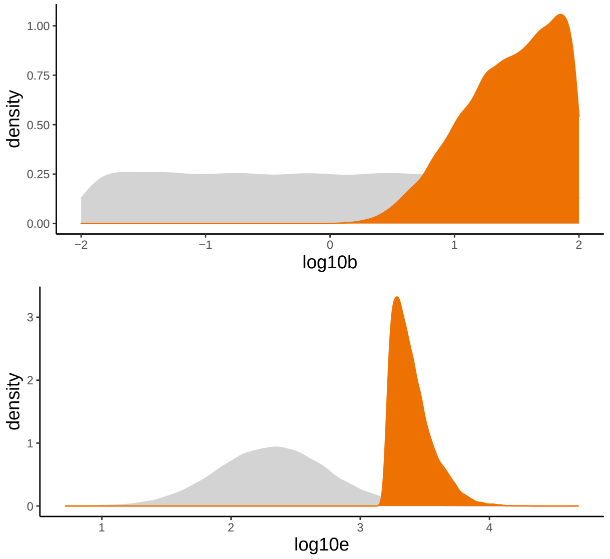

(c) Priors and posteriors

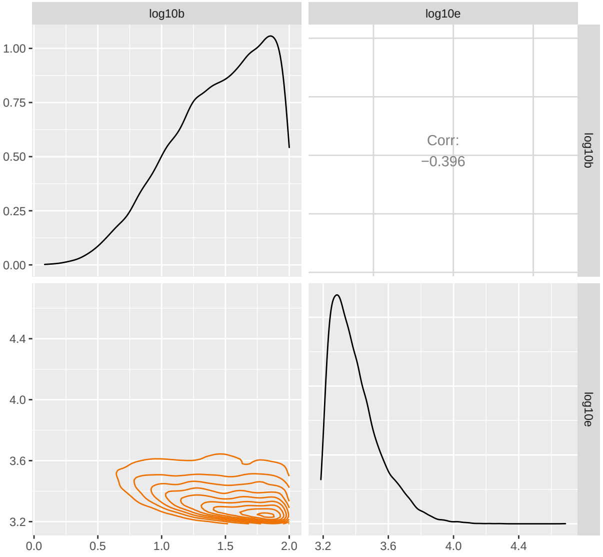

(d) Correlations between parameters

Figure 2: Dose-response curve (a), PPC (b), prior and posterior distributions (c) and correlations between parameters (d).

Data set: BRSNW_SE_survival

Table 3: Summary of parameter estimates (parameter d is set to 1) for BRSNW_SE_survival data set

Parameter	median	Q2.5	Q97.5
b	7.022	2.656	57.657
e	1377.016	1073.671	1557.188

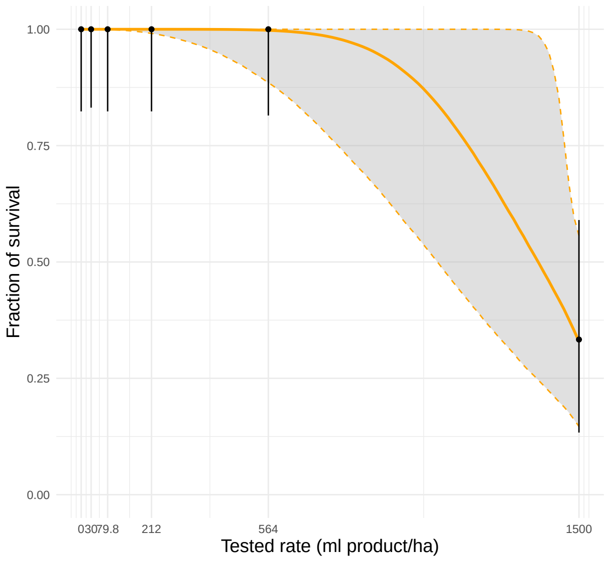

(a) Dose-response curve

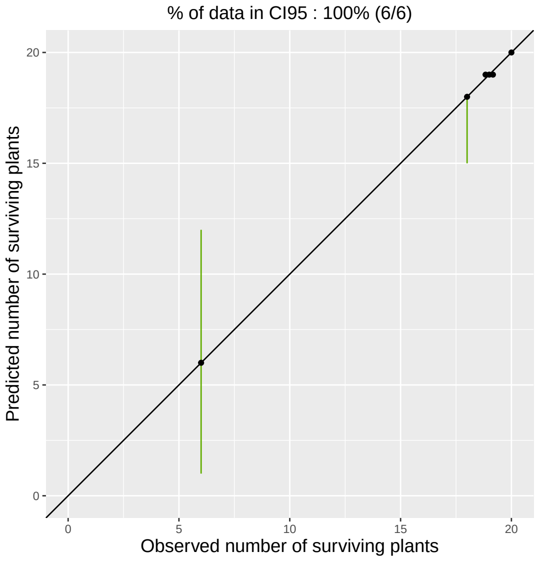

(b) Posterior predictive check (PPC)

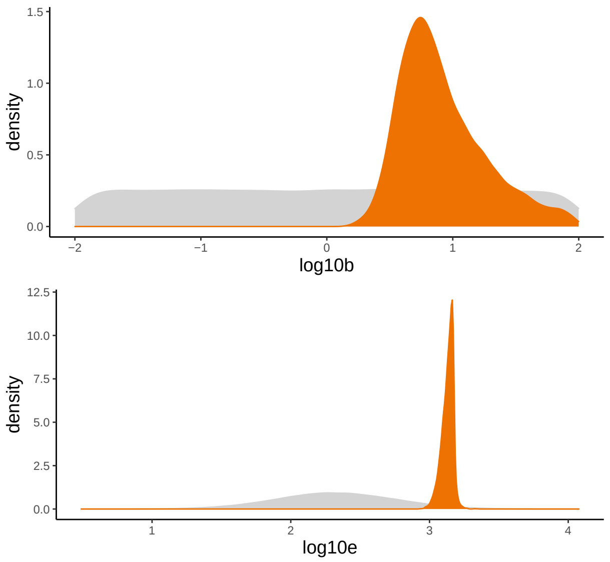

(c) Priors and posteriors

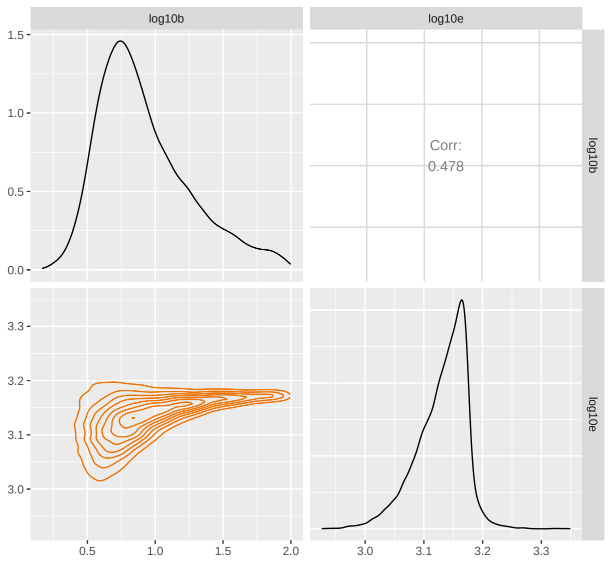

(d) Correlations between parameters

Figure 3: Dose-response curve (a), PPC (b), prior and posterior distributions (c) and correlations between parameters (d).

Data set: CUMSA_SE_survival

Table 4: Summary of parameter estimates (parameter d is set to 1) for CUMSA_SE_survival data set

Parameter	median	Q2.5	Q97.5
b	4.430	2.516	7.529
e	1024.014	796.974	1279.489

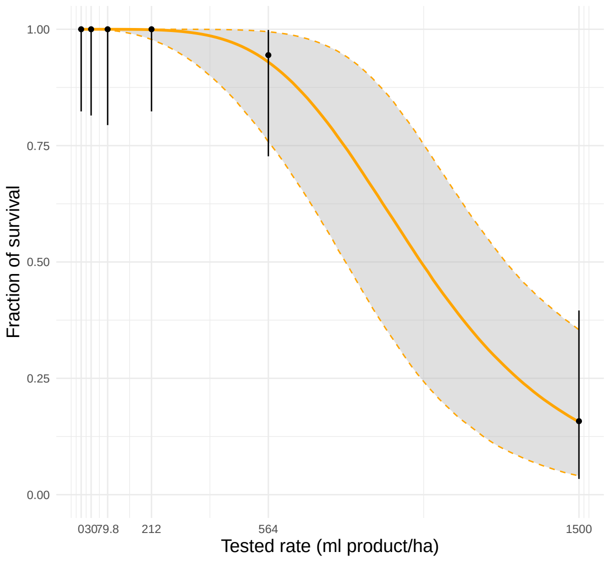

(a) Dose-response curve

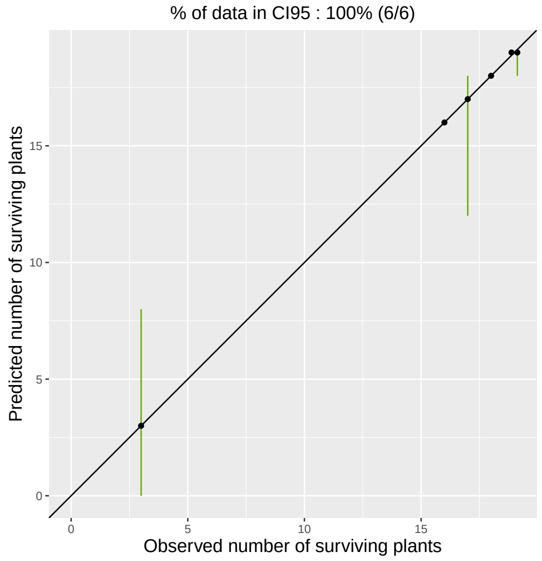

(b) Posterior predictive check (PPC)

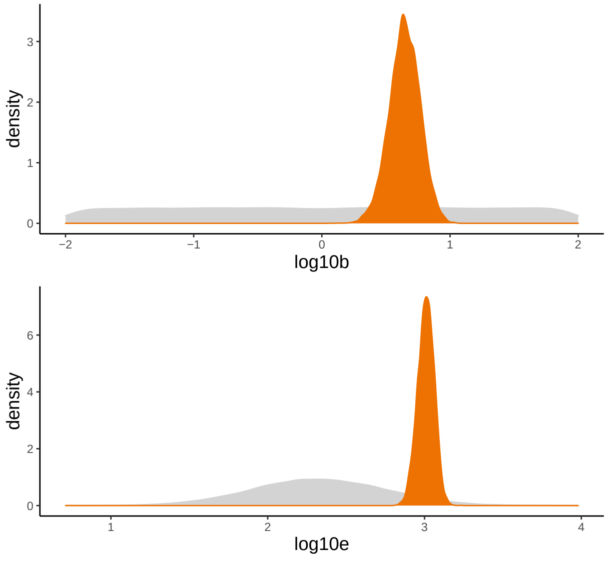

(c) Priors and posteriors

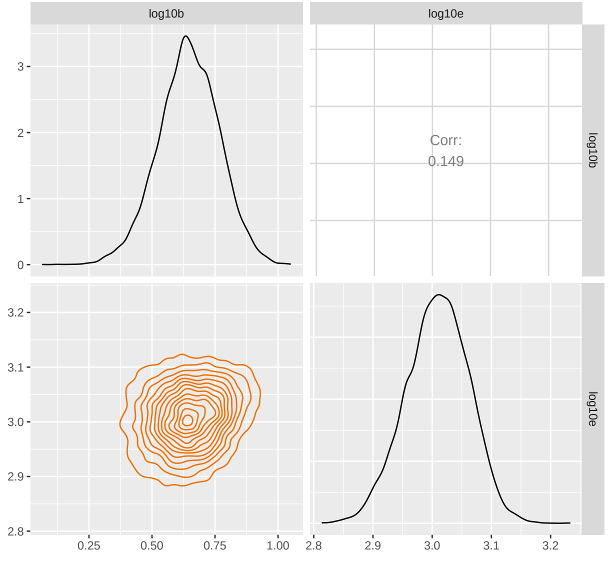

(d) Correlations between parameters

Figure 4: Dose-response curve (a), PPC (b), prior and posterior distributions (c) and correlations between parameters (d).

Data set: FAGES_SE_survival

Table 5: Summary of parameter estimates (parameter d is set to 1) for FAGES_SE_survival data set

Parameter	median	Q2.5	Q97.5
b	6.562	1.734	61.971
e	1870.314	1532.043	3884.872

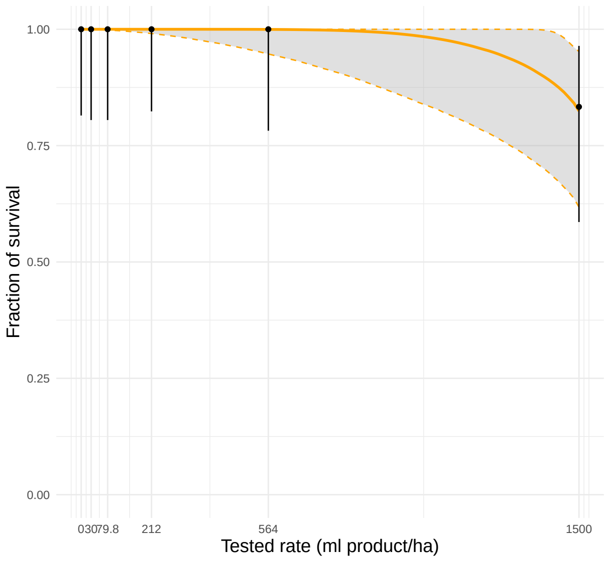

(a) Dose-response curve

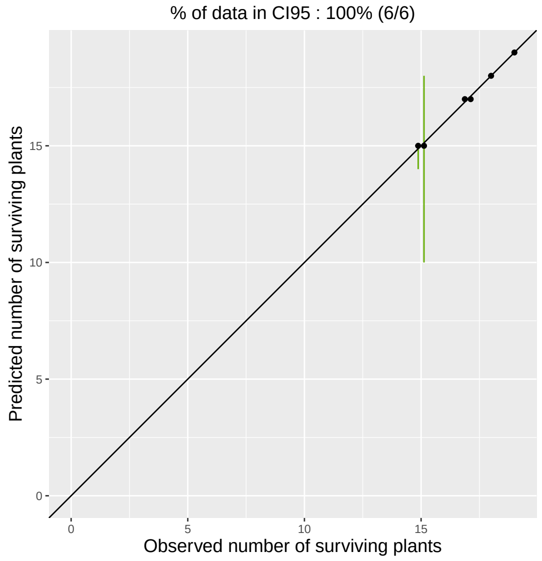

(b) Posterior predictive check (PPC)

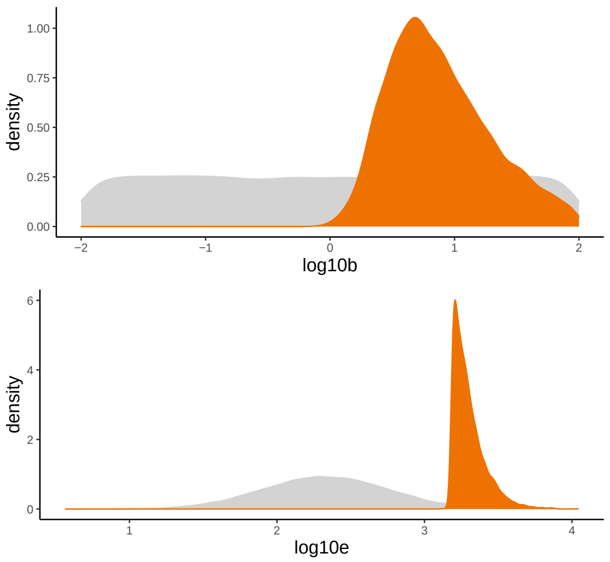

(c) Priors and posteriors

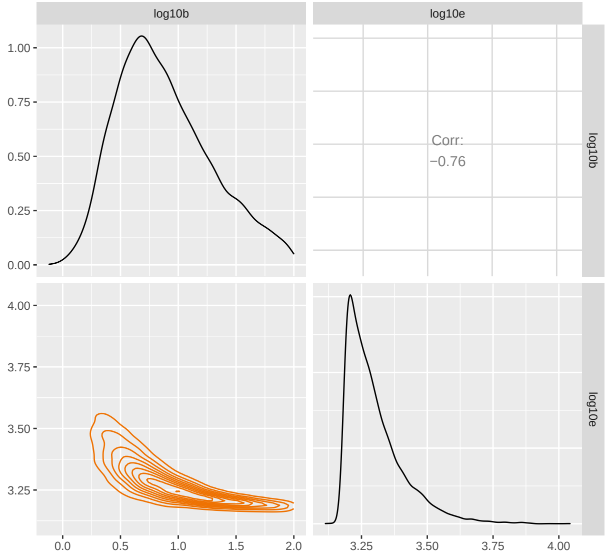

(d) Correlations between parameters

Figure 5: Dose-response curve (a), PPC (b), prior and posterior distributions (c) and correlations between parameters (d).

Data set: GLXMA_SE_survival

Table 6: Summary of parameter estimates (parameter d is set to 1) for GLXMA_SE_survival data set

Parameter	median	Q2.5	Q97.5
b	31.926	4.190	95.159
e	2318.128	1618.988	6272.430

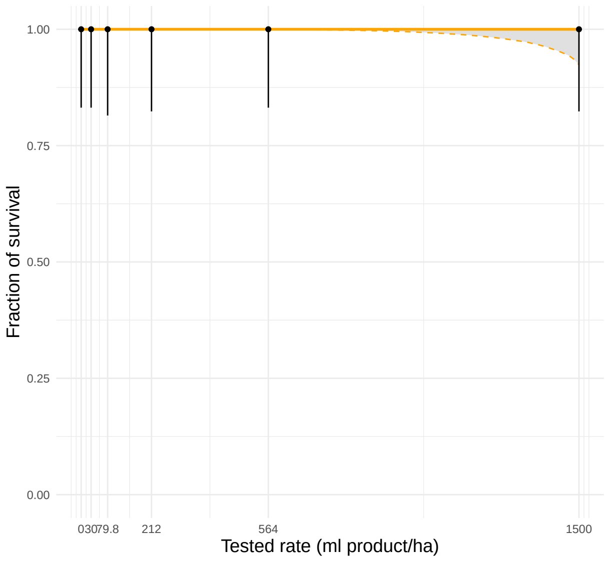

(a) Dose-response curve

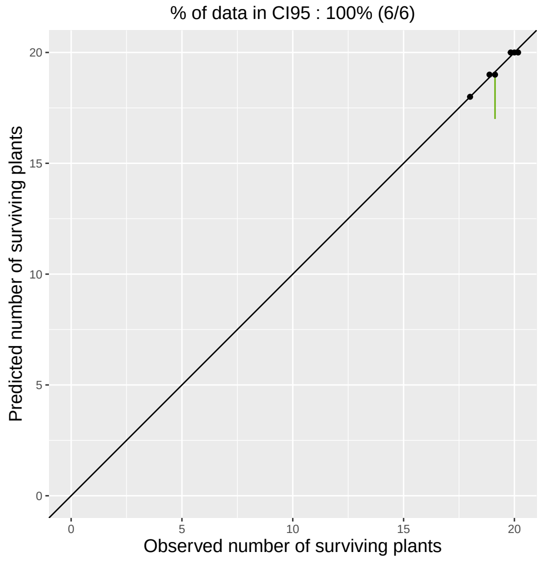

(b) Posterior predictive check (PPC)

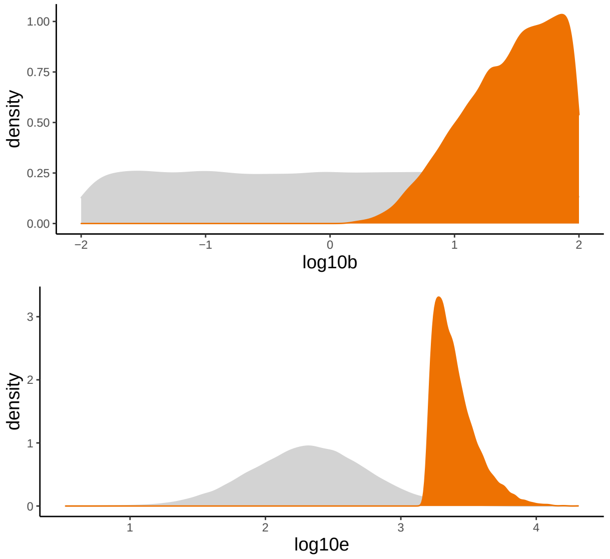

(c) Priors and posteriors

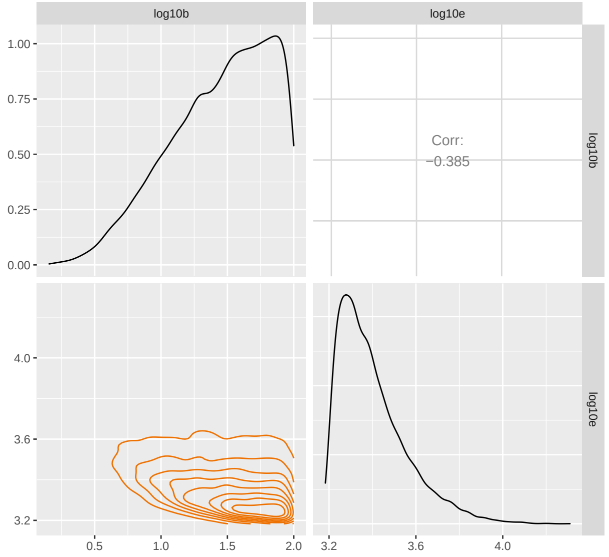

(d) Correlations between parameters

Figure 6: Dose-response curve (a), PPC (b), prior and posterior distributions (c) and correlations between parameters (d).

Data set: LOLPE_SE_survival

Table 7: Summary of parameter estimates (parameter d is set to 1) for LOLPE_SE_survival data set

Parameter	median	Q2.5	Q97.5
b	1.810	0.947	3.380
e	2461.879	1537.680	5801.728

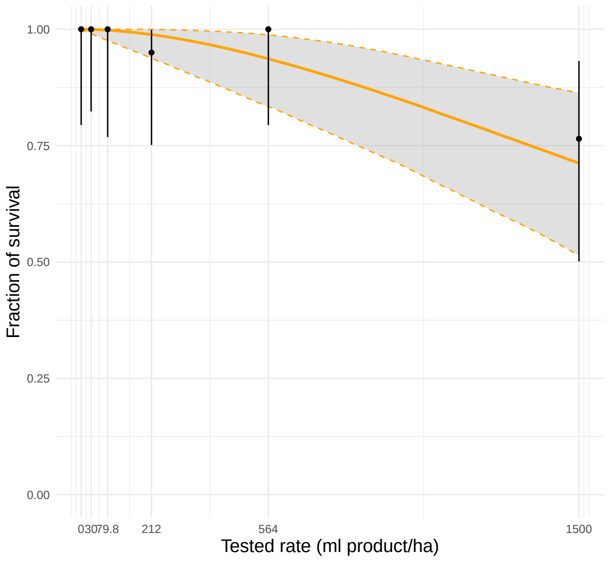

(a) Dose-response curve

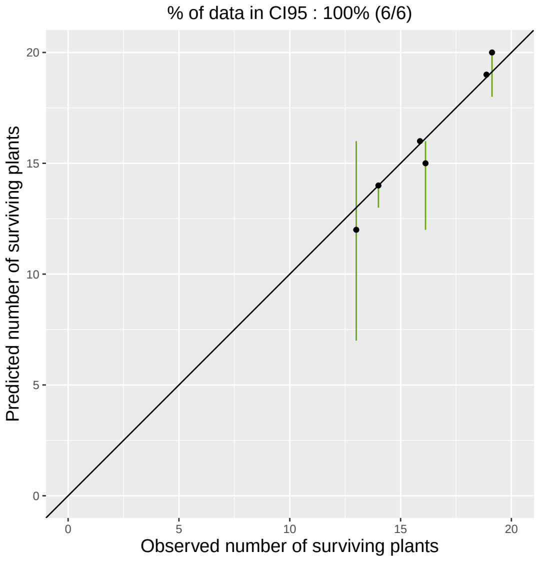

(b) Posterior predictive check (PPC)

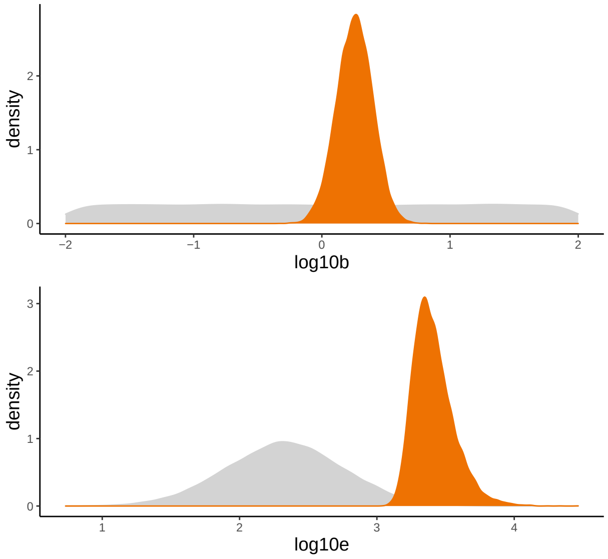

(c) Priors and posteriors

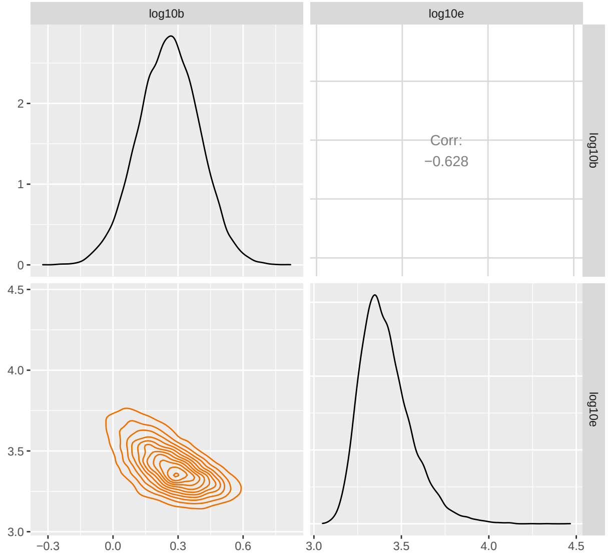

(d) Correlations between parameters

Figure 7: Dose-response curve (a), PPC (b), prior and posterior distributions (c) and correlations between parameters (d).

Data set: LYPES_SE_survival

Table 8: Summary of parameter estimates (parameter d is set to 1) for LYPES_SE_survival data set

Parameter	median	Q2.5	Q97.5
b	6.617	1.742	65.892
e	1857.573	1530.865	3901.110

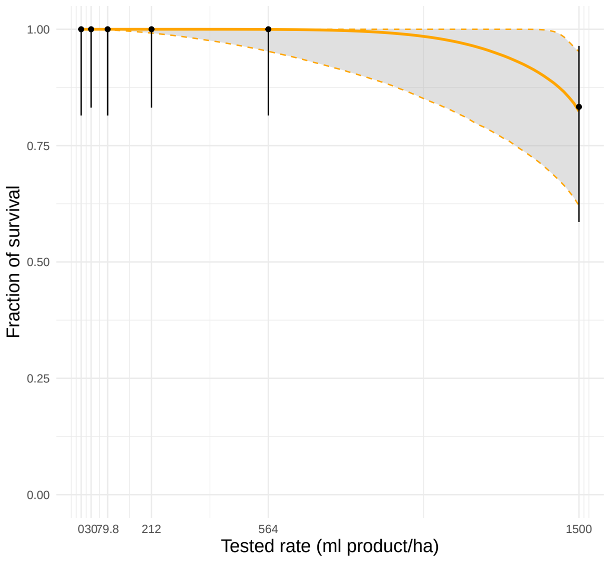

(a) Dose-response curve

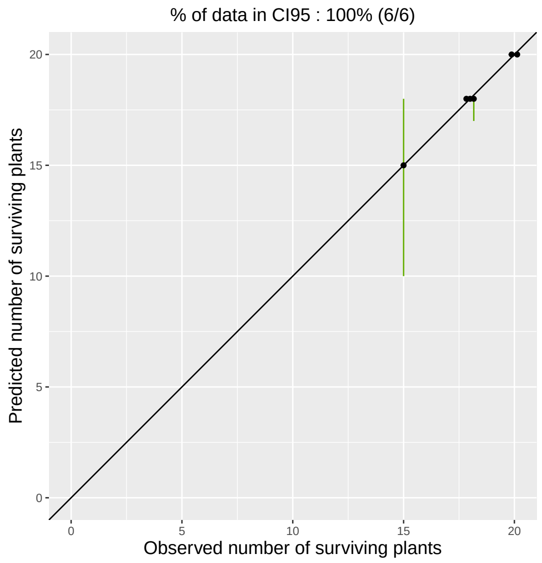

(b) Posterior predictive check (PPC)

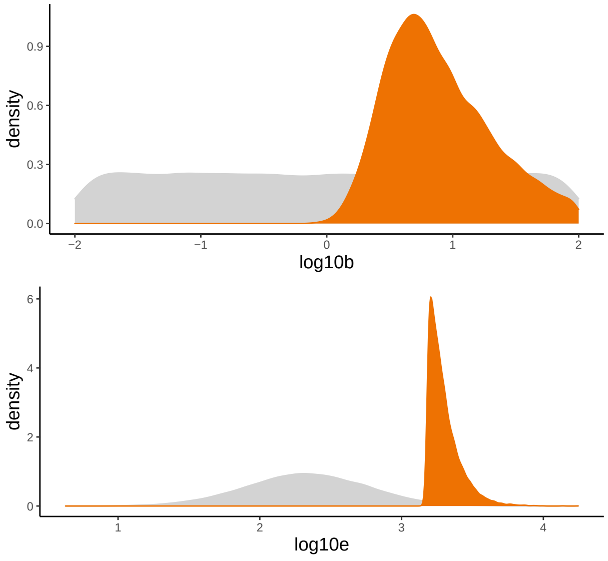

(c) Priors and posteriors

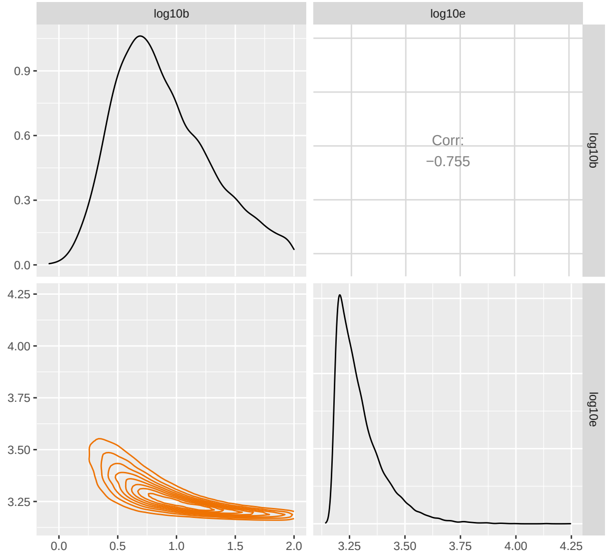

(d) Correlations between parameters

Figure 8: Dose-response curve (a), PPC (b), prior and posterior distributions (c) and correlations between parameters (d).

Data set: TRZAW_SE_survival

Table 9: Summary of parameter estimates (parameter d is set to 1) for TRZAW_SE_survival data set

Parameter	median	Q2.5	Q97.5
b	3.196	1.929	4.976
e	869.590	675.933	1143.524

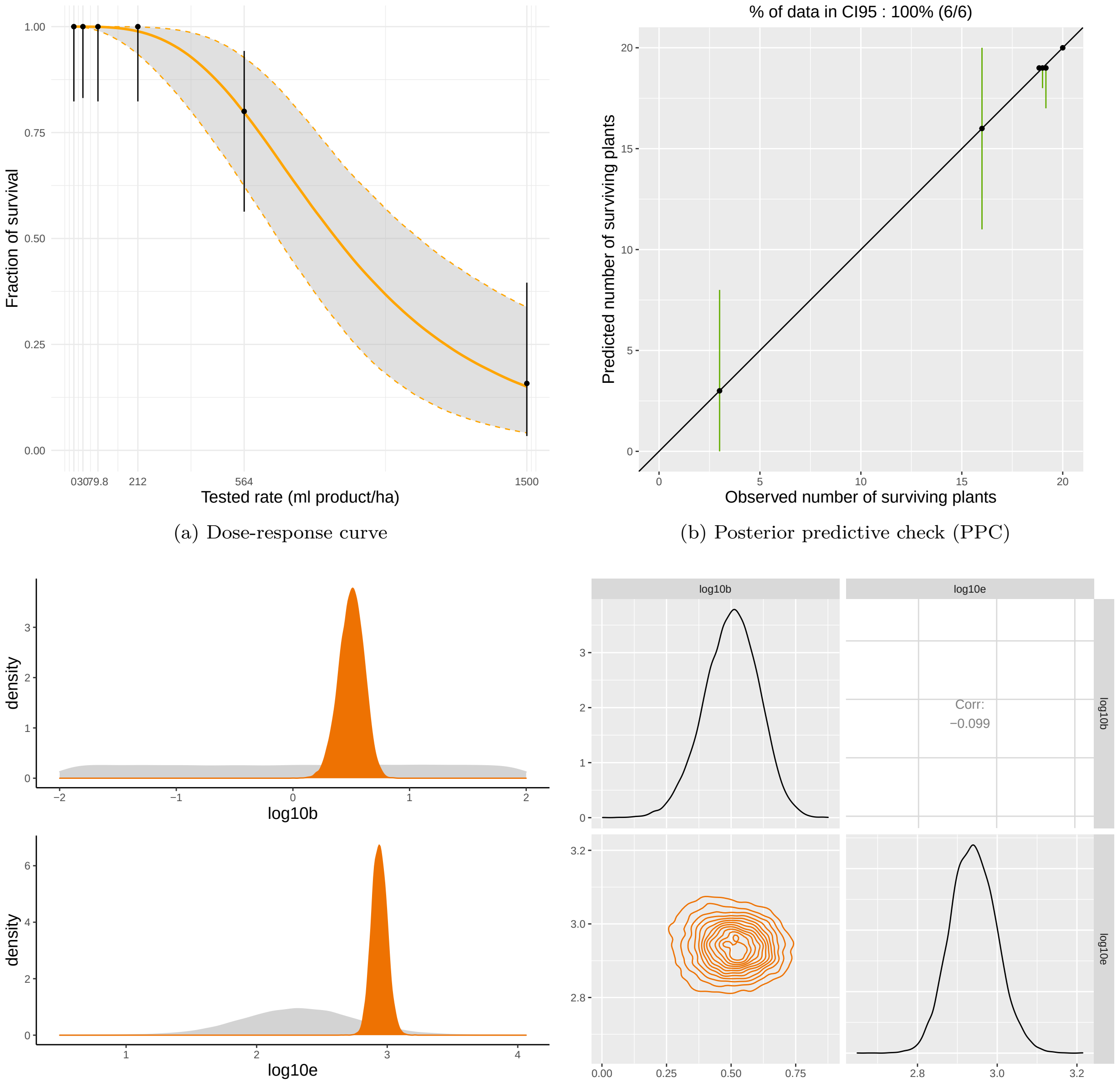

(a) Dose-response curve

(b) Posterior predictive check (PPC)

(c) Priors and posteriors

(d) Correlations between parameters

Figure 9: Dose-response curve (a), PPC (b), prior and posterior distributions (c) and correlations between parameters (d).

Data set: ZEAMA_SE_survival

Table 10: Summary of parameter estimates (parameter d is set to 1) for ZEAMA_SE_survival data set

Parameter	median	Q2.5	Q97.5
b	32.203	4.456	94.979
e	2303.701	1622.054	5941.655

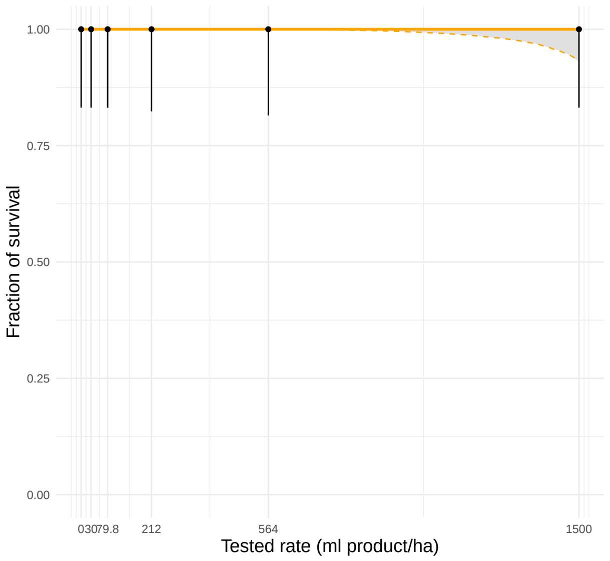

(a) Dose-response curve

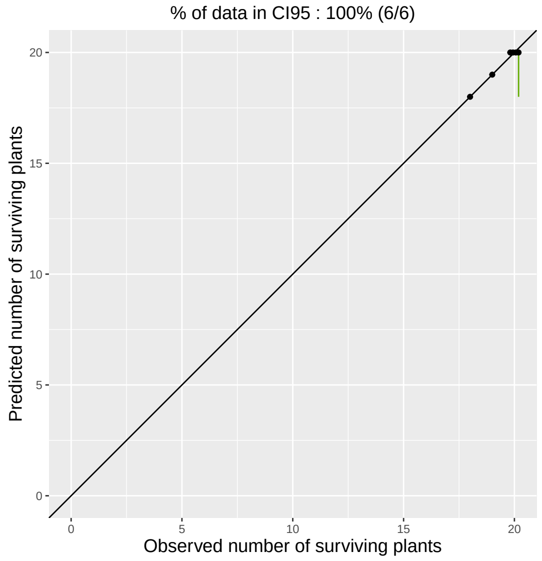

(b) Posterior predictive check (PPC)

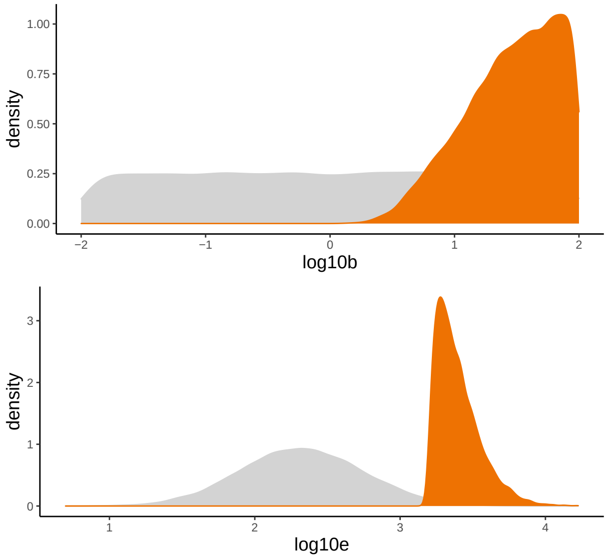

(c) Priors and posteriors

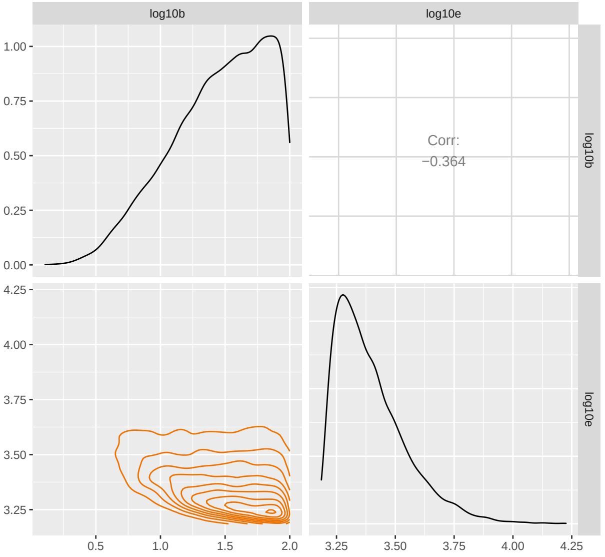

(d) Correlations between parameters

Figure 10: Dose-response curve (a), PPC (b), prior and posterior distributions (c) and correlations between parameters (d).
