# Supplementary material for: How to account for the uncertainty from standard toxicity tests in species sensitivity distributions: An example in non-target plants
Source: PLoS One. 2021 Jan 7;16(1):e0245071. doi: 10.1371/journal.pone.0245071 (PMC7790375; doi:10.1371/journal.pone.0245071)
Supplement: S1 Archive — It is a zip file containing seven folders (one folder per case study). Each folder contains five files report_xxx.pdf with detailed results of the dose-response analyses, one file corresponding to does-response analysis per endpoint. It also contains one file ER50_censoring.pdf for censored ER50 and one file SSD_analyses.pdf for results of SSD analyses. (ZIP) [file pone.0245071.s004.zip › S1_archive/Study3/report_SE_weight.pdf]

Dose-response analysis

Study 3

Seedling Emergence test - shoot dry SE_weight endpoint

25 June 2020

Contact: sandrine.charles@univ-lyon1.fr

This is a report which provides results on all performed dose-response analyses for the shoot dry SE_weight endpoint of the Seedling Emergence test for study 3.

Contents

Data set: ALLCE_SE_weight . . . 2
Data set: BEAVA_SE_weight . . . 3
Data set: BRSNW_SE_weight . . . 4
Data set: CUMSA_SE_weight . . . 5
Data set: FAGES_SE_weight . . . 6
Data set: GLXMA_SE_weight . . . 7
Data set: LOLPE_SE_weight . . . 8
Data set: LYPES_SE_weight . . . 9
Data set: TRZAW_SE_weight . . . 10
Data set: ZEAMA_SE_weight . . . 11

Data set: ALLCE_SE_weight

Table 1: Summary of parameter estimates for ALLCE_SE_weight data set

Parameter	median	Q2.5	Q97.5
b	8.798	1.261	85.575
d	0.032	0.029	0.036
e	1996.753	1532.316	4942.222
sigma	0.008	0.006	0.011

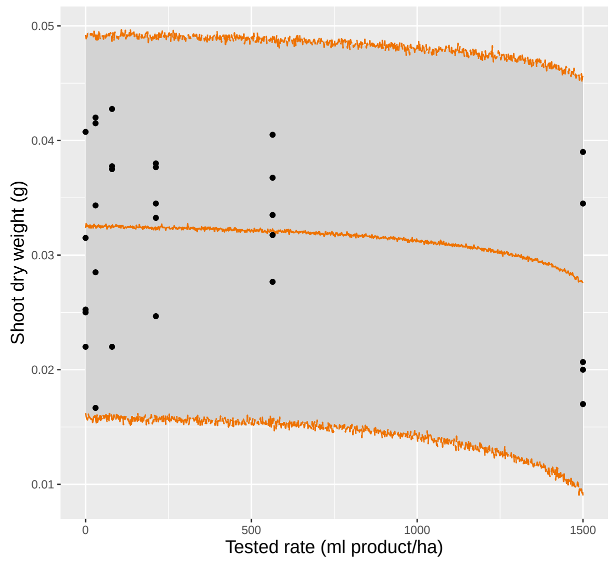

(a) Dose-response curve

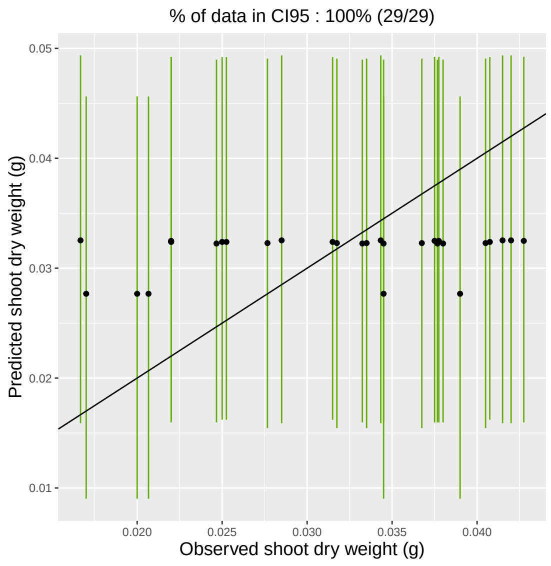

(b) Posterior predictive check (PPC)

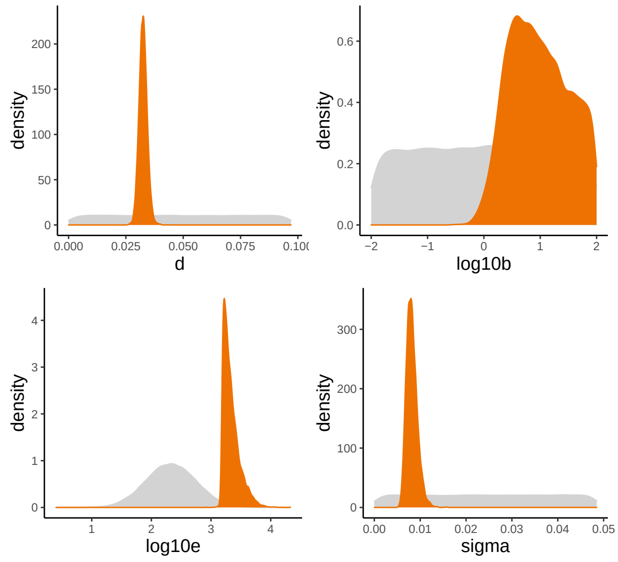

(c) Priors and posteriors

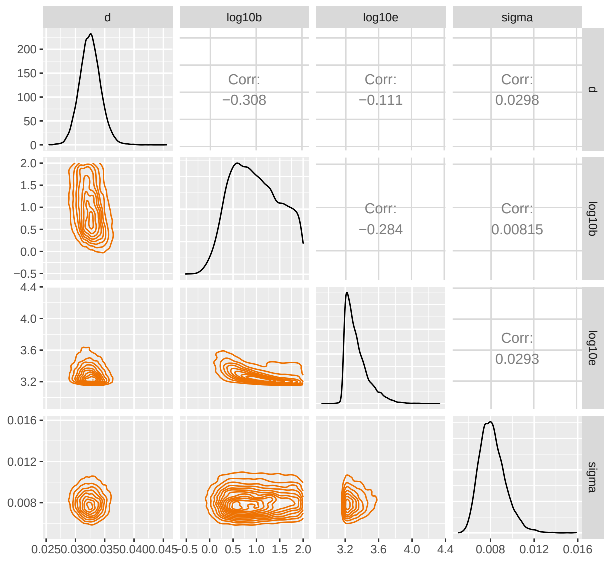

(d) Correlations between parameters

Figure 1: Dose-response curve (a), PPC (b), prior and posterior distributions (c) and correlations between parameters (d).

Data set: BEAVA_SE_weight

Table 2: Summary of parameter estimates for BEAVA_SE_weight data set

Parameter	median	Q2.5	Q97.5
b	8.275	1.597	82.410
d	1.247	1.181	1.312
e	2034.080	1544.751	4912.110
sigma	0.221	0.186	0.270

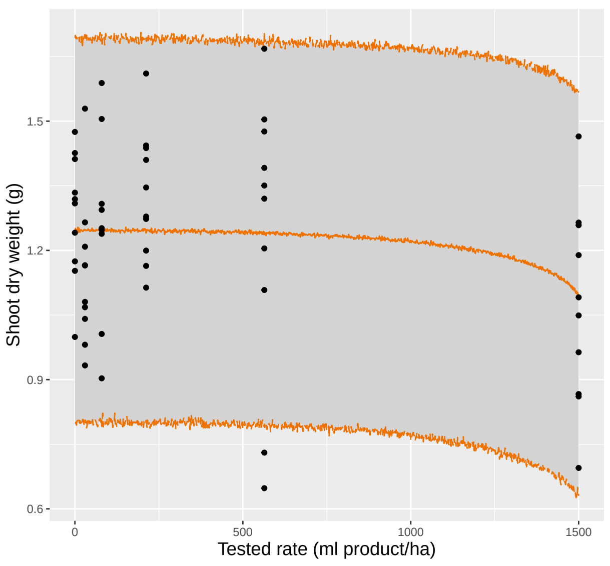

(a) Dose-response curve

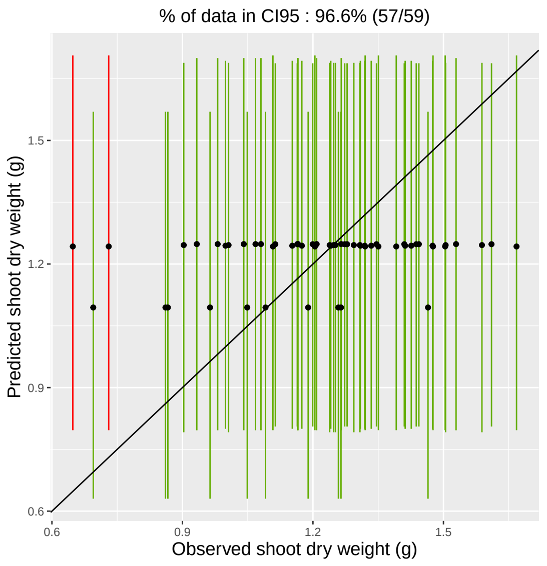

(b) Posterior predictive check (PPC)

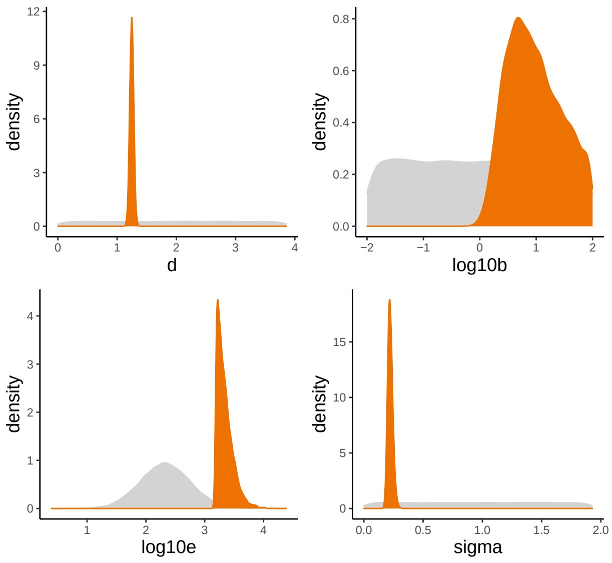

(c) Priors and posteriors

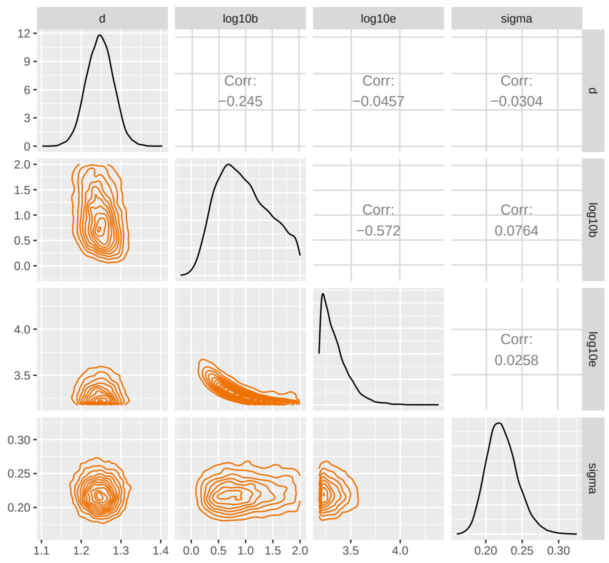

(d) Correlations between parameters

Figure 2: Dose-response curve (a), PPC (b), prior and posterior distributions (c) and correlations between parameters (d).

Data set: BRSNW_SE_weight

Table 3: Summary of parameter estimates for BRSNW_SE_weight data set

Parameter	median	Q2.5	Q97.5
b	2.850	1.699	15.829
d	1.871	1.761	1.989
e	839.927	606.825	1093.059
sigma	0.333	0.276	0.412

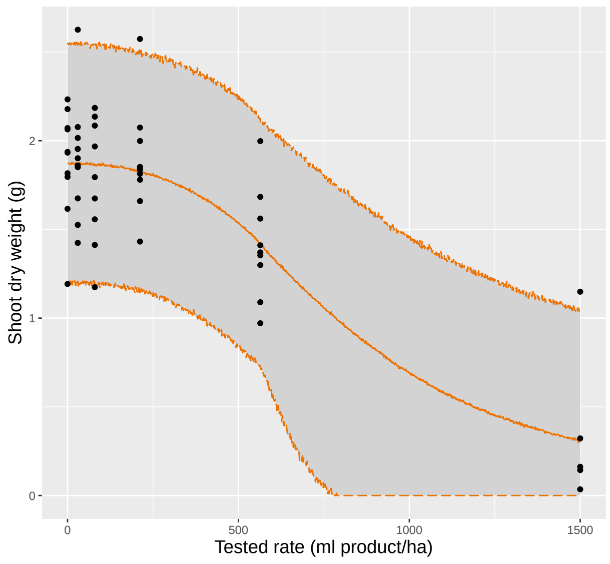

(a) Dose-response curve

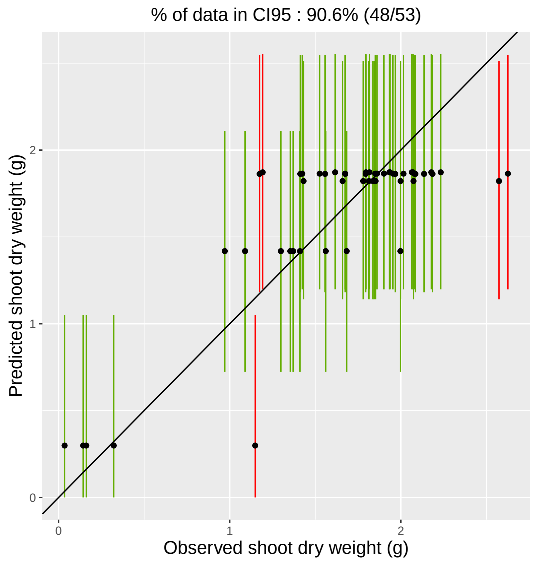

(b) Posterior predictive check (PPC)

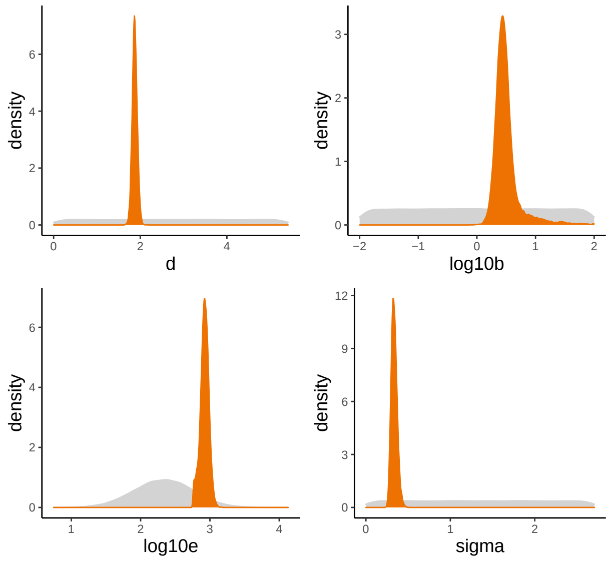

(c) Priors and posteriors

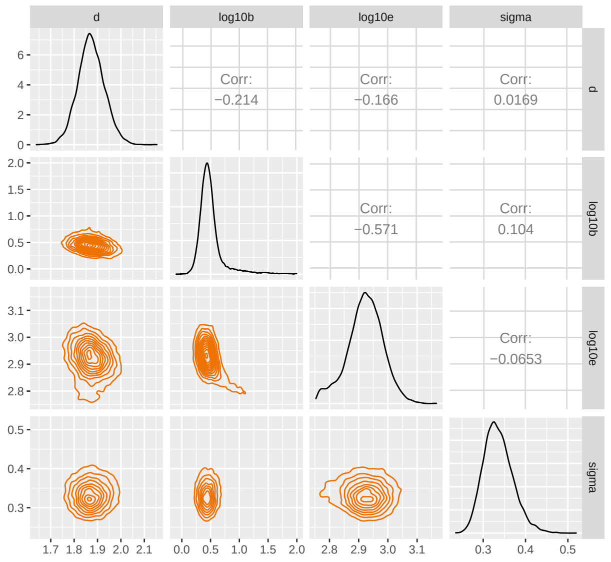

(d) Correlations between parameters

Figure 3: Dose-response curve (a), PPC (b), prior and posterior distributions (c) and correlations between parameters (d).

Data set: CUMSA_SE_weight

Table 4: Summary of parameter estimates for CUMSA_SE_weight data set

Parameter	median	Q2.5	Q97.5
b	2.608	1.850	4.451
d	3.713	3.490	3.942
e	284.863	238.887	337.332
sigma	0.540	0.446	0.673

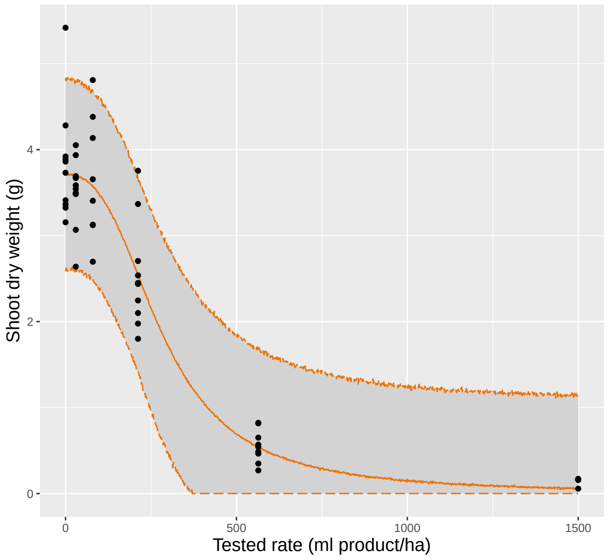

(a) Dose-response curve

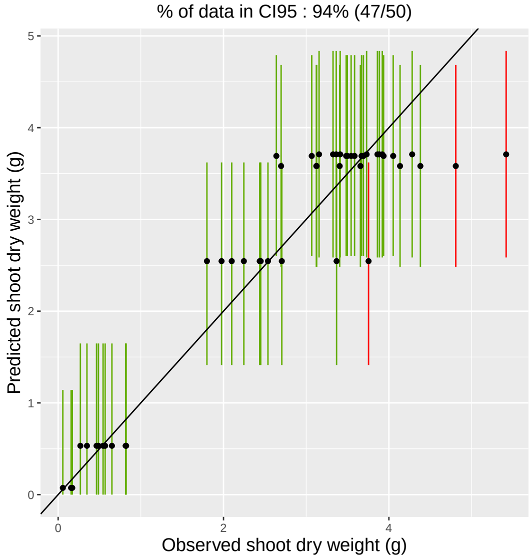

(b) Posterior predictive check (PPC)

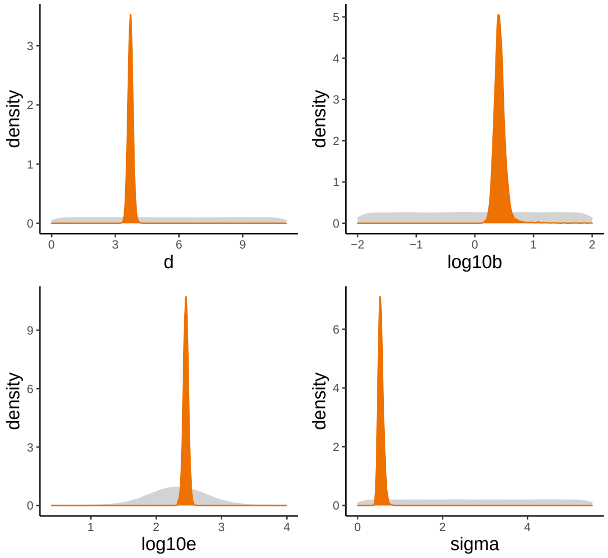

(c) Priors and posteriors

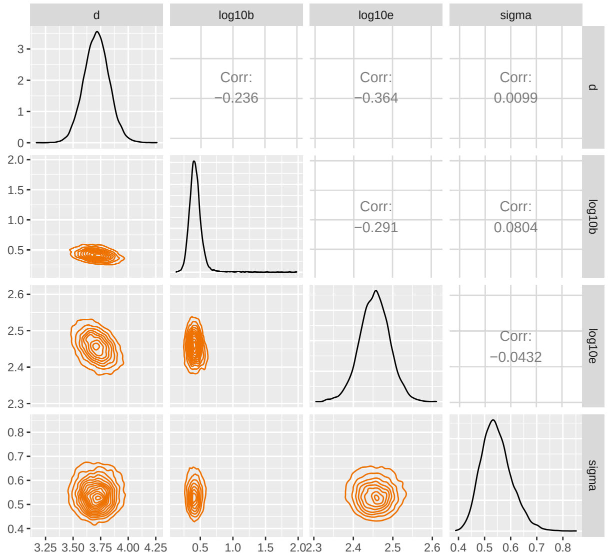

(d) Correlations between parameters

Figure 4: Dose-response curve (a), PPC (b), prior and posterior distributions (c) and correlations between parameters (d).

Data set: FAGES_SE_weight

Table 5: Summary of parameter estimates for FAGES_SE_weight data set

Parameter	median	Q2.5	Q97.5
b	1.146	0.498	3.124
d	1.727	1.541	1.994
e	1248.414	730.313	2214.897
sigma	0.435	0.365	0.533

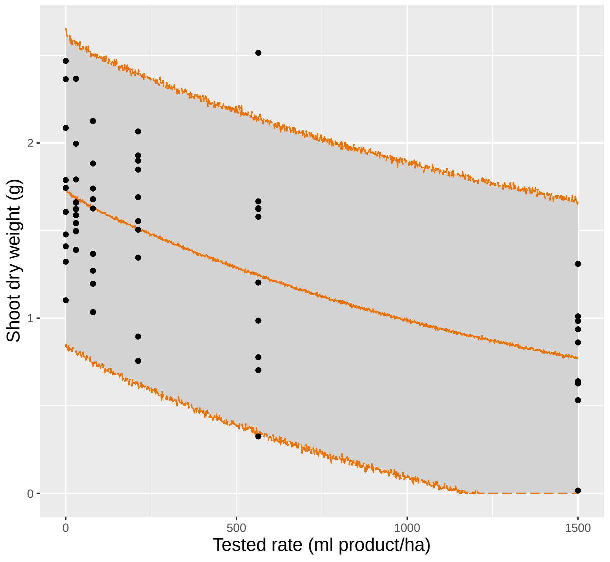

(a) Dose-response curve

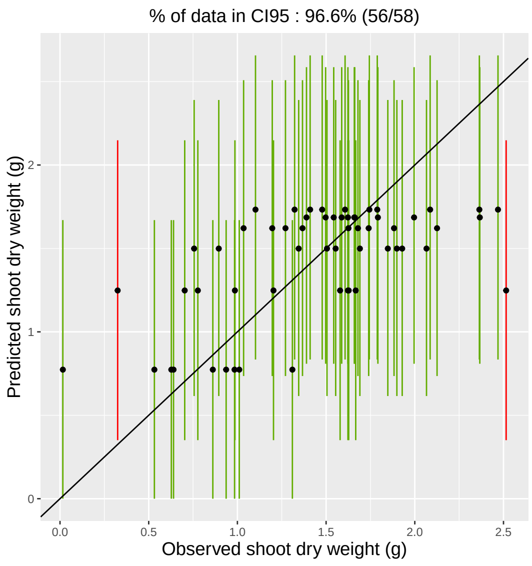

(b) Posterior predictive check (PPC)

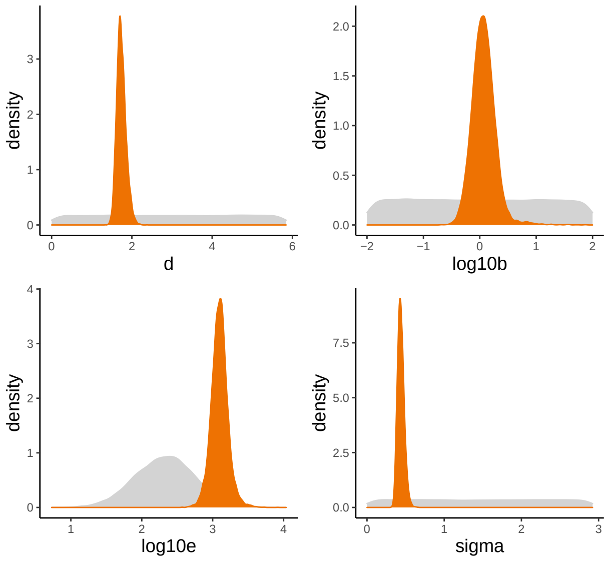

(c) Priors and posteriors

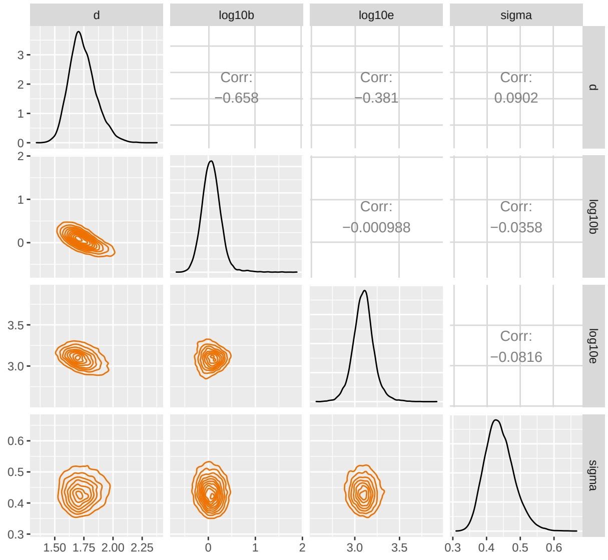

(d) Correlations between parameters

Figure 5: Dose-response curve (a), PPC (b), prior and posterior distributions (c) and correlations between parameters (d).

Data set: GLXMA_SE_weight

Table 6: Summary of parameter estimates for GLXMA_SE_weight data set

Parameter	median	Q2.5	Q97.5
b	5.882	1.844	57.119
d	1.443	1.378	1.509
e	1742.039	1523.044	2651.235
sigma	0.225	0.188	0.274

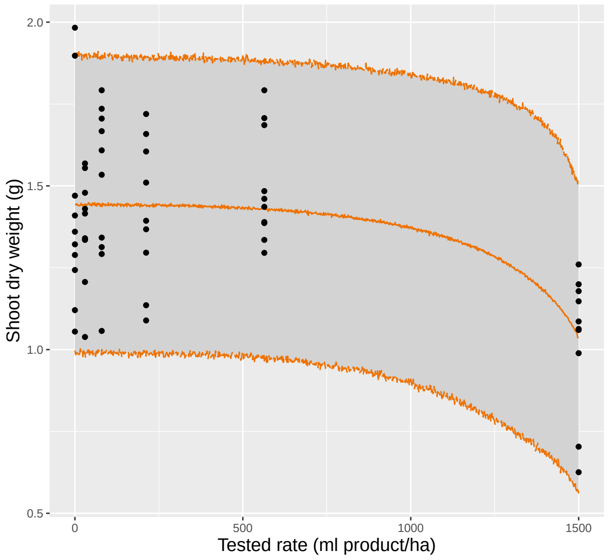

(a) Dose-response curve

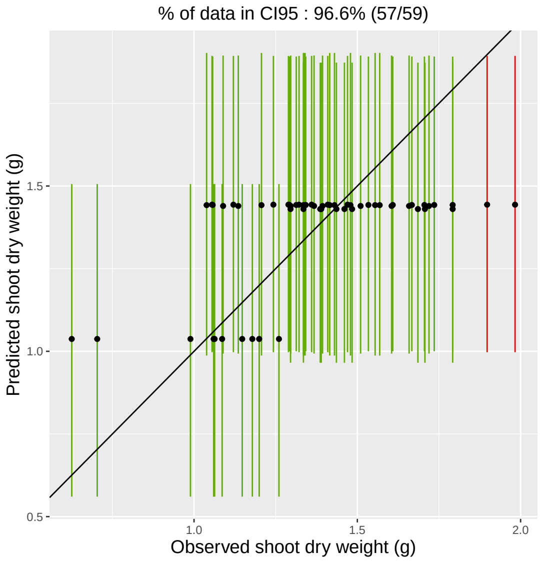

(b) Posterior predictive check (PPC)

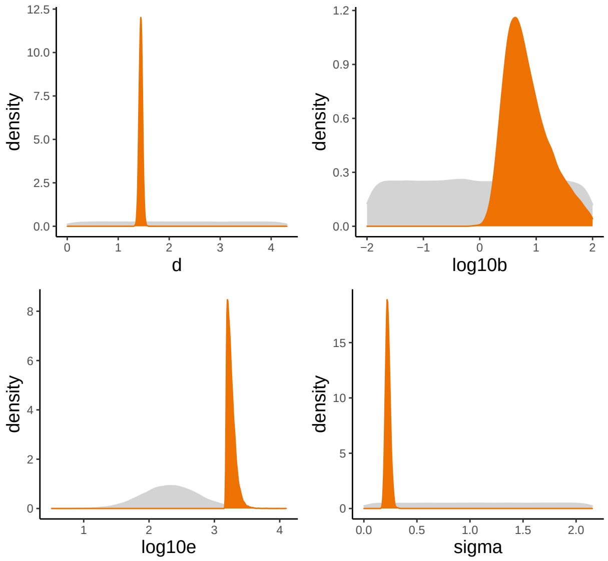

(c) Priors and posteriors

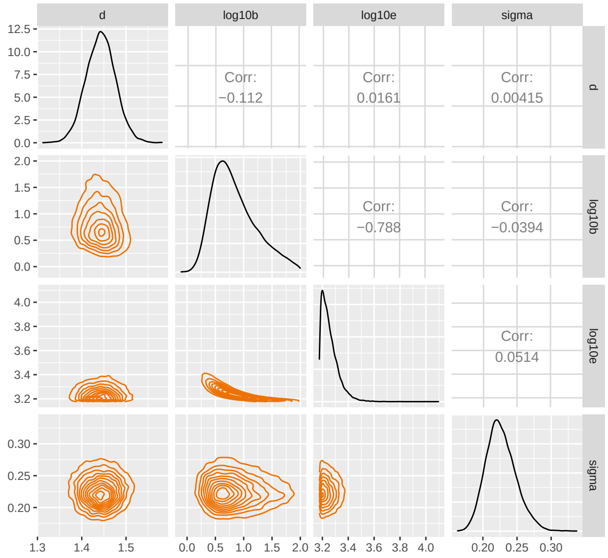

(d) Correlations between parameters

Figure 6: Dose-response curve (a), PPC (b), prior and posterior distributions (c) and correlations between parameters (d).

Data set: LOLPE_SE_weight

Table 7: Summary of parameter estimates for LOLPE_SE_weight data set

Parameter	median	Q2.5	Q97.5
b	3.865	1.016	41.831
d	0.119	0.107	0.134
e	1420.284	959.080	1924.471
sigma	0.027	0.021	0.038

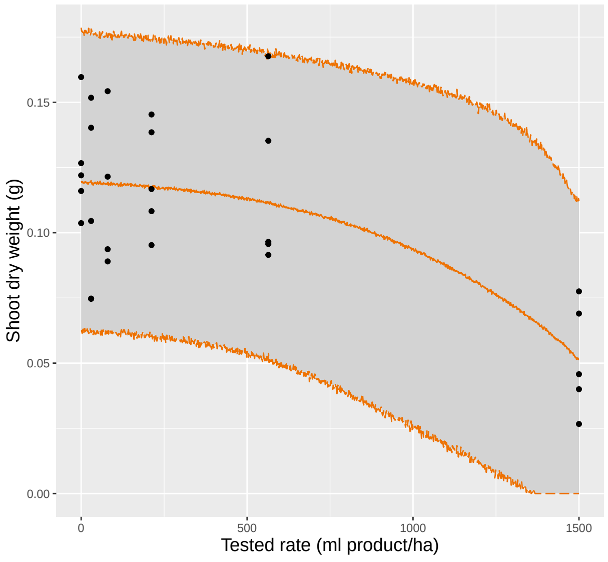

(a) Dose-response curve

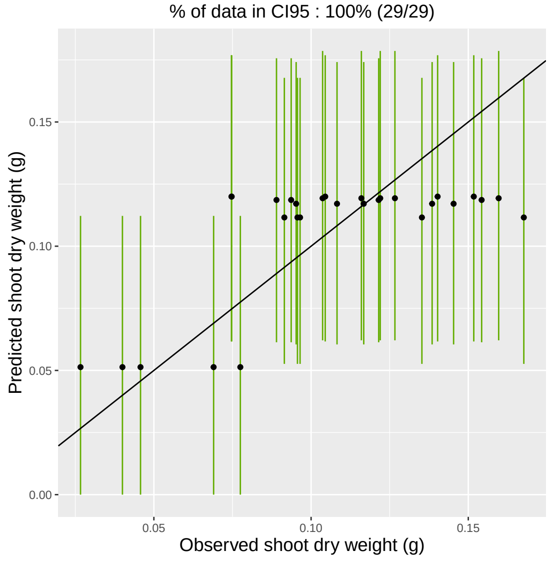

(b) Posterior predictive check (PPC)

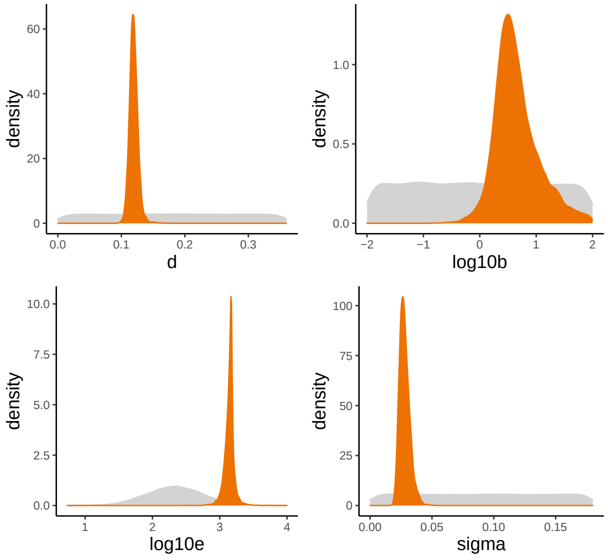

(c) Priors and posteriors

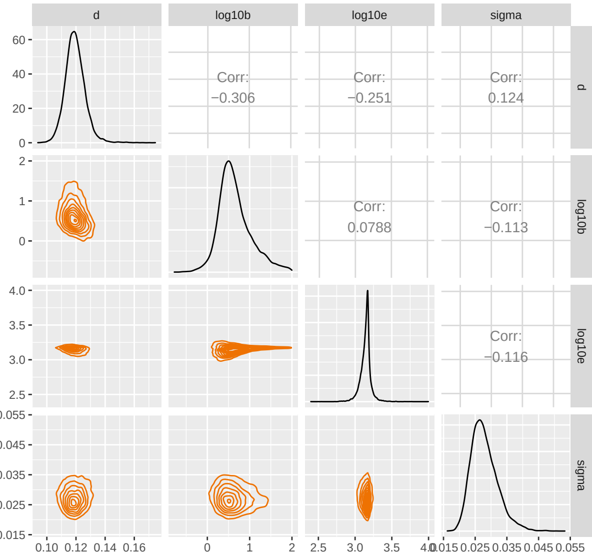

(d) Correlations between parameters

Figure 7: Dose-response curve (a), PPC (b), prior and posterior distributions (c) and correlations between parameters (d).

Data set: LYPES_SE_weight

Table 8: Summary of parameter estimates for LYPES_SE_weight data set

Parameter	median	Q2.5	Q97.5
b	4.450	2.794	46.781
d	1.332	1.258	1.404
e	772.613	583.507	970.832
sigma	0.219	0.183	0.269

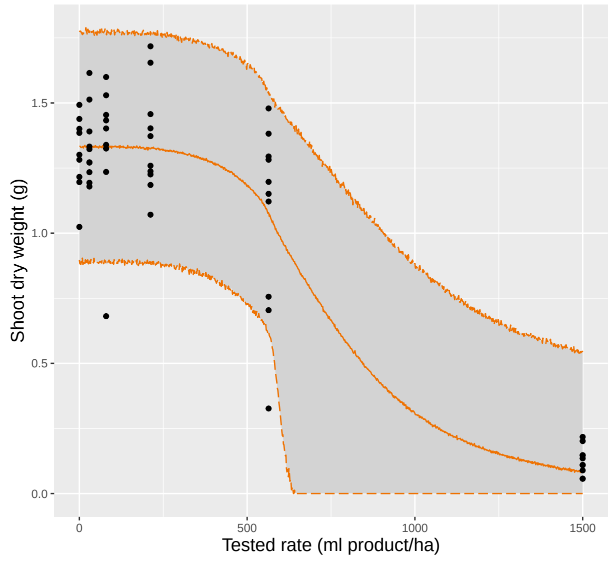

(a) Dose-response curve

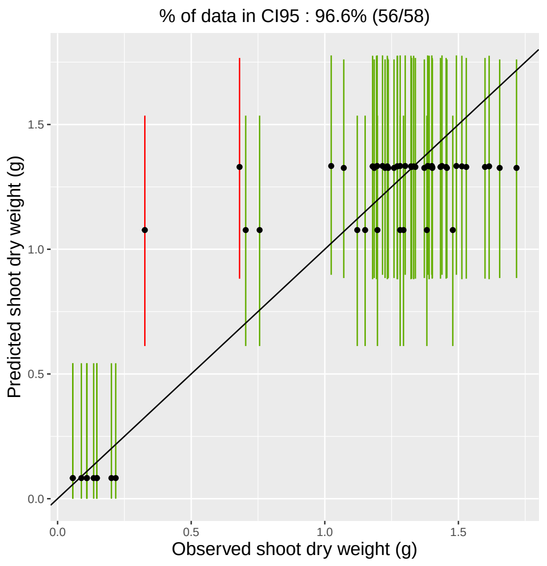

(b) Posterior predictive check (PPC)

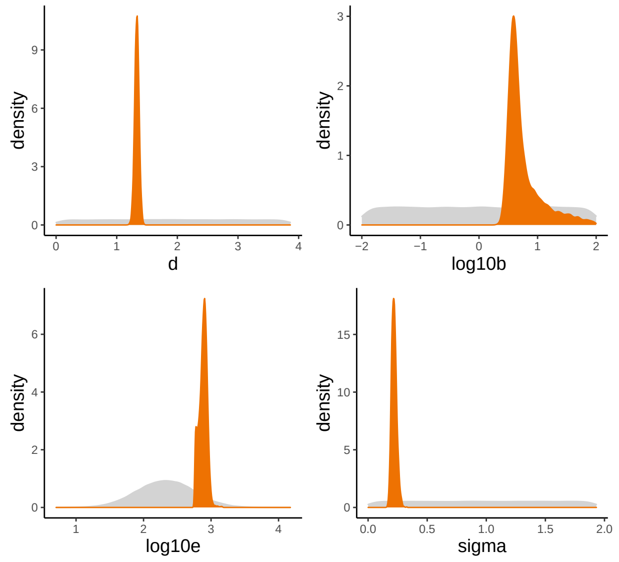

(c) Priors and posteriors

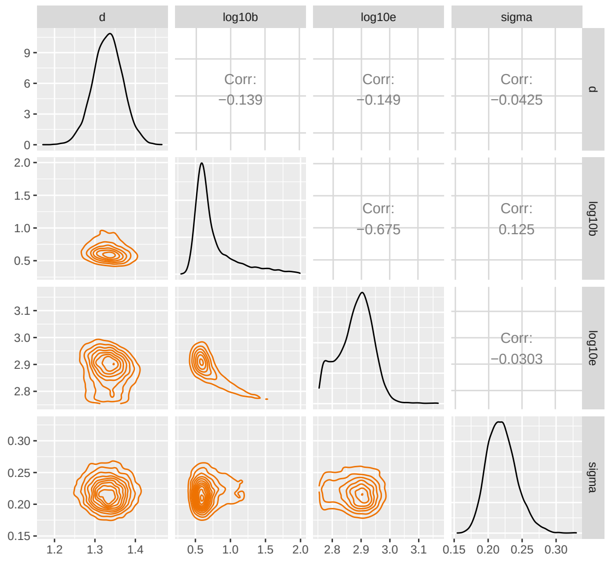

(d) Correlations between parameters

Figure 8: Dose-response curve (a), PPC (b), prior and posterior distributions (c) and correlations between parameters (d).

Data set: TRZAW_SE_weight

Table 9: Summary of parameter estimates for TRZAW_SE_weight data set

Parameter	median	Q2.5	Q97.5
b	3.944	2.421	37.643
d	0.442	0.421	0.463
e	213.878	198.568	234.769
sigma	0.036	0.028	0.049

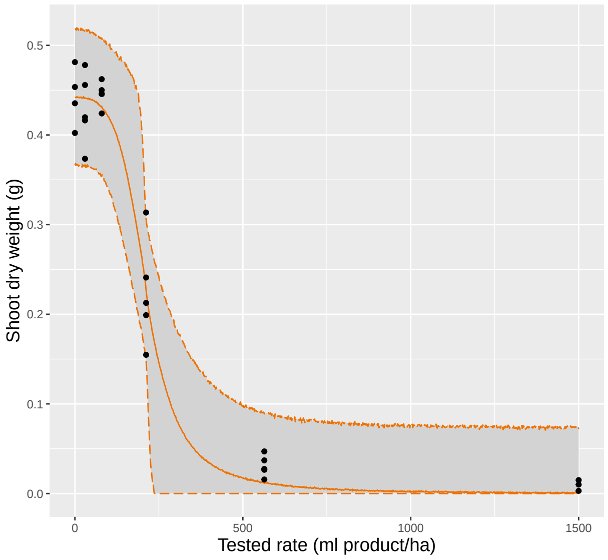

(a) Dose-response curve

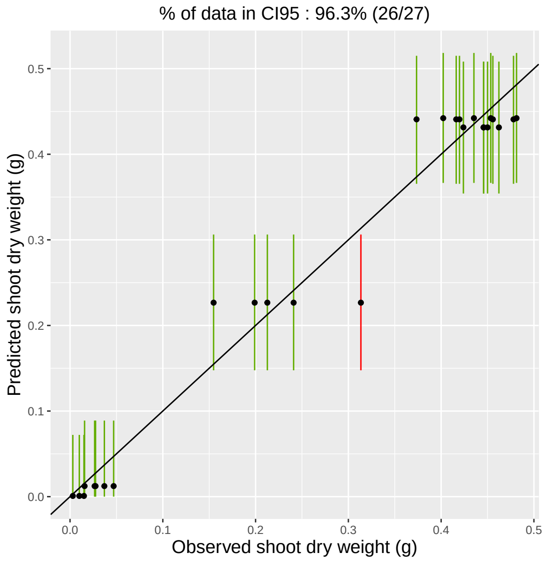

(b) Posterior predictive check (PPC)

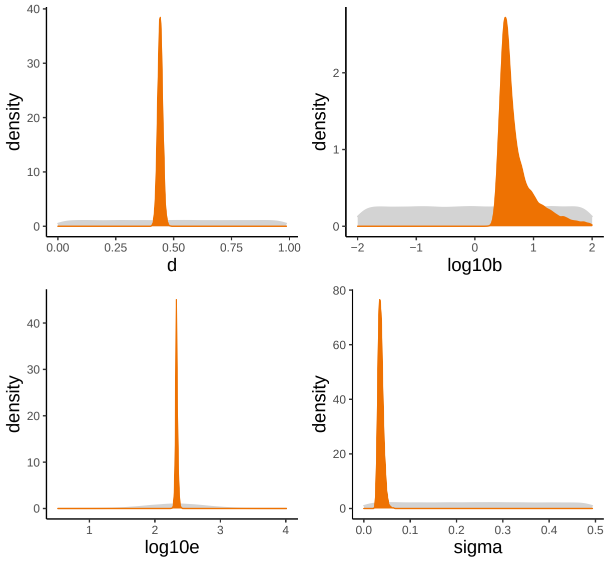

(c) Priors and posteriors

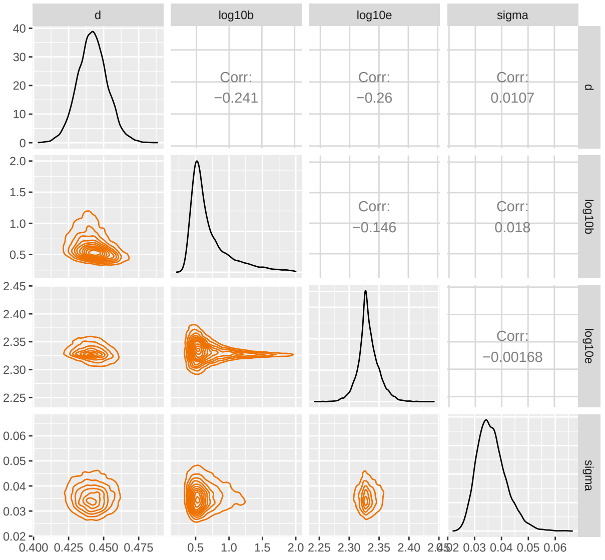

(d) Correlations between parameters

Figure 9: Dose-response curve (a), PPC (b), prior and posterior distributions (c) and correlations between parameters (d).

Data set: ZEAMA_SE_weight

Table 10: Summary of parameter estimates for ZEAMA_SE_weight data set

Parameter	median	Q2.5	Q97.5
b	19.384	2.734	91.540
d	4.123	3.953	4.298
e	2149.669	1573.526	5601.577
sigma	0.621	0.524	0.755

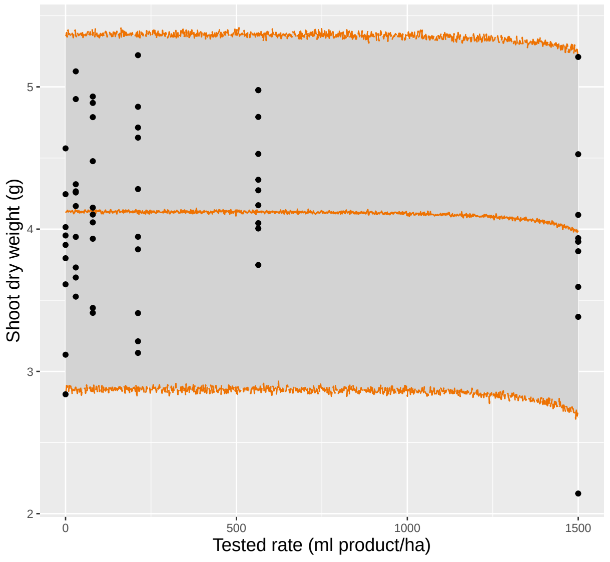

(a) Dose-response curve

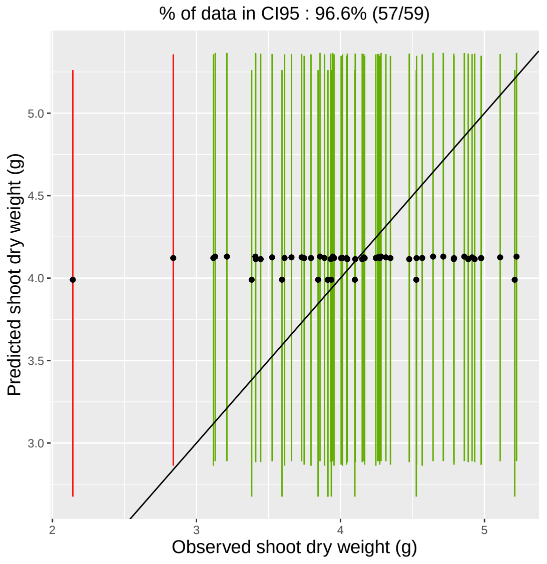

(b) Posterior predictive check (PPC)

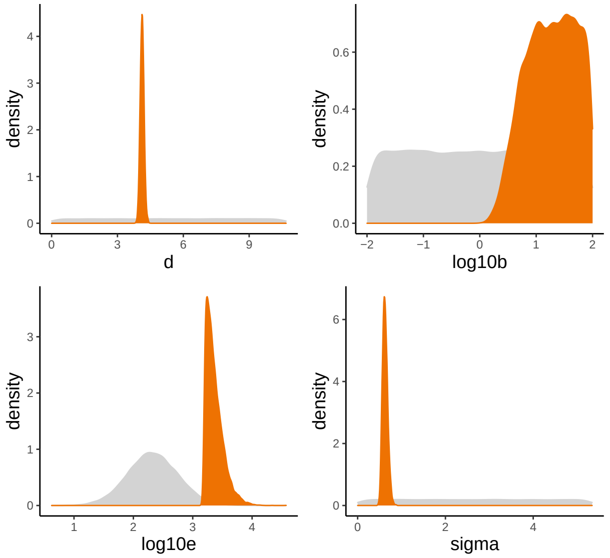

(c) Priors and posteriors

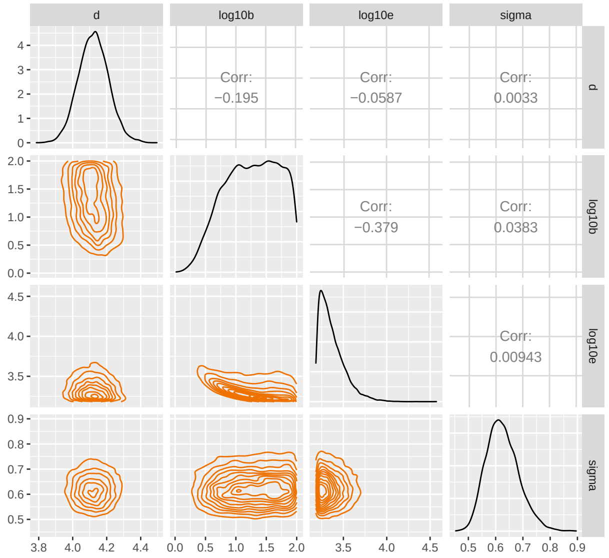

(d) Correlations between parameters

Figure 10: Dose-response curve (a), PPC (b), prior and posterior distributions (c) and correlations between parameters (d).
