# Supplementary material for: How to account for the uncertainty from standard toxicity tests in species sensitivity distributions: An example in non-target plants
Source: PLoS One. 2021 Jan 7;16(1):e0245071. doi: 10.1371/journal.pone.0245071 (PMC7790375; doi:10.1371/journal.pone.0245071)
Supplement: S1 Archive — It is a zip file containing seven folders (one folder per case study). Each folder contains five files report_xxx.pdf with detailed results of the dose-response analyses, one file corresponding to does-response analysis per endpoint. It also contains one file ER50_censoring.pdf for censored ER50 and one file SSD_analyses.pdf for results of SSD analyses. (ZIP) [file pone.0245071.s004.zip › S1_archive/Study3/report_VV_survival.pdf]

Dose-response analyses

Study 3

Vegetative Vigour test - survival endpoint

25 June 2020

Contact: sandrine.charles@univ-lyon1.fr

This is a report which provides results on all performed dose-response analyses for the survival endpoint of the Vegetative Vigour test for study 3.

Contents

Data set: ALLCE_VV_survival 2
Data set: BEAVA_VV_survival 3
Data set: BRSNW_VV_survival 4
Data set: CUMSA_VV_survival 5
Data set: FAGES_VV_survival 6
Data set: GLXMA_VV_survival 7
Data set: LOLPE_VV_survival 8
Data set: LYPES_VV_survival 9
Data set: TRZAW_VV_survival 10
Data set: ZEAMA_VV_survival 11

Data set: ALLCE_VV_survival

Table 1: Summary of parameter estimates (parameter d is set to 1) for ALLCE_VV_survival data set

Parameter	median	Q2.5	Q97.5
b	32.325	4.386	95.058
e	2307.761	1616.726	6305.021

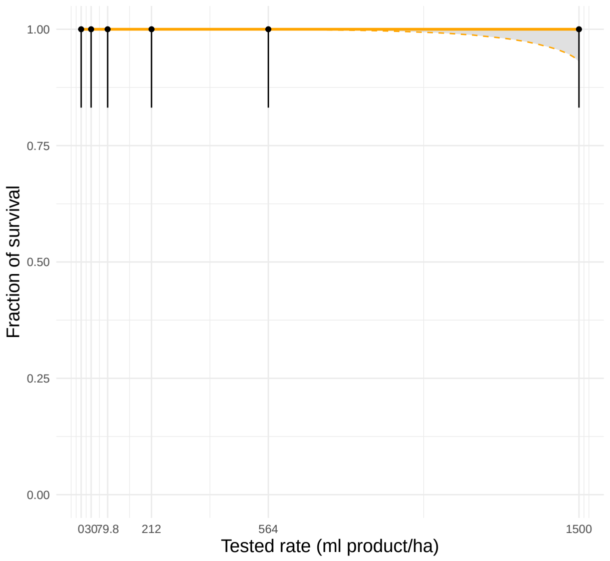

(a) Dose-response curve

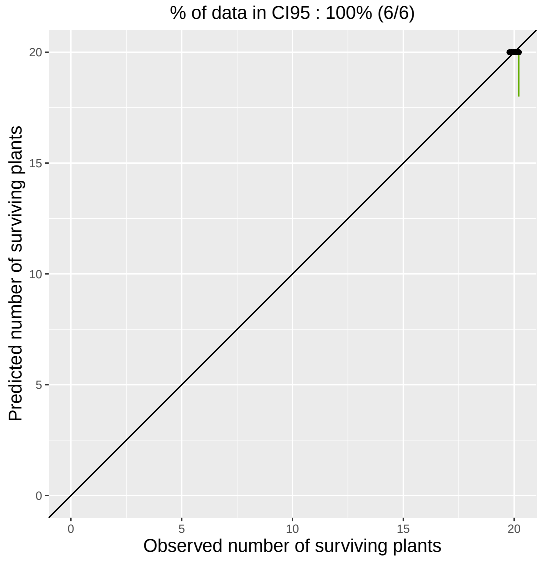

(b) Posterior predictive check (PPC)

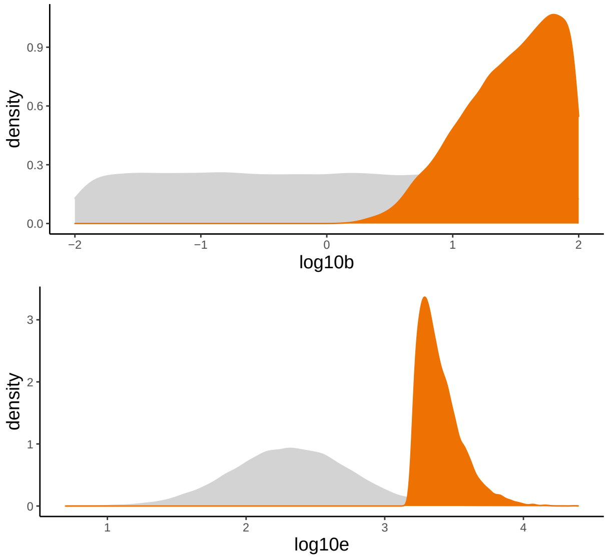

(c) Priors and posteriors

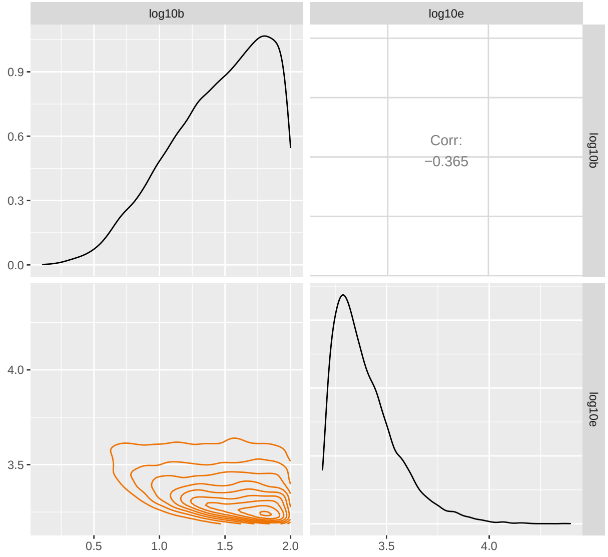

(d) Correlations between parameters

Figure 1: Dose-response curve (a), PPC (b), prior and posterior distributions (c) and correlations between parameters (d).

Data set: BEAVA_VV_survival

Table 2: Summary of parameter estimates (parameter d is set to 1) for BEAVA_VV_survival data set

Parameter	median	Q2.5	Q97.5
b	31.003	4.142	94.796
e	2328.184	1626.187	6317.185

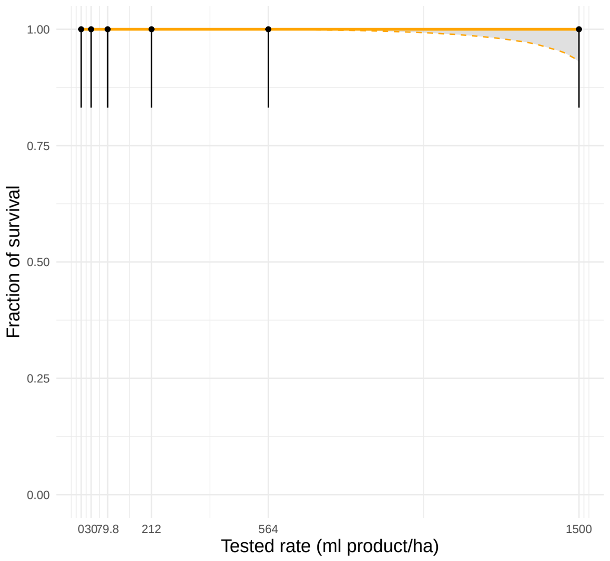

(a) Dose-response curve

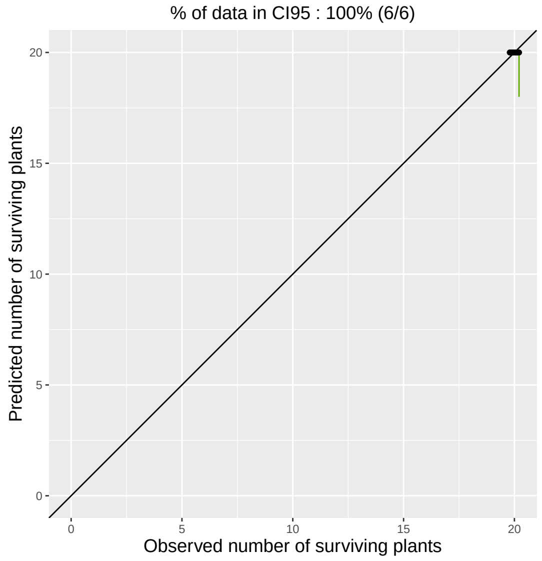

(b) Posterior predictive check (PPC)

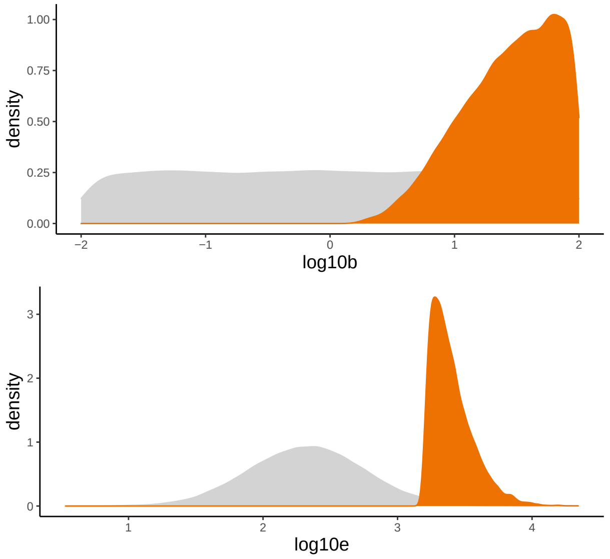

(c) Priors and posteriors

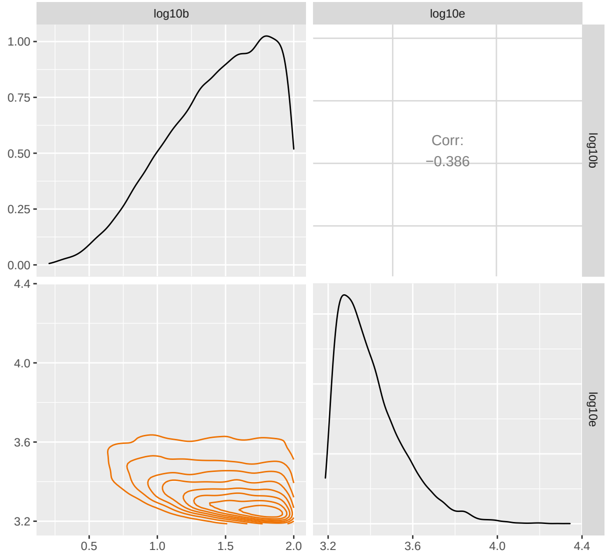

(d) Correlations between parameters

Figure 2: Dose-response curve (a), PPC (b), prior and posterior distributions (c) and correlations between parameters (d).

Data set: BRSNW_VV_survival

Table 3: Summary of parameter estimates (parameter d is set to 1) for BRSNW_VV_survival data set

Parameter	median	Q2.5	Q97.5
b	31.808	4.289	95.078
e	2307.544	1619.250	6248.358

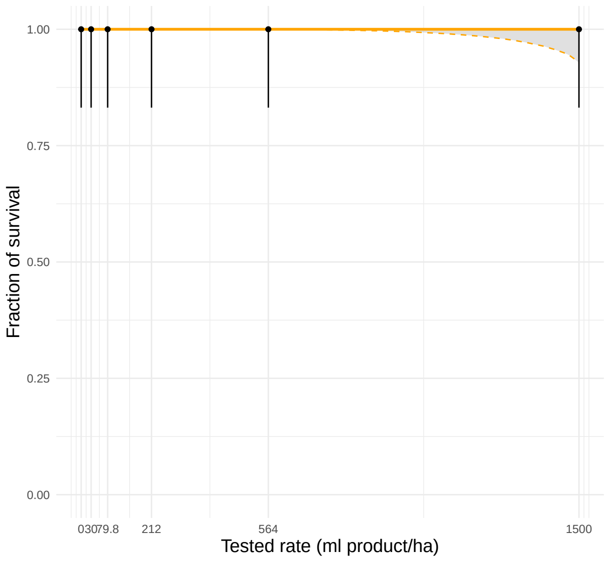

(a) Dose-response curve

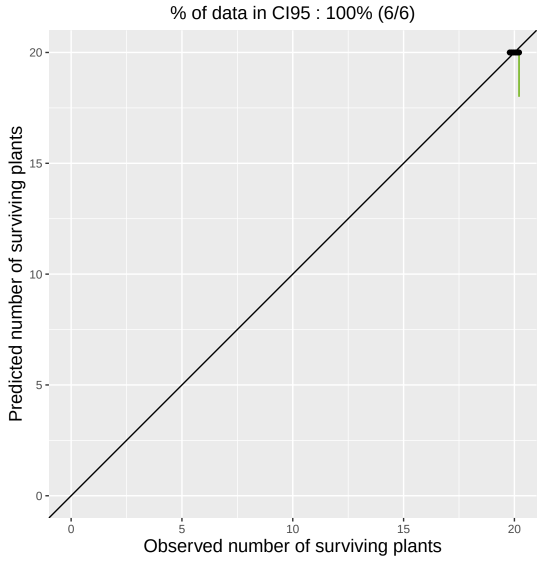

(b) Posterior predictive check (PPC)

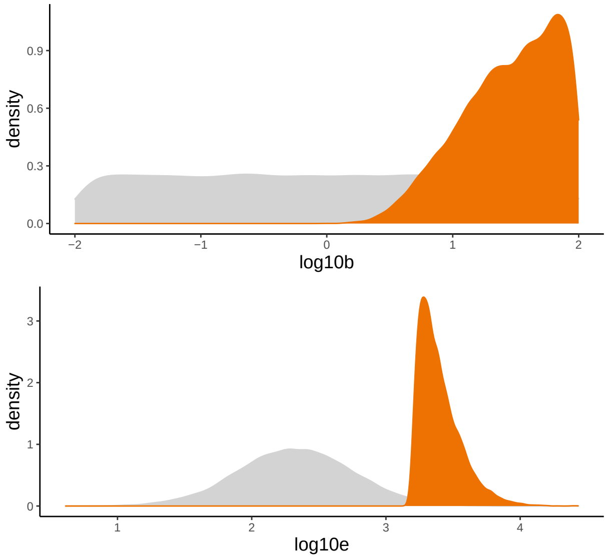

(c) Priors and posteriors

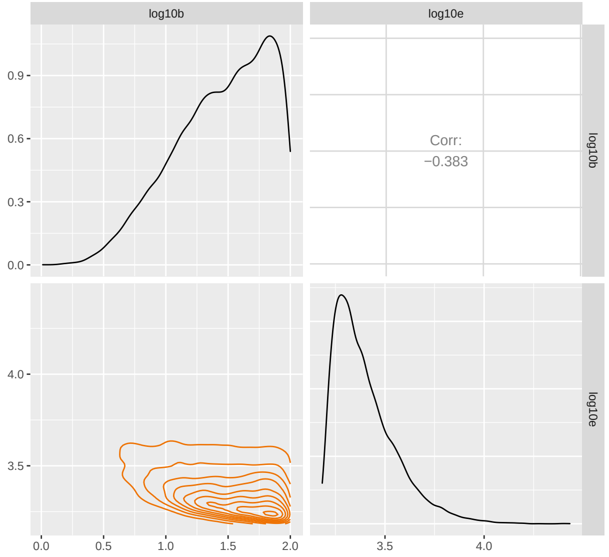

(d) Correlations between parameters

Figure 3: Dose-response curve (a), PPC (b), prior and posterior distributions (c) and correlations between parameters (d).

Data set: CUMSA_VV_survival

Table 4: Summary of parameter estimates (parameter d is set to 1) for CUMSA_VV_survival data set

Parameter	median	Q2.5	Q97.5
b	3.682	2.036	6.416
e	1190.140	936.234	1509.734

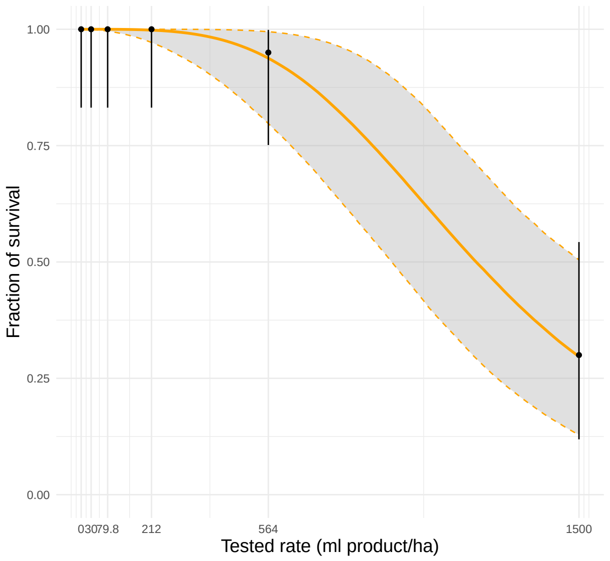

(a) Dose-response curve

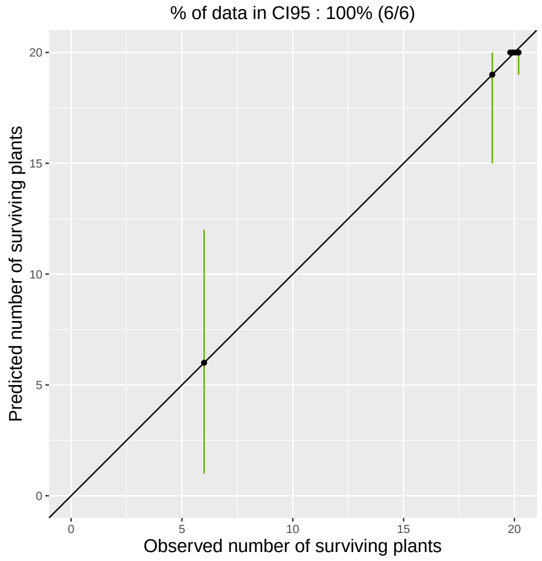

(b) Posterior predictive check (PPC)

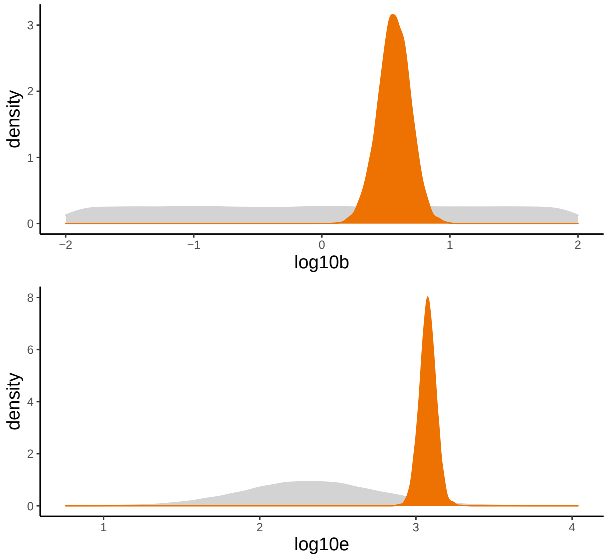

(c) Priors and posteriors

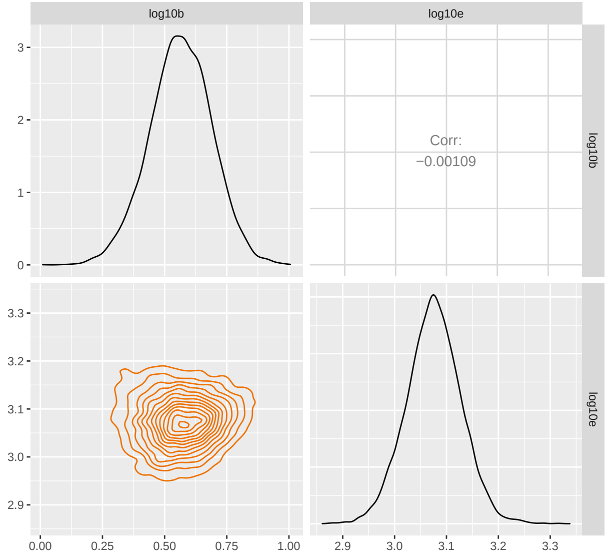

(d) Correlations between parameters

Figure 4: Dose-response curve (a), PPC (b), prior and posterior distributions (c) and correlations between parameters (d).

Data set: FAGES_VV_survival

Table 5: Summary of parameter estimates (parameter d is set to 1) for FAGES_VV_survival data set

Parameter	median	Q2.5	Q97.5
b	2.319	1.373	3.669
e	1094.095	816.159	1583.717

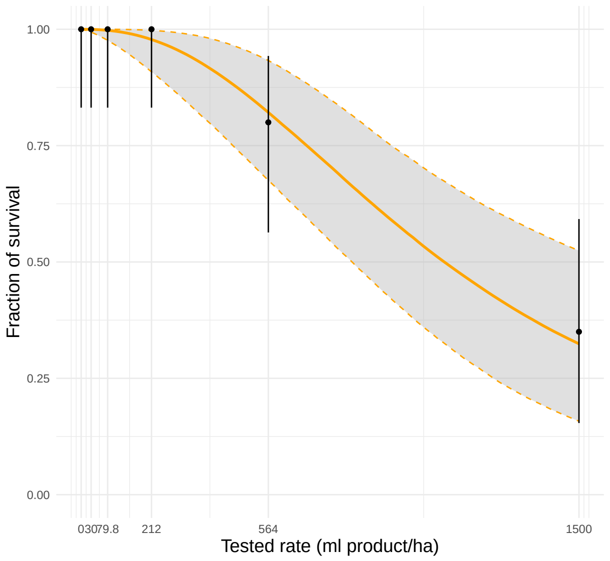

(a) Dose-response curve

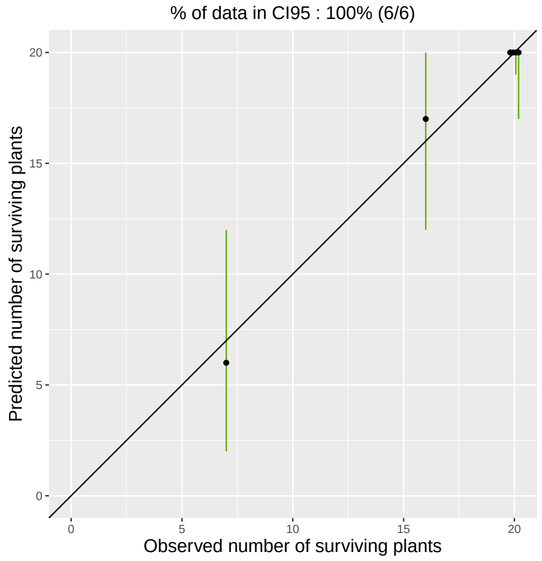

(b) Posterior predictive check (PPC)

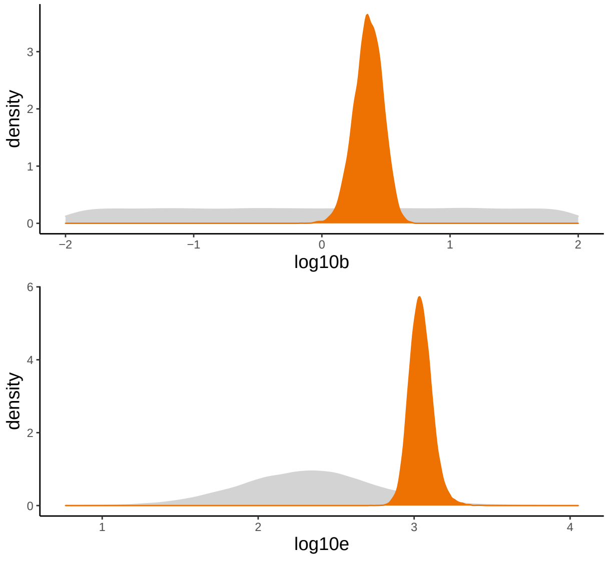

(c) Priors and posteriors

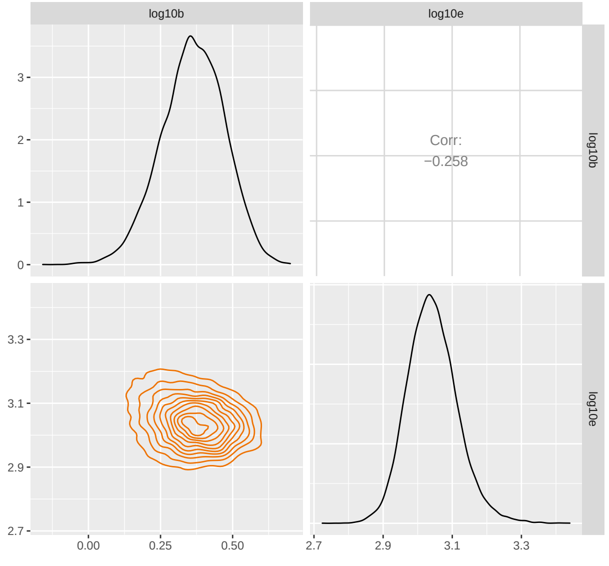

(d) Correlations between parameters

Figure 5: Dose-response curve (a), PPC (b), prior and posterior distributions (c) and correlations between parameters (d).

Data set: GLXMA_VV_survival

Table 6: Summary of parameter estimates (parameter d is set to 1) for GLXMA_VV_survival data set

Parameter	median	Q2.5	Q97.5
b	32.287	4.462	94.994
e	2294.930	1620.041	6148.954

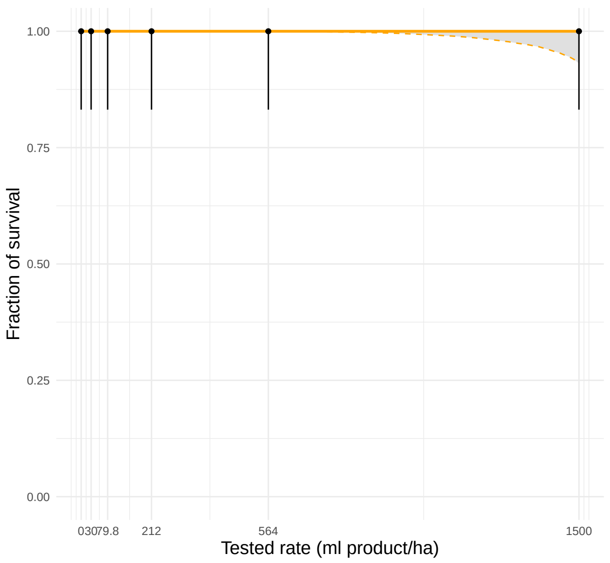

(a) Dose-response curve

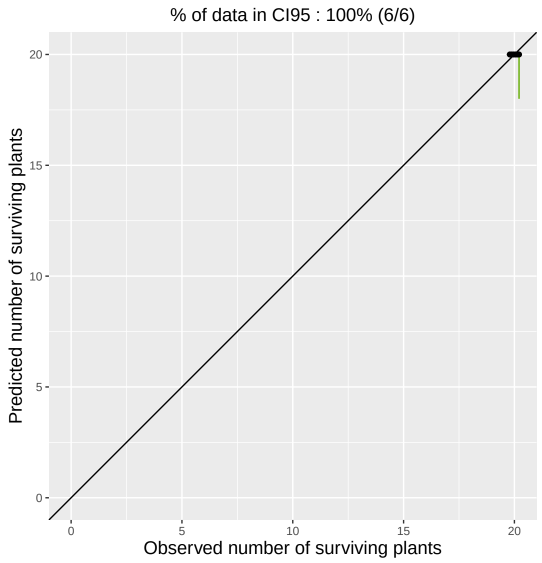

(b) Posterior predictive check (PPC)

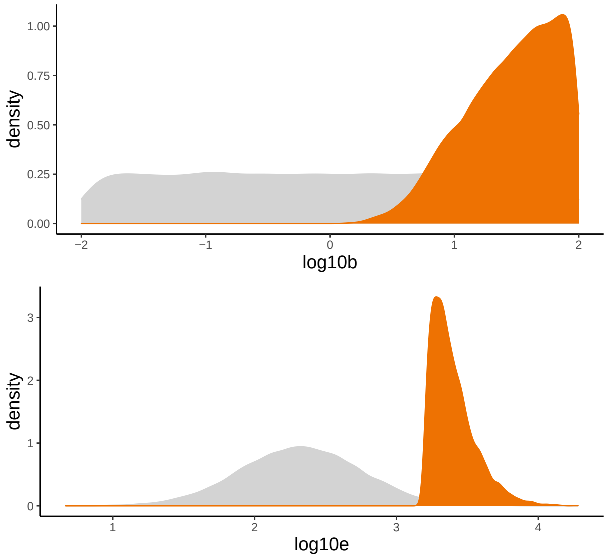

(c) Priors and posteriors

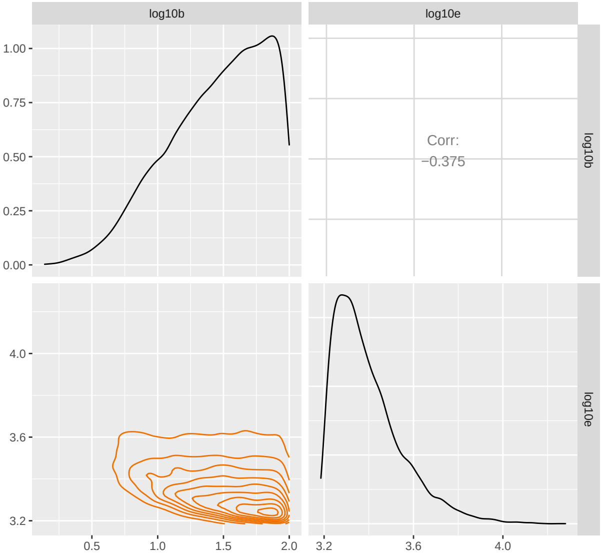

(d) Correlations between parameters

Figure 6: Dose-response curve (a), PPC (b), prior and posterior distributions (c) and correlations between parameters (d).

Data set: LOLPE_VV_survival

Table 7: Summary of parameter estimates (parameter d is set to 1) for LOLPE_VV_survival data set

Parameter	median	Q2.5	Q97.5
b	7.141	1.880	65.361
e	1869.671	1536.087	3938.123

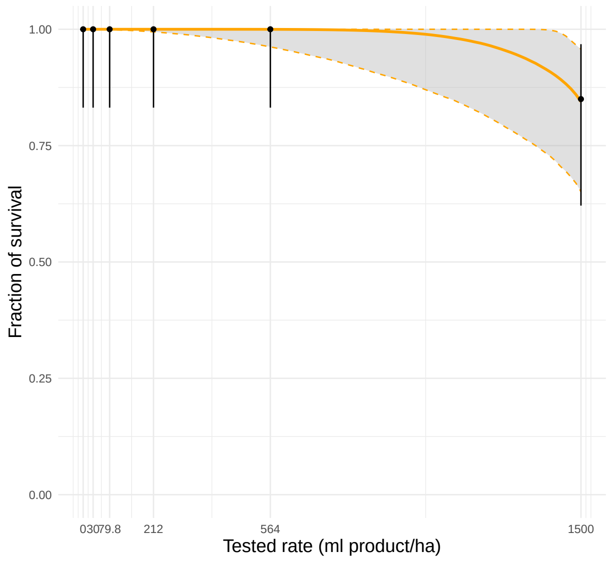

(a) Dose-response curve

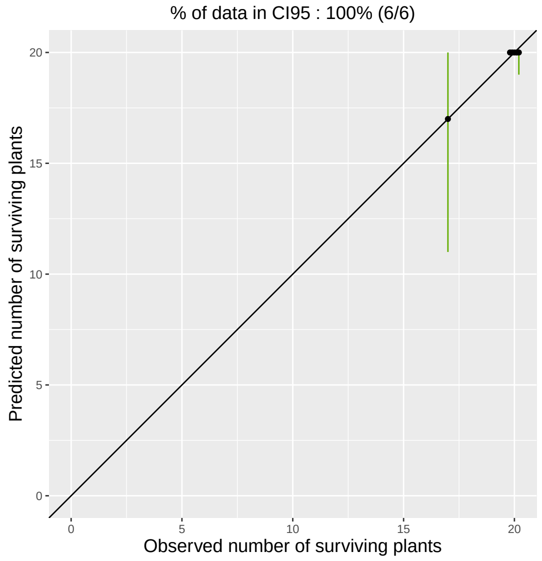

(b) Posterior predictive check (PPC)

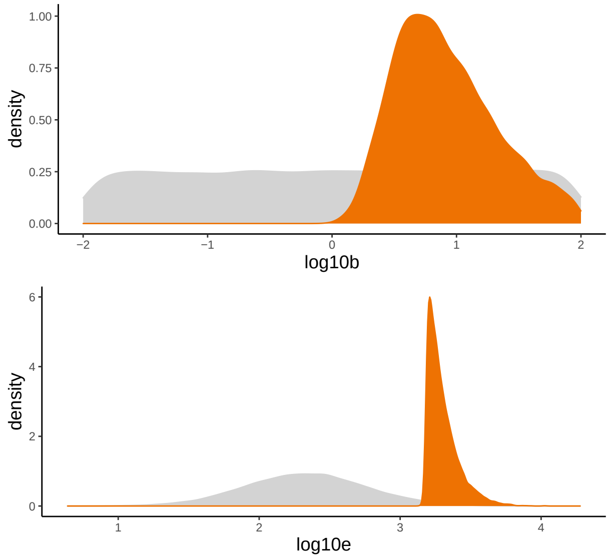

(c) Priors and posteriors

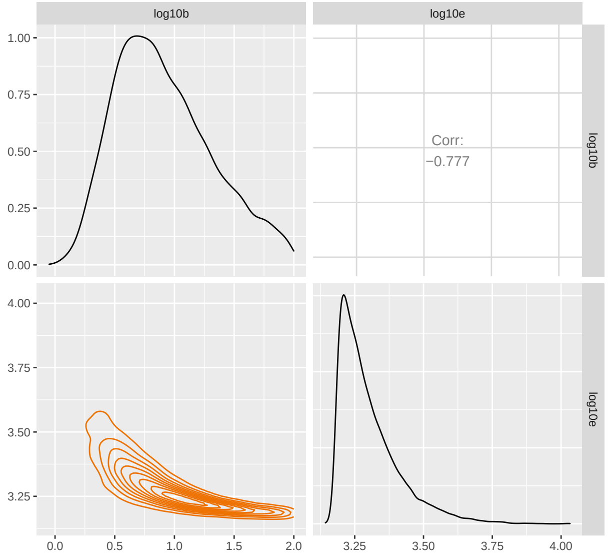

(d) Correlations between parameters

Figure 7: Dose-response curve (a), PPC (b), prior and posterior distributions (c) and correlations between parameters (d).

Data set: LYPES_VV_survival

Table 8: Summary of parameter estimates (parameter d is set to 1) for LYPES_VV_survival data set

Parameter	median	Q2.5	Q97.5
b	3.518	2.092	5.509
e	911.048	712.489	1174.539

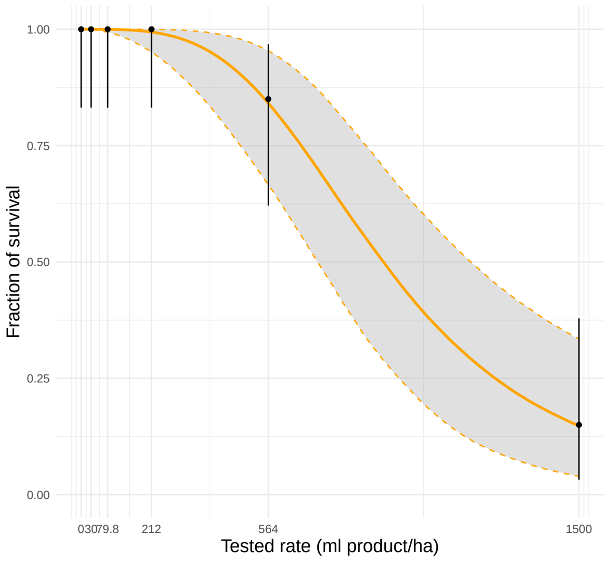

(a) Dose-response curve

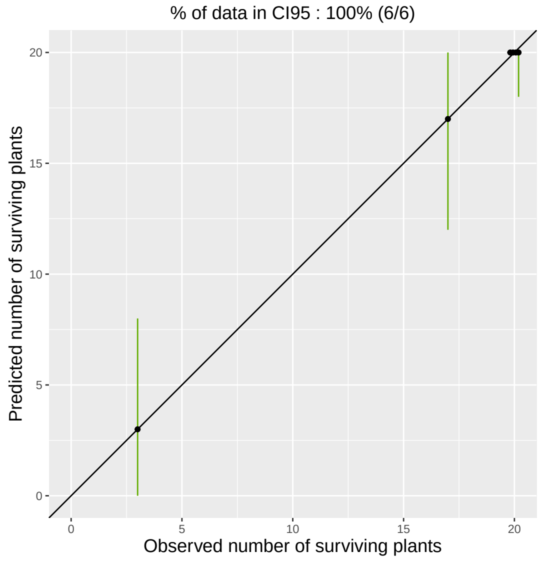

(b) Posterior predictive check (PPC)

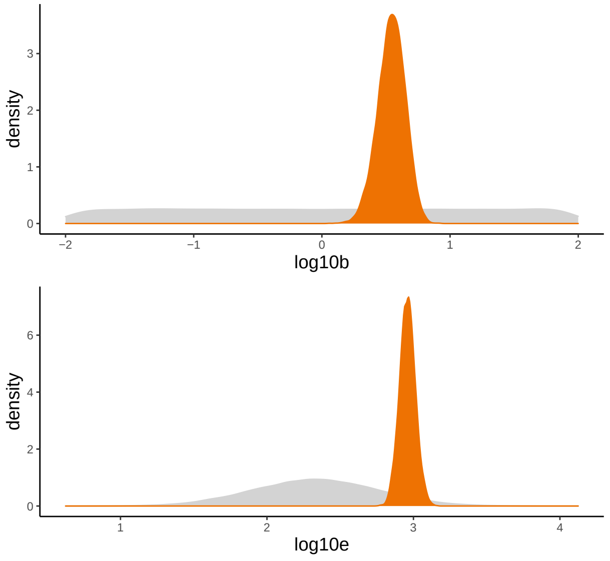

(c) Priors and posteriors

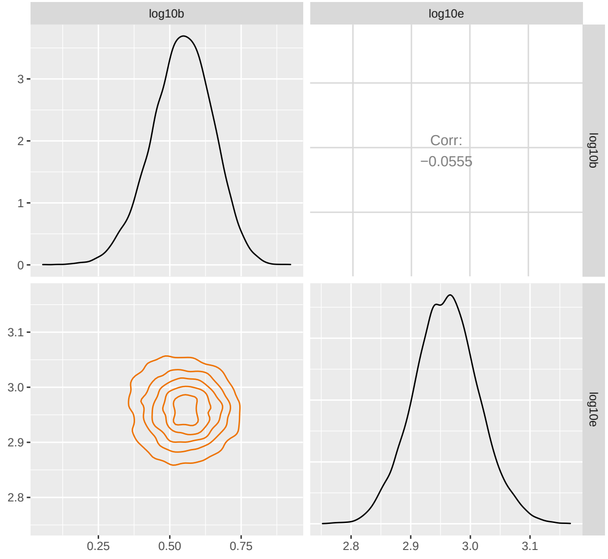

(d) Correlations between parameters

Figure 8: Dose-response curve (a), PPC (b), prior and posterior distributions (c) and correlations between parameters (d).

Data set: TRZAW_VV_survival

Table 9: Summary of parameter estimates (parameter d is set to 1) for TRZAW_VV_survival data set

Parameter	median	Q2.5	Q97.5
b	31.760	4.416	94.655
e	2292.927	1617.456	6114.726

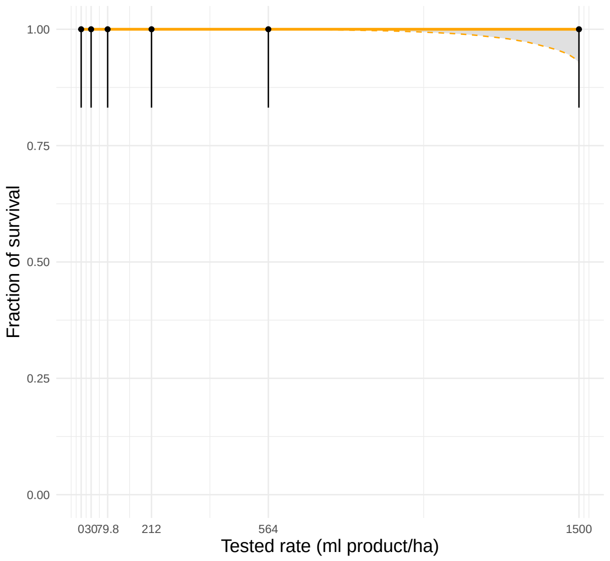

(a) Dose-response curve

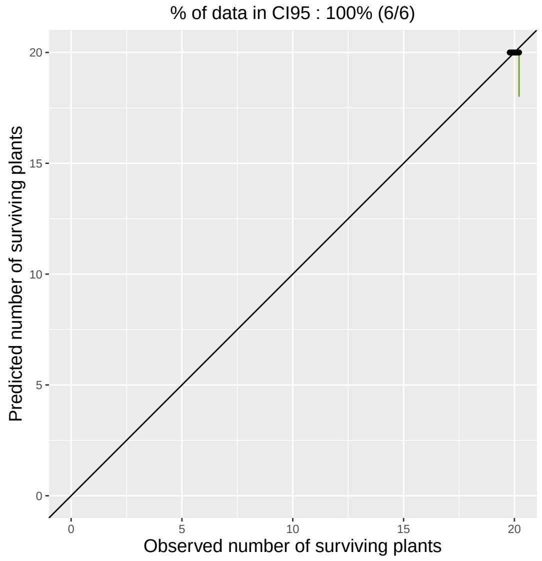

(b) Posterior predictive check (PPC)

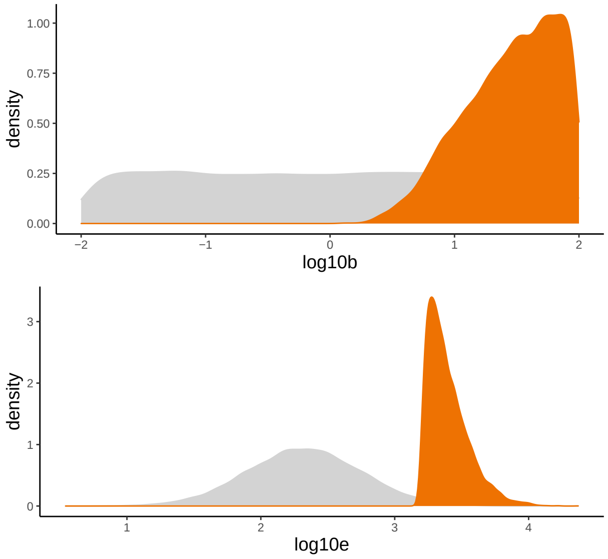

(c) Priors and posteriors

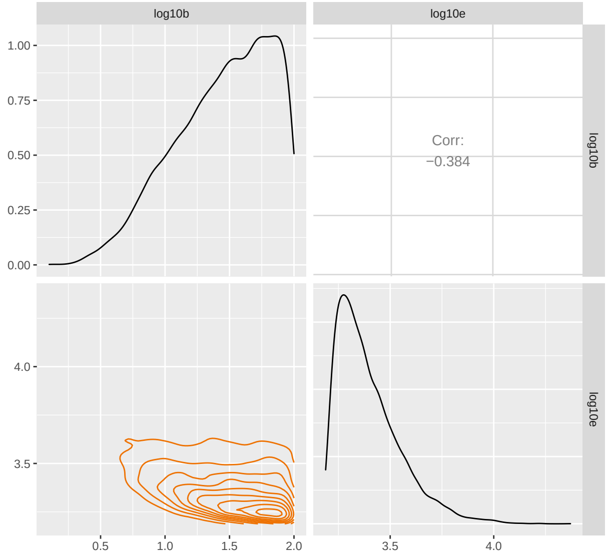

(d) Correlations between parameters

Figure 9: Dose-response curve (a), PPC (b), prior and posterior distributions (c) and correlations between parameters (d).

Data set: ZEAMA_VV_survival

Table 10: Summary of parameter estimates (parameter d is set to 1) for ZEAMA_VV_survival data set

Parameter	median	Q2.5	Q97.5
b	31.684	4.410	94.483
e	2306.614	1622.278	6210.018

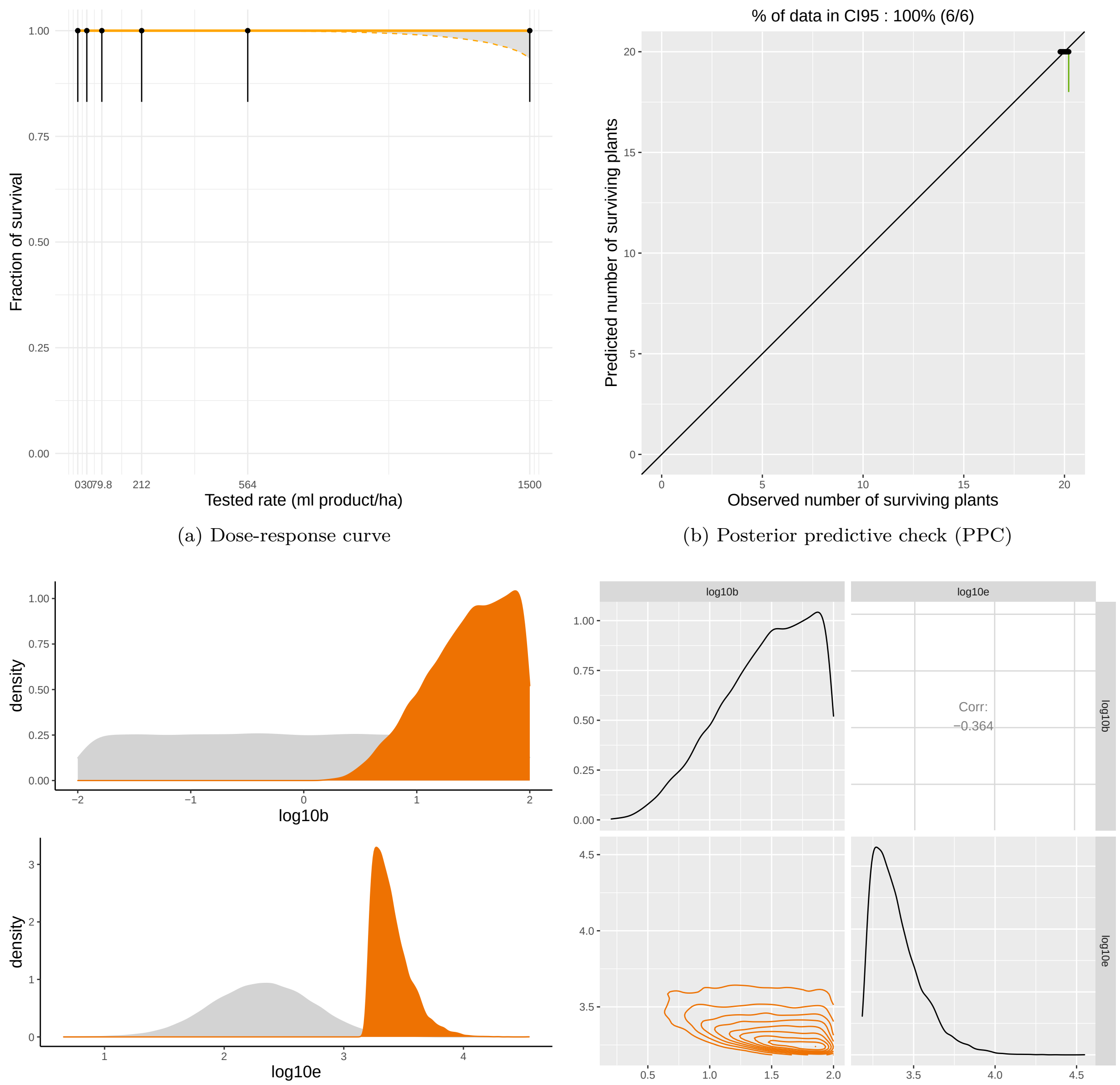

(a) Dose-response curve

(b) Posterior predictive check (PPC)

(c) Priors and posteriors

(d) Correlations between parameters

Figure 10: Dose-response curve (a), PPC (b), prior and posterior distributions (c) and correlations between parameters (d).
