# Supplementary material for: How to account for the uncertainty from standard toxicity tests in species sensitivity distributions: An example in non-target plants
Source: PLoS One. 2021 Jan 7;16(1):e0245071. doi: 10.1371/journal.pone.0245071 (PMC7790375; doi:10.1371/journal.pone.0245071)
Supplement: S1 Archive — It is a zip file containing seven folders (one folder per case study). Each folder contains five files report_xxx.pdf with detailed results of the dose-response analyses, one file corresponding to does-response analysis per endpoint. It also contains one file ER50_censoring.pdf for censored ER50 and one file SSD_analyses.pdf for results of SSD analyses. (ZIP) [file pone.0245071.s004.zip › S1_archive/Study3/report_VV_weight.pdf]

Dose-response analysis

Study 3

Vegetative Vigour test - shoot dry VV_weight endpoint

25 June 2020

Contact: sandrine.charles@univ-lyon1.fr

This is a report which provides results on all performed dose-response analyses for the shoot dry VV_weight endpoint of the Vegetative Vigour test for study 3.

Contents

Data set: ALLCE_VV_weight . . . 2
Data set: BEAVA_VV_weight . . . 3
Data set: BRSNW_VV_weight . . . 4
Data set: CUMSA_VV_weight . . . 5
Data set: FAGES_VV_weight . . . 6
Data set: GLXMA_VV_weight . . . 7
Data set: LOLPE_VV_weight . . . 8
Data set: LYPES_VV_weight . . . 9
Data set: TRZAW_VV_weight . . . 10
Data set: ZEAMA_VV_weight . . . 11

Data set: ALLCE_VV_weight

Table 1: Summary of parameter estimates for ALLCE_VV_weight data set

Parameter	median	Q2.5	Q97.5
b	3.275	1.145	42.386
d	0.318	0.288	0.350
e	1558.400	1196.420	2439.766
sigma	0.068	0.053	0.092

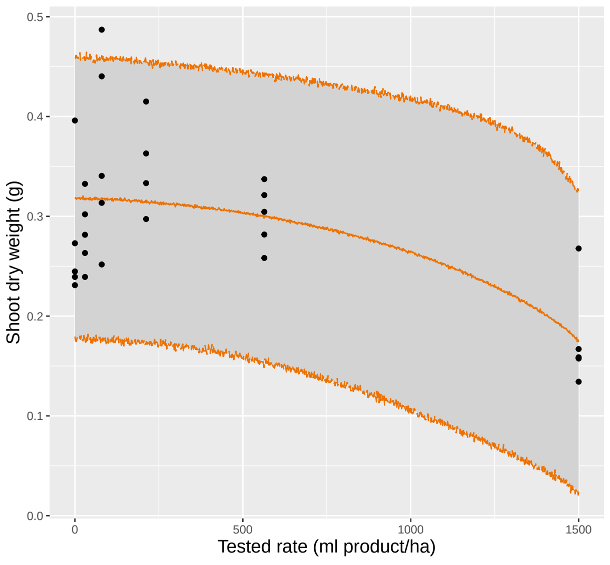

(a) Dose-response curve

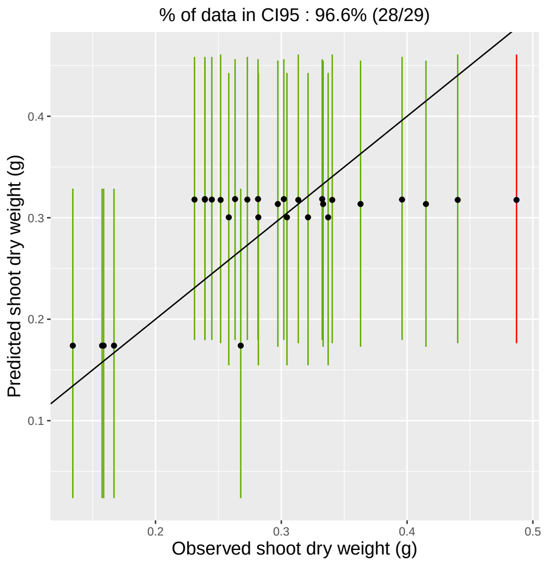

(b) Posterior predictive check (PPC)

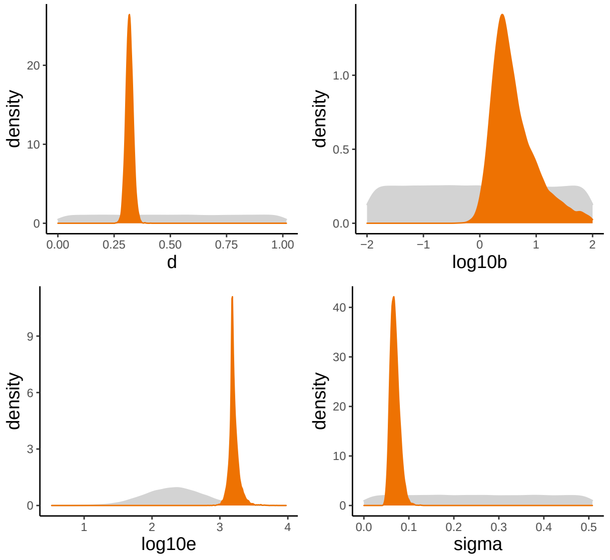

(c) Priors and posteriors

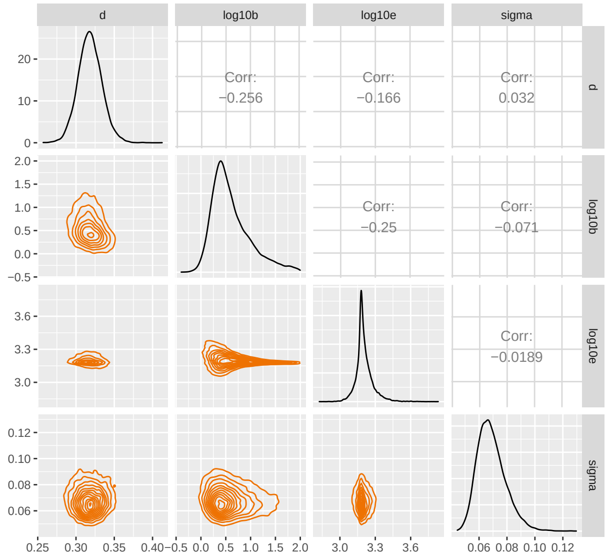

(d) Correlations between parameters

Figure 1: Dose-response curve (a), PPC (b), prior and posterior distributions (c) and correlations between parameters (d).

Data set: BEAVA_VV_weight

Table 2: Summary of parameter estimates for BEAVA_VV_weight data set

Parameter	median	Q2.5	Q97.5
b	1.413	0.718	10.140
d	4.086	3.871	4.352
e	2377.972	1609.346	4221.291
sigma	0.522	0.437	0.638

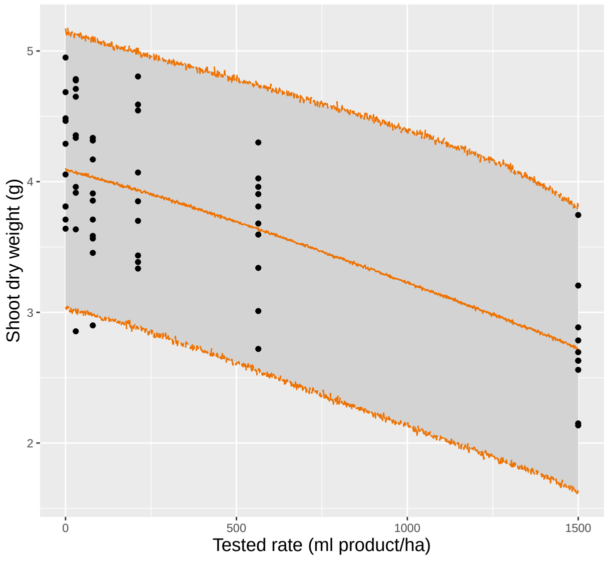

(a) Dose-response curve

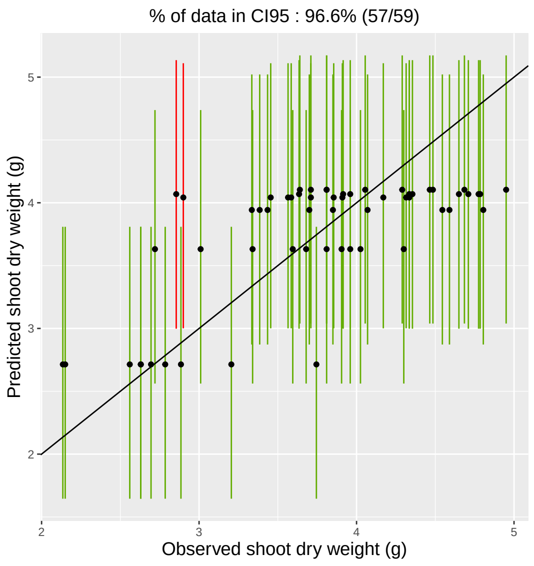

(b) Posterior predictive check (PPC)

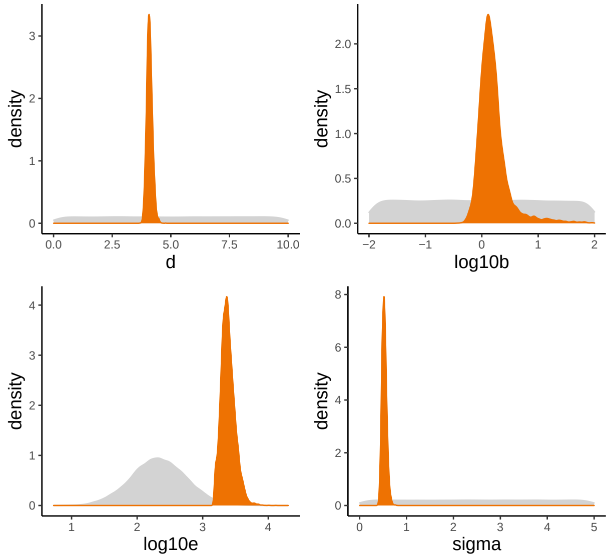

(c) Priors and posteriors

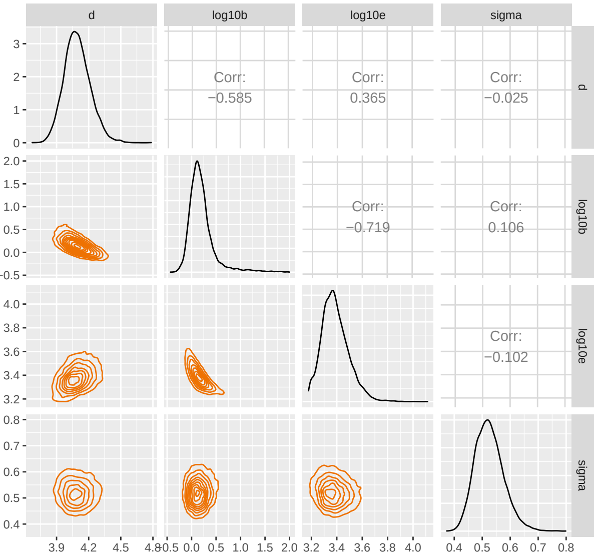

(d) Correlations between parameters

Figure 2: Dose-response curve (a), PPC (b), prior and posterior distributions (c) and correlations between parameters (d).

Data set: BRSNW_VV_weight

Table 3: Summary of parameter estimates for BRSNW_VV_weight data set

Parameter	median	Q2.5	Q97.5
b	1.605	1.214	2.123
d	8.226	7.817	8.658
e	838.599	699.395	1011.098
sigma	1.061	0.889	1.302

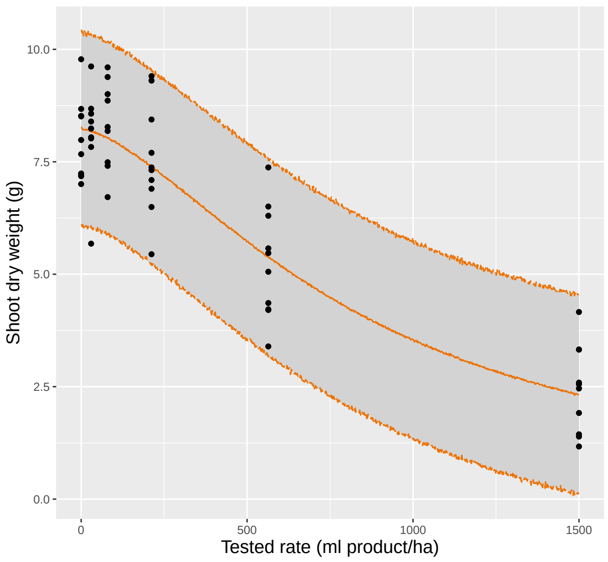

(a) Dose-response curve

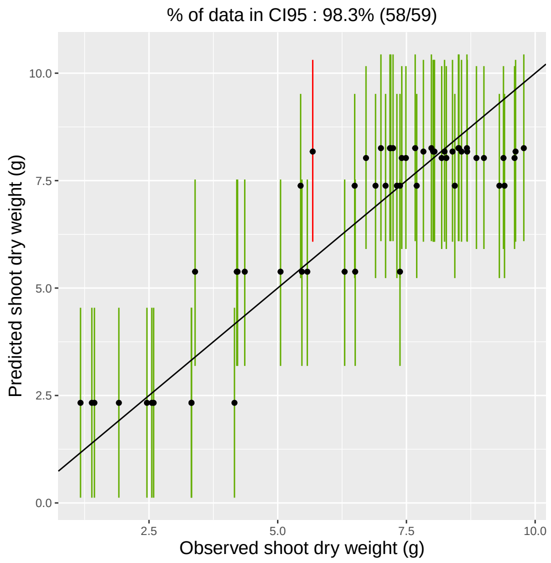

(b) Posterior predictive check (PPC)

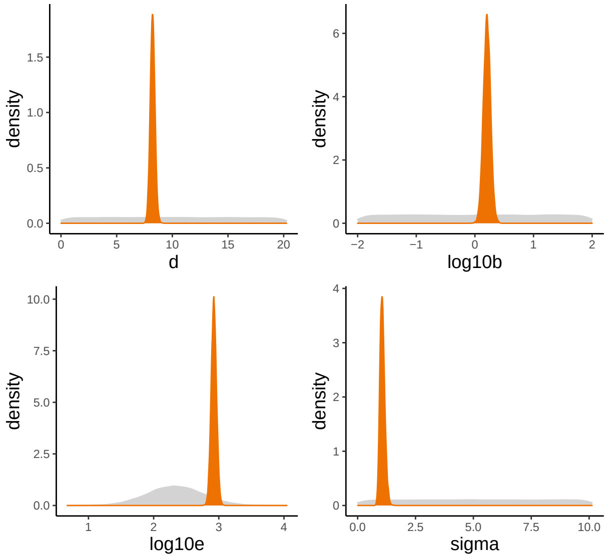

(c) Priors and posteriors

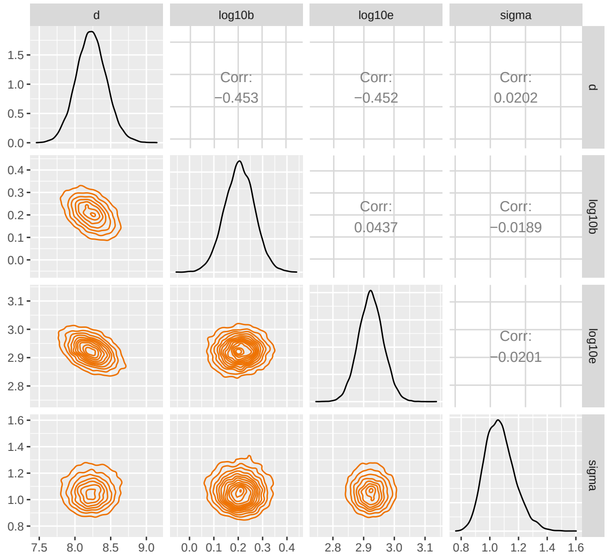

(d) Correlations between parameters

Figure 3: Dose-response curve (a), PPC (b), prior and posterior distributions (c) and correlations between parameters (d).

Data set: CUMSA_VV_weight

Table 4: Summary of parameter estimates for CUMSA_VV_weight data set

Parameter	median	Q2.5	Q97.5
b	1.245	0.979	1.620
d	11.063	10.387	11.798
e	302.975	245.543	372.040
sigma	1.277	1.060	1.581

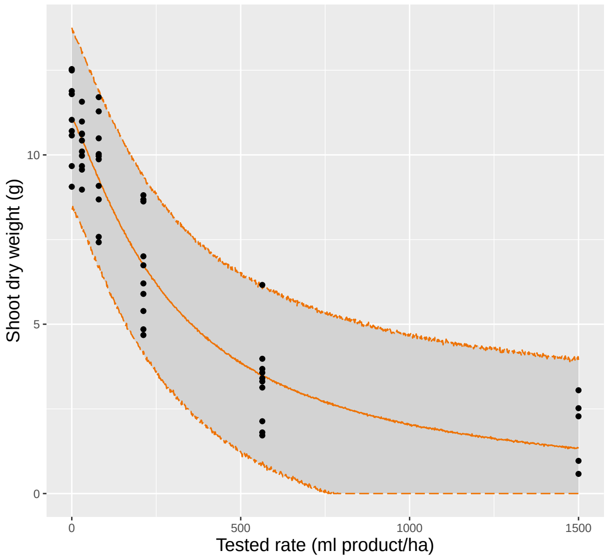

(a) Dose-response curve

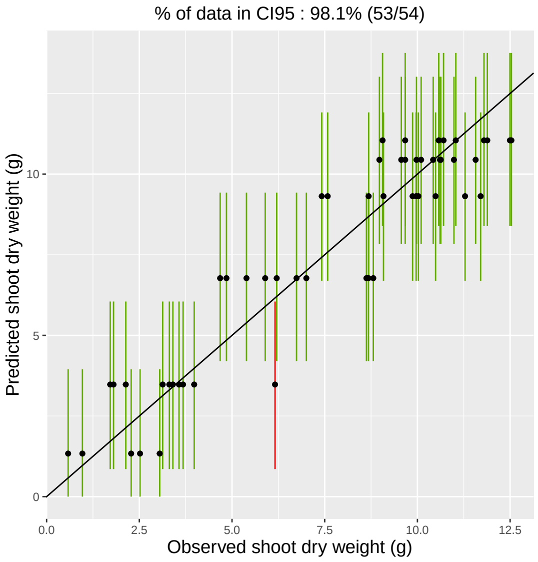

(b) Posterior predictive check (PPC)

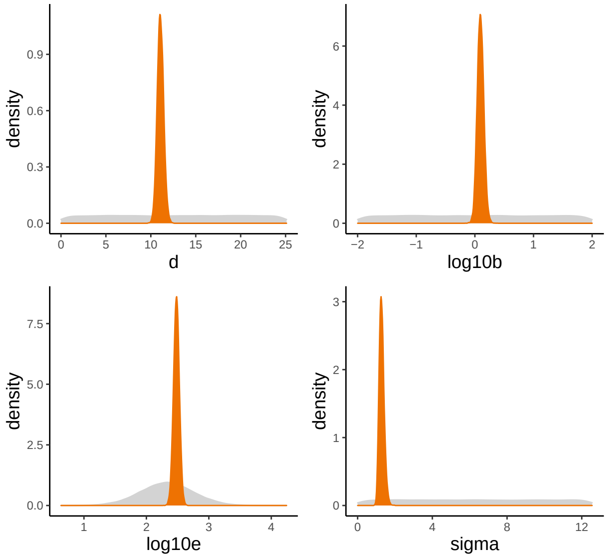

(c) Priors and posteriors

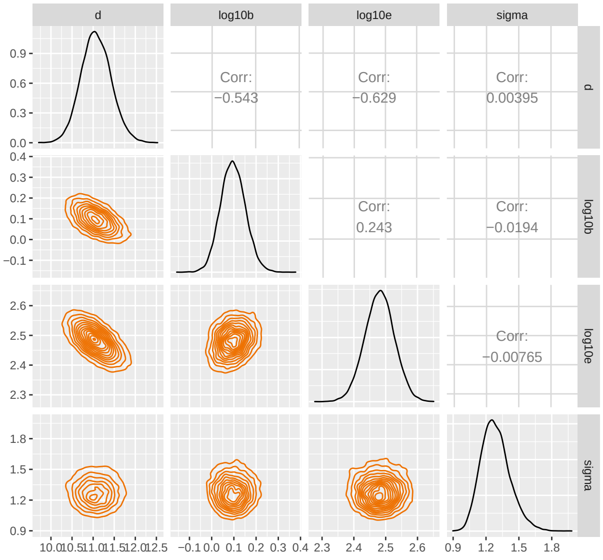

(d) Correlations between parameters

Figure 4: Dose-response curve (a), PPC (b), prior and posterior distributions (c) and correlations between parameters (d).

Data set: FAGES_VV_weight

Table 5: Summary of parameter estimates for FAGES_VV_weight data set

Parameter	median	Q2.5	Q97.5
b	1.353	1.034	1.831
d	6.613	6.129	7.129
e	262.085	205.337	333.376
sigma	0.926	0.772	1.141

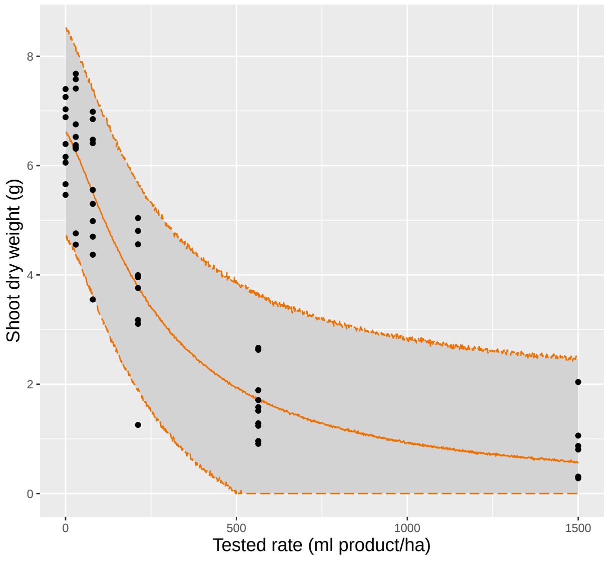

(a) Dose-response curve

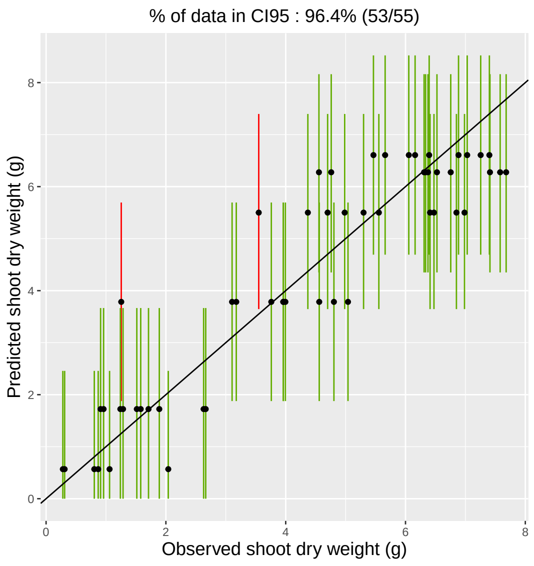

(b) Posterior predictive check (PPC)

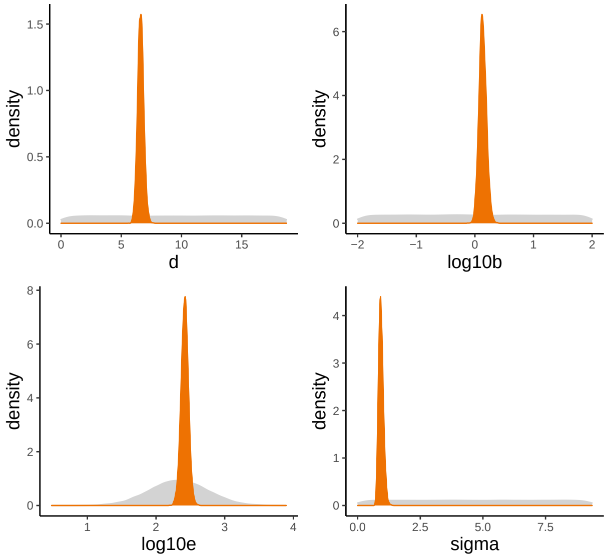

(c) Priors and posteriors

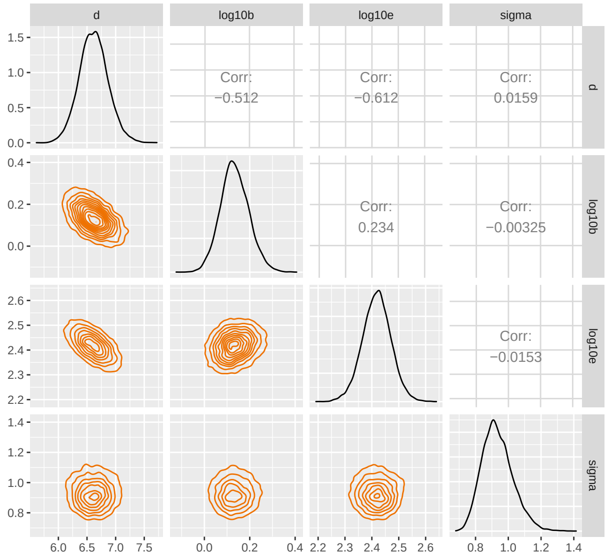

(d) Correlations between parameters

Figure 5: Dose-response curve (a), PPC (b), prior and posterior distributions (c) and correlations between parameters (d).

Data set: GLXMA_VV_weight

Table 6: Summary of parameter estimates for GLXMA_VV_weight data set

Parameter	median	Q2.5	Q97.5
b	1.395	1.090	1.773
d	5.744	5.576	5.924
e	1590.354	1396.262	1865.475
sigma	0.433	0.362	0.528

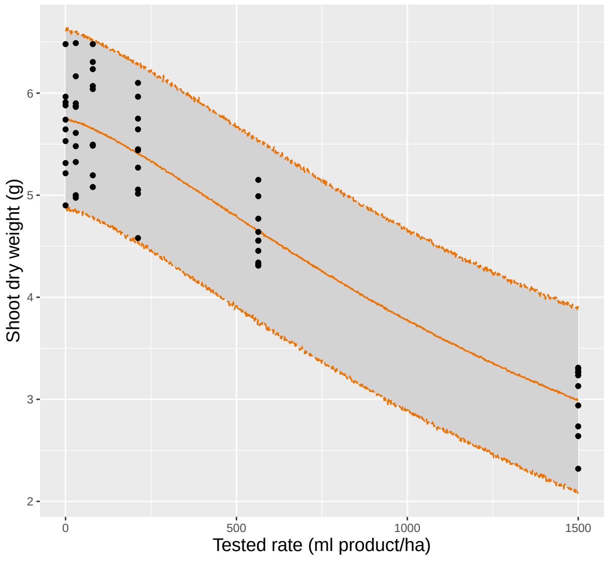

(a) Dose-response curve

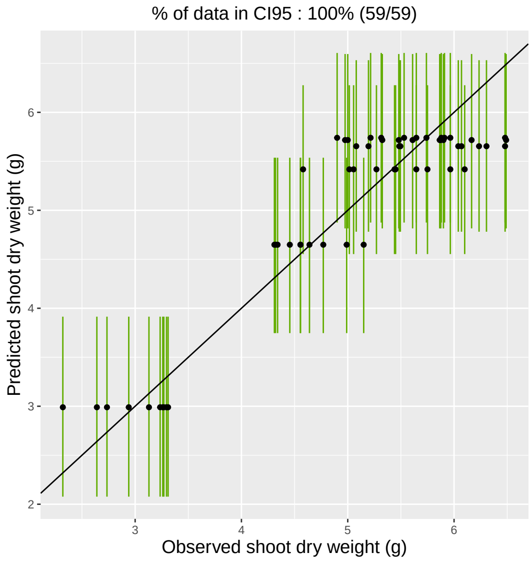

(b) Posterior predictive check (PPC)

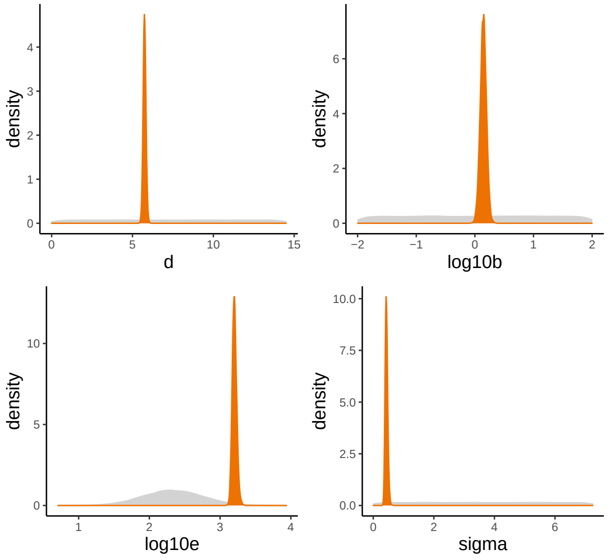

(c) Priors and posteriors

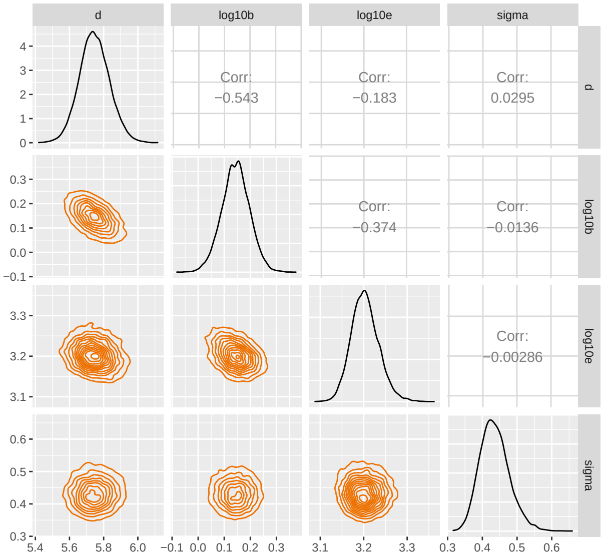

(d) Correlations between parameters

Figure 6: Dose-response curve (a), PPC (b), prior and posterior distributions (c) and correlations between parameters (d).

Data set: LOLPE_VV_weight

Table 7: Summary of parameter estimates for LOLPE_VV_weight data set

Parameter	median	Q2.5	Q97.5
b	1.124	0.726	1.715
d	0.473	0.439	0.515
e	1038.131	759.704	1420.947
sigma	0.051	0.040	0.070

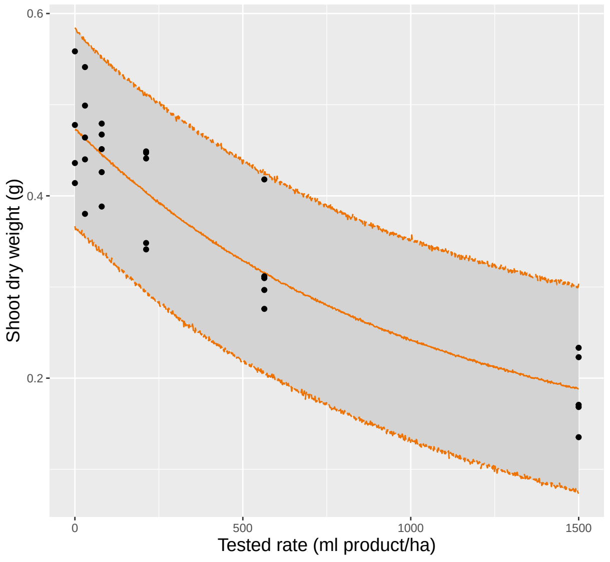

(a) Dose-response curve

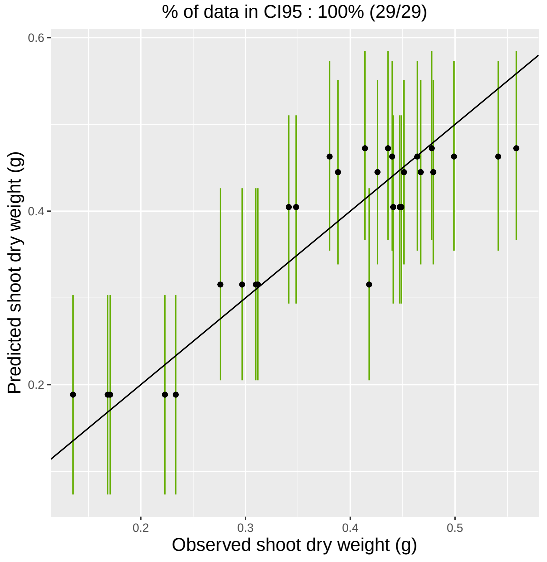

(b) Posterior predictive check (PPC)

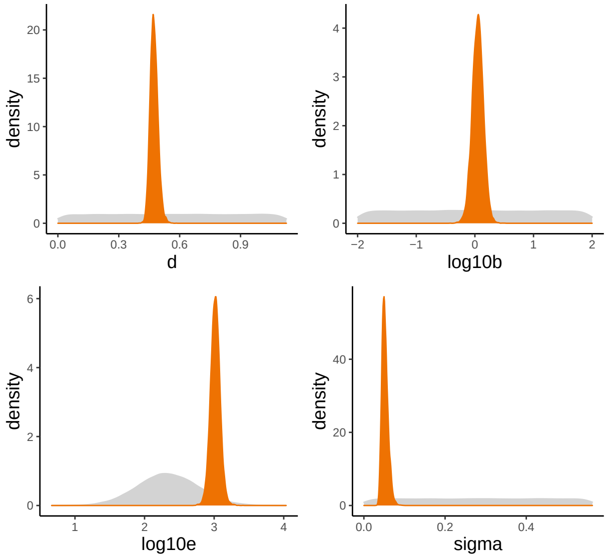

(c) Priors and posteriors

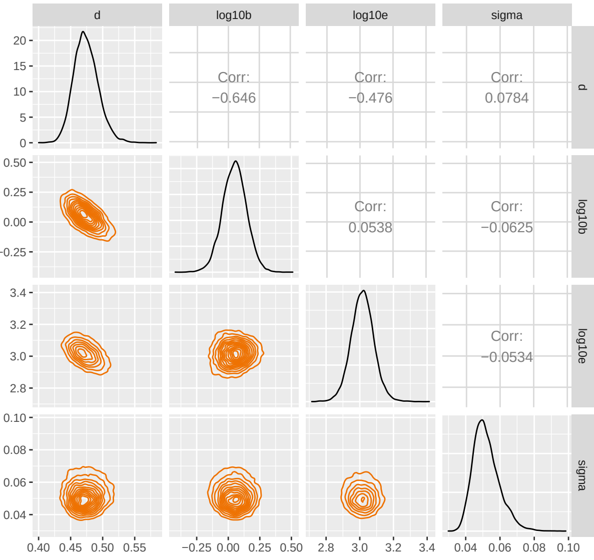

(d) Correlations between parameters

Figure 7: Dose-response curve (a), PPC (b), prior and posterior distributions (c) and correlations between parameters (d).

Data set: LYPES_VV_weight

Table 8: Summary of parameter estimates for LYPES_VV_weight data set

Parameter	median	Q2.5	Q97.5
b	1.248	0.922	1.712
d	3.135	2.852	3.435
e	147.991	109.404	199.499
sigma	0.508	0.418	0.633

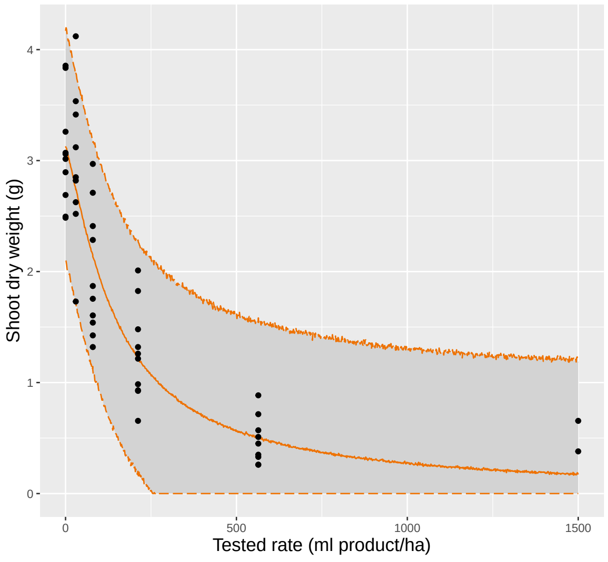

(a) Dose-response curve

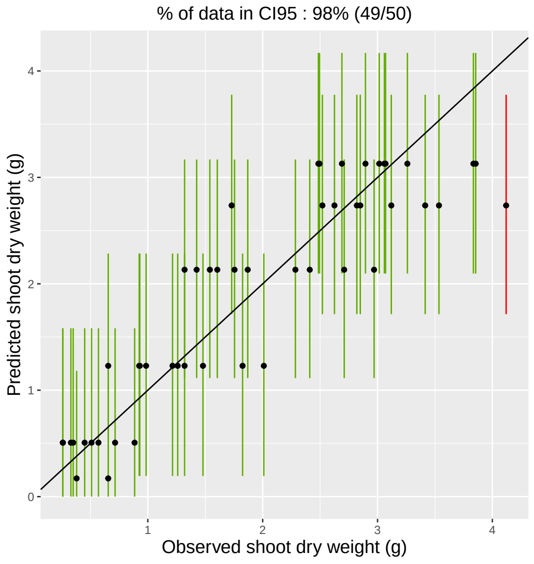

(b) Posterior predictive check (PPC)

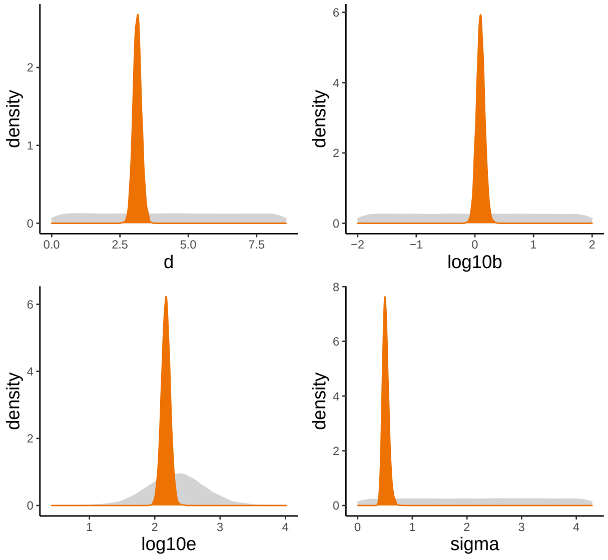

(c) Priors and posteriors

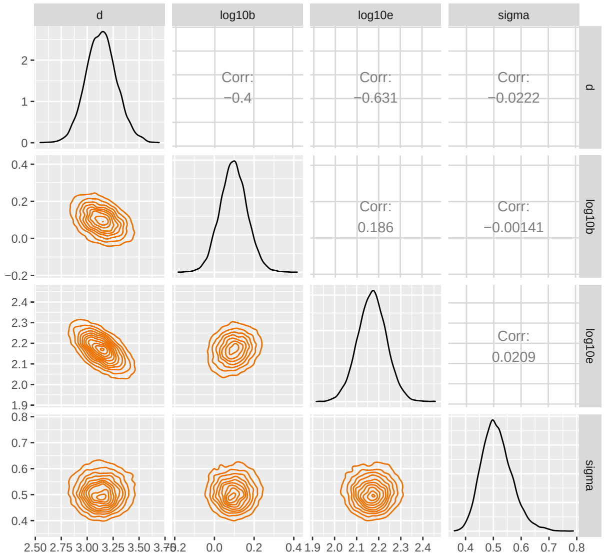

(d) Correlations between parameters

Figure 8: Dose-response curve (a), PPC (b), prior and posterior distributions (c) and correlations between parameters (d).

Data set: TRZAW_VV_weight

Table 9: Summary of parameter estimates for TRZAW_VV_weight data set

Parameter	median	Q2.5	Q97.5
b	1.846	1.431	2.423
d	0.922	0.867	0.980
e	342.308	285.442	409.994
sigma	0.087	0.067	0.118

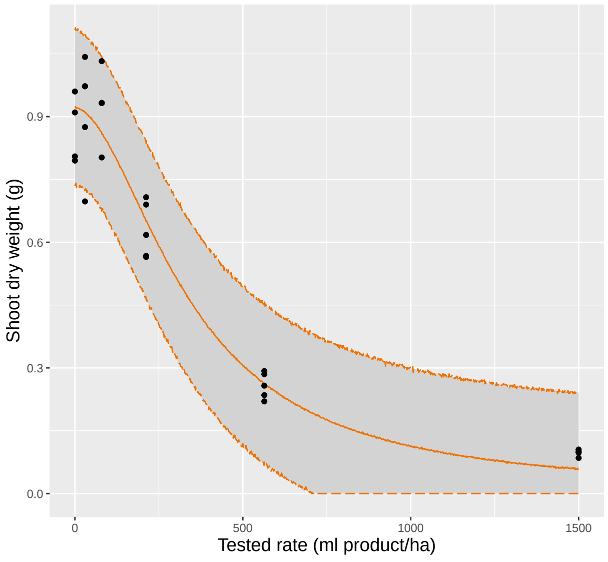

(a) Dose-response curve

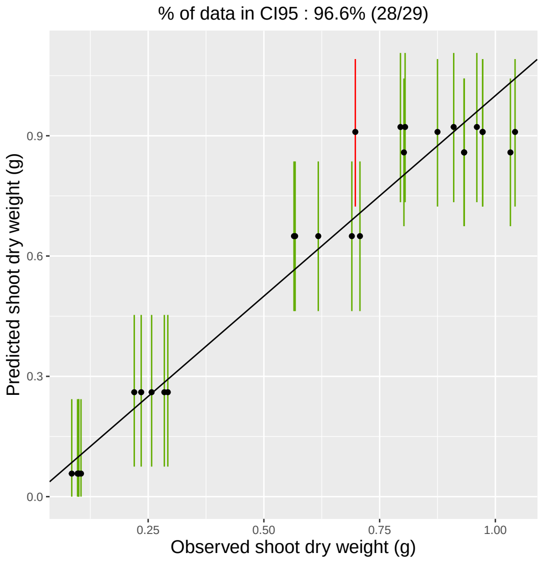

(b) Posterior predictive check (PPC)

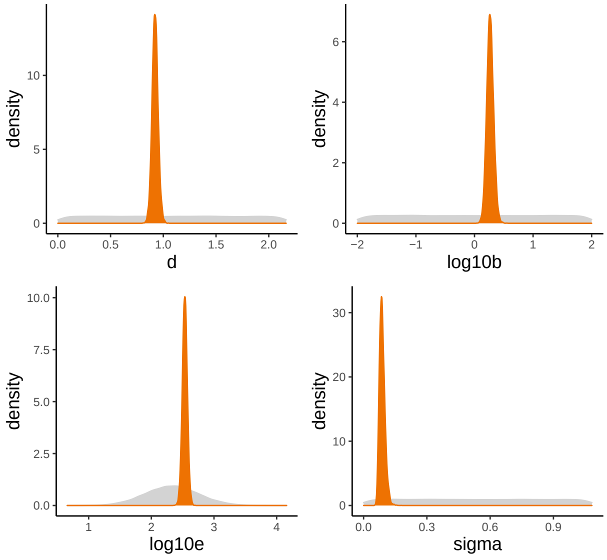

(c) Priors and posteriors

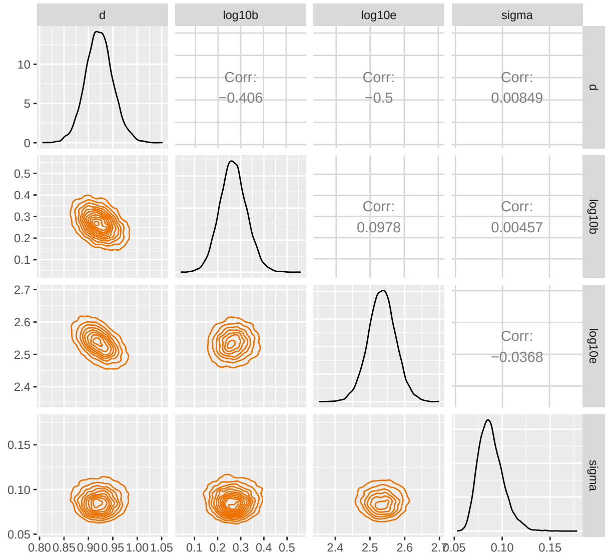

(d) Correlations between parameters

Figure 9: Dose-response curve (a), PPC (b), prior and posterior distributions (c) and correlations between parameters (d).

Data set: ZEAMA_VV_weight

Table 10: Summary of parameter estimates for ZEAMA_VV_weight data set

Parameter	median	Q2.5	Q97.5
b	1.484	1.188	1.846
d	9.111	8.739	9.514
e	682.828	586.529	792.433
sigma	0.904	0.758	1.111

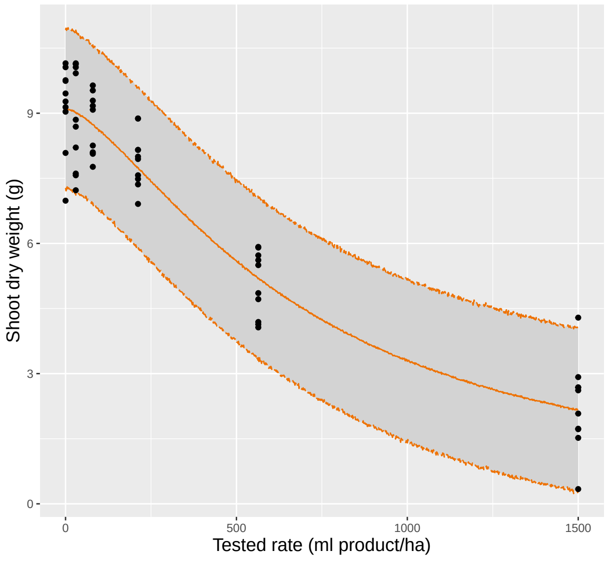

(a) Dose-response curve

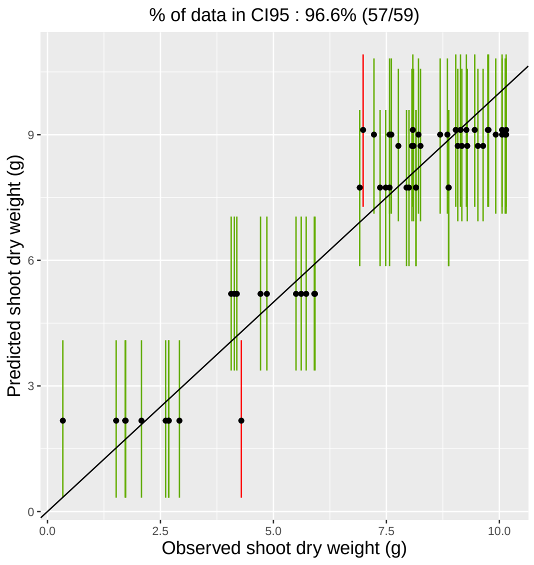

(b) Posterior predictive check (PPC)

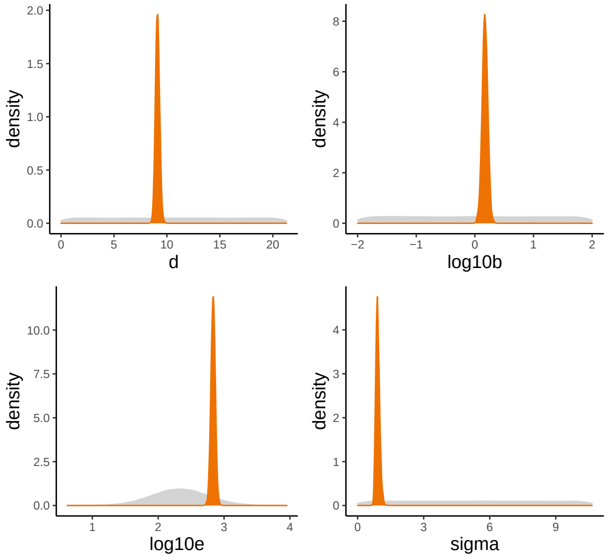

(c) Priors and posteriors

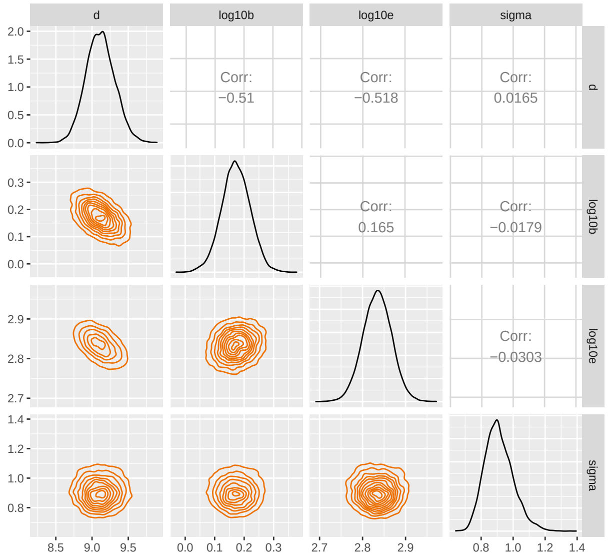

(d) Correlations between parameters

Figure 10: Dose-response curve (a), PPC (b), prior and posterior distributions (c) and correlations between parameters (d).
