# Supplementary material for: How to account for the uncertainty from standard toxicity tests in species sensitivity distributions: An example in non-target plants
Source: PLoS One. 2021 Jan 7;16(1):e0245071. doi: 10.1371/journal.pone.0245071 (PMC7790375; doi:10.1371/journal.pone.0245071)
Supplement: S1 Archive — It is a zip file containing seven folders (one folder per case study). Each folder contains five files report_xxx.pdf with detailed results of the dose-response analyses, one file corresponding to does-response analysis per endpoint. It also contains one file ER50_censoring.pdf for censored ER50 and one file SSD_analyses.pdf for results of SSD analyses. (ZIP) [file pone.0245071.s004.zip › S1_archive/Study3/SSD_analyses.pdf]

Species sensitivity distribution (SSD) analyses by fitting a log-normal distribution

Study 3

25 June 2020

Contact: sandrine.charles@univ-lyon1.fr

The document provides results on species sensitivity distribution (SSD) analyses for study 3. There are five endpoints for study 3: emergence, survival, shoot dry weight of the seedling emergence (SE) test, and survival and shoot dry weight of vegetative vigour (VV) test.

We used SSD to describe the toxicity variation of herbicides among a set of non-target terrestrial plants (NTTP). Based on this SSD, the hazardous rate at which five percent of the species are affected (HR_5) can be estimated. This document aims to compare SSD and HR_5 based on 50% effective rates (ER_{50}) generated in three different ways. We considered:

1. Point estimates (medians of posterior distributions of ER_{50}) as inputs for SSD analyses;
2. Interval-censored ER_{50} by 95% credible interval (CI95);
3. censored ER_{50} according to criterion 1 with a decision threshold T = 0.5

For each endpoint, a total of three SSD are produced:

1. SSD based on medians of posterior distributions of ER_{50} (figure title: median);
2. SSD based on CI95 of ER_{50} (figure title: CI95);
3. SSD based on censored ER_{50} according to criterion 1 with T = 0.5 (figure title: C1T0.5).

Meaning of symbols used in Tables 1-5:

- nb_ER_{50} (RC): number of ER_{50} (number of right-censored ER_{50});
- nb_distER_{50} (RC): number of distinct ER_{50} (number of distinct right-censored ER_{50});
- HR_5_est: HR_5 mean estimate;
- HR_5_median: median of HR_5;
- HR_5_LCoI95: lower bound of the 95% confidence interval (CoI95) of HR_5;
- HR_5_UCoI95: upper bound of the CoI95 of HR_5.

Meaning of legends, lines or curves in Figures 1-5:

- Nb. ER_{50} (RC): number of ER_{50} (number of right-censored ER_{50});
- Nb. distinct ER_{50} (RC): number of distinct ER_{50} (number of distinct right-censored ER_{50});
- HR_5 [CoI95]: HR_5 mean estimate [CoI95 of HR_5];
- Blue points: fractions of affected species (EPPO code) ordered by the median of ER_{50};
- Solid blue horizontal segment: interval-censored ER_{50} by the CI95 of the ER_{50};
- Dotted blue horizontal segment: right-censored ER_{50};
- Solid red curve: fitted SSD curve;
- Dotted red curves: CoI95 of the fitted SSD curve.

HR_5 results and SSD curves

HR_5 and SSD for the emergence endpiont of the SE test of study 3

Table 1: Number of (distinct) ER_{50} (right-censored) and HR_5 estimate for the emergence endpoint of the SE test of study 3 (tested rate unit: ml product/ha)

criterion	nb_ER_{50} (RC)	nb_distER_{50} (RC)	HR_5_est	HR_5_median	HR_5_LCoI95	HR_5_UCoI95	CV
median	10 (0)	10 (0)	1494.558	1553.234	1342.864	2199.330	0.108
CI95	10 (0)	10 (0)	3049.096	3049.096	3049.096	3049.096	0.000
C1T0.5	10 (10)	2 (2)	6171.963	6171.963	6171.963	6171.963	0.000

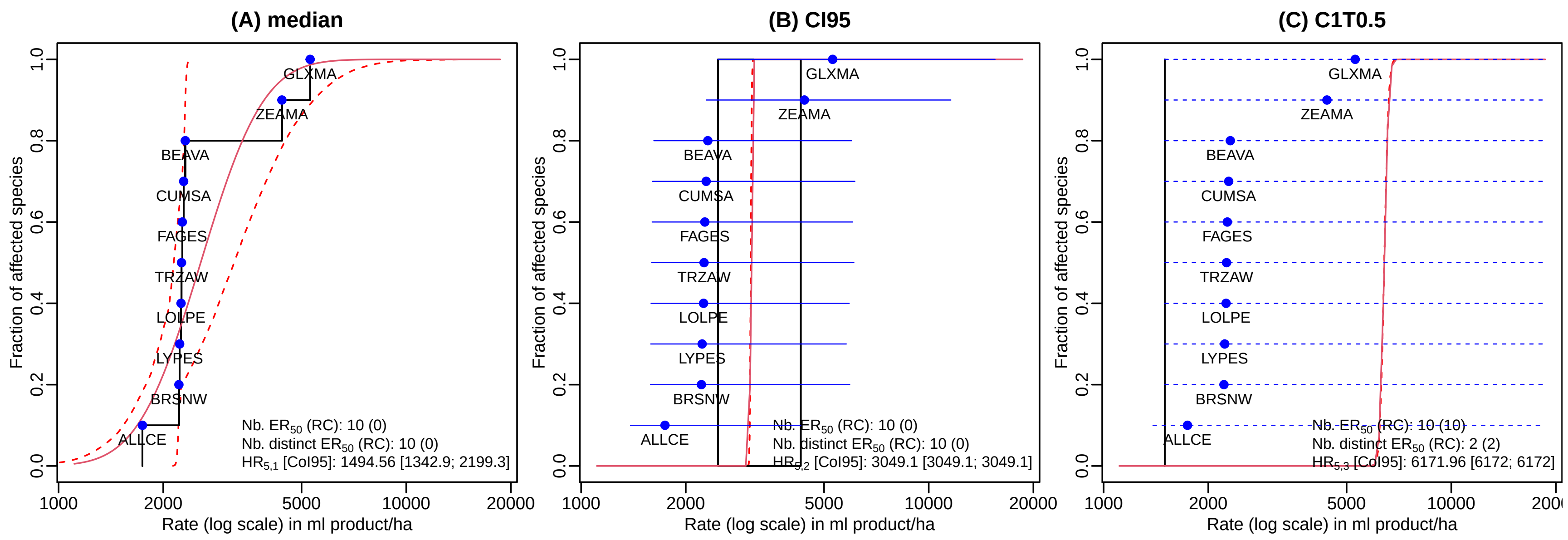

Figure 1: Example of three SSD based on respectively ER_{50} medians (A), CI95 of ER_{50} (B) and censored ER_{50} according to C1T0.5 (C) for the emergnece endpoint of the SE test of study 3.

HR_5 and SSD for the survival endpiont of the SE test of study 3

Table 2: Number of (distinct) ER_{50} (right-censored) and HR_5 estimate for the survival endpoint of the SE test of study 3 (tested rate unit: ml product/ha)

criterion	nb_ER_{50} (RC)	nb_distER_{50} (RC)	HR_5_est	HR_5_median	HR_5_LCoI95	HR_5_UCoI95	CV
median	10 (0)	10 (0)	985.199	993.663	711.974	1772.523	0.244
CI95	10 (0)	10 (0)	937.637	963.355	718.875	2486.907	0.341
C1T0.5	10 (7)	4 (1)	902.435	902.308	707.700	60043.319	3.670

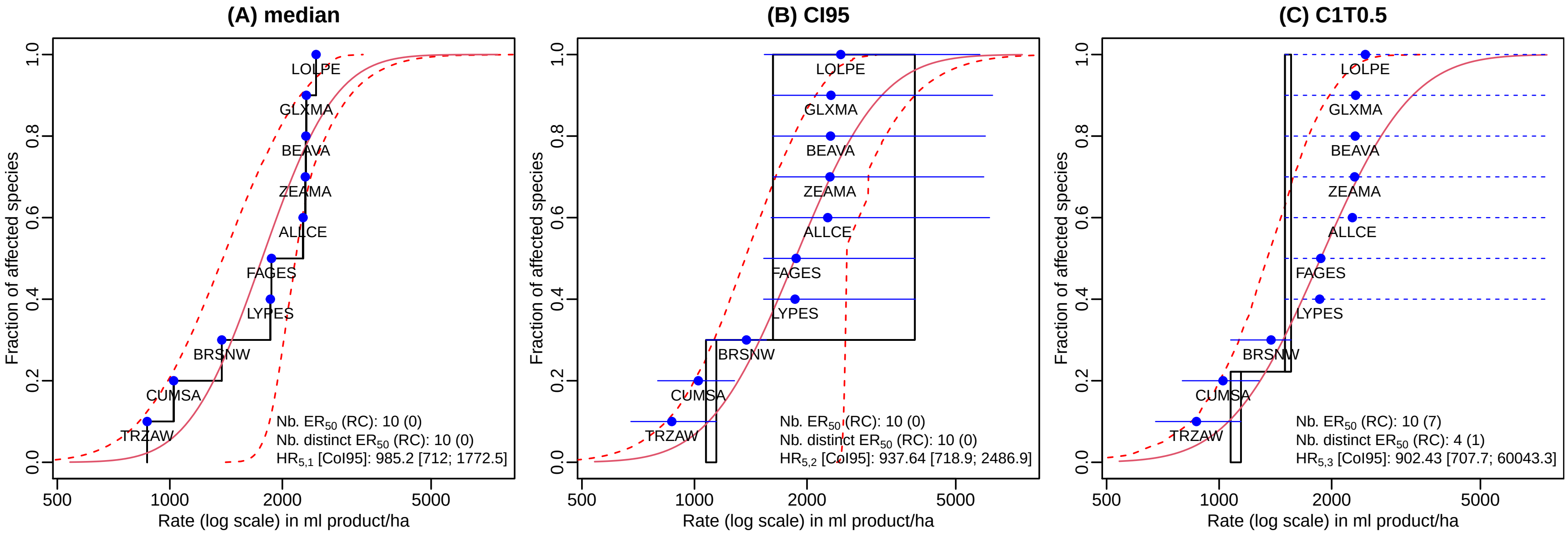

Figure 2: Example of three SSD based on respectively ER_{50} medians (A), CI95 of ER_{50} (B) and censored ER_{50} according to C1T0.5 (C) for the survival endpoint of the SE test of study 3.

HR_5 and SSD for the shoot dry weight endpiont of the SE test of study 3

Table 3: Number of (distinct) ER_{50} (right-censored) and HR_5 estimate for the shoot dry weight endpoint of the SE test of study 3 (tested rate unit: ml product/ha)

criterion	nb_ER_{50} (RC)	nb_distER_{50} (RC)	HR_5_est	HR_5_median	HR_5_LCoI95	HR_5_UCoI95	CV
median	10 (0)	10 (0)	278.499	289.957	135.026	936.582	0.583
CI95	10 (0)	10 (0)	256.987	270.993	129.565	858.505	0.618
C1T0.5	10 (4)	7 (1)	220.765	228.827	105.464	899.891	0.688

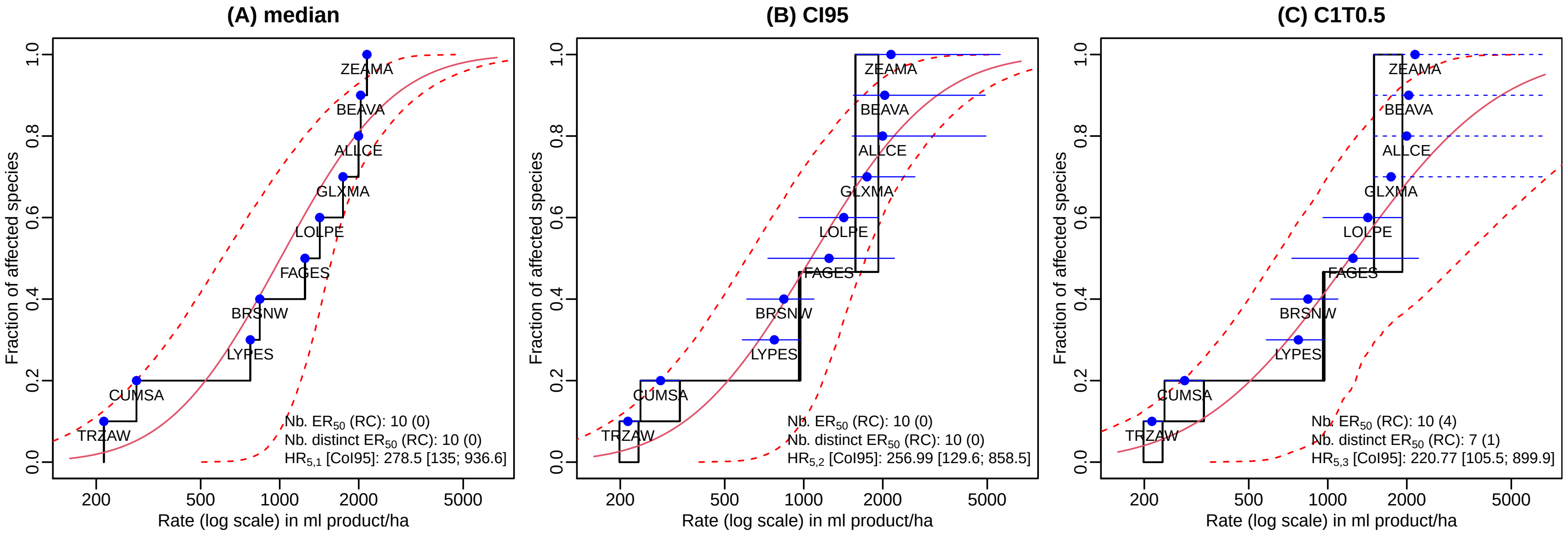

Figure 3: Example of three SSD based on respectively ER_{50} medians (A), CI95 of ER_{50} (B) and censored ER_{50} according to C1T0.5 (C) for the shoot dry weight endpoint of the SE test of study 3.

HR_5 and SSD for the survival endpiont of the VV test of study 3

Table 4: Number of (distinct) ER_{50} (right-censored) and HR_5 estimate for the survival endpoint of the VV test of study 3 (tested rate unit: ml product/ha)

criterion	nb_ER_{50} (RC)	nb_distER_{50} (RC)	HR_5_est	HR_5_median	HR_5_LCoI95	HR_5_UCoI95	CV
median	10 (0)	10 (0)	998.934	1006.836	745.456	1848.204	0.239
CI95	10 (0)	10 (0)	973.151	995.097	756.541	2929.035	0.346
C1T0.5	10 (7)	4 (1)	970.349	963.550	752.531	3160.108	0.456

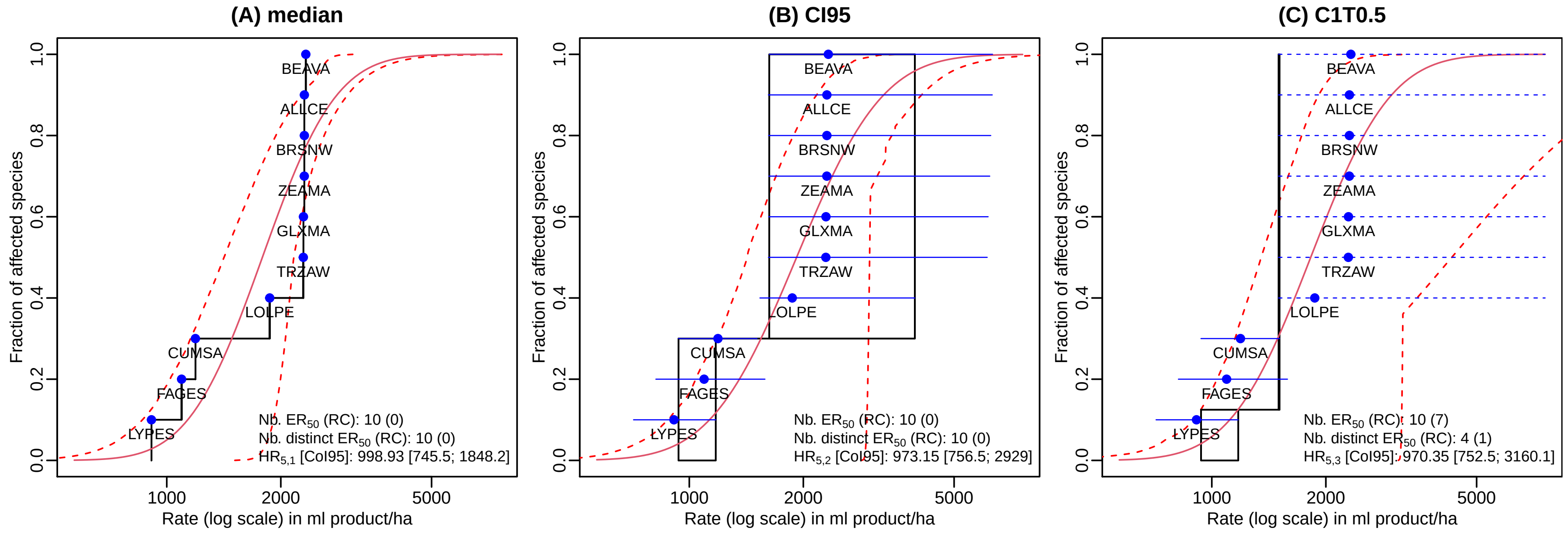

Figure 4: Example of three SSD based on respectively ER_{50} medians (A), CI95 of ER_{50} (B) and censored ER_{50} according to C1T0.5 (C) for the survival endpoint of the VV test of study 3.

HR_5 and SSD for the shoot dry weight endpiont of the VV test of study 3

Table 5: Number of (distinct) ER_{50} (right-censored) and HR_5 estimate for the shoot dry weight endpoint of the VV test of study 3 (tested rate unit: ml product/ha)

criterion	nb_ER_{50} (RC)	nb_distER_{50} (RC)	HR_5_est	HR_5_median	HR_5_LCoI95	HR_5_UCoI95	CV
median	10 (0)	10 (0)	156.494	165.648	88.913	410.637	0.440
CI95	10 (0)	10 (0)	158.350	169.867	90.336	419.016	0.462
C1T0.5	10 (3)	10 (3)	134.986	143.700	71.153	360.827	0.479

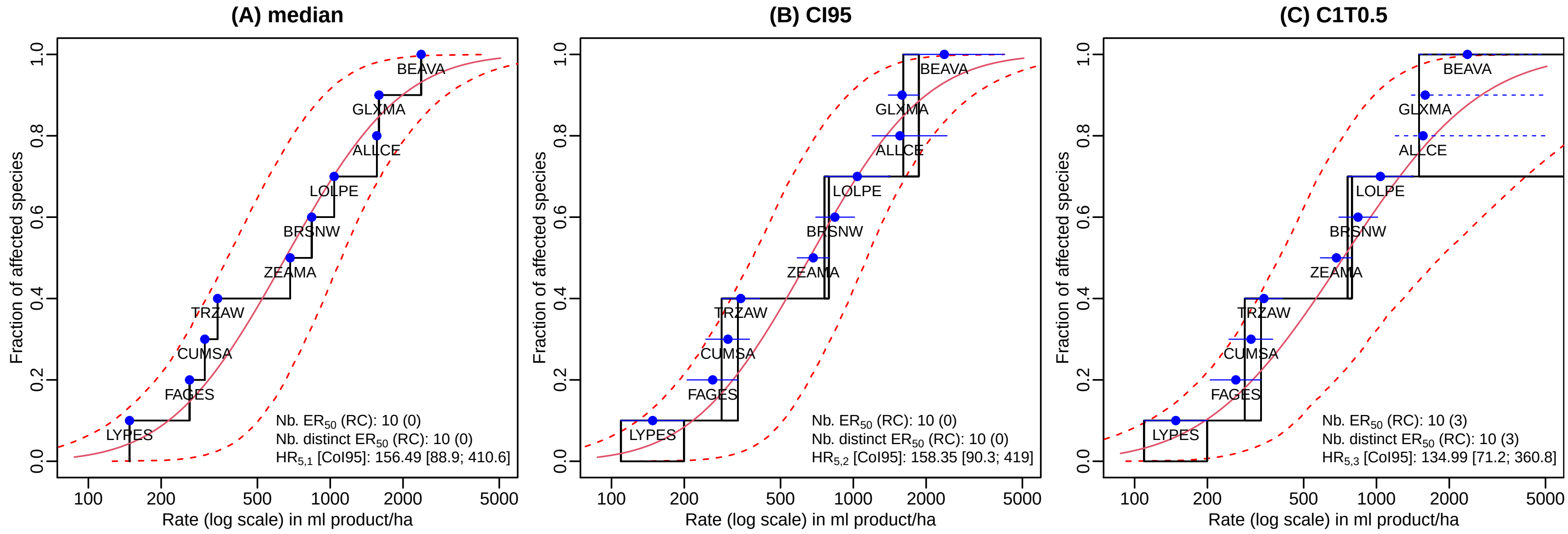

Figure 5: Example of three SSD based on respectively ER_{50} medians (A), CI95 of ER_{50} (B) and censored ER_{50} according to C1T0.5 (C) for the shoot dry weight endpoint of the VV test of study 3.
