# Supplementary material for: How to account for the uncertainty from standard toxicity tests in species sensitivity distributions: An example in non-target plants
Source: PLoS One. 2021 Jan 7;16(1):e0245071. doi: 10.1371/journal.pone.0245071 (PMC7790375; doi:10.1371/journal.pone.0245071)
Supplement: S1 Archive — It is a zip file containing seven folders (one folder per case study). Each folder contains five files report_xxx.pdf with detailed results of the dose-response analyses, one file corresponding to does-response analysis per endpoint. It also contains one file ER50_censoring.pdf for censored ER50 and one file SSD_analyses.pdf for results of SSD analyses. (ZIP) [file pone.0245071.s004.zip › S1_archive/Study4/ER50_censoring.pdf]

ER_{50} censoring after dose-response analyses
Study 4

25 June 2020

Contact: sandrine.charles@univ-lyon1.fr

This document provides the ER_{50} censoring after dose-response analyses for study 4, including four parts:

- Raw data description (test item rates);
- Summary on ER_{50} from a Bayesian dose-response fit;
- Censoring on ER_{50} according to two criteria;
  - Criterion 1 (C_1) based on the ratio of the probablities, that is the ratio of the probability that the ER_{50} lies within the range of the LCI95 and the maximum tested rate over the probability that the ER_{50} lies in the CI95;
  - Criterion 2 (C_2) based on the ratio of distances, that is the ratio of the distance between the LCI95 of ER_{50} and the maximum tested rate over the extend of the CI95 of ER_{50};
- Graphical presentation of censored ER_{50} per species per endpoint.

Meaning of symbols used in the document

- min_rate: minimum tested rate;
- max_rate: maximum tested rate;
- ER_{50}: 50% effective rate;
- CI95: 95% credible interval;
- ER_{50}_median: median of ER_{50};
- ER_{50}_LCI95: lower bound of the CI95 of ER_{50};
- ER_{50}_UCI95: upper bound of the CI95 of ER_{50};
- C_1: criterion based on the ratio of probabilities;
- C_2: criterion based on the ratio of distances;
- T: decision threshold;
- Censored ER_{50} [lower bound; upper bound]:
  - C1T0.0: censored ER_{50} according to C_1 with T = 0.0;
  - C1T0.2: censored ER_{50} according to C_1 with T = 0.2;

- C1T0.4: censored ER_{50} according to C_1 with $T = 0.4$;
- C1T0.5: censored ER_{50} according to C_1 with $T = 0.5$;
- C1T0.6: censored ER_{50} according to C_1 with $T = 0.6$;
- C1T0.8: censored ER_{50} according to C_1 with $T = 0.8$;
- C1T1.0: censored ER_{50} according to C_1 with $T = 1.0$;
- C2T0.0: censored ER_{50} according to C_2 with $T = 0.0$;
- C2T0.2: censored ER_{50} according to C_2 with $T = 0.2$;
- C2T0.4: censored ER_{50} according to C_2 with $T = 0.4$;
- C2T0.5: censored ER_{50} according to C_2 with $T = 0.5$;
- C2T0.6: censored ER_{50} according to C_2 with $T = 0.6$;
- C2T0.8: censored ER_{50} according to C_2 with $T = 0.8$;
- C2T1.0: censored ER_{50} according to C_2 with $T = 1.0$.

Seeding emergence (SE) test of study 4

Raw data description (test item rates) for the SE test

```
Number of species:  10
EPPO code:  ALLCE AVESA BEAVA BRSNW CUMSA GLXMA HELAN LYPES TRZAW ZEAMA
```

Table 1: Correspondance between species and common names according to the EPPO code

EPPO code	Species name	Common name
ALLCE	*Allium cepa*	Onion
AVESA	*Avena sativa*	Oat
BEAVA	*Beta vulgaris*	Sugar beet
BRSNW	*Brassica napus winter*	Oilseed rape
CUMSA	*Cucumis sativus*	Cucumber
GLXMA	*Glycine max*	Soybean
HELAN	*Helianthus annuus*	Sunflower
LYPES	*Solanum lycopersicum*	Tomato
TRZAW	*Triticum aestivum winter*	Winter wheat
ZEAMA	*Zea mays subs. Amylacea*	Corn

SE test - emergence endpoint

Table 2: Minimum and maximum tested rates, median, quantile 2.5% and quantile 97.5% of the ER_{50} posterior distribution - emergence endpoint of the SE test of study 4

species	min_rate	max_rate	ER_{50}_median	ER_{50}_LCI95	ER_{50}_UCI95
ALLCE	0.56	6.76	8.737041	7.038026	16.46936
AVESA	35.73	1000.00	1405.379614	1051.828431	3219.67561
BEAVA	6.76	189.04	246.873086	192.772785	535.73311
BRSNW	6.76	189.04	287.260946	143.556281	745.98707
CUMSA	15.54	434.78	1344.513953	705.782776	3354.31976
GLXMA	15.54	434.78	619.582480	459.192649	1410.82322
HELAN	15.54	434.78	670.562044	407.863522	1441.41726
LYPES	15.54	434.78	532.937958	439.202495	1124.71900
TRZAW	35.73	1000.00	1420.399659	1061.659232	3185.00617
ZEAMA	35.73	1000.00	3051.784859	1639.983311	7638.38729

Table 3: Censored ER_{50} according to criterion 1 and the seven decision thresholds - emergence endpoint of the SE test of study 4

species	C1T0.0	C1T0.2	C1T0.4	C1T0.5	C1T0.6	C1T0.8	C1T1.0
ALLCE	[6.76; Inf)	[6.76; Inf)	[6.76; Inf)	[6.76; Inf)	[6.76; Inf)	[6.76; Inf)	[6.76; Inf)
AVESA	[1000; Inf)	[1000; Inf)	[1000; Inf)	[1000; Inf)	[1000; Inf)	[1000; Inf)	[1000; Inf)
BEAVA	[189.04; Inf)	[189.04; Inf)	[189.04; Inf)	[189.04; Inf)	[189.04; Inf)	[189.04; Inf)	[189.04; Inf)
BRSNW	[143.56; 745.99]	[143.56; Inf)	[143.56; Inf)	[143.56; Inf)	[143.56; Inf)	[143.56; Inf)	[143.56; Inf)
CUMSA	[434.78; Inf)	[434.78; Inf)	[434.78; Inf)	[434.78; Inf)	[434.78; Inf)	[434.78; Inf)	[434.78; Inf)
GLXMA	[434.78; Inf)	[434.78; Inf)	[434.78; Inf)	[434.78; Inf)	[434.78; Inf)	[434.78; Inf)	[434.78; Inf)
HELAN	[407.86; 1441.42]	[407.86; Inf)	[407.86; Inf)	[407.86; Inf)	[407.86; Inf)	[407.86; Inf)	[407.86; Inf)
LYPES	[434.78; Inf)	[434.78; Inf)	[434.78; Inf)	[434.78; Inf)	[434.78; Inf)	[434.78; Inf)	[434.78; Inf)
TRZAW	[1000; Inf)	[1000; Inf)	[1000; Inf)	[1000; Inf)	[1000; Inf)	[1000; Inf)	[1000; Inf)
ZEAMA	[1000; Inf)	[1000; Inf)	[1000; Inf)	[1000; Inf)	[1000; Inf)	[1000; Inf)	[1000; Inf)

Table 4: Censored ER_{50} according to criterion 2 and the seven decision thresholds - emergence endpoint of the SE test of study 4

species	C2T0.0	C2T0.2	C2T0.4	C2T0.5	C2T0.6	C2T0.8	C2T1.0
ALLCE	[6.76; Inf)	[6.76; Inf)	[6.76; Inf)	[6.76; Inf)	[6.76; Inf)	[6.76; Inf)	[6.76; Inf)
AVESA	[1000; Inf)	[1000; Inf)	[1000; Inf)	[1000; Inf)	[1000; Inf)	[1000; Inf)	[1000; Inf)
BEAVA	[189.04; Inf)	[189.04; Inf)	[189.04; Inf)	[189.04; Inf)	[189.04; Inf)	[189.04; Inf)	[189.04; Inf)
BRSNW	[143.56; 745.99]	[143.56; Inf)	[143.56; Inf)	[143.56; Inf)	[143.56; Inf)	[143.56; Inf)	[143.56; Inf)
CUMSA	[434.78; Inf)	[434.78; Inf)	[434.78; Inf)	[434.78; Inf)	[434.78; Inf)	[434.78; Inf)	[434.78; Inf)
GLXMA	[434.78; Inf)	[434.78; Inf)	[434.78; Inf)	[434.78; Inf)	[434.78; Inf)	[434.78; Inf)	[434.78; Inf)
HELAN	[407.86; 1441.42]	[407.86; Inf)	[407.86; Inf)	[407.86; Inf)	[407.86; Inf)	[407.86; Inf)	[407.86; Inf)
LYPES	[434.78; Inf)	[434.78; Inf)	[434.78; Inf)	[434.78; Inf)	[434.78; Inf)	[434.78; Inf)	[434.78; Inf)
TRZAW	[1000; Inf)	[1000; Inf)	[1000; Inf)	[1000; Inf)	[1000; Inf)	[1000; Inf)	[1000; Inf)
ZEAMA	[1000; Inf)	[1000; Inf)	[1000; Inf)	[1000; Inf)	[1000; Inf)	[1000; Inf)	[1000; Inf)

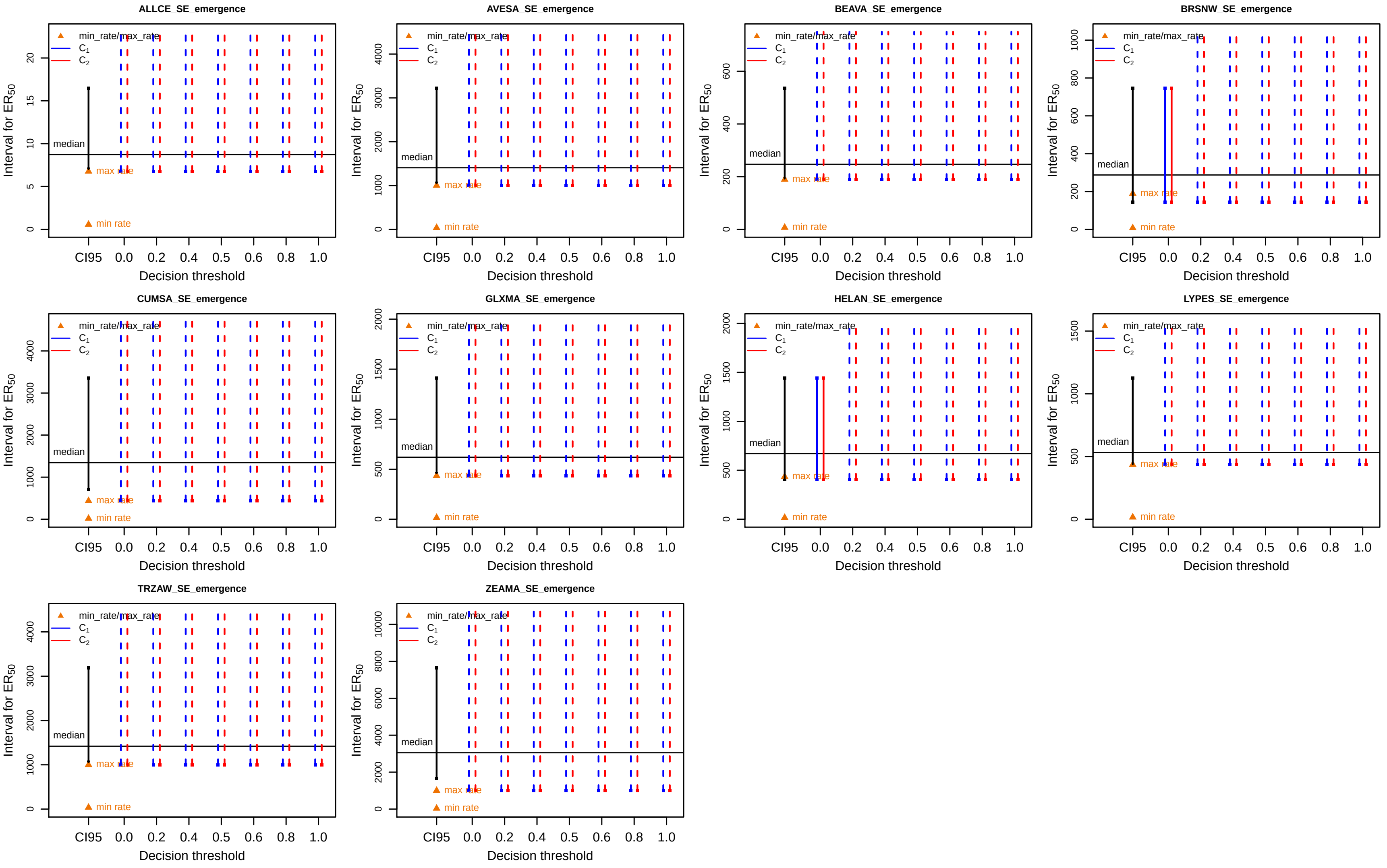

Figure 1: CI95 of ER_{50} and censored ER_{50} according to both criteria and the seven decision thresholds for all species - emergence endpoint of the SE test of study 4. The two orange triangles stand for the lowest and the highest tested rates. Vertical segments of three different colors (black, blue, red) represent the CI95 of ER_{50}, the censored ER_{50} according to C_1 and the censored ER_{50} according to C_2, respectively; solid vertical segments are for bounded intervals while dotted vertical segments stand for right-unbounded intervals; the black horizontal line represents the median of the ER_{50}.

SE test - survival endpoint

Table 5: Minimum and maximum tested rates, median, quantile 2.5% and quantile 97.5% of the ER_{50} posterior distribution - survival endpoint of the SE test of study 4

species	min_rate	max_rate	ER_{50}_median	ER_{50}_LCI95	ER_{50}_UCI95
ALLCE	0.56	6.76	8.766559	7.024317	16.15135
AVESA	35.73	1000.00	1029.936820	908.489654	1393.72141
BEAVA	6.76	189.04	91.197586	65.274126	137.67171
BRSNW	6.76	189.04	86.434567	65.437407	119.94335
CUMSA	15.54	434.78	1180.949935	628.530163	2904.80875
GLXMA	15.54	434.78	498.271721	385.881736	797.66190
HELAN	15.54	434.78	201.733229	156.996534	268.47073
LYPES	15.54	434.78	281.457792	197.888782	459.51627
TRZAW	35.73	1000.00	1461.707649	1072.711633	3253.78464
ZEAMA	35.73	1000.00	1100.902673	989.903499	1676.11282

Table 6: Censored ER_{50} according to criterion 1 and the seven decision thresholds - survival endpoint of the SE test of study 4

species	C1T0.0	C1T0.2	C1T0.4	C1T0.5	C1T0.6	C1T0.8	C1T1.0
ALLCE	[6.76; Inf)	[6.76; Inf)	[6.76; Inf)	[6.76; Inf)	[6.76; Inf)	[6.76; Inf)	[6.76; Inf)
AVESA	[908.49; 1393.72]	[908.49; 1393.72]	[908.49; Inf)	[908.49; Inf)	[908.49; Inf)	[908.49; Inf)	[908.49; Inf)
BEAVA	[65.27; 137.67]	[65.27; 137.67]	[65.27; 137.67]	[65.27; 137.67]	[65.27; 137.67]	[65.27; 137.67]	[65.27; 137.67]
BRSNW	[65.44; 119.94]	[65.44; 119.94]	[65.44; 119.94]	[65.44; 119.94]	[65.44; 119.94]	[65.44; 119.94]	[65.44; 119.94]
CUMSA	[434.78; Inf)	[434.78; Inf)	[434.78; Inf)	[434.78; Inf)	[434.78; Inf)	[434.78; Inf)	[434.78; Inf)
GLXMA	[385.88; 797.66]	[385.88; Inf)	[385.88; Inf)	[385.88; Inf)	[385.88; Inf)	[385.88; Inf)	[385.88; Inf)
HELAN	[157; 268.47]	[157; 268.47]	[157; 268.47]	[157; 268.47]	[157; 268.47]	[157; 268.47]	[157; 268.47]
LYPES	[197.89; 459.52]	[197.89; 459.52]	[197.89; 459.52]	[197.89; 459.52]	[197.89; 459.52]	[197.89; 459.52]	[197.89; Inf)
TRZAW	[1000; Inf)	[1000; Inf)	[1000; Inf)	[1000; Inf)	[1000; Inf)	[1000; Inf)	[1000; Inf)
ZEAMA	[989.9; 1676.11]	[989.9; Inf)	[989.9; Inf)	[989.9; Inf)	[989.9; Inf)	[989.9; Inf)	[989.9; Inf)

Table 7: Censored ER_{50} according to criterion 2 and the seven decision thresholds - survival endpoint of the SE test of study 4

species	C2T0.0	C2T0.2	C2T0.4	C2T0.5	C2T0.6	C2T0.8	C2T1.0
ALLCE	[6.76; Inf)	[6.76; Inf)	[6.76; Inf)	[6.76; Inf)	[6.76; Inf)	[6.76; Inf)	[6.76; Inf)
AVESA	[908.49; 1393.72]	[908.49; Inf)	[908.49; Inf)	[908.49; Inf)	[908.49; Inf)	[908.49; Inf)	[908.49; Inf)
BEAVA	[65.27; 137.67]	[65.27; 137.67]	[65.27; 137.67]	[65.27; 137.67]	[65.27; 137.67]	[65.27; 137.67]	[65.27; 137.67]
BRSNW	[65.44; 119.94]	[65.44; 119.94]	[65.44; 119.94]	[65.44; 119.94]	[65.44; 119.94]	[65.44; 119.94]	[65.44; 119.94]
CUMSA	[434.78; Inf)	[434.78; Inf)	[434.78; Inf)	[434.78; Inf)	[434.78; Inf)	[434.78; Inf)	[434.78; Inf)
GLXMA	[385.88; 797.66]	[385.88; Inf)	[385.88; Inf)	[385.88; Inf)	[385.88; Inf)	[385.88; Inf)	[385.88; Inf)
HELAN	[157; 268.47]	[157; 268.47]	[157; 268.47]	[157; 268.47]	[157; 268.47]	[157; 268.47]	[157; 268.47]
LYPES	[197.89; 459.52]	[197.89; 459.52]	[197.89; 459.52]	[197.89; 459.52]	[197.89; 459.52]	[197.89; 459.52]	[197.89; Inf)
TRZAW	[1000; Inf)	[1000; Inf)	[1000; Inf)	[1000; Inf)	[1000; Inf)	[1000; Inf)	[1000; Inf)
ZEAMA	[989.9; 1676.11]	[989.9; Inf)	[989.9; Inf)	[989.9; Inf)	[989.9; Inf)	[989.9; Inf)	[989.9; Inf)

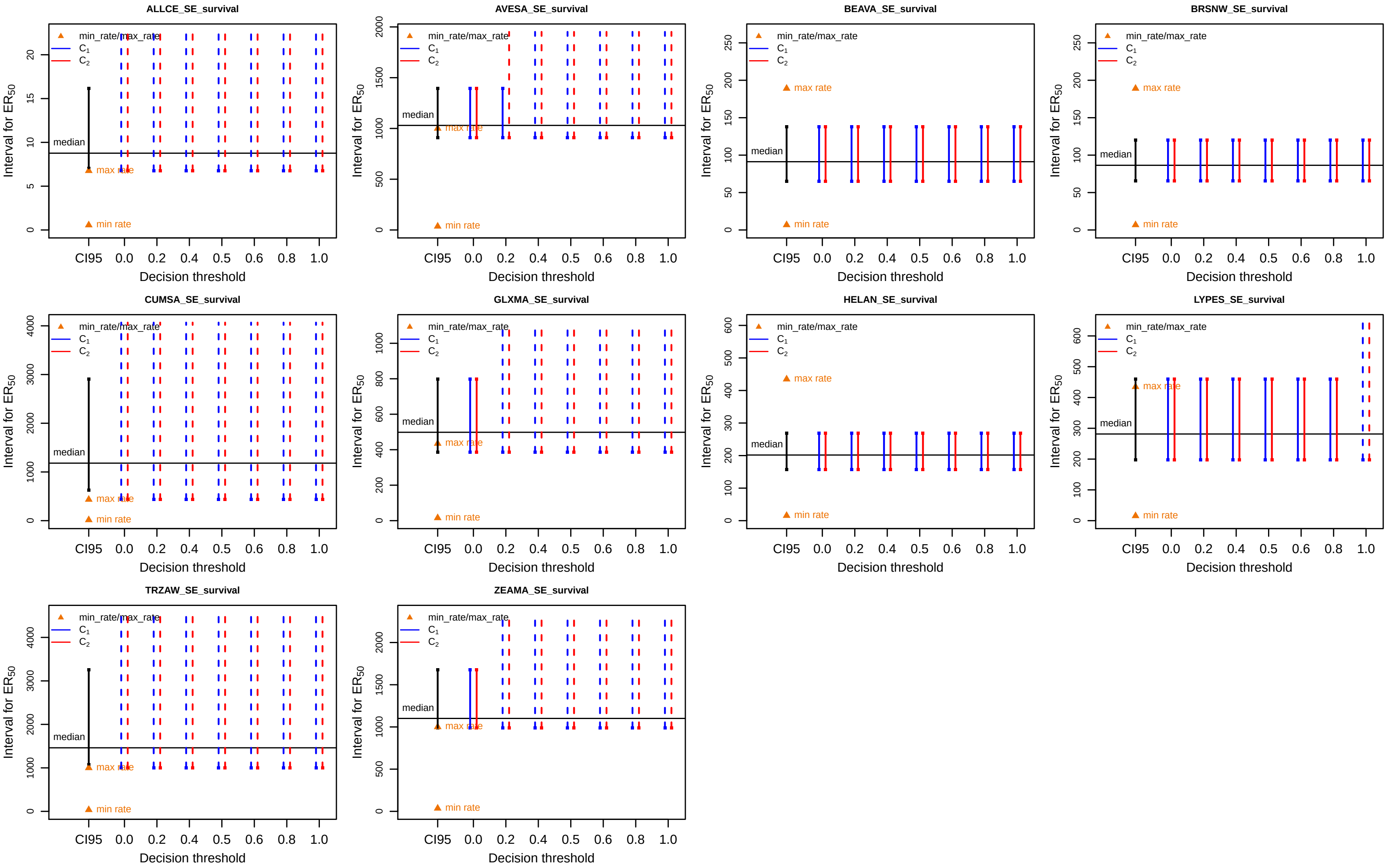

Figure 2: CI95 of ER_{50} and censored ER_{50} according to both criteria and the seven decision thresholds for all species - survival endpoint of the SE test of study 4. The two orange triangles stand for the lowest and the highest tested rates. Vertical segments of three different colors (black, blue, red) represent the CI95 of ER_{50}, the censored ER_{50} according to C_1 and the censored ER_{50} according to C_2, respectively; solid vertical segments are for bounded intervals while dotted vertical segments stand for right-unbounded intervals; the black horizontal line represents the median of the ER_{50}.

SE test - shoot dry weight endpoint

Table 8: Minimum and maximum tested rates, median, quantile 2.5% and quantile 97.5% of the ER_{50} posterior distribution - shoot dry weight endpoint of the SE test of study 4

species	min_rate	max_rate	ER_{50}_median	ER_{50}_LCI95	ER_{50}_UCI95
ALLCE	0.56	6.76	8.029177	1.188855	15.36391
AVESA	35.73	1000.00	110.670711	93.192080	130.30975
BEAVA	6.76	189.04	54.288835	41.533828	70.85636
BRSNW	6.76	189.04	44.903145	34.535775	61.39932
CUMSA	15.54	434.78	98.976576	80.277296	120.27585
GLXMA	15.54	434.78	186.608595	143.242485	237.51404
HELAN	15.54	434.78	69.388116	56.476707	84.42902
LYPES	15.54	434.78	42.106879	35.352712	52.33716
TRZAW	35.73	1000.00	880.597034	672.857523	1195.80341
ZEAMA	35.73	1000.00	302.849317	219.225441	417.38999

Table 9: Censored ER_{50} according to criterion 1 and the seven decision thresholds - shoot dry weight endpoint of the SE test of study 4

species	C1T0.0	C1T0.2	C1T0.4	C1T0.5	C1T0.6	C1T0.8	C1T1.0
ALLCE	[1.19; 15.36]	[1.19; Inf)	[1.19; Inf)	[1.19; Inf)	[1.19; Inf)	[1.19; Inf)	[1.19; Inf)
AVESA	[93.19; 130.31]	[93.19; 130.31]	[93.19; 130.31]	[93.19; 130.31]	[93.19; 130.31]	[93.19; 130.31]	[93.19; 130.31]
BEAVA	[41.53; 70.86]	[41.53; 70.86]	[41.53; 70.86]	[41.53; 70.86]	[41.53; 70.86]	[41.53; 70.86]	[41.53; 70.86]
BRSNW	[34.54; 61.4]	[34.54; 61.4]	[34.54; 61.4]	[34.54; 61.4]	[34.54; 61.4]	[34.54; 61.4]	[34.54; 61.4]
CUMSA	[80.28; 120.28]	[80.28; 120.28]	[80.28; 120.28]	[80.28; 120.28]	[80.28; 120.28]	[80.28; 120.28]	[80.28; 120.28]
GLXMA	[143.24; 237.51]	[143.24; 237.51]	[143.24; 237.51]	[143.24; 237.51]	[143.24; 237.51]	[143.24; 237.51]	[143.24; 237.51]
HELAN	[56.48; 84.43]	[56.48; 84.43]	[56.48; 84.43]	[56.48; 84.43]	[56.48; 84.43]	[56.48; 84.43]	[56.48; 84.43]
LYPES	[35.35; 52.34]	[35.35; 52.34]	[35.35; 52.34]	[35.35; 52.34]	[35.35; 52.34]	[35.35; 52.34]	[35.35; 52.34]
TRZAW	[672.86; 1195.8]	[672.86; 1195.8]	[672.86; 1195.8]	[672.86; 1195.8]	[672.86; 1195.8]	[672.86; 1195.8]	[672.86; Inf)
ZEAMA	[219.23; 417.39]	[219.23; 417.39]	[219.23; 417.39]	[219.23; 417.39]	[219.23; 417.39]	[219.23; 417.39]	[219.23; 417.39]

Table 10: Censored ER_{50} according to criterion 2 and the seven decision thresholds - shoot dry weight endpoint of the SE test of study 4

species	C2T0.0	C2T0.2	C2T0.4	C2T0.5	C2T0.6	C2T0.8	C2T1.0
ALLCE	[1.19; 15.36]	[1.19; 15.36]	[1.19; Inf)	[1.19; Inf)	[1.19; Inf)	[1.19; Inf)	[1.19; Inf)
AVESA	[93.19; 130.31]	[93.19; 130.31]	[93.19; 130.31]	[93.19; 130.31]	[93.19; 130.31]	[93.19; 130.31]	[93.19; 130.31]
BEAVA	[41.53; 70.86]	[41.53; 70.86]	[41.53; 70.86]	[41.53; 70.86]	[41.53; 70.86]	[41.53; 70.86]	[41.53; 70.86]
BRSNW	[34.54; 61.4]	[34.54; 61.4]	[34.54; 61.4]	[34.54; 61.4]	[34.54; 61.4]	[34.54; 61.4]	[34.54; 61.4]
CUMSA	[80.28; 120.28]	[80.28; 120.28]	[80.28; 120.28]	[80.28; 120.28]	[80.28; 120.28]	[80.28; 120.28]	[80.28; 120.28]
GLXMA	[143.24; 237.51]	[143.24; 237.51]	[143.24; 237.51]	[143.24; 237.51]	[143.24; 237.51]	[143.24; 237.51]	[143.24; 237.51]
HELAN	[56.48; 84.43]	[56.48; 84.43]	[56.48; 84.43]	[56.48; 84.43]	[56.48; 84.43]	[56.48; 84.43]	[56.48; 84.43]
LYPES	[35.35; 52.34]	[35.35; 52.34]	[35.35; 52.34]	[35.35; 52.34]	[35.35; 52.34]	[35.35; 52.34]	[35.35; 52.34]
TRZAW	[672.86; 1195.8]	[672.86; 1195.8]	[672.86; 1195.8]	[672.86; 1195.8]	[672.86; 1195.8]	[672.86; Inf)	[672.86; Inf)
ZEAMA	[219.23; 417.39]	[219.23; 417.39]	[219.23; 417.39]	[219.23; 417.39]	[219.23; 417.39]	[219.23; 417.39]	[219.23; 417.39]

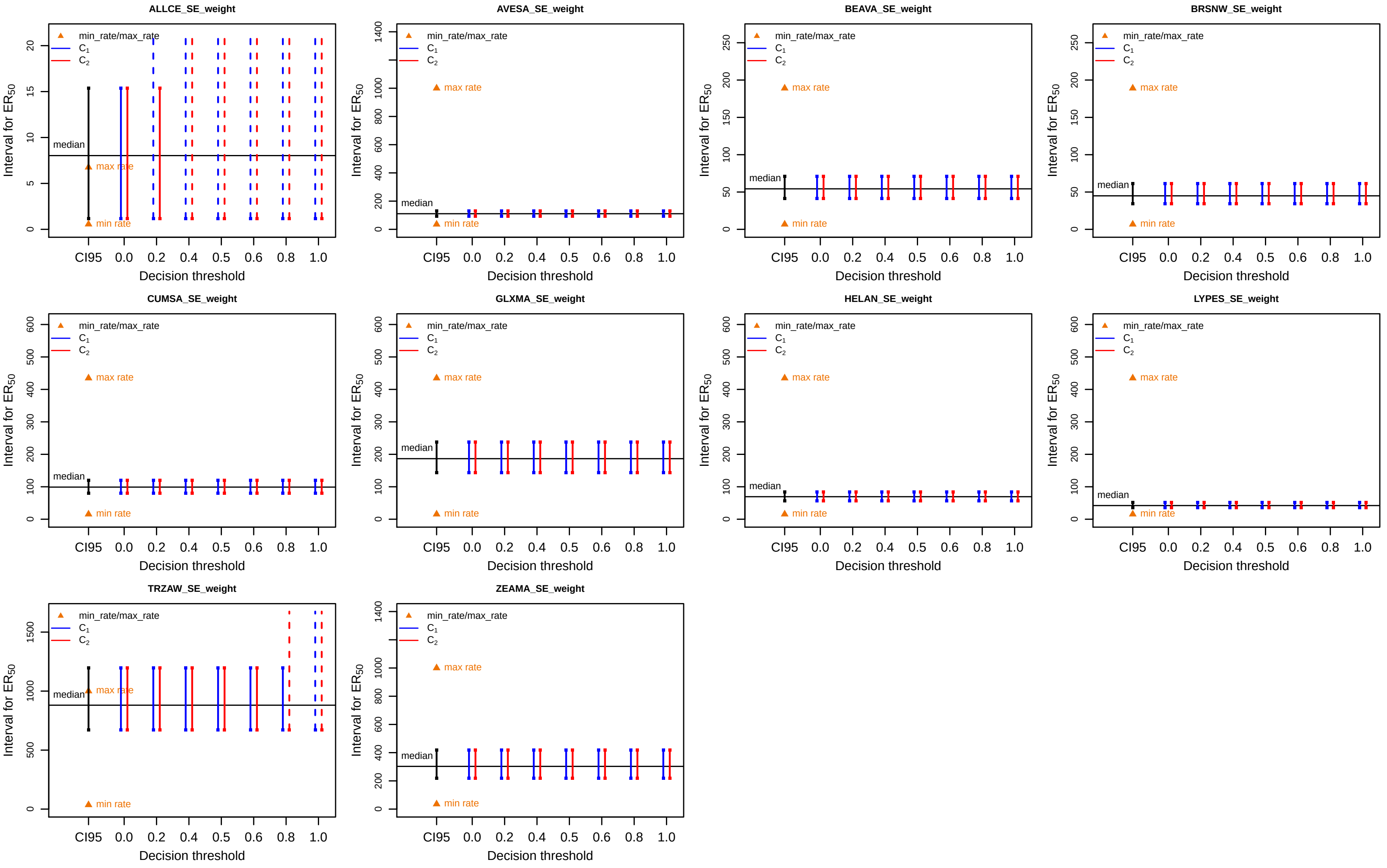

Figure 3: CI95 of ER_{50} and censored ER_{50} according to both criteria and the seven decision thresholds for all species - shoot dry weight endpoint of the SE test of study 4. The two orange triangles stand for the lowest and the highest tested rates. Vertical segments of three different colors (black, blue, red) represent the CI95 of ER_{50}, the censored ER_{50} according to C_1 and the censored ER_{50} according to C_2, respectively; solid vertical segments are for bounded intervals while dotted vertical segments stand for right-unbounded intervals; the black horizontal line represents the median of the ER_{50}.

Vegatative vigor (VV) test of study 4

Raw data description (test item rates) for VV test

```
Number of species:  10
EPPO code:  ALLCE AVESA BEAVA BRSNW CUMSA GLXMA HELAN LYPES TRZAW ZEAMA
```

Table 11: Corresponding species and common names to the EPPO code

EPPO code	Species name	Common name
ALLCE	*Allium cepa*	Onion
AVESA	*Avena sativa*	Oat
BEAVA	*Beta vulgaris*	Sugar beet
BRSNW	*Brassica napus winter*	Oilseed rape
CUMSA	*Cucumis sativus*	Cucumber
GLXMA	*Glycine max*	Soybean
HELAN	*Helianthus annuus*	Sunflower
LYPES	*Solanum lycopersicum*	Tomato
TRZAW	*Triticum aestivum winter*	Winter wheat
ZEAMA	*Zea mays subs. Amylacea*	Corn

VV test - survival endpoint

Table 12: Minimum and maximum tested rates, median, quantile 2.5% and quantile 97.5% of the ER_{50} posterior distribution - survival endpoint of the VV test of study 4

species	min_rate	max_rate	ER_{50}_median	ER_{50}_LCI95	ER_{50}_UCI95
ALLCE	15.54	434.78	459.04671	278.56665	980.24652
AVESA	35.73	1000.00	446.87709	406.81364	524.02506
BEAVA	6.76	189.04	122.09935	79.74769	215.88531
BRSNW	6.76	189.04	239.51344	175.27606	443.41989
CUMSA	1.28	434.78	521.83495	329.41783	1139.96972
GLXMA	15.54	434.78	377.47383	300.89074	427.58637
HELAN	15.54	434.78	67.16214	50.99899	79.27503
LYPES	1.28	434.78	129.54932	104.79857	159.33122
TRZAW	35.73	1000.00	1466.79538	1075.00672	3369.87270
ZEAMA	35.73	1000.00	1219.00940	1022.24357	2299.02627

Table 13: Censored ER_{50} according to criterion 1 and the seven decision thresholds - survival endpoint of the VV test of study 4

species	C1T0.0	C1T0.2	C1T0.4	C1T0.5	C1T0.6	C1T0.8	C1T1.0
ALLCE	[278.57; 980.25]	[278.57; 980.25]	[278.57; 980.25]	[278.57; Inf)	[278.57; Inf)	[278.57; Inf)	[278.57; Inf)
AVESA	[406.81; 524.03]	[406.81; 524.03]	[406.81; 524.03]	[406.81; 524.03]	[406.81; 524.03]	[406.81; 524.03]	[406.81; 524.03]
BEAVA	[79.75; 215.89]	[79.75; 215.89]	[79.75; 215.89]	[79.75; 215.89]	[79.75; 215.89]	[79.75; 215.89]	[79.75; Inf)
BRSNW	[175.28; 443.42]	[175.28; Inf)	[175.28; Inf)	[175.28; Inf)	[175.28; Inf)	[175.28; Inf)	[175.28; Inf)
CUMSA	[329.42; 1139.97]	[329.42; 1139.97]	[329.42; Inf)	[329.42; Inf)	[329.42; Inf)	[329.42; Inf)	[329.42; Inf)
GLXMA	[300.89; 427.59]	[300.89; 427.59]	[300.89; 427.59]	[300.89; 427.59]	[300.89; 427.59]	[300.89; 427.59]	[300.89; 427.59]
HELAN	[51; 79.28]	[51; 79.28]	[51; 79.28]	[51; 79.28]	[51; 79.28]	[51; 79.28]	[51; 79.28]
LYPES	[104.8; 159.33]	[104.8; 159.33]	[104.8; 159.33]	[104.8; 159.33]	[104.8; 159.33]	[104.8; 159.33]	[104.8; 159.33]
TRZAW	[1000; Inf)	[1000; Inf)	[1000; Inf)	[1000; Inf)	[1000; Inf)	[1000; Inf)	[1000; Inf)
ZEAMA	[1000; Inf)	[1000; Inf)	[1000; Inf)	[1000; Inf)	[1000; Inf)	[1000; Inf)	[1000; Inf)

Table 14: Censored ER_{50} according to criterion 2 and the seven decision thresholds - survival endpoint of the VV test of study 4

species	C2T0.0	C2T0.2	C2T0.4	C2T0.5	C2T0.6	C2T0.8	C2T1.0
ALLCE	[278.57; 980.25]	[278.57; 980.25]	[278.57; Inf)	[278.57; Inf)	[278.57; Inf)	[278.57; Inf)	[278.57; Inf)
AVESA	[406.81; 524.03]	[406.81; 524.03]	[406.81; 524.03]	[406.81; 524.03]	[406.81; 524.03]	[406.81; 524.03]	[406.81; 524.03]
BEAVA	[79.75; 215.89]	[79.75; 215.89]	[79.75; 215.89]	[79.75; 215.89]	[79.75; 215.89]	[79.75; 215.89]	[79.75; Inf)
BRSNW	[175.28; 443.42]	[175.28; Inf)	[175.28; Inf)	[175.28; Inf)	[175.28; Inf)	[175.28; Inf)	[175.28; Inf)
CUMSA	[329.42; 1139.97]	[329.42; Inf)	[329.42; Inf)	[329.42; Inf)	[329.42; Inf)	[329.42; Inf)	[329.42; Inf)
GLXMA	[300.89; 427.59]	[300.89; 427.59]	[300.89; 427.59]	[300.89; 427.59]	[300.89; 427.59]	[300.89; 427.59]	[300.89; 427.59]
HELAN	[51; 79.28]	[51; 79.28]	[51; 79.28]	[51; 79.28]	[51; 79.28]	[51; 79.28]	[51; 79.28]
LYPES	[104.8; 159.33]	[104.8; 159.33]	[104.8; 159.33]	[104.8; 159.33]	[104.8; 159.33]	[104.8; 159.33]	[104.8; 159.33]
TRZAW	[1000; Inf)	[1000; Inf)	[1000; Inf)	[1000; Inf)	[1000; Inf)	[1000; Inf)	[1000; Inf)
ZEAMA	[1000; Inf)	[1000; Inf)	[1000; Inf)	[1000; Inf)	[1000; Inf)	[1000; Inf)	[1000; Inf)

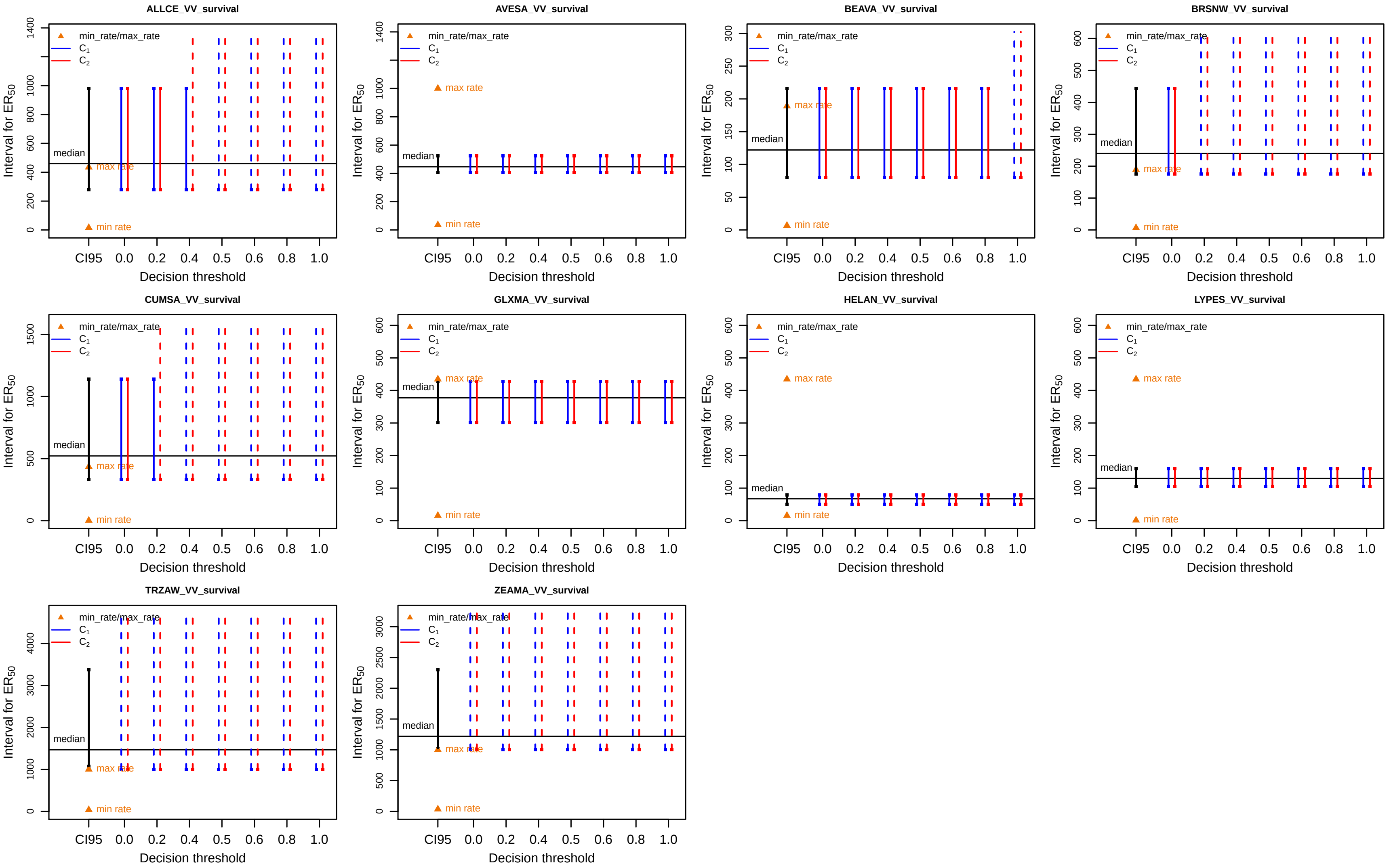

Figure 4: CI95 of ER_{50} and censored ER_{50} according to both criteria and the seven decision thresholds for all species - survival endpoint of the VV test of study 4. The two orange triangles stand for the lowest and the highest tested rates. Vertical segments of three different colors (black, blue, red) represent the CI95 of ER_{50}, the censored ER_{50} according to C_1 and the censored ER_{50} according to C_2, respectively; solid vertical segments are for bounded intervals while dotted vertical segments stand for right-unbounded intervals; the black horizontal line represents the median of the ER_{50}.

VV test - shoot dry weight endpoint

Table 15: Minimum and maximum tested rates, median, quantile 2.5% and quantile 97.5% of the ER_{50} posterior distribution - shoot dry weight endpoint of the VV test of study 4

species	min_rate	max_rate	ER_{50}_median	ER_{50}_LCI95	ER_{50}_UCI95
ALLCE	15.54	434.78	150.608832	52.123408	370.24495
AVESA	35.73	434.78	115.250100	101.207736	131.44158
BEAVA	6.76	189.04	8.573542	6.797297	10.42677
BRSNW	6.76	189.04	19.299001	17.088727	21.94969
CUMSA	1.28	434.78	16.930835	13.965220	20.65933
GLXMA	15.54	434.78	33.033028	29.031360	37.89127
HELAN	15.54	82.19	23.906723	20.713573	27.80414
LYPES	1.28	189.04	15.171551	14.137252	15.60803
TRZAW	35.73	1000.00	2036.537531	1042.973486	4831.95673
ZEAMA	35.73	1000.00	403.883960	367.325215	443.15490

Table 16: Censored ER_{50} according to criterion 1 and the seven decision thresholds - shoot dry weight endpoint of the VV test of study 4

species	C1T0.0	C1T0.2	C1T0.4	C1T0.5	C1T0.6	C1T0.8	C1T1.0
ALLCE	[52.12; 370.24]	[52.12; 370.24]	[52.12; 370.24]	[52.12; 370.24]	[52.12; 370.24]	[52.12; 370.24]	[52.12; 370.24]
AVESA	[101.21; 131.44]	[101.21; 131.44]	[101.21; 131.44]	[101.21; 131.44]	[101.21; 131.44]	[101.21; 131.44]	[101.21; 131.44]
BEAVA	[6.8; 10.43]	[6.8; 10.43]	[6.8; 10.43]	[6.8; 10.43]	[6.8; 10.43]	[6.8; 10.43]	[6.8; 10.43]
BRSNW	[17.09; 21.95]	[17.09; 21.95]	[17.09; 21.95]	[17.09; 21.95]	[17.09; 21.95]	[17.09; 21.95]	[17.09; 21.95]
CUMSA	[13.97; 20.66]	[13.97; 20.66]	[13.97; 20.66]	[13.97; 20.66]	[13.97; 20.66]	[13.97; 20.66]	[13.97; 20.66]
GLXMA	[29.03; 37.89]	[29.03; 37.89]	[29.03; 37.89]	[29.03; 37.89]	[29.03; 37.89]	[29.03; 37.89]	[29.03; 37.89]
HELAN	[20.71; 27.8]	[20.71; 27.8]	[20.71; 27.8]	[20.71; 27.8]	[20.71; 27.8]	[20.71; 27.8]	[20.71; 27.8]
LYPES	[14.14; 15.61]	[14.14; 15.61]	[14.14; 15.61]	[14.14; 15.61]	[14.14; 15.61]	[14.14; 15.61]	[14.14; 15.61]
TRZAW	[1000; Inf)	[1000; Inf)	[1000; Inf)	[1000; Inf)	[1000; Inf)	[1000; Inf)	[1000; Inf)
ZEAMA	[367.33; 443.15]	[367.33; 443.15]	[367.33; 443.15]	[367.33; 443.15]	[367.33; 443.15]	[367.33; 443.15]	[367.33; 443.15]

Table 17: Censored ER_{50} according to criterion 2 and the seven decision thresholds - shoot dry weight endpoint of the VV test of study 4

species	C2T0.0	C2T0.2	C2T0.4	C2T0.5	C2T0.6	C2T0.8	C2T1.0
ALLCE	[52.12; 370.24]	[52.12; 370.24]	[52.12; 370.24]	[52.12; 370.24]	[52.12; 370.24]	[52.12; 370.24]	[52.12; 370.24]
AVESA	[101.21; 131.44]	[101.21; 131.44]	[101.21; 131.44]	[101.21; 131.44]	[101.21; 131.44]	[101.21; 131.44]	[101.21; 131.44]
BEAVA	[6.8; 10.43]	[6.8; 10.43]	[6.8; 10.43]	[6.8; 10.43]	[6.8; 10.43]	[6.8; 10.43]	[6.8; 10.43]
BRSNW	[17.09; 21.95]	[17.09; 21.95]	[17.09; 21.95]	[17.09; 21.95]	[17.09; 21.95]	[17.09; 21.95]	[17.09; 21.95]
CUMSA	[13.97; 20.66]	[13.97; 20.66]	[13.97; 20.66]	[13.97; 20.66]	[13.97; 20.66]	[13.97; 20.66]	[13.97; 20.66]
GLXMA	[29.03; 37.89]	[29.03; 37.89]	[29.03; 37.89]	[29.03; 37.89]	[29.03; 37.89]	[29.03; 37.89]	[29.03; 37.89]
HELAN	[20.71; 27.8]	[20.71; 27.8]	[20.71; 27.8]	[20.71; 27.8]	[20.71; 27.8]	[20.71; 27.8]	[20.71; 27.8]
LYPES	[14.14; 15.61]	[14.14; 15.61]	[14.14; 15.61]	[14.14; 15.61]	[14.14; 15.61]	[14.14; 15.61]	[14.14; 15.61]
TRZAW	[1000; Inf)	[1000; Inf)	[1000; Inf)	[1000; Inf)	[1000; Inf)	[1000; Inf)	[1000; Inf)
ZEAMA	[367.33; 443.15]	[367.33; 443.15]	[367.33; 443.15]	[367.33; 443.15]	[367.33; 443.15]	[367.33; 443.15]	[367.33; 443.15]

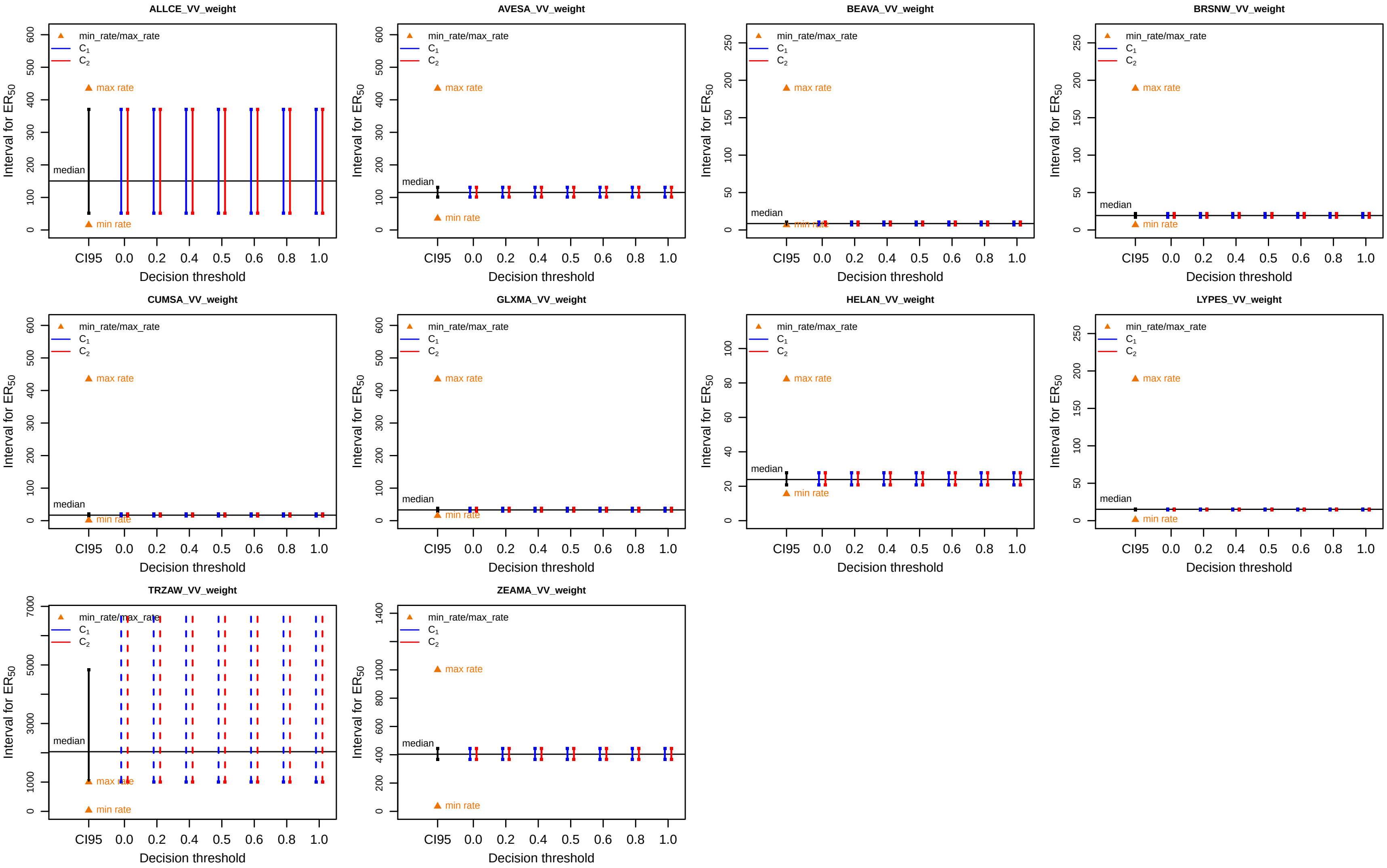

Figure 5: CI95 of ER_{50} and censored ER_{50} according to both criteria and the seven decision thresholds for all species - shoot dry weight endpoint of the VV test of study 4. The two orange triangles stand for the lowest and the highest tested rates. Vertical segments of three different colors (black, blue, red) represent the CI95 of ER_{50}, the censored ER_{50} according to C_1 and the censored ER_{50} according to C_2, respectively; solid vertical segments are for bounded intervals while dotted vertical segments stand for right-unbounded intervals; the black horizontal line represents the median of the ER_{50}.
