# Supplementary material for: How to account for the uncertainty from standard toxicity tests in species sensitivity distributions: An example in non-target plants
Source: PLoS One. 2021 Jan 7;16(1):e0245071. doi: 10.1371/journal.pone.0245071 (PMC7790375; doi:10.1371/journal.pone.0245071)
Supplement: S1 Archive — It is a zip file containing seven folders (one folder per case study). Each folder contains five files report_xxx.pdf with detailed results of the dose-response analyses, one file corresponding to does-response analysis per endpoint. It also contains one file ER50_censoring.pdf for censored ER50 and one file SSD_analyses.pdf for results of SSD analyses. (ZIP) [file pone.0245071.s004.zip › S1_archive/Study4/report_SE_emergence.pdf]

Dose-response analyses

Study 4

Seedling Emergence test - emergence endpoint

25 June 2020

Contact: sandrine.charles@univ-lyon1.fr

This is a report which provides results on all performed dose-response analyses for the emergence endpoint of the Seedling Emergence test for study 4.

Contents

Data set: ALLCE_SE_emergence . . . 2
Data set: AVESA_SE_emergence . . . 3
Data set: BEAVA_SE_emergence . . . 4
Data set: BRSNW_SE_emergence . . . 5
Data set: CUMSA_SE_emergence . . . 6
Data set: GLXMA_SE_emergence . . . 7
Data set: HELAN_SE_emergence . . . 8
Data set: LYPES_SE_emergence . . . 9
Data set: TRZAW_SE_emergence . . . 10
Data set: ZEAMA_SE_emergence . . . 11

Data set: ALLCE_SE_emergence

Table 1: Summary of parameter estimates for ALLCE_SE_emergence data set

Parameter	median	Q2.5	Q97.5
b	28.304	3.314	94.325
d	0.741	0.649	0.823
e	8.737	7.038	16.469

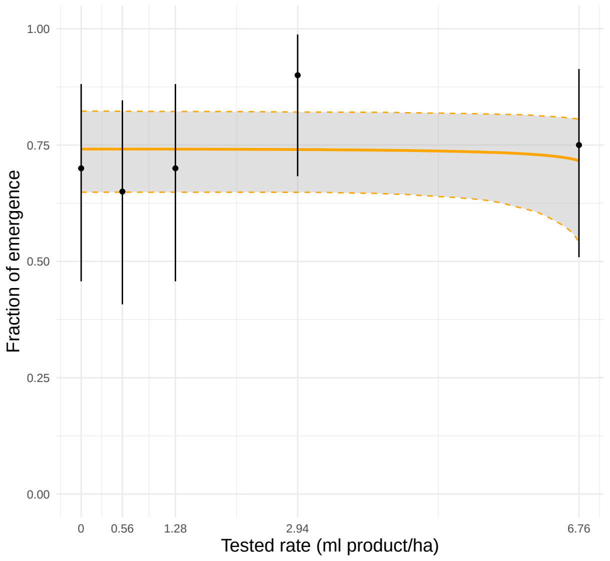

(a) Dose-response curve

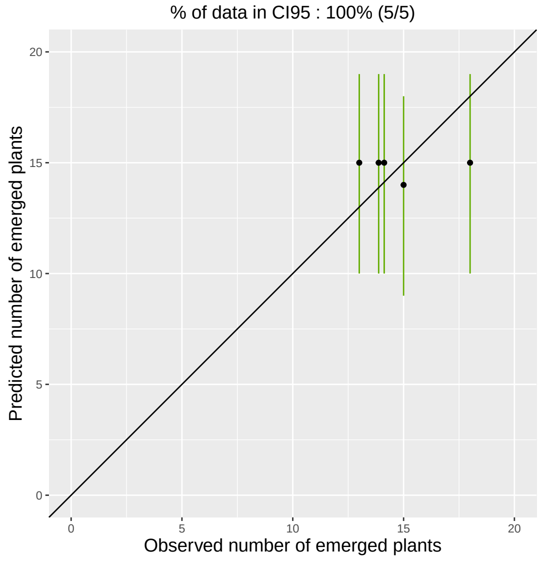

(b) Posterior predictive check (PPC)

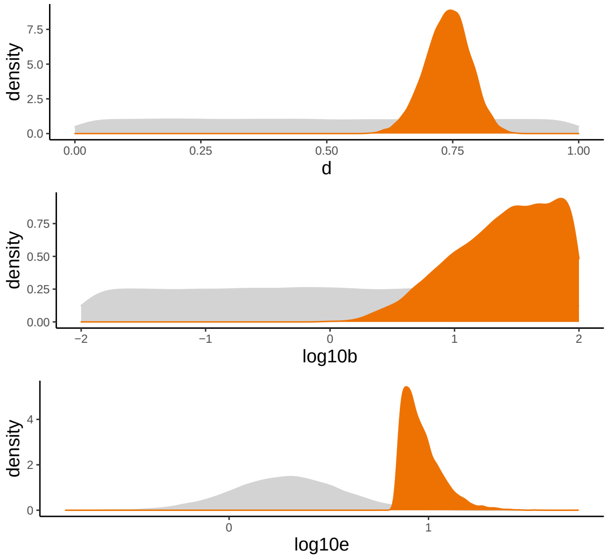

(c) Priors and posteriors

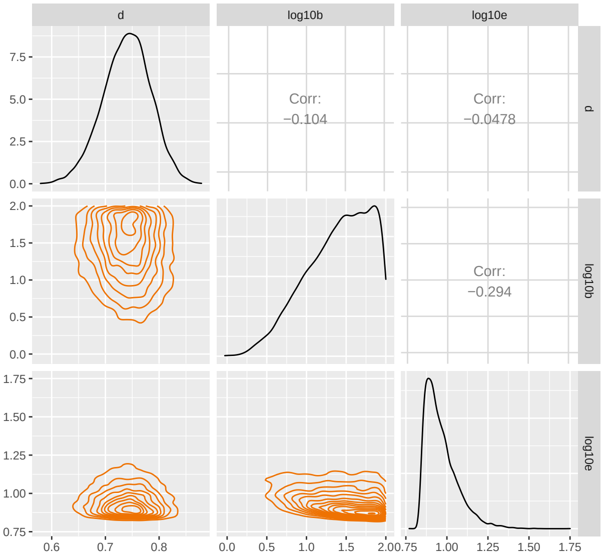

(d) Correlations between parameters

Figure 1: Dose-response curve (a), PPC (b), prior and posterior distributions (c) and correlations between parameters (d).

Data set: AVESA_SE_emergence

Table 2: Summary of parameter estimates for AVESA_SE_emergence data set

Parameter	median	Q2.5	Q97.5
b	26.782	3.558	93.486
d	0.972	0.931	0.993
e	1405.380	1051.828	3219.676

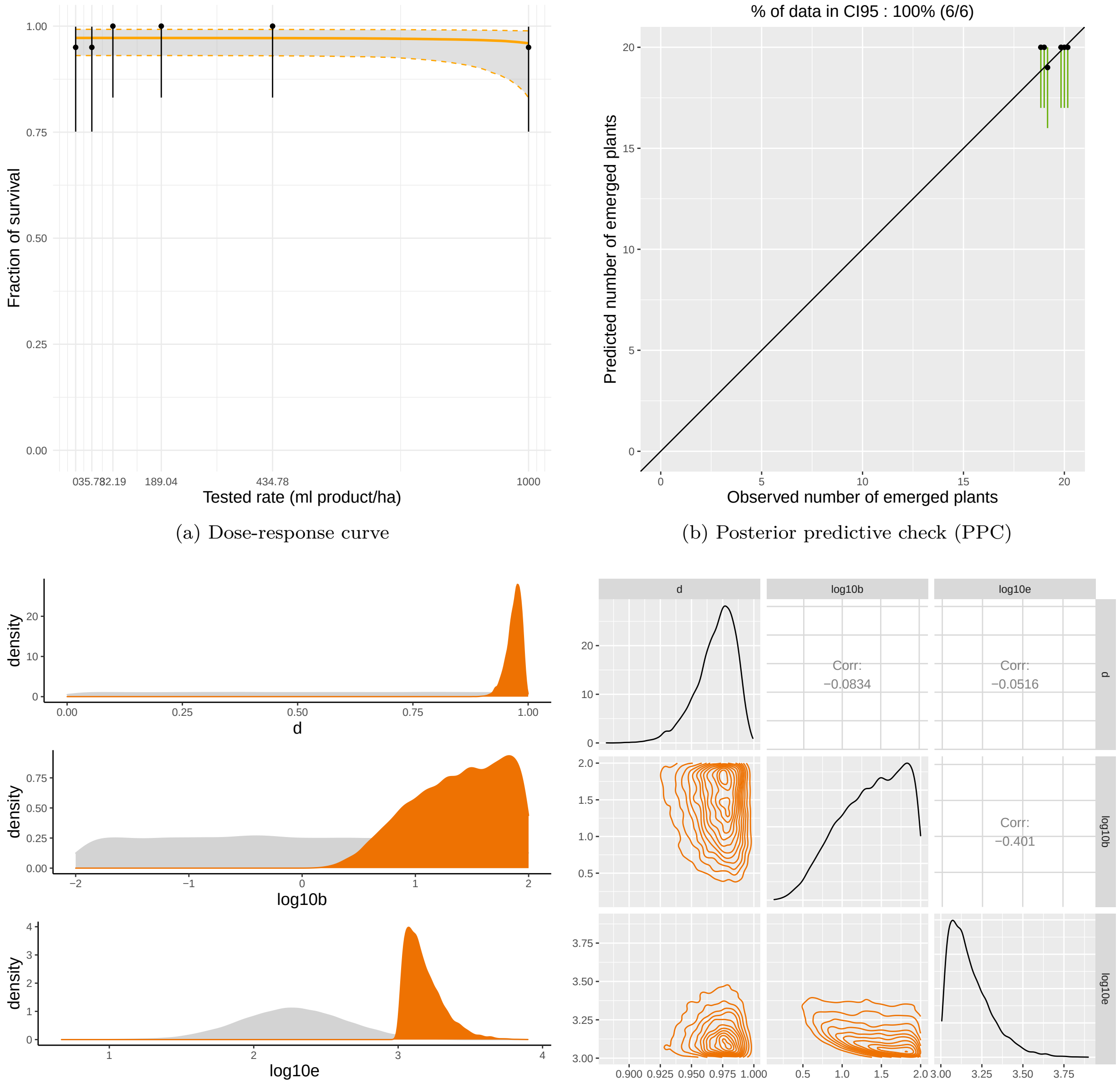

(a) Dose-response curve

(b) Posterior predictive check (PPC)

(c) Priors and posteriors

(d) Correlations between parameters

Figure 2: Dose-response curve (a), PPC (b), prior and posterior distributions (c) and correlations between parameters (d).

Data set: BEAVA_SE_emergence

Table 3: Summary of parameter estimates for BEAVA_SE_emergence data set

Parameter	median	Q2.5	Q97.5
b	5.403	1.157	77.886
d	0.918	0.847	0.972
e	246.873	192.773	535.733

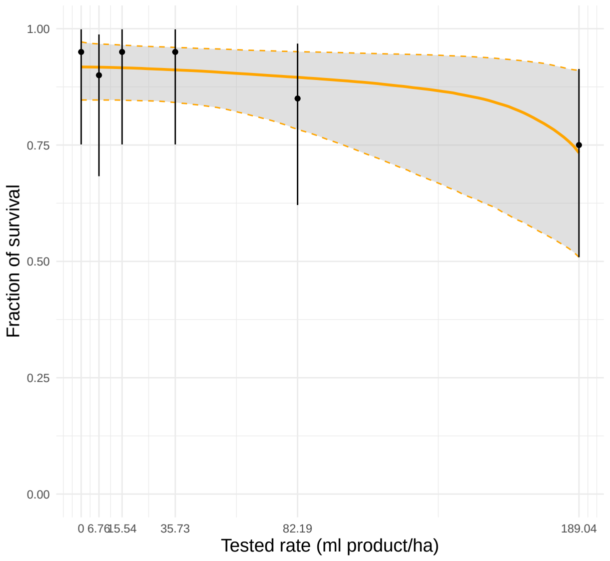

(a) Dose-response curve

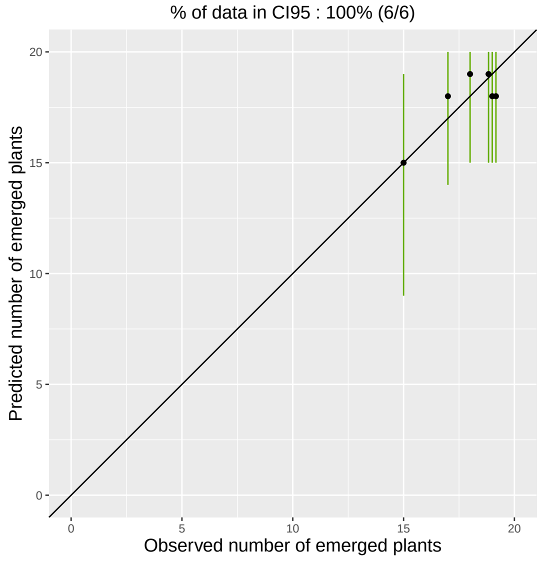

(b) Posterior predictive check (PPC)

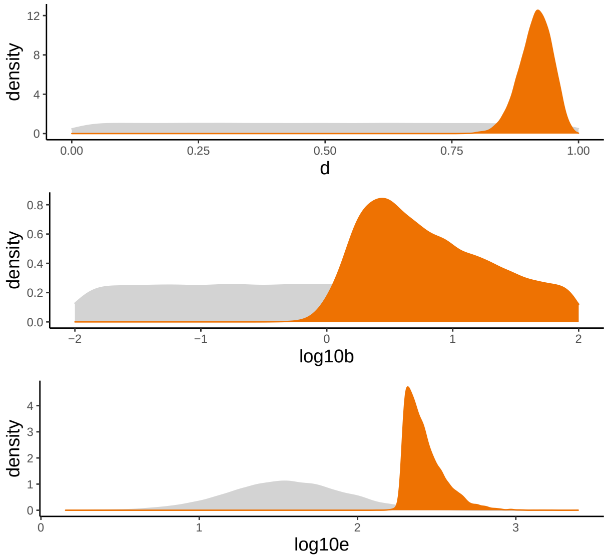

(c) Priors and posteriors

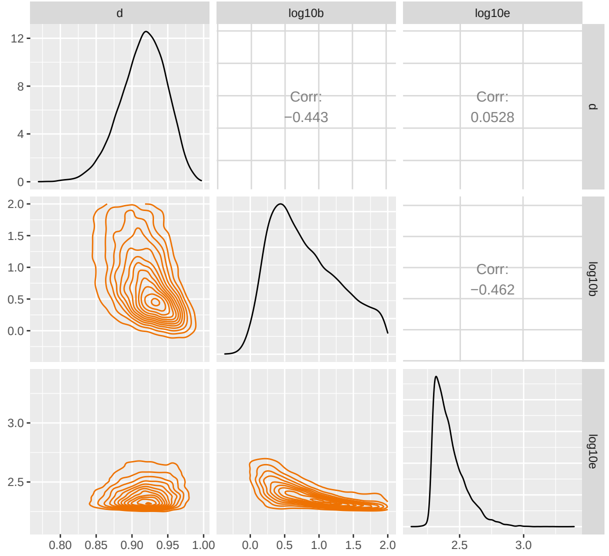

(d) Correlations between parameters

Figure 3: Dose-response curve (a), PPC (b), prior and posterior distributions (c) and correlations between parameters (d).

Data set: BRSNW_SE_emergence

Table 4: Summary of parameter estimates (parameter d is set to 1) for BRSNW_SE_emergence data set

Parameter	median	Q2.5	Q97.5
b	0.669	0.395	1.043
e	287.261	143.556	745.987

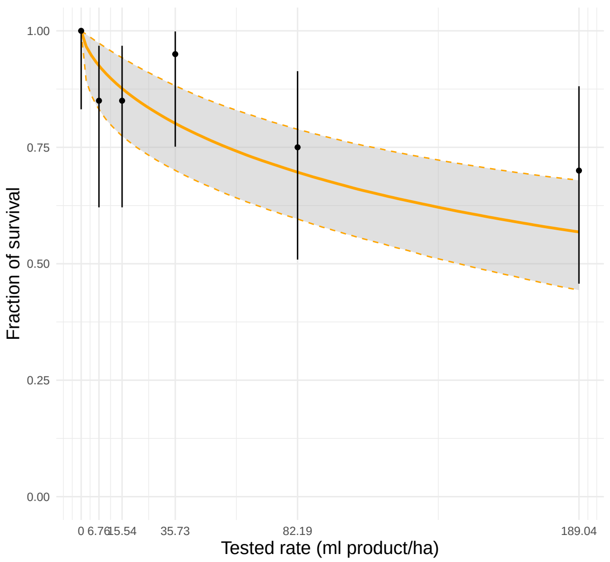

(a) Dose-response curve

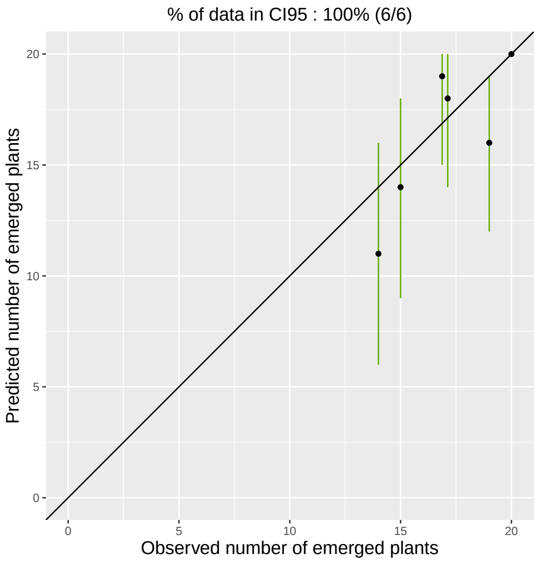

(b) Posterior predictive check (PPC)

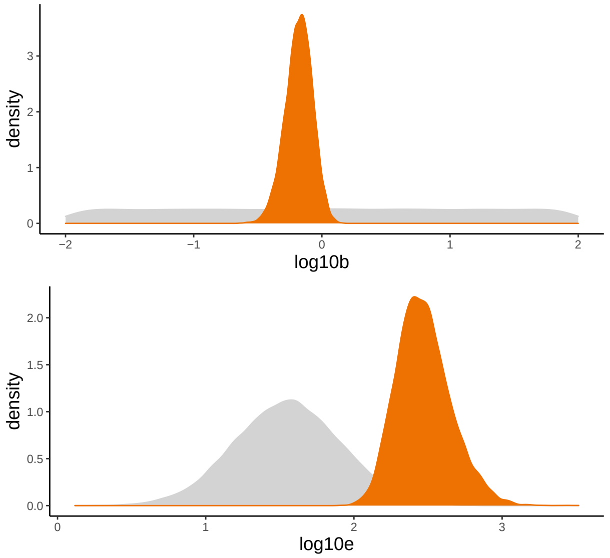

(c) Priors and posteriors

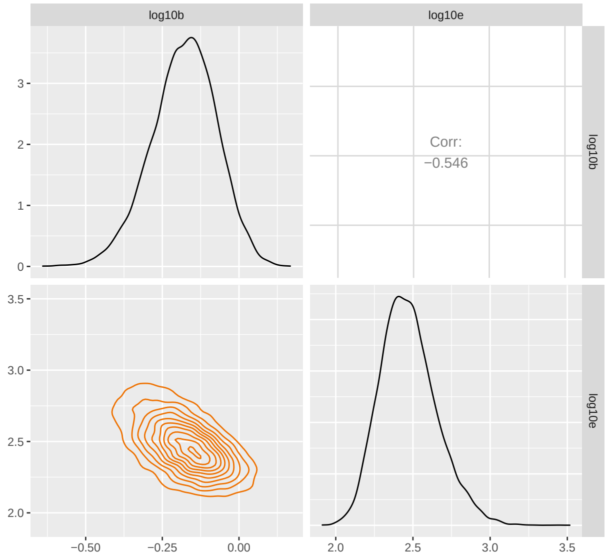

(d) Correlations between parameters

Figure 4: Dose-response curve (a), PPC (b), prior and posterior distributions (c) and correlations between parameters (d).

Data set: CUMSA_SE_emergence

Table 5: Summary of parameter estimates (parameter d is set to 1) for CUMSA_SE_emergence data set

Parameter	median	Q2.5	Q97.5
b	1.295	0.815	2.063
e	1344.514	705.783	3354.320

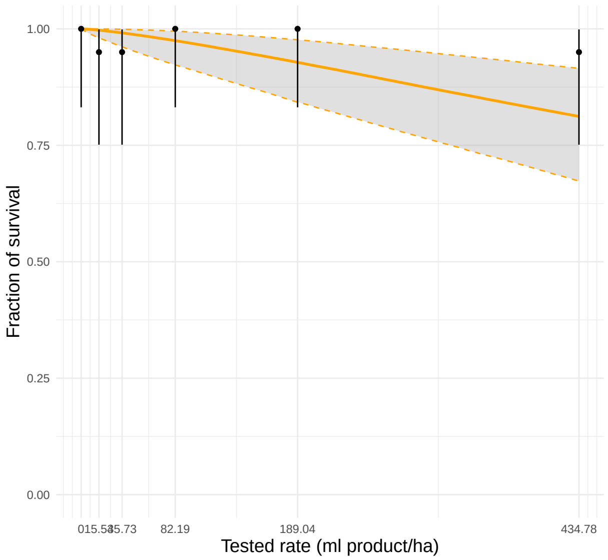

(a) Dose-response curve

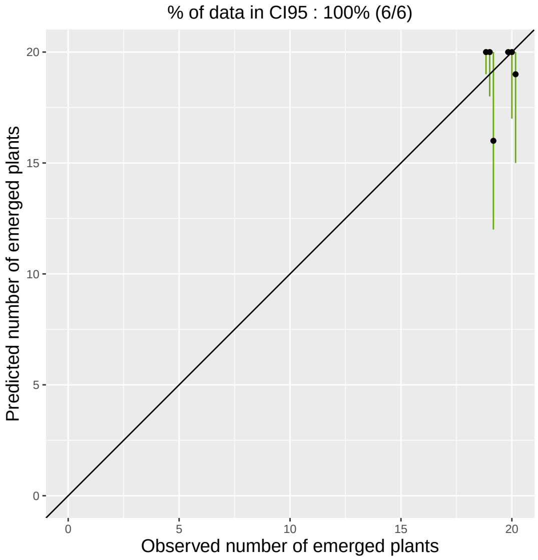

(b) Posterior predictive check (PPC)

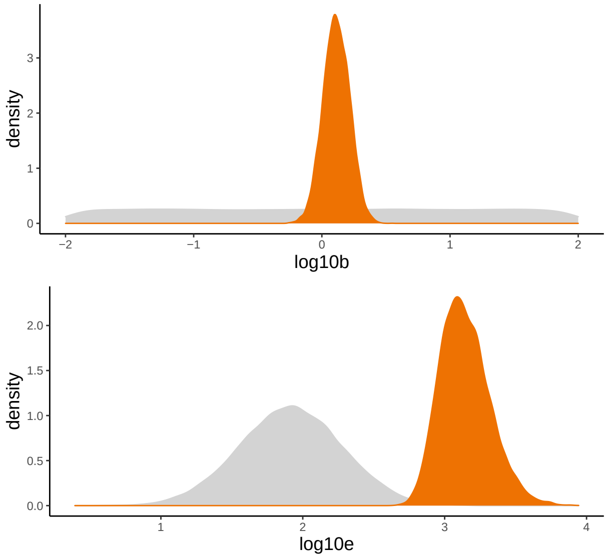

(c) Priors and posteriors

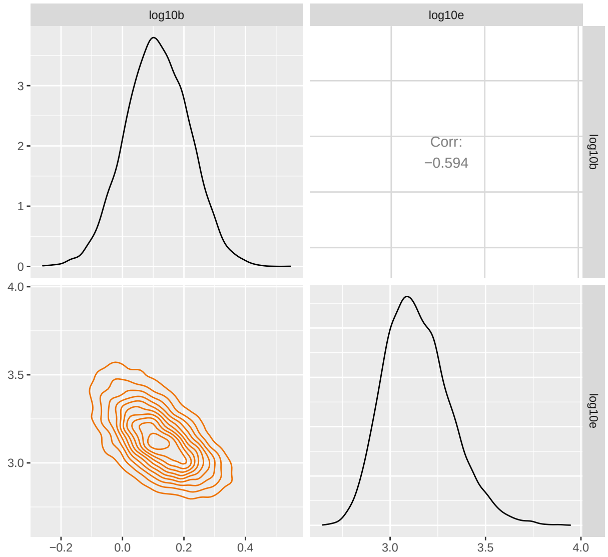

(d) Correlations between parameters

Figure 5: Dose-response curve (a), PPC (b), prior and posterior distributions (c) and correlations between parameters (d).

Data set: GLXMA_SE_emergence

Table 6: Summary of parameter estimates for GLXMA_SE_emergence data set

Parameter	median	Q2.5	Q97.5
b	28.030	3.726	94.461
d	0.963	0.919	0.988
e	619.582	459.193	1410.823

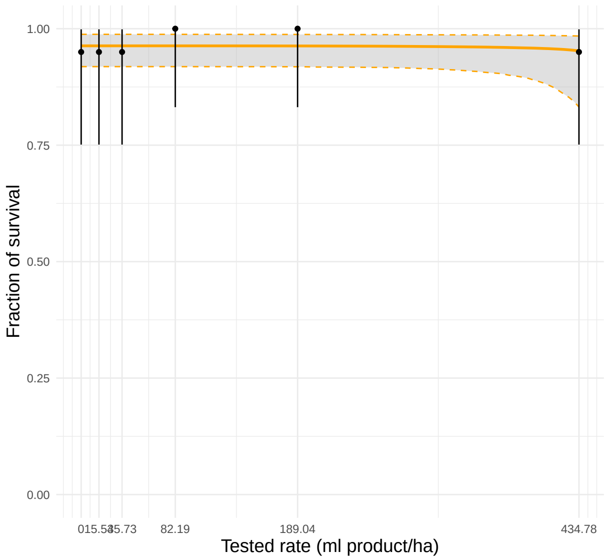

(a) Dose-response curve

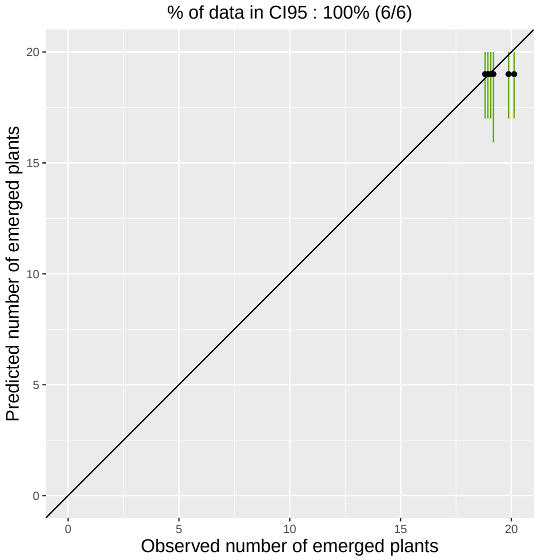

(b) Posterior predictive check (PPC)

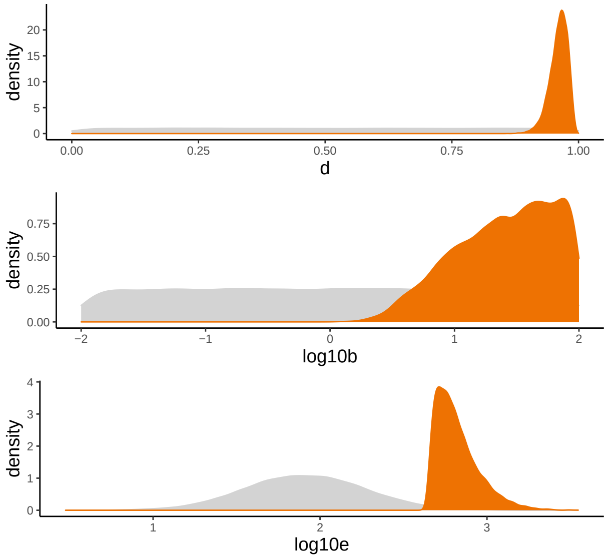

(c) Priors and posteriors

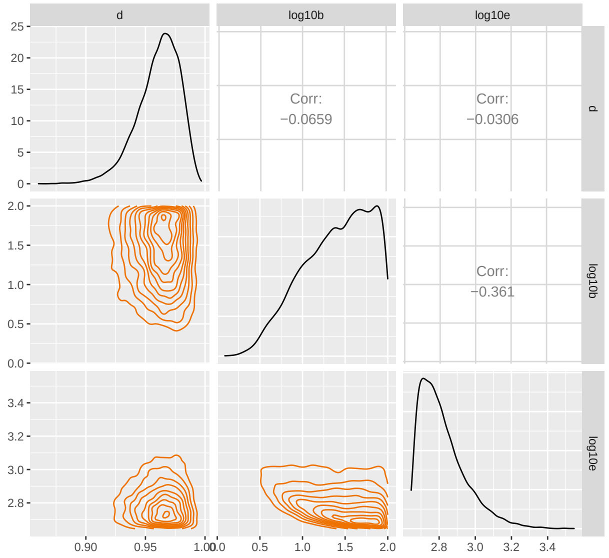

(d) Correlations between parameters

Figure 6: Dose-response curve (a), PPC (b), prior and posterior distributions (c) and correlations between parameters (d).

Data set: HELAN_SE_emergence

Table 7: Summary of parameter estimates (parameter d is set to 1) for HELAN_SE_emergence data set

Parameter	median	Q2.5	Q97.5
b	1.302	0.791	2.103
e	670.562	407.864	1441.417

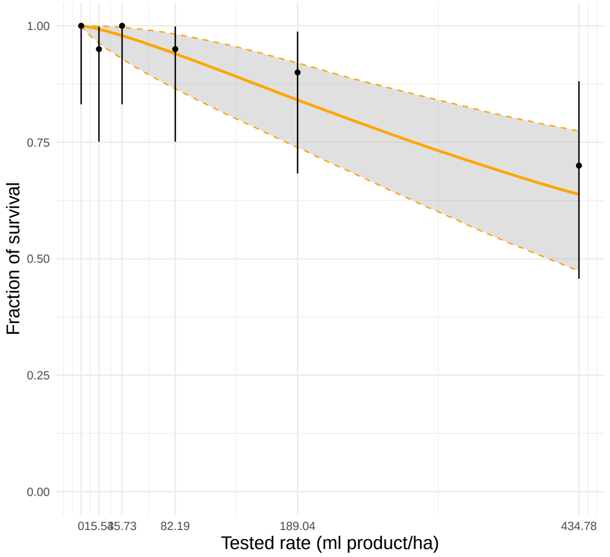

(a) Dose-response curve

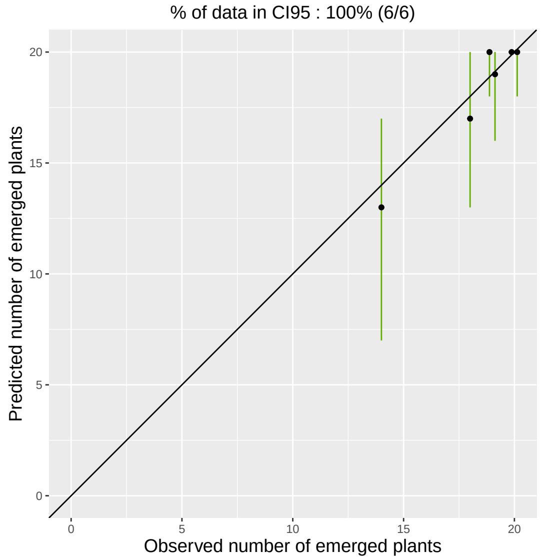

(b) Posterior predictive check (PPC)

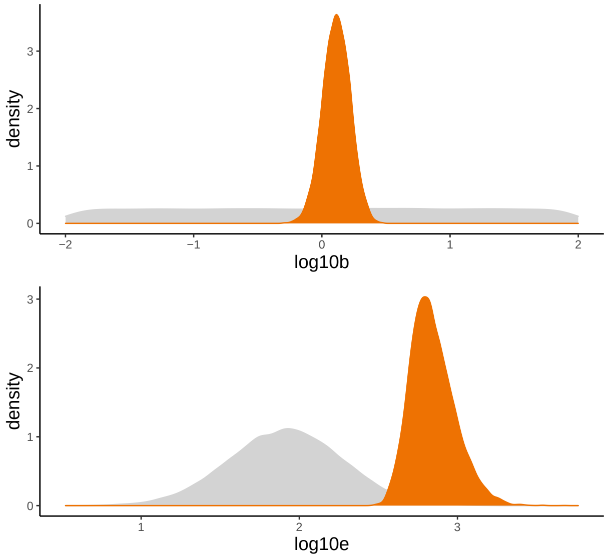

(c) Priors and posteriors

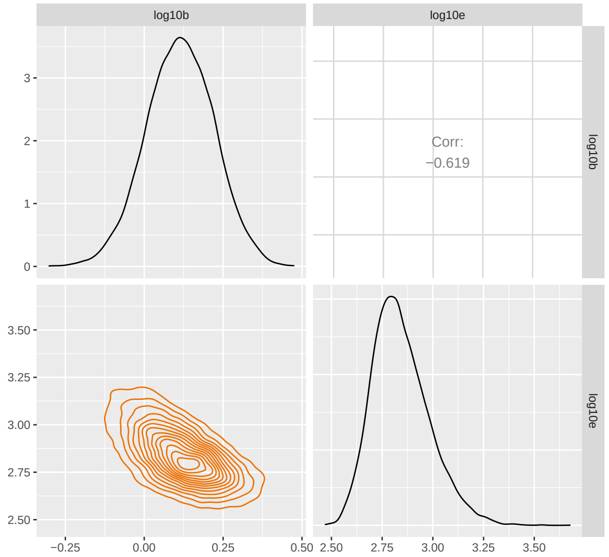

(d) Correlations between parameters

Figure 7: Dose-response curve (a), PPC (b), prior and posterior distributions (c) and correlations between parameters (d).

Data set: LYPES_SE_emergence

Table 8: Summary of parameter estimates for LYPES_SE_emergence data set

Parameter	median	Q2.5	Q97.5
b	6.959	1.423	75.728
d	0.893	0.820	0.948
e	532.938	439.202	1124.719

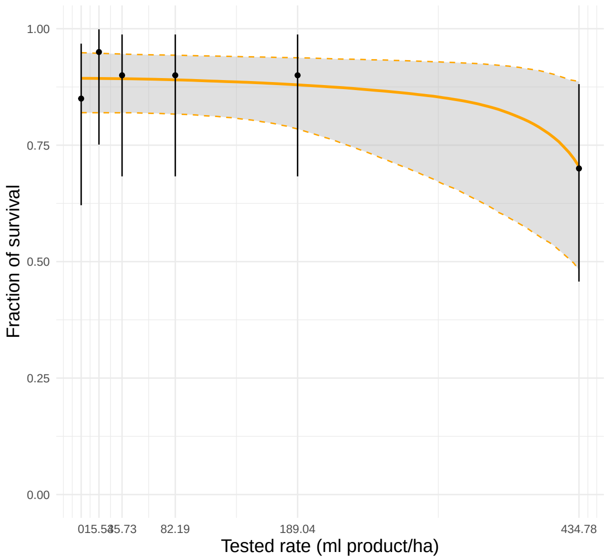

(a) Dose-response curve

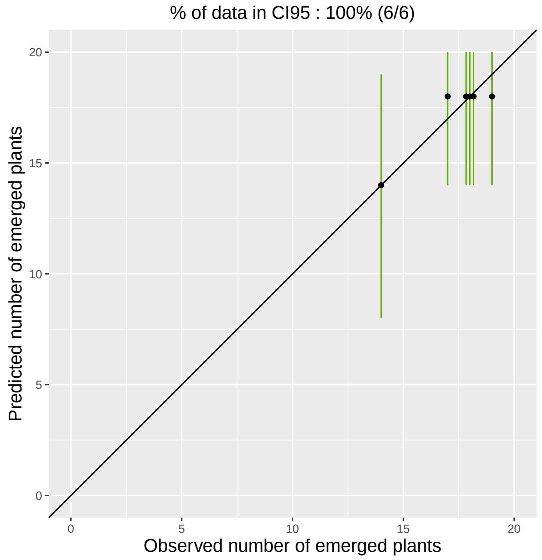

(b) Posterior predictive check (PPC)

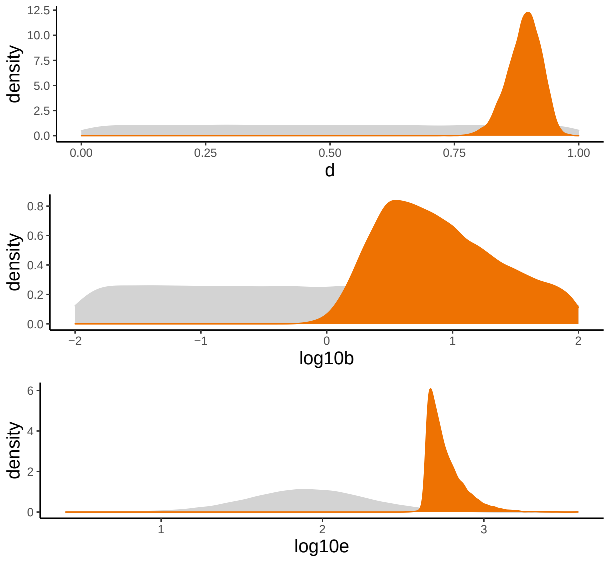

(c) Priors and posteriors

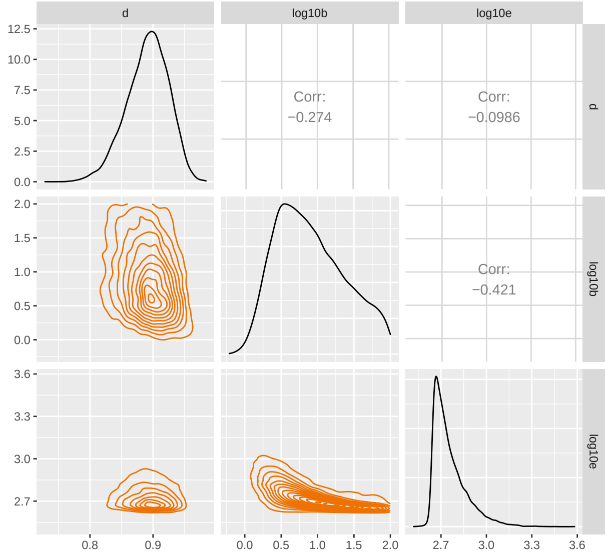

(d) Correlations between parameters

Figure 8: Dose-response curve (a), PPC (b), prior and posterior distributions (c) and correlations between parameters (d).

Data set: TRZAW_SE_emergence

Table 9: Summary of parameter estimates for TRZAW_SE_emergence data set

Parameter	median	Q2.5	Q97.5
b	30.012	3.919	95.029
d	0.947	0.898	0.977
e	1420.400	1061.659	3185.006

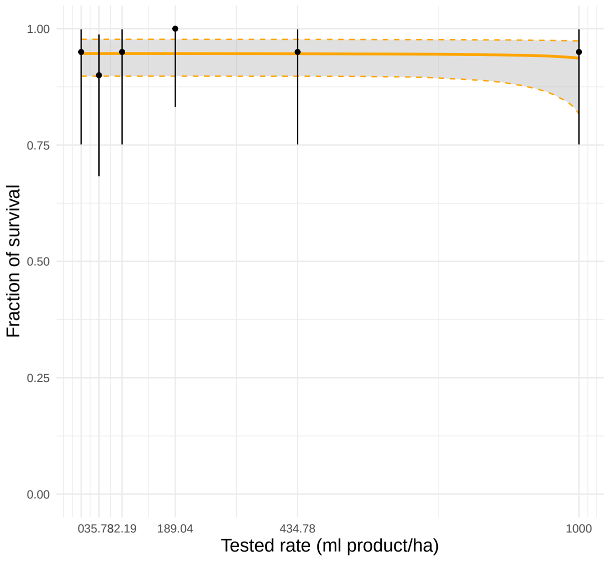

(a) Dose-response curve

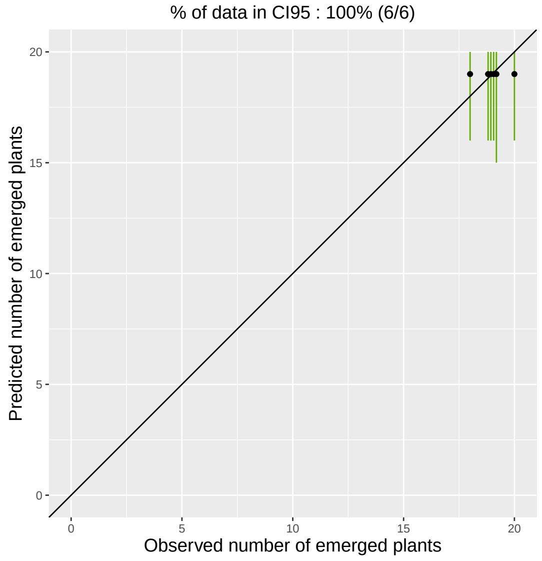

(b) Posterior predictive check (PPC)

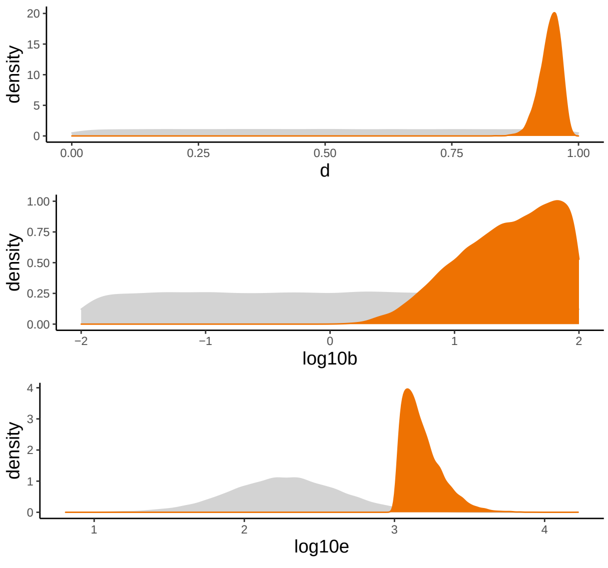

(c) Priors and posteriors

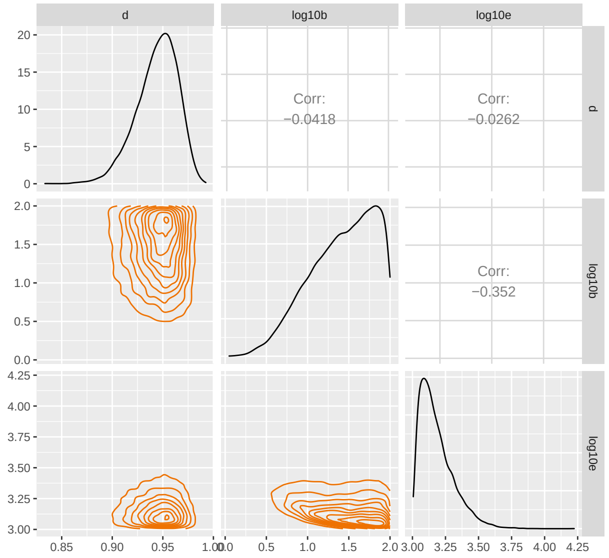

(d) Correlations between parameters

Figure 9: Dose-response curve (a), PPC (b), prior and posterior distributions (c) and correlations between parameters (d).

Data set: ZEAMA_SE_emergence

Table 10: Summary of parameter estimates (parameter d is set to 1) for ZEAMA_SE_emergence data set

Parameter	median	Q2.5	Q97.5
b	1.518	0.932	2.524
e	3051.785	1639.983	7638.387

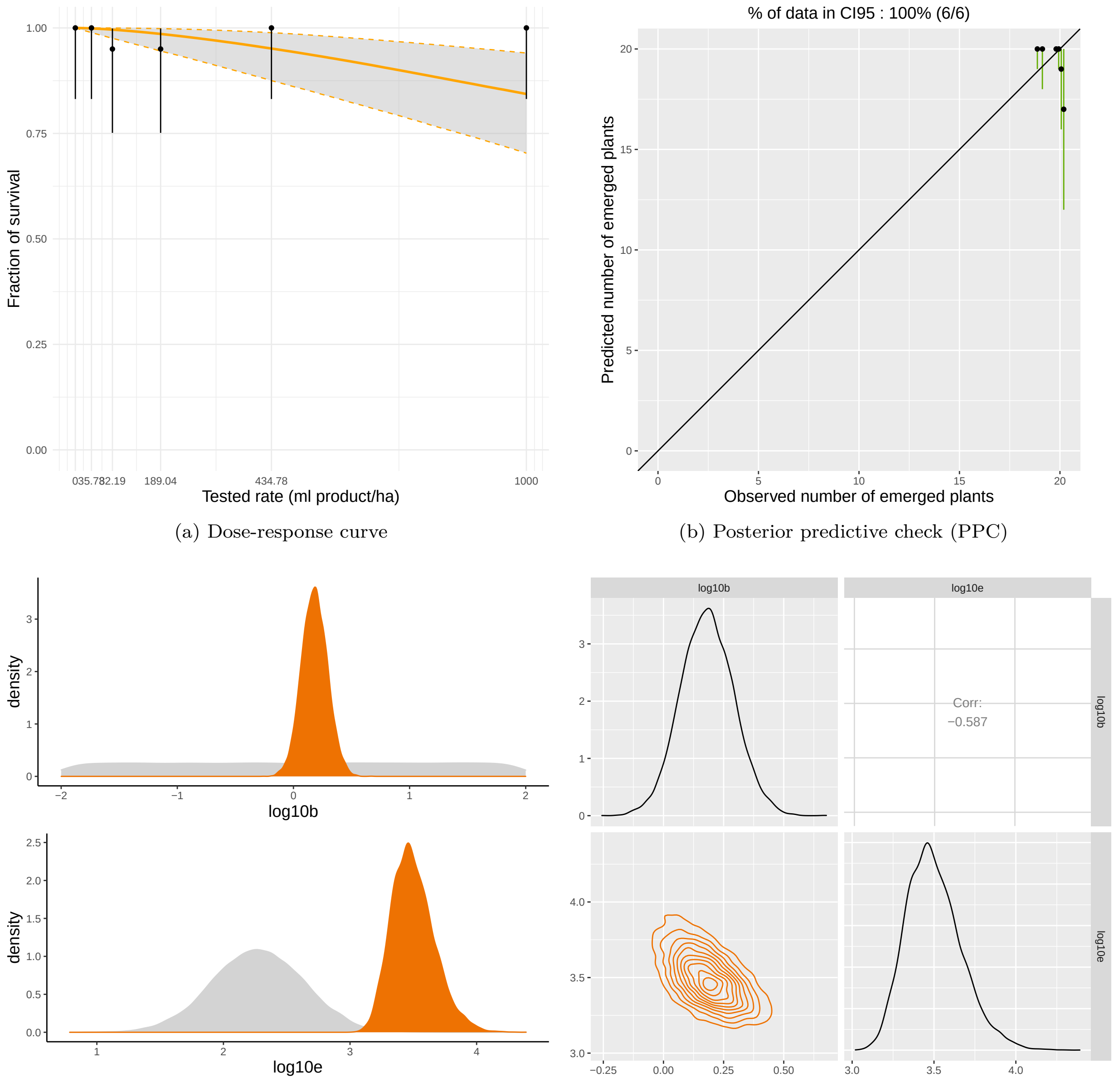

(a) Dose-response curve

(b) Posterior predictive check (PPC)

(c) Priors and posteriors

(d) Correlations between parameters

Figure 10: Dose-response curve (a), PPC (b), prior and posterior distributions (c) and correlations between parameters (d).
