# Supplementary material for: How to account for the uncertainty from standard toxicity tests in species sensitivity distributions: An example in non-target plants
Source: PLoS One. 2021 Jan 7;16(1):e0245071. doi: 10.1371/journal.pone.0245071 (PMC7790375; doi:10.1371/journal.pone.0245071)
Supplement: S1 Archive — It is a zip file containing seven folders (one folder per case study). Each folder contains five files report_xxx.pdf with detailed results of the dose-response analyses, one file corresponding to does-response analysis per endpoint. It also contains one file ER50_censoring.pdf for censored ER50 and one file SSD_analyses.pdf for results of SSD analyses. (ZIP) [file pone.0245071.s004.zip › S1_archive/Study4/report_SE_weight.pdf]

Dose-response analysis

Study 4

Seedling Emergence test - shoot dry SE_weight endpoint

25 June 2020

Contact: sandrine.charles@univ-lyon1.fr

This is a report which provides results on all performed dose-response analyses for the shoot dry SE_weight endpoint of the Seedling Emergence test for study 4.

Contents

Data set: ALLCE_SE_weight 2
Data set: AVESA_SE_weight 3
Data set: BEAVA_SE_weight 4
Data set: BRSNW_SE_weight 5
Data set: CUMSA_SE_weight 6
Data set: GLXMA_SE_weight 7
Data set: HELAN_SE_weight 8
Data set: LYPES_SE_weight 9
Data set: TRZAW_SE_weight 10
Data set: ZEAMA_SE_weight 11

Data set: ALLCE_SE_weight

Table 1: Summary of parameter estimates for ALLCE_SE_weight data set

Parameter	median	Q2.5	Q97.5
b	12.432	0.016	90.220
d	0.021	0.017	0.034
e	8.029	1.189	15.364
sigma	0.008	0.006	0.013

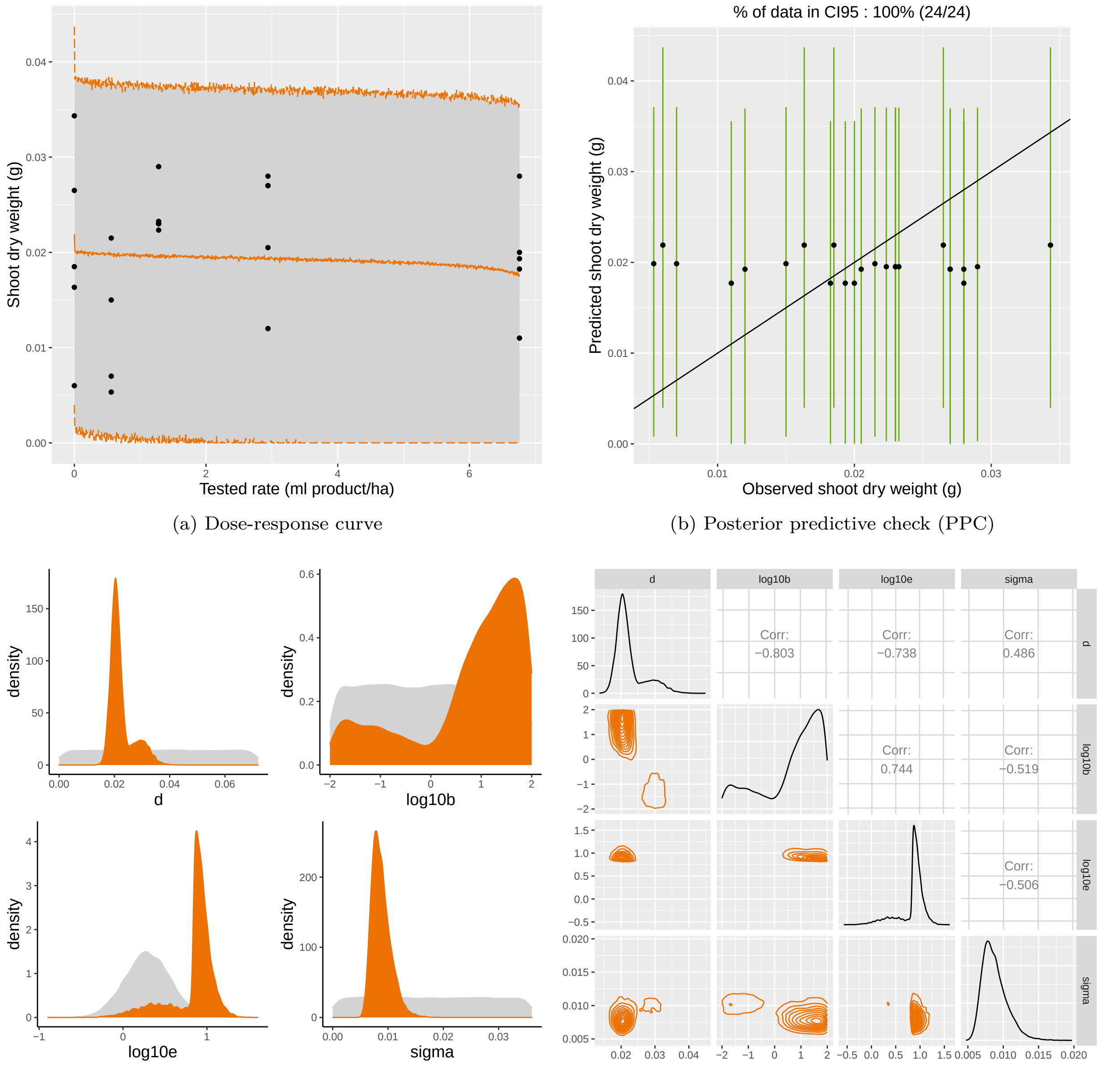

(a) Dose-response curve

(b) Posterior predictive check (PPC)

(c) Priors and posteriors

(d) Correlations between parameters

Figure 1: Dose-response curve (a), PPC (b), prior and posterior distributions (c) and correlations between parameters (d).

Data set: AVESA_SE_weight

Table 2: Summary of parameter estimates for AVESA_SE_weight data set

Parameter	median	Q2.5	Q97.5
b	1.710	1.377	2.164
d	0.752	0.698	0.807
e	110.671	93.192	130.310
sigma	0.059	0.045	0.080

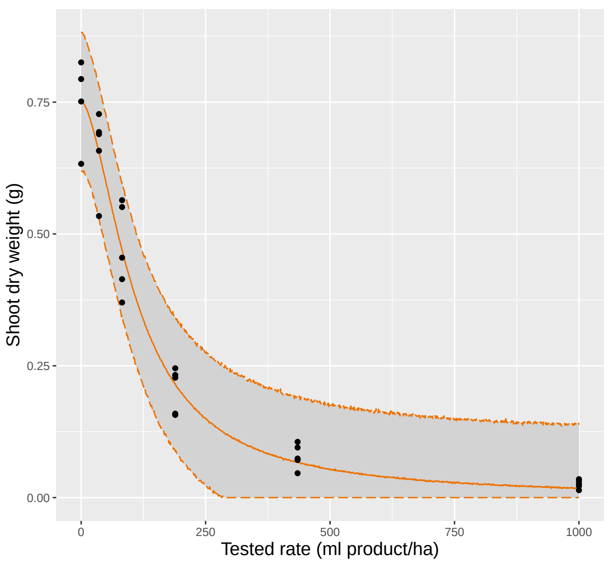

(a) Dose-response curve

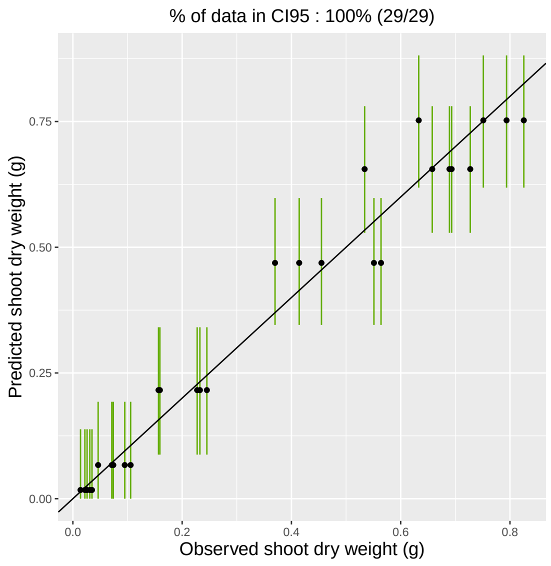

(b) Posterior predictive check (PPC)

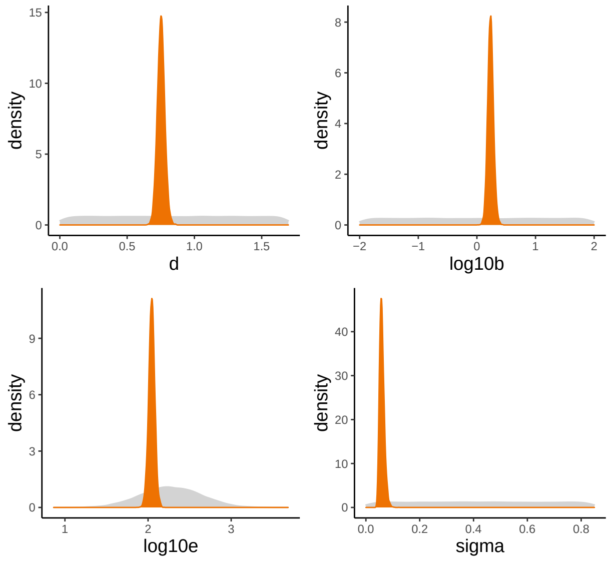

(c) Priors and posteriors

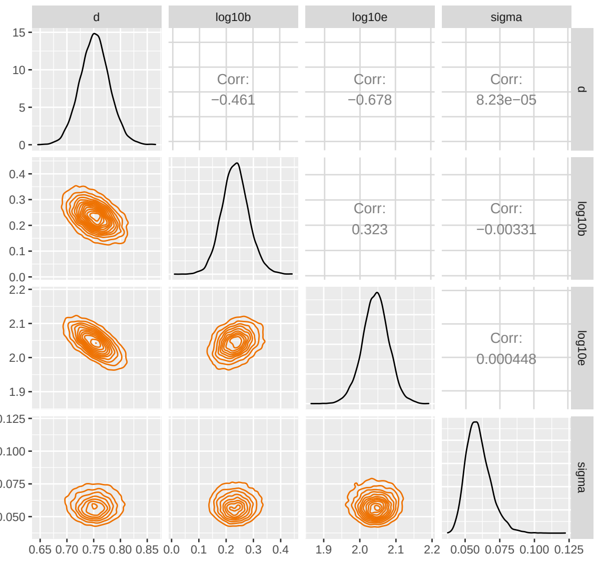

(d) Correlations between parameters

Figure 2: Dose-response curve (a), PPC (b), prior and posterior distributions (c) and correlations between parameters (d).

Data set: BEAVA_SE_weight

Table 3: Summary of parameter estimates for BEAVA_SE_weight data set

Parameter	median	Q2.5	Q97.5
b	1.305	0.911	1.955
d	1.735	1.590	1.897
e	54.289	41.534	70.856
sigma	0.257	0.212	0.321

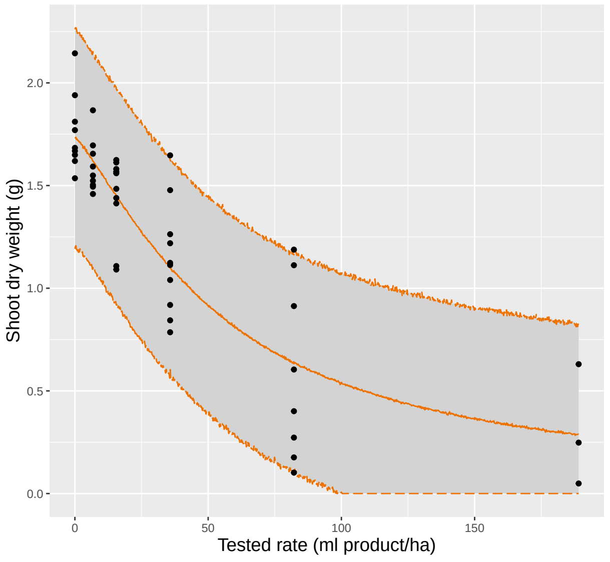

(a) Dose-response curve

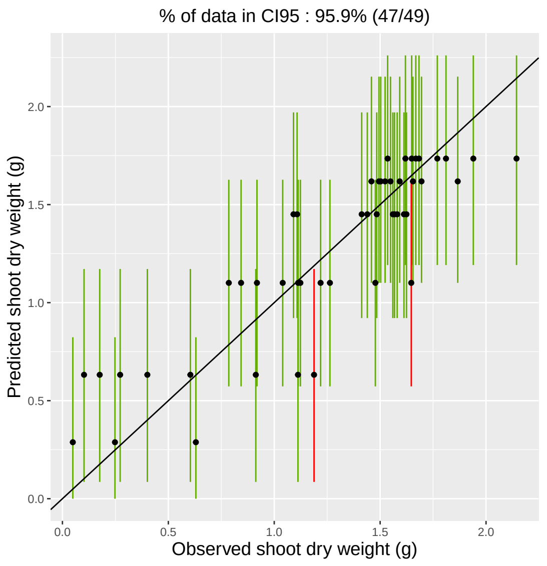

(b) Posterior predictive check (PPC)

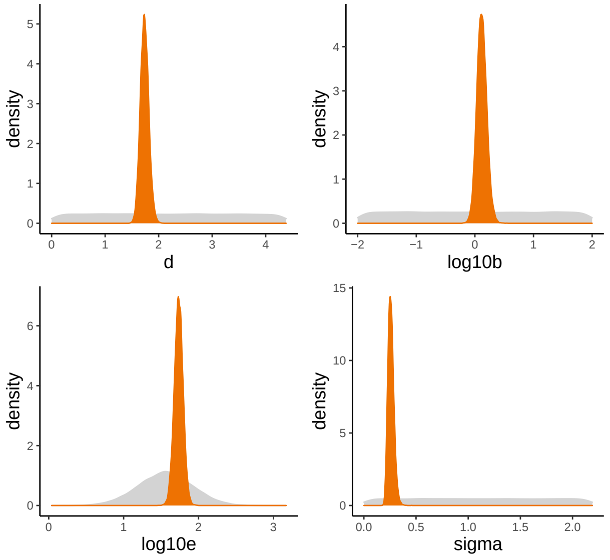

(c) Priors and posteriors

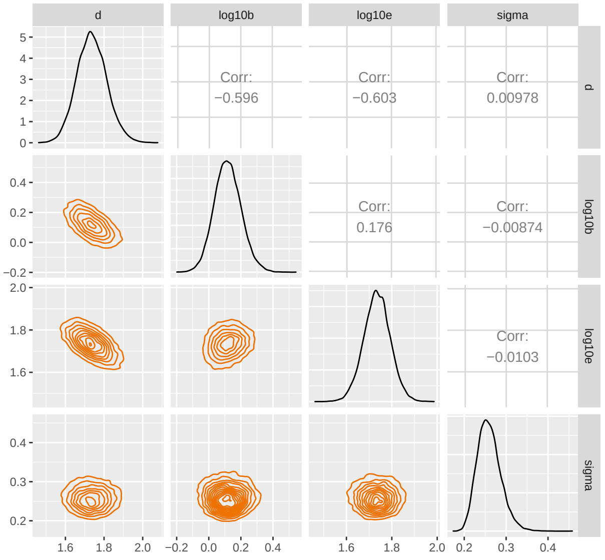

(d) Correlations between parameters

Figure 3: Dose-response curve (a), PPC (b), prior and posterior distributions (c) and correlations between parameters (d).

Data set: BRSNW_SE_weight

Table 4: Summary of parameter estimates for BRSNW_SE_weight data set

Parameter	median	Q2.5	Q97.5
b	2.081	1.277	3.965
d	3.167	2.857	3.504
e	44.903	34.536	61.399
sigma	0.711	0.583	0.885

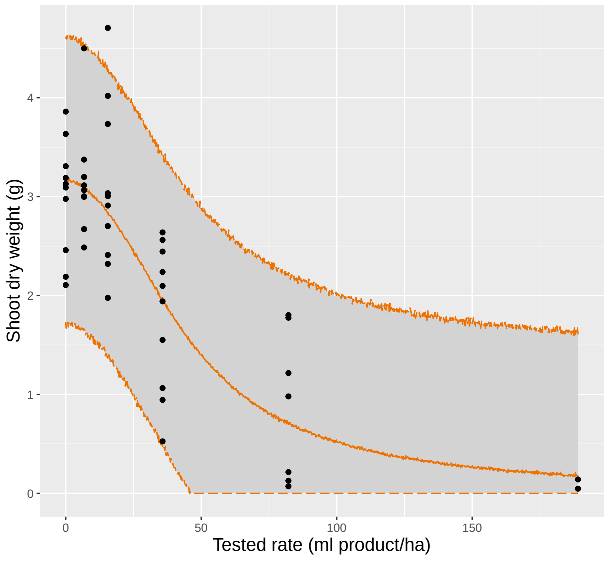

(a) Dose-response curve

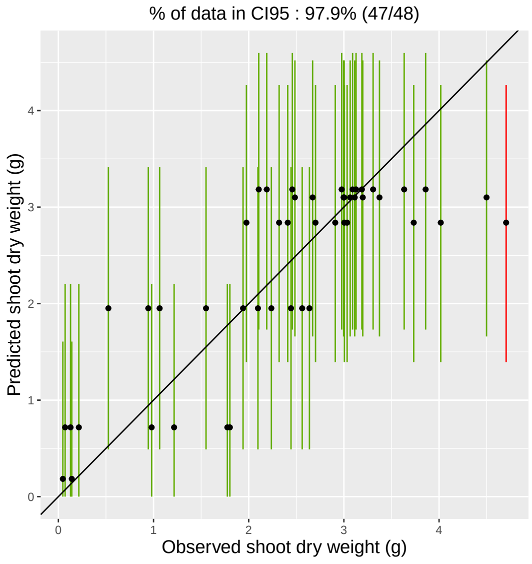

(b) Posterior predictive check (PPC)

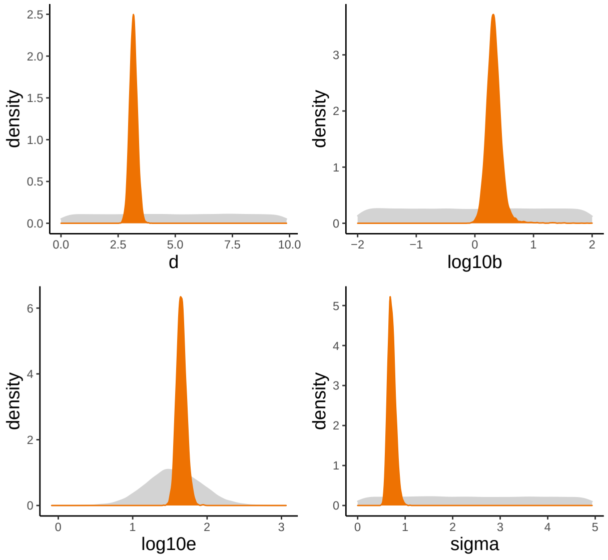

(c) Priors and posteriors

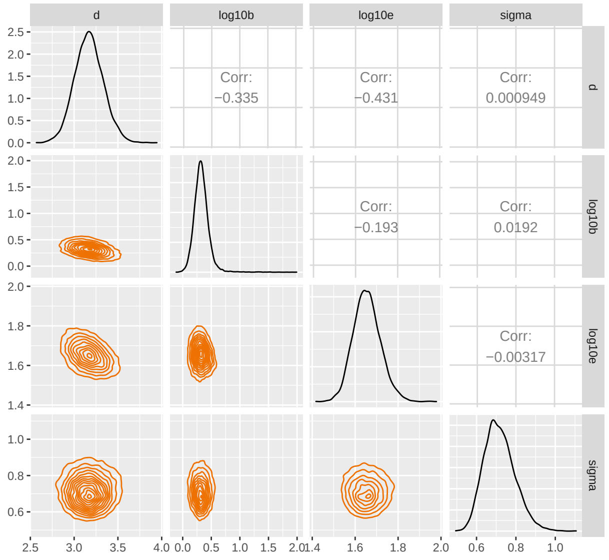

(d) Correlations between parameters

Figure 4: Dose-response curve (a), PPC (b), prior and posterior distributions (c) and correlations between parameters (d).

Data set: CUMSA_SE_weight

Table 5: Summary of parameter estimates for CUMSA_SE_weight data set

Parameter	median	Q2.5	Q97.5
b	2.231	1.562	3.395
d	4.413	4.062	4.796
e	98.977	80.277	120.276
sigma	0.751	0.631	0.915

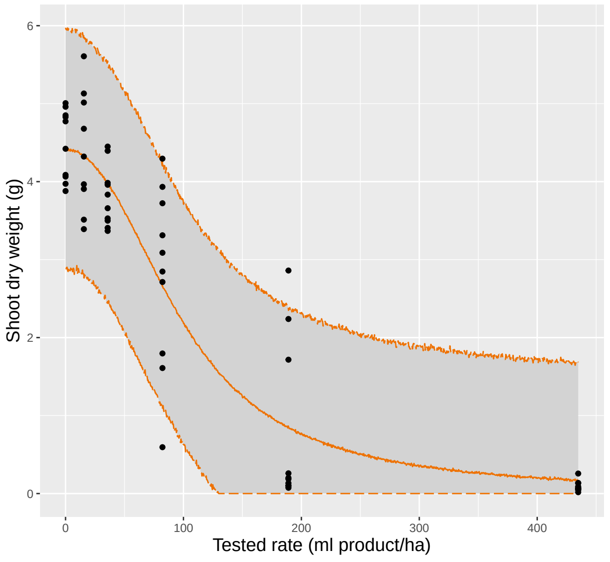

(a) Dose-response curve

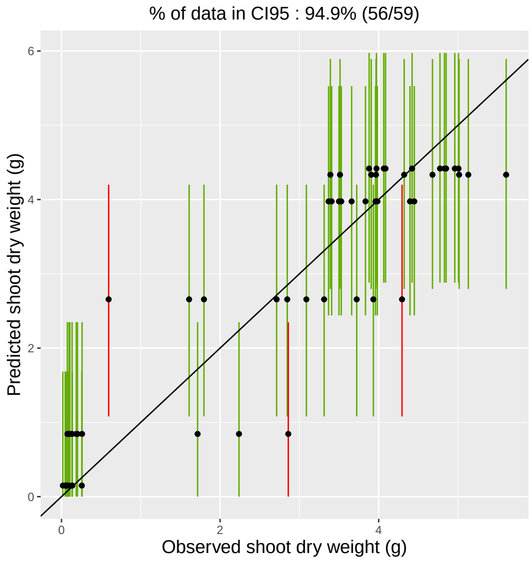

(b) Posterior predictive check (PPC)

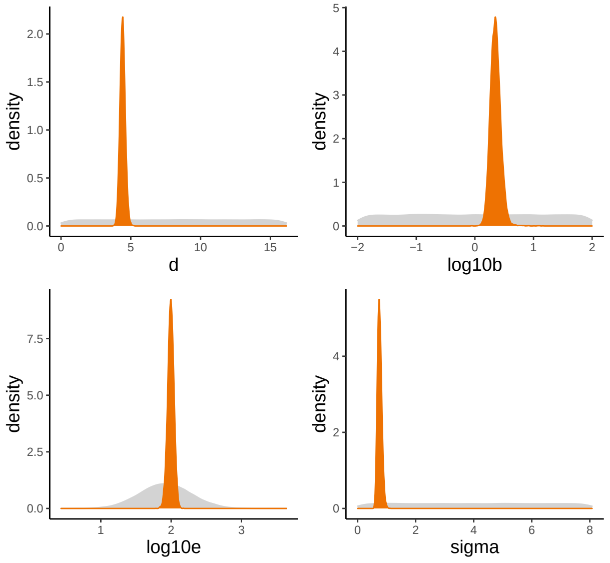

(c) Priors and posteriors

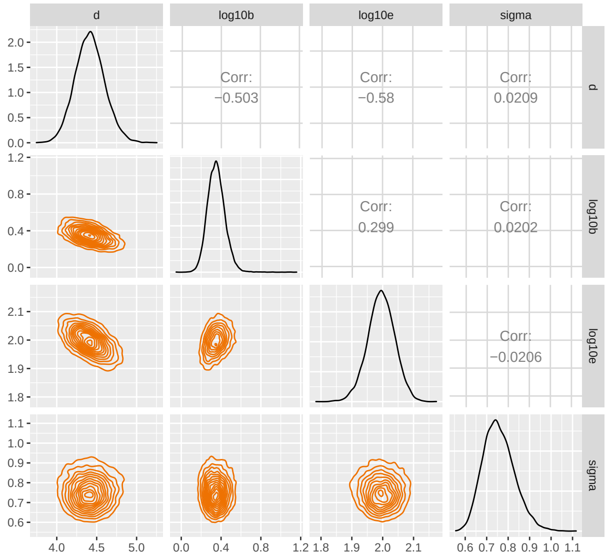

(d) Correlations between parameters

Figure 5: Dose-response curve (a), PPC (b), prior and posterior distributions (c) and correlations between parameters (d).

Data set: GLXMA_SE_weight

Table 6: Summary of parameter estimates for GLXMA_SE_weight data set

Parameter	median	Q2.5	Q97.5
b	1.263	0.900	1.798
d	1.696	1.572	1.840
e	186.609	143.242	237.514
sigma	0.247	0.206	0.303

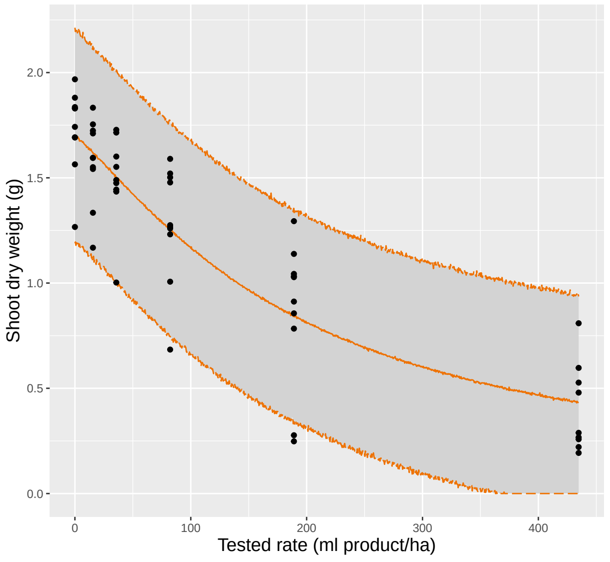

(a) Dose-response curve

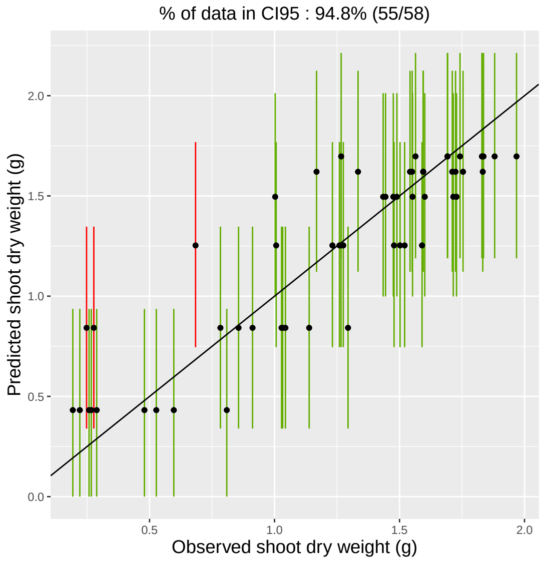

(b) Posterior predictive check (PPC)

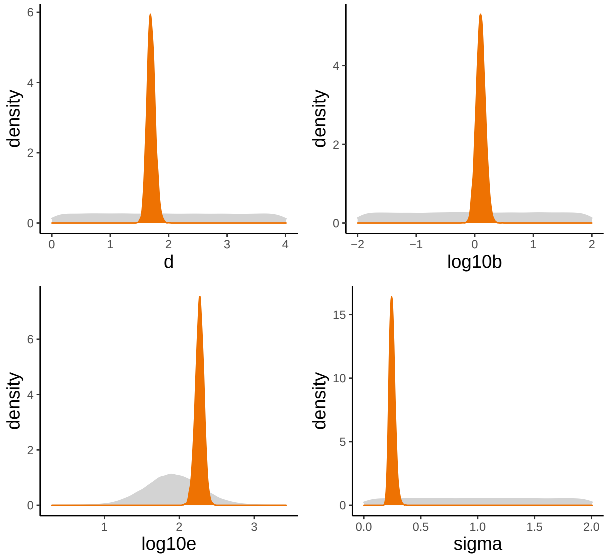

(c) Priors and posteriors

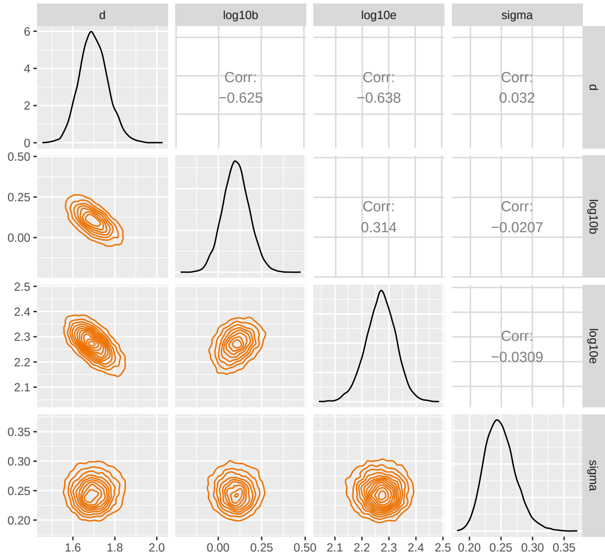

(d) Correlations between parameters

Figure 6: Dose-response curve (a), PPC (b), prior and posterior distributions (c) and correlations between parameters (d).

Data set: HELAN_SE_weight

Table 7: Summary of parameter estimates for HELAN_SE_weight data set

Parameter	median	Q2.5	Q97.5
b	1.901	1.377	2.807
d	1.043	0.958	1.135
e	69.388	56.477	84.429
sigma	0.154	0.127	0.194

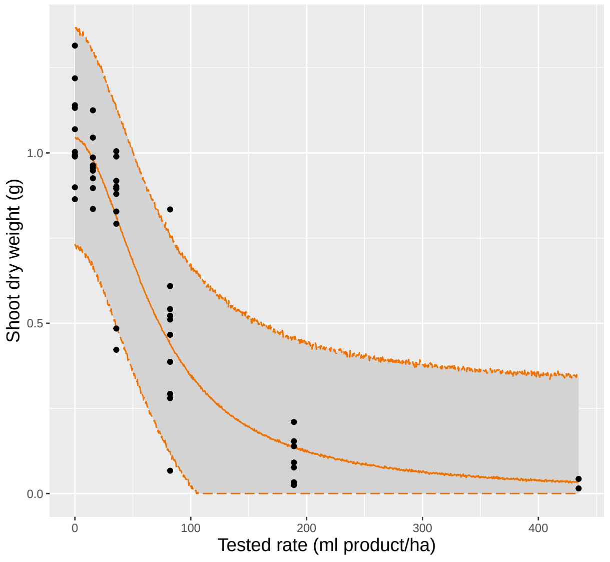

(a) Dose-response curve

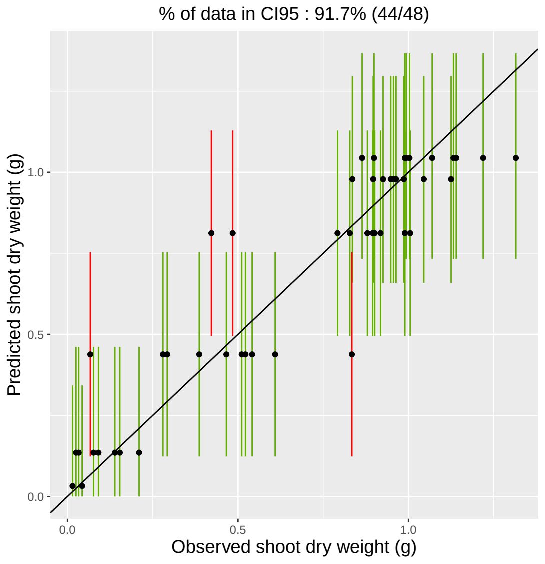

(b) Posterior predictive check (PPC)

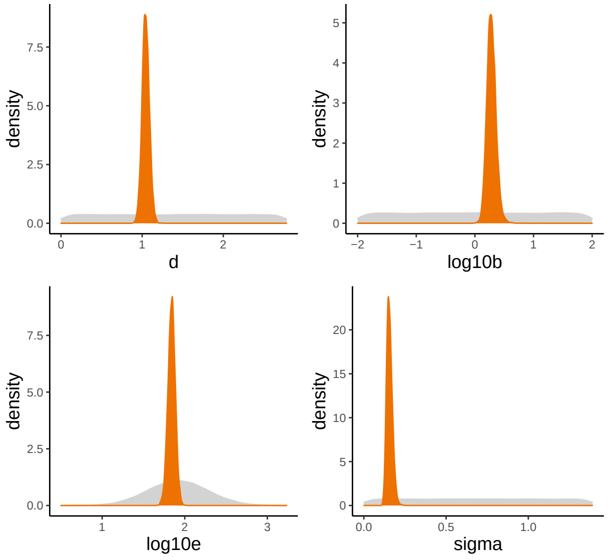

(c) Priors and posteriors

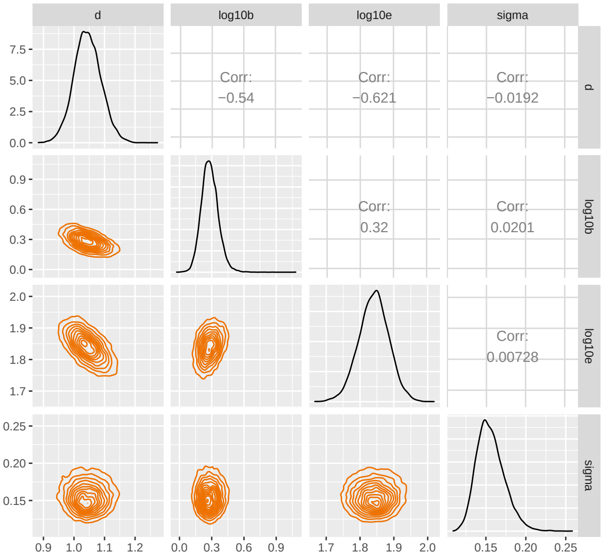

(d) Correlations between parameters

Figure 7: Dose-response curve (a), PPC (b), prior and posterior distributions (c) and correlations between parameters (d).

Data set: LYPES_SE_weight

Table 8: Summary of parameter estimates for LYPES_SE_weight data set

Parameter	median	Q2.5	Q97.5
b	3.121	1.856	18.591
d	1.205	1.076	1.348
e	42.107	35.353	52.337
sigma	0.240	0.199	0.298

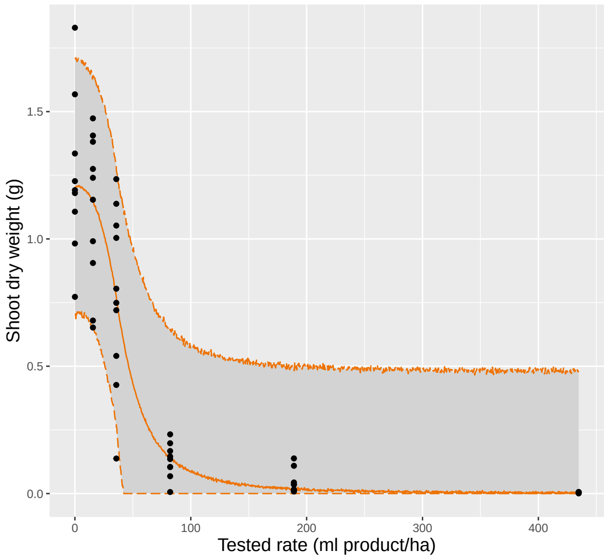

(a) Dose-response curve

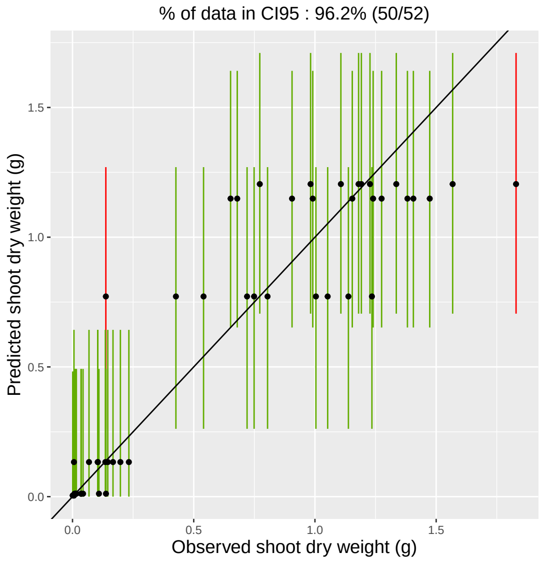

(b) Posterior predictive check (PPC)

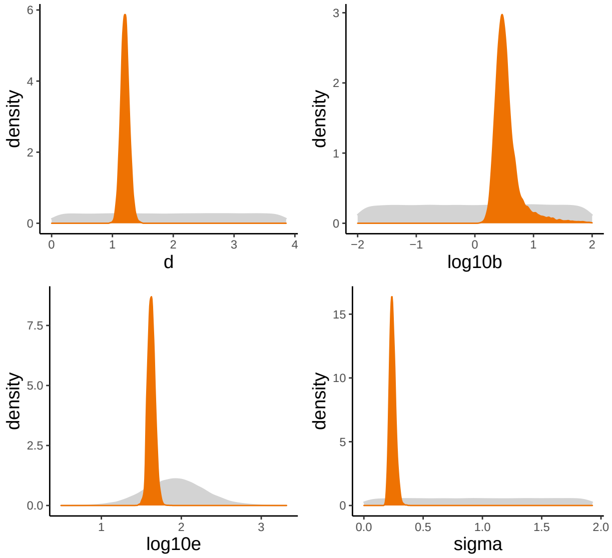

(c) Priors and posteriors

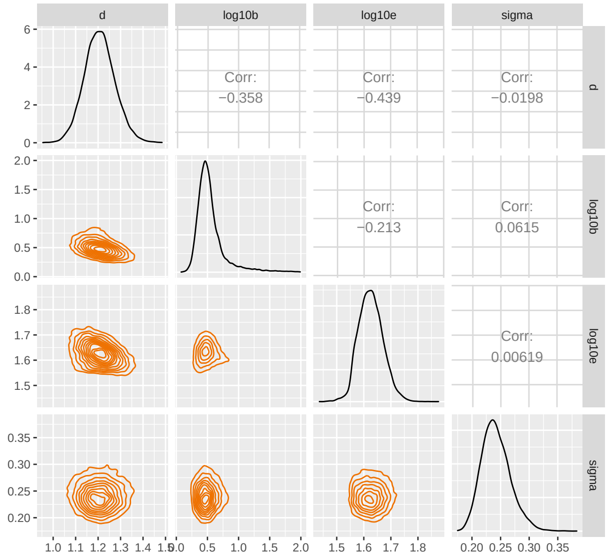

(d) Correlations between parameters

Figure 8: Dose-response curve (a), PPC (b), prior and posterior distributions (c) and correlations between parameters (d).

Data set: TRZAW_SE_weight

Table 9: Summary of parameter estimates for TRZAW_SE_weight data set

Parameter	median	Q2.5	Q97.5
b	0.846	0.643	1.114
d	0.610	0.575	0.647
e	880.597	672.858	1195.803
sigma	0.045	0.034	0.061

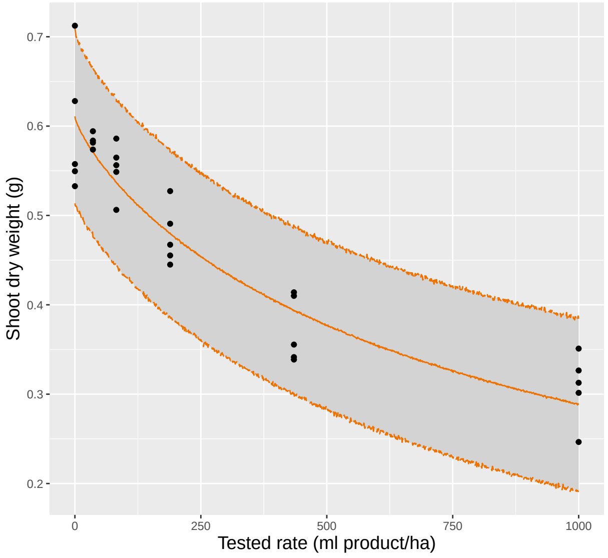

(a) Dose-response curve

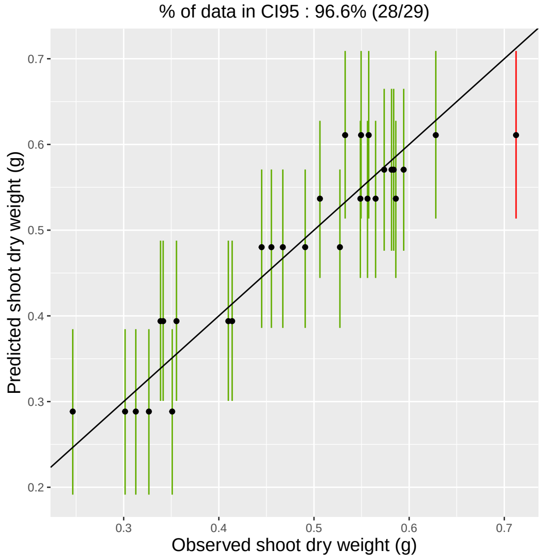

(b) Posterior predictive check (PPC)

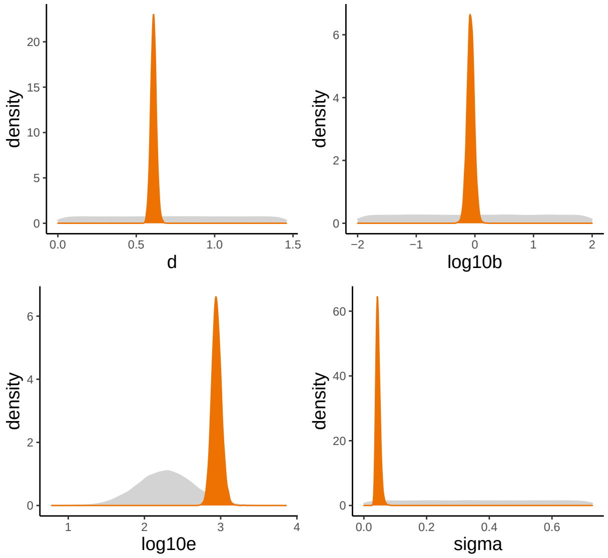

(c) Priors and posteriors

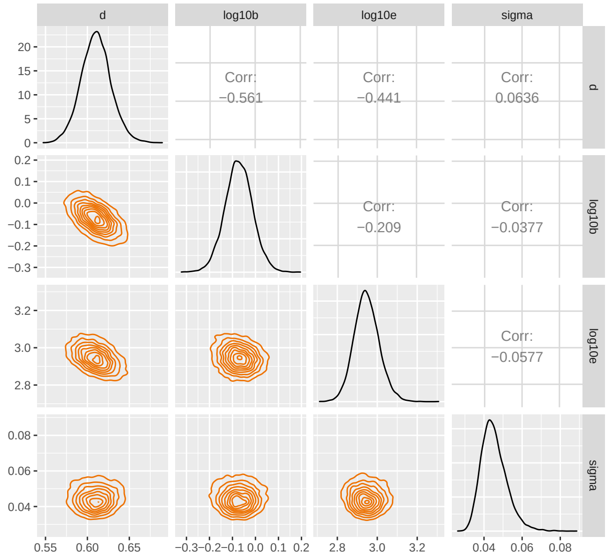

(d) Correlations between parameters

Figure 9: Dose-response curve (a), PPC (b), prior and posterior distributions (c) and correlations between parameters (d).

Data set: ZEAMA_SE_weight

Table 10: Summary of parameter estimates for ZEAMA_SE_weight data set

Parameter	median	Q2.5	Q97.5
b	1.103	0.792	1.557
d	5.416	4.937	5.954
e	302.849	219.225	417.390
sigma	0.906	0.757	1.115

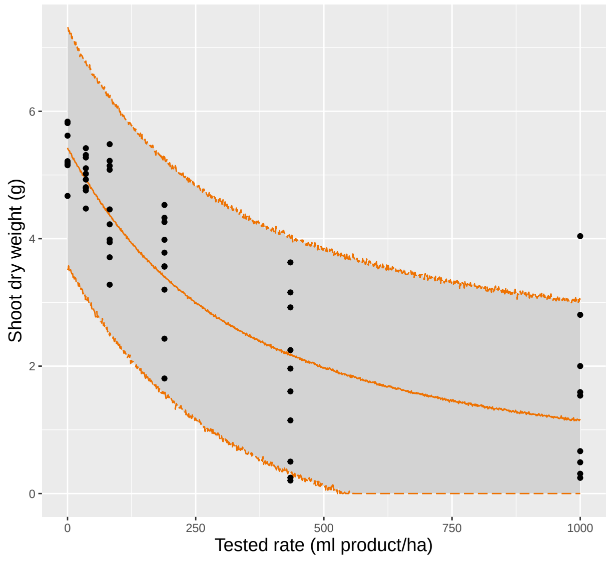

(a) Dose-response curve

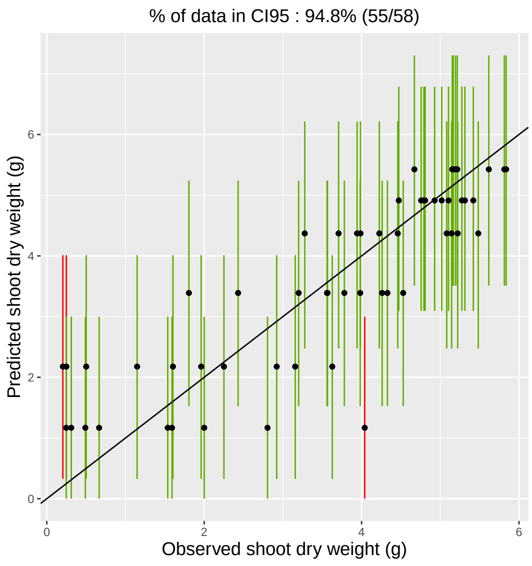

(b) Posterior predictive check (PPC)

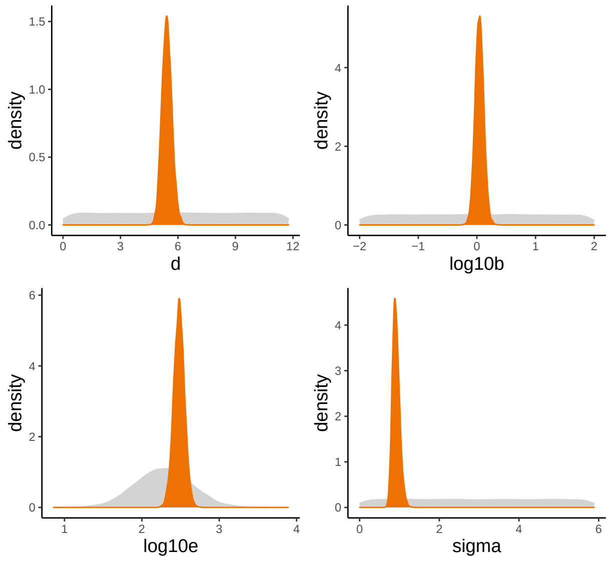

(c) Priors and posteriors

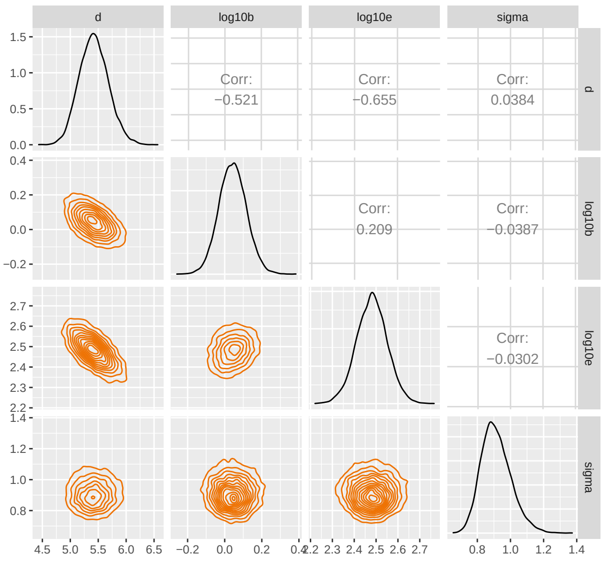

(d) Correlations between parameters

Figure 10: Dose-response curve (a), PPC (b), prior and posterior distributions (c) and correlations between parameters (d).
