# Supplementary material for: How to account for the uncertainty from standard toxicity tests in species sensitivity distributions: An example in non-target plants
Source: PLoS One. 2021 Jan 7;16(1):e0245071. doi: 10.1371/journal.pone.0245071 (PMC7790375; doi:10.1371/journal.pone.0245071)
Supplement: S1 Archive — It is a zip file containing seven folders (one folder per case study). Each folder contains five files report_xxx.pdf with detailed results of the dose-response analyses, one file corresponding to does-response analysis per endpoint. It also contains one file ER50_censoring.pdf for censored ER50 and one file SSD_analyses.pdf for results of SSD analyses. (ZIP) [file pone.0245071.s004.zip › S1_archive/Study4/report_VV_weight.pdf]

Dose-response analysis

Study 4

Vegetative Vigour test - shoot dry VV_weight endpoint

25 June 2020

Contact: sandrine.charles@univ-lyon1.fr

This is a report which provides results on all performed dose-response analyses for the shoot dry VV_weight endpoint of the Vegetative Vigour test for study 4.

Contents

Data set: ALLCE_VV_weight 2
Data set: AVESA_VV_weight 3
Data set: BEAVA_VV_weight 4
Data set: BRSNW_VV_weight 5
Data set: CUMSA_VV_weight 6
Data set: GLXMA_VV_weight 7
Data set: HELAN_VV_weight 8
Data set: LYPES_VV_weight 9
Data set: TRZAW_VV_weight 10
Data set: ZEAMA_VV_weight 11

Data set: ALLCE_VV_weight

Table 1: Summary of parameter estimates for ALLCE_VV_weight data set

Parameter	median	Q2.5	Q97.5
b	0.752	0.153	3.744
d	0.209	0.159	0.262
e	150.609	52.123	370.245
sigma	0.056	0.043	0.076

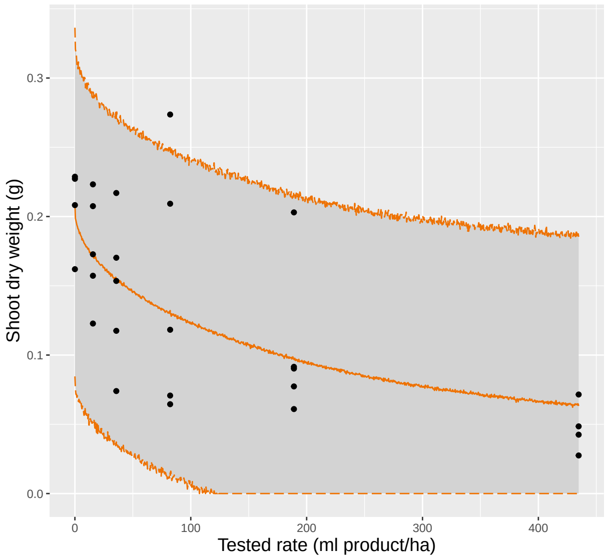

(a) Dose-response curve

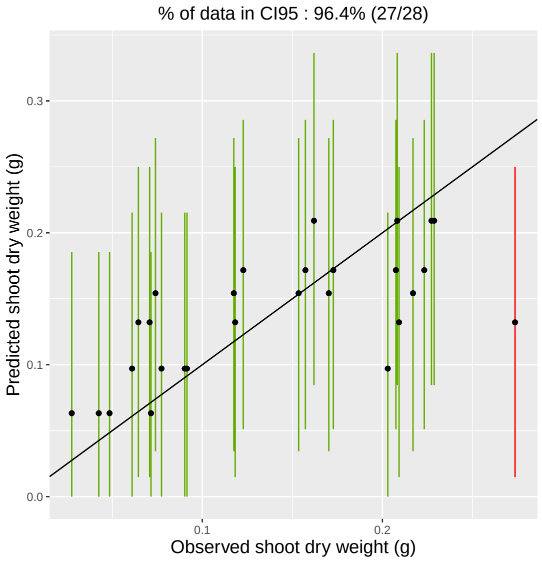

(b) Posterior predictive check (PPC)

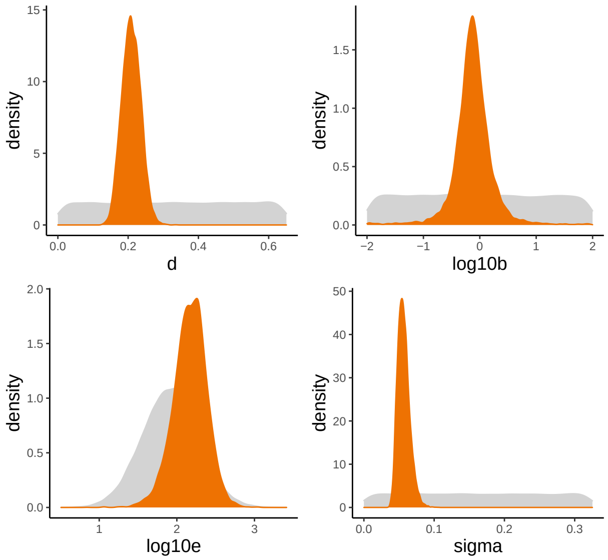

(c) Priors and posteriors

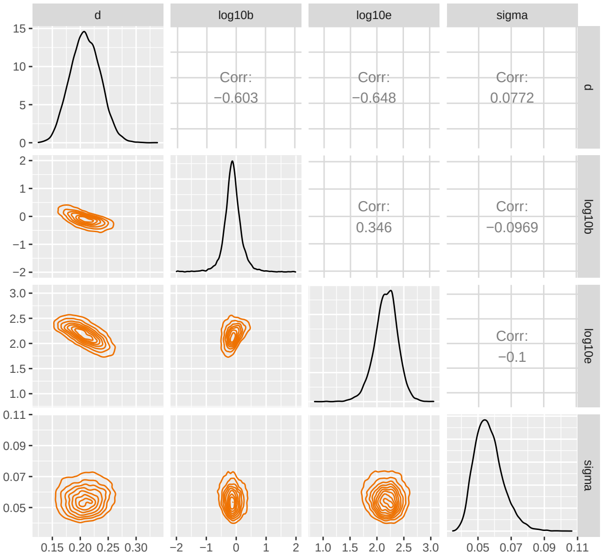

(d) Correlations between parameters

Figure 1: Dose-response curve (a), PPC (b), prior and posterior distributions (c) and correlations between parameters (d).

Data set: AVESA_VV_weight

Table 2: Summary of parameter estimates for AVESA_VV_weight data set

Parameter	median	Q2.5	Q97.5
b	2.507	1.923	3.346
d	2.335	2.178	2.502
e	115.250	101.208	131.442
sigma	0.193	0.145	0.275

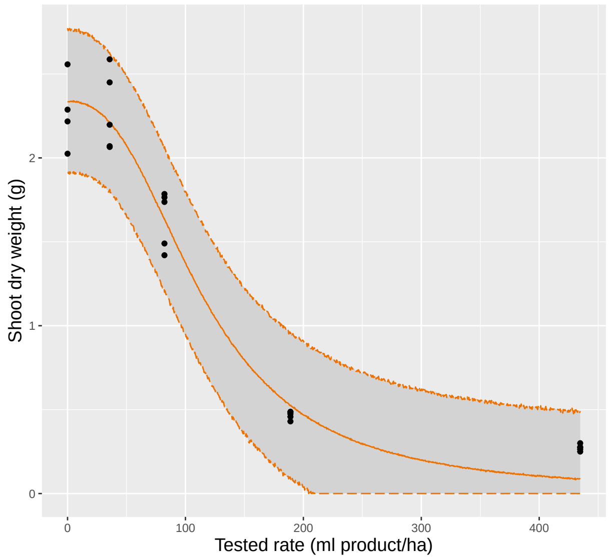

(a) Dose-response curve

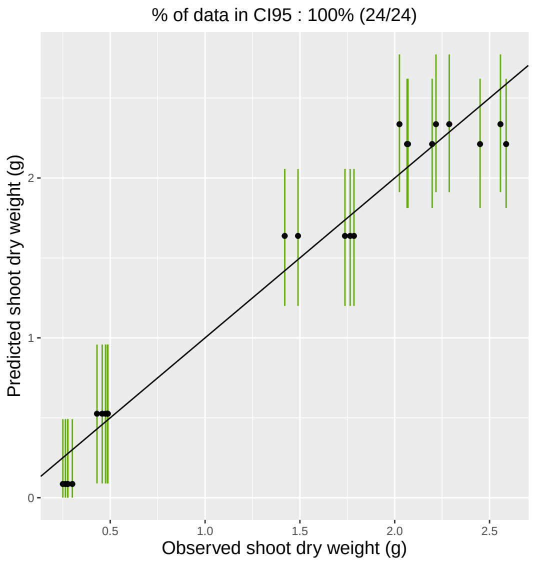

(b) Posterior predictive check (PPC)

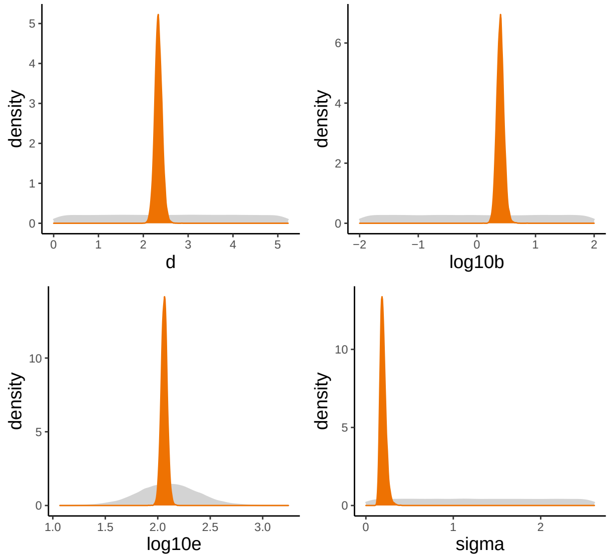

(c) Priors and posteriors

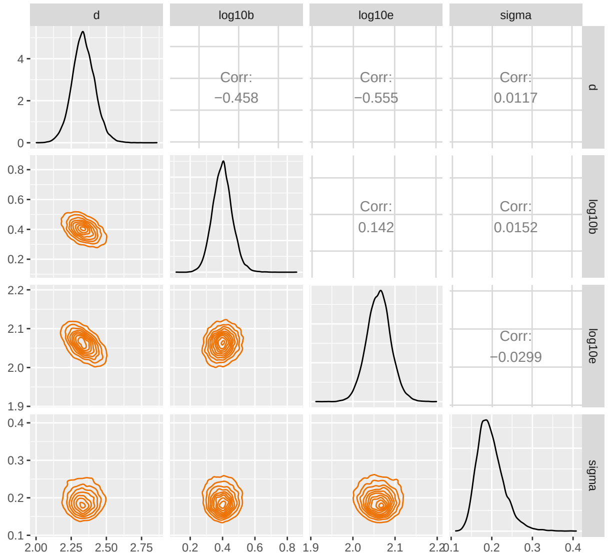

(d) Correlations between parameters

Figure 2: Dose-response curve (a), PPC (b), prior and posterior distributions (c) and correlations between parameters (d).

Data set: BEAVA_VV_weight

Table 3: Summary of parameter estimates for BEAVA_VV_weight data set

Parameter	median	Q2.5	Q97.5
b	1.198	0.888	1.638
d	4.245	3.949	4.536
e	8.574	6.797	10.427
sigma	0.447	0.371	0.554

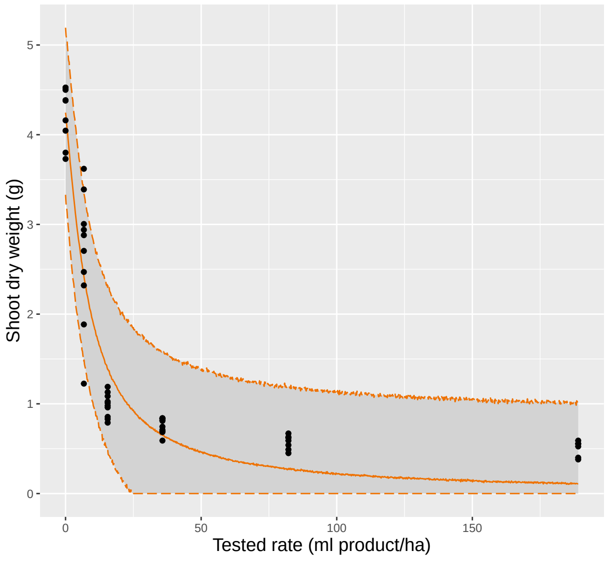

(a) Dose-response curve

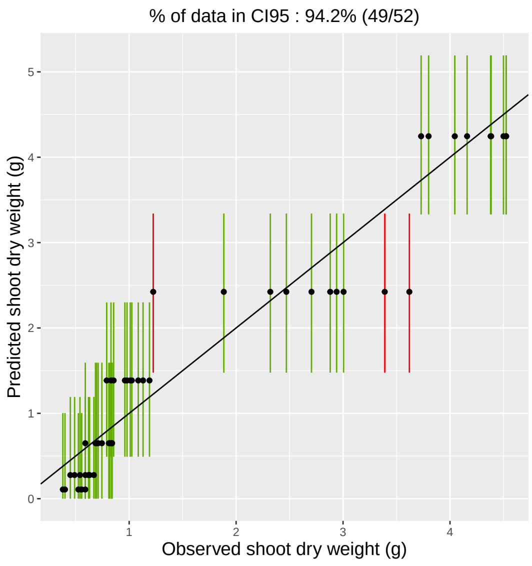

(b) Posterior predictive check (PPC)

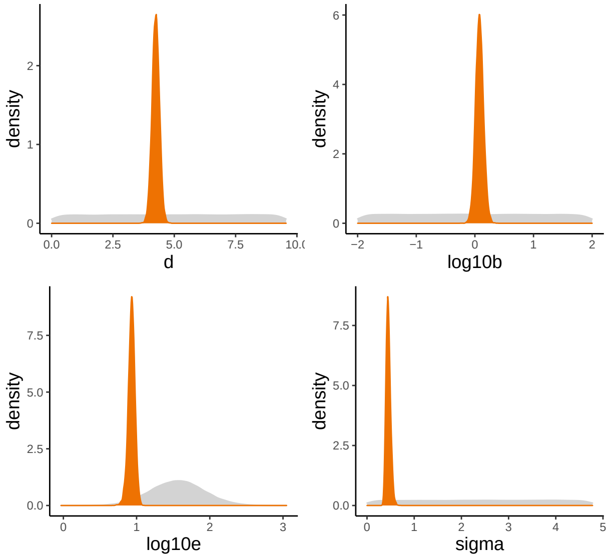

(c) Priors and posteriors

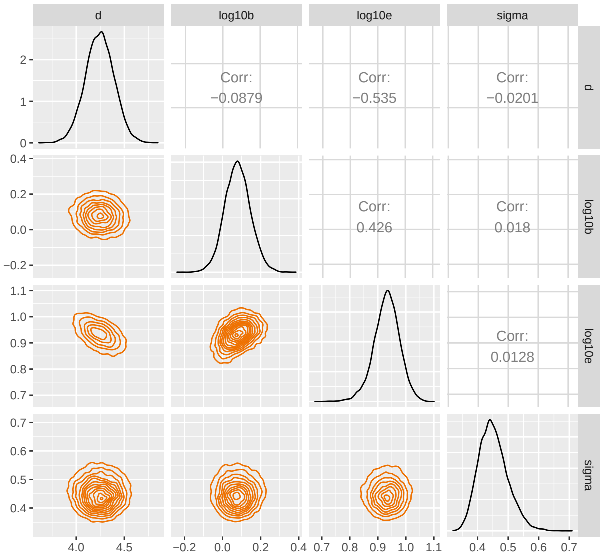

(d) Correlations between parameters

Figure 3: Dose-response curve (a), PPC (b), prior and posterior distributions (c) and correlations between parameters (d).

Data set: BRSNW_VV_weight

Table 4: Summary of parameter estimates for BRSNW_VV_weight data set

Parameter	median	Q2.5	Q97.5
b	1.997	1.606	2.528
d	9.193	8.677	9.732
e	19.299	17.089	21.950
sigma	0.927	0.774	1.135

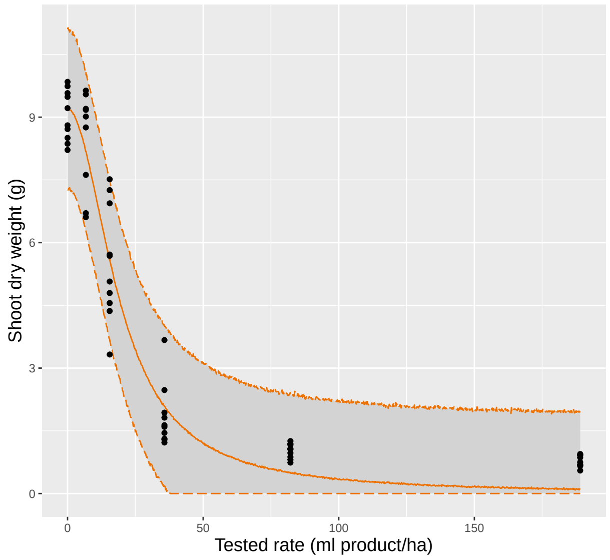

(a) Dose-response curve

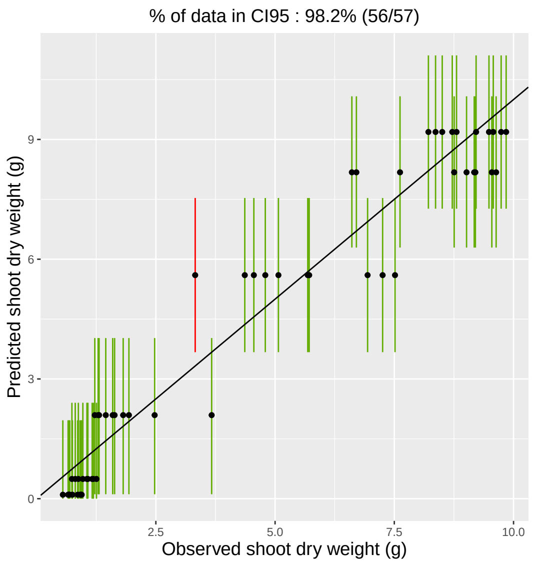

(b) Posterior predictive check (PPC)

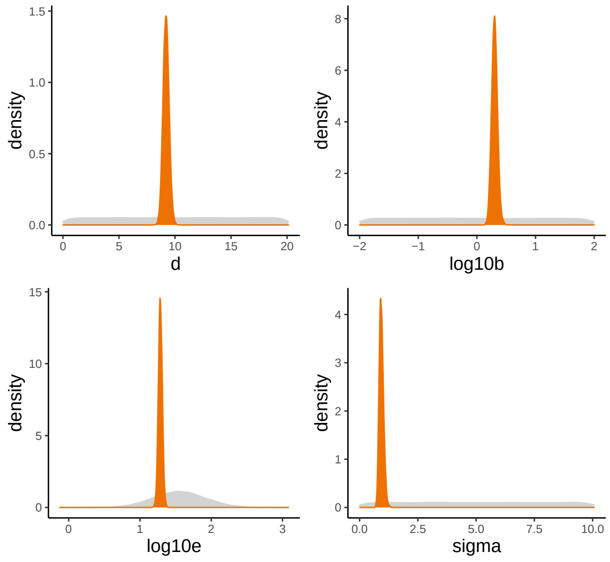

(c) Priors and posteriors

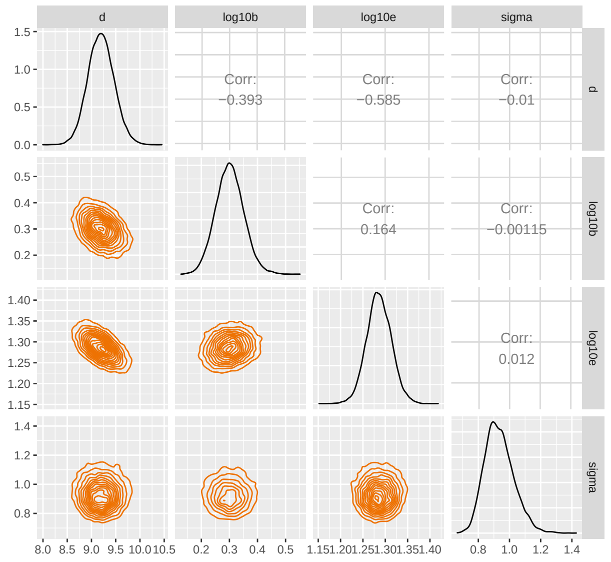

(d) Correlations between parameters

Figure 4: Dose-response curve (a), PPC (b), prior and posterior distributions (c) and correlations between parameters (d).

Data set: CUMSA_VV_weight

Table 5: Summary of parameter estimates for CUMSA_VV_weight data set

Parameter	median	Q2.5	Q97.5
b	1.032	0.848	1.274
d	16.883	16.052	17.747
e	16.931	13.965	20.659
sigma	2.177	1.912	2.512

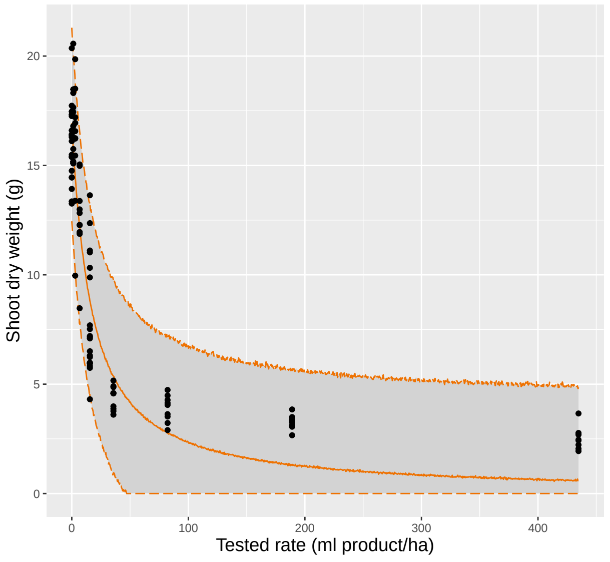

(a) Dose-response curve

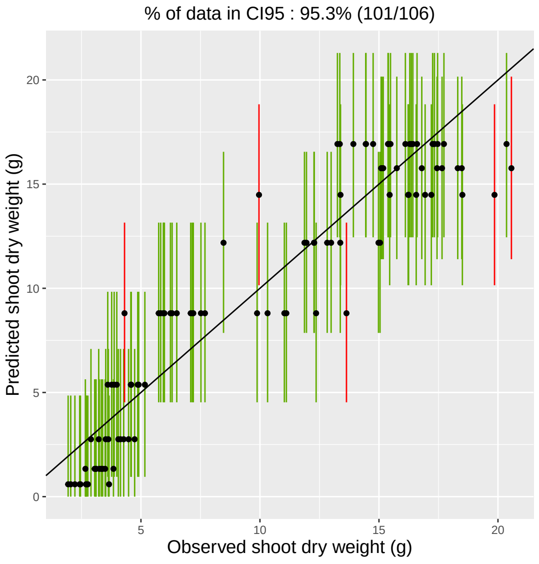

(b) Posterior predictive check (PPC)

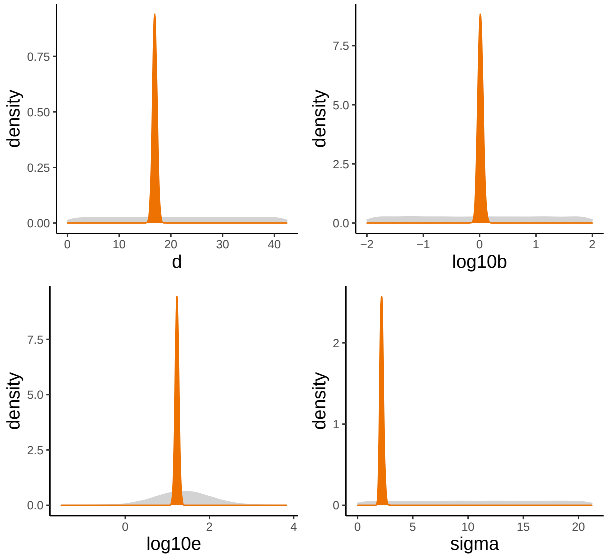

(c) Priors and posteriors

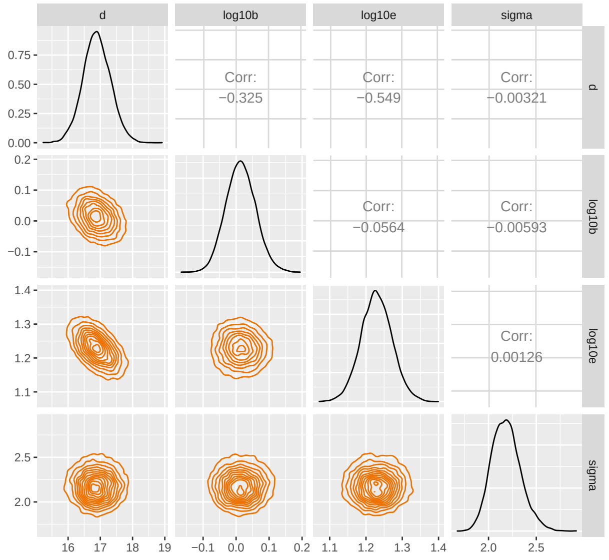

(d) Correlations between parameters

Figure 5: Dose-response curve (a), PPC (b), prior and posterior distributions (c) and correlations between parameters (d).

Data set: GLXMA_VV_weight

Table 6: Summary of parameter estimates for GLXMA_VV_weight data set

Parameter	median	Q2.5	Q97.5
b	1.545	1.279	1.889
d	6.491	6.122	6.851
e	33.033	29.031	37.891
sigma	0.579	0.482	0.716

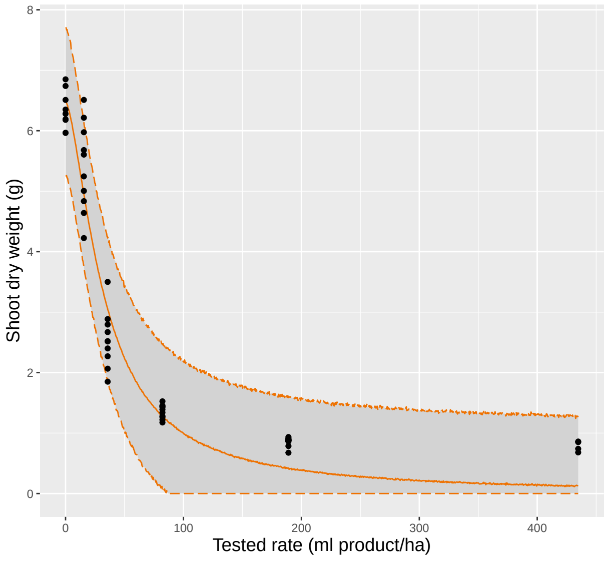

(a) Dose-response curve

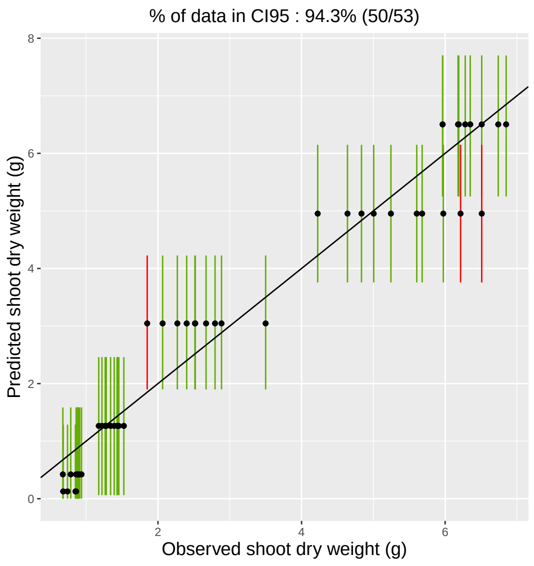

(b) Posterior predictive check (PPC)

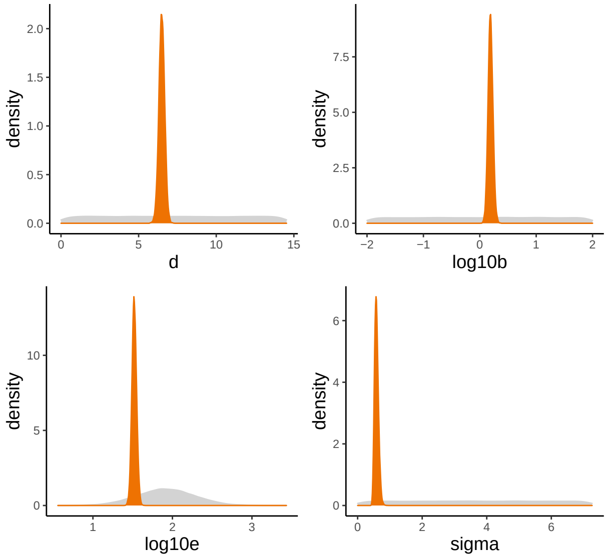

(c) Priors and posteriors

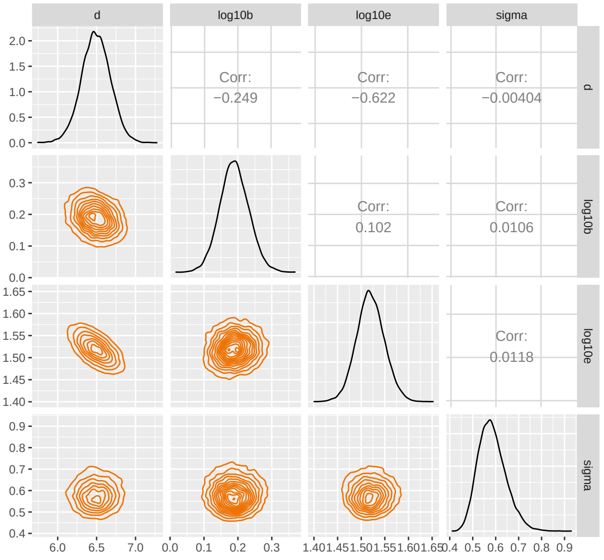

(d) Correlations between parameters

Figure 6: Dose-response curve (a), PPC (b), prior and posterior distributions (c) and correlations between parameters (d).

Data set: HELAN_VV_weight

Table 7: Summary of parameter estimates for HELAN_VV_weight data set

Parameter	median	Q2.5	Q97.5
b	2.308	1.662	3.130
d	3.900	3.591	4.215
e	23.907	20.714	27.804
sigma	0.461	0.360	0.626

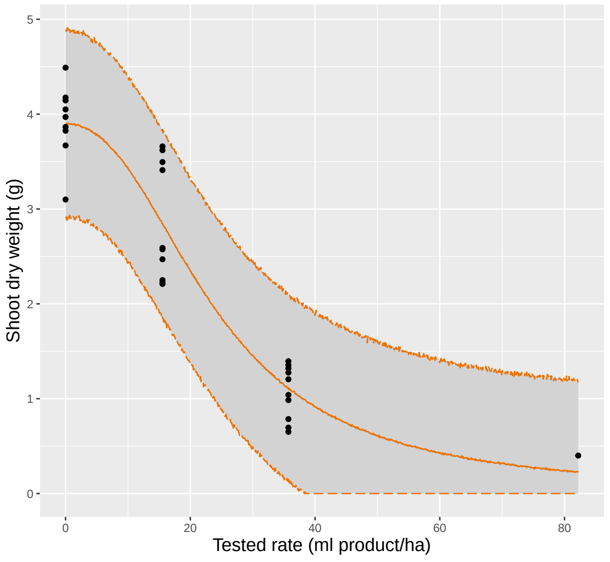

(a) Dose-response curve

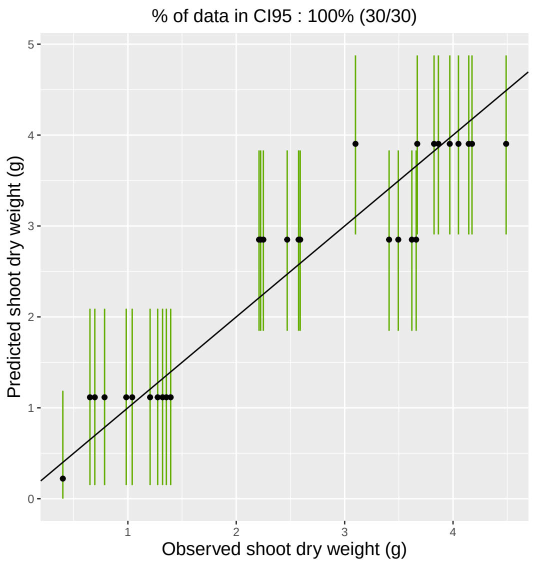

(b) Posterior predictive check (PPC)

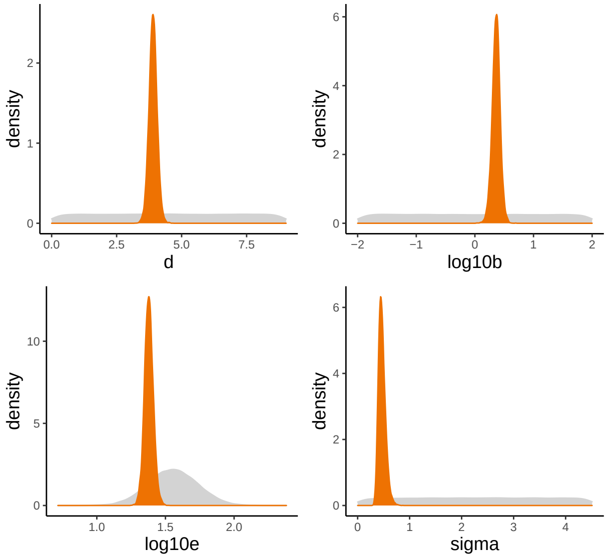

(c) Priors and posteriors

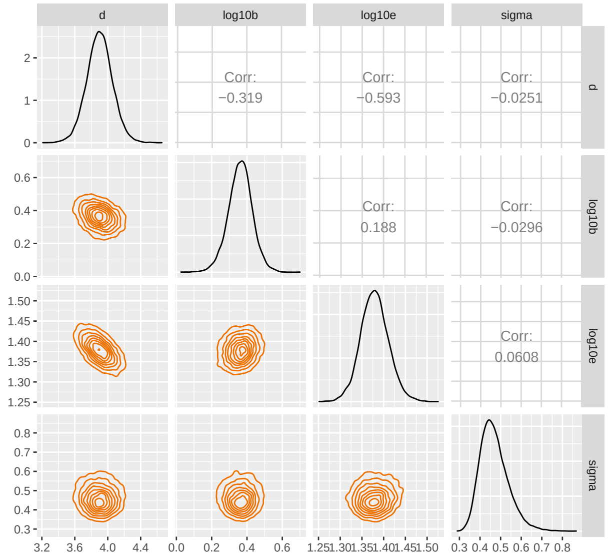

(d) Correlations between parameters

Figure 7: Dose-response curve (a), PPC (b), prior and posterior distributions (c) and correlations between parameters (d).

Data set: LYPES_VV_weight

Table 8: Summary of parameter estimates for LYPES_VV_weight data set

Parameter	median	Q2.5	Q97.5
b	9.088	3.794	65.630
d	6.341	6.044	6.638
e	15.172	14.137	15.608
sigma	1.032	0.898	1.211

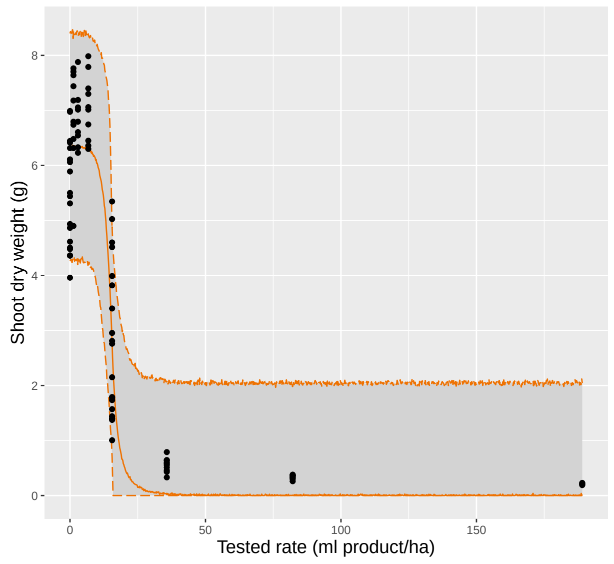

(a) Dose-response curve

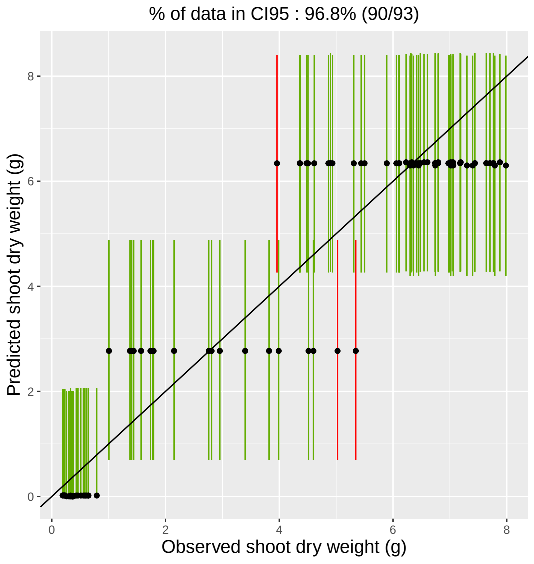

(b) Posterior predictive check (PPC)

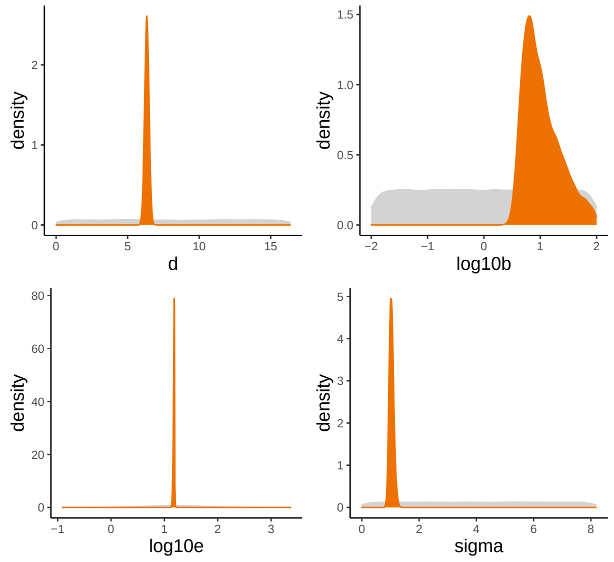

(c) Priors and posteriors

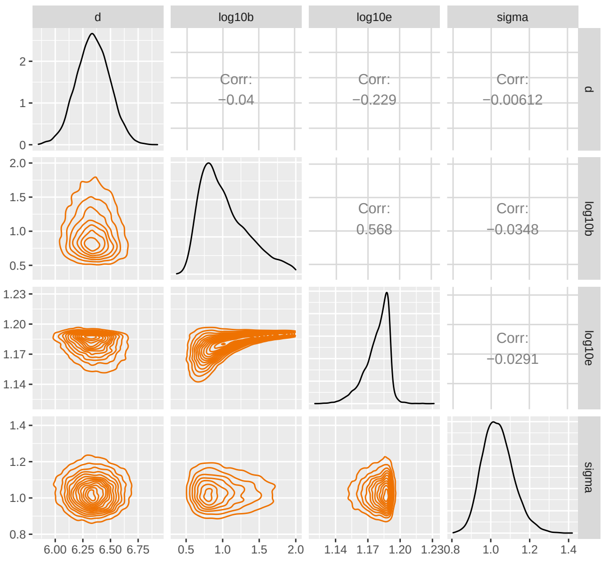

(d) Correlations between parameters

Figure 8: Dose-response curve (a), PPC (b), prior and posterior distributions (c) and correlations between parameters (d).

Data set: TRZAW_VV_weight

Table 9: Summary of parameter estimates for TRZAW_VV_weight data set

Parameter	median	Q2.5	Q97.5
b	2.260	0.927	49.330
d	1.757	1.688	1.841
e	2036.538	1042.973	4831.957
sigma	0.129	0.098	0.176

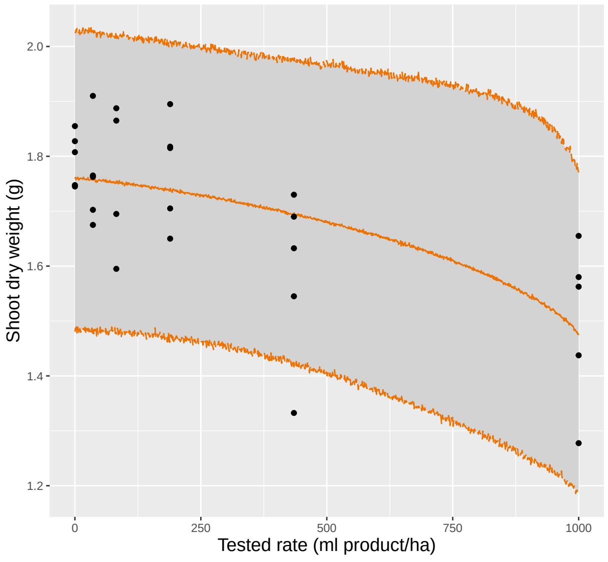

(a) Dose-response curve

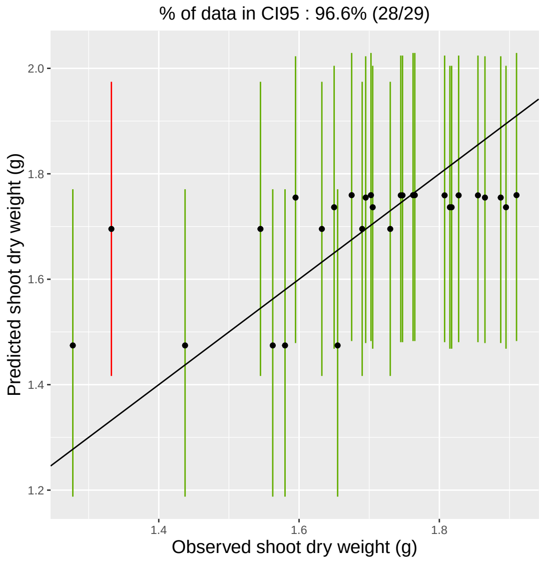

(b) Posterior predictive check (PPC)

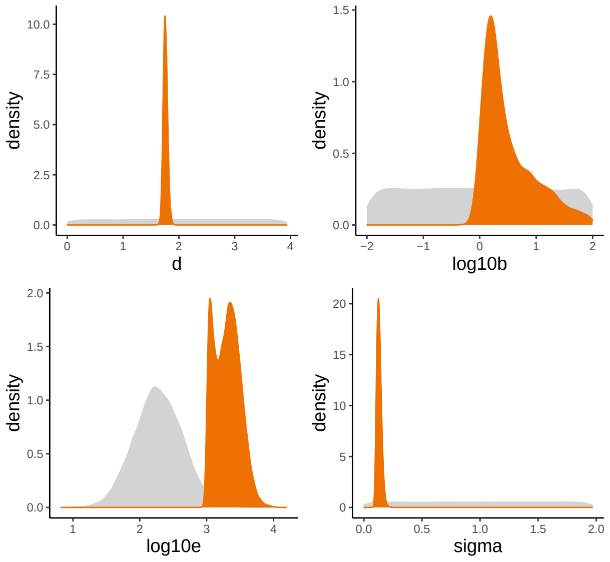

(c) Priors and posteriors

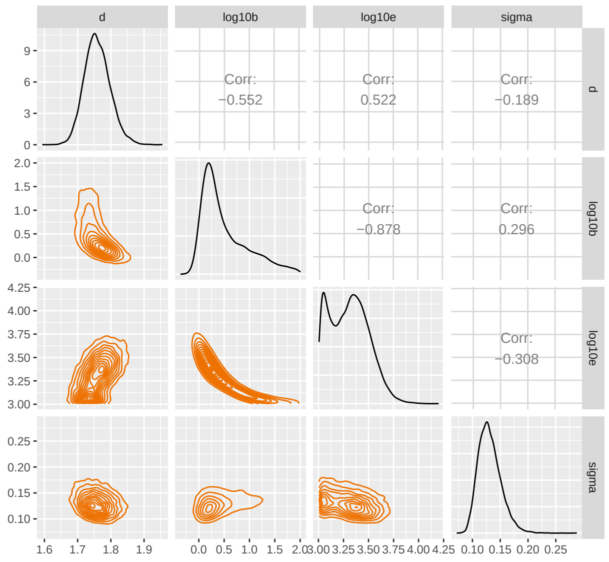

(d) Correlations between parameters

Figure 9: Dose-response curve (a), PPC (b), prior and posterior distributions (c) and correlations between parameters (d).

Data set: ZEAMA_VV_weight

Table 10: Summary of parameter estimates for ZEAMA_VV_weight data set

Parameter	median	Q2.5	Q97.5
b	2.950	2.235	5.011
d	10.714	10.269	11.160
e	403.884	367.325	443.155
sigma	1.188	0.993	1.462

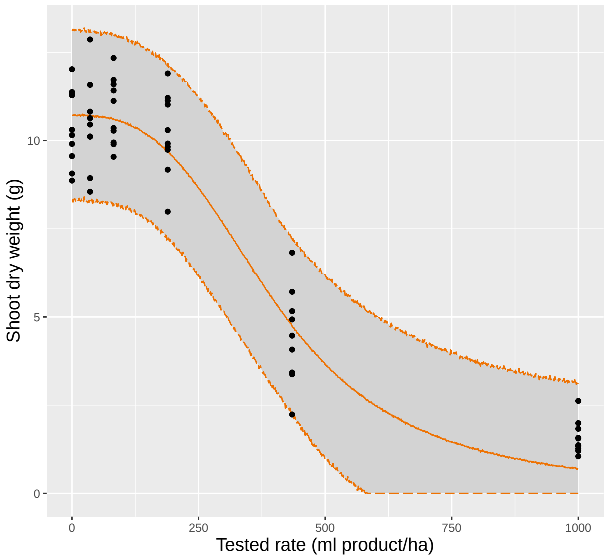

(a) Dose-response curve

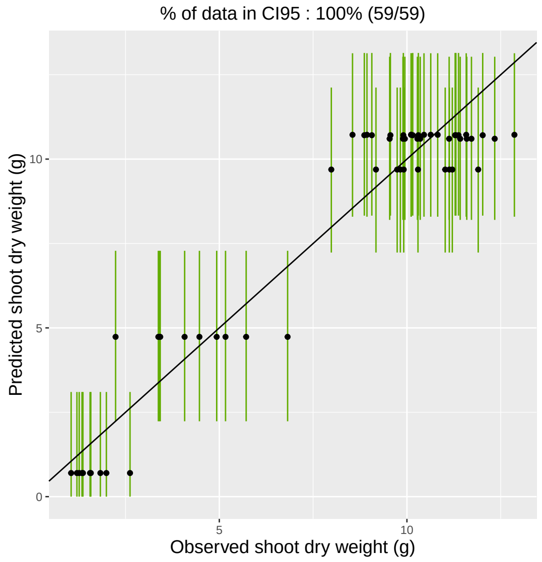

(b) Posterior predictive check (PPC)

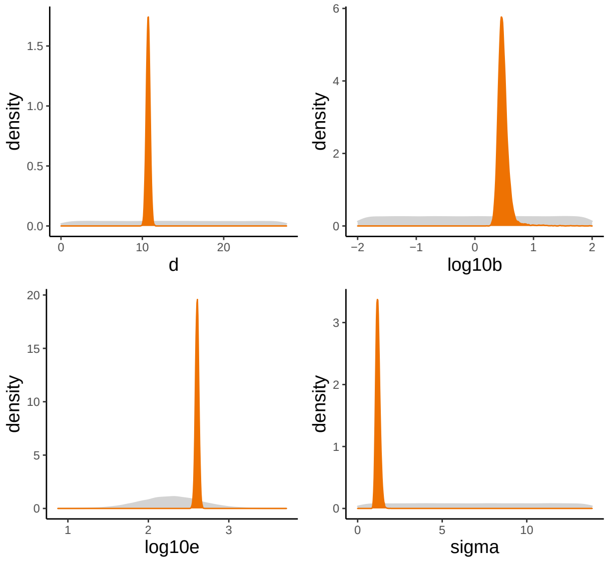

(c) Priors and posteriors

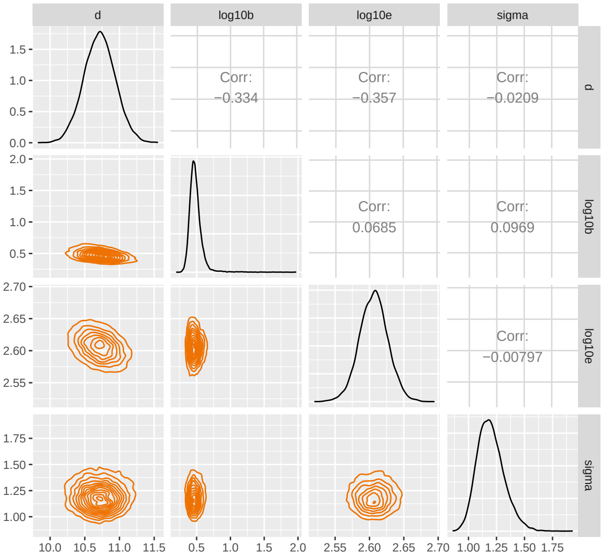

(d) Correlations between parameters

Figure 10: Dose-response curve (a), PPC (b), prior and posterior distributions (c) and correlations between parameters (d).
