# Supplementary material for: How to account for the uncertainty from standard toxicity tests in species sensitivity distributions: An example in non-target plants
Source: PLoS One. 2021 Jan 7;16(1):e0245071. doi: 10.1371/journal.pone.0245071 (PMC7790375; doi:10.1371/journal.pone.0245071)
Supplement: S1 Archive — It is a zip file containing seven folders (one folder per case study). Each folder contains five files report_xxx.pdf with detailed results of the dose-response analyses, one file corresponding to does-response analysis per endpoint. It also contains one file ER50_censoring.pdf for censored ER50 and one file SSD_analyses.pdf for results of SSD analyses. (ZIP) [file pone.0245071.s004.zip › S1_archive/Study5/ER50_censoring.pdf]

ER_{50} censoring after dose-response analyses

Study 5

25 June 2020

Contact: sandrine.charles@univ-lyon1.fr

This document provides the ER_{50} censoring after dose-response analyses for study 5, including four parts:

- Raw data description (test item rates);
- Summary on ER_{50} from a Bayesian dose-response fit;
- Censoring on ER_{50} according to two criteria;
  - Criterion 1 (C_1) based on the ratio of the probablities, that is the ratio of the probability that the ER_{50} lies within the range of the LCI95 and the maximum tested rate over the probability that the ER_{50} lies in the CI95;
  - Criterion 2 (C_2) based on the ratio of distances, that is the ratio of the distance between the LCI95 of ER_{50} and the maximum tested rate over the extend of the CI95 of ER_{50};
- Graphical presentation of censored ER_{50} per species per endpoint.

Meaning of symbols used in the document

- min_rate: minimum tested rate;
- max_rate: maximum tested rate;
- ER_{50}: 50% effective rate;
- CI95: 95% credible interval;
- ER_{50}_median: median of ER_{50};
- ER_{50}_LCI95: lower bound of the CI95 of ER_{50};
- ER_{50}_UCI95: upper bound of the CI95 of ER_{50};
- C_1: criterion based on the ratio of probabilities;
- C_2: criterion based on the ratio of distances;
- T: decision threshold;
- Censored ER_{50} [lower bound; upper bound]:
  - C1T0.0: censored ER_{50} according to C_1 with T = 0.0;
  - C1T0.2: censored ER_{50} according to C_1 with T = 0.2;

- C1T0.4: censored ER_{50} according to C_1 with T = 0.4;
- C1T0.5: censored ER_{50} according to C_1 with T = 0.5;
- C1T0.6: censored ER_{50} according to C_1 with T = 0.6;
- C1T0.8: censored ER_{50} according to C_1 with T = 0.8;
- C1T1.0: censored ER_{50} according to C_1 with T = 1.0;
- C2T0.0: censored ER_{50} according to C_2 with T = 0.0;
- C2T0.2: censored ER_{50} according to C_2 with T = 0.2;
- C2T0.4: censored ER_{50} according to C_2 with T = 0.4;
- C2T0.5: censored ER_{50} according to C_2 with T = 0.5;
- C2T0.6: censored ER_{50} according to C_2 with T = 0.6;
- C2T0.8: censored ER_{50} according to C_2 with T = 0.8;
- C2T1.0: censored ER_{50} according to C_2 with T = 1.0.

Seeding emergence (SE) test of study 5

Raw data description (test item rates) for the SE test

```
Number of species:  10
EPPO code:  ALLCE AVESA BEAVA BRSNW CUMSA GLXMA HELAN LOLPE LYPES ZEAMA
```

Table 1: Correspondance between species and common names according to the EPPO code

EPPO code	Species name	Common name
ALLCE	*Allium cepa*	Onion
AVESA	*Avena sativa*	Oat
BEAVA	*Beta vulgaris*	Sugar beet
BRSNW	*Brassica napus winter*	Oilseed rape
CUMSA	*Cucumis sativus*	Cucumber
GLXMA	*Glycine max*	Soybean
HELAN	*Helianthus annuus*	Sunflower
LOLPE	*Lolium perenne*	Perennial ryegrass
LYPES	*Solanum lycopersicum*	Tomato
ZEAMA	*Zea mays subs. Amylacea*	Corn

Table 2: Tested rates per species (g a.s./ha) for the SE test of study 5

ALLCE	AVESA	BEAVA	BRSNW	CUMSA	GLXMA	HELAN	LOLPE	LYPES	ZEAMA
0.00	0.00	0.00	0.00	0.00	0.00	0.00	0.00	0.00	0.00
0.06	0.31	0.31	0.06	0.31	0.31	0.71	0.06	0.14	0.71
0.14	0.71	0.71	0.14	0.71	0.71	1.64	0.14	0.31	1.64
0.31	1.64	1.64	0.31	1.64	1.64	3.78	0.31	0.71	3.78
0.71	3.78	3.78	0.71	3.78	3.78	8.70	0.71	1.64	8.70
1.64	8.70	8.70	1.64	8.70	8.70	20.00	1.64	3.78	20.00

SE test - emergence endpoint

Table 3: Minimum and maximum tested rates, median, quantile 2.5% and quantile 97.5% of the ER_{50} posterior distribution - emergence endpoint of the SE test of study 5

species	min_rate	max_rate	ER_{50}_median	ER_{50}_LCI95	ER_{50}_UCI95
ALLCE	0.06	1.64	1.7798989	1.4715284	3.436262
AVESA	0.31	8.70	12.1901348	9.1443014	27.693953
BEAVA	0.31	8.70	1.0385165	0.7883039	1.363658
BRSNW	0.06	1.64	1.0119606	0.8000093	1.417281
CUMSA	0.31	8.70	11.9048080	8.9067399	27.043342
GLXMA	0.31	8.70	5.9598572	4.1877554	9.939031
HELAN	0.71	20.00	11.2599297	8.2338463	16.475005
LOLPE	0.06	1.64	1.0023960	0.6803018	1.601058
LYPES	0.14	3.78	0.5648074	0.4429383	0.719484
ZEAMA	0.71	20.00	56.8813778	31.0177070	137.112966

Table 4: Censored ER_{50} according to criterion 1 and the seven decision thresholds - emergence endpoint of the SE test of study 5

species	C1T0.0	C1T0.2	C1T0.4	C1T0.5	C1T0.6	C1T0.8	C1T1.0
ALLCE	[1.47; 3.44]	[1.47; Inf)	[1.47; Inf)	[1.47; Inf)	[1.47; Inf)	[1.47; Inf)	[1.47; Inf)
AVESA	[8.7; Inf)	[8.7; Inf)	[8.7; Inf)	[8.7; Inf)	[8.7; Inf)	[8.7; Inf)	[8.7; Inf)
BEAVA	[0.79; 1.36]	[0.79; 1.36]	[0.79; 1.36]	[0.79; 1.36]	[0.79; 1.36]	[0.79; 1.36]	[0.79; 1.36]
BRSNW	[0.8; 1.42]	[0.8; 1.42]	[0.8; 1.42]	[0.8; 1.42]	[0.8; 1.42]	[0.8; 1.42]	[0.8; 1.42]
CUMSA	[8.7; Inf)	[8.7; Inf)	[8.7; Inf)	[8.7; Inf)	[8.7; Inf)	[8.7; Inf)	[8.7; Inf)
GLXMA	[4.19; 9.94]	[4.19; 9.94]	[4.19; 9.94]	[4.19; 9.94]	[4.19; 9.94]	[4.19; 9.94]	[4.19; Inf)
HELAN	[8.23; 16.48]	[8.23; 16.48]	[8.23; 16.48]	[8.23; 16.48]	[8.23; 16.48]	[8.23; 16.48]	[8.23; 16.48]
LOLPE	[0.68; 1.6]	[0.68; 1.6]	[0.68; 1.6]	[0.68; 1.6]	[0.68; 1.6]	[0.68; 1.6]	[0.68; 1.6]
LYPES	[0.44; 0.72]	[0.44; 0.72]	[0.44; 0.72]	[0.44; 0.72]	[0.44; 0.72]	[0.44; 0.72]	[0.44; 0.72]
ZEAMA	[20; Inf)	[20; Inf)	[20; Inf)	[20; Inf)	[20; Inf)	[20; Inf)	[20; Inf)

Table 5: Censored ER_{50} according to criterion 2 and the seven decision thresholds - emergence endpoint of the SE test of study 5

species	C2T0.0	C2T0.2	C2T0.4	C2T0.5	C2T0.6	C2T0.8	C2T1.0
ALLCE	[1.47; 3.44]	[1.47; Inf)	[1.47; Inf)	[1.47; Inf)	[1.47; Inf)	[1.47; Inf)	[1.47; Inf)
AVESA	[8.7; Inf)	[8.7; Inf)	[8.7; Inf)	[8.7; Inf)	[8.7; Inf)	[8.7; Inf)	[8.7; Inf)
BEAVA	[0.79; 1.36]	[0.79; 1.36]	[0.79; 1.36]	[0.79; 1.36]	[0.79; 1.36]	[0.79; 1.36]	[0.79; 1.36]
BRSNW	[0.8; 1.42]	[0.8; 1.42]	[0.8; 1.42]	[0.8; 1.42]	[0.8; 1.42]	[0.8; 1.42]	[0.8; 1.42]
CUMSA	[8.7; Inf)	[8.7; Inf)	[8.7; Inf)	[8.7; Inf)	[8.7; Inf)	[8.7; Inf)	[8.7; Inf)
GLXMA	[4.19; 9.94]	[4.19; 9.94]	[4.19; 9.94]	[4.19; 9.94]	[4.19; 9.94]	[4.19; Inf)	[4.19; Inf)
HELAN	[8.23; 16.48]	[8.23; 16.48]	[8.23; 16.48]	[8.23; 16.48]	[8.23; 16.48]	[8.23; 16.48]	[8.23; 16.48]
LOLPE	[0.68; 1.6]	[0.68; 1.6]	[0.68; 1.6]	[0.68; 1.6]	[0.68; 1.6]	[0.68; 1.6]	[0.68; 1.6]
LYPES	[0.44; 0.72]	[0.44; 0.72]	[0.44; 0.72]	[0.44; 0.72]	[0.44; 0.72]	[0.44; 0.72]	[0.44; 0.72]
ZEAMA	[20; Inf)	[20; Inf)	[20; Inf)	[20; Inf)	[20; Inf)	[20; Inf)	[20; Inf)

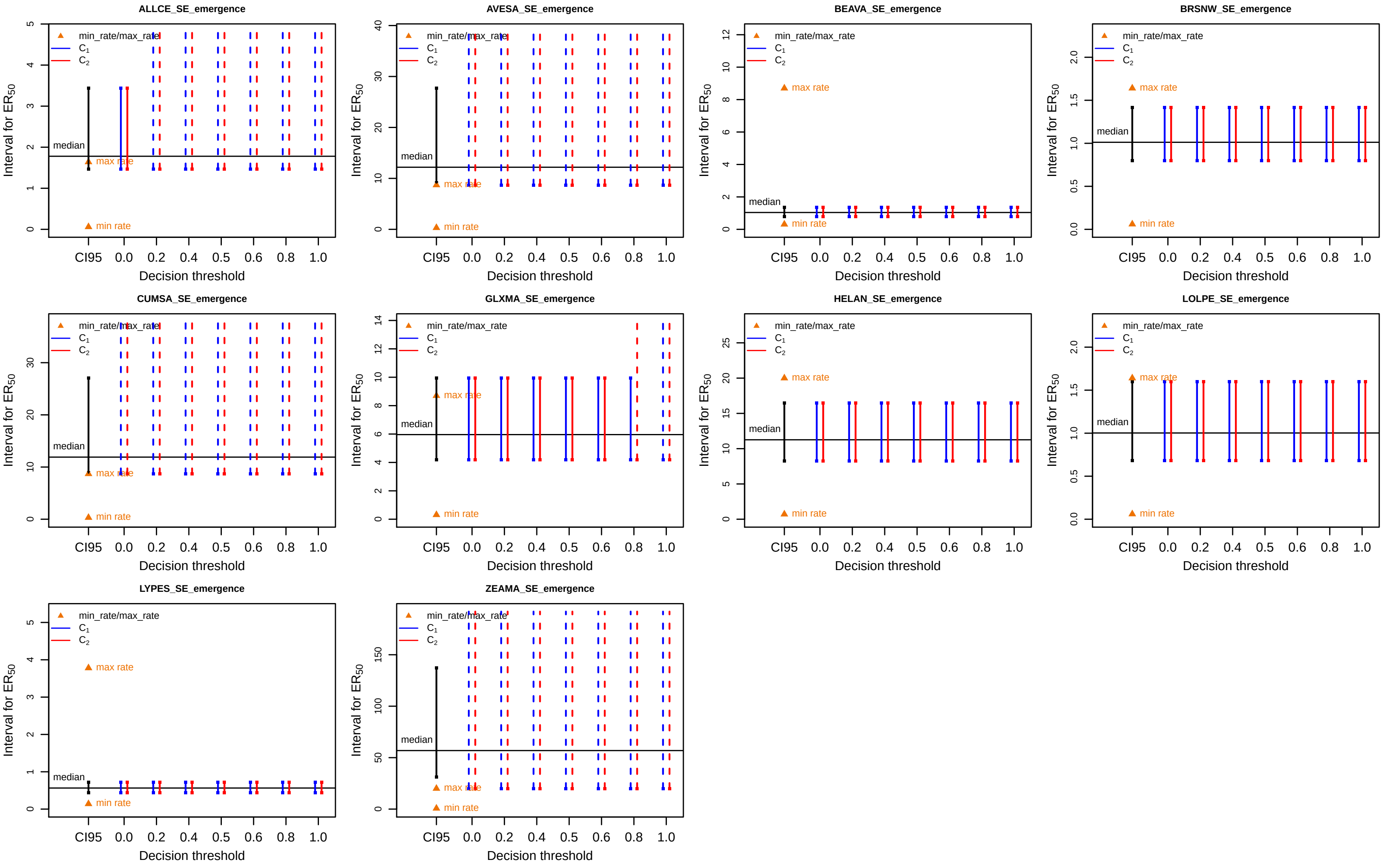

Figure 1: CI95 of ER_{50} and censored ER_{50} according to both criteria and the seven decision thresholds for all species - emergence endpoint of the SE test of study 5. The two orange triangles stand for the lowest and the highest tested rates. Vertical segments of three different colors (black, blue, red) represent the CI95 of ER_{50}, the censored ER_{50} according to C_1 and the censored ER_{50} according to C_2, respectively; solid vertical segments are for bounded intervals while dotted vertical segments stand for right-unbounded intervals; the black horizontal line represents the median of the ER_{50}.

SE test - survival endpoint

Table 6: Minimum and maximum tested rates, median, quantile 2.5% and quantile 97.5% of the ER_{50} posterior distribution - survival endpoint of the SE test of study 5

species	min_rate	max_rate	ER_{50}_median	ER_{50}_LCI95	ER_{50}_UCI95
ALLCE	0.06	1.64	1.0664773	0.7998902	1.5742948
AVESA	0.31	8.70	3.2038302	2.3493533	4.4546145
BEAVA	0.31	3.78	2.2041222	1.3081209	4.7371786
BRSNW	0.06	1.64	2.1620943	1.5512479	4.9030580
CUMSA	0.31	8.70	2.3642531	1.8994351	2.9740635
GLXMA	0.31	8.70	4.8630262	3.3392492	8.5641636
HELAN	0.71	20.00	24.0729447	14.0455066	56.1966236
LOLPE	0.06	1.64	0.3044791	0.2208635	0.4443894
LYPES	0.14	1.64	1.5424684	0.8497711	3.4439586
ZEAMA	0.71	20.00	29.1210040	21.4191832	67.5622890

Table 7: Censored ER_{50} according to criterion 1 and the seven decision thresholds - survival endpoint of the SE test of study 5

species	C1T0.0	C1T0.2	C1T0.4	C1T0.5	C1T0.6	C1T0.8	C1T1.0
ALLCE	[0.8; 1.57]	[0.8; 1.57]	[0.8; 1.57]	[0.8; 1.57]	[0.8; 1.57]	[0.8; 1.57]	[0.8; 1.57]
AVESA	[2.35; 4.45]	[2.35; 4.45]	[2.35; 4.45]	[2.35; 4.45]	[2.35; 4.45]	[2.35; 4.45]	[2.35; 4.45]
BEAVA	[1.31; 4.74]	[1.31; 4.74]	[1.31; 4.74]	[1.31; 4.74]	[1.31; 4.74]	[1.31; 4.74]	[1.31; Inf)
BRSNW	[1.55; 4.9]	[1.55; Inf)	[1.55; Inf)	[1.55; Inf)	[1.55; Inf)	[1.55; Inf)	[1.55; Inf)
CUMSA	[1.9; 2.97]	[1.9; 2.97]	[1.9; 2.97]	[1.9; 2.97]	[1.9; 2.97]	[1.9; 2.97]	[1.9; 2.97]
GLXMA	[3.34; 8.56]	[3.34; 8.56]	[3.34; 8.56]	[3.34; 8.56]	[3.34; 8.56]	[3.34; 8.56]	[3.34; 8.56]
HELAN	[14.05; 56.2]	[14.05; 56.2]	[14.05; Inf)	[14.05; Inf)	[14.05; Inf)	[14.05; Inf)	[14.05; Inf)
LOLPE	[0.22; 0.44]	[0.22; 0.44]	[0.22; 0.44]	[0.22; 0.44]	[0.22; 0.44]	[0.22; 0.44]	[0.22; 0.44]
LYPES	[0.85; 3.44]	[0.85; 3.44]	[0.85; 3.44]	[0.85; 3.44]	[0.85; Inf)	[0.85; Inf)	[0.85; Inf)
ZEAMA	[20; Inf)	[20; Inf)	[20; Inf)	[20; Inf)	[20; Inf)	[20; Inf)	[20; Inf)

Table 8: Censored ER_{50} according to criterion 2 and the seven decision thresholds - survival endpoint of the SE test of study 5

species	C2T0.0	C2T0.2	C2T0.4	C2T0.5	C2T0.6	C2T0.8	C2T1.0
ALLCE	[0.8; 1.57]	[0.8; 1.57]	[0.8; 1.57]	[0.8; 1.57]	[0.8; 1.57]	[0.8; 1.57]	[0.8; 1.57]
AVESA	[2.35; 4.45]	[2.35; 4.45]	[2.35; 4.45]	[2.35; 4.45]	[2.35; 4.45]	[2.35; 4.45]	[2.35; 4.45]
BEAVA	[1.31; 4.74]	[1.31; 4.74]	[1.31; 4.74]	[1.31; 4.74]	[1.31; 4.74]	[1.31; Inf)	[1.31; Inf)
BRSNW	[1.55; 4.9]	[1.55; Inf)	[1.55; Inf)	[1.55; Inf)	[1.55; Inf)	[1.55; Inf)	[1.55; Inf)
CUMSA	[1.9; 2.97]	[1.9; 2.97]	[1.9; 2.97]	[1.9; 2.97]	[1.9; 2.97]	[1.9; 2.97]	[1.9; 2.97]
GLXMA	[3.34; 8.56]	[3.34; 8.56]	[3.34; 8.56]	[3.34; 8.56]	[3.34; 8.56]	[3.34; 8.56]	[3.34; 8.56]
HELAN	[14.05; 56.2]	[14.05; Inf)	[14.05; Inf)	[14.05; Inf)	[14.05; Inf)	[14.05; Inf)	[14.05; Inf)
LOLPE	[0.22; 0.44]	[0.22; 0.44]	[0.22; 0.44]	[0.22; 0.44]	[0.22; 0.44]	[0.22; 0.44]	[0.22; 0.44]
LYPES	[0.85; 3.44]	[0.85; 3.44]	[0.85; Inf)	[0.85; Inf)	[0.85; Inf)	[0.85; Inf)	[0.85; Inf)
ZEAMA	[20; Inf)	[20; Inf)	[20; Inf)	[20; Inf)	[20; Inf)	[20; Inf)	[20; Inf)

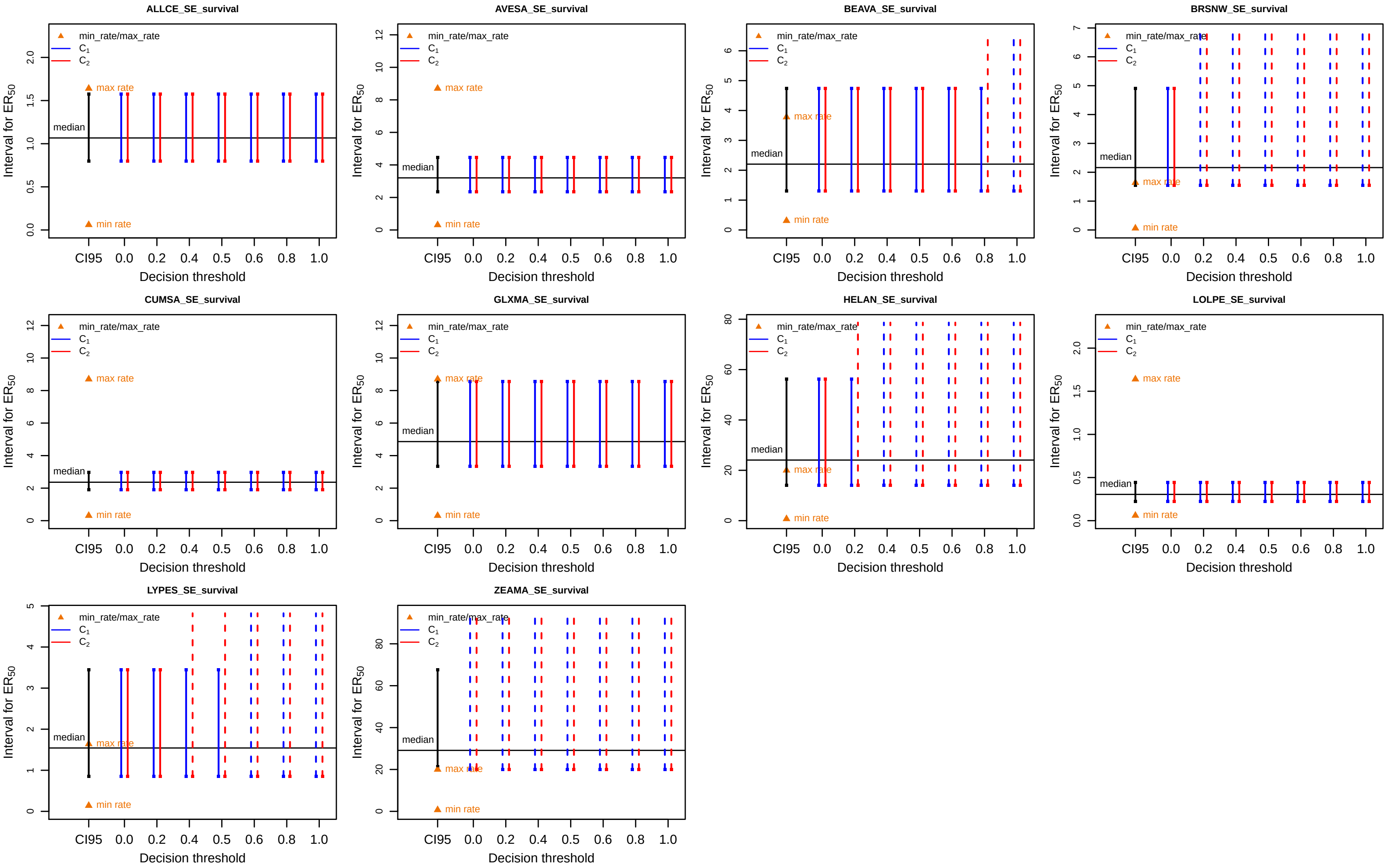

Figure 2: CI95 of ER_{50} and censored ER_{50} according to both criteria and the seven decision thresholds for all species - survival endpoint of the SE test of study 5. The two orange triangles stand for the lowest and the highest tested rates. Vertical segments of three different colors (black, blue, red) represent the CI95 of ER_{50}, the censored ER_{50} according to C_1 and the censored ER_{50} according to C_2, respectively; solid vertical segments are for bounded intervals while dotted vertical segments stand for right-unbounded intervals; the black horizontal line represents the median of the ER_{50}.

SE test - shoot dry weight endpoint

Table 9: Minimum and maximum tested rates, median, quantile 2.5% and quantile 97.5% of the ER_{50} posterior distribution - shoot dry weight endpoint of the SE test of study 5

species	min_rate	max_rate	ER_{50}_median	ER_{50}_LCI95	ER_{50}_UCI95
ALLCE	0.06	1.64	0.6480721	0.3517645	1.2167003
AVESA	0.31	8.70	4.7282231	1.5209009	13.5959393
BEAVA	0.31	3.78	1.9022893	1.3302308	3.5389054
BRSNW	0.06	1.64	1.3582213	0.9952897	1.9740726
CUMSA	0.31	3.78	3.3462115	1.7445912	7.1231272
GLXMA	0.31	8.70	7.2405108	3.7141763	17.5090073
HELAN	0.71	20.00	14.2765806	9.4745389	24.4350081
LOLPE	0.06	0.71	0.3304909	0.2103769	0.6084404
LYPES	0.14	1.64	1.5252406	0.8323109	2.7265812
ZEAMA	0.71	20.00	25.8970379	18.1053801	43.9587133

Table 10: Censored ER_{50} according to criterion 1 and the seven decision thresholds - shoot dry weight endpoint of the SE test of study 5

species	C1T0.0	C1T0.2	C1T0.4	C1T0.5	C1T0.6	C1T0.8	C1T1.0
ALLCE	[0.35; 1.22]	[0.35; 1.22]	[0.35; 1.22]	[0.35; 1.22]	[0.35; 1.22]	[0.35; 1.22]	[0.35; 1.22]
AVESA	[1.52; 13.6]	[1.52; 13.6]	[1.52; 13.6]	[1.52; 13.6]	[1.52; 13.6]	[1.52; 13.6]	[1.52; Inf)
BEAVA	[1.33; 3.54]	[1.33; 3.54]	[1.33; 3.54]	[1.33; 3.54]	[1.33; 3.54]	[1.33; 3.54]	[1.33; 3.54]
BRSNW	[1; 1.97]	[1; 1.97]	[1; 1.97]	[1; 1.97]	[1; 1.97]	[1; 1.97]	[1; Inf)
CUMSA	[1.74; 7.12]	[1.74; 7.12]	[1.74; 7.12]	[1.74; 7.12]	[1.74; 7.12]	[1.74; Inf)	[1.74; Inf)
GLXMA	[3.71; 17.51]	[3.71; 17.51]	[3.71; 17.51]	[3.71; 17.51]	[3.71; 17.51]	[3.71; Inf)	[3.71; Inf)
HELAN	[9.47; 24.44]	[9.47; 24.44]	[9.47; 24.44]	[9.47; 24.44]	[9.47; 24.44]	[9.47; 24.44]	[9.47; Inf)
LOLPE	[0.21; 0.61]	[0.21; 0.61]	[0.21; 0.61]	[0.21; 0.61]	[0.21; 0.61]	[0.21; 0.61]	[0.21; 0.61]
LYPES	[0.83; 2.73]	[0.83; 2.73]	[0.83; 2.73]	[0.83; 2.73]	[0.83; 2.73]	[0.83; Inf)	[0.83; Inf)
ZEAMA	[18.11; 43.96]	[18.11; Inf)	[18.11; Inf)	[18.11; Inf)	[18.11; Inf)	[18.11; Inf)	[18.11; Inf)

Table 11: Censored ER_{50} according to criterion 2 and the seven decision thresholds - shoot dry weight endpoint of the SE test of study 5

species	C2T0.0	C2T0.2	C2T0.4	C2T0.5	C2T0.6	C2T0.8	C2T1.0
ALLCE	[0.35; 1.22]	[0.35; 1.22]	[0.35; 1.22]	[0.35; 1.22]	[0.35; 1.22]	[0.35; 1.22]	[0.35; 1.22]
AVESA	[1.52; 13.6]	[1.52; 13.6]	[1.52; 13.6]	[1.52; 13.6]	[1.52; Inf)	[1.52; Inf)	[1.52; Inf)
BEAVA	[1.33; 3.54]	[1.33; 3.54]	[1.33; 3.54]	[1.33; 3.54]	[1.33; 3.54]	[1.33; 3.54]	[1.33; 3.54]
BRSNW	[1; 1.97]	[1; 1.97]	[1; 1.97]	[1; 1.97]	[1; 1.97]	[1; Inf)	[1; Inf)
CUMSA	[1.74; 7.12]	[1.74; 7.12]	[1.74; Inf)	[1.74; Inf)	[1.74; Inf)	[1.74; Inf)	[1.74; Inf)
GLXMA	[3.71; 17.51]	[3.71; 17.51]	[3.71; Inf)	[3.71; Inf)	[3.71; Inf)	[3.71; Inf)	[3.71; Inf)
HELAN	[9.47; 24.44]	[9.47; 24.44]	[9.47; 24.44]	[9.47; 24.44]	[9.47; 24.44]	[9.47; Inf)	[9.47; Inf)
LOLPE	[0.21; 0.61]	[0.21; 0.61]	[0.21; 0.61]	[0.21; 0.61]	[0.21; 0.61]	[0.21; 0.61]	[0.21; 0.61]
LYPES	[0.83; 2.73]	[0.83; 2.73]	[0.83; 2.73]	[0.83; Inf)	[0.83; Inf)	[0.83; Inf)	[0.83; Inf)
ZEAMA	[18.11; 43.96]	[18.11; Inf)	[18.11; Inf)	[18.11; Inf)	[18.11; Inf)	[18.11; Inf)	[18.11; Inf)

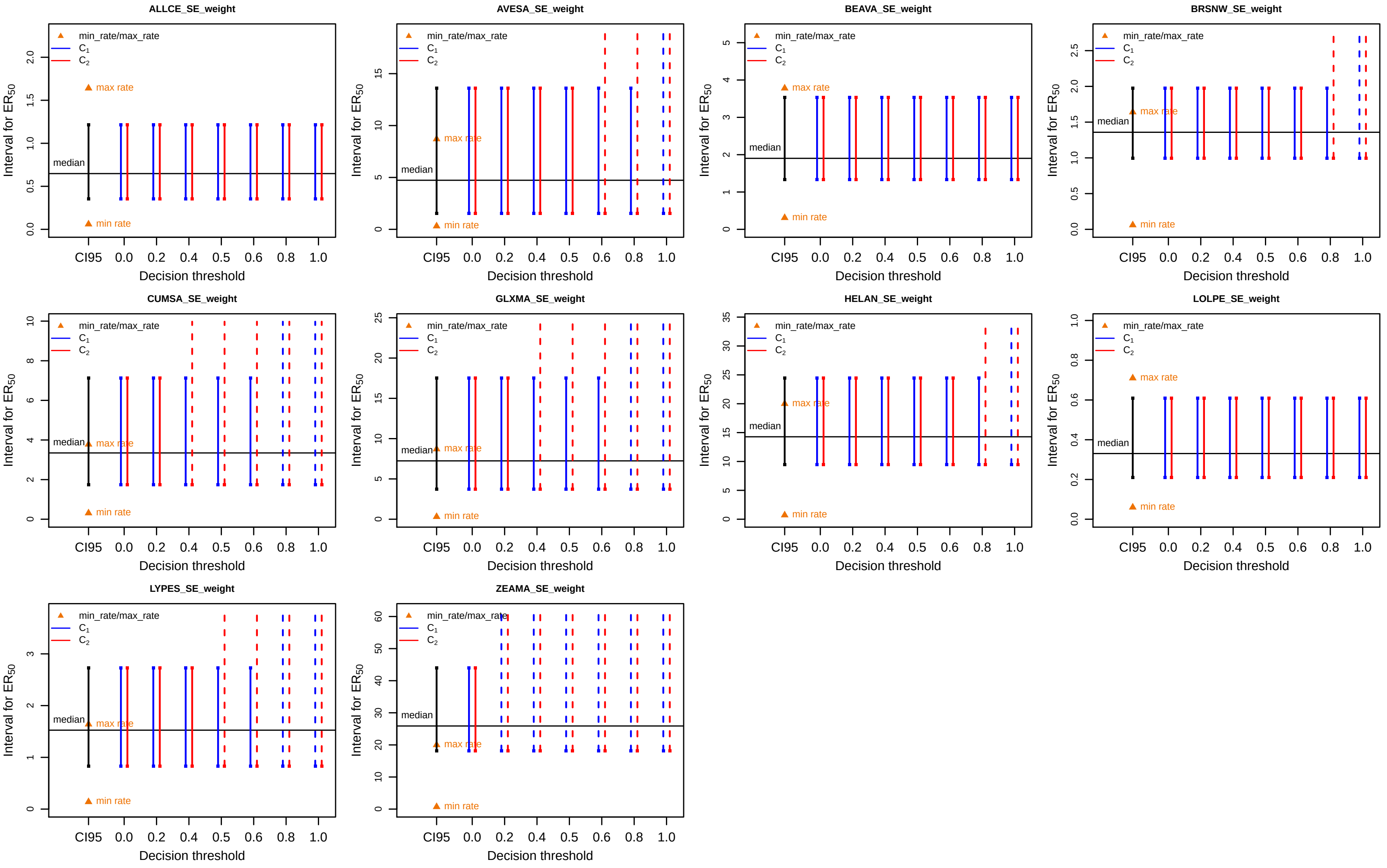

Figure 3: CI95 of ER_{50} and censored ER_{50} according to both criteria and the seven decision thresholds for all species - shoot dry weight endpoint of the SE test of study 5. The two orange triangles stand for the lowest and the highest tested rates. Vertical segments of three different colors (black, blue, red) represent the CI95 of ER_{50}, the censored ER_{50} according to C_1 and the censored ER_{50} according to C_2, respectively; solid vertical segments are for bounded intervals while dotted vertical segments stand for right-unbounded intervals; the black horizontal line represents the median of the ER_{50}.

Vegatative vigor (VV) test of study 5

Raw data description (test item rates) for VV test

```
Number of species:  10
EPPO code:  ALLCE AVESA BEAVA BRSNW CUMSA GLXMA HELAN LOLPE LYPES ZEAMA
```

Table 12: Corresponding species and common names to the EPPO code

EPPO code	Species name	Common name
ALLCE	*Allium cepa*	Onion
AVESA	*Avena sativa*	Oat
BEAVA	*Beta vulgaris*	Sugar beet
BRSNW	*Brassica napus winter*	Oilseed rape
CUMSA	*Cucumis sativus*	Cucumber
GLXMA	*Glycine max*	Soybean
HELAN	*Helianthus annuus*	Sunflower
LOLPE	*Lolium perenne*	Perennial ryegrass
LYPES	*Solanum lycopersicum*	Tomato
ZEAMA	*Zea mays subs. Amylacea*	Corn

Table 13: Tested rates per species (g a.s./ha) for the VV test of study 5

ALLCE	AVESA	BEAVA	BRSNW	CUMSA	GLXMA	HELAN	LOLPE	LYPES	ZEAMA
0.00	0.00	0.00	0.00	0.00	0.00	0.00	0.00	0.00	0.00
0.71	0.71	0.71	0.71	0.71	0.71	0.71	0.71	0.71	0.71
1.64	1.64	1.64	1.64	1.64	1.64	1.64	1.64	1.64	1.64
3.78	3.78	3.78	3.78	3.78	3.78	3.78	3.78	3.78	3.78
8.70	8.70	8.70	8.70	8.70	8.70	8.70	8.70	8.70	8.70
20.00	20.00	20.00	20.00	20.00	20.00	20.00	20.00	20.00	20.00

VV test - survival endpoint

Table 14: Minimum and maximum tested rates, median, quantile 2.5% and quantile 97.5% of the ER_{50} posterior distribution - survival endpoint of the VV test of study 5

species	min_rate	max_rate	ER_{50}_median	ER_{50}_LCI95	ER_{50}_UCI95
ALLCE	0.71	20	29.12750	21.49025	66.91813
AVESA	0.71	20	29.48479	21.57068	68.82209
BEAVA	0.71	20	22.07225	17.30363	34.06630
BRSNW	0.71	20	22.67073	20.16516	36.41978
CUMSA	0.71	20	25.51747	20.75922	54.40700
GLXMA	0.71	20	29.27164	21.46367	67.40928
HELAN	0.71	20	29.00610	21.45724	68.55465
LOLPE	0.71	20	14.70916	10.78881	22.27225
LYPES	0.71	20	25.53670	20.74360	55.32747
ZEAMA	0.71	20	29.09508	21.39812	67.29809

Table 15: Censored ER_{50} according to criterion 1 and the seven decision thresholds - survival endpoint of the VV test of study 5

species	C1T0.0	C1T0.2	C1T0.4	C1T0.5	C1T0.6	C1T0.8	C1T1.0
ALLCE	[20; Inf)	[20; Inf)	[20; Inf)	[20; Inf)	[20; Inf)	[20; Inf)	[20; Inf)
AVESA	[20; Inf)	[20; Inf)	[20; Inf)	[20; Inf)	[20; Inf)	[20; Inf)	[20; Inf)
BEAVA	[17.3; 34.07]	[17.3; 34.07]	[17.3; Inf)	[17.3; Inf)	[17.3; Inf)	[17.3; Inf)	[17.3; Inf)
BRSNW	[20; Inf)	[20; Inf)	[20; Inf)	[20; Inf)	[20; Inf)	[20; Inf)	[20; Inf)
CUMSA	[20; Inf)	[20; Inf)	[20; Inf)	[20; Inf)	[20; Inf)	[20; Inf)	[20; Inf)
GLXMA	[20; Inf)	[20; Inf)	[20; Inf)	[20; Inf)	[20; Inf)	[20; Inf)	[20; Inf)
HELAN	[20; Inf)	[20; Inf)	[20; Inf)	[20; Inf)	[20; Inf)	[20; Inf)	[20; Inf)
LOLPE	[10.79; 22.27]	[10.79; 22.27]	[10.79; 22.27]	[10.79; 22.27]	[10.79; 22.27]	[10.79; 22.27]	[10.79; Inf)
LYPES	[20; Inf)	[20; Inf)	[20; Inf)	[20; Inf)	[20; Inf)	[20; Inf)	[20; Inf)
ZEAMA	[20; Inf)	[20; Inf)	[20; Inf)	[20; Inf)	[20; Inf)	[20; Inf)	[20; Inf)

Table 16: Censored ER_{50} according to criterion 2 and the seven decision thresholds - survival endpoint of the VV test of study 5

species	C2T0.0	C2T0.2	C2T0.4	C2T0.5	C2T0.6	C2T0.8	C2T1.0
ALLCE	[20; Inf)	[20; Inf)	[20; Inf)	[20; Inf)	[20; Inf)	[20; Inf)	[20; Inf)
AVESA	[20; Inf)	[20; Inf)	[20; Inf)	[20; Inf)	[20; Inf)	[20; Inf)	[20; Inf)
BEAVA	[17.3; 34.07]	[17.3; Inf)	[17.3; Inf)	[17.3; Inf)	[17.3; Inf)	[17.3; Inf)	[17.3; Inf)
BRSNW	[20; Inf)	[20; Inf)	[20; Inf)	[20; Inf)	[20; Inf)	[20; Inf)	[20; Inf)
CUMSA	[20; Inf)	[20; Inf)	[20; Inf)	[20; Inf)	[20; Inf)	[20; Inf)	[20; Inf)
GLXMA	[20; Inf)	[20; Inf)	[20; Inf)	[20; Inf)	[20; Inf)	[20; Inf)	[20; Inf)
HELAN	[20; Inf)	[20; Inf)	[20; Inf)	[20; Inf)	[20; Inf)	[20; Inf)	[20; Inf)
LOLPE	[10.79; 22.27]	[10.79; 22.27]	[10.79; 22.27]	[10.79; 22.27]	[10.79; 22.27]	[10.79; 22.27]	[10.79; Inf)
LYPES	[20; Inf)	[20; Inf)	[20; Inf)	[20; Inf)	[20; Inf)	[20; Inf)	[20; Inf)
ZEAMA	[20; Inf)	[20; Inf)	[20; Inf)	[20; Inf)	[20; Inf)	[20; Inf)	[20; Inf)

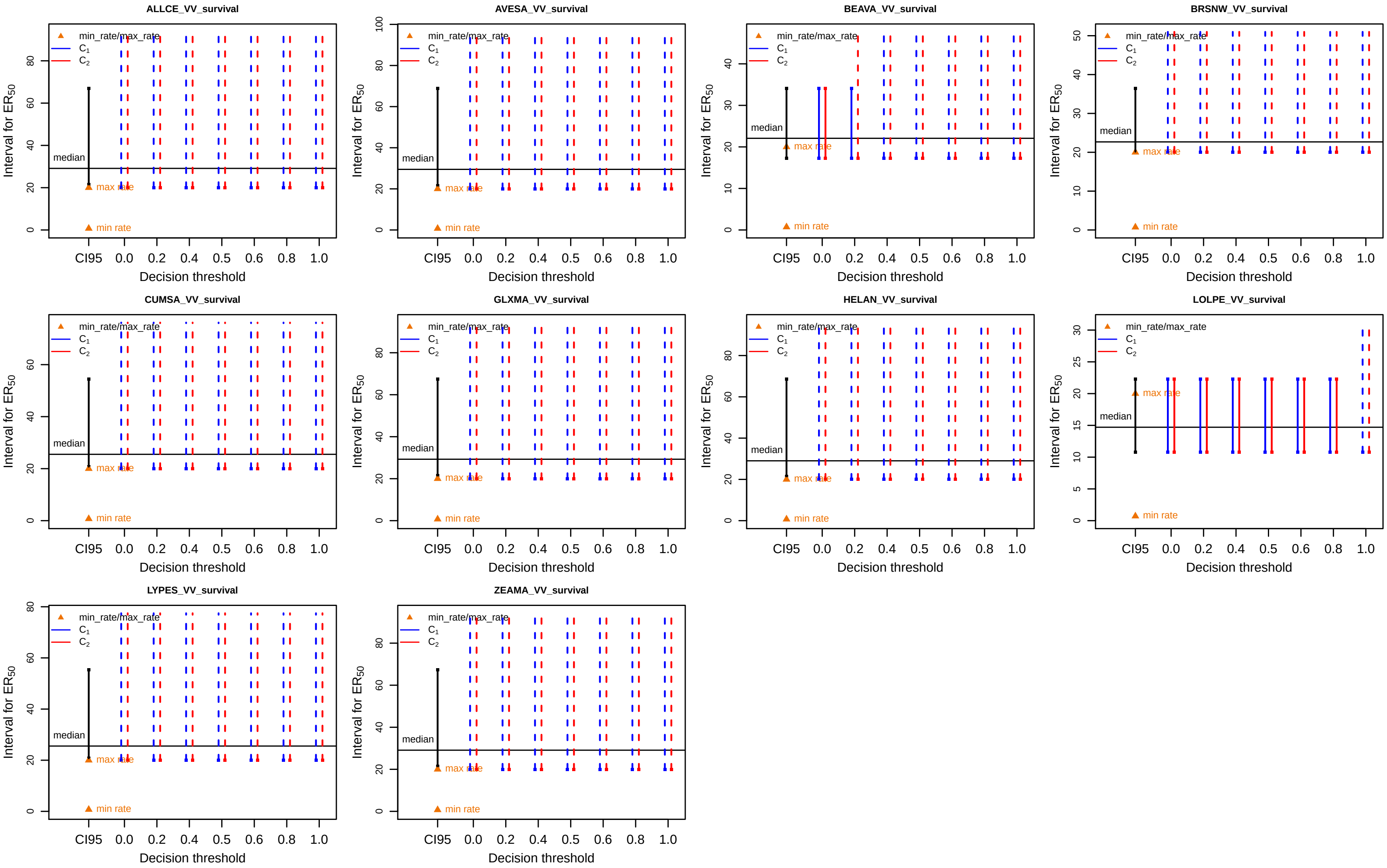

Figure 4: CI95 of ER_{50} and censored ER_{50} according to both criteria and the seven decision thresholds for all species - survival endpoint of the VV test of study 5. The two orange triangles stand for the lowest and the highest tested rates. Vertical segments of three different colors (black, blue, red) represent the CI95 of ER_{50}, the censored ER_{50} according to C_1 and the censored ER_{50} according to C_2, respectively; solid vertical segments are for bounded intervals while dotted vertical segments stand for right-unbounded intervals; the black horizontal line represents the median of the ER_{50}.

VV test - shoot dry weight endpoint

Table 17: Minimum and maximum tested rates, median, quantile 2.5% and quantile 97.5% of the ER_{50} posterior distribution - shoot dry weight endpoint of the VV test of study 5

species	min_rate	max_rate	ER_{50}_median	ER_{50}_LCI95	ER_{50}_UCI95
ALLCE	0.71	20	27.68189	20.43390	63.57449
AVESA	0.71	20	35.22214	20.74155	91.85155
BEAVA	0.71	20	77.45164	23.49213	202.77502
BRSNW	0.71	20	16.32283	12.90296	21.35944
CUMSA	0.71	20	39.48567	18.51737	95.86113
GLXMA	0.71	20	27.21835	20.79535	75.76000
HELAN	0.71	20	29.09447	21.46366	69.35618
LOLPE	0.71	20	10.85255	5.83366	20.12310
LYPES	0.71	20	26.85860	20.64803	83.43055
ZEAMA	0.71	20	71.24945	39.84040	150.22693

Table 18: Censored ER_{50} according to criterion 1 and the seven decision thresholds - shoot dry weight endpoint of the VV test of study 5

species	C1T0.0	C1T0.2	C1T0.4	C1T0.5	C1T0.6	C1T0.8	C1T1.0
ALLCE	[20; Inf)	[20; Inf)	[20; Inf)	[20; Inf)	[20; Inf)	[20; Inf)	[20; Inf)
AVESA	[20; Inf)	[20; Inf)	[20; Inf)	[20; Inf)	[20; Inf)	[20; Inf)	[20; Inf)
BEAVA	[20; Inf)	[20; Inf)	[20; Inf)	[20; Inf)	[20; Inf)	[20; Inf)	[20; Inf)
BRSNW	[12.9; 21.36]	[12.9; 21.36]	[12.9; 21.36]	[12.9; 21.36]	[12.9; 21.36]	[12.9; 21.36]	[12.9; Inf)
CUMSA	[18.52; 95.86]	[18.52; Inf)	[18.52; Inf)	[18.52; Inf)	[18.52; Inf)	[18.52; Inf)	[18.52; Inf)
GLXMA	[20; Inf)	[20; Inf)	[20; Inf)	[20; Inf)	[20; Inf)	[20; Inf)	[20; Inf)
HELAN	[20; Inf)	[20; Inf)	[20; Inf)	[20; Inf)	[20; Inf)	[20; Inf)	[20; Inf)
LOLPE	[5.83; 20.12]	[5.83; 20.12]	[5.83; 20.12]	[5.83; 20.12]	[5.83; 20.12]	[5.83; 20.12]	[5.83; Inf)
LYPES	[20; Inf)	[20; Inf)	[20; Inf)	[20; Inf)	[20; Inf)	[20; Inf)	[20; Inf)
ZEAMA	[20; Inf)	[20; Inf)	[20; Inf)	[20; Inf)	[20; Inf)	[20; Inf)	[20; Inf)

Table 19: Censored ER_{50} according to criterion 2 and the seven decision thresholds - shoot dry weight endpoint of the VV test of study 5

species	C2T0.0	C2T0.2	C2T0.4	C2T0.5	C2T0.6	C2T0.8	C2T1.0
ALLCE	[20; Inf)	[20; Inf)	[20; Inf)	[20; Inf)	[20; Inf)	[20; Inf)	[20; Inf)
AVESA	[20; Inf)	[20; Inf)	[20; Inf)	[20; Inf)	[20; Inf)	[20; Inf)	[20; Inf)
BEAVA	[20; Inf)	[20; Inf)	[20; Inf)	[20; Inf)	[20; Inf)	[20; Inf)	[20; Inf)
BRSNW	[12.9; 21.36]	[12.9; 21.36]	[12.9; 21.36]	[12.9; 21.36]	[12.9; 21.36]	[12.9; 21.36]	[12.9; Inf)
CUMSA	[18.52; 95.86]	[18.52; Inf)	[18.52; Inf)	[18.52; Inf)	[18.52; Inf)	[18.52; Inf)	[18.52; Inf)
GLXMA	[20; Inf)	[20; Inf)	[20; Inf)	[20; Inf)	[20; Inf)	[20; Inf)	[20; Inf)
HELAN	[20; Inf)	[20; Inf)	[20; Inf)	[20; Inf)	[20; Inf)	[20; Inf)	[20; Inf)
LOLPE	[5.83; 20.12]	[5.83; 20.12]	[5.83; 20.12]	[5.83; 20.12]	[5.83; 20.12]	[5.83; 20.12]	[5.83; Inf)
LYPES	[20; Inf)	[20; Inf)	[20; Inf)	[20; Inf)	[20; Inf)	[20; Inf)	[20; Inf)
ZEAMA	[20; Inf)	[20; Inf)	[20; Inf)	[20; Inf)	[20; Inf)	[20; Inf)	[20; Inf)

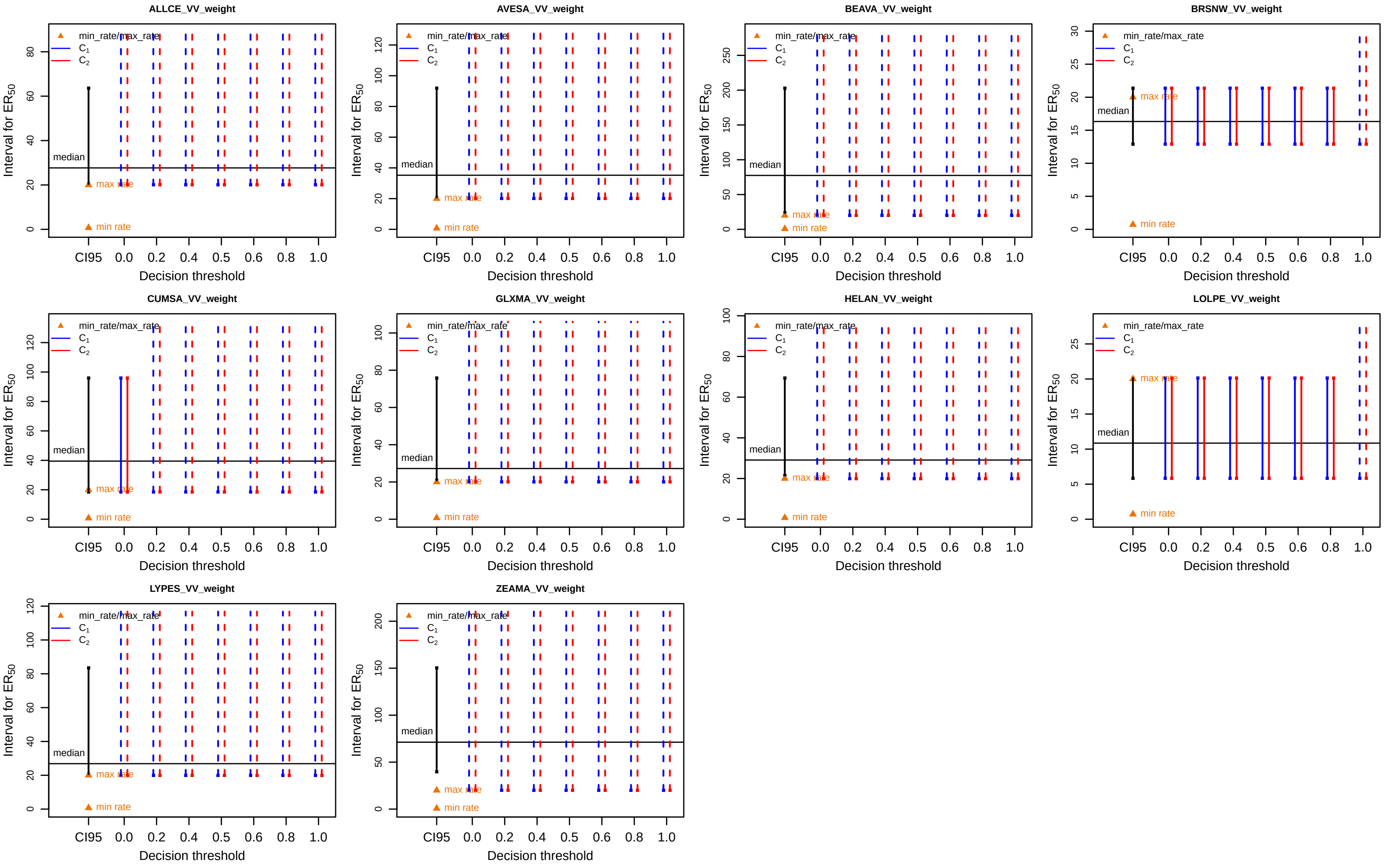

Figure 5: CI95 of ER_{50} and censored ER_{50} according to both criteria and the seven decision thresholds for all species - shoot dry weight endpoint of the VV test of study 5. The two orange triangles stand for the lowest and the highest tested rates. Vertical segments of three different colors (black, blue, red) represent the CI95 of ER_{50}, the censored ER_{50} according to C_1 and the censored ER_{50} according to C_2, respectively; solid vertical segments are for bounded intervals while dotted vertical segments stand for right-unbounded intervals; the black horizontal line represents the median of the ER_{50}.
