# Supplementary material for: How to account for the uncertainty from standard toxicity tests in species sensitivity distributions: An example in non-target plants
Source: PLoS One. 2021 Jan 7;16(1):e0245071. doi: 10.1371/journal.pone.0245071 (PMC7790375; doi:10.1371/journal.pone.0245071)
Supplement: S1 Archive — It is a zip file containing seven folders (one folder per case study). Each folder contains five files report_xxx.pdf with detailed results of the dose-response analyses, one file corresponding to does-response analysis per endpoint. It also contains one file ER50_censoring.pdf for censored ER50 and one file SSD_analyses.pdf for results of SSD analyses. (ZIP) [file pone.0245071.s004.zip › S1_archive/Study5/report_SE_emergence.pdf]

Dose-response analyses

Study 5

Seedling Emergence test - emergence endpoint

25 June 2020

Contact: sandrine.charles@univ-lyon1.fr

This is a report which provides results on all performed dose-response analyses for the emergence endpoint of the Seedling Emergence test for study 5.

Contents

Data set: ALLCE_SE_emergence 2
Data set: AVESA_SE_emergence 3
Data set: BEAVA_SE_emergence 4
Data set: BRSNW_SE_emergence 5
Data set: CUMSA_SE_emergence 6
Data set: GLXMA_SE_emergence 7
Data set: HELAN_SE_emergence 8
Data set: LOLPE_SE_emergence 9
Data set: LYPES_SE_emergence 10
Data set: ZEAMA_SE_emergence 11

Data set: ALLCE_SE_emergence

Table 1: Summary of parameter estimates for ALLCE_SE_emergence data set

Parameter	median	Q2.5	Q97.5
b	5.748	1.380	66.785
d	0.786	0.695	0.862
e	1.780	1.472	3.436

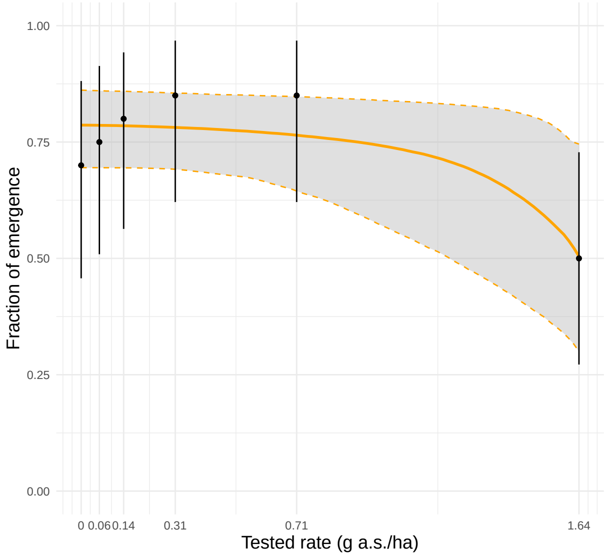

(a) Dose-response curve

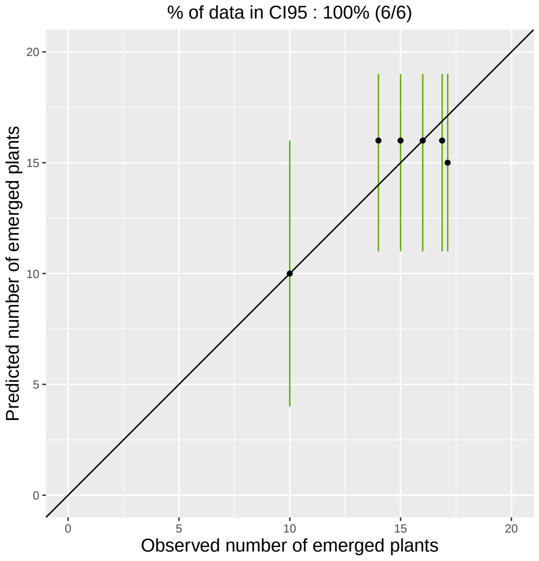

(b) Posterior predictive check (PPC)

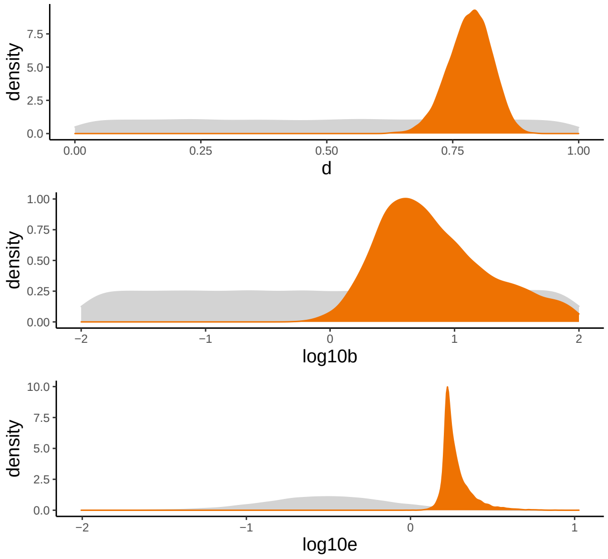

(c) Priors and posteriors

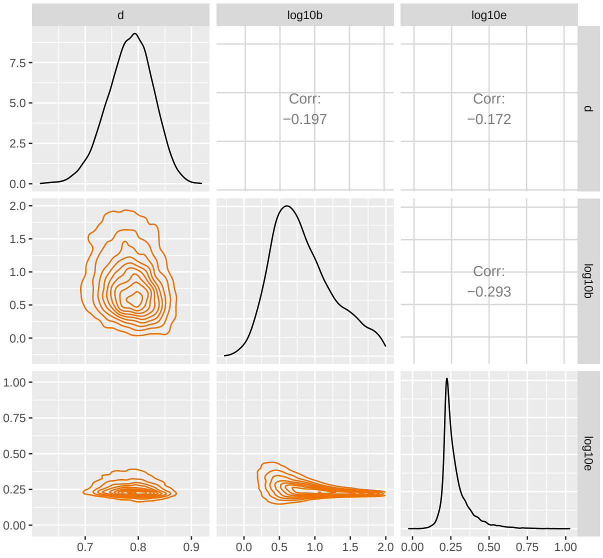

(d) Correlations between parameters

Figure 1: Dose-response curve (a), PPC (b), prior and posterior distributions (c) and correlations between parameters (d).

Data set: AVESA_SE_emergence

Table 2: Summary of parameter estimates for AVESA_SE_emergence data set

Parameter	median	Q2.5	Q97.5
b	24.649	3.344	93.220
d	0.981	0.945	0.997
e	12.190	9.144	27.694

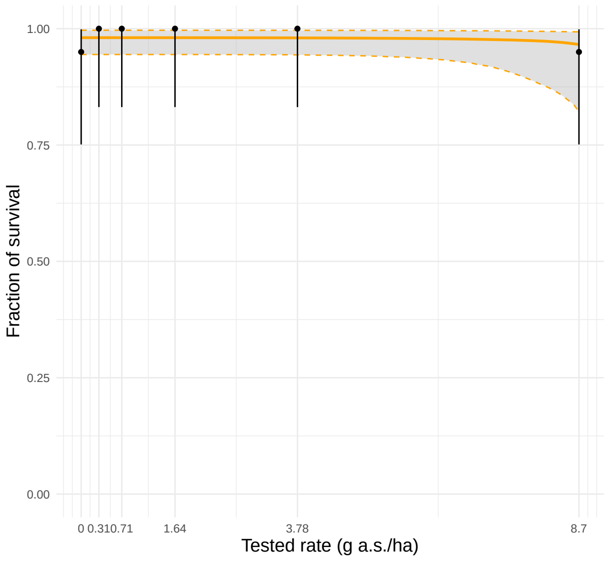

(a) Dose-response curve

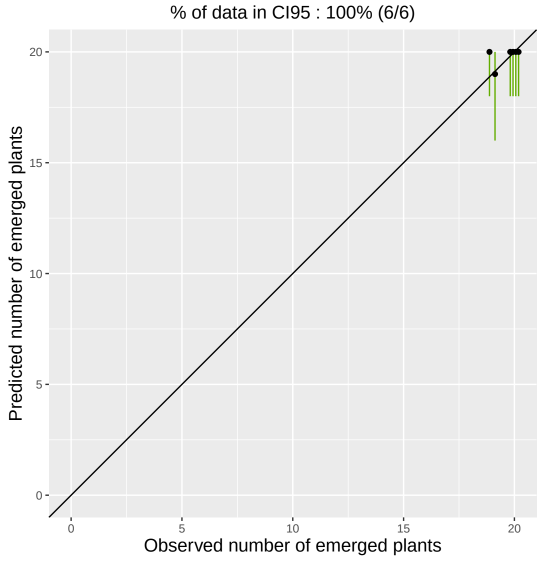

(b) Posterior predictive check (PPC)

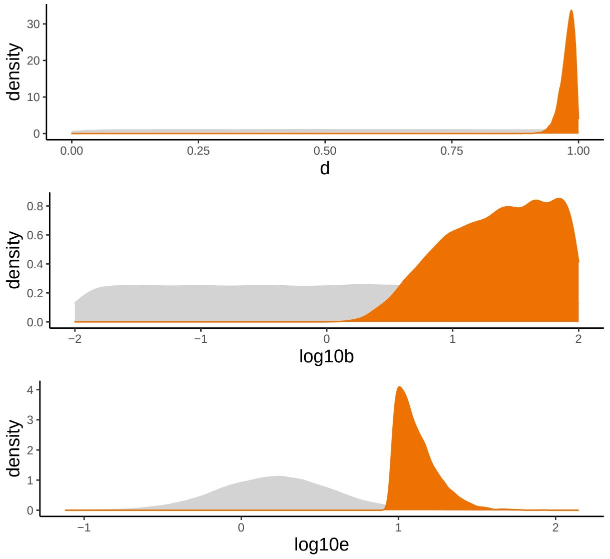

(c) Priors and posteriors

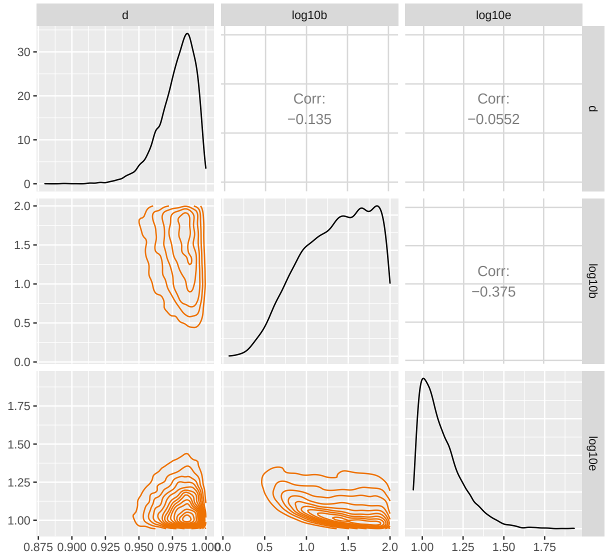

(d) Correlations between parameters

Figure 2: Dose-response curve (a), PPC (b), prior and posterior distributions (c) and correlations between parameters (d).

Data set: BEAVA_SE_emergence

Table 3: Summary of parameter estimates (parameter d is set to 1) for BEAVA_SE_emergence data set

Parameter	median	Q2.5	Q97.5
b	2.339	1.552	3.353
e	1.039	0.788	1.364

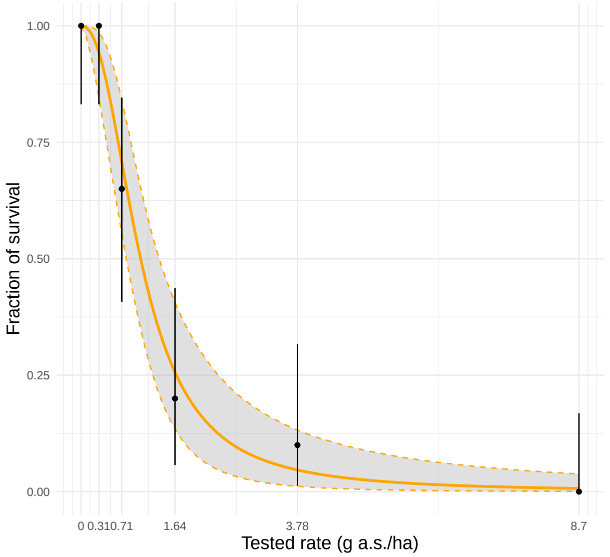

(a) Dose-response curve

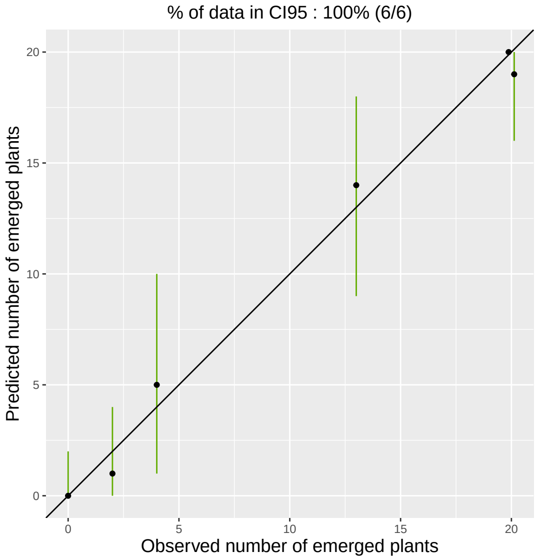

(b) Posterior predictive check (PPC)

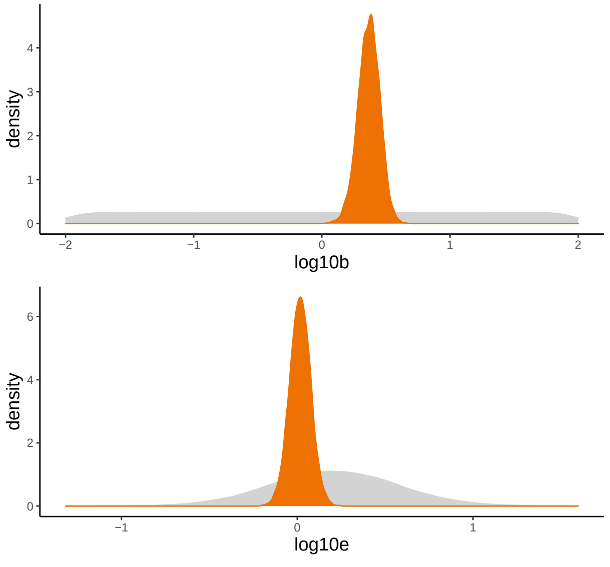

(c) Priors and posteriors

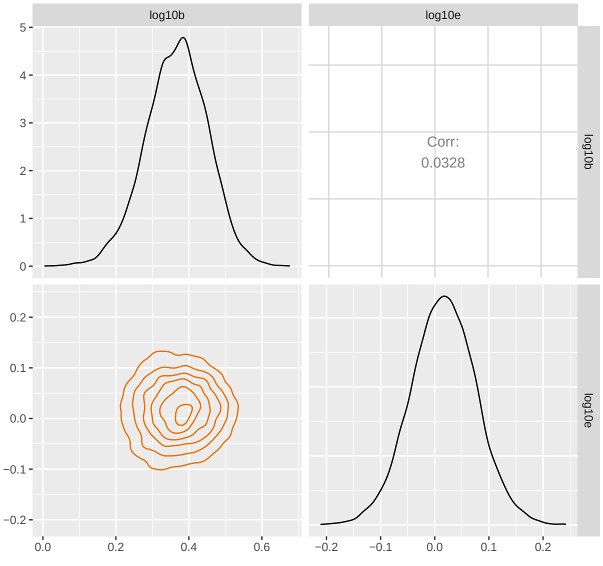

(d) Correlations between parameters

Figure 3: Dose-response curve (a), PPC (b), prior and posterior distributions (c) and correlations between parameters (d).

Data set: BRSNW_SE_emergence

Table 4: Summary of parameter estimates for BRSNW_SE_emergence data set

Parameter	median	Q2.5	Q97.5
b	4.407	2.338	12.679
d	0.956	0.892	0.989
e	1.012	0.800	1.417

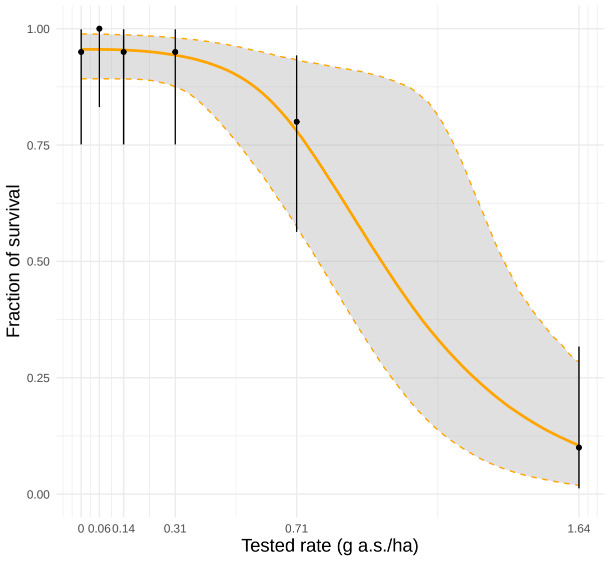

(a) Dose-response curve

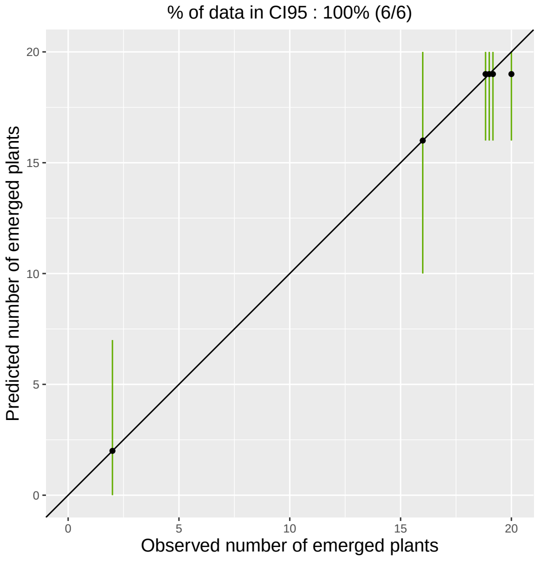

(b) Posterior predictive check (PPC)

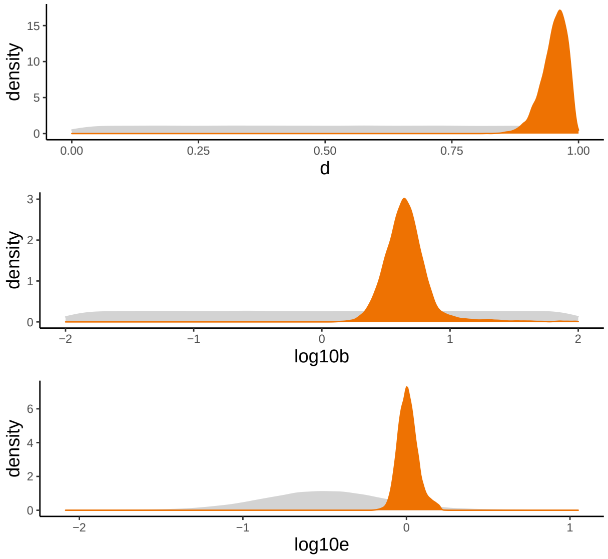

(c) Priors and posteriors

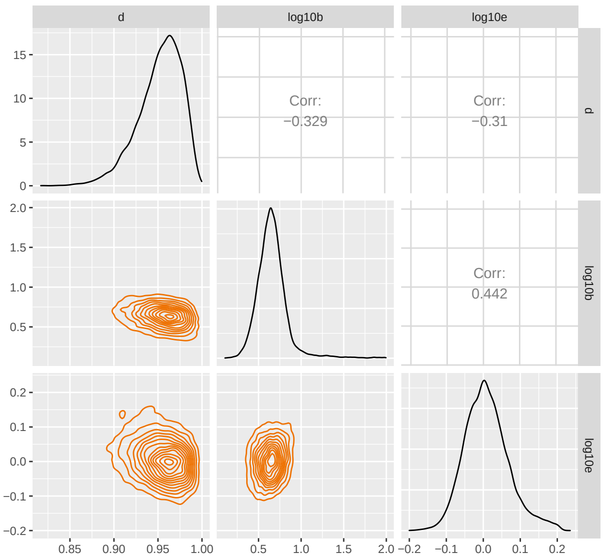

(d) Correlations between parameters

Figure 4: Dose-response curve (a), PPC (b), prior and posterior distributions (c) and correlations between parameters (d).

Data set: CUMSA_SE_emergence

Table 5: Summary of parameter estimates for CUMSA_SE_emergence data set

Parameter	median	Q2.5	Q97.5
b	7.652	0.931	85.180
d	0.877	0.799	0.955
e	11.905	8.907	27.043

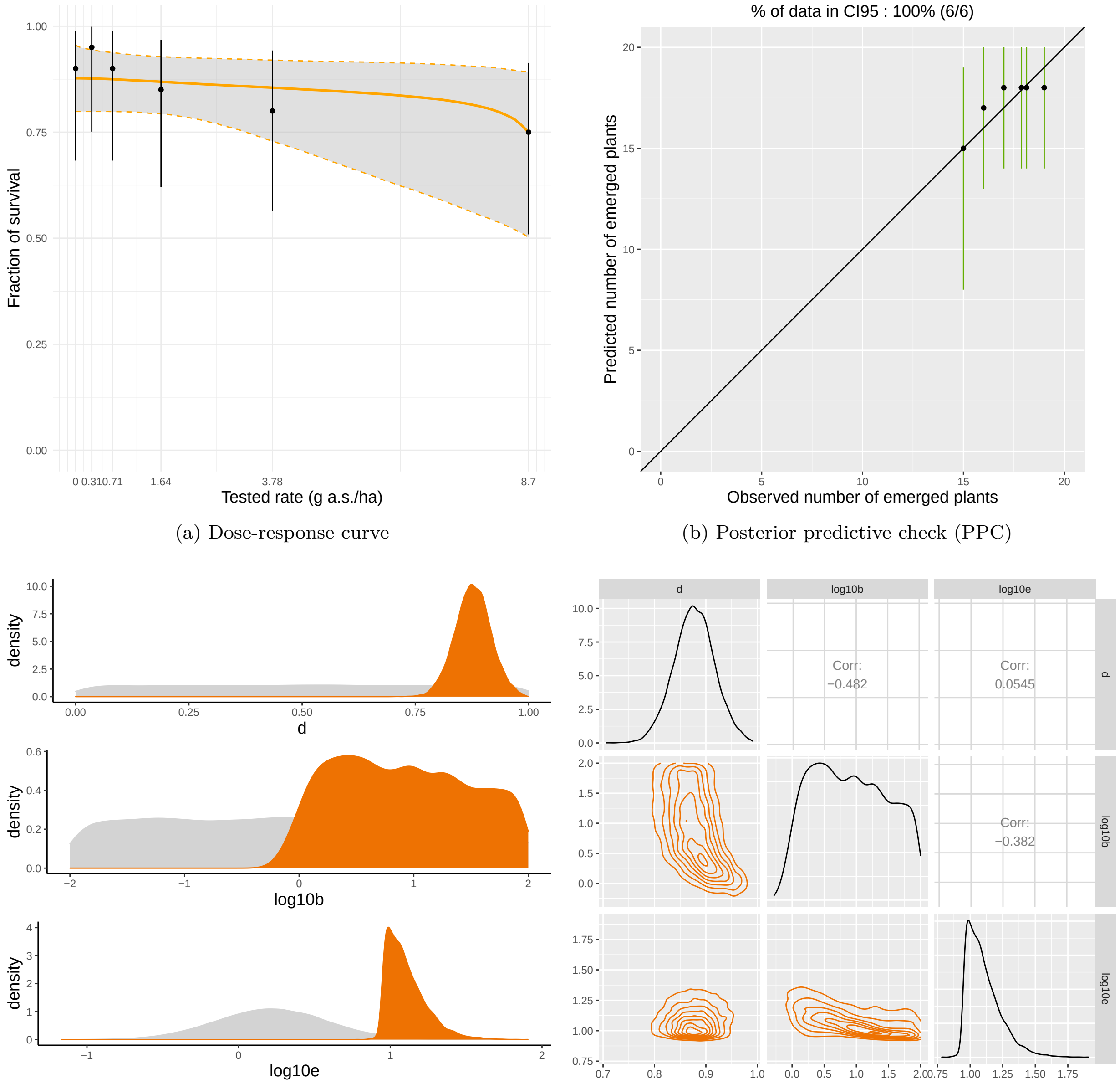

(a) Dose-response curve

(b) Posterior predictive check (PPC)

(c) Priors and posteriors

(d) Correlations between parameters

Figure 5: Dose-response curve (a), PPC (b), prior and posterior distributions (c) and correlations between parameters (d).

Data set: GLXMA_SE_emergence

Table 6: Summary of parameter estimates (parameter d is set to 1) for GLXMA_SE_emergence data set

Parameter	median	Q2.5	Q97.5
b	1.492	0.894	2.260
e	5.960	4.188	9.939

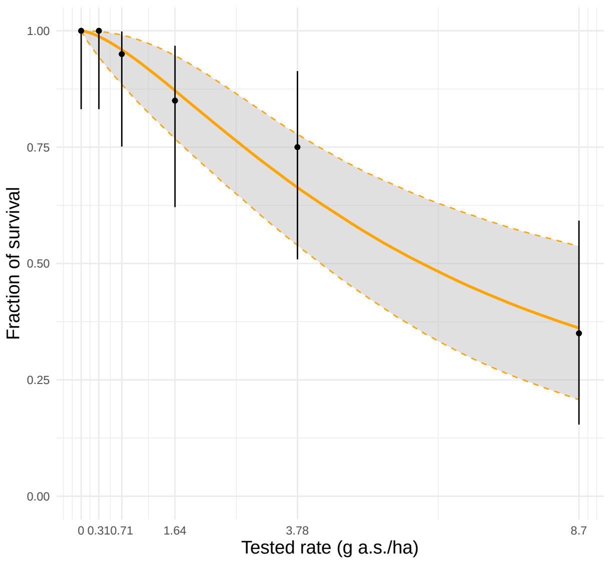

(a) Dose-response curve

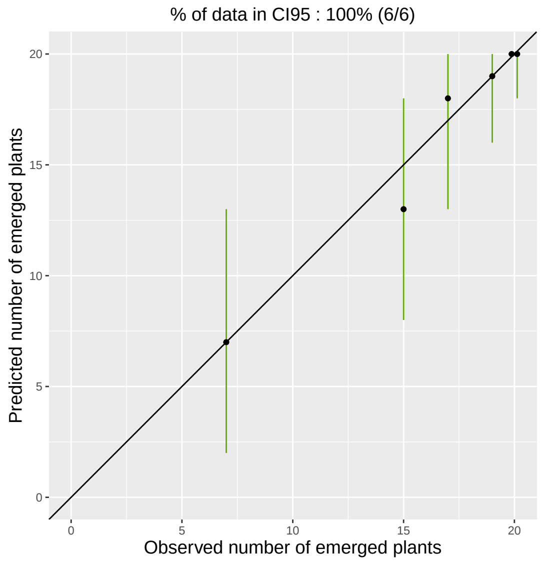

(b) Posterior predictive check (PPC)

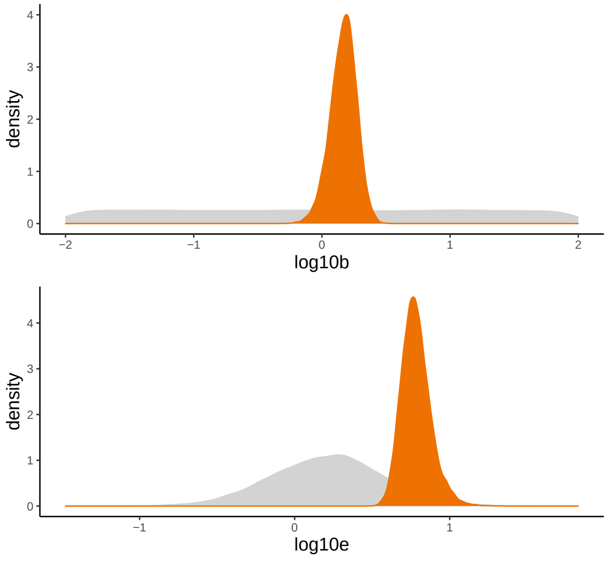

(c) Priors and posteriors

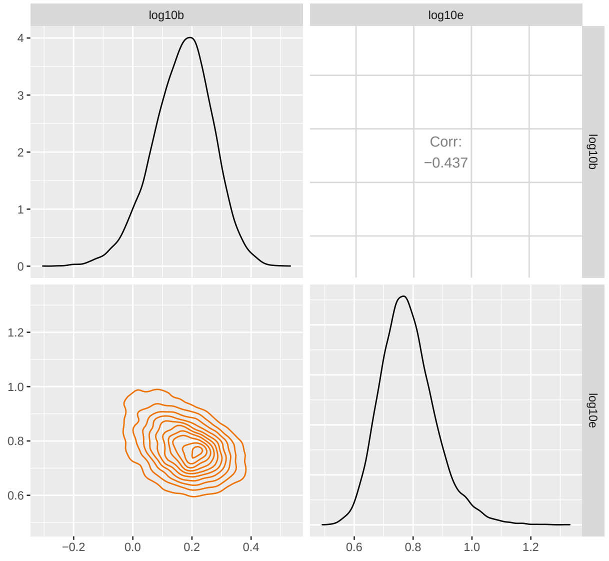

(d) Correlations between parameters

Figure 6: Dose-response curve (a), PPC (b), prior and posterior distributions (c) and correlations between parameters (d).

Data set: HELAN_SE_emergence

Table 7: Summary of parameter estimates for HELAN_SE_emergence data set

Parameter	median	Q2.5	Q97.5
b	1.961	1.214	2.977
d	0.960	0.896	0.990
e	11.260	8.234	16.475

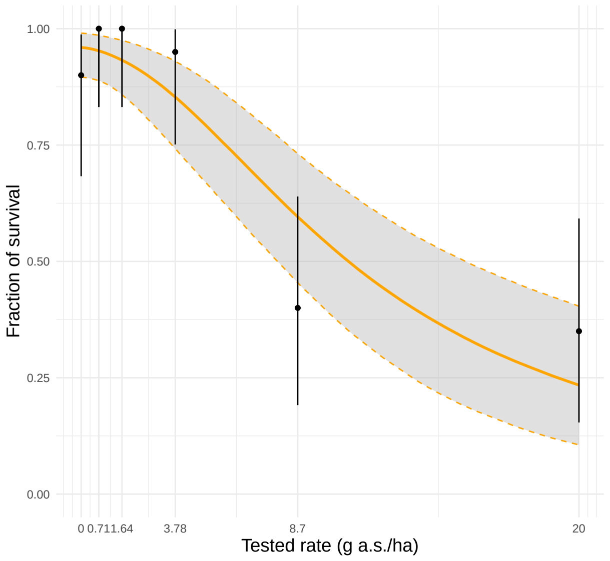

(a) Dose-response curve

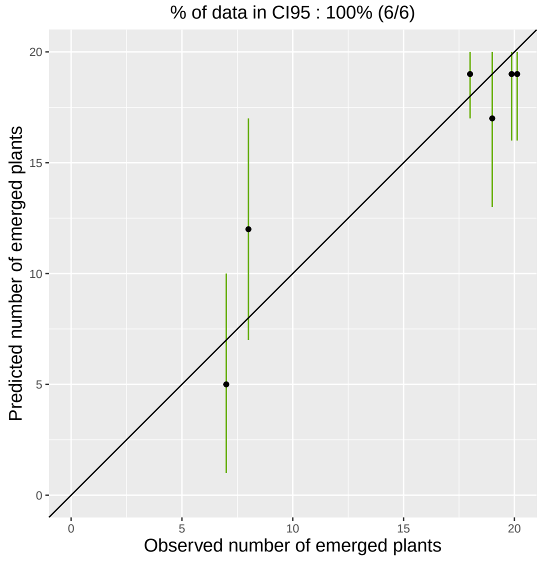

(b) Posterior predictive check (PPC)

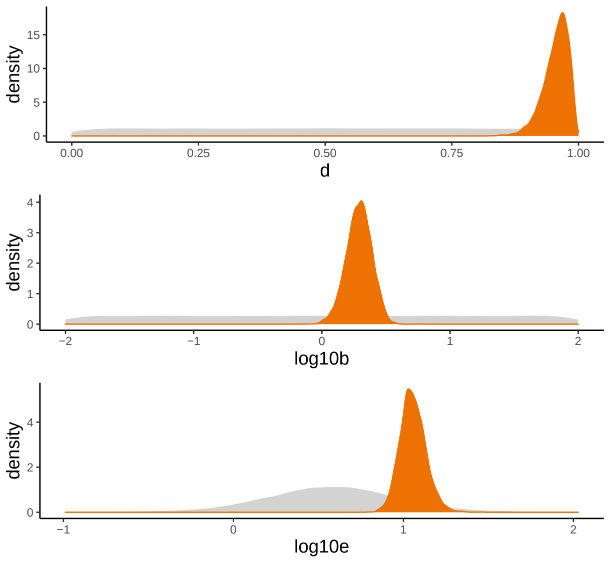

(c) Priors and posteriors

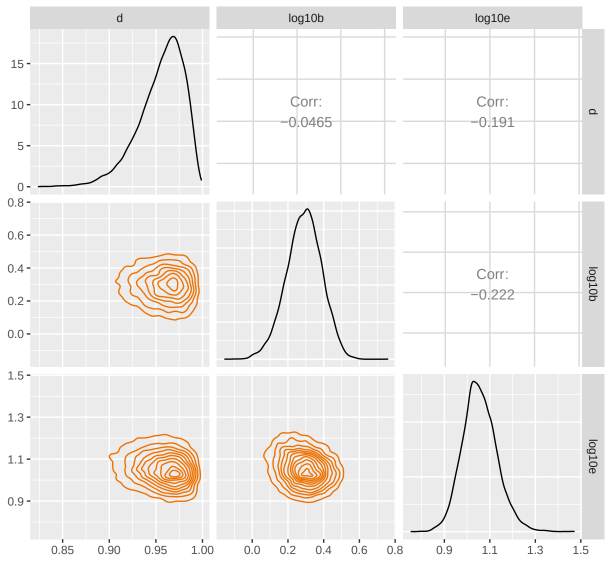

(d) Correlations between parameters

Figure 7: Dose-response curve (a), PPC (b), prior and posterior distributions (c) and correlations between parameters (d).

Data set: LOLPE_SE_emergence

Table 8: Summary of parameter estimates for LOLPE_SE_emergence data set

Parameter	median	Q2.5	Q97.5
b	1.807	0.948	3.242
d	0.886	0.795	0.954
e	1.002	0.680	1.601

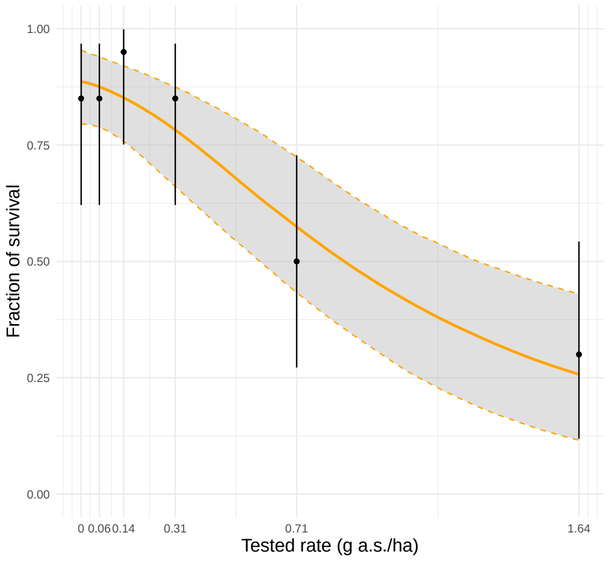

(a) Dose-response curve

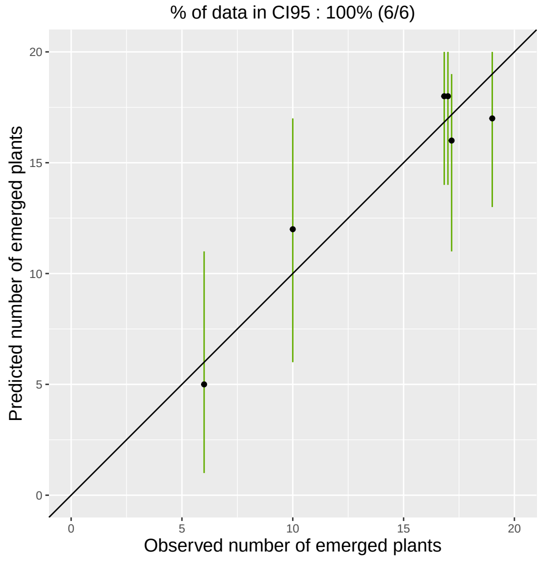

(b) Posterior predictive check (PPC)

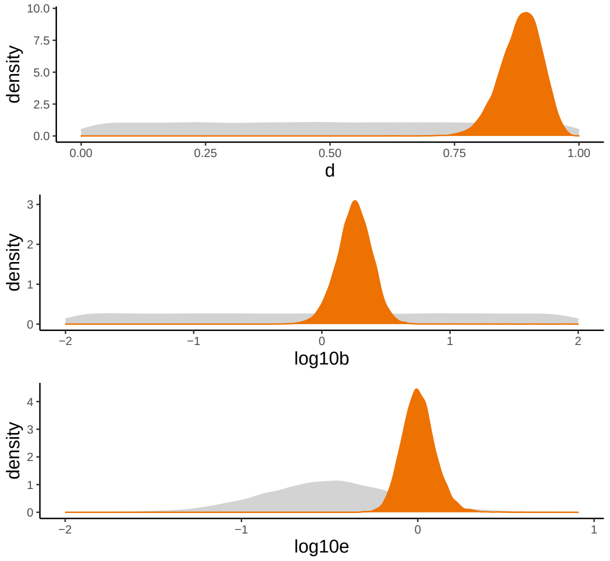

(c) Priors and posteriors

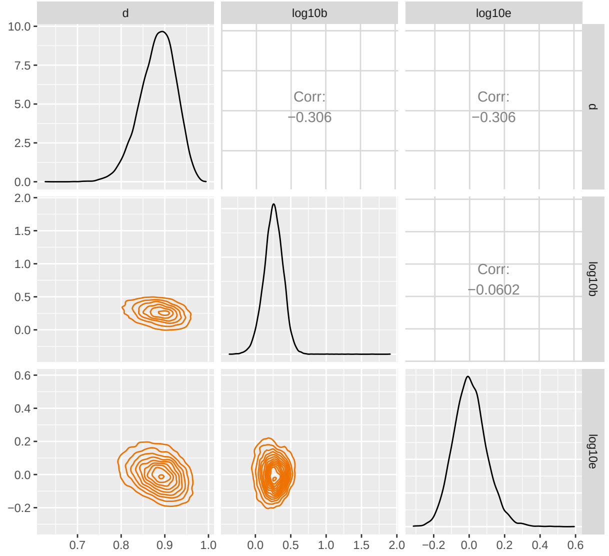

(d) Correlations between parameters

Figure 8: Dose-response curve (a), PPC (b), prior and posterior distributions (c) and correlations between parameters (d).

Data set: LYPES_SE_emergence

Table 9: Summary of parameter estimates for LYPES_SE_emergence data set

Parameter	median	Q2.5	Q97.5
b	3.340	2.166	5.625
d	0.958	0.875	0.994
e	0.565	0.443	0.719

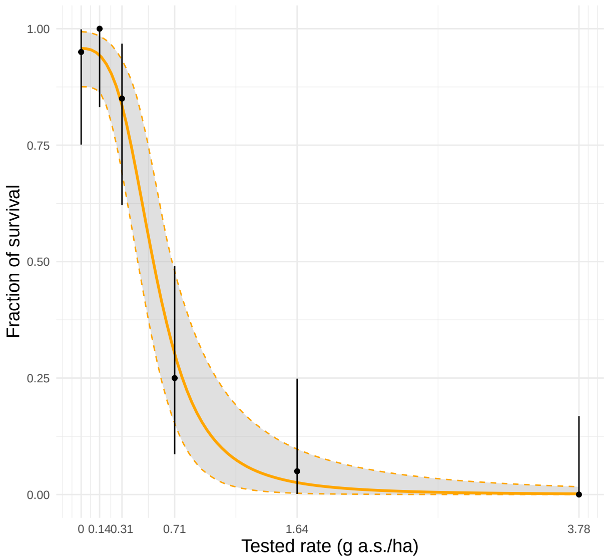

(a) Dose-response curve

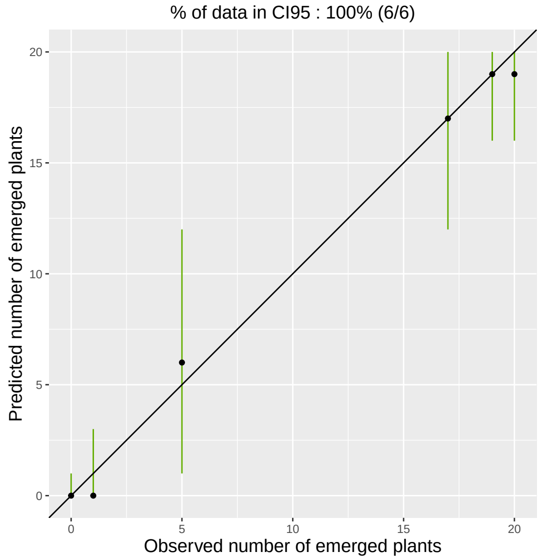

(b) Posterior predictive check (PPC)

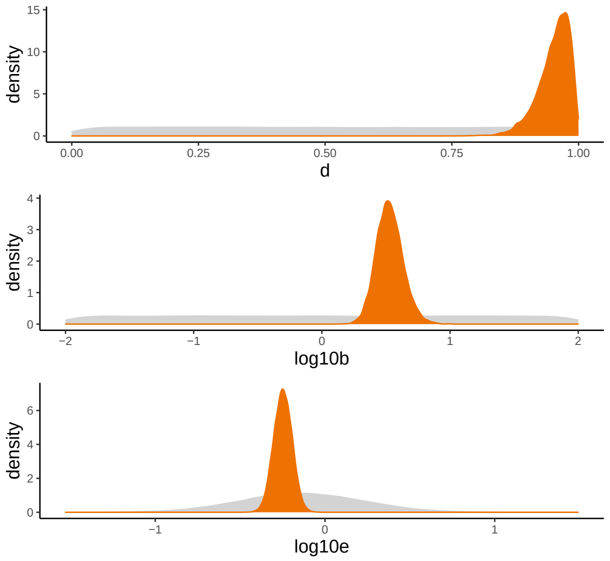

(c) Priors and posteriors

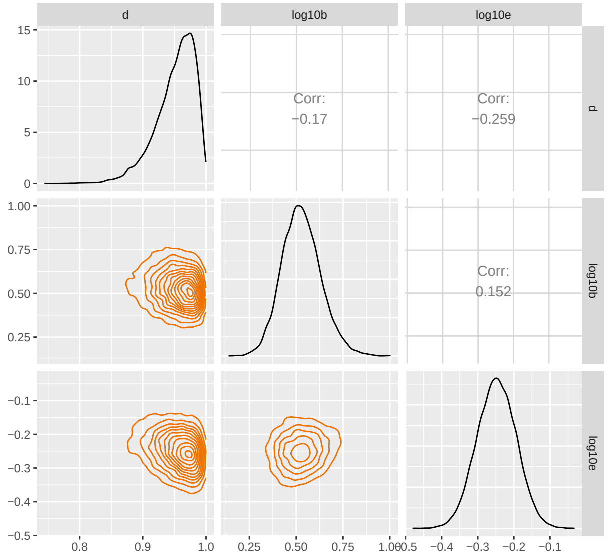

(d) Correlations between parameters

Figure 9: Dose-response curve (a), PPC (b), prior and posterior distributions (c) and correlations between parameters (d).

Data set: ZEAMA_SE_emergence

Table 10: Summary of parameter estimates (parameter d is set to 1) for ZEAMA_SE_emergence data set

Parameter	median	Q2.5	Q97.5
b	1.663	0.986	2.825
e	56.881	31.018	137.113

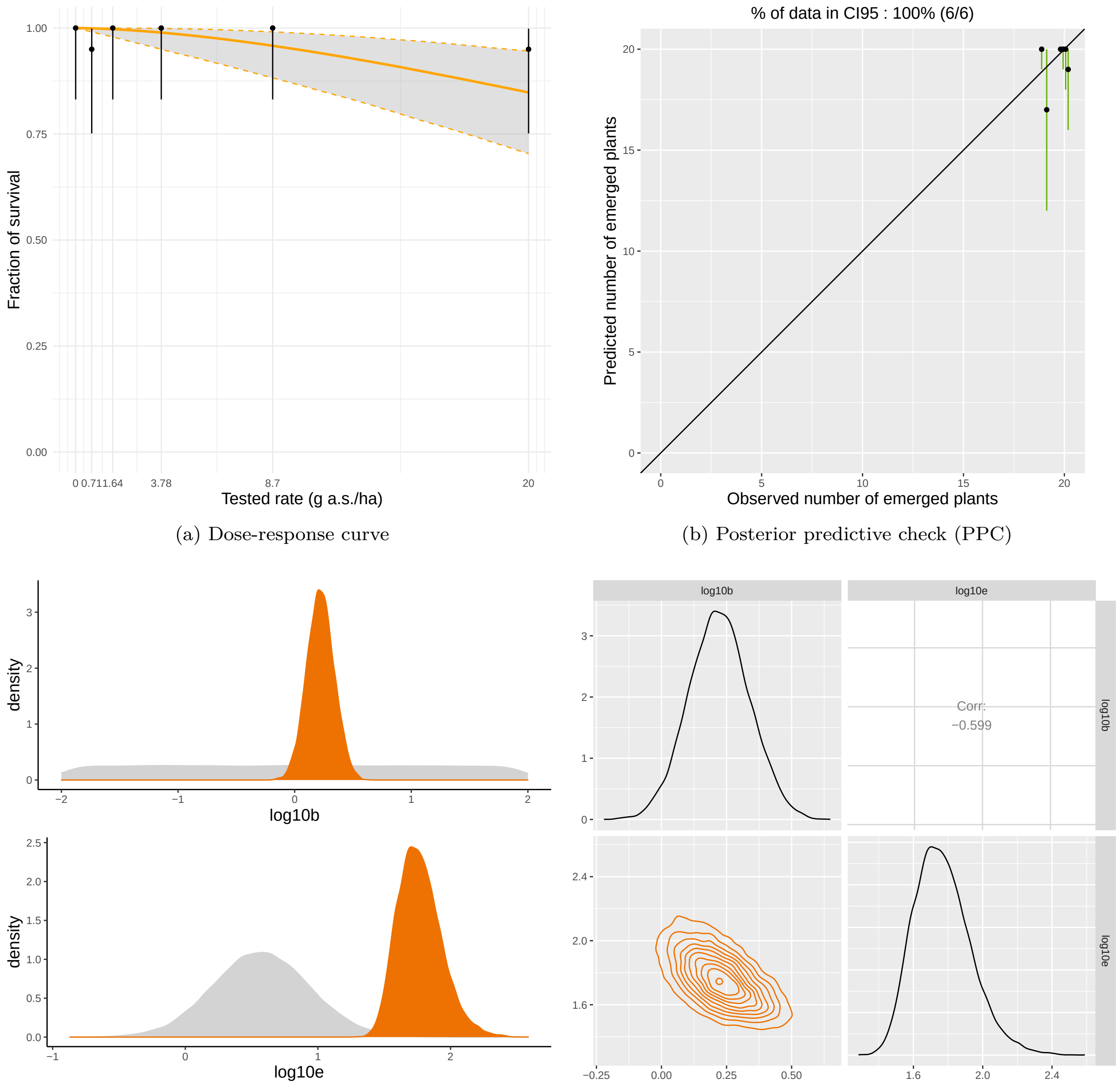

(a) Dose-response curve

(b) Posterior predictive check (PPC)

(c) Priors and posteriors

(d) Correlations between parameters

Figure 10: Dose-response curve (a), PPC (b), prior and posterior distributions (c) and correlations between parameters (d).
