# Supplementary material for: How to account for the uncertainty from standard toxicity tests in species sensitivity distributions: An example in non-target plants
Source: PLoS One. 2021 Jan 7;16(1):e0245071. doi: 10.1371/journal.pone.0245071 (PMC7790375; doi:10.1371/journal.pone.0245071)
Supplement: S1 Archive — It is a zip file containing seven folders (one folder per case study). Each folder contains five files report_xxx.pdf with detailed results of the dose-response analyses, one file corresponding to does-response analysis per endpoint. It also contains one file ER50_censoring.pdf for censored ER50 and one file SSD_analyses.pdf for results of SSD analyses. (ZIP) [file pone.0245071.s004.zip › S1_archive/Study5/report_SE_survival.pdf]

Dose-response analyses

Study 5

Seedling Emergence test - survival endpoint

25 June 2020

Contact: sandrine.charles@univ-lyon1.fr

This is a report which provides results on all performed dose-response analyses for the survival endpoint of the Seedling Emergence test for study 5.

Contents

Data set: ALLCE_SE_survival 2
Data set: AVESA_SE_survival 3
Data set: BEAVA_SE_survival 4
Data set: BRSNW_SE_survival 5
Data set: CUMSA_SE_survival 6
Data set: GLXMA_SE_survival 7
Data set: HELAN_SE_survival 8
Data set: LOLPE_SE_survival 9
Data set: LYPES_SE_survival 10
Data set: ZEAMA_SE_survival 11

Data set: ALLCE_SE_survival

Table 1: Summary of parameter estimates (parameter d is set to 1) for ALLCE_SE_survival data set

Parameter	median	Q2.5	Q97.5
b	2.715	1.484	4.537
e	1.066	0.800	1.574

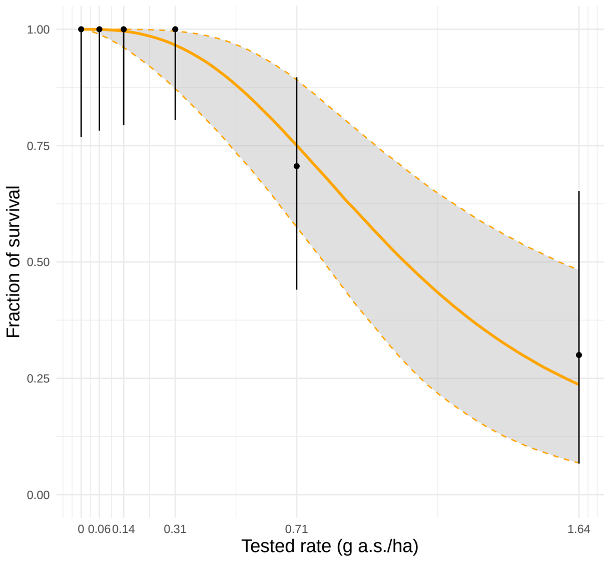

(a) Dose-response curve

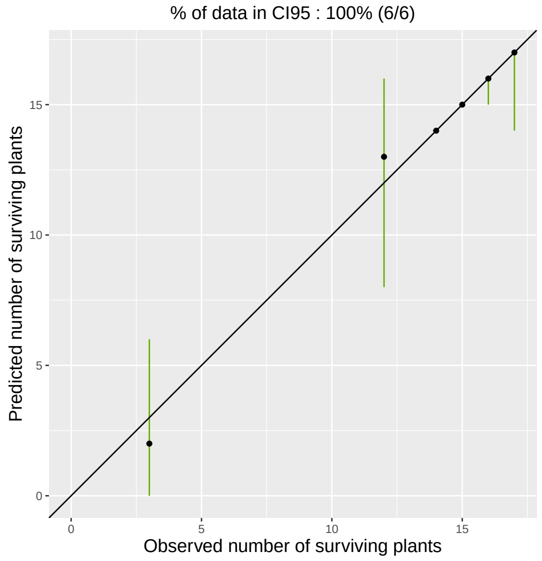

(b) Posterior predictive check (PPC)

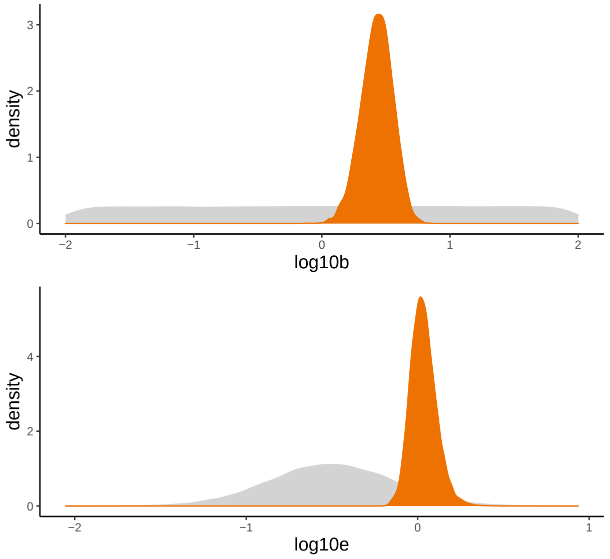

(c) Priors and posteriors

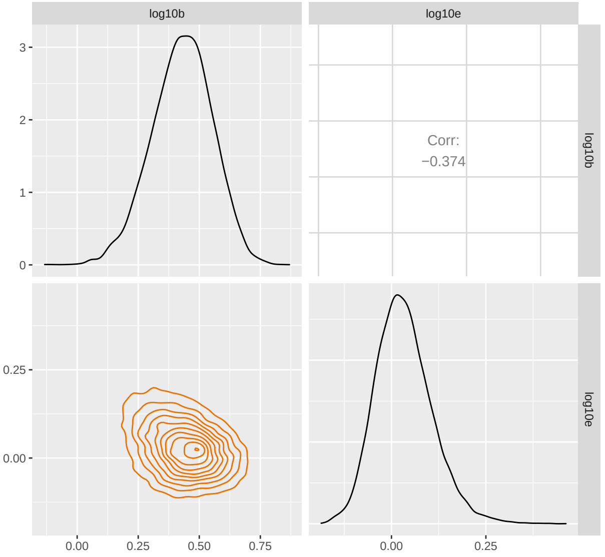

(d) Correlations between parameters

Figure 1: Dose-response curve (a), PPC (b), prior and posterior distributions (c) and correlations between parameters (d).

Data set: AVESA_SE_survival

Table 2: Summary of parameter estimates for AVESA_SE_survival data set

Parameter	median	Q2.5	Q97.5
b	2.144	1.358	3.432
d	0.962	0.885	0.994
e	3.204	2.349	4.455

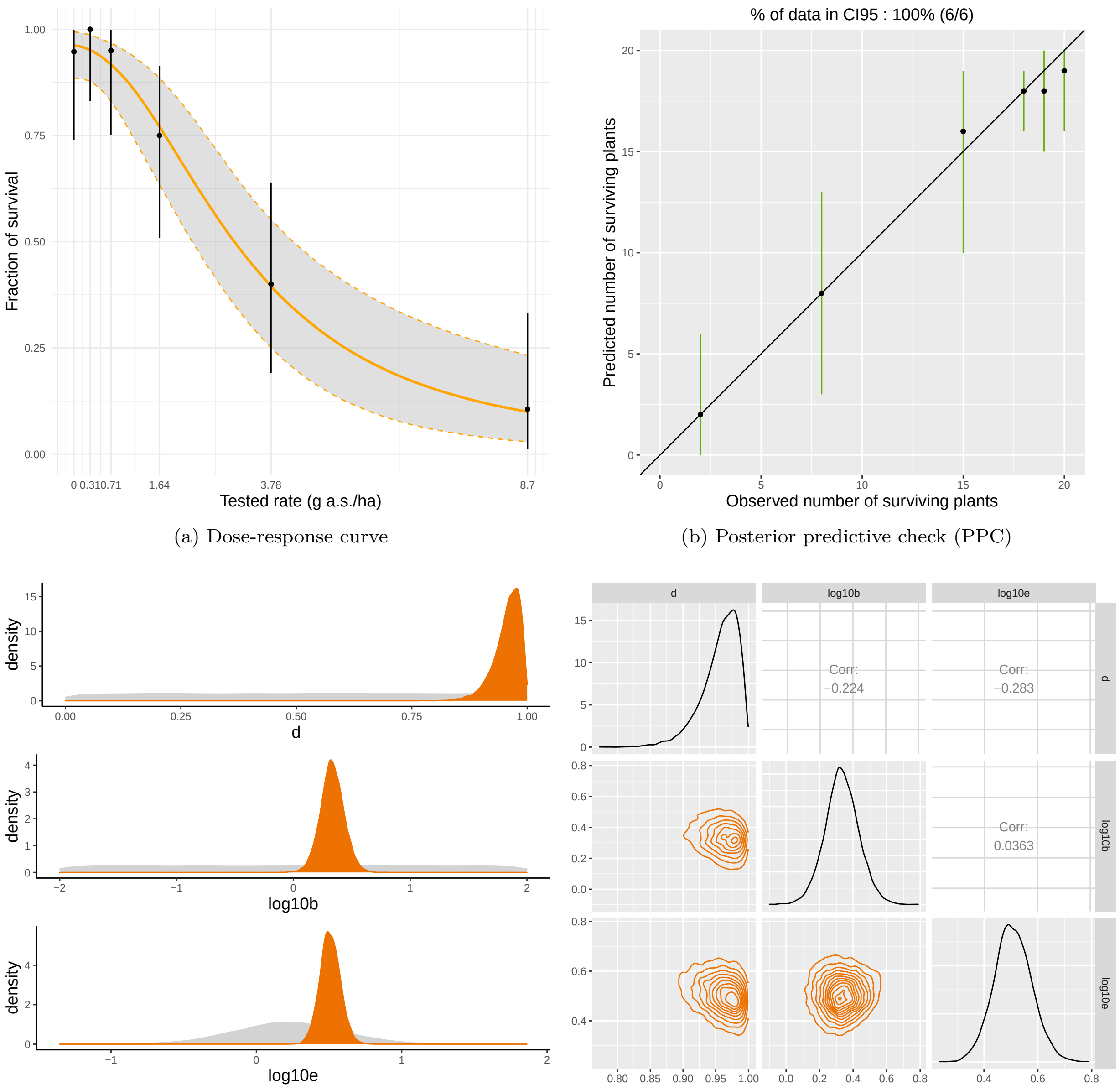

(a) Dose-response curve

(b) Posterior predictive check (PPC)

(c) Priors and posteriors

(d) Correlations between parameters

Figure 2: Dose-response curve (a), PPC (b), prior and posterior distributions (c) and correlations between parameters (d).

Data set: BEAVA_SE_survival

Table 3: Summary of parameter estimates (parameter d is set to 1) for BEAVA_SE_survival data set

Parameter	median	Q2.5	Q97.5
b	1.830	0.884	3.361
e	2.204	1.308	4.737

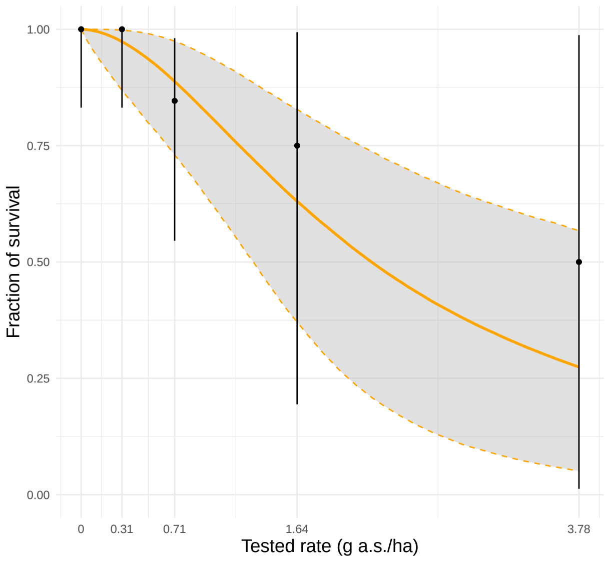

(a) Dose-response curve

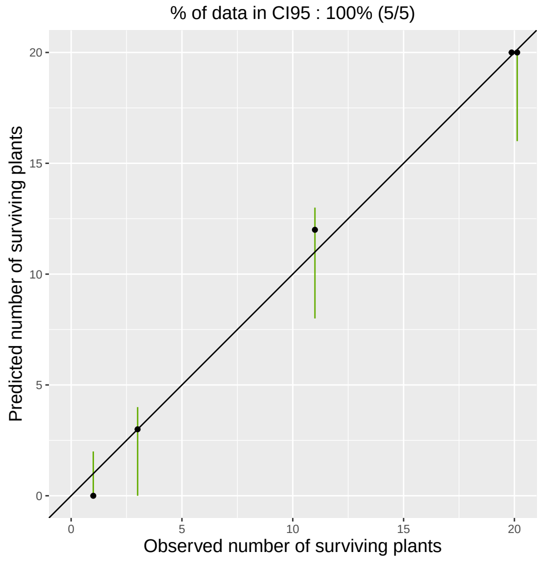

(b) Posterior predictive check (PPC)

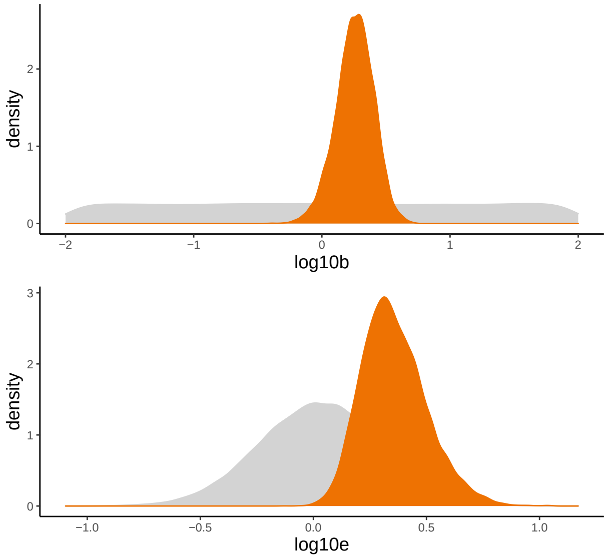

(c) Priors and posteriors

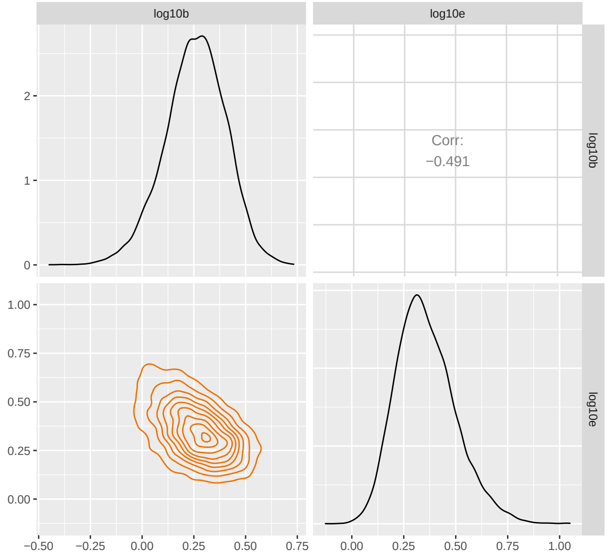

(d) Correlations between parameters

Figure 3: Dose-response curve (a), PPC (b), prior and posterior distributions (c) and correlations between parameters (d).

Data set: BRSNW_SE_survival

Table 4: Summary of parameter estimates (parameter d is set to 1) for BRSNW_SE_survival data set

Parameter	median	Q2.5	Q97.5
b	21.022	2.757	92.735
e	2.162	1.551	4.903

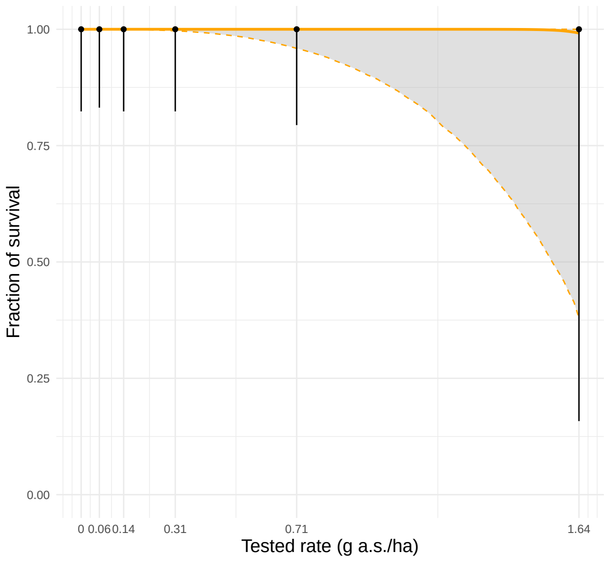

(a) Dose-response curve

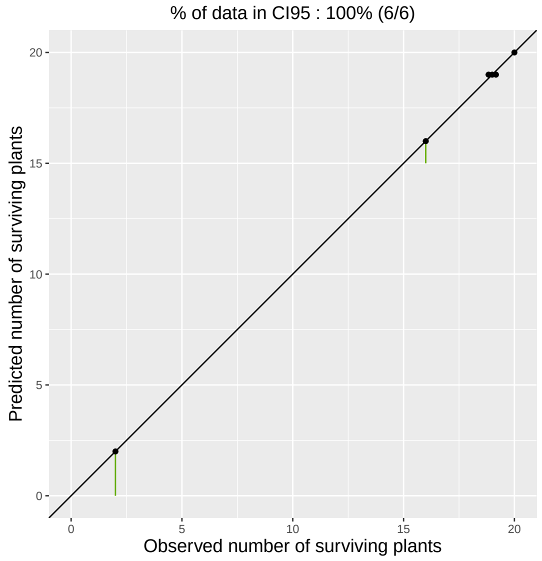

(b) Posterior predictive check (PPC)

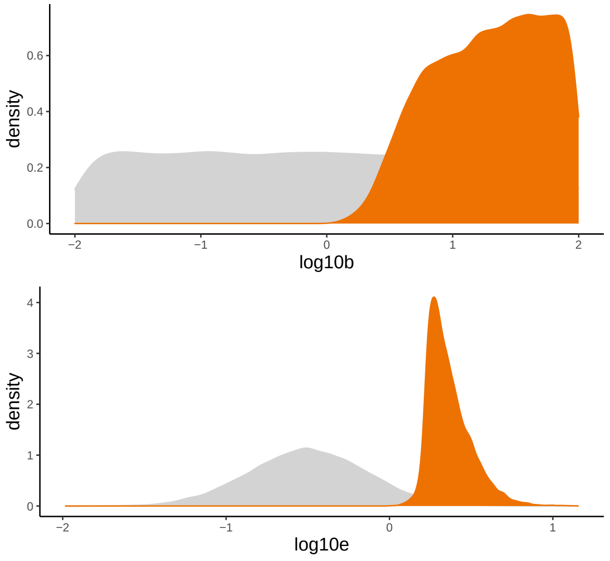

(c) Priors and posteriors

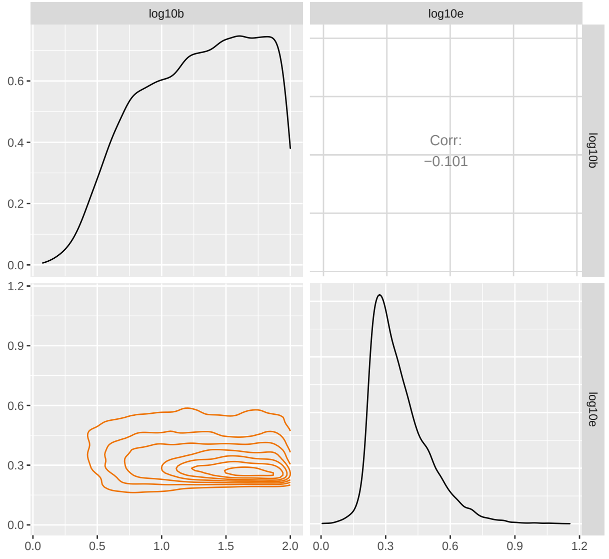

(d) Correlations between parameters

Figure 4: Dose-response curve (a), PPC (b), prior and posterior distributions (c) and correlations between parameters (d).

Data set: CUMSA_SE_survival

Table 5: Summary of parameter estimates (parameter d is set to 1) for CUMSA_SE_survival data set

Parameter	median	Q2.5	Q97.5
b	4.276	2.630	6.807
e	2.364	1.899	2.974

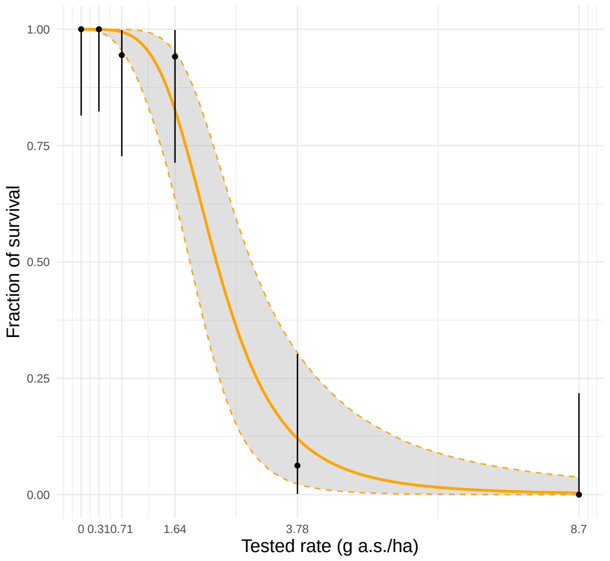

(a) Dose-response curve

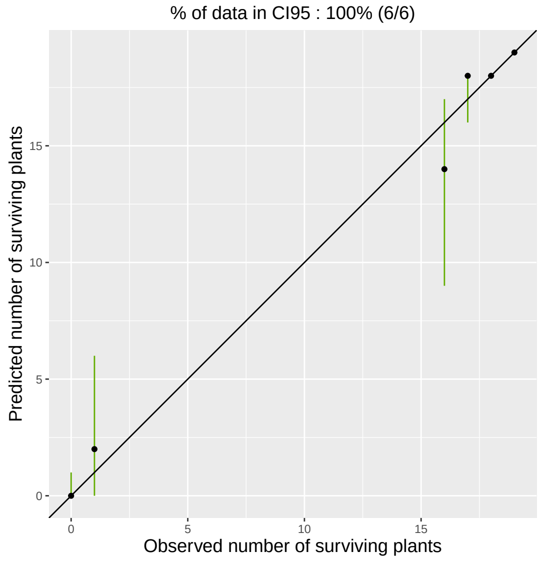

(b) Posterior predictive check (PPC)

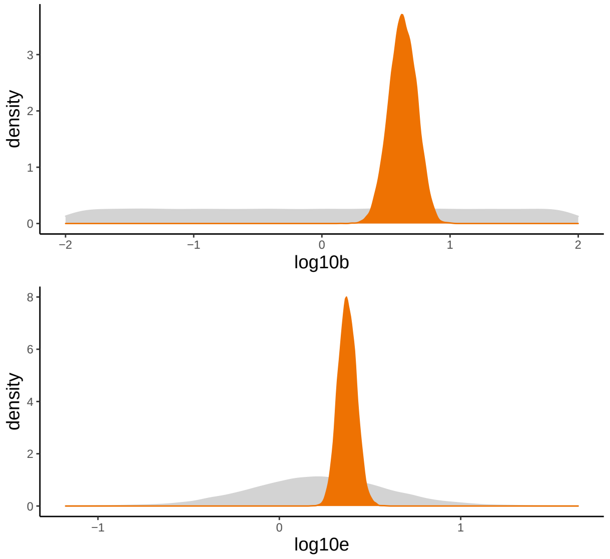

(c) Priors and posteriors

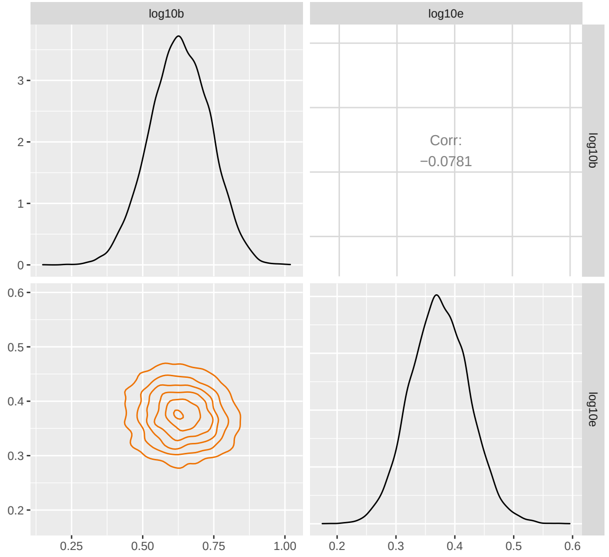

(d) Correlations between parameters

Figure 5: Dose-response curve (a), PPC (b), prior and posterior distributions (c) and correlations between parameters (d).

Data set: GLXMA_SE_survival

Table 6: Summary of parameter estimates (parameter d is set to 1) for GLXMA_SE_survival data set

Parameter	median	Q2.5	Q97.5
b	1.827	1.049	2.973
e	4.863	3.339	8.564

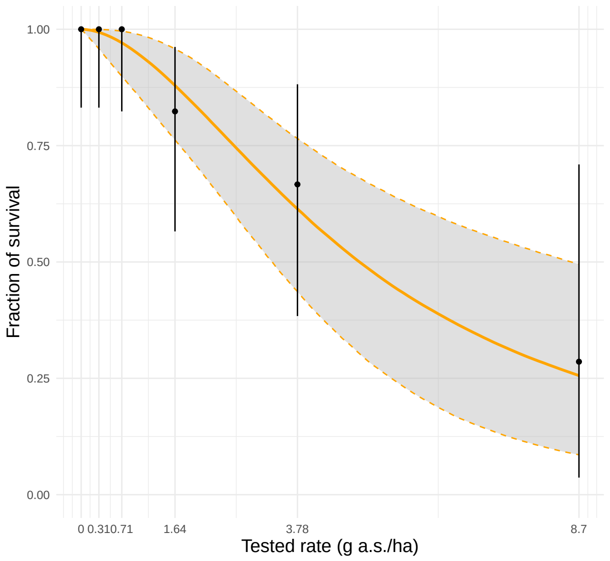

(a) Dose-response curve

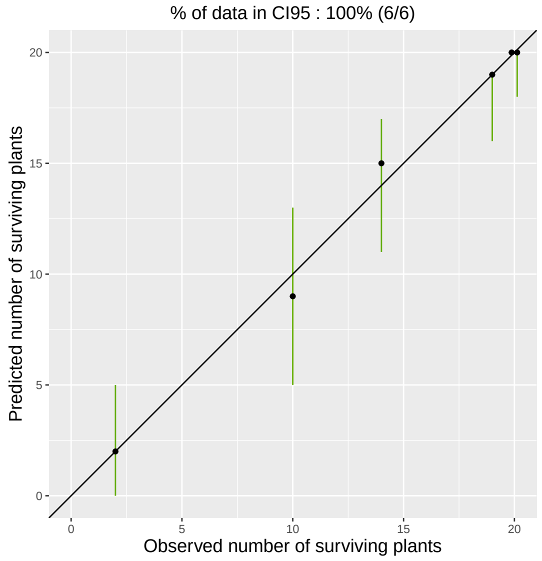

(b) Posterior predictive check (PPC)

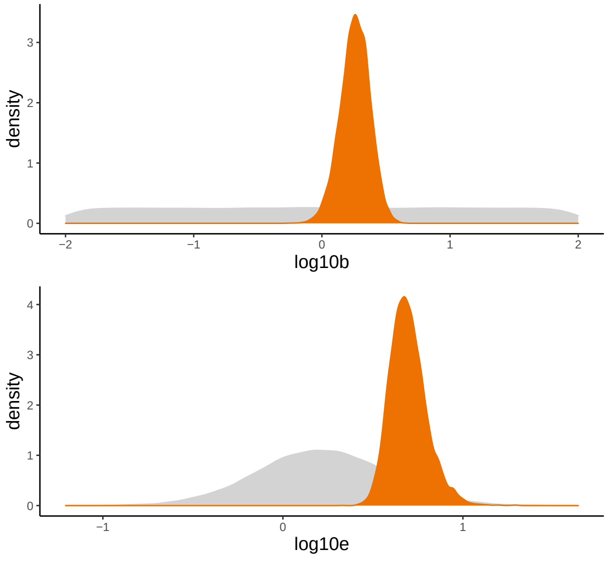

(c) Priors and posteriors

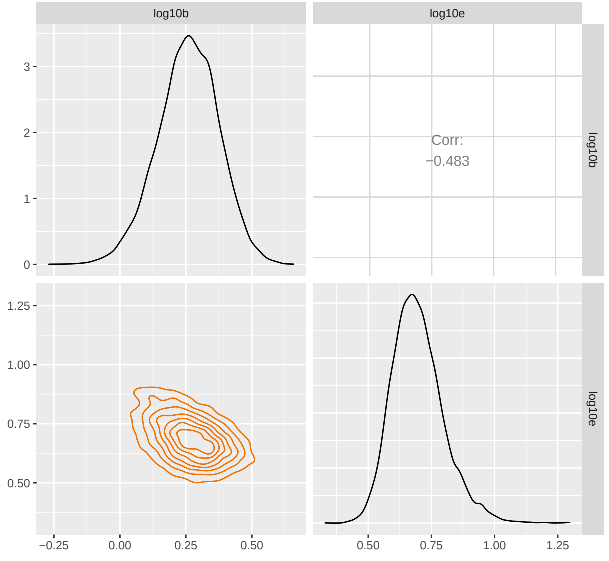

(d) Correlations between parameters

Figure 6: Dose-response curve (a), PPC (b), prior and posterior distributions (c) and correlations between parameters (d).

Data set: HELAN_SE_survival

Table 7: Summary of parameter estimates (parameter d is set to 1) for HELAN_SE_survival data set

Parameter	median	Q2.5	Q97.5
b	1.630	0.925	2.817
e	24.073	14.046	56.197

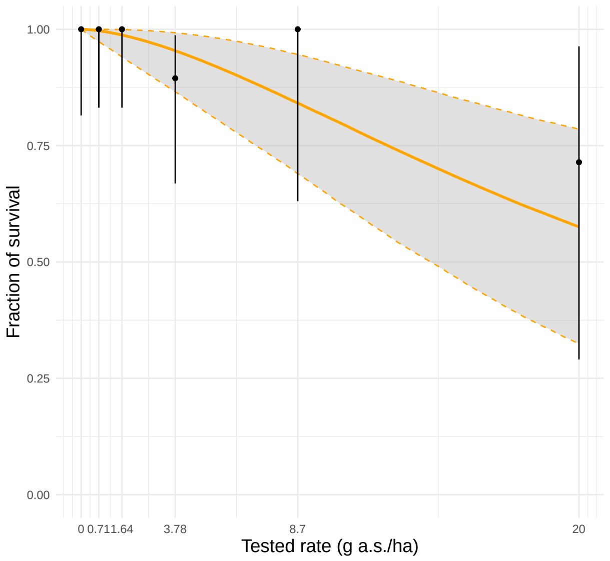

(a) Dose-response curve

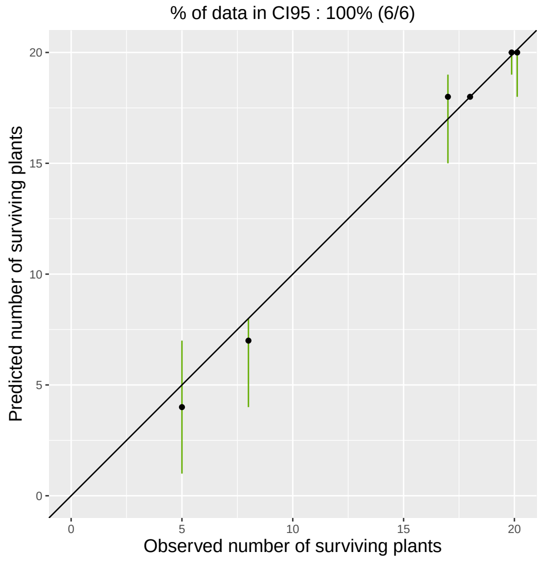

(b) Posterior predictive check (PPC)

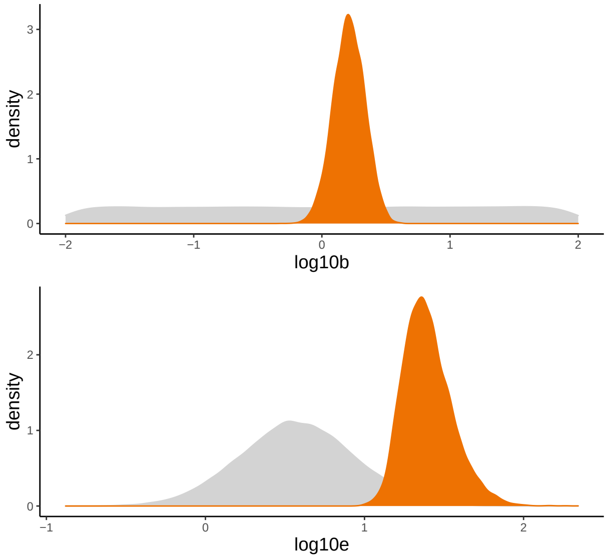

(c) Priors and posteriors

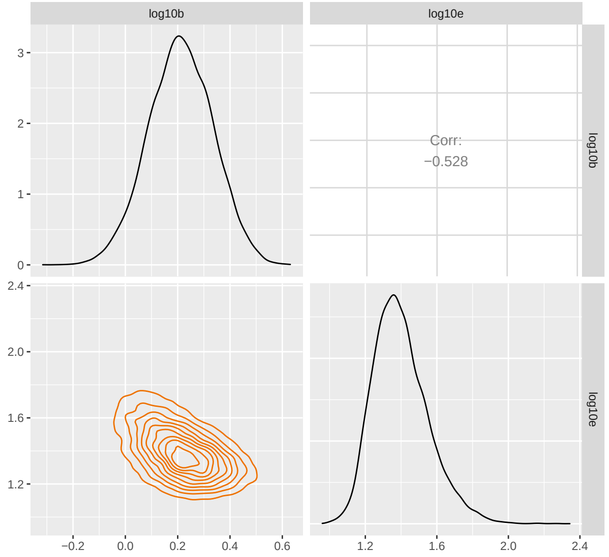

(d) Correlations between parameters

Figure 7: Dose-response curve (a), PPC (b), prior and posterior distributions (c) and correlations between parameters (d).

Data set: LOLPE_SE_survival

Table 8: Summary of parameter estimates (parameter d is set to 1) for LOLPE_SE_survival data set

Parameter	median	Q2.5	Q97.5
b	1.987	1.159	3.108
e	0.304	0.221	0.444

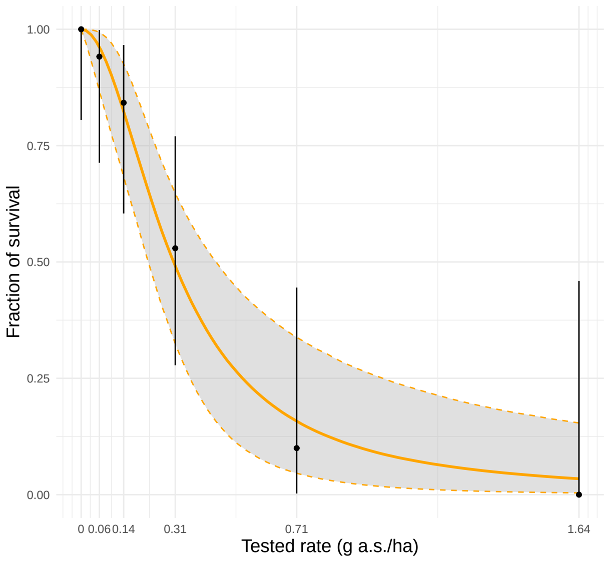

(a) Dose-response curve

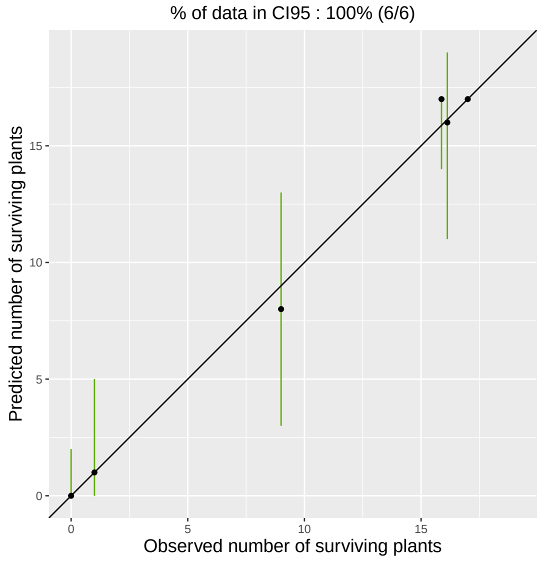

(b) Posterior predictive check (PPC)

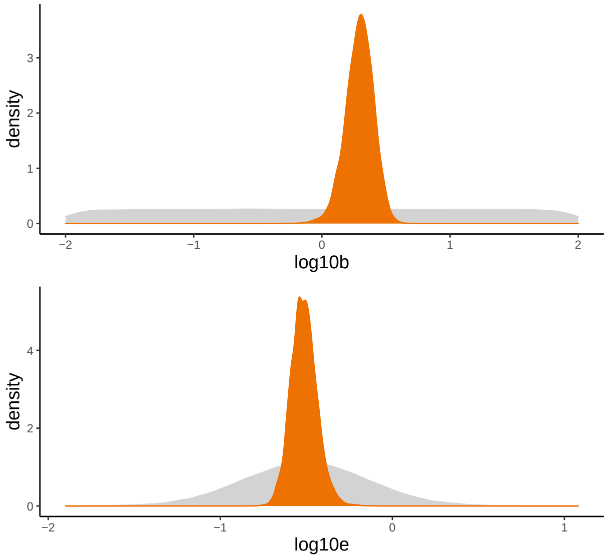

(c) Priors and posteriors

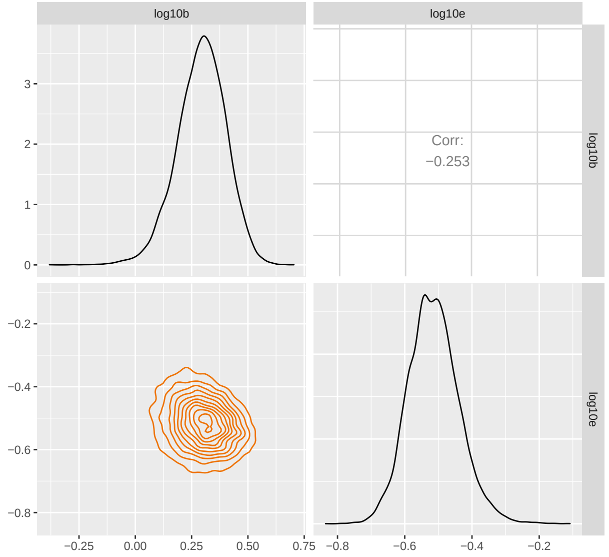

(d) Correlations between parameters

Figure 8: Dose-response curve (a), PPC (b), prior and posterior distributions (c) and correlations between parameters (d).

Data set: LYPES_SE_survival

Table 9: Summary of parameter estimates (parameter d is set to 1) for LYPES_SE_survival data set

Parameter	median	Q2.5	Q97.5
b	1.878	0.991	3.448
e	1.542	0.850	3.444

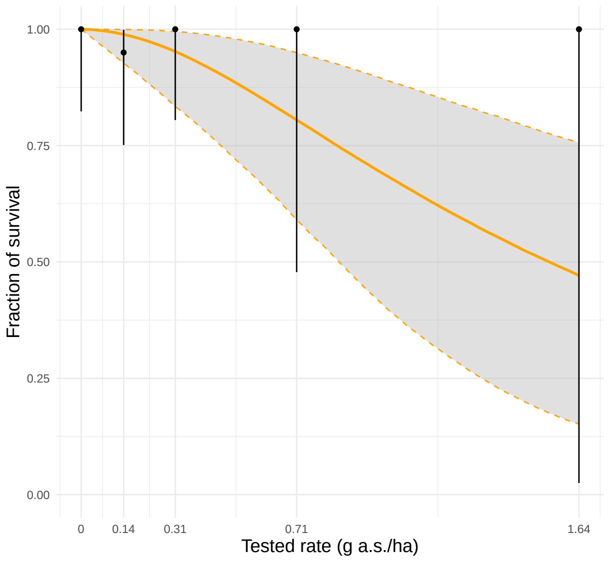

(a) Dose-response curve

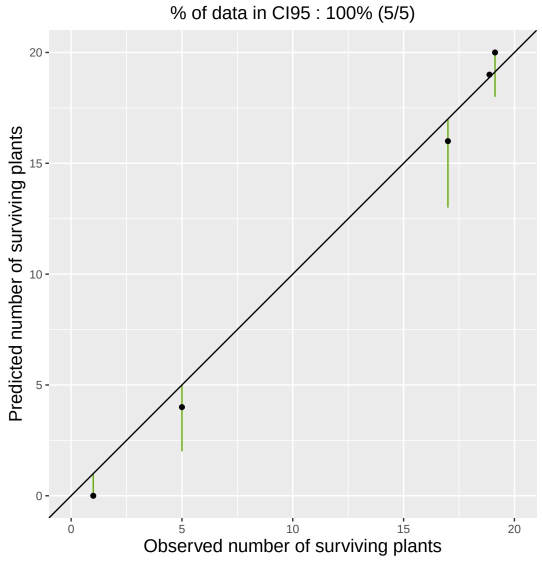

(b) Posterior predictive check (PPC)

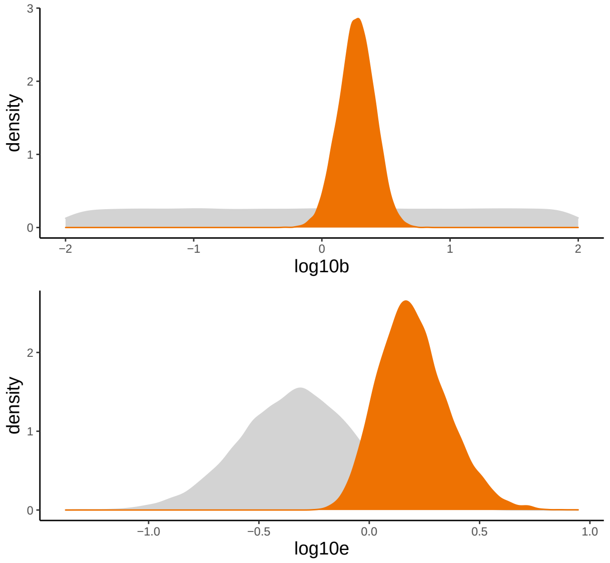

(c) Priors and posteriors

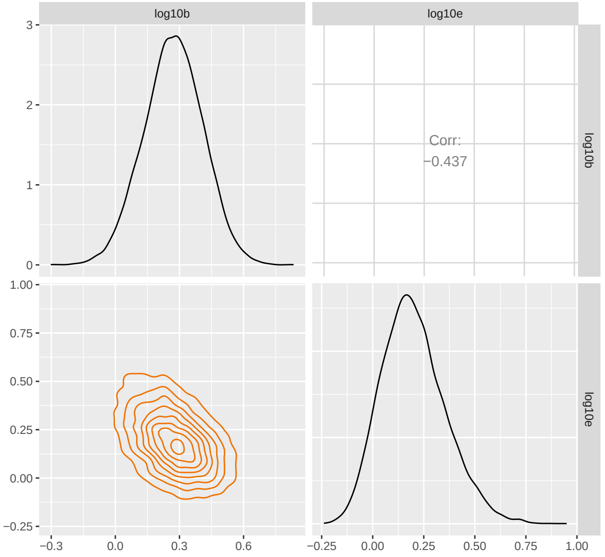

(d) Correlations between parameters

Figure 9: Dose-response curve (a), PPC (b), prior and posterior distributions (c) and correlations between parameters (d).

Data set: ZEAMA_SE_survival

Table 10: Summary of parameter estimates (parameter d is set to 1) for ZEAMA_SE_survival data set

Parameter	median	Q2.5	Q97.5
b	33.631	4.836	95.653
e	29.121	21.419	67.562

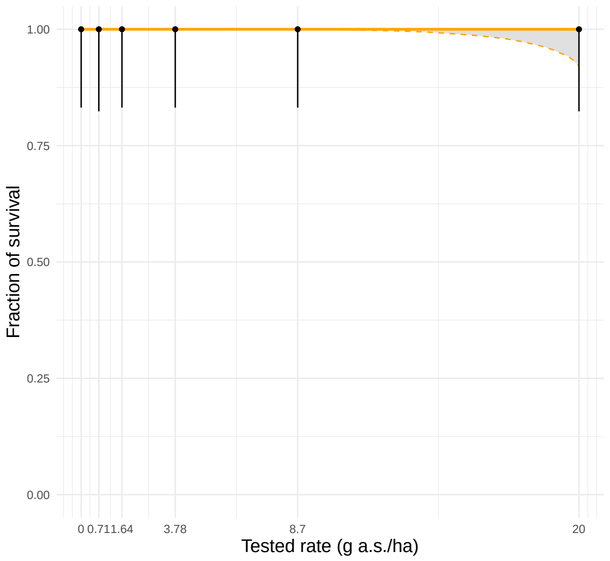

(a) Dose-response curve

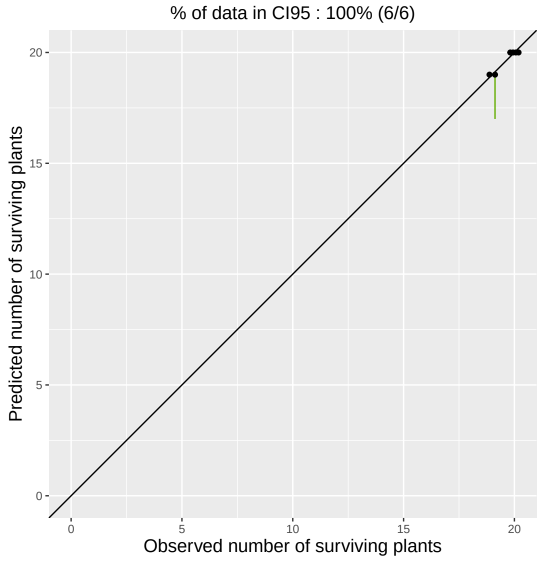

(b) Posterior predictive check (PPC)

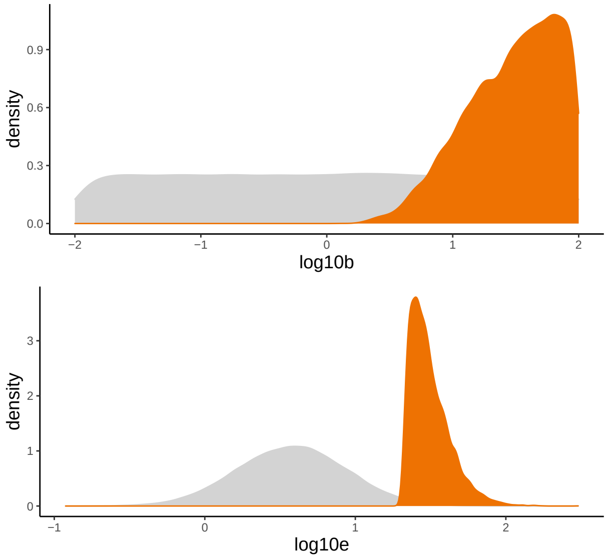

(c) Priors and posteriors

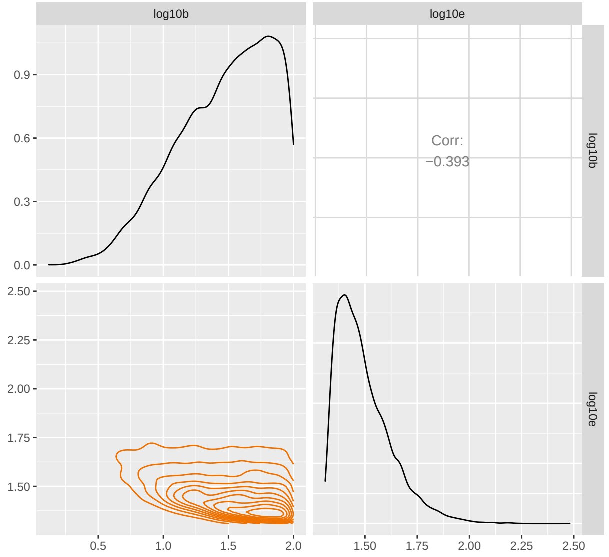

(d) Correlations between parameters

Figure 10: Dose-response curve (a), PPC (b), prior and posterior distributions (c) and correlations between parameters (d).
