# Supplementary material for: How to account for the uncertainty from standard toxicity tests in species sensitivity distributions: An example in non-target plants
Source: PLoS One. 2021 Jan 7;16(1):e0245071. doi: 10.1371/journal.pone.0245071 (PMC7790375; doi:10.1371/journal.pone.0245071)
Supplement: S1 Archive — It is a zip file containing seven folders (one folder per case study). Each folder contains five files report_xxx.pdf with detailed results of the dose-response analyses, one file corresponding to does-response analysis per endpoint. It also contains one file ER50_censoring.pdf for censored ER50 and one file SSD_analyses.pdf for results of SSD analyses. (ZIP) [file pone.0245071.s004.zip › S1_archive/Study5/report_SE_weight.pdf]

Dose-response analysis

Study 5

Seedling Emergence test - shoot dry SE_weight endpoint

25 June 2020

Contact: sandrine.charles@univ-lyon1.fr

This is a report which provides results on all performed dose-response analyses for the shoot dry SE_weight endpoint of the Seedling Emergence test for study 5.

Contents

Data set: ALLCE_SE_weight . . . 2
Data set: AVESA_SE_weight . . . 3
Data set: BEAVA_SE_weight . . . 4
Data set: BRSNW_SE_weight . . . 5
Data set: CUMSA_SE_weight . . . 6
Data set: GLXMA_SE_weight . . . 7
Data set: HELAN_SE_weight . . . 8
Data set: LOLPE_SE_weight . . . 9
Data set: LYPES_SE_weight . . . 10
Data set: ZEAMA_SE_weight . . . 11

Data set: ALLCE_SE_weight

Table 1: Summary of parameter estimates for ALLCE_SE_weight data set

Parameter	median	Q2.5	Q97.5
b	1.068	0.484	2.297
d	0.042	0.035	0.049
e	0.648	0.352	1.217
sigma	0.009	0.007	0.012

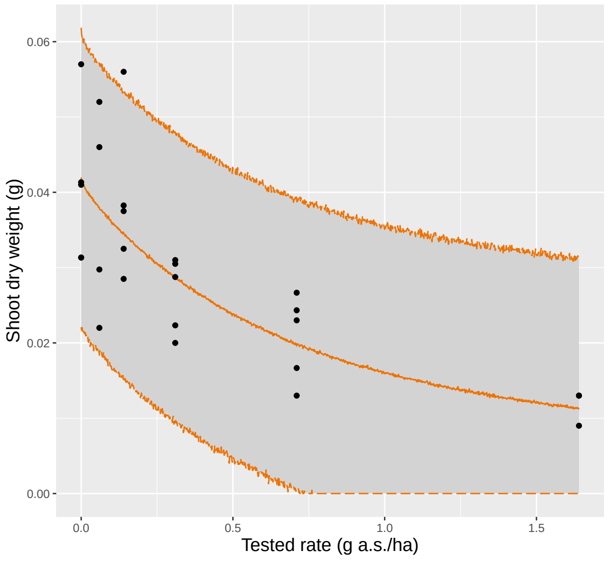

(a) Dose-response curve

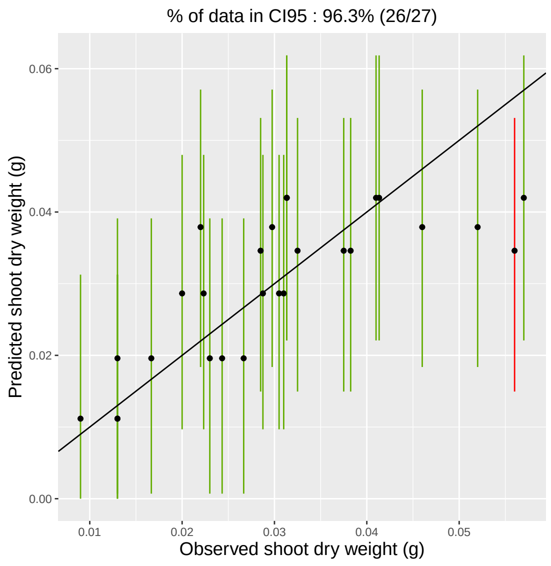

(b) Posterior predictive check (PPC)

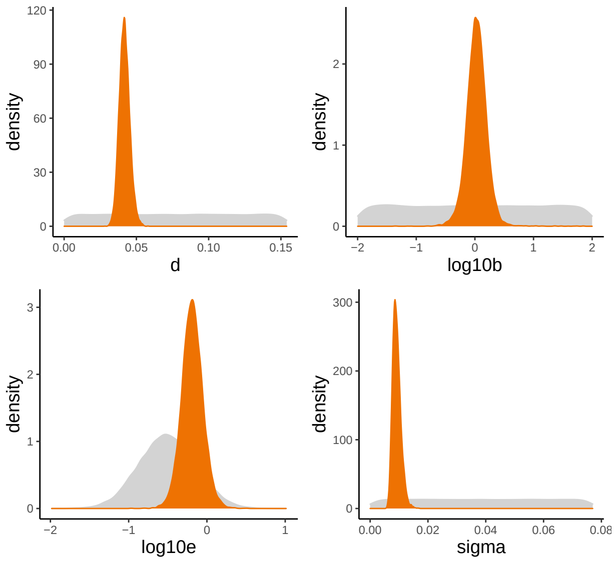

(c) Priors and posteriors

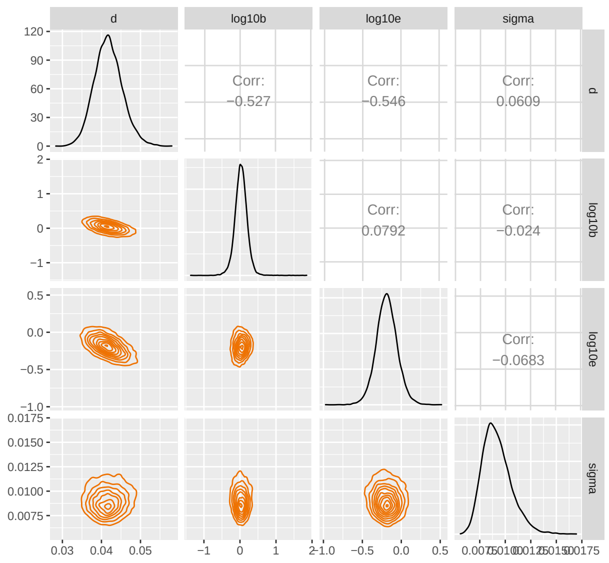

(d) Correlations between parameters

Figure 1: Dose-response curve (a), PPC (b), prior and posterior distributions (c) and correlations between parameters (d).

Data set: AVESA_SE_weight

Table 2: Summary of parameter estimates for AVESA_SE_weight data set

Parameter	median	Q2.5	Q97.5
b	0.358	0.077	0.771
d	0.876	0.740	1.017
e	4.728	1.521	13.596
sigma	0.133	0.100	0.187

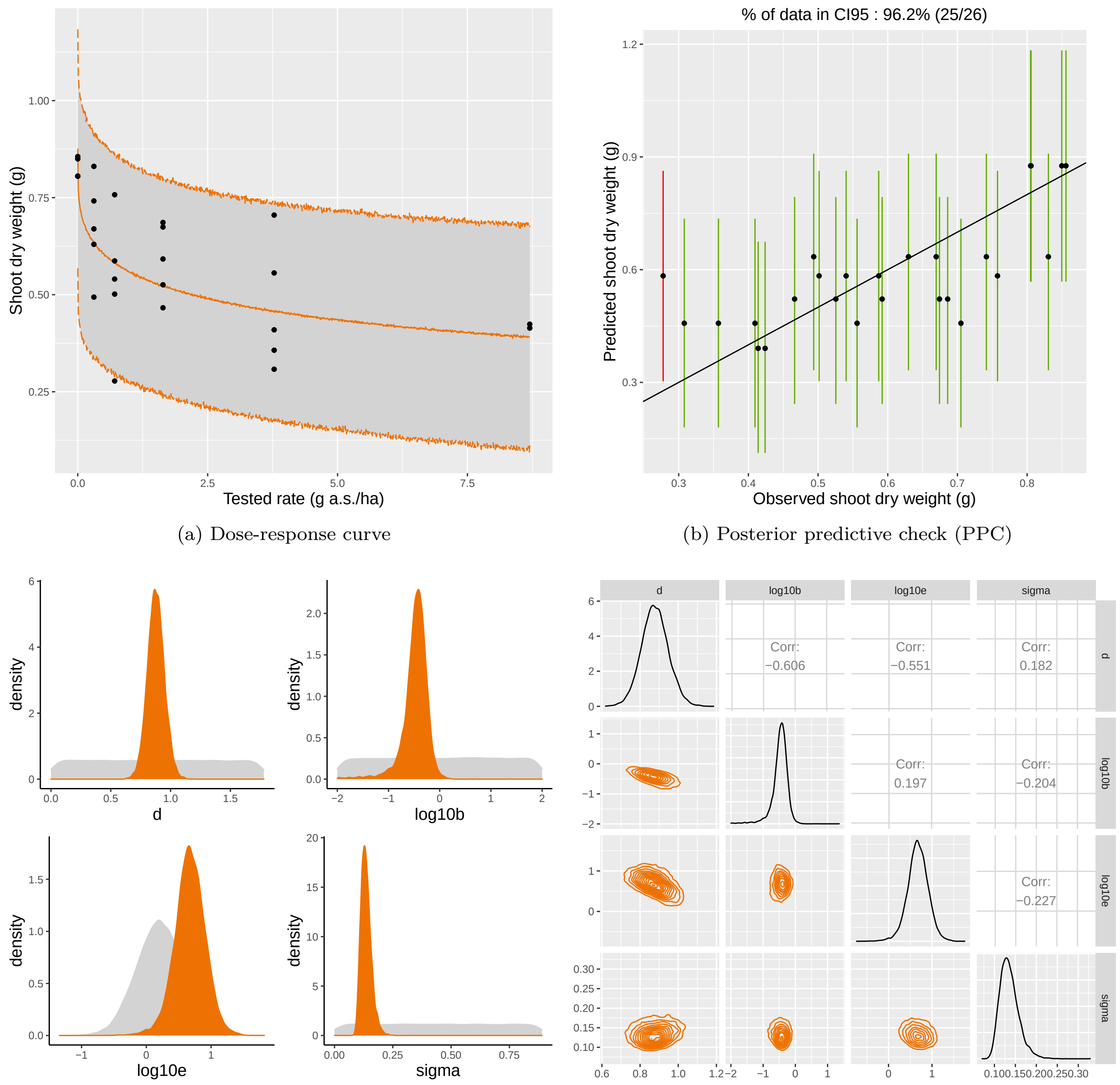

(a) Dose-response curve

(b) Posterior predictive check (PPC)

(c) Priors and posteriors

(d) Correlations between parameters

Figure 2: Dose-response curve (a), PPC (b), prior and posterior distributions (c) and correlations between parameters (d).

Data set: BEAVA_SE_weight

Table 3: Summary of parameter estimates for BEAVA_SE_weight data set

Parameter	median	Q2.5	Q97.5
b	6.949	1.175	82.756
d	1.911	1.661	2.206
e	1.902	1.330	3.539
sigma	0.630	0.498	0.846

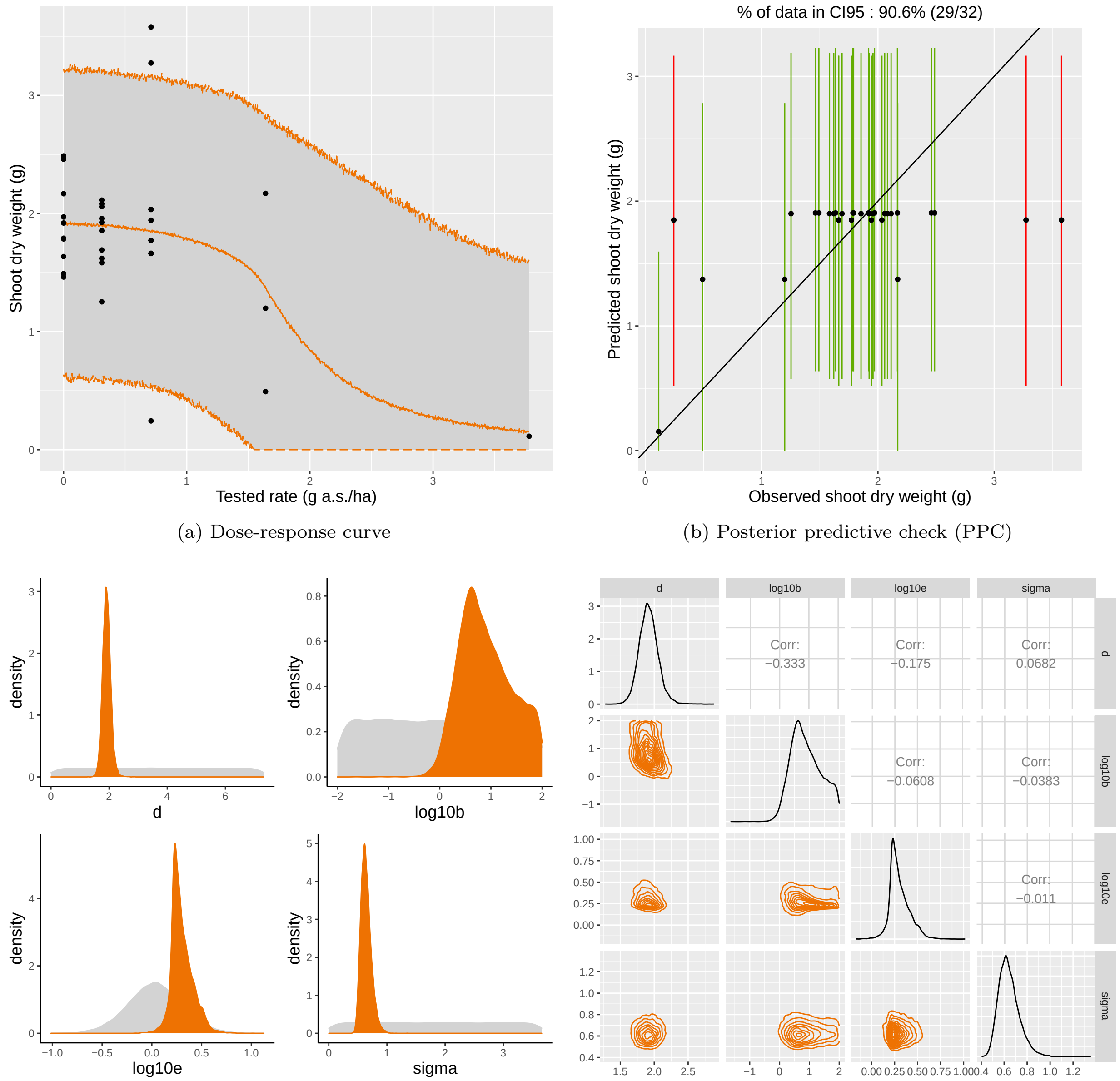

(a) Dose-response curve

(b) Posterior predictive check (PPC)

(c) Priors and posteriors

(d) Correlations between parameters

Figure 3: Dose-response curve (a), PPC (b), prior and posterior distributions (c) and correlations between parameters (d).

Data set: BRSNW_SE_weight

Table 4: Summary of parameter estimates for BRSNW_SE_weight data set

Parameter	median	Q2.5	Q97.5
b	2.821	1.431	10.526
d	3.095	2.969	3.239
e	1.358	0.995	1.974
sigma	0.348	0.285	0.439

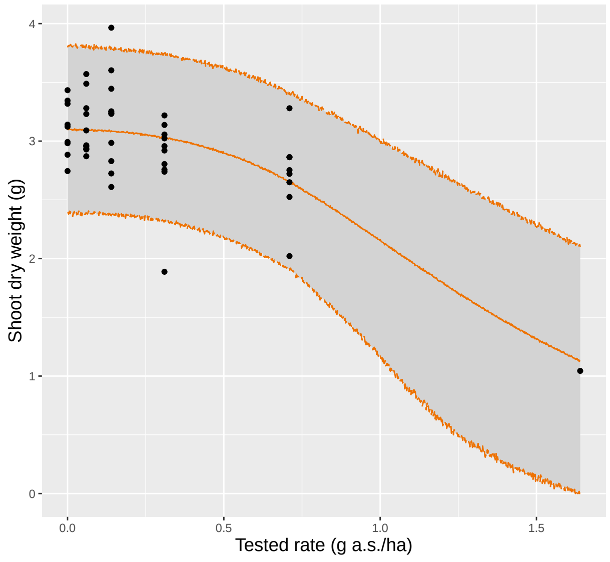

(a) Dose-response curve

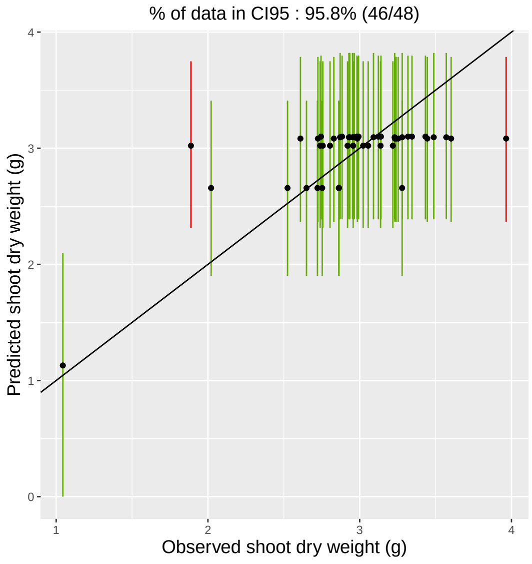

(b) Posterior predictive check (PPC)

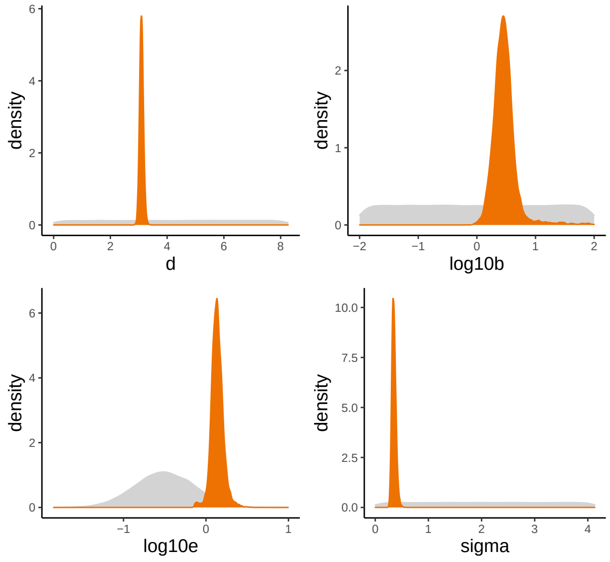

(c) Priors and posteriors

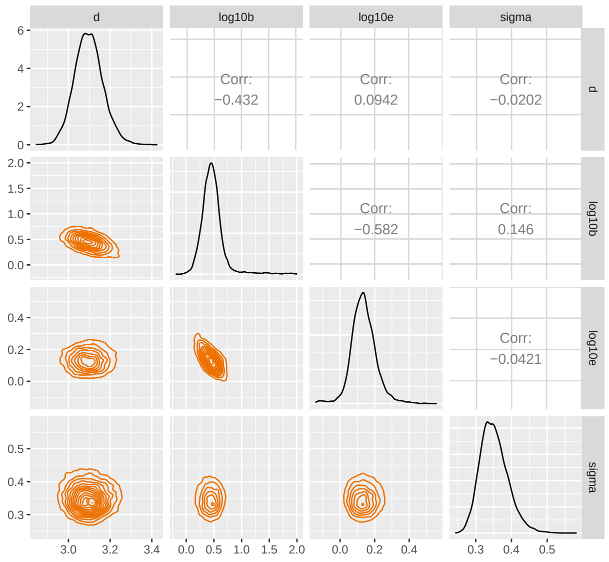

(d) Correlations between parameters

Figure 4: Dose-response curve (a), PPC (b), prior and posterior distributions (c) and correlations between parameters (d).

Data set: CUMSA_SE_weight

Table 5: Summary of parameter estimates for CUMSA_SE_weight data set

Parameter	median	Q2.5	Q97.5
b	5.662	0.738	85.792
d	4.654	4.005	5.643
e	3.346	1.745	7.123
sigma	1.761	1.417	2.274

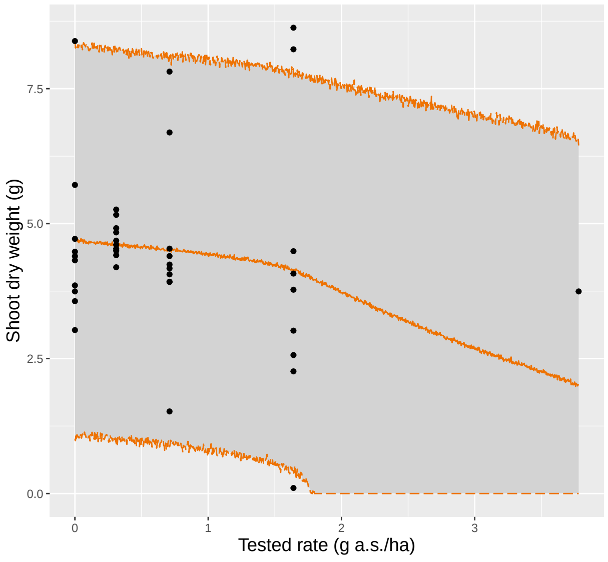

(a) Dose-response curve

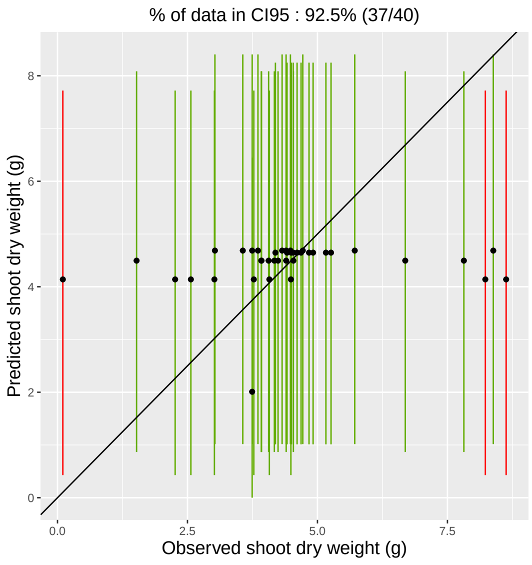

(b) Posterior predictive check (PPC)

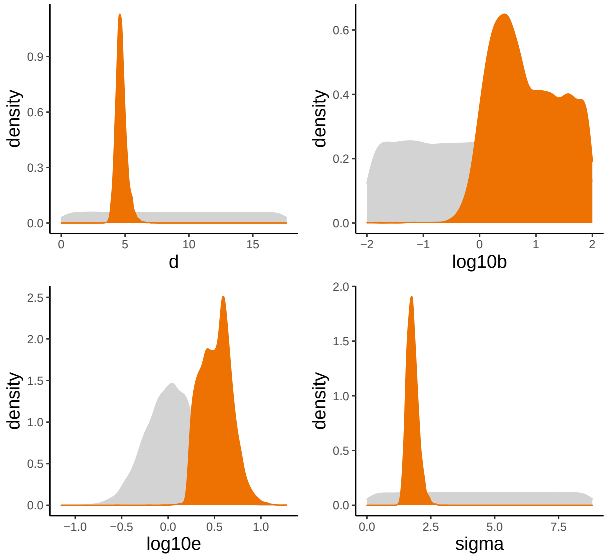

(c) Priors and posteriors

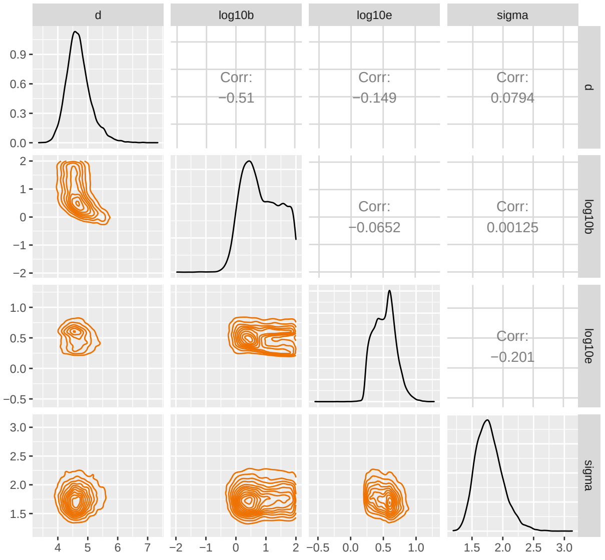

(d) Correlations between parameters

Figure 5: Dose-response curve (a), PPC (b), prior and posterior distributions (c) and correlations between parameters (d).

Data set: GLXMA_SE_weight

Table 6: Summary of parameter estimates for GLXMA_SE_weight data set

Parameter	median	Q2.5	Q97.5
b	0.648	0.346	1.307
d	1.553	1.357	1.763
e	7.241	3.714	17.509
sigma	0.311	0.257	0.389

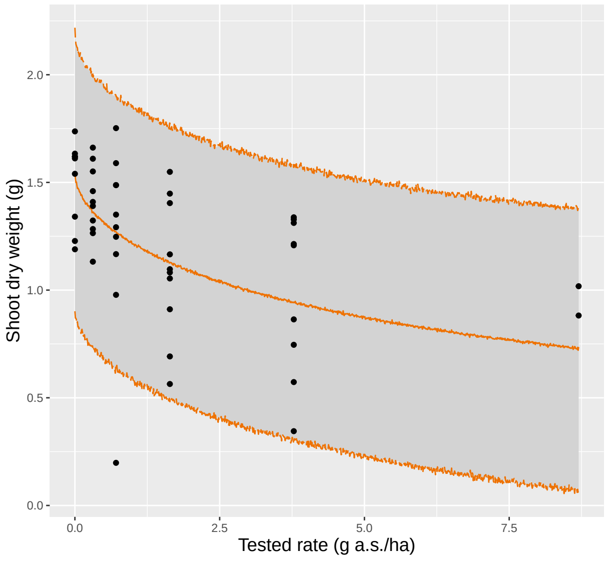

(a) Dose-response curve

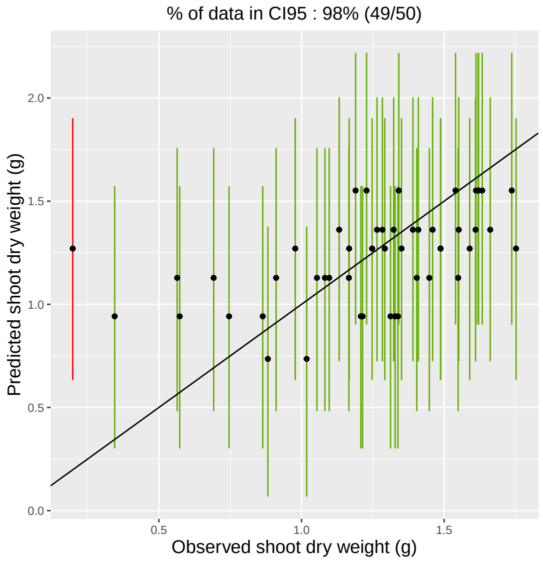

(b) Posterior predictive check (PPC)

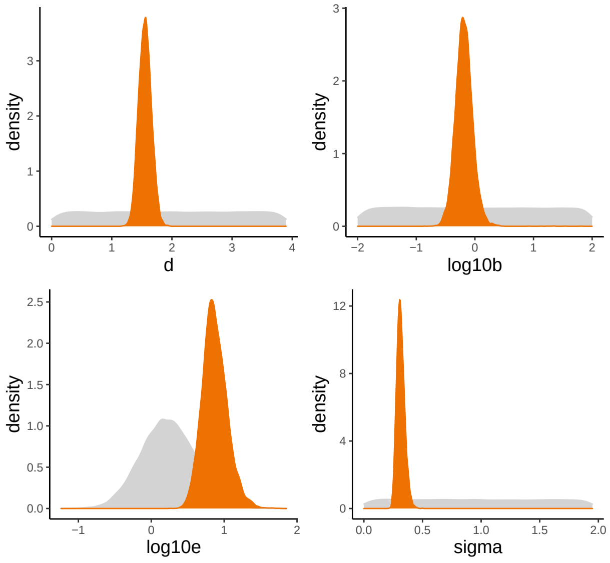

(c) Priors and posteriors

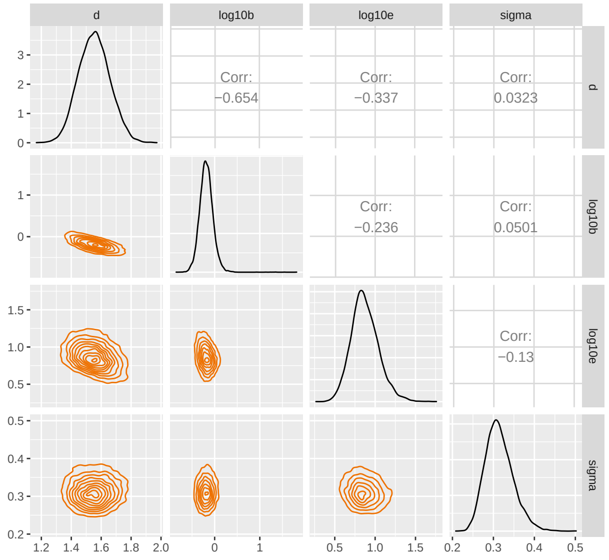

(d) Correlations between parameters

Figure 6: Dose-response curve (a), PPC (b), prior and posterior distributions (c) and correlations between parameters (d).

Data set: HELAN_SE_weight

Table 7: Summary of parameter estimates for HELAN_SE_weight data set

Parameter	median	Q2.5	Q97.5
b	0.901	0.592	1.408
d	0.892	0.820	0.972
e	14.277	9.475	24.435
sigma	0.135	0.111	0.169

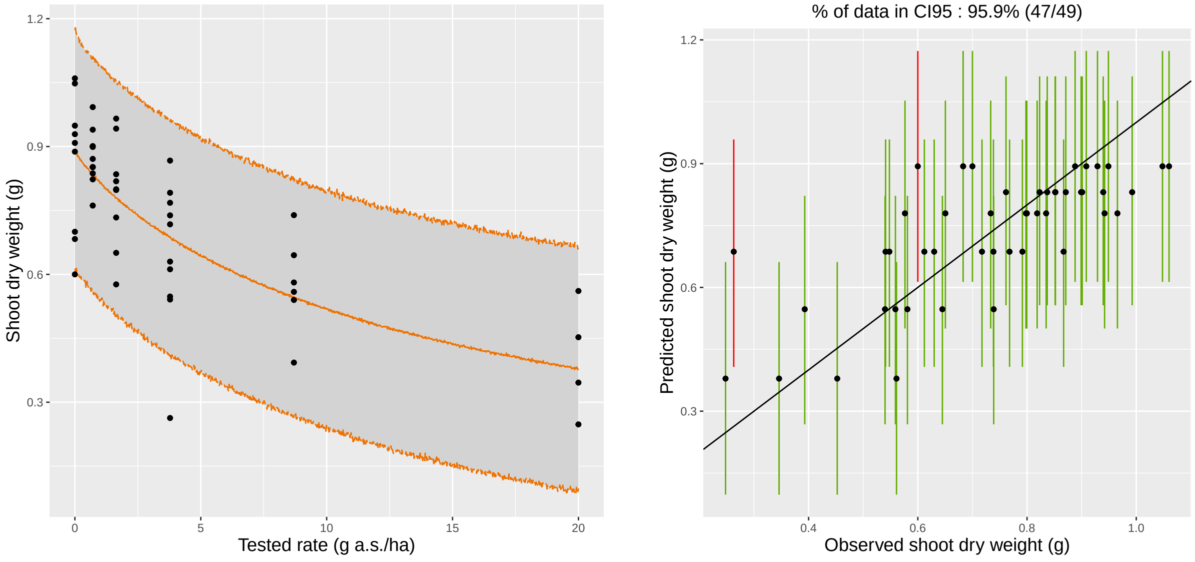

(a) Dose-response curve (b) Posterior predictive check (PPC)

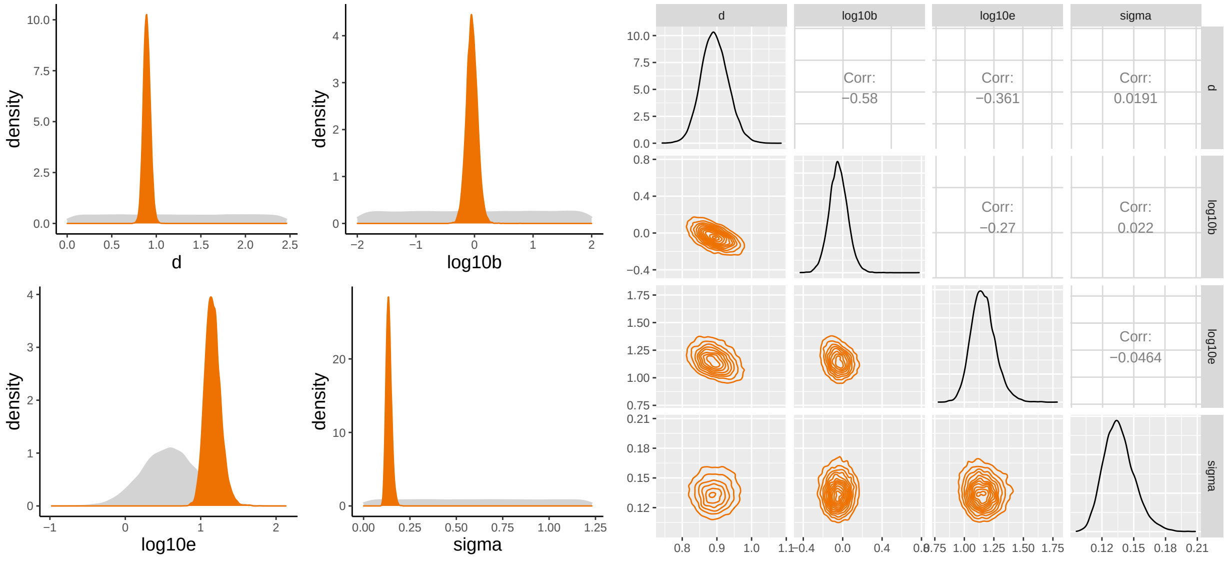

(c) Priors and posteriors (d) Correlations between parameters

Figure 7: Dose-response curve (a), PPC (b), prior and posterior distributions (c) and correlations between parameters (d).

Data set: LOLPE_SE_weight

Table 8: Summary of parameter estimates for LOLPE_SE_weight data set

Parameter	median	Q2.5	Q97.5
b	1.744	0.683	16.338
d	0.155	0.130	0.183
e	0.330	0.210	0.608
sigma	0.034	0.024	0.052

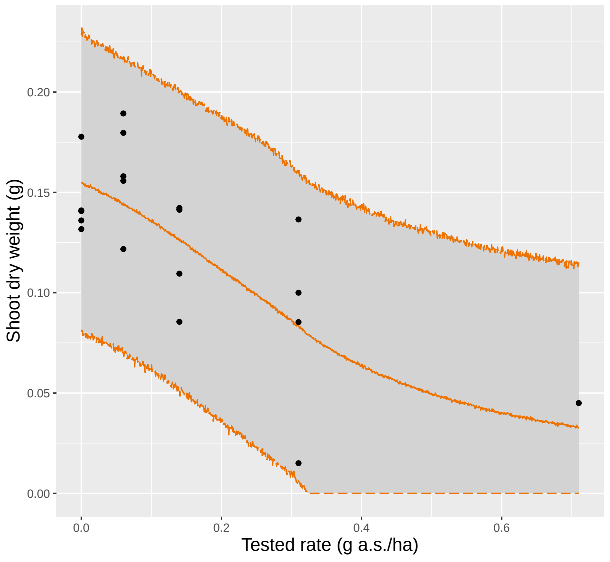

(a) Dose-response curve

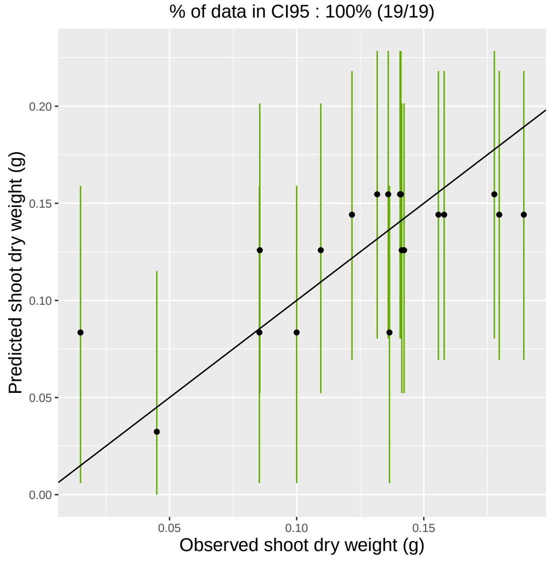

(b) Posterior predictive check (PPC)

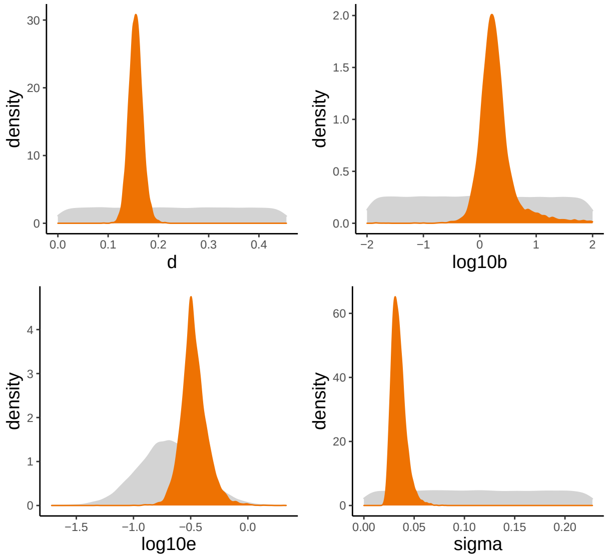

(c) Priors and posteriors

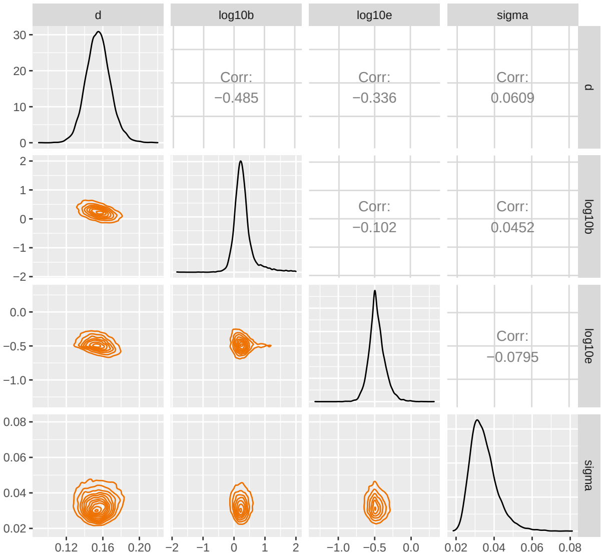

(d) Correlations between parameters

Figure 8: Dose-response curve (a), PPC (b), prior and posterior distributions (c) and correlations between parameters (d).

Data set: LYPES_SE_weight

Table 9: Summary of parameter estimates for LYPES_SE_weight data set

Parameter	median	Q2.5	Q97.5
b	2.810	0.934	60.826
d	1.648	1.499	1.844
e	1.525	0.832	2.727
sigma	0.345	0.272	0.460

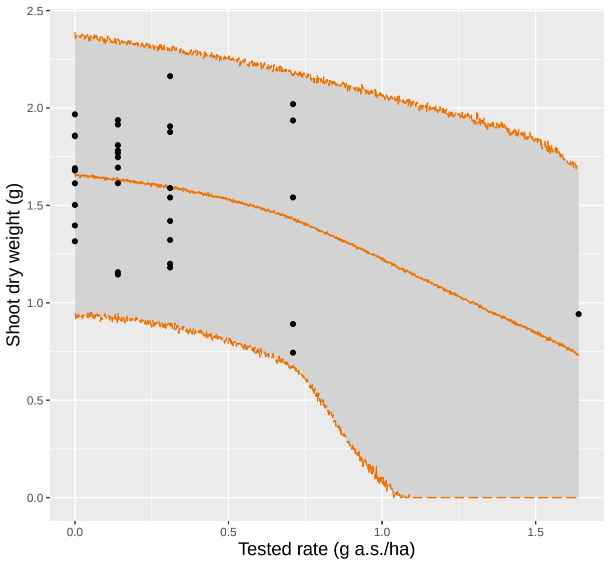

(a) Dose-response curve

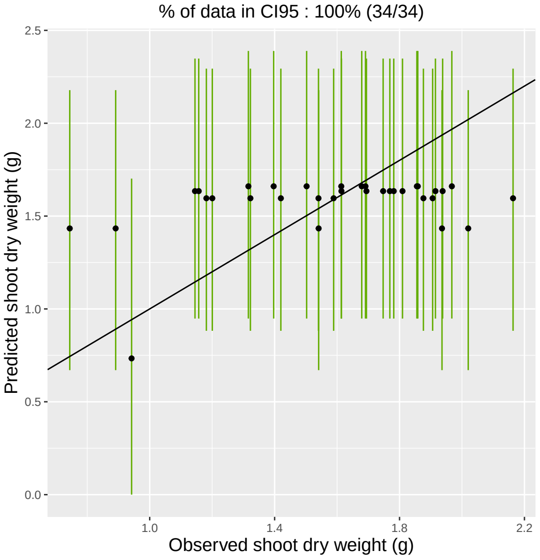

(b) Posterior predictive check (PPC)

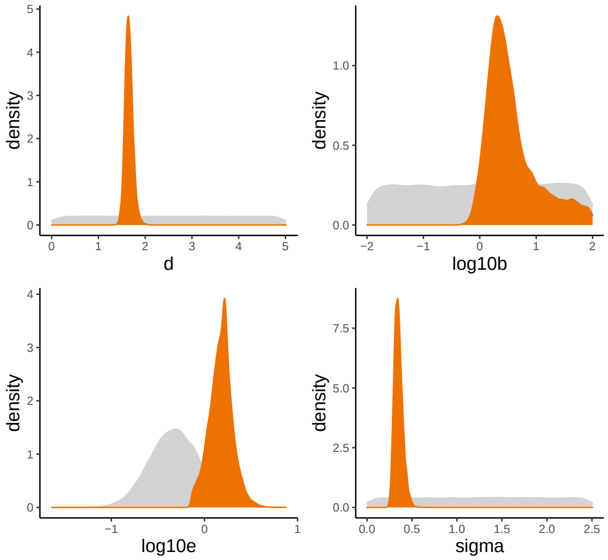

(c) Priors and posteriors

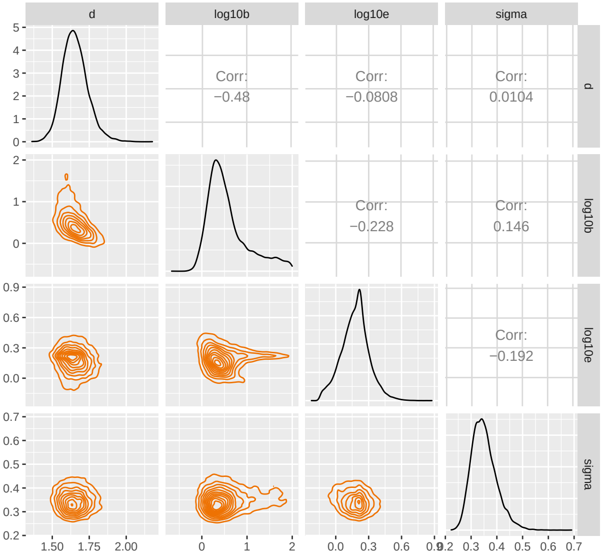

(d) Correlations between parameters

Figure 9: Dose-response curve (a), PPC (b), prior and posterior distributions (c) and correlations between parameters (d).

Data set: ZEAMA_SE_weight

Table 10: Summary of parameter estimates for ZEAMA_SE_weight data set

Parameter	median	Q2.5	Q97.5
b	0.850	0.494	1.807
d	4.969	4.531	5.471
e	25.897	18.105	43.959
sigma	0.728	0.610	0.888

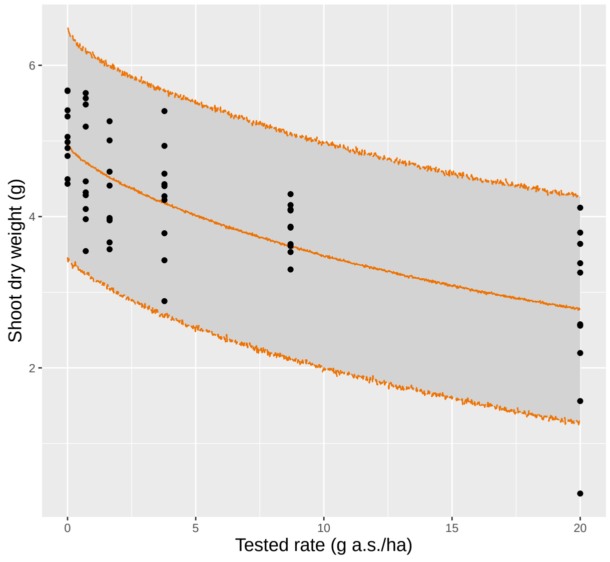

(a) Dose-response curve

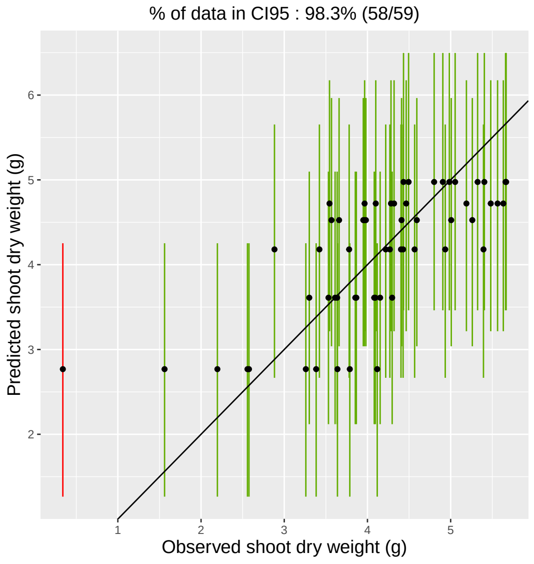

(b) Posterior predictive check (PPC)

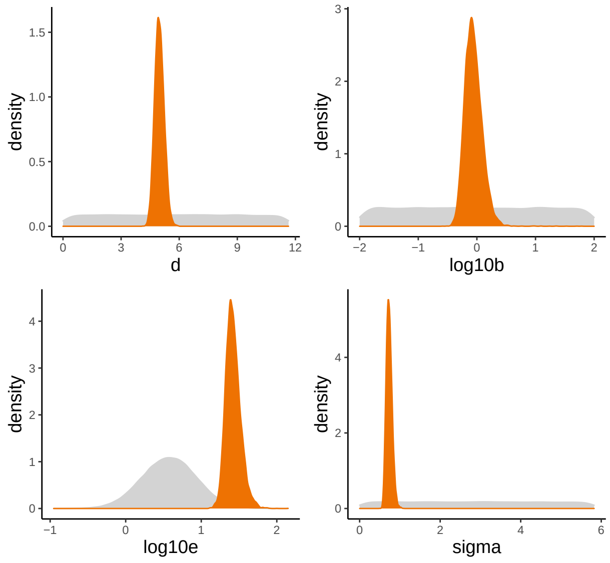

(c) Priors and posteriors

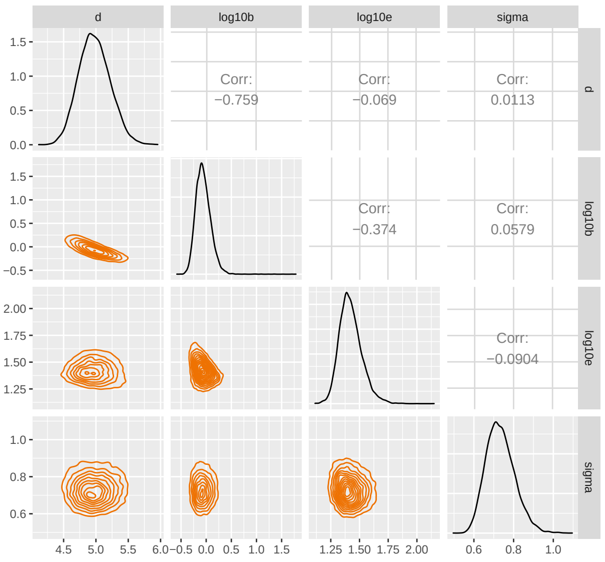

(d) Correlations between parameters

Figure 10: Dose-response curve (a), PPC (b), prior and posterior distributions (c) and correlations between parameters (d).
