# Supplementary material for: How to account for the uncertainty from standard toxicity tests in species sensitivity distributions: An example in non-target plants
Source: PLoS One. 2021 Jan 7;16(1):e0245071. doi: 10.1371/journal.pone.0245071 (PMC7790375; doi:10.1371/journal.pone.0245071)
Supplement: S1 Archive — It is a zip file containing seven folders (one folder per case study). Each folder contains five files report_xxx.pdf with detailed results of the dose-response analyses, one file corresponding to does-response analysis per endpoint. It also contains one file ER50_censoring.pdf for censored ER50 and one file SSD_analyses.pdf for results of SSD analyses. (ZIP) [file pone.0245071.s004.zip › S1_archive/Study5/report_VV_weight.pdf]

Dose-response analysis

Study 5

Vegetative Vigour test - shoot dry VV_weight endpoint

25 June 2020

Contact: sandrine.charles@univ-lyon1.fr

This is a report which provides results on all performed dose-response analyses for the shoot dry VV_weight endpoint of the Vegetative Vigour test for study 5.

Contents

Data set: ALLCE_VV_weight . . . 2
Data set: AVESA_VV_weight . . . 3
Data set: BEAVA_VV_weight . . . 4
Data set: BRSNW_VV_weight . . . 5
Data set: CUMSA_VV_weight . . . 6
Data set: GLXMA_VV_weight . . . 7
Data set: HELAN_VV_weight . . . 8
Data set: LOLPE_VV_weight . . . 9
Data set: LYPES_VV_weight . . . 10
Data set: ZEAMA_VV_weight . . . 11

Data set: ALLCE_VV_weight

Table 1: Summary of parameter estimates for ALLCE_VV_weight data set

Parameter	median	Q2.5	Q97.5
b	20.818	0.702	93.181
d	0.156	0.138	0.187
e	27.682	20.434	63.574
sigma	0.047	0.037	0.065

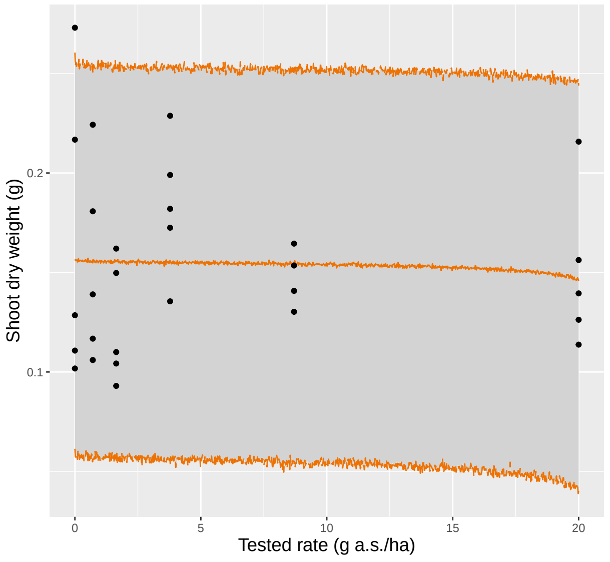

(a) Dose-response curve

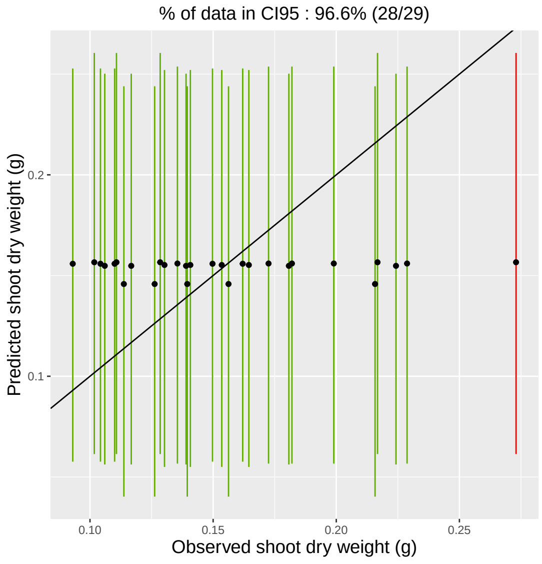

(b) Posterior predictive check (PPC)

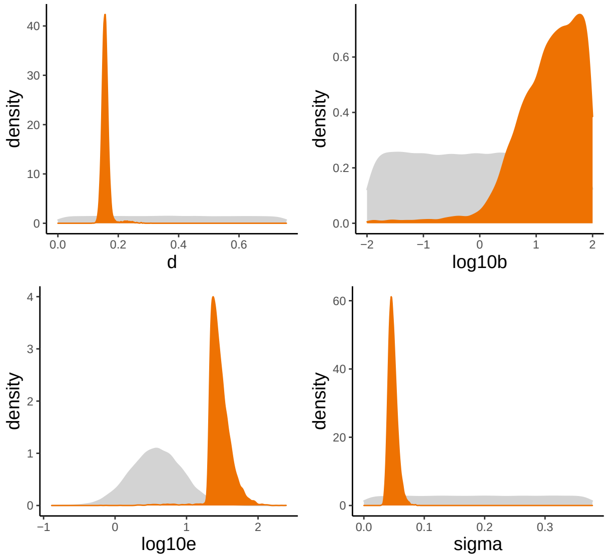

(c) Priors and posteriors

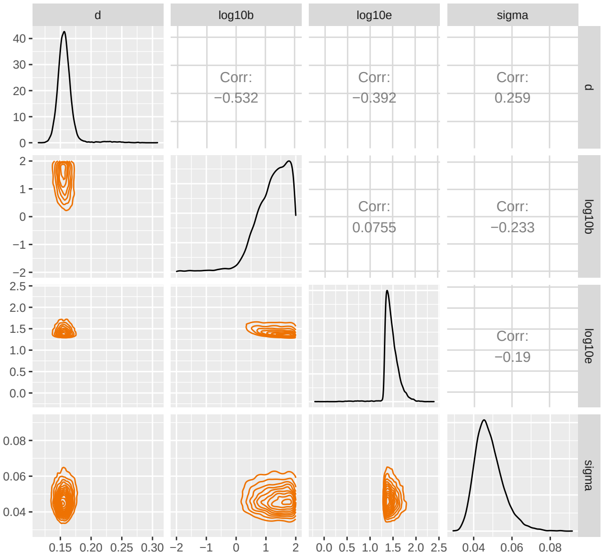

(d) Correlations between parameters

Figure 1: Dose-response curve (a), PPC (b), prior and posterior distributions (c) and correlations between parameters (d).

Data set: AVESA_VV_weight

Table 2: Summary of parameter estimates for AVESA_VV_weight data set

Parameter	median	Q2.5	Q97.5
b	2.903	0.819	65.617
d	2.225	2.110	2.380
e	35.222	20.742	91.852
sigma	0.219	0.168	0.301

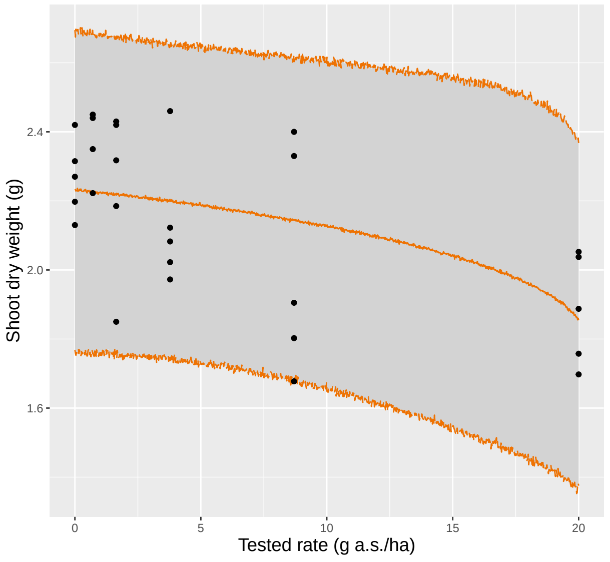

(a) Dose-response curve

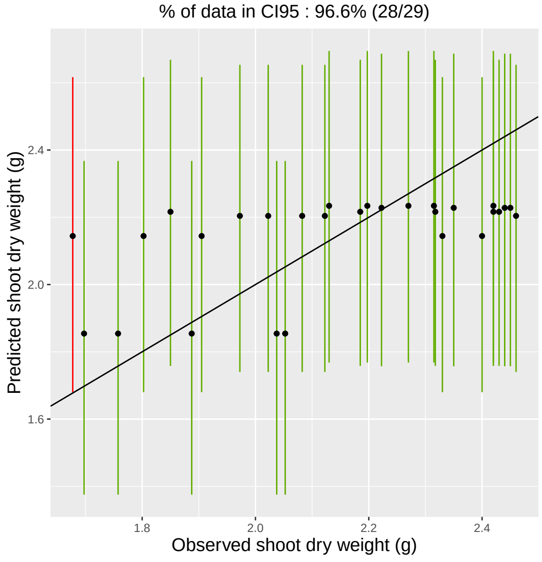

(b) Posterior predictive check (PPC)

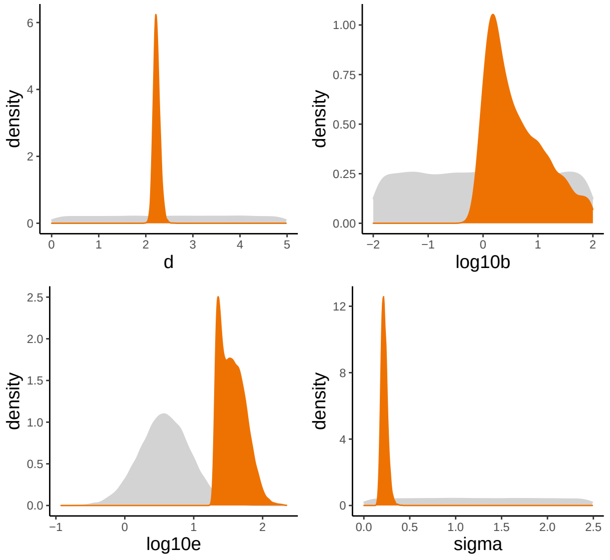

(c) Priors and posteriors

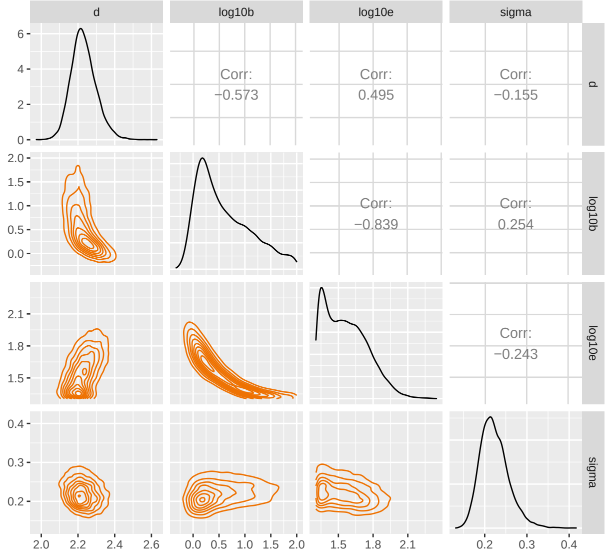

(d) Correlations between parameters

Figure 2: Dose-response curve (a), PPC (b), prior and posterior distributions (c) and correlations between parameters (d).

Data set: BEAVA_VV_weight

Table 3: Summary of parameter estimates for BEAVA_VV_weight data set

Parameter	median	Q2.5	Q97.5
b	0.363	0.222	23.472
d	5.023	3.984	5.590
e	77.452	23.492	202.775
sigma	0.649	0.536	0.810

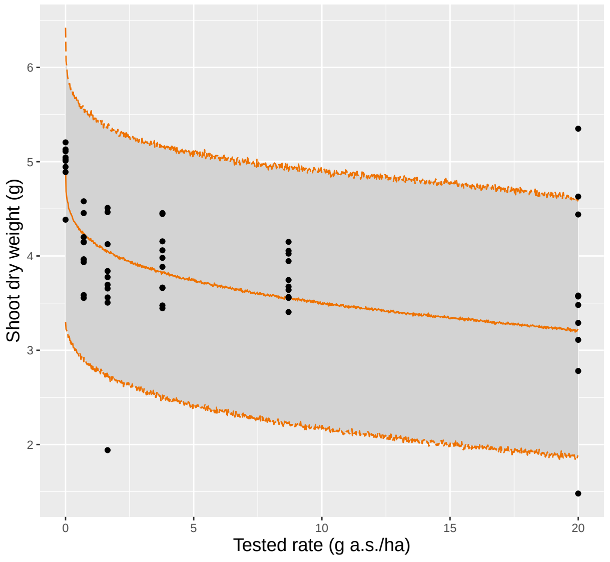

(a) Dose-response curve

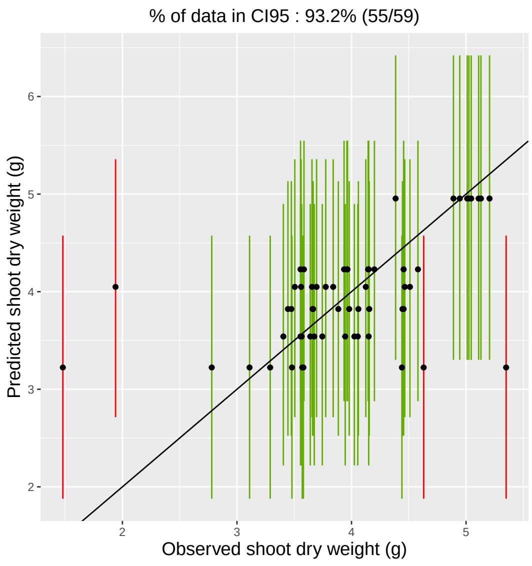

(b) Posterior predictive check (PPC)

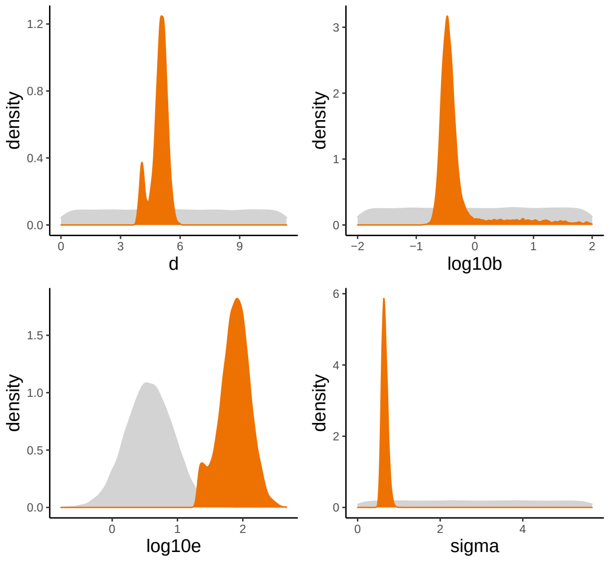

(c) Priors and posteriors

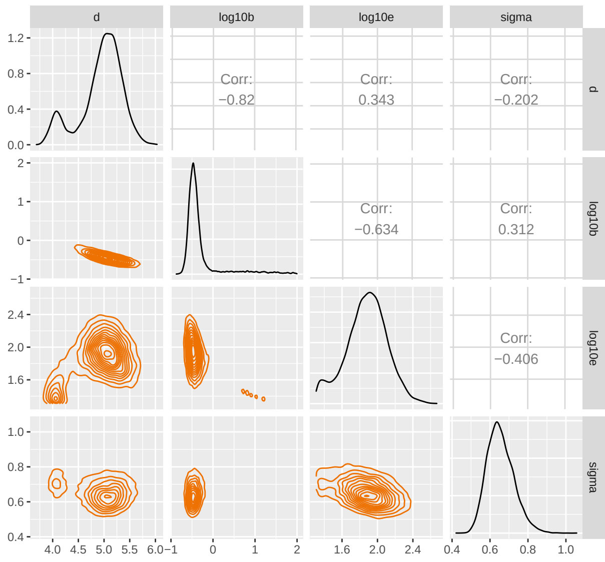

(d) Correlations between parameters

Figure 3: Dose-response curve (a), PPC (b), prior and posterior distributions (c) and correlations between parameters (d).

Data set: BRSNW_VV_weight

Table 4: Summary of parameter estimates for BRSNW_VV_weight data set

Parameter	median	Q2.5	Q97.5
b	1.426	0.934	2.180
d	9.567	8.955	10.274
e	16.323	12.903	21.359
sigma	1.429	1.196	1.743

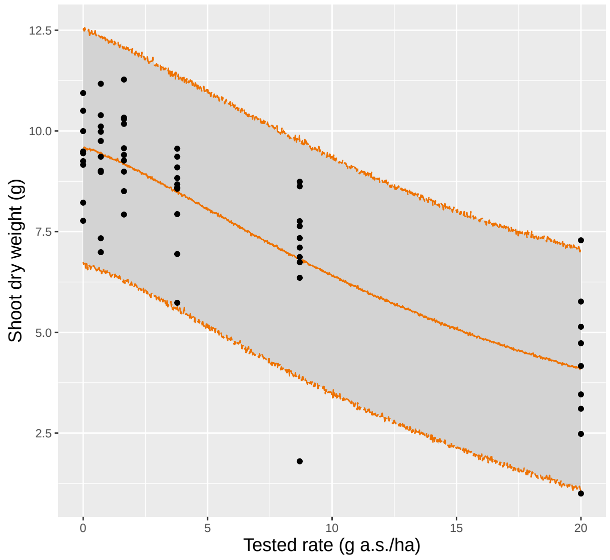

(a) Dose-response curve

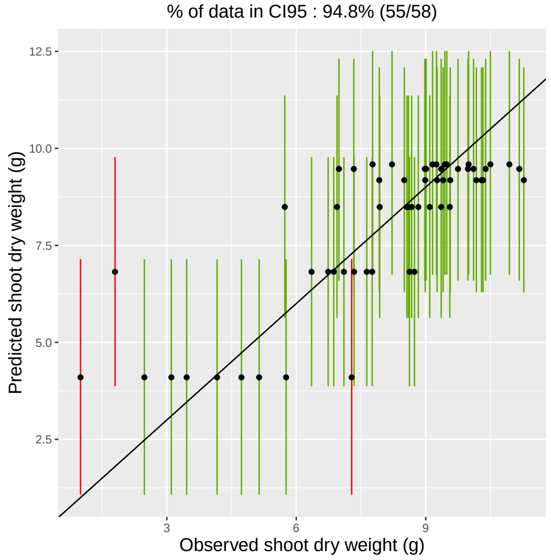

(b) Posterior predictive check (PPC)

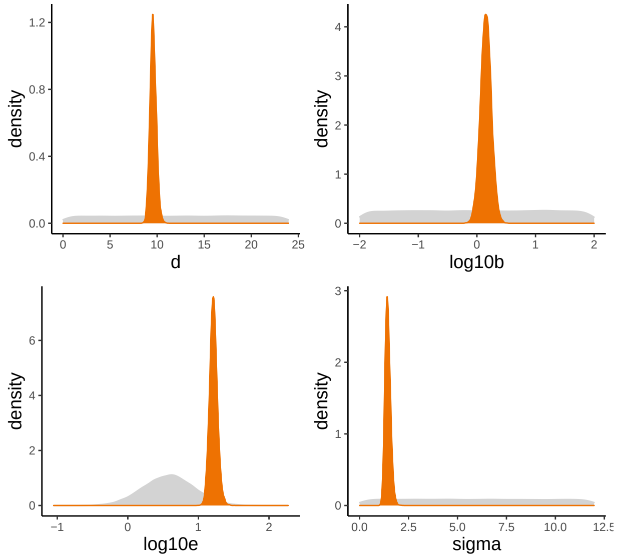

(c) Priors and posteriors

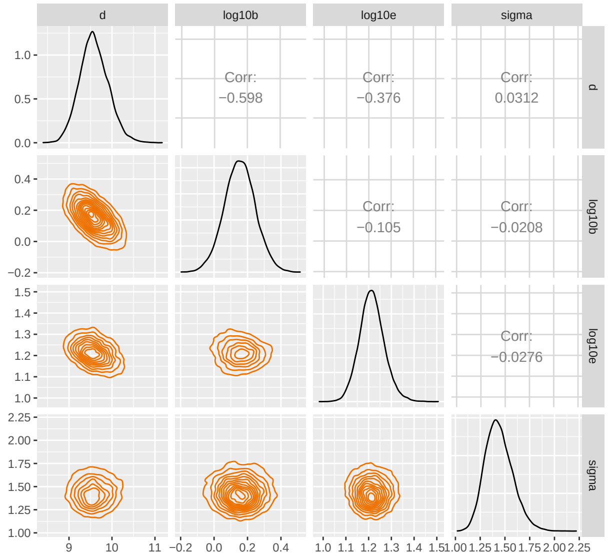

(d) Correlations between parameters

Figure 4: Dose-response curve (a), PPC (b), prior and posterior distributions (c) and correlations between parameters (d).

Data set: CUMSA_VV_weight

Table 5: Summary of parameter estimates for CUMSA_VV_weight data set

Parameter	median	Q2.5	Q97.5
b	0.223	0.154	0.313
d	14.567	13.713	15.448
e	39.486	18.517	95.861
sigma	1.281	1.069	1.578

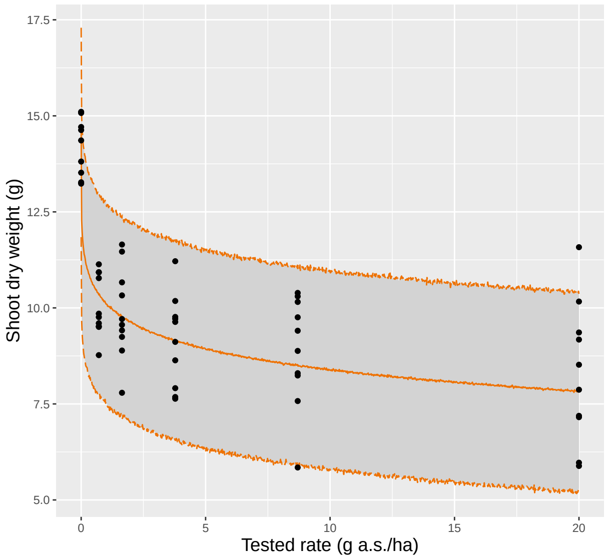

(a) Dose-response curve

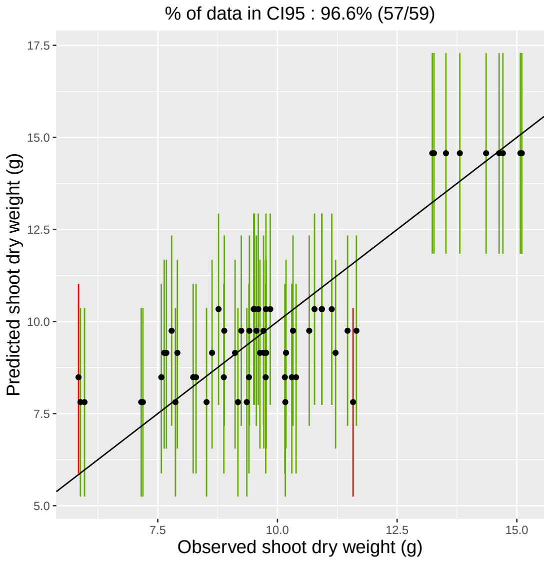

(b) Posterior predictive check (PPC)

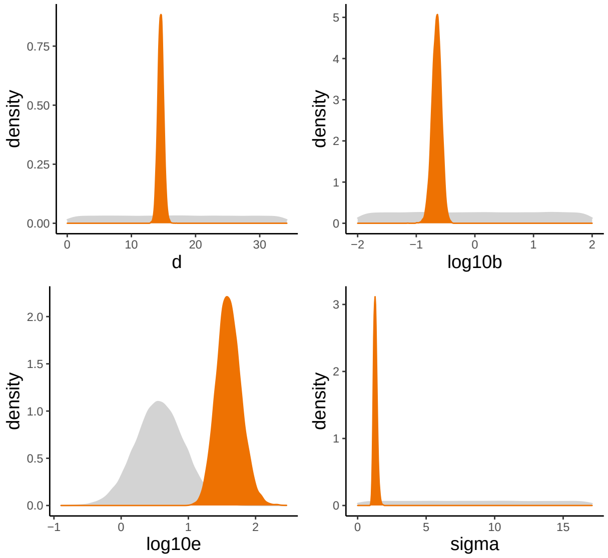

(c) Priors and posteriors

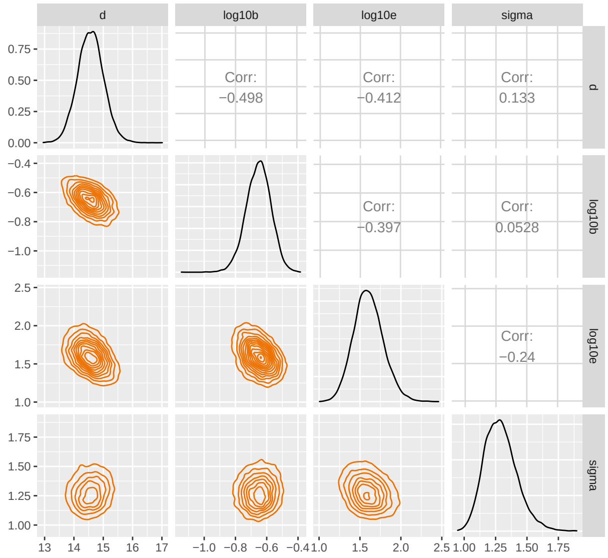

(d) Correlations between parameters

Figure 5: Dose-response curve (a), PPC (b), prior and posterior distributions (c) and correlations between parameters (d).

Data set: GLXMA_VV_weight

Table 6: Summary of parameter estimates for GLXMA_VV_weight data set

Parameter	median	Q2.5	Q97.5
b	12.401	1.747	86.278
d	4.158	4.052	4.275
e	27.218	20.795	75.760
sigma	0.357	0.300	0.437

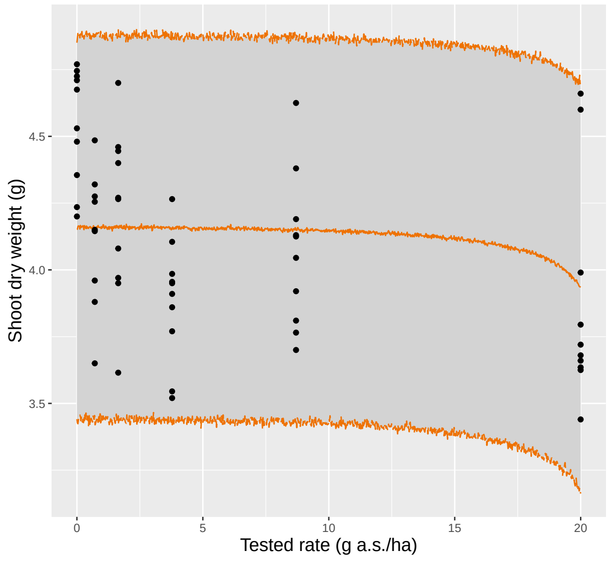

(a) Dose-response curve

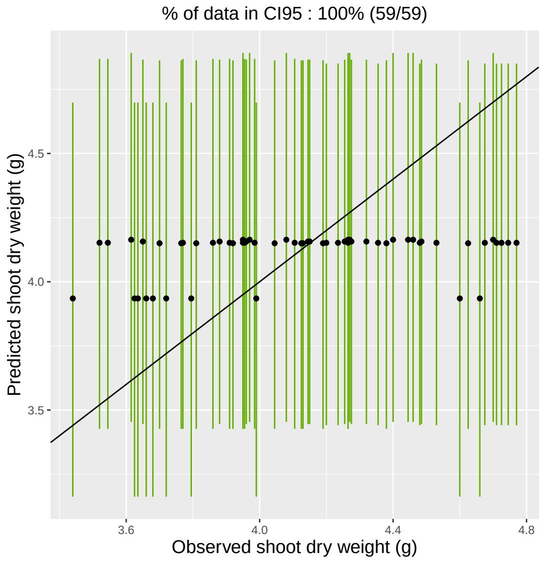

(b) Posterior predictive check (PPC)

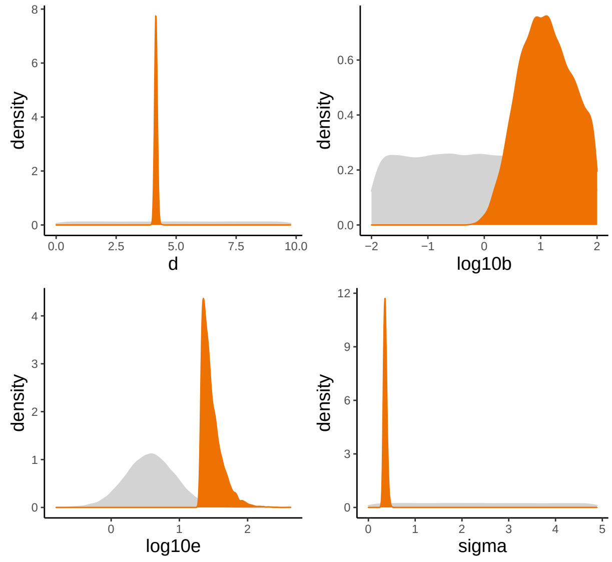

(c) Priors and posteriors

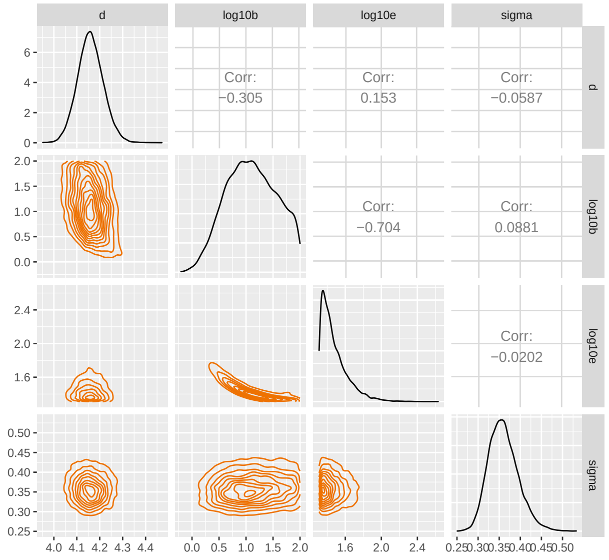

(d) Correlations between parameters

Figure 6: Dose-response curve (a), PPC (b), prior and posterior distributions (c) and correlations between parameters (d).

Data set: HELAN_VV_weight

Table 7: Summary of parameter estimates for HELAN_VV_weight data set

Parameter	median	Q2.5	Q97.5
b	34.254	5.156	95.332
d	4.532	4.430	4.631
e	29.094	21.464	69.356
sigma	0.385	0.322	0.470

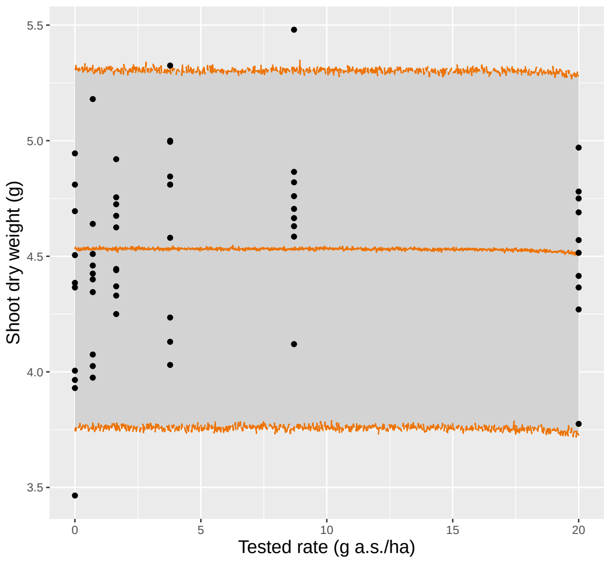

(a) Dose-response curve

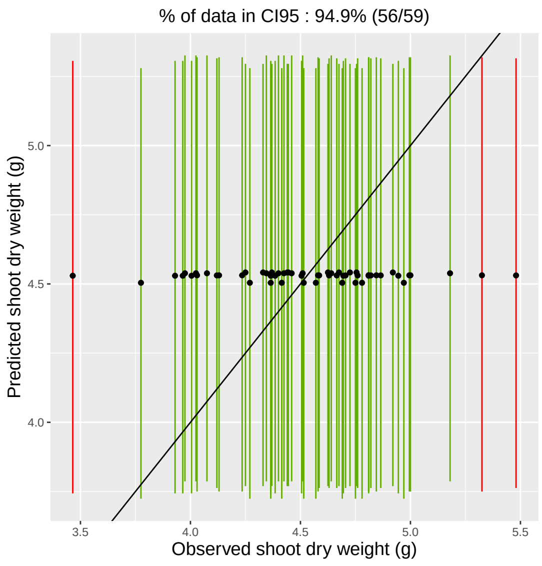

(b) Posterior predictive check (PPC)

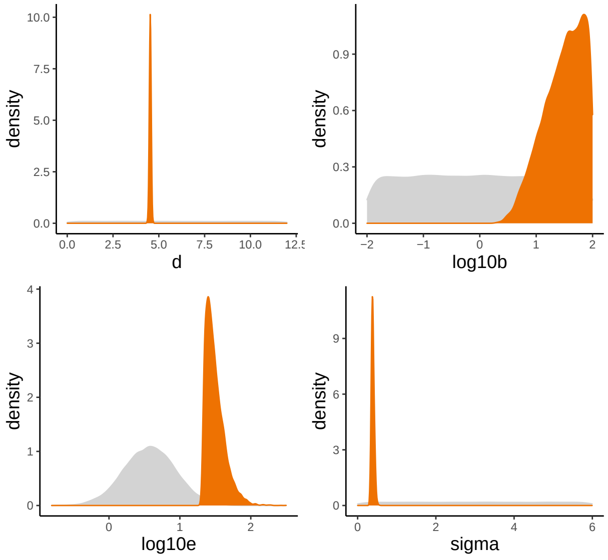

(c) Priors and posteriors

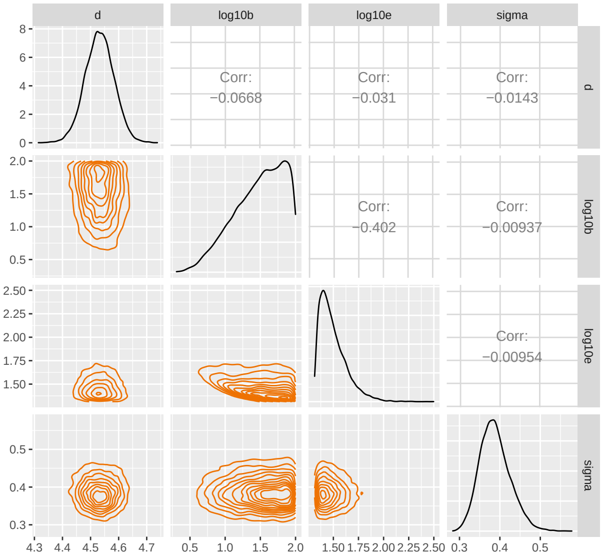

(d) Correlations between parameters

Figure 7: Dose-response curve (a), PPC (b), prior and posterior distributions (c) and correlations between parameters (d).

Data set: LOLPE_VV_weight

Table 8: Summary of parameter estimates for LOLPE_VV_weight data set

Parameter	median	Q2.5	Q97.5
b	1.096	0.490	2.631
d	0.279	0.239	0.330
e	10.853	5.834	20.123
sigma	0.059	0.046	0.081

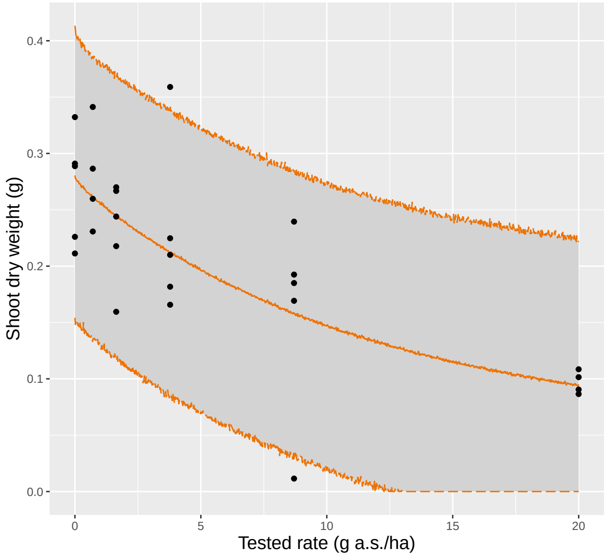

(a) Dose-response curve

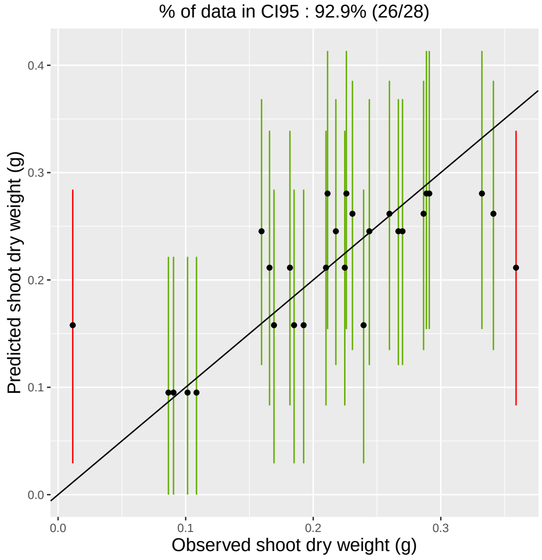

(b) Posterior predictive check (PPC)

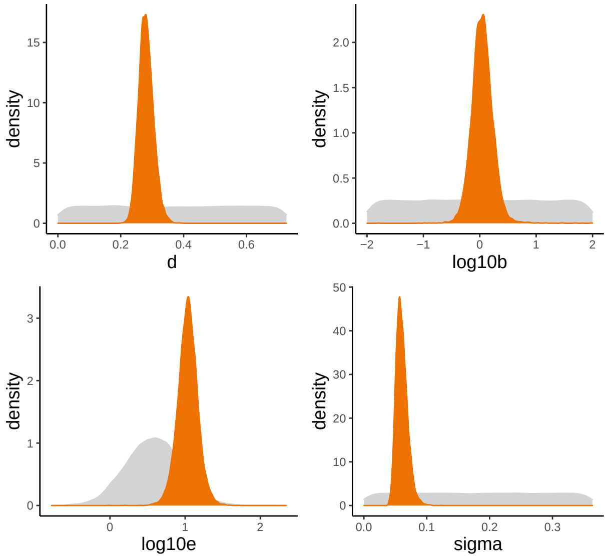

(c) Priors and posteriors

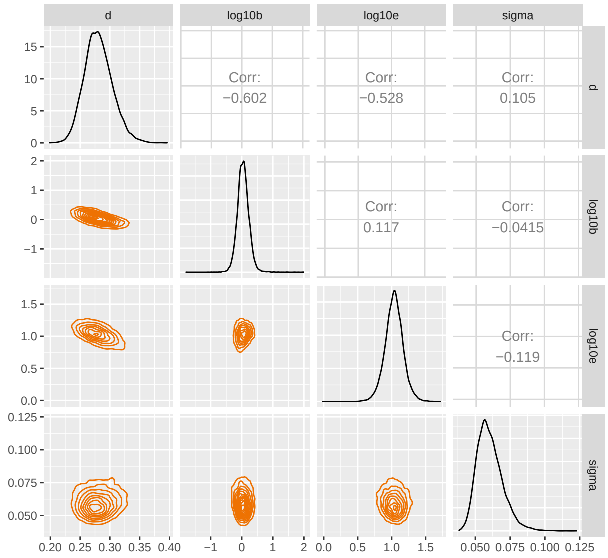

(d) Correlations between parameters

Figure 8: Dose-response curve (a), PPC (b), prior and posterior distributions (c) and correlations between parameters (d).

Data set: LYPES_VV_weight

Table 9: Summary of parameter estimates for LYPES_VV_weight data set

Parameter	median	Q2.5	Q97.5
b	7.485	1.332	70.260
d	5.006	4.902	5.128
e	26.859	20.648	83.431
sigma	0.349	0.292	0.427

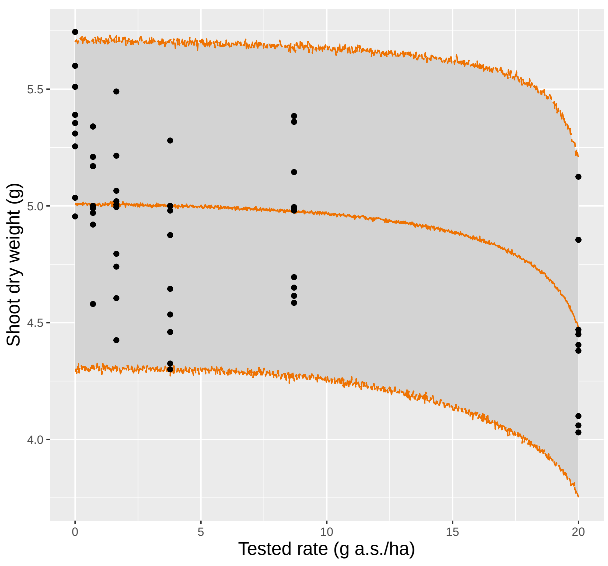

(a) Dose-response curve

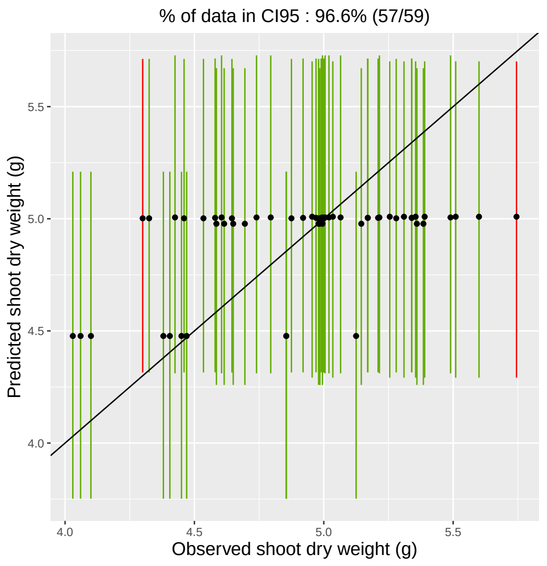

(b) Posterior predictive check (PPC)

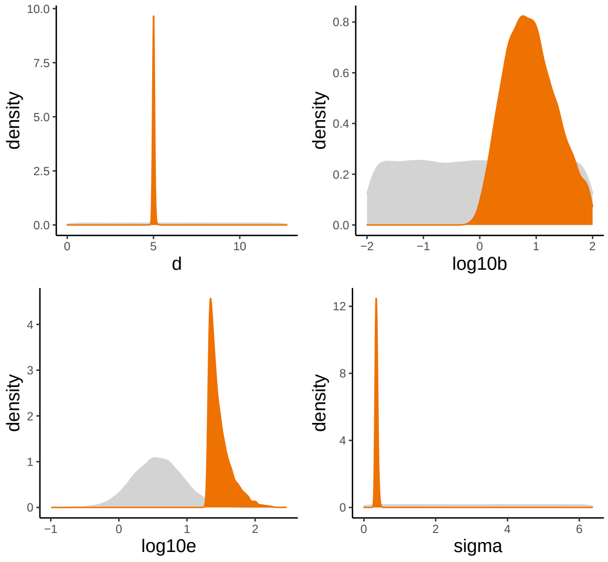

(c) Priors and posteriors

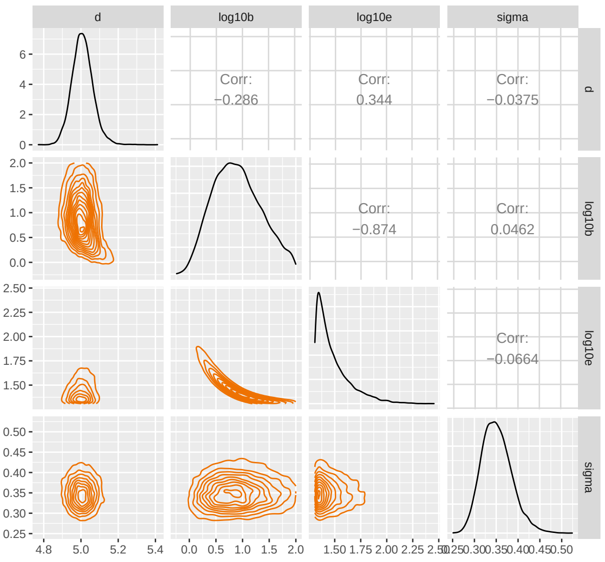

(d) Correlations between parameters

Figure 9: Dose-response curve (a), PPC (b), prior and posterior distributions (c) and correlations between parameters (d).

Data set: ZEAMA_VV_weight

Table 10: Summary of parameter estimates for ZEAMA_VV_weight data set

Parameter	median	Q2.5	Q97.5
b	0.556	0.384	0.880
d	8.792	8.160	9.438
e	71.249	39.840	150.227
sigma	1.018	0.850	1.256

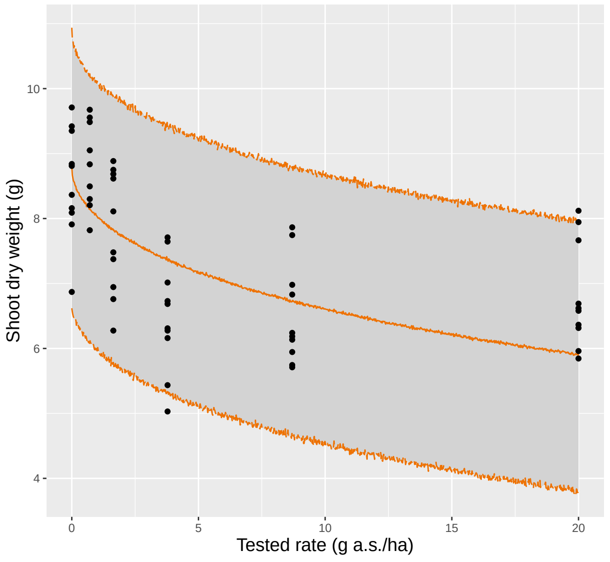

(a) Dose-response curve

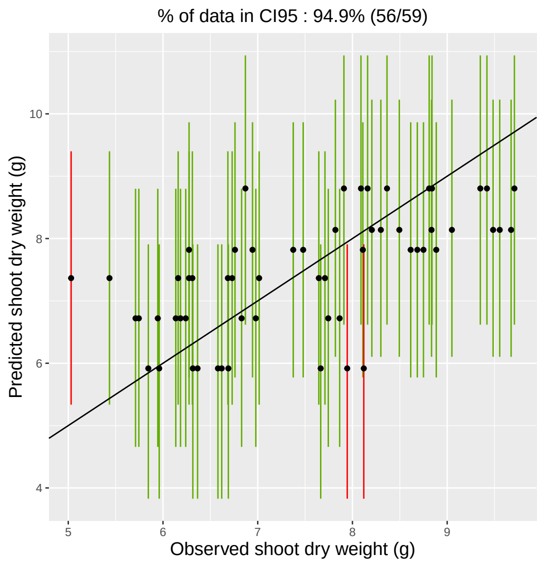

(b) Posterior predictive check (PPC)

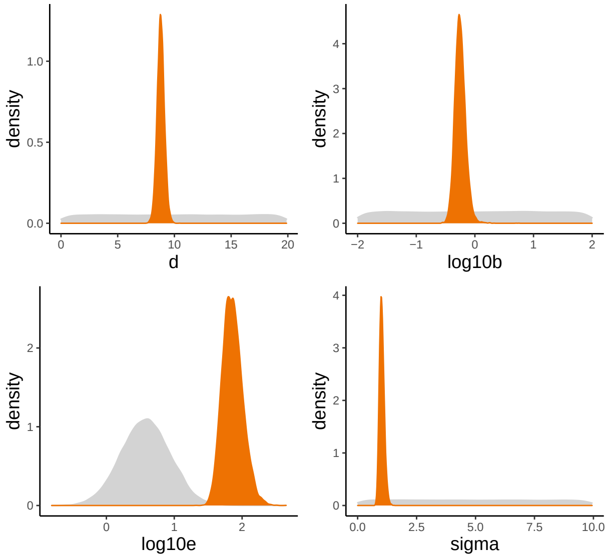

(c) Priors and posteriors

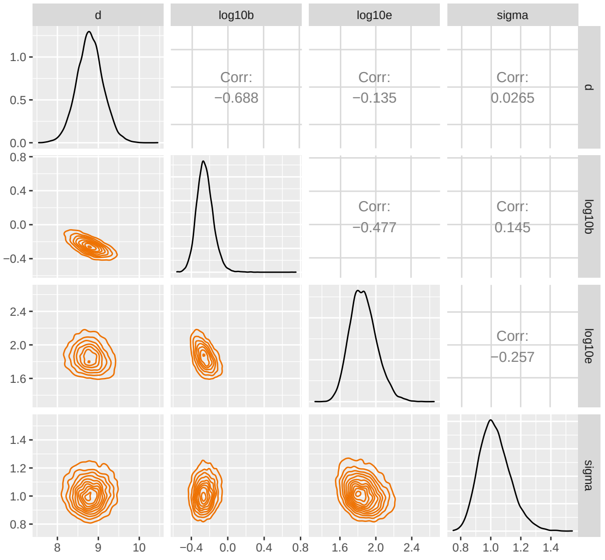

(d) Correlations between parameters

Figure 10: Dose-response curve (a), PPC (b), prior and posterior distributions (c) and correlations between parameters (d).
