# Supplementary material for: How to account for the uncertainty from standard toxicity tests in species sensitivity distributions: An example in non-target plants
Source: PLoS One. 2021 Jan 7;16(1):e0245071. doi: 10.1371/journal.pone.0245071 (PMC7790375; doi:10.1371/journal.pone.0245071)
Supplement: S1 Archive — It is a zip file containing seven folders (one folder per case study). Each folder contains five files report_xxx.pdf with detailed results of the dose-response analyses, one file corresponding to does-response analysis per endpoint. It also contains one file ER50_censoring.pdf for censored ER50 and one file SSD_analyses.pdf for results of SSD analyses. (ZIP) [file pone.0245071.s004.zip › S1_archive/Study6/ER50_censoring.pdf]

ER_{50} censoring after dose-response analyses

Study 6

25 June 2020

Contact: sandrine.charles@univ-lyon1.fr

This document provides the ER_{50} censoring after dose-response analyses for study 6, including four parts:

- Raw data description (test item rates);
- Summary on ER_{50} from a Bayesian dose-response fit;
- Censoring on ER_{50} according to two criteria;
  - Criterion 1 (C_1) based on the ratio of the probablities, that is the ratio of the probability that the ER_{50} lies within the range of the LCI95 and the maximum tested rate over the probability that the ER_{50} lies in the CI95;
  - Criterion 2 (C_2) based on the ratio of distances, that is the ratio of the distance between the LCI95 of ER_{50} and the maximum tested rate over the extend of the CI95 of ER_{50};
- Graphical presentation of censored ER_{50} per species per endpoint.

Meaning of symbols used in the document

- min_rate: minimum tested rate;
- max_rate: maximum tested rate;
- ER_{50}: 50% effective rate;
- CI95: 95% credible interval;
- ER_{50}_median: median of ER_{50};
- ER_{50}_LCI95: lower bound of the CI95 of ER_{50};
- ER_{50}_UCI95: upper bound of the CI95 of ER_{50};
- C_1: criterion based on the ratio of probabilities;
- C_2: criterion based on the ratio of distances;
- T: decision threshold;
- Censored ER_{50} [lower bound; upper bound]:
  - C1T0.0: censored ER_{50} according to C_1 with T = 0.0;
  - C1T0.2: censored ER_{50} according to C_1 with T = 0.2;

- C1T0.4: censored ER_{50} according to C_1 with T = 0.4;
- C1T0.5: censored ER_{50} according to C_1 with T = 0.5;
- C1T0.6: censored ER_{50} according to C_1 with T = 0.6;
- C1T0.8: censored ER_{50} according to C_1 with T = 0.8;
- C1T1.0: censored ER_{50} according to C_1 with T = 1.0;
- C2T0.0: censored ER_{50} according to C_2 with T = 0.0;
- C2T0.2: censored ER_{50} according to C_2 with T = 0.2;
- C2T0.4: censored ER_{50} according to C_2 with T = 0.4;
- C2T0.5: censored ER_{50} according to C_2 with T = 0.5;
- C2T0.6: censored ER_{50} according to C_2 with T = 0.6;
- C2T0.8: censored ER_{50} according to C_2 with T = 0.8;
- C2T1.0: censored ER_{50} according to C_2 with T = 1.0.

Seeding emergence (SE) test of study 6

Raw data description (test item rates) for the SE test

```
Number of species:  10
EPPO code:  ALLCE AVESA BEAVA BRSNW CUMSA GLXMA HELAN LYPES TRZAW ZEAMA
```

Table 1: Correspondance between species and common names according to the EPPO code

EPPO code	Species name	Common name
ALLCE	*Allium cepa*	Onion
AVESA	*Avena sativa*	Oat
BEAVA	*Beta vulgaris*	Sugar beet
BRSNW	*Brassica napus winter*	Oilseed rape
CUMSA	*Cucumis sativus*	Cucumber
GLXMA	*Glycine max*	Soybean
HELAN	*Helianthus annuus*	Sunflower
LYPES	*Solanum lycopersicum*	Tomato
TRZAW	*Triticum aestivum winter*	Winter wheat
ZEAMA	*Zea mays subs. Amylacea*	Corn

Table 2: Tested rates per species (g product/ha) for the SE test of study 6

ALLCE	AVESA	BEAVA	BRSNW	CUMSA	GLXMA	HELAN	LYPES	TRZAW	ZEAMA
0.0	0.0	0.0	0.0	0.0	0.0	0.0	0.0	0.0	0.0
6.4	6.4	6.4	6.4	6.4	6.4	6.4	6.4	6.4	6.4
14.1	14.1	14.1	14.1	14.1	14.1	14.1	14.1	14.1	14.1
31.0	31.0	31.0	31.0	31.0	31.0	31.0	31.0	31.0	31.0
68.2	68.2	68.2	68.2	68.2	68.2	68.2	68.2	68.2	68.2
150.0	150.0	150.0	150.0	150.0	150.0	150.0	150.0	150.0	150.0

SE test - emergence endpoint

Table 3: Minimum and maximum tested rates, median, quantile 2.5% and quantile 97.5% of the ER_{50} posterior distribution - emergence endpoint of the SE test of study 6

species	min_rate	max_rate	ER_{50}_median	ER_{50}_LCI95	ER_{50}_UCI95
ALLCE	6.4	150	165.84639	93.41040	344.66856
AVESA	6.4	150	319.15495	184.58742	714.32574
BEAVA	6.4	150	292.41911	188.07722	617.98770
BRSNW	6.4	150	66.48414	50.83196	90.23706
CUMSA	6.4	150	528.09684	262.24843	1274.03724
GLXMA	6.4	150	525.31626	272.33937	1279.09122
HELAN	6.4	150	466.65940	249.51915	1100.25552
LYPES	6.4	150	490.43485	239.84061	1224.28064
TRZAW	6.4	150	437.28725	237.79902	1049.46727
ZEAMA	6.4	150	411.48407	228.89682	947.33129

Table 4: Censored ER_{50} according to criterion 1 and the seven decision thresholds - emergence endpoint of the SE test of study 6

species	C1T0.0	C1T0.2	C1T0.4	C1T0.5	C1T0.6	C1T0.8	C1T1.0
ALLCE	[93.41; 344.67]	[93.41; 344.67]	[93.41; Inf)	[93.41; Inf)	[93.41; Inf)	[93.41; Inf)	[93.41; Inf)
AVESA	[150; Inf)	[150; Inf)	[150; Inf)	[150; Inf)	[150; Inf)	[150; Inf)	[150; Inf)
BEAVA	[150; Inf)	[150; Inf)	[150; Inf)	[150; Inf)	[150; Inf)	[150; Inf)	[150; Inf)
BRSNW	[50.83; 90.24]	[50.83; 90.24]	[50.83; 90.24]	[50.83; 90.24]	[50.83; 90.24]	[50.83; 90.24]	[50.83; 90.24]
CUMSA	[150; Inf)	[150; Inf)	[150; Inf)	[150; Inf)	[150; Inf)	[150; Inf)	[150; Inf)
GLXMA	[150; Inf)	[150; Inf)	[150; Inf)	[150; Inf)	[150; Inf)	[150; Inf)	[150; Inf)
HELAN	[150; Inf)	[150; Inf)	[150; Inf)	[150; Inf)	[150; Inf)	[150; Inf)	[150; Inf)
LYPES	[150; Inf)	[150; Inf)	[150; Inf)	[150; Inf)	[150; Inf)	[150; Inf)	[150; Inf)
TRZAW	[150; Inf)	[150; Inf)	[150; Inf)	[150; Inf)	[150; Inf)	[150; Inf)	[150; Inf)
ZEAMA	[150; Inf)	[150; Inf)	[150; Inf)	[150; Inf)	[150; Inf)	[150; Inf)	[150; Inf)

Table 5: Censored ER_{50} according to criterion 2 and the seven decision thresholds - emergence endpoint of the SE test of study 6

species	C2T0.0	C2T0.2	C2T0.4	C2T0.5	C2T0.6	C2T0.8	C2T1.0
ALLCE	[93.41; 344.67]	[93.41; 344.67]	[93.41; Inf)	[93.41; Inf)	[93.41; Inf)	[93.41; Inf)	[93.41; Inf)
AVESA	[150; Inf)	[150; Inf)	[150; Inf)	[150; Inf)	[150; Inf)	[150; Inf)	[150; Inf)
BEAVA	[150; Inf)	[150; Inf)	[150; Inf)	[150; Inf)	[150; Inf)	[150; Inf)	[150; Inf)
BRSNW	[50.83; 90.24]	[50.83; 90.24]	[50.83; 90.24]	[50.83; 90.24]	[50.83; 90.24]	[50.83; 90.24]	[50.83; 90.24]
CUMSA	[150; Inf)	[150; Inf)	[150; Inf)	[150; Inf)	[150; Inf)	[150; Inf)	[150; Inf)
GLXMA	[150; Inf)	[150; Inf)	[150; Inf)	[150; Inf)	[150; Inf)	[150; Inf)	[150; Inf)
HELAN	[150; Inf)	[150; Inf)	[150; Inf)	[150; Inf)	[150; Inf)	[150; Inf)	[150; Inf)
LYPES	[150; Inf)	[150; Inf)	[150; Inf)	[150; Inf)	[150; Inf)	[150; Inf)	[150; Inf)
TRZAW	[150; Inf)	[150; Inf)	[150; Inf)	[150; Inf)	[150; Inf)	[150; Inf)	[150; Inf)
ZEAMA	[150; Inf)	[150; Inf)	[150; Inf)	[150; Inf)	[150; Inf)	[150; Inf)	[150; Inf)

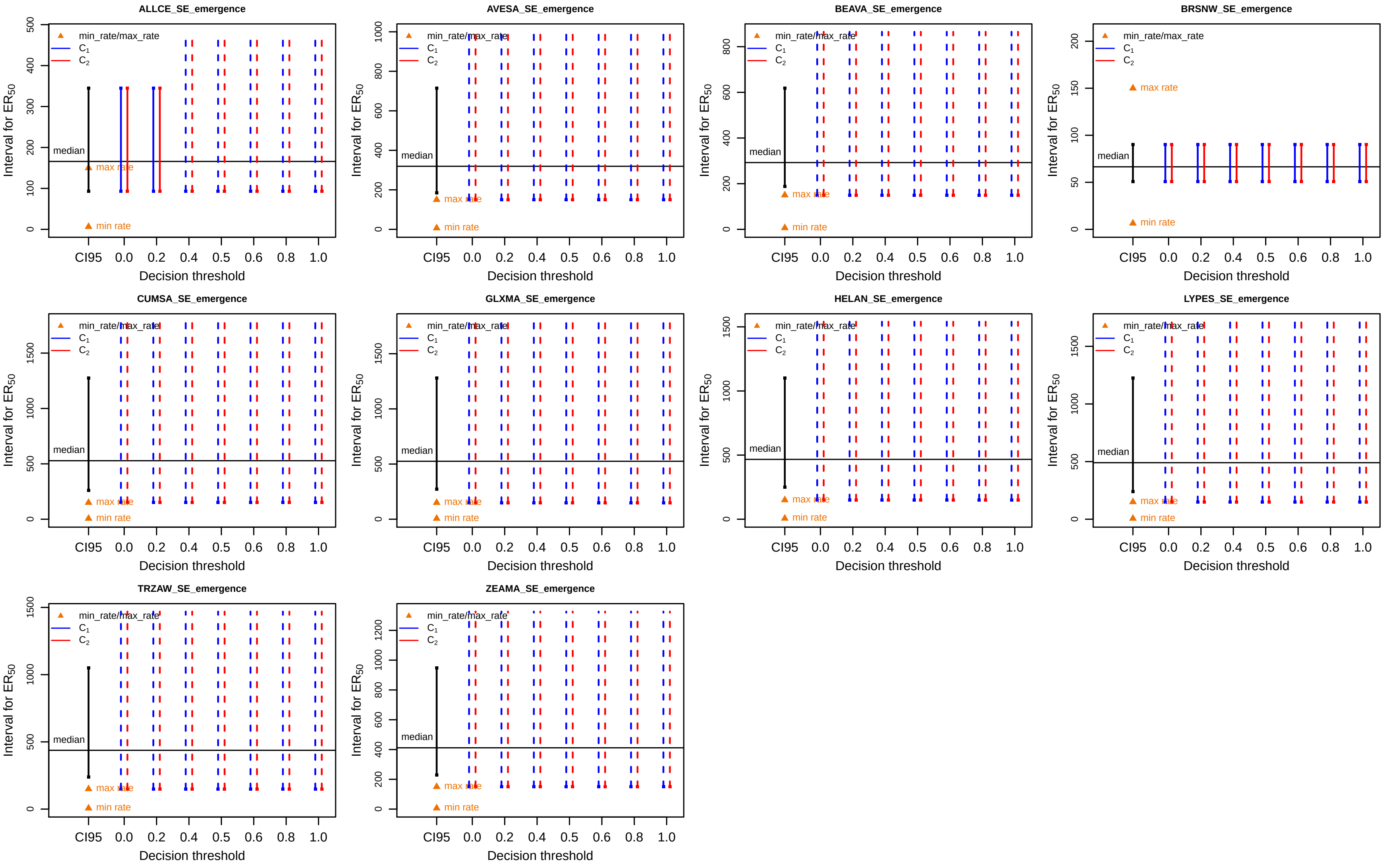

Figure 1: CI95 of ER_{50} and censored ER_{50} according to both criteria and the seven decision thresholds for all species - emergence endpoint of the SE test of study 6. The two orange triangles stand for the lowest and the highest tested rates. Vertical segments of three different colors (black, blue, red) represent the CI95 of ER_{50}, the censored ER_{50} according to C_1 and the censored ER_{50} according to C_2, respectively; solid vertical segments are for bounded intervals while dotted vertical segments stand for right-unbounded intervals; the black horizontal line represents the median of the ER_{50}.

SE test - survival endpoint

Table 6: Minimum and maximum tested rates, median, quantile 2.5% and quantile 97.5% of the ER_{50} posterior distribution - survival endpoint of the SE test of study 6

species	min_rate	max_rate	ER_{50}_median	ER_{50}_LCI95	ER_{50}_UCI95
ALLCE	6.4	150	20.21929	15.06547	27.46527
AVESA	6.4	150	351.25424	211.44586	779.35065
BEAVA	6.4	150	32.85584	26.91823	40.25397
BRSNW	6.4	150	84.73088	61.44102	138.35590
CUMSA	6.4	150	215.78732	160.72380	482.99716
GLXMA	6.4	150	213.80089	160.77484	459.94825
HELAN	6.4	150	130.69007	104.18586	174.42724
LYPES	6.4	150	181.07821	136.44826	324.49977
TRZAW	6.4	150	214.50084	160.70502	479.16101
ZEAMA	6.4	150	215.30855	160.31237	472.46807

Table 7: Censored ER_{50} according to criterion 1 and the seven decision thresholds - survival endpoint of the SE test of study 6

species	C1T0.0	C1T0.2	C1T0.4	C1T0.5	C1T0.6	C1T0.8	C1T1.0
ALLCE	[15.07; 27.47]	[15.07; 27.47]	[15.07; 27.47]	[15.07; 27.47]	[15.07; 27.47]	[15.07; 27.47]	[15.07; 27.47]
AVESA	[150; Inf)	[150; Inf)	[150; Inf)	[150; Inf)	[150; Inf)	[150; Inf)	[150; Inf)
BEAVA	[26.92; 40.25]	[26.92; 40.25]	[26.92; 40.25]	[26.92; 40.25]	[26.92; 40.25]	[26.92; 40.25]	[26.92; 40.25]
BRSNW	[61.44; 138.36]	[61.44; 138.36]	[61.44; 138.36]	[61.44; 138.36]	[61.44; 138.36]	[61.44; 138.36]	[61.44; 138.36]
CUMSA	[150; Inf)	[150; Inf)	[150; Inf)	[150; Inf)	[150; Inf)	[150; Inf)	[150; Inf)
GLXMA	[150; Inf)	[150; Inf)	[150; Inf)	[150; Inf)	[150; Inf)	[150; Inf)	[150; Inf)
HELAN	[104.19; 174.43]	[104.19; 174.43]	[104.19; 174.43]	[104.19; 174.43]	[104.19; 174.43]	[104.19; 174.43]	[104.19; Inf)
LYPES	[136.45; 324.5]	[136.45; Inf)	[136.45; Inf)	[136.45; Inf)	[136.45; Inf)	[136.45; Inf)	[136.45; Inf)
TRZAW	[150; Inf)	[150; Inf)	[150; Inf)	[150; Inf)	[150; Inf)	[150; Inf)	[150; Inf)
ZEAMA	[150; Inf)	[150; Inf)	[150; Inf)	[150; Inf)	[150; Inf)	[150; Inf)	[150; Inf)

Table 8: Censored ER_{50} according to criterion 2 and the seven decision thresholds - survival endpoint of the SE test of study 6

species	C2T0.0	C2T0.2	C2T0.4	C2T0.5	C2T0.6	C2T0.8	C2T1.0
ALLCE	[15.07; 27.47]	[15.07; 27.47]	[15.07; 27.47]	[15.07; 27.47]	[15.07; 27.47]	[15.07; 27.47]	[15.07; 27.47]
AVESA	[150; Inf)	[150; Inf)	[150; Inf)	[150; Inf)	[150; Inf)	[150; Inf)	[150; Inf)
BEAVA	[26.92; 40.25]	[26.92; 40.25]	[26.92; 40.25]	[26.92; 40.25]	[26.92; 40.25]	[26.92; 40.25]	[26.92; 40.25]
BRSNW	[61.44; 138.36]	[61.44; 138.36]	[61.44; 138.36]	[61.44; 138.36]	[61.44; 138.36]	[61.44; 138.36]	[61.44; 138.36]
CUMSA	[150; Inf)	[150; Inf)	[150; Inf)	[150; Inf)	[150; Inf)	[150; Inf)	[150; Inf)
GLXMA	[150; Inf)	[150; Inf)	[150; Inf)	[150; Inf)	[150; Inf)	[150; Inf)	[150; Inf)
HELAN	[104.19; 174.43]	[104.19; 174.43]	[104.19; 174.43]	[104.19; 174.43]	[104.19; 174.43]	[104.19; Inf)	[104.19; Inf)
LYPES	[136.45; 324.5]	[136.45; Inf)	[136.45; Inf)	[136.45; Inf)	[136.45; Inf)	[136.45; Inf)	[136.45; Inf)
TRZAW	[150; Inf)	[150; Inf)	[150; Inf)	[150; Inf)	[150; Inf)	[150; Inf)	[150; Inf)
ZEAMA	[150; Inf)	[150; Inf)	[150; Inf)	[150; Inf)	[150; Inf)	[150; Inf)	[150; Inf)

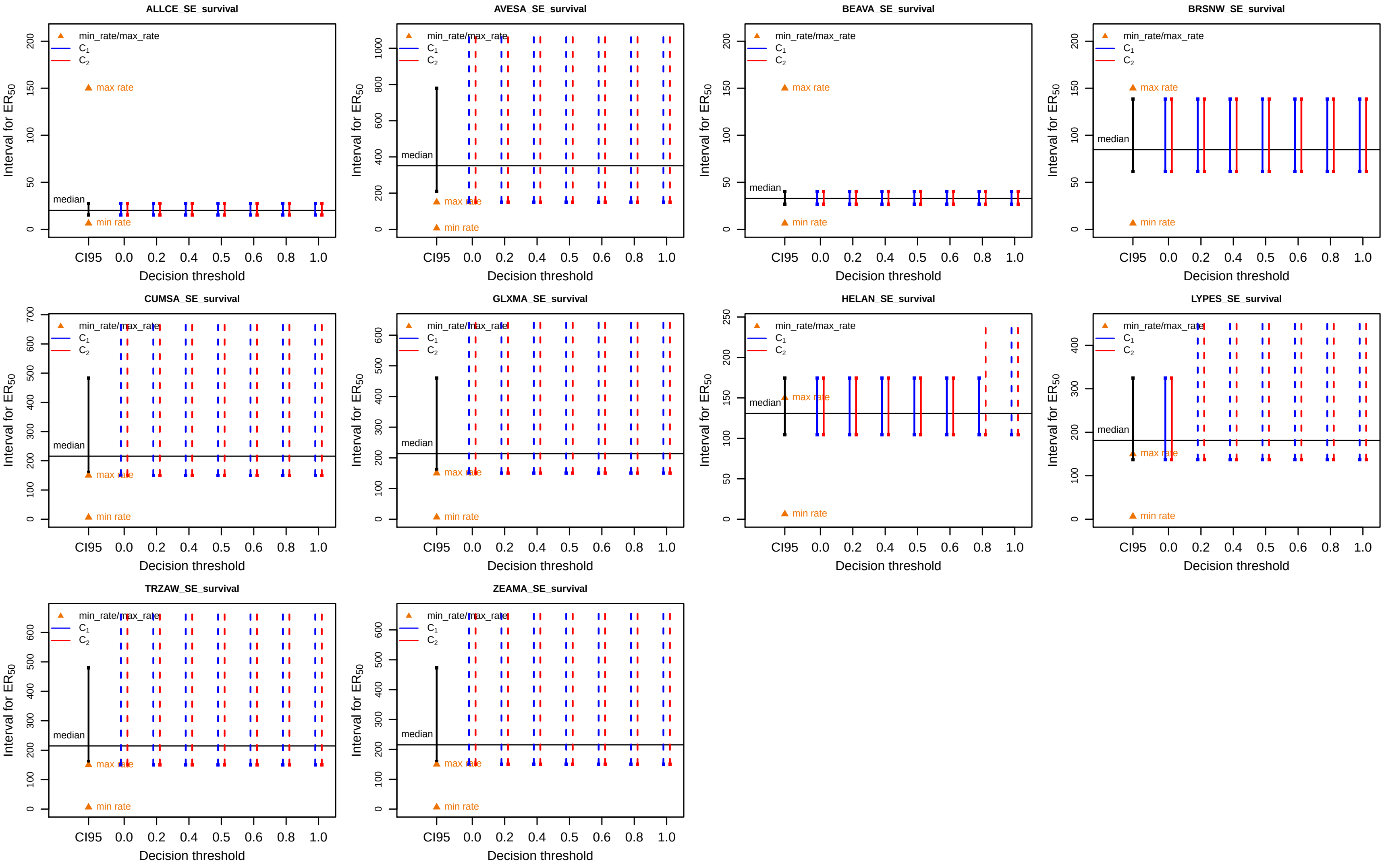

Figure 2: CI95 of ER_{50} and censored ER_{50} according to both criteria and the seven decision thresholds for all species - survival endpoint of the SE test of study 6. The two orange triangles stand for the lowest and the highest tested rates. Vertical segments of three different colors (black, blue, red) represent the CI95 of ER_{50}, the censored ER_{50} according to C_1 and the censored ER_{50} according to C_2, respectively; solid vertical segments are for bounded intervals while dotted vertical segments stand for right-unbounded intervals; the black horizontal line represents the median of the ER_{50}.

SE test - shoot dry weight endpoint

Table 9: Minimum and maximum tested rates, median, quantile 2.5% and quantile 97.5% of the ER_{50} posterior distribution - shoot dry weight endpoint of the SE test of study 6

species	min_rate	max_rate	ER_{50}_median	ER_{50}_LCI95	ER_{50}_UCI95
ALLCE	6.4	31.0	12.04560	7.699122	19.23066
AVESA	6.4	150.0	25.20738	21.097805	29.87952
BEAVA	6.4	68.2	11.05411	9.636836	12.72220
BRSNW	6.4	68.2	21.52147	16.760696	28.25219
CUMSA	6.4	150.0	28.64279	20.359200	40.75775
GLXMA	6.4	150.0	145.28995	121.452865	176.90209
HELAN	6.4	150.0	48.60438	38.692419	60.36431
LYPES	6.4	150.0	21.31697	18.196236	25.11975
TRZAW	6.4	150.0	167.74420	138.635252	229.05563
ZEAMA	6.4	150.0	194.49779	153.444603	300.68310

Table 10: Censored ER_{50} according to criterion 1 and the seven decision thresholds - shoot dry weight endpoint of the SE test of study 6

species	C1T0.0	C1T0.2	C1T0.4	C1T0.5	C1T0.6	C1T0.8	C1T1.0
ALLCE	[7.7; 19.23]	[7.7; 19.23]	[7.7; 19.23]	[7.7; 19.23]	[7.7; 19.23]	[7.7; 19.23]	[7.7; 19.23]
AVESA	[21.1; 29.88]	[21.1; 29.88]	[21.1; 29.88]	[21.1; 29.88]	[21.1; 29.88]	[21.1; 29.88]	[21.1; 29.88]
BEAVA	[9.64; 12.72]	[9.64; 12.72]	[9.64; 12.72]	[9.64; 12.72]	[9.64; 12.72]	[9.64; 12.72]	[9.64; 12.72]
BRSNW	[16.76; 28.25]	[16.76; 28.25]	[16.76; 28.25]	[16.76; 28.25]	[16.76; 28.25]	[16.76; 28.25]	[16.76; 28.25]
CUMSA	[20.36; 40.76]	[20.36; 40.76]	[20.36; 40.76]	[20.36; 40.76]	[20.36; 40.76]	[20.36; 40.76]	[20.36; 40.76]
GLXMA	[121.45; 176.9]	[121.45; 176.9]	[121.45; 176.9]	[121.45; 176.9]	[121.45; 176.9]	[121.45; Inf)	[121.45; Inf)
HELAN	[38.69; 60.36]	[38.69; 60.36]	[38.69; 60.36]	[38.69; 60.36]	[38.69; 60.36]	[38.69; 60.36]	[38.69; 60.36]
LYPES	[18.2; 25.12]	[18.2; 25.12]	[18.2; 25.12]	[18.2; 25.12]	[18.2; 25.12]	[18.2; 25.12]	[18.2; 25.12]
TRZAW	[138.64; 229.06]	[138.64; Inf)	[138.64; Inf)	[138.64; Inf)	[138.64; Inf)	[138.64; Inf)	[138.64; Inf)
ZEAMA	[150; Inf)	[150; Inf)	[150; Inf)	[150; Inf)	[150; Inf)	[150; Inf)	[150; Inf)

Table 11: Censored ER_{50} according to criterion 2 and the seven decision thresholds - shoot dry weight endpoint of the SE test of study 6

species	C2T0.0	C2T0.2	C2T0.4	C2T0.5	C2T0.6	C2T0.8	C2T1.0
ALLCE	[7.7; 19.23]	[7.7; 19.23]	[7.7; 19.23]	[7.7; 19.23]	[7.7; 19.23]	[7.7; 19.23]	[7.7; 19.23]
AVESA	[21.1; 29.88]	[21.1; 29.88]	[21.1; 29.88]	[21.1; 29.88]	[21.1; 29.88]	[21.1; 29.88]	[21.1; 29.88]
BEAVA	[9.64; 12.72]	[9.64; 12.72]	[9.64; 12.72]	[9.64; 12.72]	[9.64; 12.72]	[9.64; 12.72]	[9.64; 12.72]
BRSNW	[16.76; 28.25]	[16.76; 28.25]	[16.76; 28.25]	[16.76; 28.25]	[16.76; 28.25]	[16.76; 28.25]	[16.76; 28.25]
CUMSA	[20.36; 40.76]	[20.36; 40.76]	[20.36; 40.76]	[20.36; 40.76]	[20.36; 40.76]	[20.36; 40.76]	[20.36; 40.76]
GLXMA	[121.45; 176.9]	[121.45; 176.9]	[121.45; 176.9]	[121.45; 176.9]	[121.45; Inf)	[121.45; Inf)	[121.45; Inf)
HELAN	[38.69; 60.36]	[38.69; 60.36]	[38.69; 60.36]	[38.69; 60.36]	[38.69; 60.36]	[38.69; 60.36]	[38.69; 60.36]
LYPES	[18.2; 25.12]	[18.2; 25.12]	[18.2; 25.12]	[18.2; 25.12]	[18.2; 25.12]	[18.2; 25.12]	[18.2; 25.12]
TRZAW	[138.64; 229.06]	[138.64; Inf)	[138.64; Inf)	[138.64; Inf)	[138.64; Inf)	[138.64; Inf)	[138.64; Inf)
ZEAMA	[150; Inf)	[150; Inf)	[150; Inf)	[150; Inf)	[150; Inf)	[150; Inf)	[150; Inf)

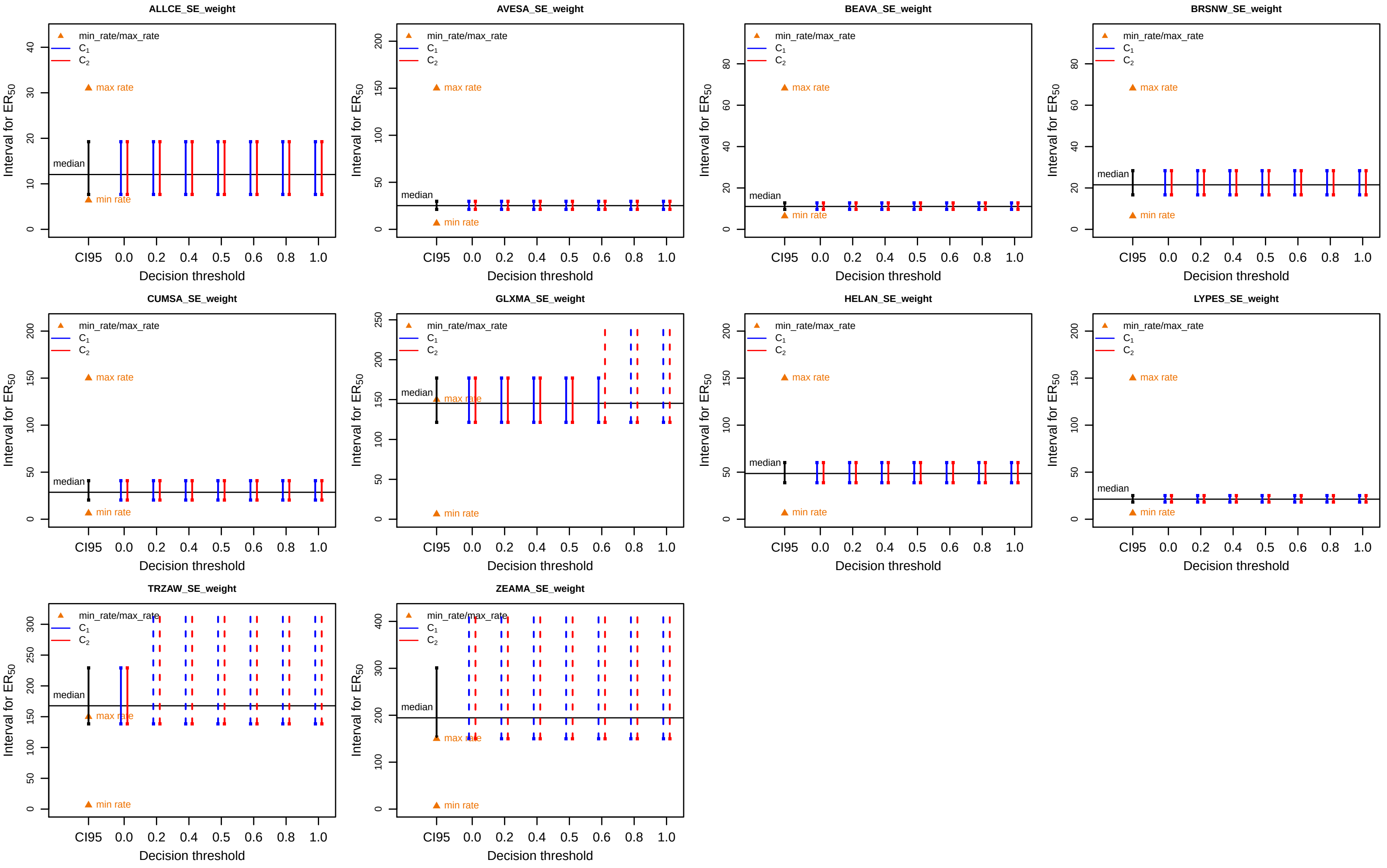

Figure 3: CI95 of ER_{50} and censored ER_{50} according to both criteria and the seven decision thresholds for all species - shoot dry weight endpoint of the SE test of study 6. The two orange triangles stand for the lowest and the highest tested rates. Vertical segments of three different colors (black, blue, red) represent the CI95 of ER_{50}, the censored ER_{50} according to C_1 and the censored ER_{50} according to C_2, respectively; solid vertical segments are for bounded intervals while dotted vertical segments stand for right-unbounded intervals; the black horizontal line represents the median of the ER_{50}.

Vegatative vigor (VV) test of study 6

Raw data description (test item rates) for VV test

```
Number of species:  10
EPPO code:  ALLCE AVESA BEAVA BRSNW CUMSA GLXMA HELAN LYPES TRZAW ZEAMA
```

Table 12: Corresponding species and common names to the EPPO code

EPPO code	Species name	Common name
ALLCE	*Allium cepa*	Onion
AVESA	*Avena sativa*	Oat
BEAVA	*Beta vulgaris*	Sugar beet
BRSNW	*Brassica napus winter*	Oilseed rape
CUMSA	*Cucumis sativus*	Cucumber
GLXMA	*Glycine max*	Soybean
HELAN	*Helianthus annuus*	Sunflower
LYPES	*Solanum lycopersicum*	Tomato
TRZAW	*Triticum aestivum winter*	Winter wheat
ZEAMA	*Zea mays subs. Amylacea*	Corn

Table 13: Tested rates per species (g product/ha) for the VV test of study 6

ALLCE	AVESA	BEAVA	BRSNW	CUMSA	GLXMA	HELAN	LYPES	TRZAW	ZEAMA
0.0	0.0	0.0	0.0	0.0	0.0	0.0	0.0	0.0	0.0
2.9	14.1	2.9	2.9	2.9	2.9	2.9	2.9	14.1	14.1
6.4	31.0	6.4	6.4	6.4	6.4	6.4	6.4	31.0	31.0
14.1	68.2	14.1	14.1	14.1	14.1	14.1	14.1	68.2	68.2
31.0	150.0	31.0	31.0	31.0	31.0	31.0	31.0	150.0	150.0
68.2	330.0	68.2	68.2	68.2	68.2	68.2	68.2	330.0	330.0

VV test - survival endpoint

Table 14: Minimum and maximum tested rates, median, quantile 2.5% and quantile 97.5% of the ER_{50} posterior distribution - survival endpoint of the VV test of study 6

species	min_rate	max_rate	ER_{50}_median	ER_{50}_LCI95	ER_{50}_UCI95
ALLCE	2.9	68.2	81.65770	69.73608	145.76681
AVESA	14.1	330.0	47.28865	34.01723	62.89949
BEAVA	2.9	68.2	62.62223	47.83854	92.69804
BRSNW	2.9	68.2	45.34150	37.80220	54.41266
CUMSA	2.9	68.2	98.26838	73.16119	214.72208
GLXMA	2.9	68.2	97.59306	72.70578	208.11535
HELAN	2.9	68.2	24.96320	19.36288	29.76643
LYPES	2.9	68.2	86.74461	70.69633	178.58910
TRZAW	14.1	330.0	417.56250	342.23371	858.39988
ZEAMA	14.1	330.0	384.80181	335.02835	664.40402

Table 15: Censored ER_{50} according to criterion 1 and the seven decision thresholds - survival endpoint of the VV test of study 6

species	C1T0.0	C1T0.2	C1T0.4	C1T0.5	C1T0.6	C1T0.8	C1T1.0
ALLCE	[68.2; Inf)	[68.2; Inf)	[68.2; Inf)	[68.2; Inf)	[68.2; Inf)	[68.2; Inf)	[68.2; Inf)
AVESA	[34.02; 62.9]	[34.02; 62.9]	[34.02; 62.9]	[34.02; 62.9]	[34.02; 62.9]	[34.02; 62.9]	[34.02; 62.9]
BEAVA	[47.84; 92.7]	[47.84; 92.7]	[47.84; 92.7]	[47.84; 92.7]	[47.84; 92.7]	[47.84; Inf)	[47.84; Inf)
BRSNW	[37.8; 54.41]	[37.8; 54.41]	[37.8; 54.41]	[37.8; 54.41]	[37.8; 54.41]	[37.8; 54.41]	[37.8; 54.41]
CUMSA	[68.2; Inf)	[68.2; Inf)	[68.2; Inf)	[68.2; Inf)	[68.2; Inf)	[68.2; Inf)	[68.2; Inf)
GLXMA	[68.2; Inf)	[68.2; Inf)	[68.2; Inf)	[68.2; Inf)	[68.2; Inf)	[68.2; Inf)	[68.2; Inf)
HELAN	[19.36; 29.77]	[19.36; 29.77]	[19.36; 29.77]	[19.36; 29.77]	[19.36; 29.77]	[19.36; 29.77]	[19.36; 29.77]
LYPES	[68.2; Inf)	[68.2; Inf)	[68.2; Inf)	[68.2; Inf)	[68.2; Inf)	[68.2; Inf)	[68.2; Inf)
TRZAW	[330; Inf)	[330; Inf)	[330; Inf)	[330; Inf)	[330; Inf)	[330; Inf)	[330; Inf)
ZEAMA	[330; Inf)	[330; Inf)	[330; Inf)	[330; Inf)	[330; Inf)	[330; Inf)	[330; Inf)

Table 16: Censored ER_{50} according to criterion 2 and the seven decision thresholds - survival endpoint of the VV test of study 6

species	C2T0.0	C2T0.2	C2T0.4	C2T0.5	C2T0.6	C2T0.8	C2T1.0
ALLCE	[68.2; Inf)	[68.2; Inf)	[68.2; Inf)	[68.2; Inf)	[68.2; Inf)	[68.2; Inf)	[68.2; Inf)
AVESA	[34.02; 62.9]	[34.02; 62.9]	[34.02; 62.9]	[34.02; 62.9]	[34.02; 62.9]	[34.02; 62.9]	[34.02; 62.9]
BEAVA	[47.84; 92.7]	[47.84; 92.7]	[47.84; 92.7]	[47.84; Inf)	[47.84; Inf)	[47.84; Inf)	[47.84; Inf)
BRSNW	[37.8; 54.41]	[37.8; 54.41]	[37.8; 54.41]	[37.8; 54.41]	[37.8; 54.41]	[37.8; 54.41]	[37.8; 54.41]
CUMSA	[68.2; Inf)	[68.2; Inf)	[68.2; Inf)	[68.2; Inf)	[68.2; Inf)	[68.2; Inf)	[68.2; Inf)
GLXMA	[68.2; Inf)	[68.2; Inf)	[68.2; Inf)	[68.2; Inf)	[68.2; Inf)	[68.2; Inf)	[68.2; Inf)
HELAN	[19.36; 29.77]	[19.36; 29.77]	[19.36; 29.77]	[19.36; 29.77]	[19.36; 29.77]	[19.36; 29.77]	[19.36; 29.77]
LYPES	[68.2; Inf)	[68.2; Inf)	[68.2; Inf)	[68.2; Inf)	[68.2; Inf)	[68.2; Inf)	[68.2; Inf)
TRZAW	[330; Inf)	[330; Inf)	[330; Inf)	[330; Inf)	[330; Inf)	[330; Inf)	[330; Inf)
ZEAMA	[330; Inf)	[330; Inf)	[330; Inf)	[330; Inf)	[330; Inf)	[330; Inf)	[330; Inf)

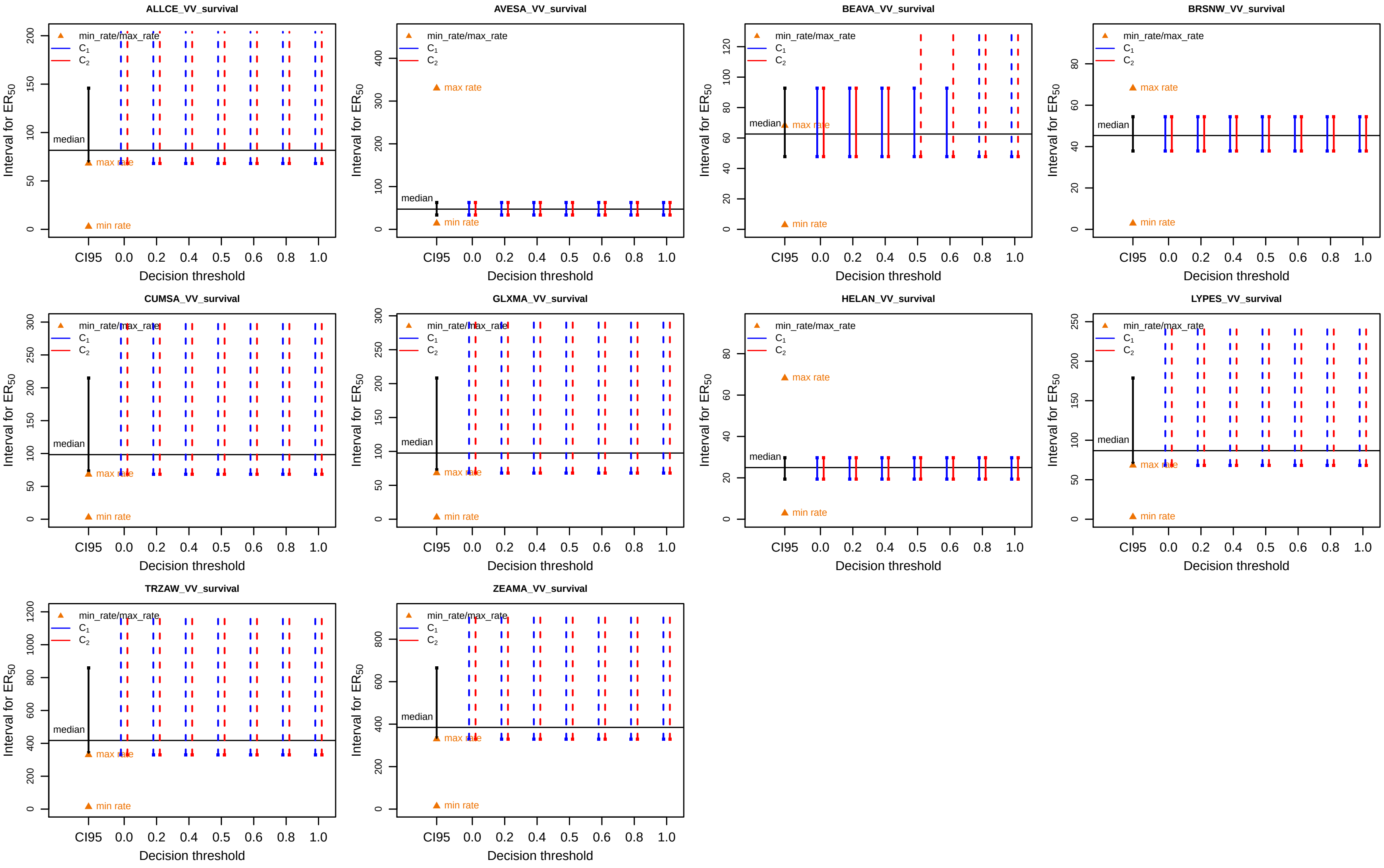

Figure 4: CI95 of ER_{50} and censored ER_{50} according to both criteria and the seven decision thresholds for all species - survival endpoint of the VV test of study 6. The two orange triangles stand for the lowest and the highest tested rates. Vertical segments of three different colors (black, blue, red) represent the CI95 of ER_{50}, the censored ER_{50} according to C_1 and the censored ER_{50} according to C_2, respectively; solid vertical segments are for bounded intervals while dotted vertical segments stand for right-unbounded intervals; the black horizontal line represents the median of the ER_{50}.

VV test - shoot dry weight endpoint

Table 17: Minimum and maximum tested rates, median, quantile 2.5% and quantile 97.5% of the ER_{50} posterior distribution - shoot dry weight endpoint of the VV test of study 6

species	min_rate	max_rate	ER_{50}_median	ER_{50}_LCI95	ER_{50}_UCI95
ALLCE	2.9	68.2	53.199528	43.458650	65.797358
AVESA	14.1	31.0	31.970554	26.372781	37.222474
BEAVA	2.9	68.2	7.201706	5.817101	9.056635
BRSNW	2.9	68.2	8.772378	6.973150	11.149813
CUMSA	2.9	68.2	14.146864	11.162789	18.087363
GLXMA	2.9	68.2	14.248683	12.357617	16.367365
HELAN	2.9	31.0	12.793299	11.728779	13.931216
LYPES	2.9	68.2	10.757460	9.141098	12.670217
TRZAW	14.1	330.0	248.881842	219.795063	281.520375
ZEAMA	14.1	330.0	213.231472	189.844121	240.395827

Table 18: Censored ER_{50} according to criterion 1 and the seven decision thresholds - shoot dry weight endpoint of the VV test of study 6

species	C1T0.0	C1T0.2	C1T0.4	C1T0.5	C1T0.6	C1T0.8	C1T1.0
ALLCE	[43.46; 65.8]	[43.46; 65.8]	[43.46; 65.8]	[43.46; 65.8]	[43.46; 65.8]	[43.46; 65.8]	[43.46; 65.8]
AVESA	[26.37; 37.22]	[26.37; 37.22]	[26.37; Inf)	[26.37; Inf)	[26.37; Inf)	[26.37; Inf)	[26.37; Inf)
BEAVA	[5.82; 9.06]	[5.82; 9.06]	[5.82; 9.06]	[5.82; 9.06]	[5.82; 9.06]	[5.82; 9.06]	[5.82; 9.06]
BRSNW	[6.97; 11.15]	[6.97; 11.15]	[6.97; 11.15]	[6.97; 11.15]	[6.97; 11.15]	[6.97; 11.15]	[6.97; 11.15]
CUMSA	[11.16; 18.09]	[11.16; 18.09]	[11.16; 18.09]	[11.16; 18.09]	[11.16; 18.09]	[11.16; 18.09]	[11.16; 18.09]
GLXMA	[12.36; 16.37]	[12.36; 16.37]	[12.36; 16.37]	[12.36; 16.37]	[12.36; 16.37]	[12.36; 16.37]	[12.36; 16.37]
HELAN	[11.73; 13.93]	[11.73; 13.93]	[11.73; 13.93]	[11.73; 13.93]	[11.73; 13.93]	[11.73; 13.93]	[11.73; 13.93]
LYPES	[9.14; 12.67]	[9.14; 12.67]	[9.14; 12.67]	[9.14; 12.67]	[9.14; 12.67]	[9.14; 12.67]	[9.14; 12.67]
TRZAW	[219.8; 281.52]	[219.8; 281.52]	[219.8; 281.52]	[219.8; 281.52]	[219.8; 281.52]	[219.8; 281.52]	[219.8; 281.52]
ZEAMA	[189.84; 240.4]	[189.84; 240.4]	[189.84; 240.4]	[189.84; 240.4]	[189.84; 240.4]	[189.84; 240.4]	[189.84; 240.4]

Table 19: Censored ER_{50} according to criterion 2 and the seven decision thresholds - shoot dry weight endpoint of the VV test of study 6

species	C2T0.0	C2T0.2	C2T0.4	C2T0.5	C2T0.6	C2T0.8	C2T1.0
ALLCE	[43.46; 65.8]	[43.46; 65.8]	[43.46; 65.8]	[43.46; 65.8]	[43.46; 65.8]	[43.46; 65.8]	[43.46; 65.8]
AVESA	[26.37; 37.22]	[26.37; 37.22]	[26.37; 37.22]	[26.37; Inf)	[26.37; Inf)	[26.37; Inf)	[26.37; Inf)
BEAVA	[5.82; 9.06]	[5.82; 9.06]	[5.82; 9.06]	[5.82; 9.06]	[5.82; 9.06]	[5.82; 9.06]	[5.82; 9.06]
BRSNW	[6.97; 11.15]	[6.97; 11.15]	[6.97; 11.15]	[6.97; 11.15]	[6.97; 11.15]	[6.97; 11.15]	[6.97; 11.15]
CUMSA	[11.16; 18.09]	[11.16; 18.09]	[11.16; 18.09]	[11.16; 18.09]	[11.16; 18.09]	[11.16; 18.09]	[11.16; 18.09]
GLXMA	[12.36; 16.37]	[12.36; 16.37]	[12.36; 16.37]	[12.36; 16.37]	[12.36; 16.37]	[12.36; 16.37]	[12.36; 16.37]
HELAN	[11.73; 13.93]	[11.73; 13.93]	[11.73; 13.93]	[11.73; 13.93]	[11.73; 13.93]	[11.73; 13.93]	[11.73; 13.93]
LYPES	[9.14; 12.67]	[9.14; 12.67]	[9.14; 12.67]	[9.14; 12.67]	[9.14; 12.67]	[9.14; 12.67]	[9.14; 12.67]
TRZAW	[219.8; 281.52]	[219.8; 281.52]	[219.8; 281.52]	[219.8; 281.52]	[219.8; 281.52]	[219.8; 281.52]	[219.8; 281.52]
ZEAMA	[189.84; 240.4]	[189.84; 240.4]	[189.84; 240.4]	[189.84; 240.4]	[189.84; 240.4]	[189.84; 240.4]	[189.84; 240.4]

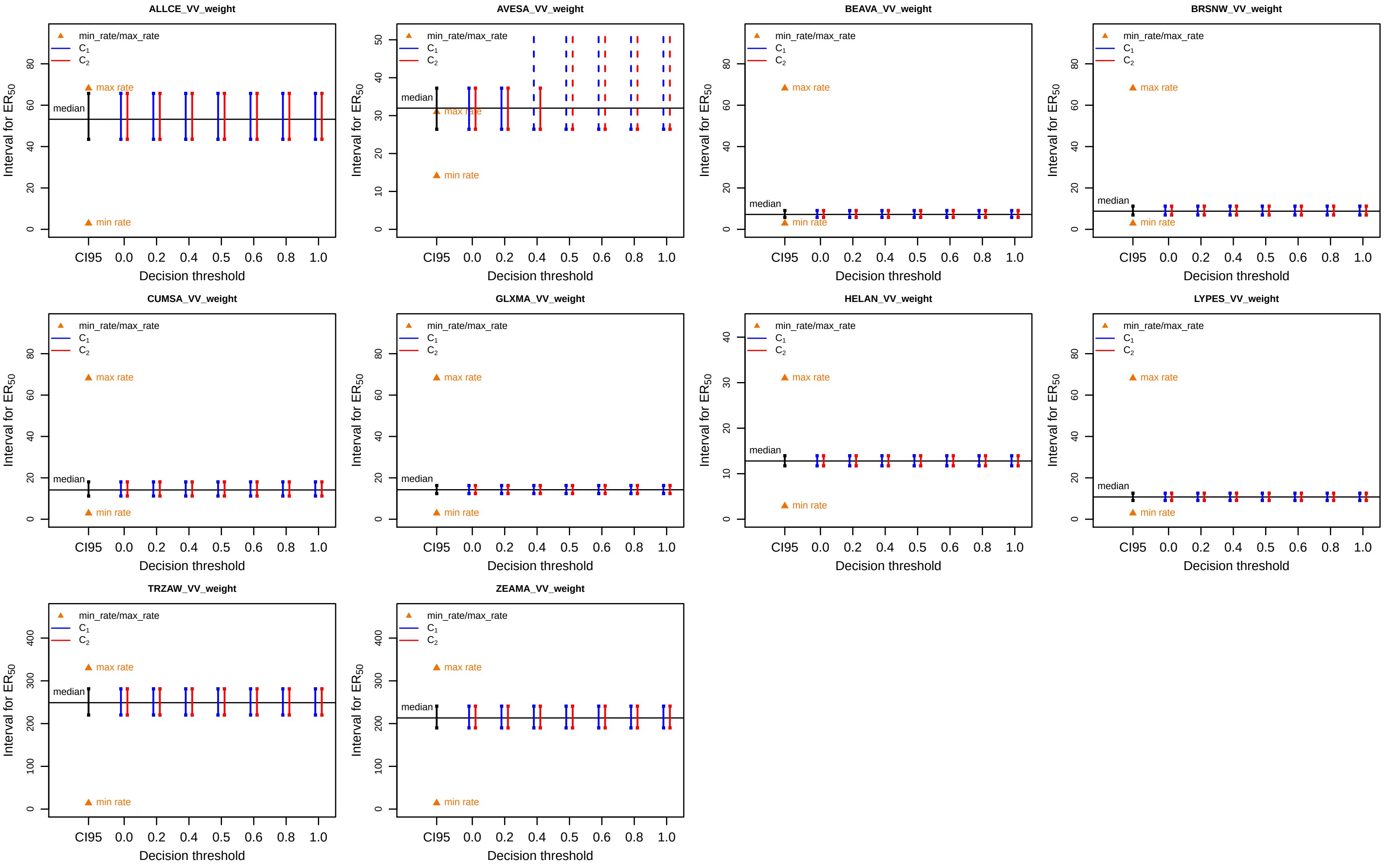

Figure 5: CI95 of ER_{50} and censored ER_{50} according to both criteria and the seven decision thresholds for all species - shoot dry weight endpoint of the VV test of study 6. The two orange triangles stand for the lowest and the highest tested rates. Vertical segments of three different colors (black, blue, red) represent the CI95 of ER_{50}, the censored ER_{50} according to C_1 and the censored ER_{50} according to C_2, respectively; solid vertical segments are for bounded intervals while dotted vertical segments stand for right-unbounded intervals; the black horizontal line represents the median of the ER_{50}.
