# Supplementary material for: How to account for the uncertainty from standard toxicity tests in species sensitivity distributions: An example in non-target plants
Source: PLoS One. 2021 Jan 7;16(1):e0245071. doi: 10.1371/journal.pone.0245071 (PMC7790375; doi:10.1371/journal.pone.0245071)
Supplement: S1 Archive — It is a zip file containing seven folders (one folder per case study). Each folder contains five files report_xxx.pdf with detailed results of the dose-response analyses, one file corresponding to does-response analysis per endpoint. It also contains one file ER50_censoring.pdf for censored ER50 and one file SSD_analyses.pdf for results of SSD analyses. (ZIP) [file pone.0245071.s004.zip › S1_archive/Study6/report_SE_emergence.pdf]

Dose-response analyses

Study 6

Seedling Emergence test - emergence endpoint

25 June 2020

Contact: sandrine.charles@univ-lyon1.fr

This is a report which provides results on all performed dose-response analyses for the emergence endpoint of the Seedling Emergence test for study 6.

Contents

Data set: ALLCE_SE_emergence . . . 2
Data set: AVESA_SE_emergence . . . 3
Data set: BEAVA_SE_emergence . . . 4
Data set: BRSNW_SE_emergence . . . 5
Data set: CUMSA_SE_emergence . . . 6
Data set: GLXMA_SE_emergence . . . 7
Data set: HELAN_SE_emergence . . . 8
Data set: LYPES_SE_emergence . . . 9
Data set: TRZAW_SE_emergence . . . 10
Data set: ZEAMA_SE_emergence . . . 11

Data set: ALLCE_SE_emergence

Table 1: Summary of parameter estimates for ALLCE_SE_emergence data set

Parameter	median	Q2.5	Q97.5
b	1.800	0.581	55.708
d	0.751	0.630	0.877
e	165.846	93.410	344.669

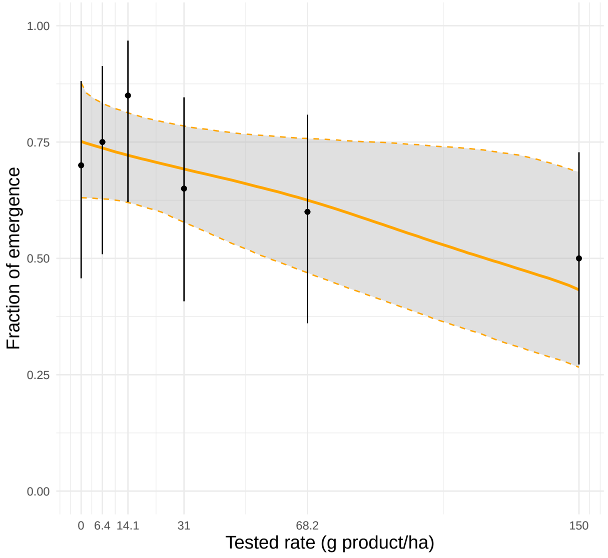

(a) Dose-response curve

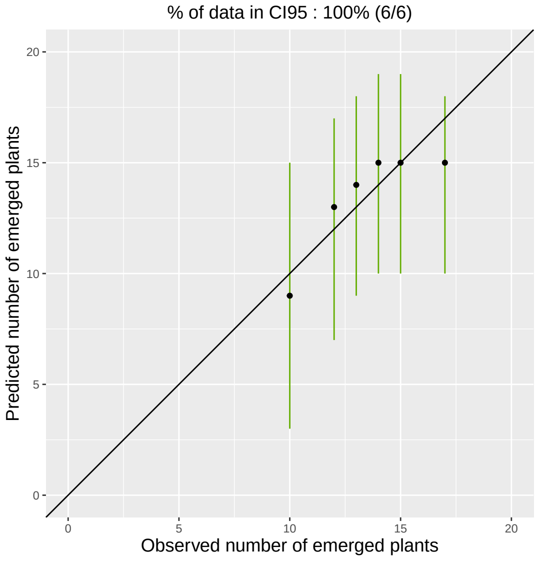

(b) Posterior predictive check (PPC)

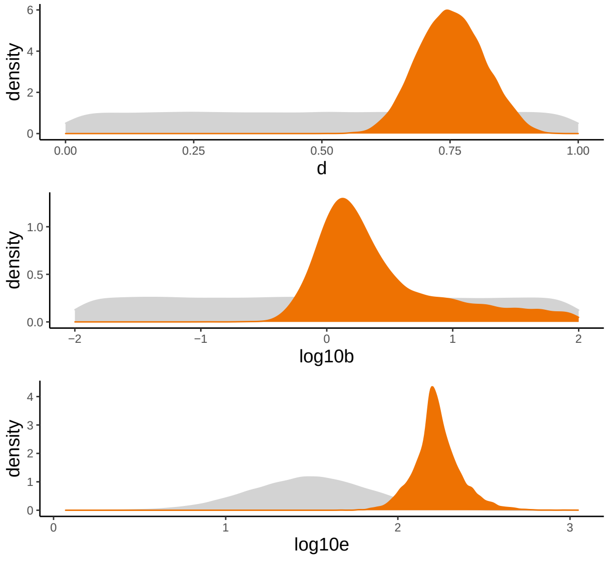

(c) Priors and posteriors

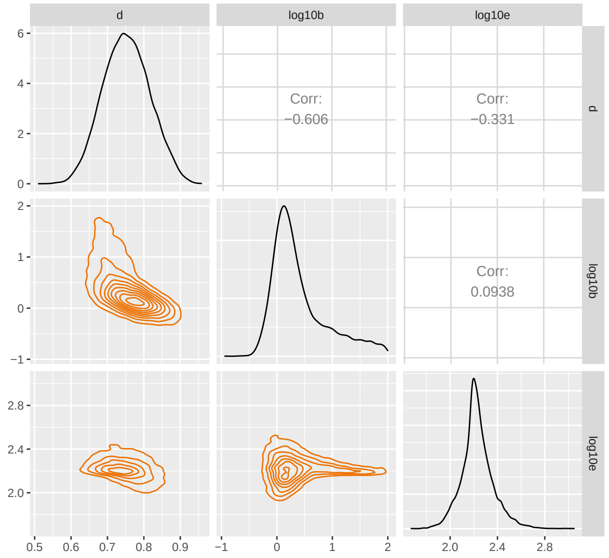

(d) Correlations between parameters

Figure 1: Dose-response curve (a), PPC (b), prior and posterior distributions (c) and correlations between parameters (d).

Data set: AVESA_SE_emergence

Table 2: Summary of parameter estimates (parameter d is set to 1) for AVESA_SE_emergence data set

Parameter	median	Q2.5	Q97.5
b	1.372	0.837	2.226
e	319.155	184.587	714.326

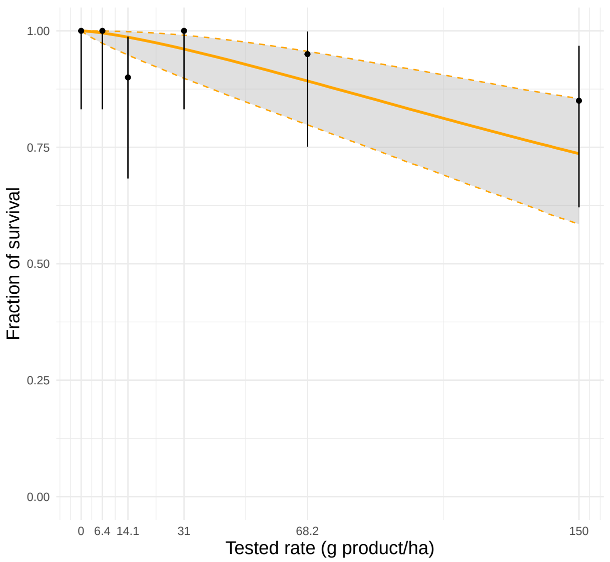

(a) Dose-response curve

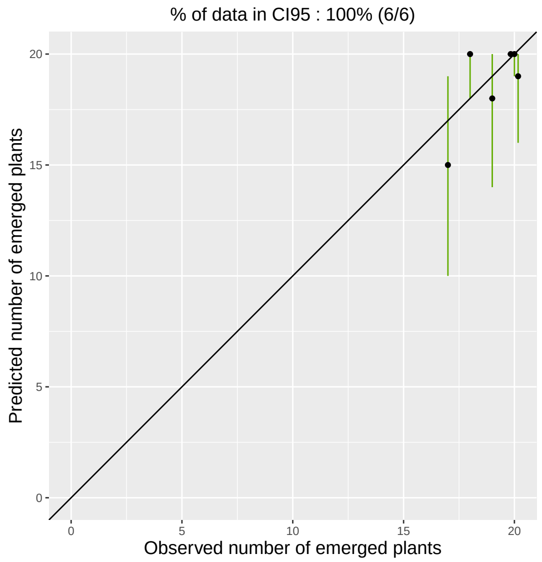

(b) Posterior predictive check (PPC)

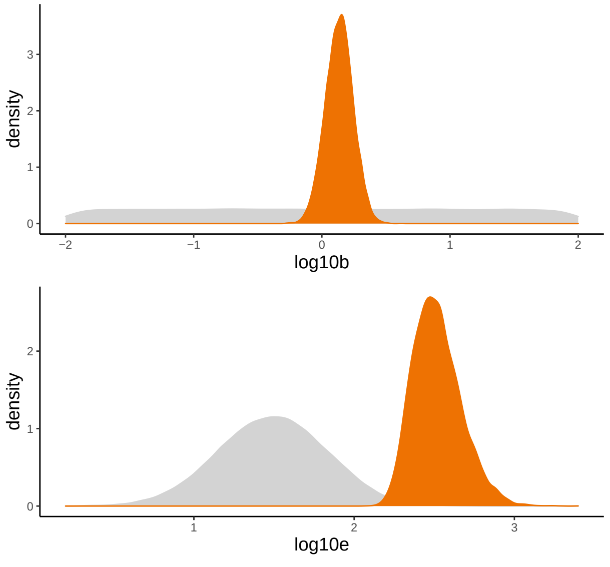

(c) Priors and posteriors

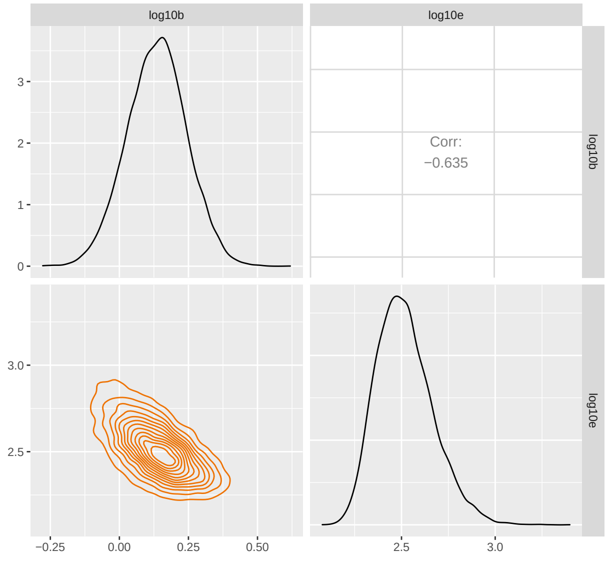

(d) Correlations between parameters

Figure 2: Dose-response curve (a), PPC (b), prior and posterior distributions (c) and correlations between parameters (d).

Data set: BEAVA_SE_emergence

Table 3: Summary of parameter estimates (parameter d is set to 1) for BEAVA_SE_emergence data set

Parameter	median	Q2.5	Q97.5
b	2.282	1.257	4.313
e	292.419	188.077	617.988

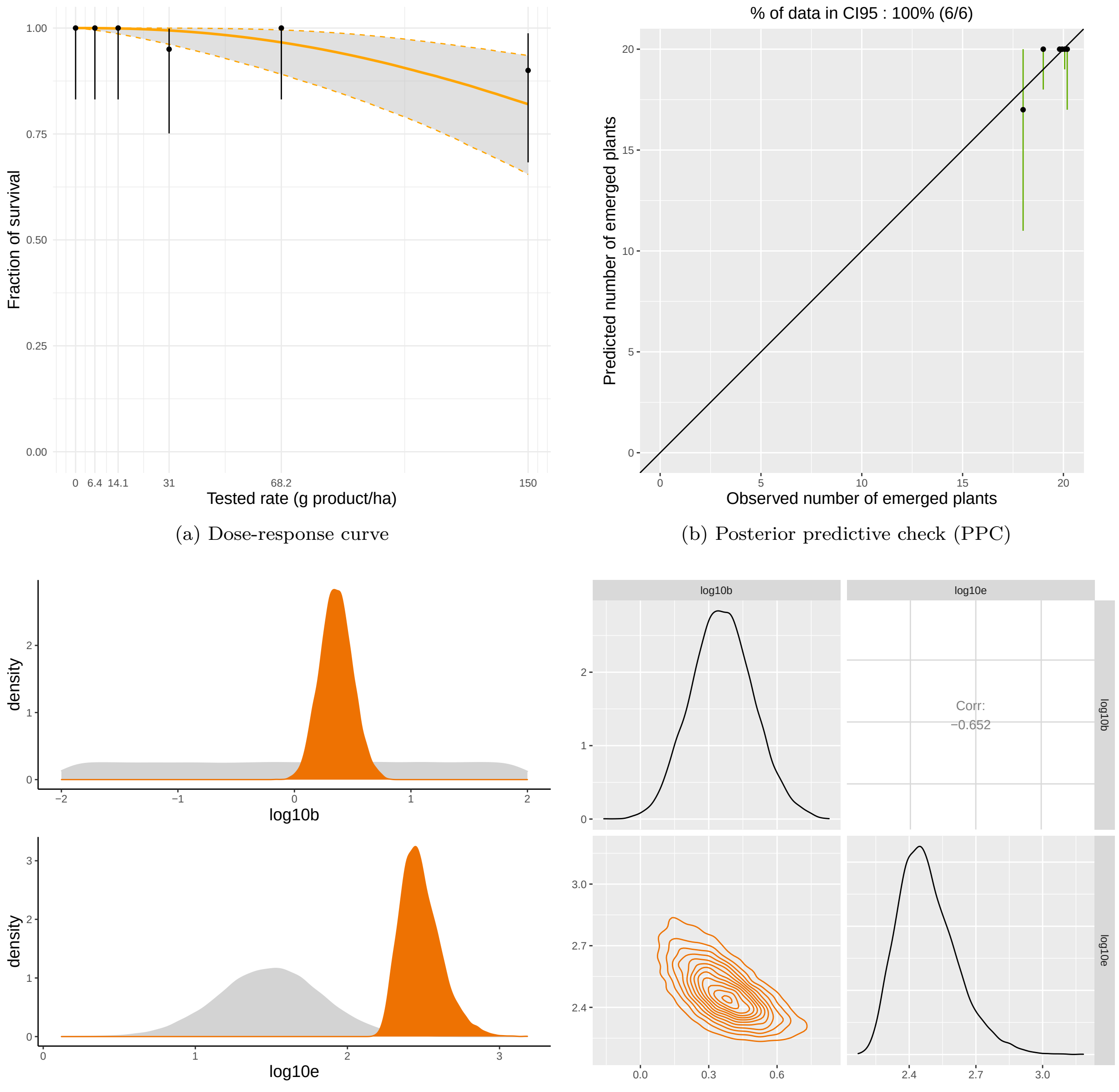

(a) Dose-response curve

(b) Posterior predictive check (PPC)

(c) Priors and posteriors

(d) Correlations between parameters

Figure 3: Dose-response curve (a), PPC (b), prior and posterior distributions (c) and correlations between parameters (d).

Data set: BRSNW_SE_emergence

Table 4: Summary of parameter estimates (parameter d is set to 1) for BRSNW_SE_emergence data set

Parameter	median	Q2.5	Q97.5
b	2.063	1.325	2.999
e	66.484	50.832	90.237

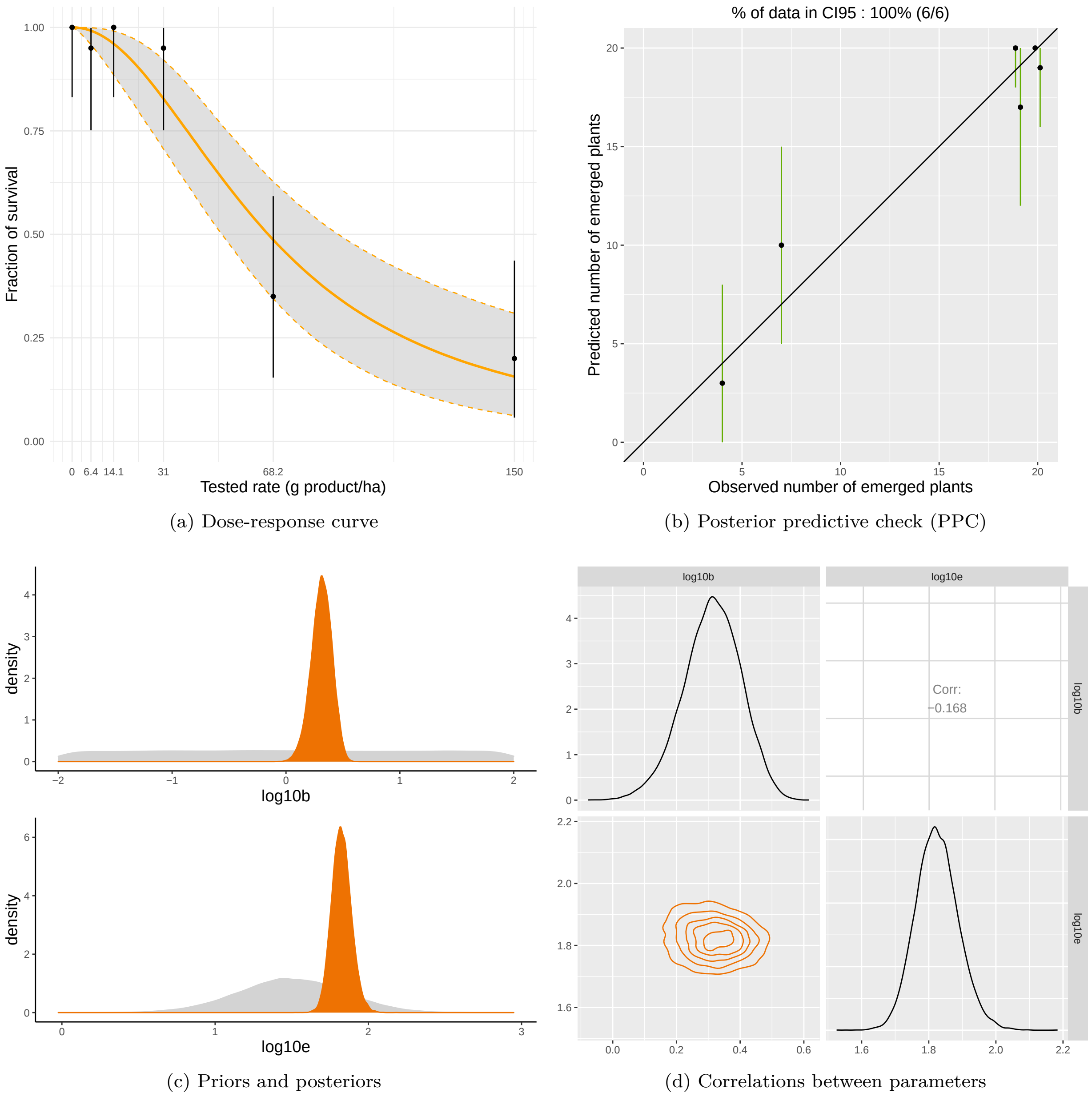

(a) Dose-response curve
(b) Posterior predictive check (PPC)
(c) Priors and posteriors
(d) Correlations between parameters

Figure 4: Dose-response curve (a), PPC (b), prior and posterior distributions (c) and correlations between parameters (d).

Data set: CUMSA_SE_emergence

Table 5: Summary of parameter estimates (parameter d is set to 1) for CUMSA_SE_emergence data set

Parameter	median	Q2.5	Q97.5
b	1.020	0.671	1.520
e	528.097	262.248	1274.037

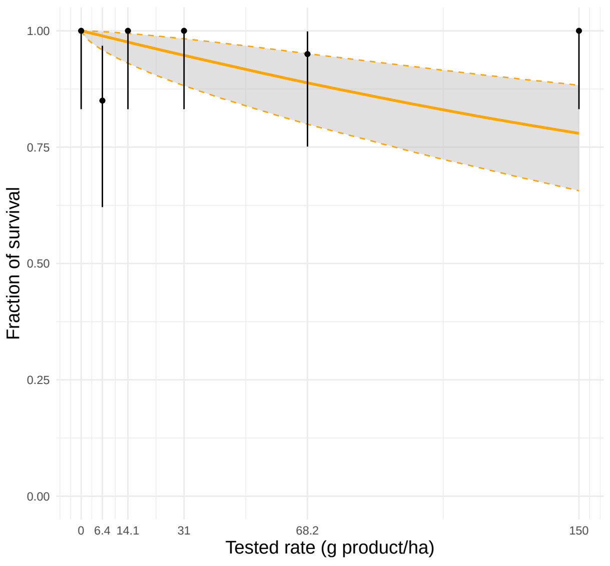

(a) Dose-response curve

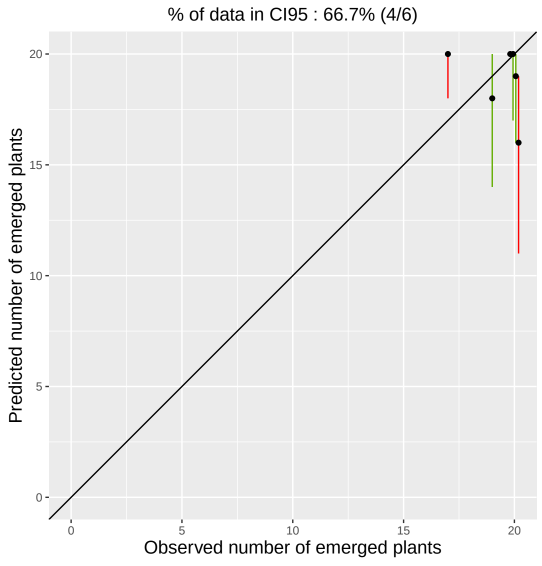

(b) Posterior predictive check (PPC)

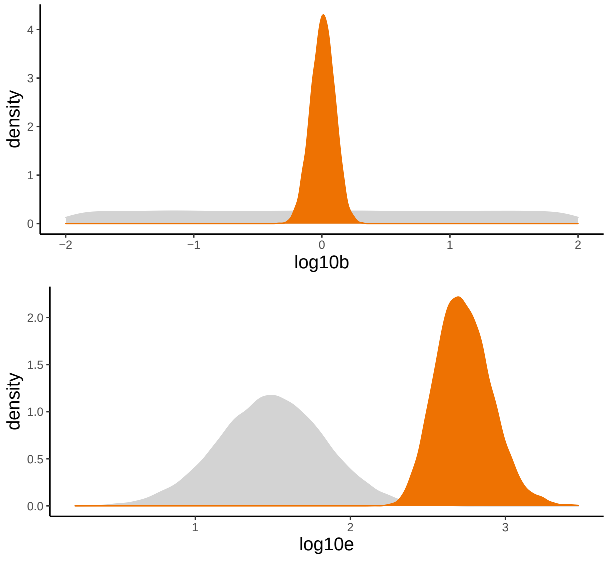

(c) Priors and posteriors

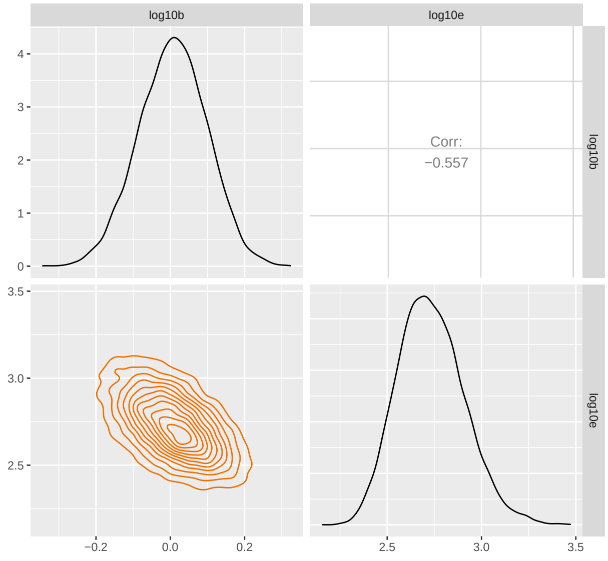

(d) Correlations between parameters

Figure 5: Dose-response curve (a), PPC (b), prior and posterior distributions (c) and correlations between parameters (d).

Data set: GLXMA_SE_emergence

Table 6: Summary of parameter estimates (parameter d is set to 1) for GLXMA_SE_emergence data set

Parameter	median	Q2.5	Q97.5
b	1.308	0.829	2.049
e	525.316	272.339	1279.091

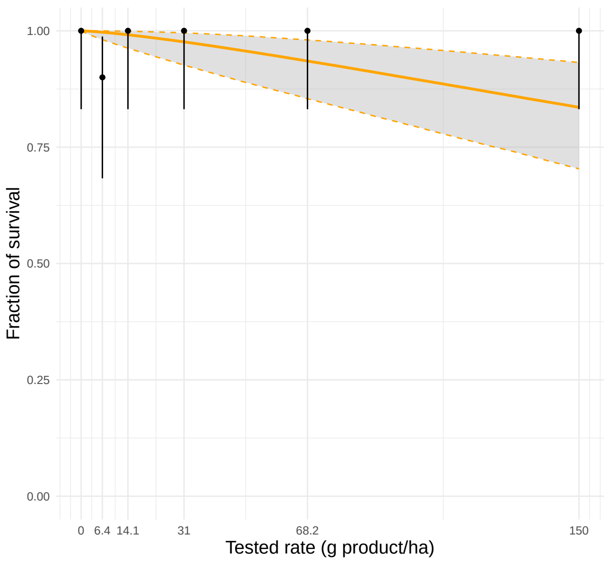

(a) Dose-response curve

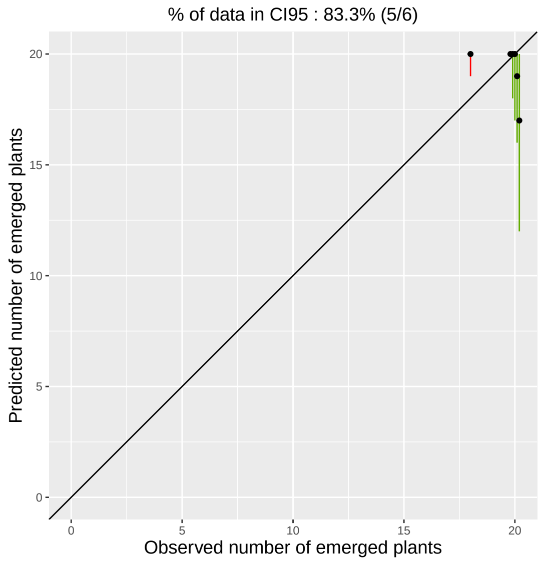

(b) Posterior predictive check (PPC)

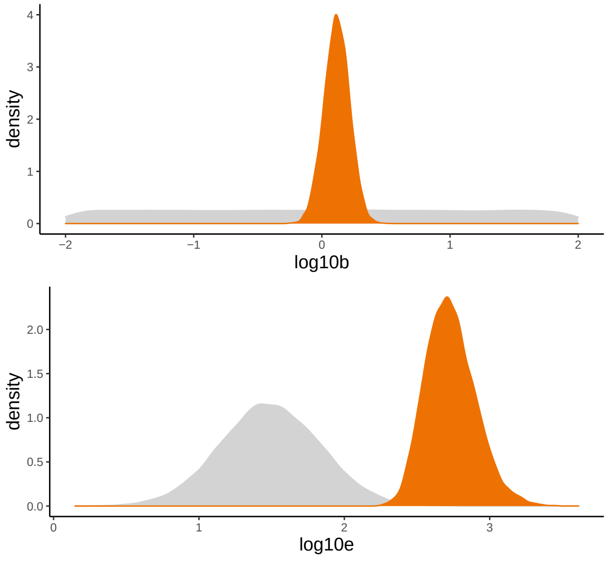

(c) Priors and posteriors

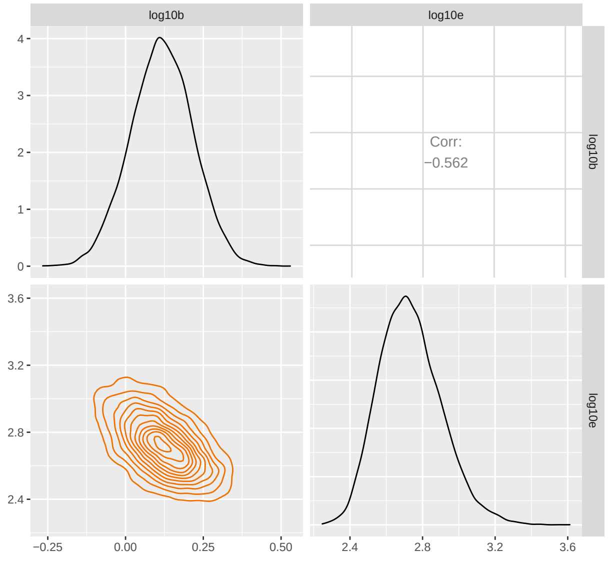

(d) Correlations between parameters

Figure 6: Dose-response curve (a), PPC (b), prior and posterior distributions (c) and correlations between parameters (d).

Data set: HELAN_SE_emergence

Table 7: Summary of parameter estimates (parameter d is set to 1) for HELAN_SE_emergence data set

Parameter	median	Q2.5	Q97.5
b	1.287	0.813	2.032
e	466.659	249.519	1100.256

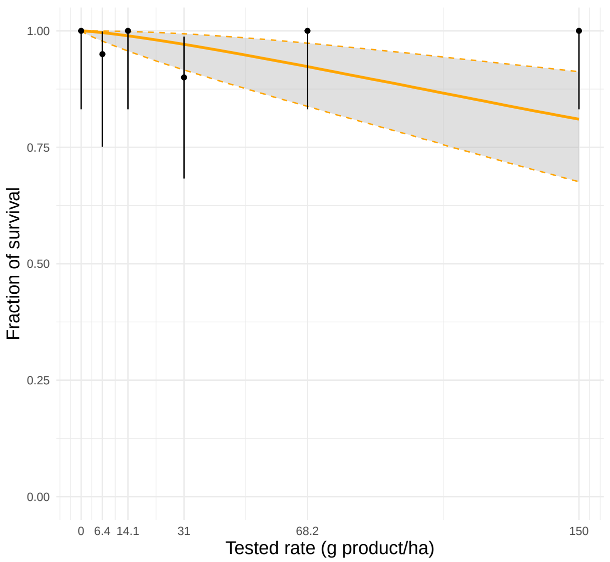

(a) Dose-response curve

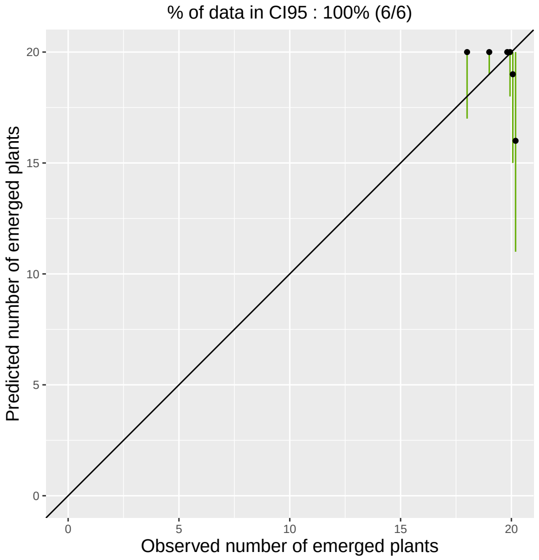

(b) Posterior predictive check (PPC)

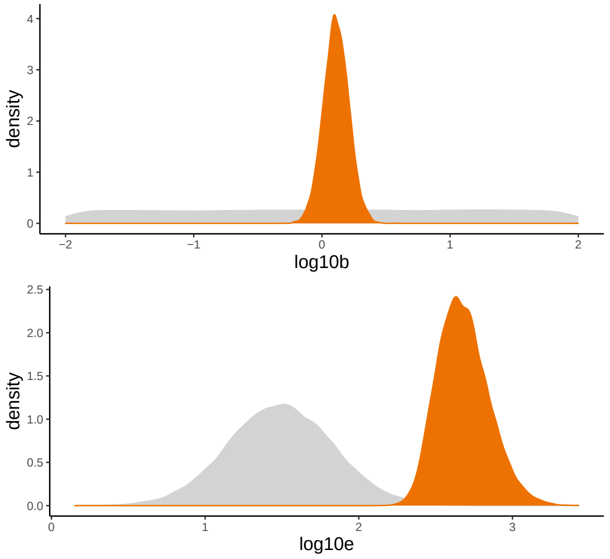

(c) Priors and posteriors

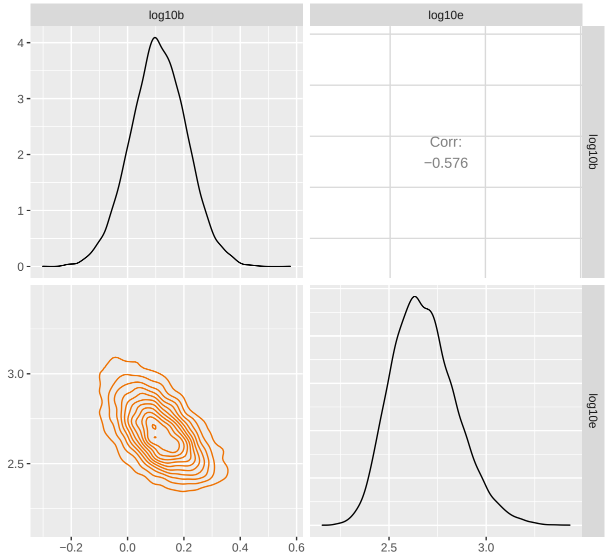

(d) Correlations between parameters

Figure 7: Dose-response curve (a), PPC (b), prior and posterior distributions (c) and correlations between parameters (d).

Data set: LYPES_SE_emergence

Table 8: Summary of parameter estimates (parameter d is set to 1) for LYPES_SE_emergence data set

Parameter	median	Q2.5	Q97.5
b	0.808	0.532	1.191
e	490.435	239.841	1224.281

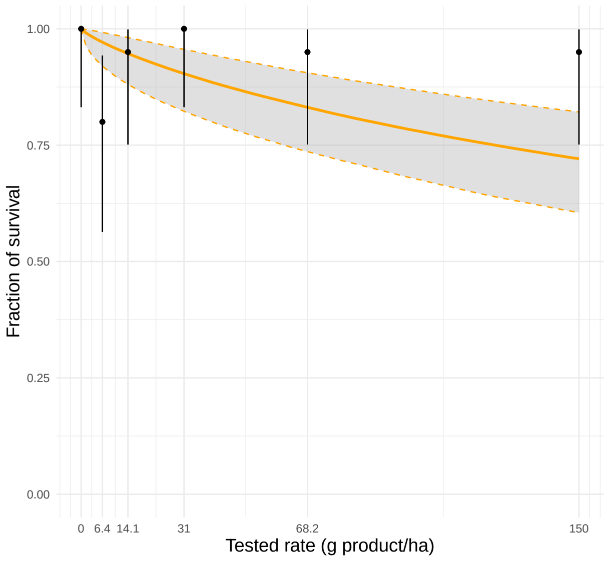

(a) Dose-response curve

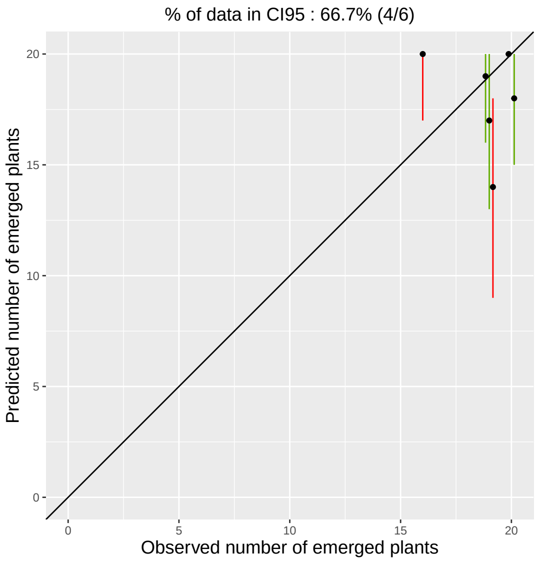

(b) Posterior predictive check (PPC)

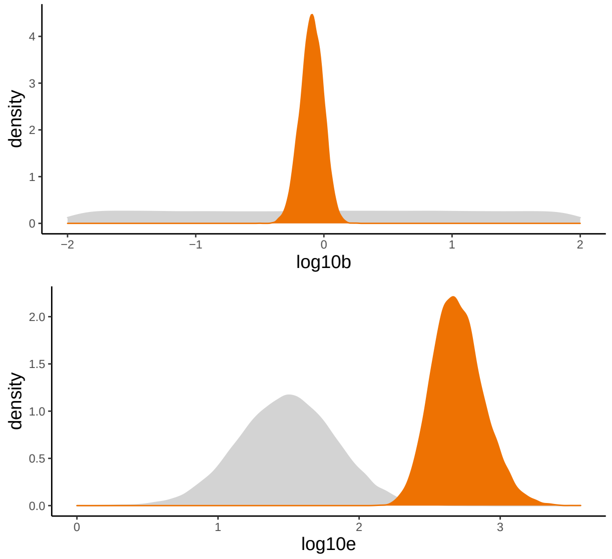

(c) Priors and posteriors

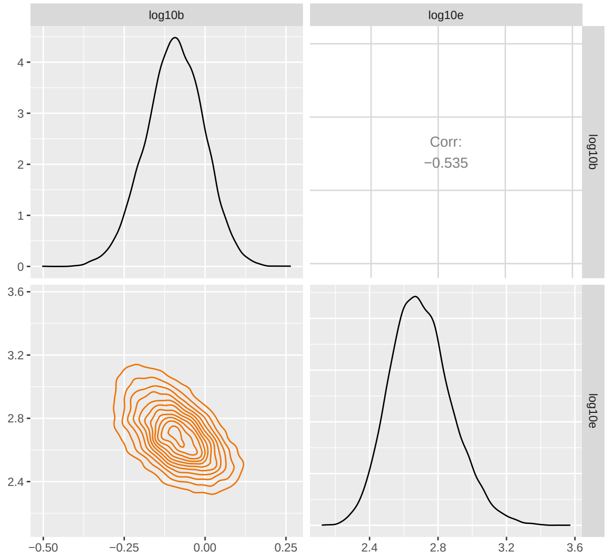

(d) Correlations between parameters

Figure 8: Dose-response curve (a), PPC (b), prior and posterior distributions (c) and correlations between parameters (d).

Data set: TRZAW_SE_emergence

Table 9: Summary of parameter estimates (parameter d is set to 1) for TRZAW_SE_emergence data set

Parameter	median	Q2.5	Q97.5
b	1.375	0.856	2.188
e	437.287	237.799	1049.467

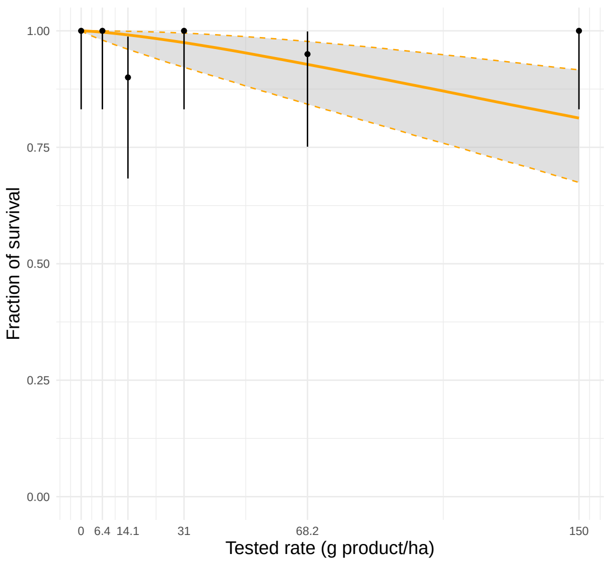

(a) Dose-response curve

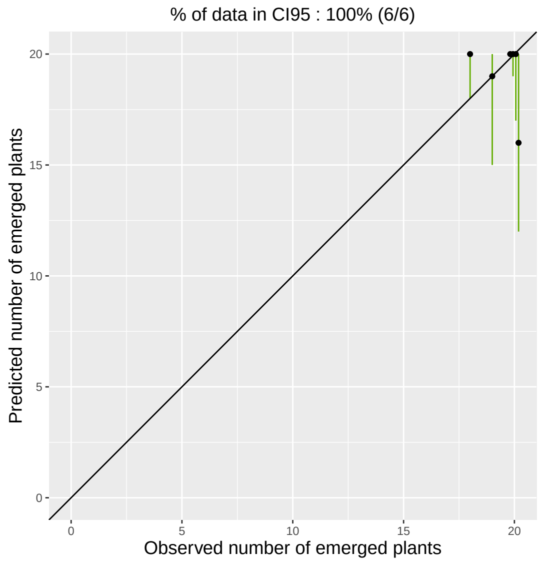

(b) Posterior predictive check (PPC)

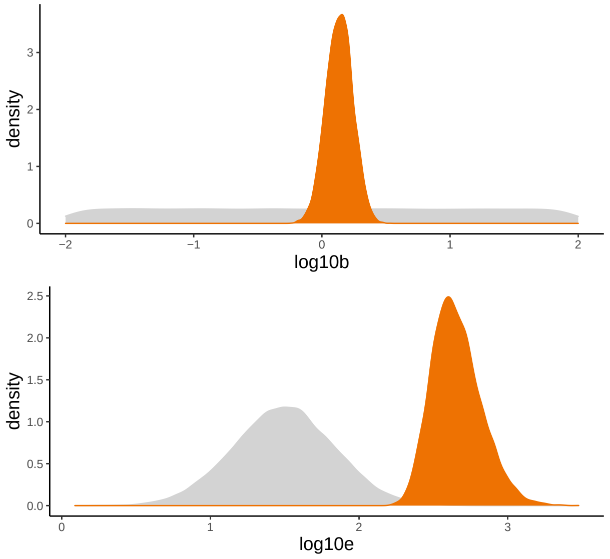

(c) Priors and posteriors

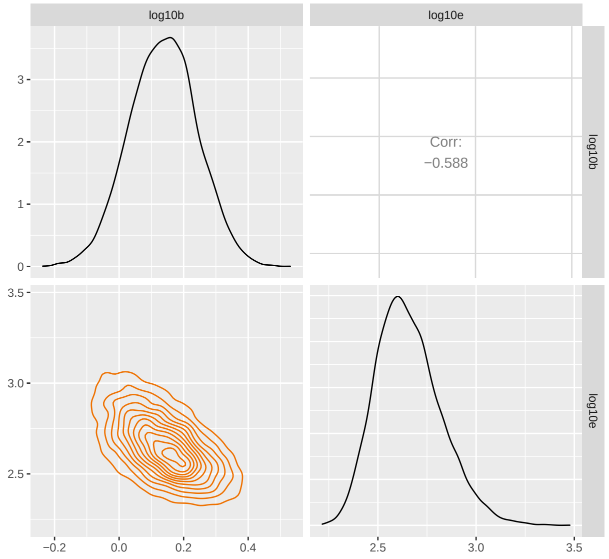

(d) Correlations between parameters

Figure 9: Dose-response curve (a), PPC (b), prior and posterior distributions (c) and correlations between parameters (d).

Data set: ZEAMA_SE_emergence

Table 10: Summary of parameter estimates (parameter d is set to 1) for ZEAMA_SE_emergence data set

Parameter	median	Q2.5	Q97.5
b	1.462	0.904	2.366
e	411.484	228.897	947.331

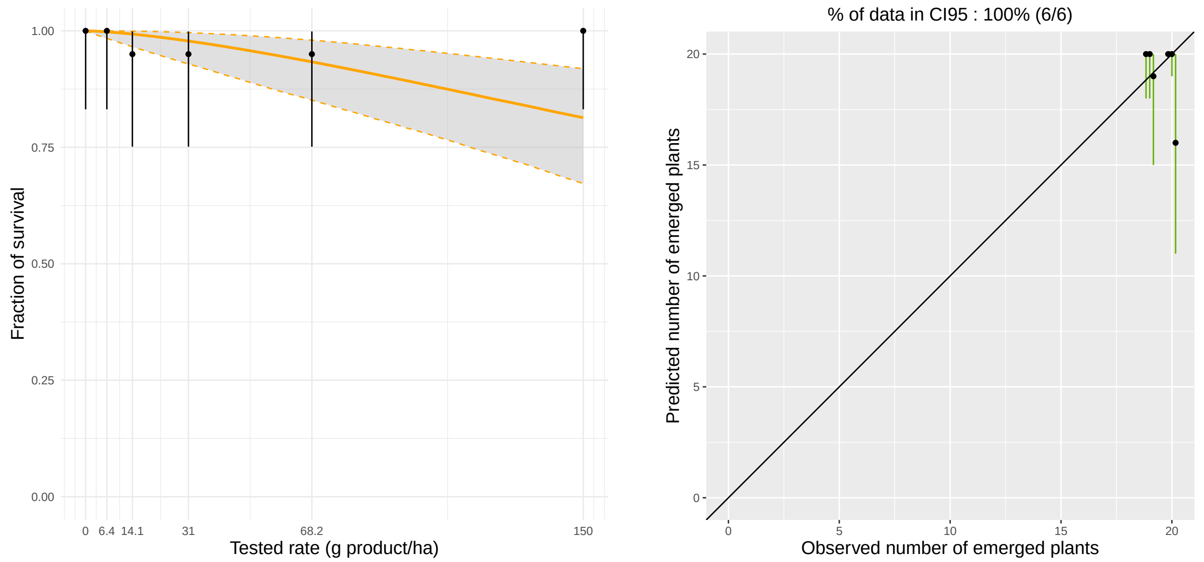

(a) Dose-response curve

(b) Posterior predictive check (PPC)

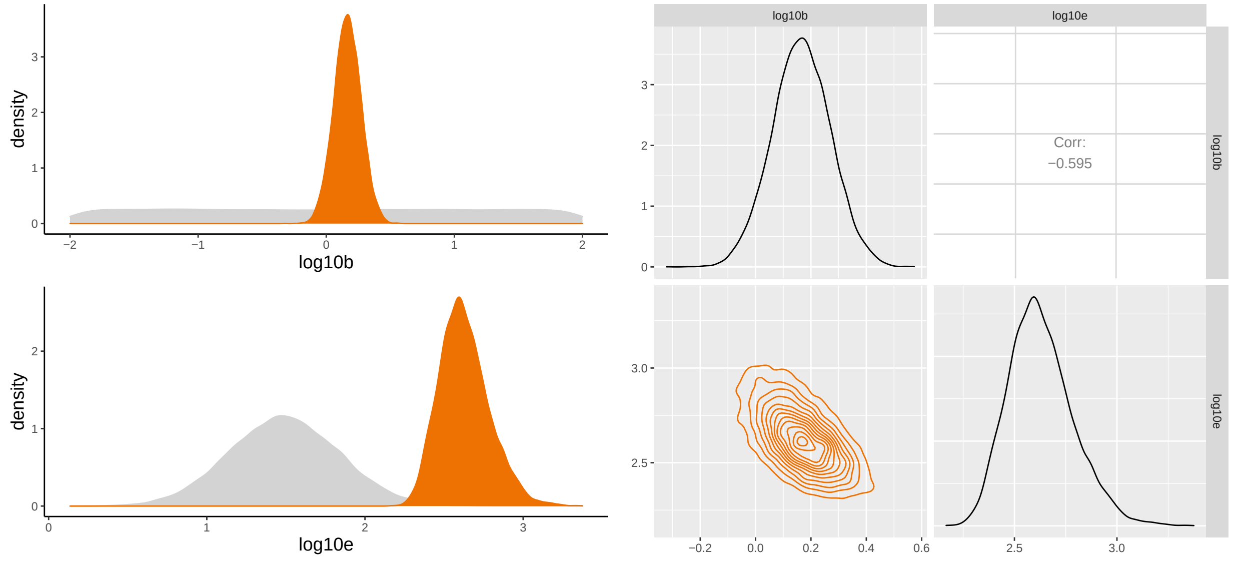

(c) Priors and posteriors

(d) Correlations between parameters

Figure 10: Dose-response curve (a), PPC (b), prior and posterior distributions (c) and correlations between parameters (d).
