# Supplementary material for: How to account for the uncertainty from standard toxicity tests in species sensitivity distributions: An example in non-target plants
Source: PLoS One. 2021 Jan 7;16(1):e0245071. doi: 10.1371/journal.pone.0245071 (PMC7790375; doi:10.1371/journal.pone.0245071)
Supplement: S1 Archive — It is a zip file containing seven folders (one folder per case study). Each folder contains five files report_xxx.pdf with detailed results of the dose-response analyses, one file corresponding to does-response analysis per endpoint. It also contains one file ER50_censoring.pdf for censored ER50 and one file SSD_analyses.pdf for results of SSD analyses. (ZIP) [file pone.0245071.s004.zip › S1_archive/Study6/report_SE_weight.pdf]

Dose-response analysis

Study 6

Seedling Emergence test - shoot dry SE_weight endpoint

25 June 2020

Contact: sandrine.charles@univ-lyon1.fr

This is a report which provides results on all performed dose-response analyses for the shoot dry SE_weight endpoint of the Seedling Emergence test for study 6.

Contents

Data set: ALLCE_SE_weight . . . 2
Data set: AVESA_SE_weight . . . 3
Data set: BEAVA_SE_weight . . . 4
Data set: BRSNW_SE_weight . . . 5
Data set: CUMSA_SE_weight . . . 6
Data set: GLXMA_SE_weight . . . 7
Data set: HELAN_SE_weight . . . 8
Data set: LYPES_SE_weight . . . 9
Data set: TRZAW_SE_weight . . . 10
Data set: ZEAMA_SE_weight . . . 11

Data set: ALLCE_SE_weight

Table 1: Summary of parameter estimates for ALLCE_SE_weight data set

Parameter	median	Q2.5	Q97.5
b	3.223	0.283	62.946
d	0.023	0.017	0.029
e	12.046	7.699	19.231
sigma	0.007	0.005	0.011

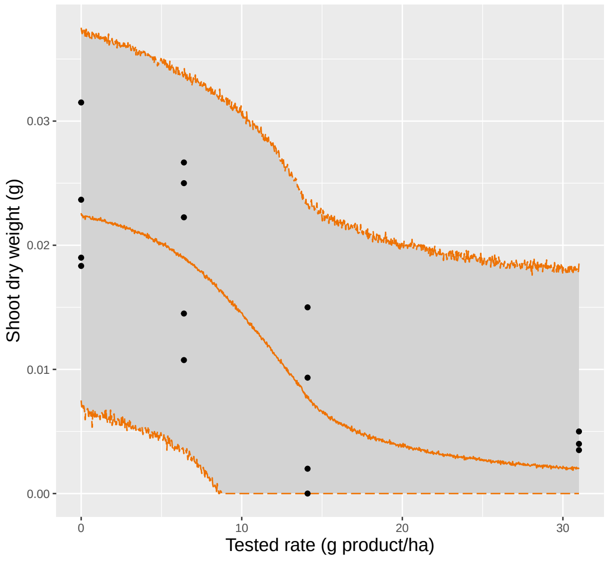

(a) Dose-response curve

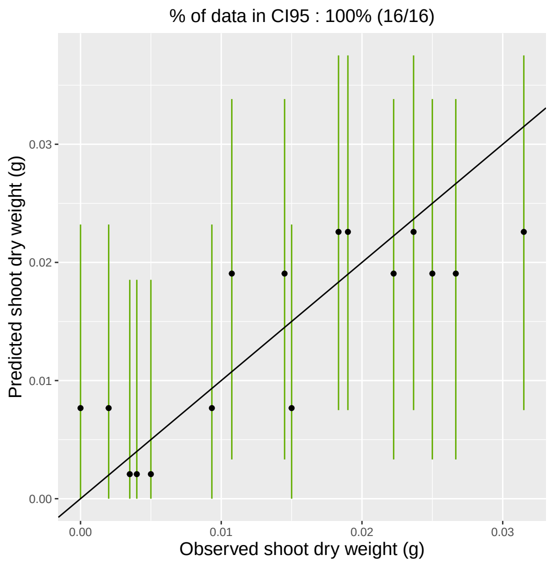

(b) Posterior predictive check (PPC)

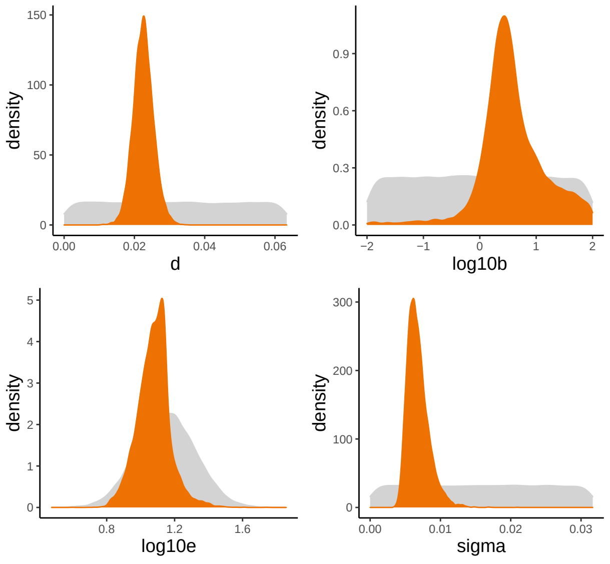

(c) Priors and posteriors

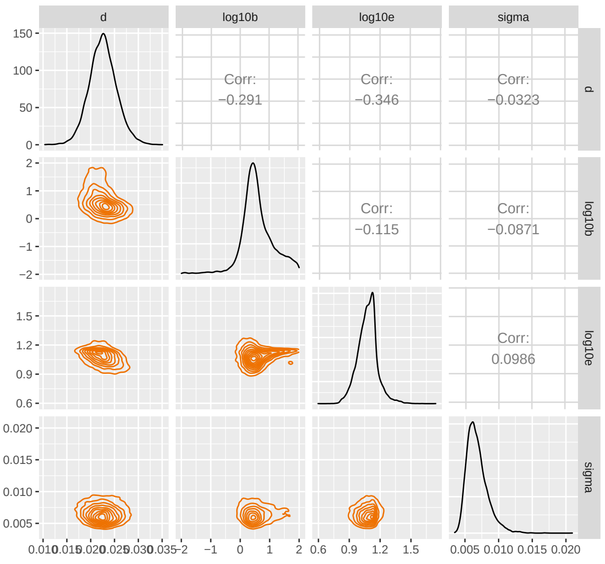

(d) Correlations between parameters

Figure 1: Dose-response curve (a), PPC (b), prior and posterior distributions (c) and correlations between parameters (d).

Data set: AVESA_SE_weight

Table 2: Summary of parameter estimates for AVESA_SE_weight data set

Parameter	median	Q2.5	Q97.5
b	2.114	1.579	3.046
d	0.476	0.438	0.516
e	25.207	21.098	29.880
sigma	0.048	0.037	0.065

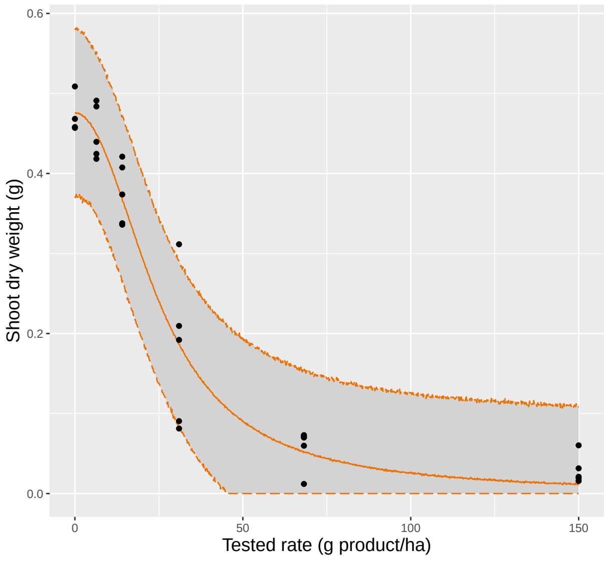

(a) Dose-response curve

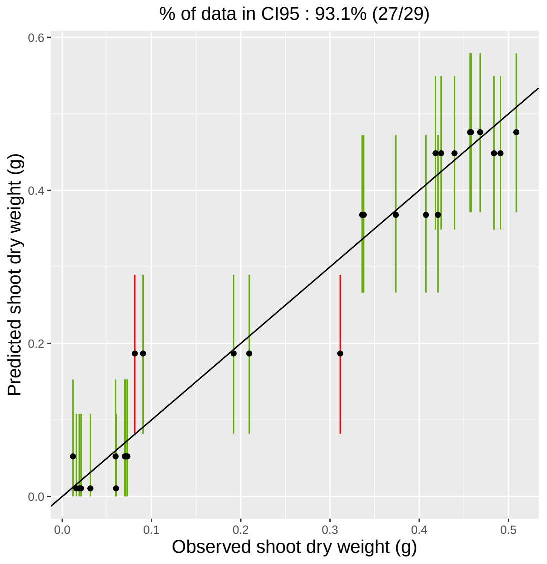

(b) Posterior predictive check (PPC)

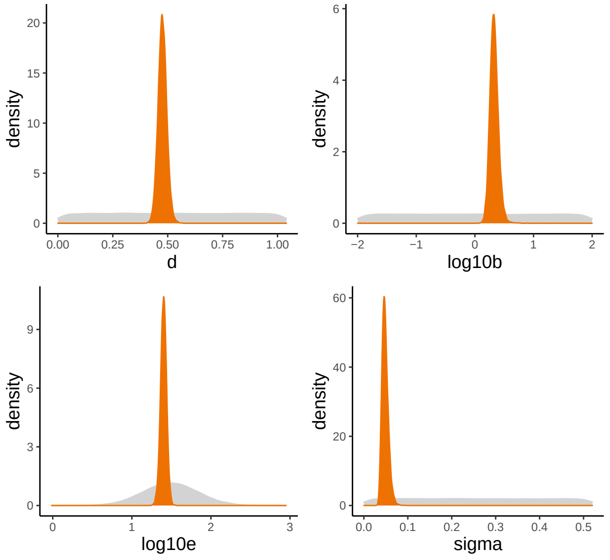

(c) Priors and posteriors

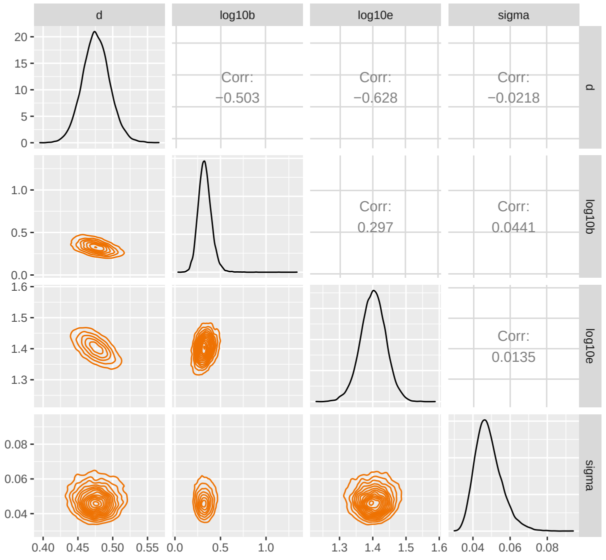

(d) Correlations between parameters

Figure 2: Dose-response curve (a), PPC (b), prior and posterior distributions (c) and correlations between parameters (d).

Data set: BEAVA_SE_weight

Table 3: Summary of parameter estimates for BEAVA_SE_weight data set

Parameter	median	Q2.5	Q97.5
b	2.401	1.807	3.324
d	0.574	0.529	0.619
e	11.054	9.637	12.722
sigma	0.068	0.055	0.088

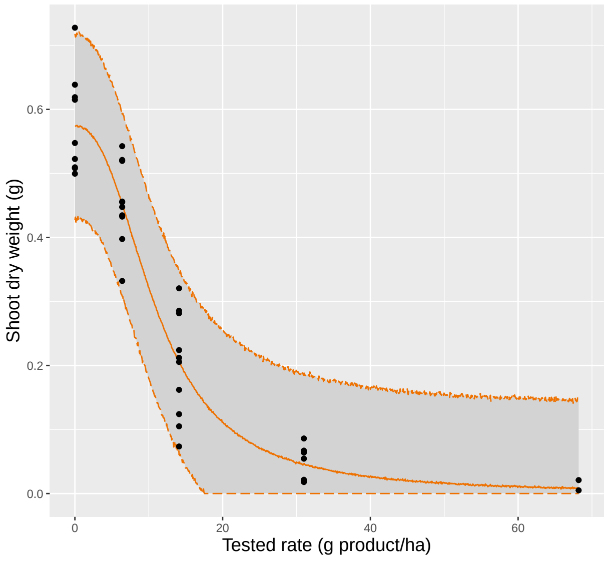

(a) Dose-response curve

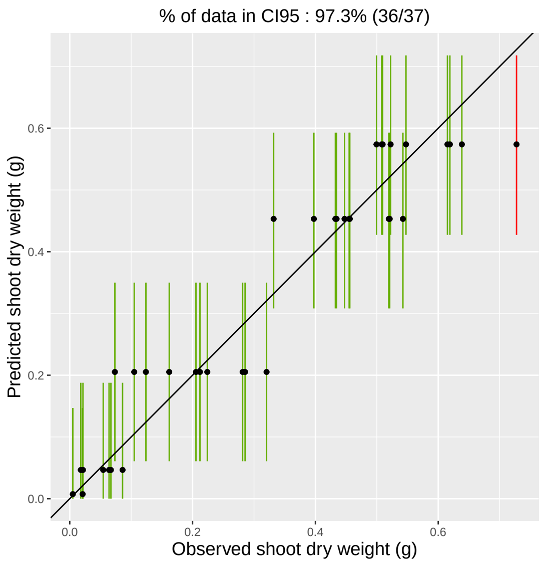

(b) Posterior predictive check (PPC)

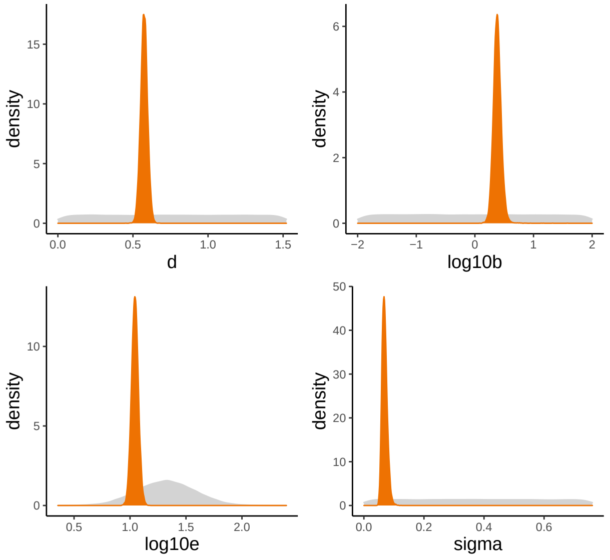

(c) Priors and posteriors

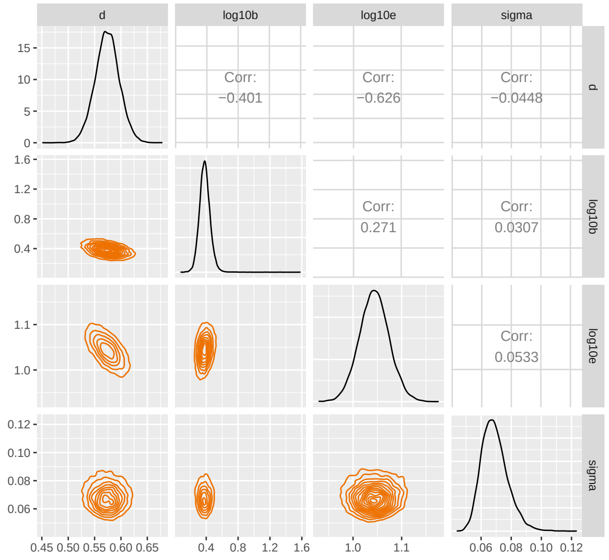

(d) Correlations between parameters

Figure 3: Dose-response curve (a), PPC (b), prior and posterior distributions (c) and correlations between parameters (d).

Data set: BRSNW_SE_weight

Table 4: Summary of parameter estimates for BRSNW_SE_weight data set

Parameter	median	Q2.5	Q97.5
b	2.937	1.786	7.862
d	0.775	0.682	0.871
e	21.521	16.761	28.252
sigma	0.183	0.149	0.233

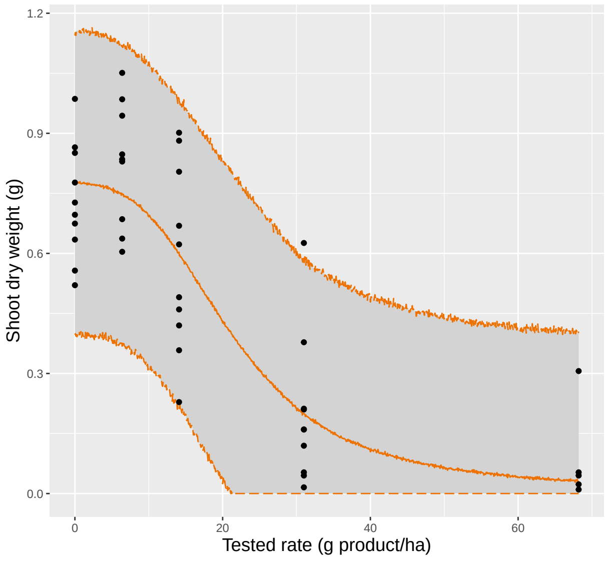

(a) Dose-response curve

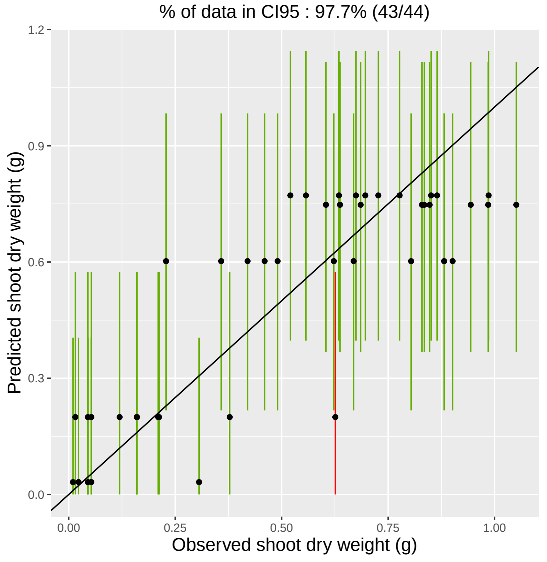

(b) Posterior predictive check (PPC)

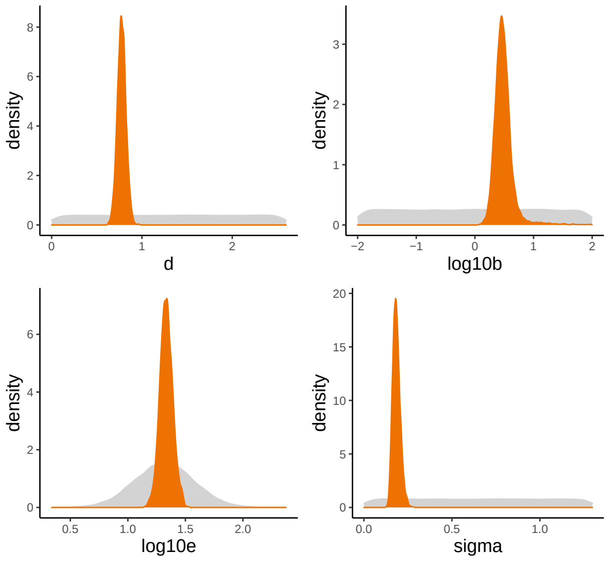

(c) Priors and posteriors

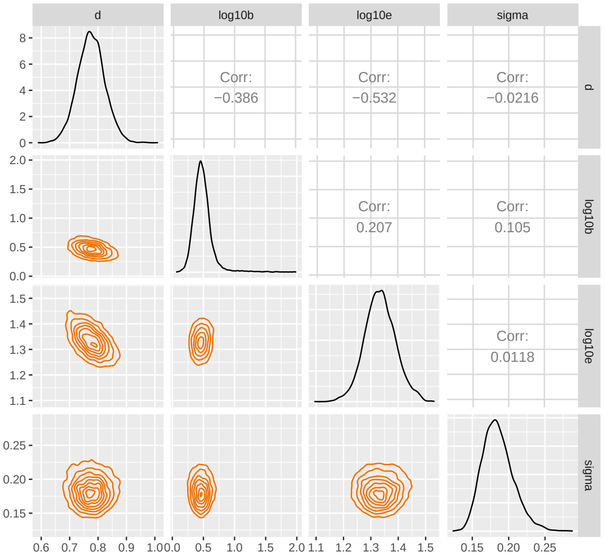

(d) Correlations between parameters

Figure 4: Dose-response curve (a), PPC (b), prior and posterior distributions (c) and correlations between parameters (d).

Data set: CUMSA_SE_weight

Table 5: Summary of parameter estimates for CUMSA_SE_weight data set

Parameter	median	Q2.5	Q97.5
b	1.061	0.780	1.445
d	1.897	1.700	2.097
e	28.643	20.359	40.758
sigma	0.336	0.281	0.410

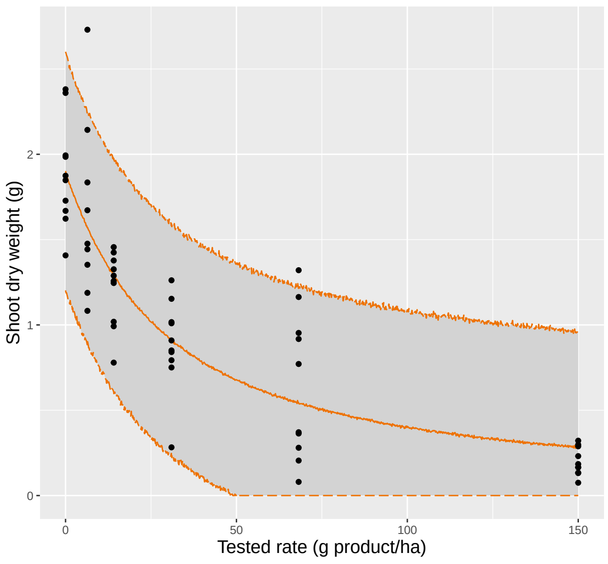

(a) Dose-response curve

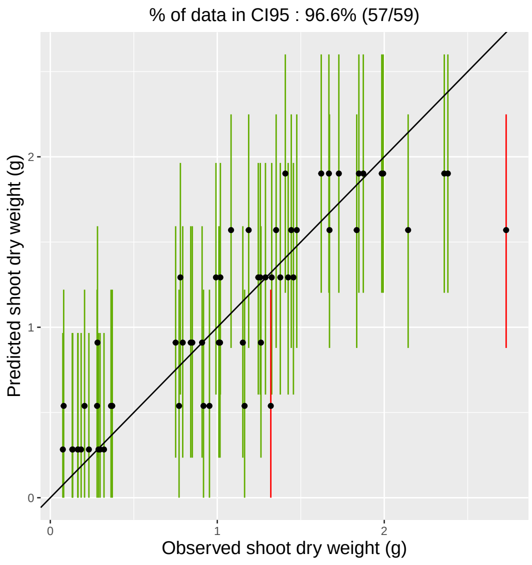

(b) Posterior predictive check (PPC)

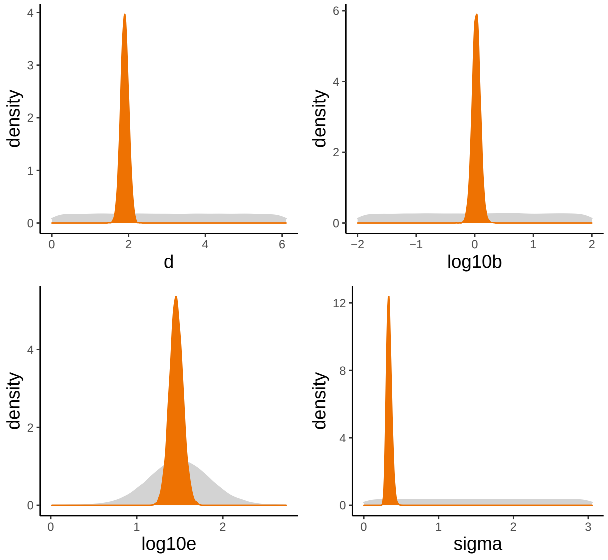

(c) Priors and posteriors

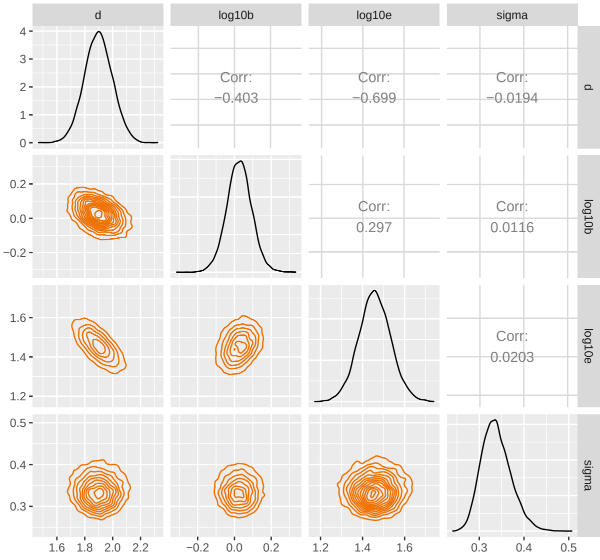

(d) Correlations between parameters

Figure 5: Dose-response curve (a), PPC (b), prior and posterior distributions (c) and correlations between parameters (d).

Data set: GLXMA_SE_weight

Table 6: Summary of parameter estimates for GLXMA_SE_weight data set

Parameter	median	Q2.5	Q97.5
b	2.901	1.006	25.938
d	1.211	1.136	1.333
e	145.290	121.453	176.902
sigma	0.214	0.179	0.260

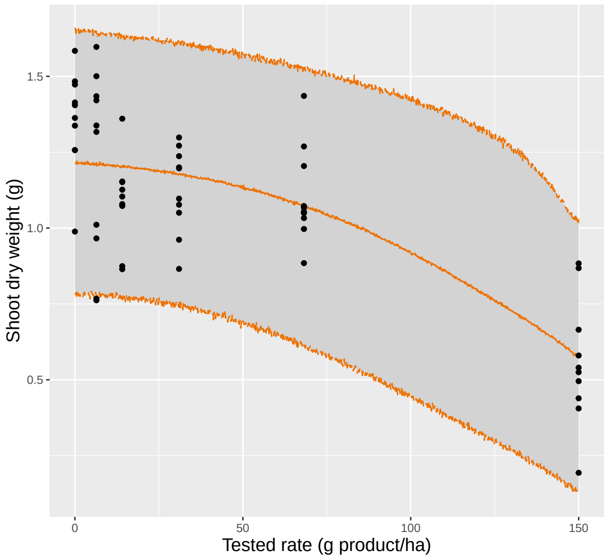

(a) Dose-response curve

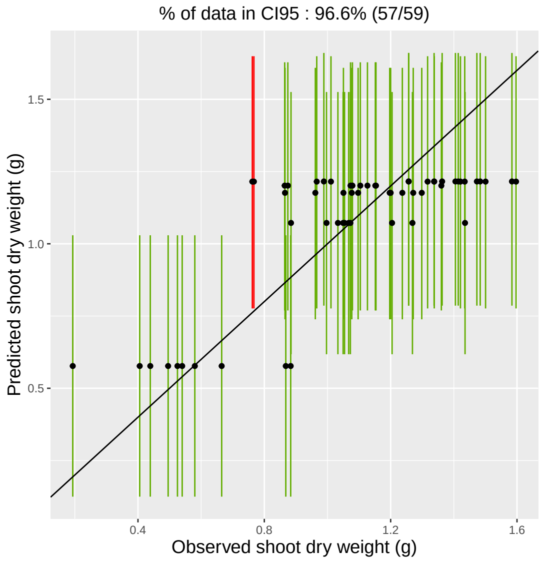

(b) Posterior predictive check (PPC)

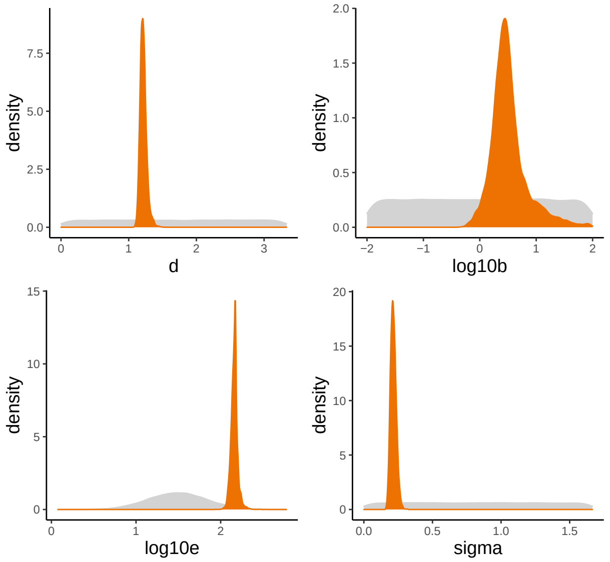

(c) Priors and posteriors

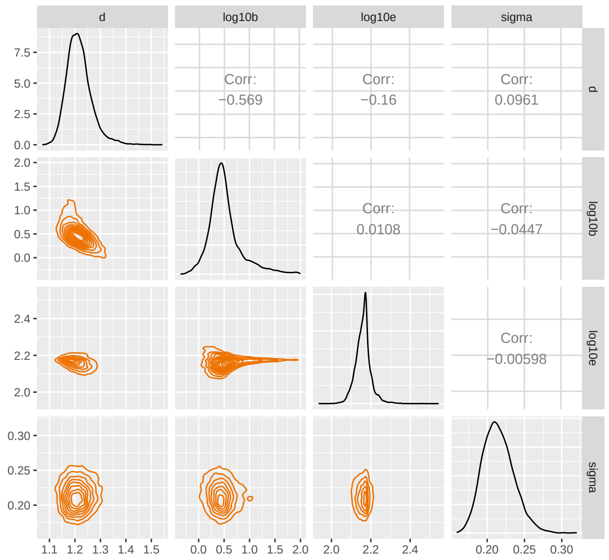

(d) Correlations between parameters

Figure 6: Dose-response curve (a), PPC (b), prior and posterior distributions (c) and correlations between parameters (d).

Data set: HELAN_SE_weight

Table 7: Summary of parameter estimates for HELAN_SE_weight data set

Parameter	median	Q2.5	Q97.5
b	2.423	1.520	4.328
d	0.426	0.389	0.466
e	48.604	38.692	60.364
sigma	0.085	0.071	0.105

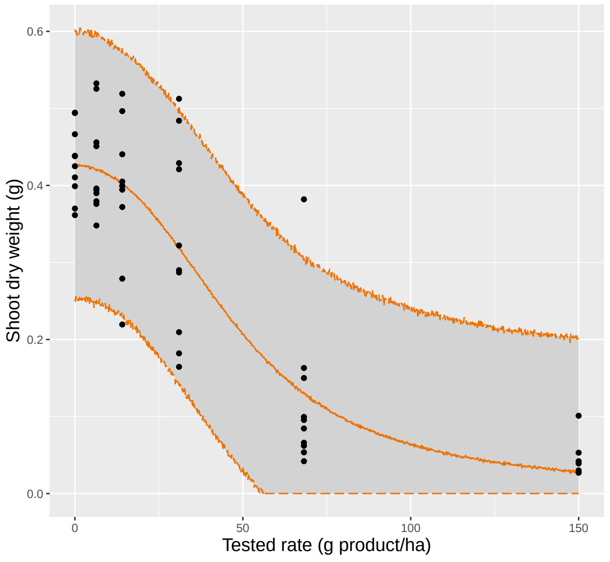

(a) Dose-response curve

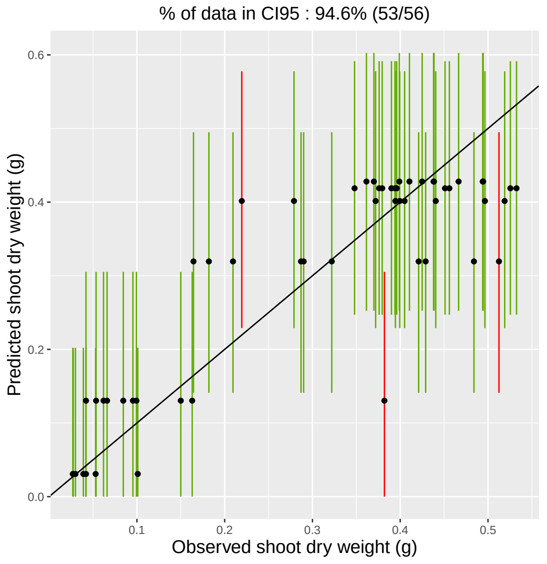

(b) Posterior predictive check (PPC)

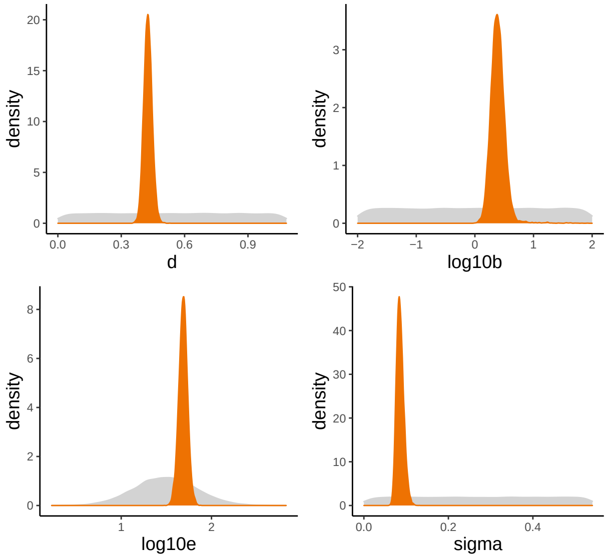

(c) Priors and posteriors

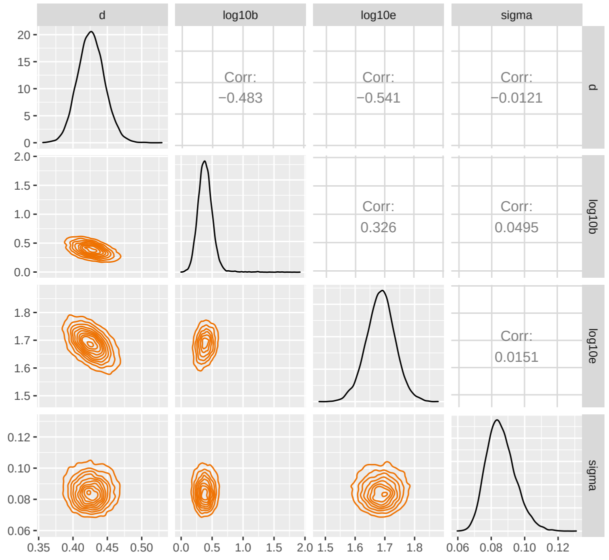

(d) Correlations between parameters

Figure 7: Dose-response curve (a), PPC (b), prior and posterior distributions (c) and correlations between parameters (d).

Data set: LYPES_SE_weight

Table 8: Summary of parameter estimates for LYPES_SE_weight data set

Parameter	median	Q2.5	Q97.5
b	2.819	2.014	4.190
d	0.738	0.678	0.803
e	21.317	18.196	25.120
sigma	0.114	0.095	0.141

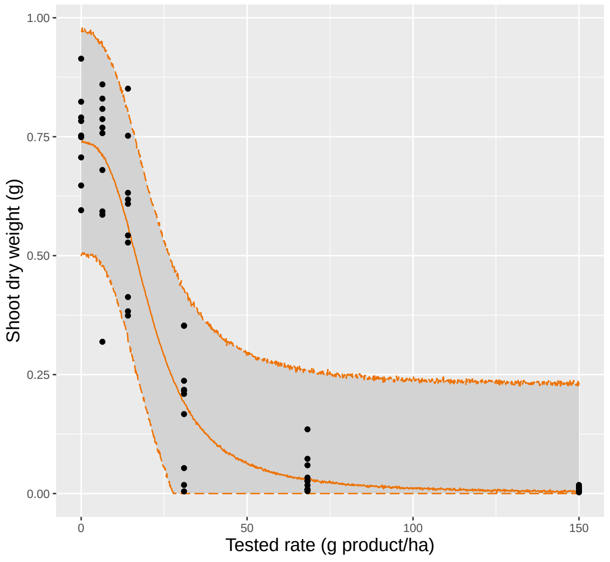

(a) Dose-response curve

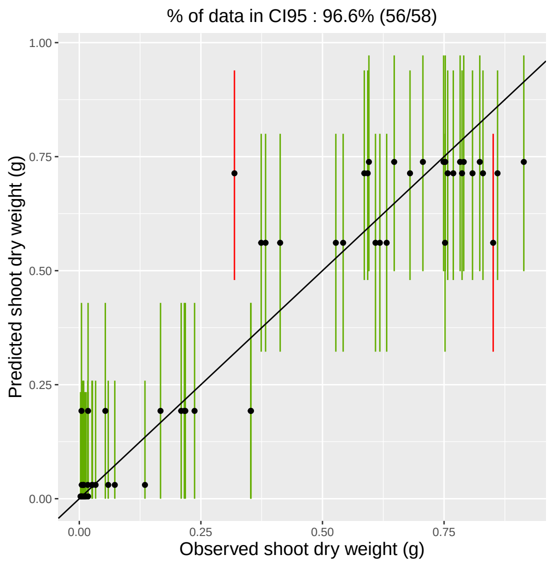

(b) Posterior predictive check (PPC)

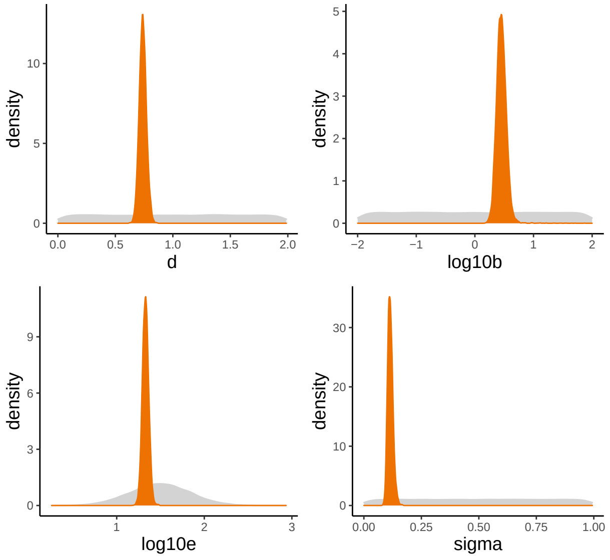

(c) Priors and posteriors

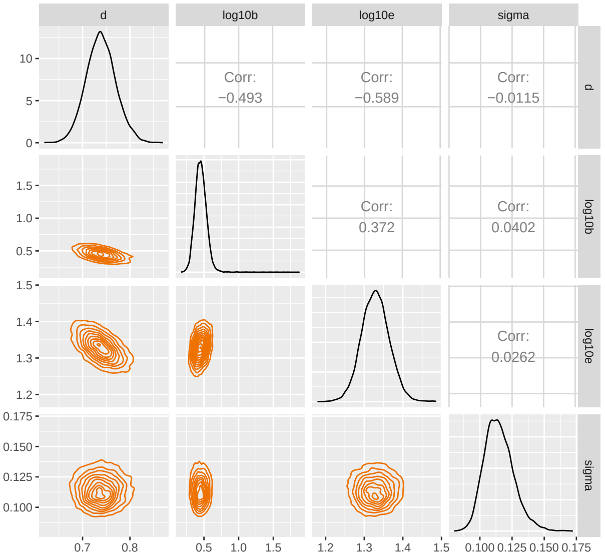

(d) Correlations between parameters

Figure 8: Dose-response curve (a), PPC (b), prior and posterior distributions (c) and correlations between parameters (d).

Data set: TRZAW_SE_weight

Table 9: Summary of parameter estimates for TRZAW_SE_weight data set

Parameter	median	Q2.5	Q97.5
b	1.777	0.992	4.966
d	0.331	0.310	0.356
e	167.744	138.635	229.056
sigma	0.035	0.027	0.047

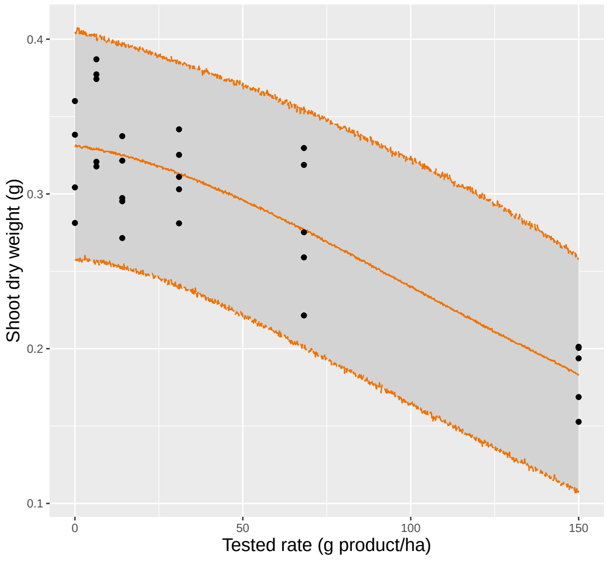

(a) Dose-response curve

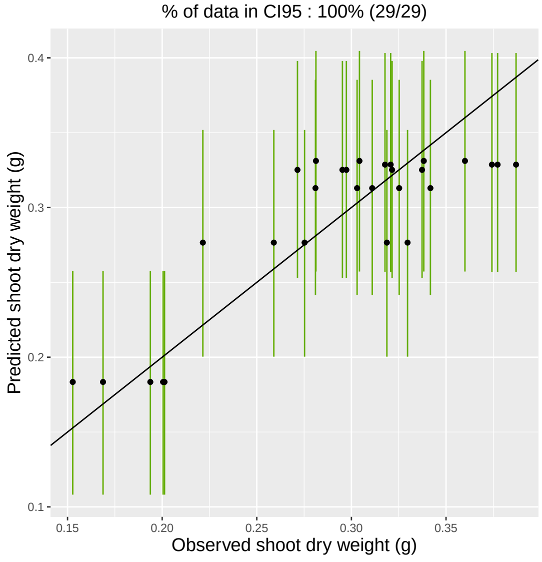

(b) Posterior predictive check (PPC)

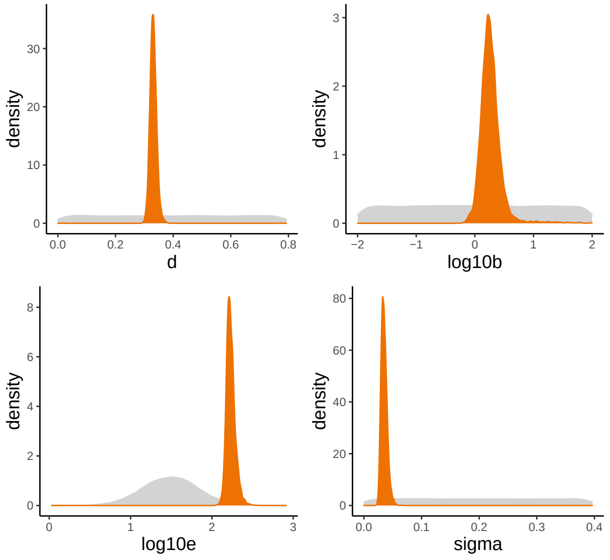

(c) Priors and posteriors

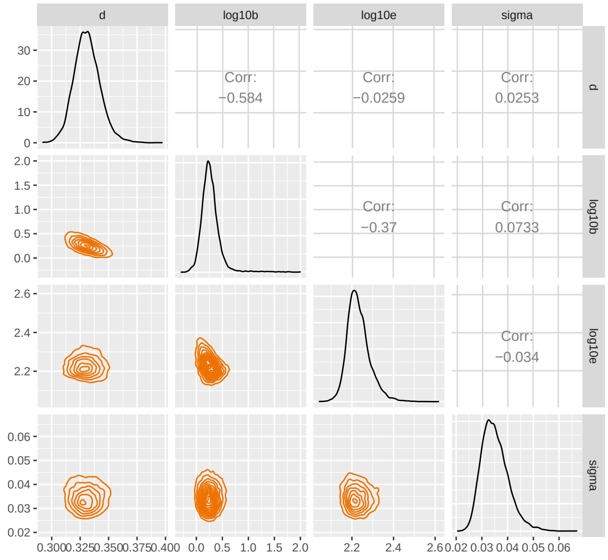

(d) Correlations between parameters

Figure 9: Dose-response curve (a), PPC (b), prior and posterior distributions (c) and correlations between parameters (d).

Data set: ZEAMA_SE_weight

Table 10: Summary of parameter estimates for ZEAMA_SE_weight data set

Parameter	median	Q2.5	Q97.5
b	2.072	1.051	21.372
d	1.876	1.762	2.011
e	194.498	153.445	300.683
sigma	0.316	0.265	0.387

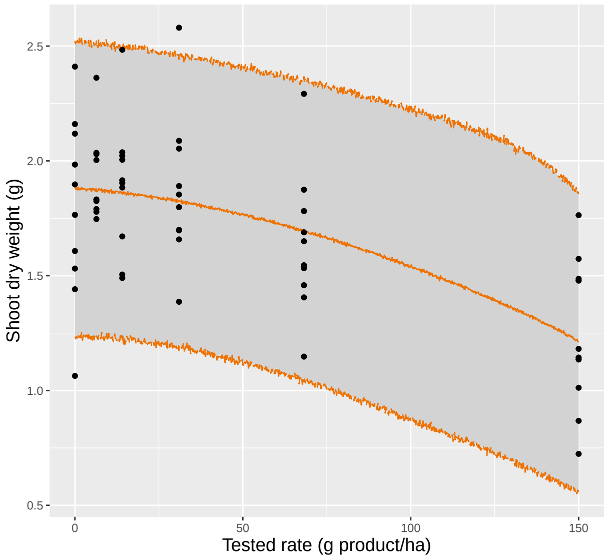

(a) Dose-response curve

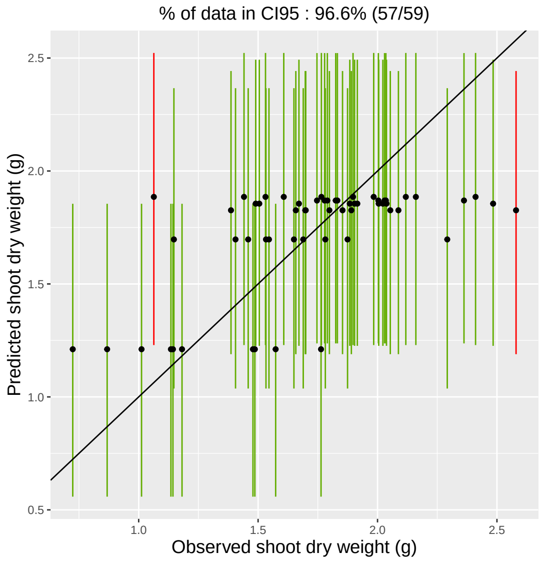

(b) Posterior predictive check (PPC)

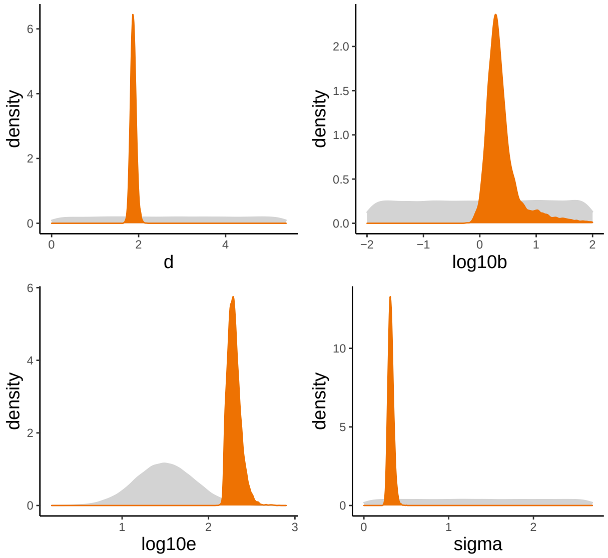

(c) Priors and posteriors

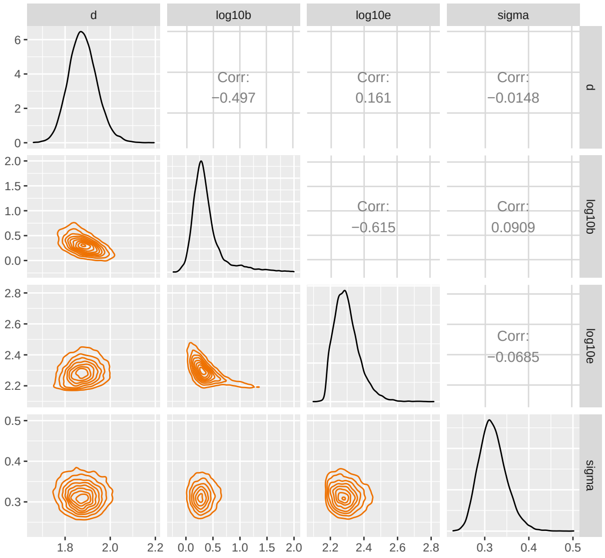

(d) Correlations between parameters

Figure 10: Dose-response curve (a), PPC (b), prior and posterior distributions (c) and correlations between parameters (d).
