# Supplementary material for: How to account for the uncertainty from standard toxicity tests in species sensitivity distributions: An example in non-target plants
Source: PLoS One. 2021 Jan 7;16(1):e0245071. doi: 10.1371/journal.pone.0245071 (PMC7790375; doi:10.1371/journal.pone.0245071)
Supplement: S1 Archive — It is a zip file containing seven folders (one folder per case study). Each folder contains five files report_xxx.pdf with detailed results of the dose-response analyses, one file corresponding to does-response analysis per endpoint. It also contains one file ER50_censoring.pdf for censored ER50 and one file SSD_analyses.pdf for results of SSD analyses. (ZIP) [file pone.0245071.s004.zip › S1_archive/Study6/report_VV_weight.pdf]

Dose-response analysis

Study 6

Vegetative Vigour test - shoot dry VV_weight endpoint

25 June 2020

Contact: sandrine.charles@univ-lyon1.fr

This is a report which provides results on all performed dose-response analyses for the shoot dry VV_weight endpoint of the Vegetative Vigour test for study 6.

Contents

Data set: ALLCE_VV_weight . . . 2
Data set: AVESA_VV_weight . . . 3
Data set: BEAVA_VV_weight . . . 4
Data set: BRSNW_VV_weight . . . 5
Data set: CUMSA_VV_weight . . . 6
Data set: GLXMA_VV_weight . . . 7
Data set: HELAN_VV_weight . . . 8
Data set: LYPES_VV_weight . . . 9
Data set: TRZAW_VV_weight . . . 10
Data set: ZEAMA_VV_weight . . . 11

Data set: ALLCE_VV_weight

Table 1: Summary of parameter estimates for ALLCE_VV_weight data set

Parameter	median	Q2.5	Q97.5
b	3.278	1.899	15.795
d	0.350	0.325	0.377
e	53.200	43.459	65.797
sigma	0.051	0.039	0.070

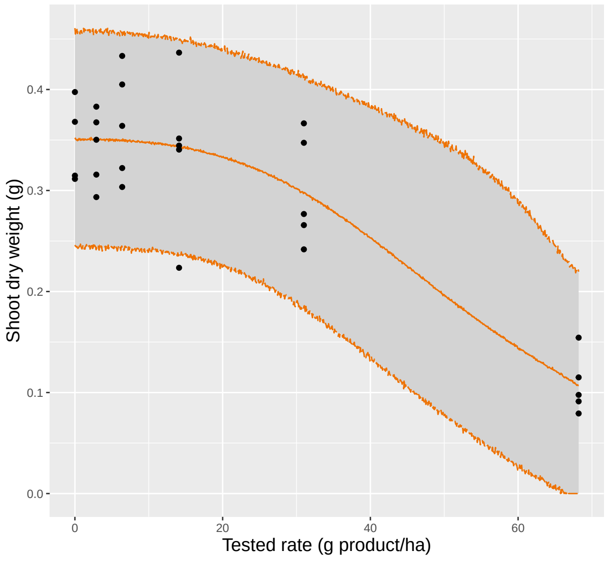

(a) Dose-response curve

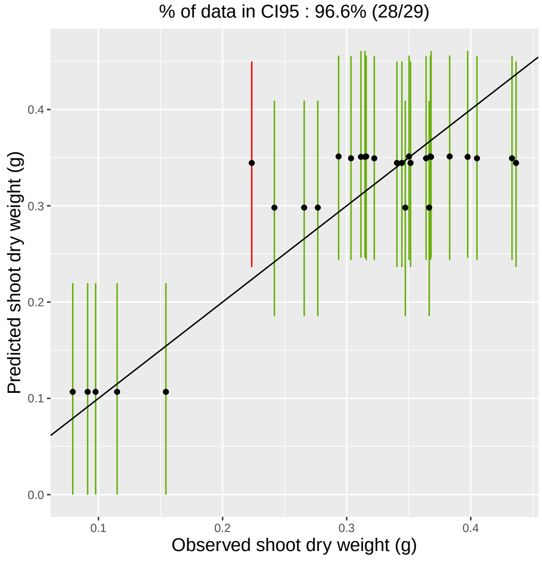

(b) Posterior predictive check (PPC)

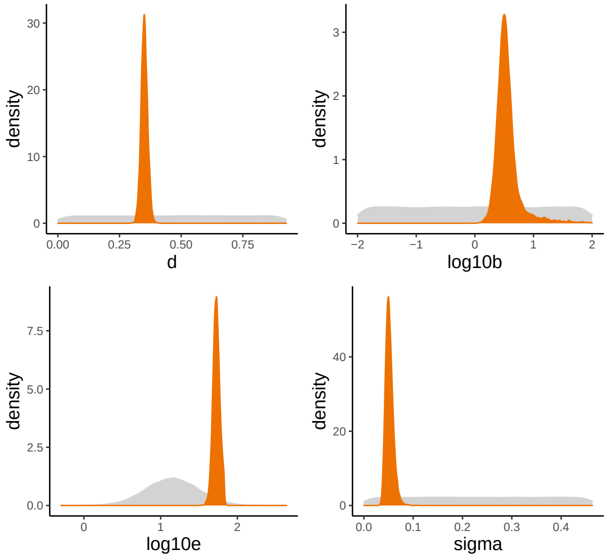

(c) Priors and posteriors

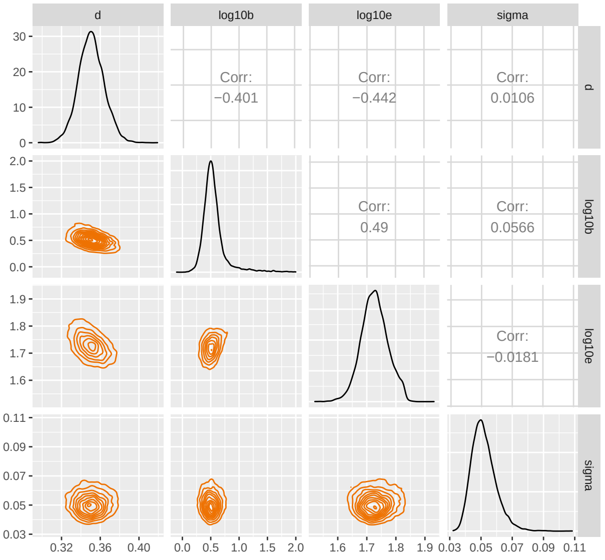

(d) Correlations between parameters

Figure 1: Dose-response curve (a), PPC (b), prior and posterior distributions (c) and correlations between parameters (d).

Data set: AVESA_VV_weight

Table 2: Summary of parameter estimates for AVESA_VV_weight data set

Parameter	median	Q2.5	Q97.5
b	2.275	1.272	20.065
d	1.225	1.092	1.368
e	31.971	26.373	37.222
sigma	0.112	0.075	0.196

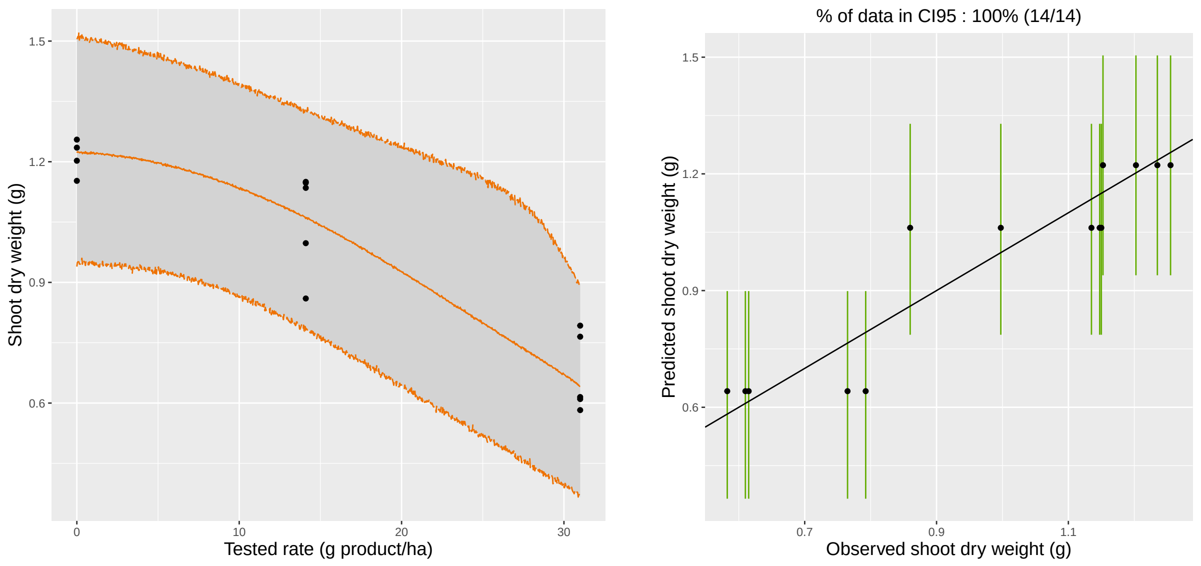

(a) Dose-response curve (b) Posterior predictive check (PPC)

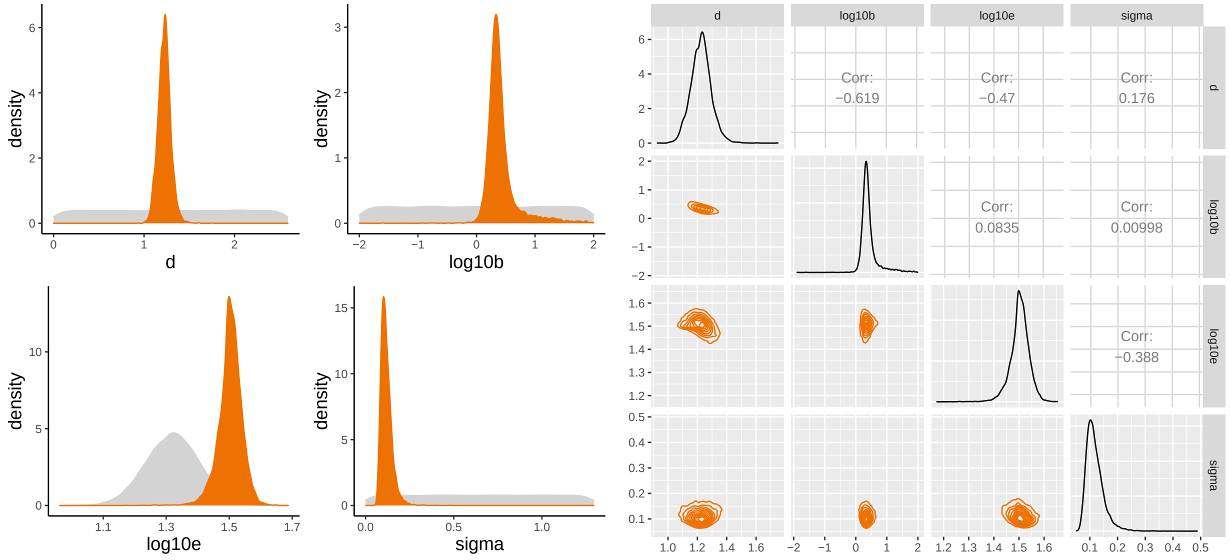

(c) Priors and posteriors (d) Correlations between parameters

Figure 2: Dose-response curve (a), PPC (b), prior and posterior distributions (c) and correlations between parameters (d).

Data set: BEAVA_VV_weight

Table 3: Summary of parameter estimates for BEAVA_VV_weight data set

Parameter	median	Q2.5	Q97.5
b	1.200	0.932	1.564
d	3.545	3.272	3.826
e	7.202	5.817	9.057
sigma	0.453	0.378	0.556

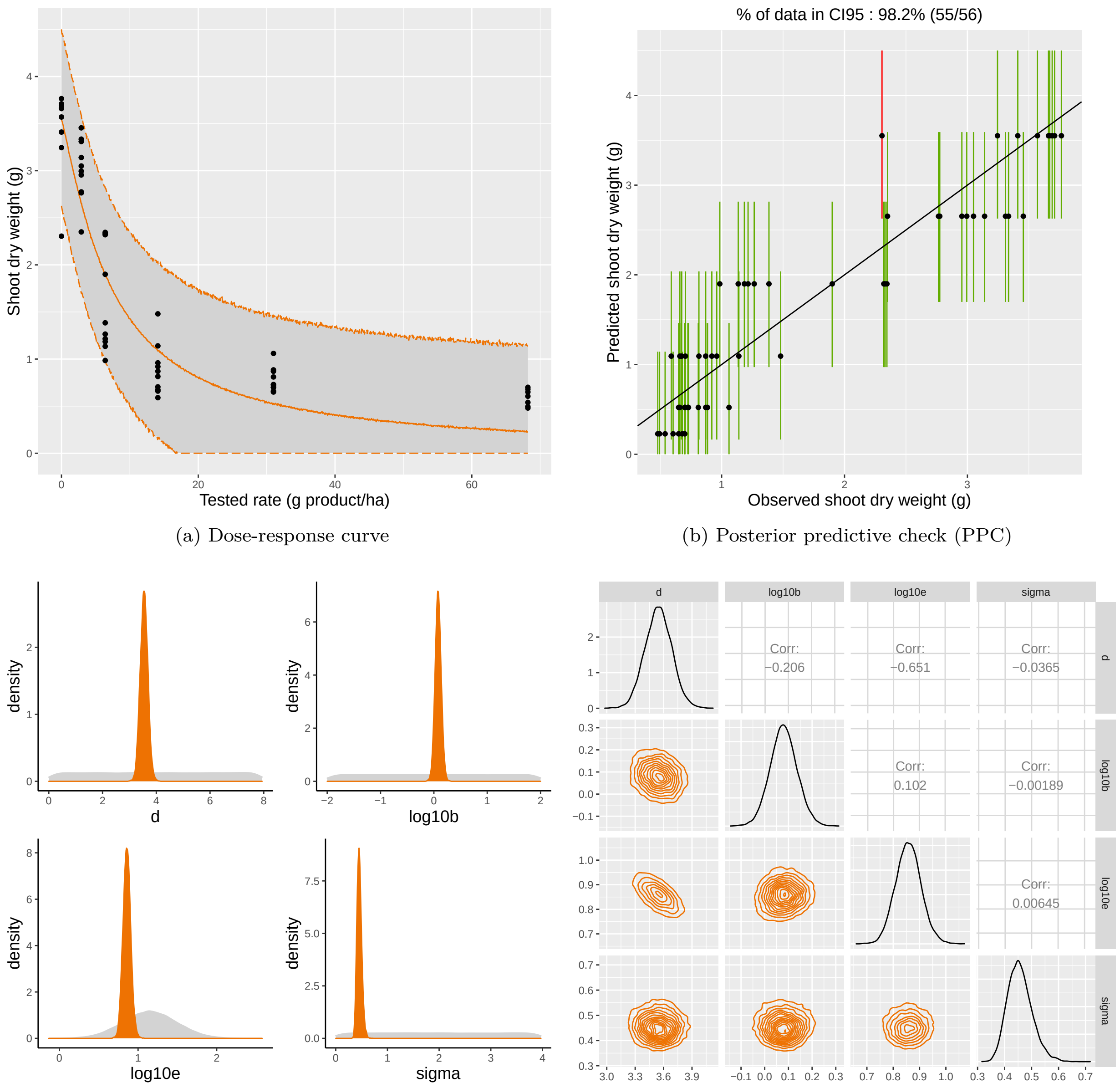

(a) Dose-response curve

(b) Posterior predictive check (PPC)

(c) Priors and posteriors

(d) Correlations between parameters

Figure 3: Dose-response curve (a), PPC (b), prior and posterior distributions (c) and correlations between parameters (d).

Data set: BRSNW_VV_weight

Table 4: Summary of parameter estimates for BRSNW_VV_weight data set

Parameter	median	Q2.5	Q97.5
b	1.842	1.306	2.683
d	5.065	4.559	5.593
e	8.772	6.973	11.150
sigma	0.912	0.754	1.130

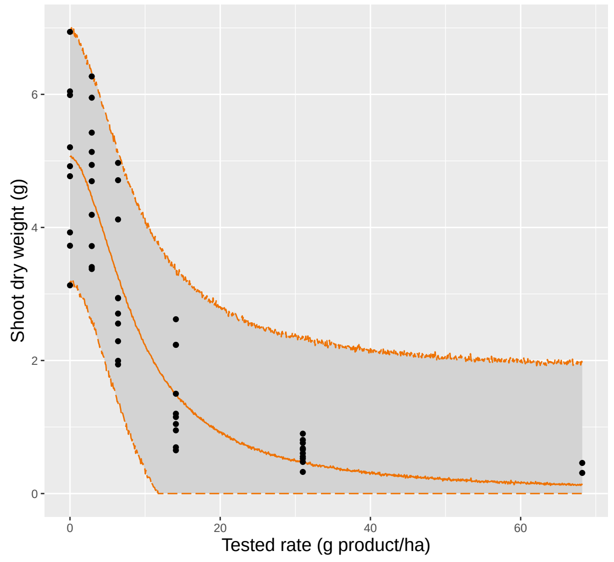

(a) Dose-response curve

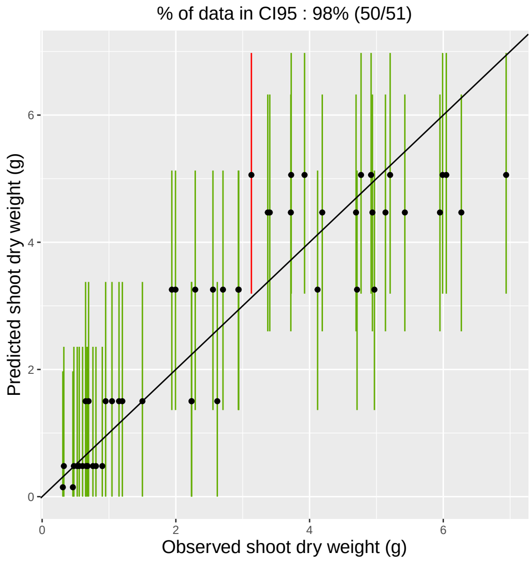

(b) Posterior predictive check (PPC)

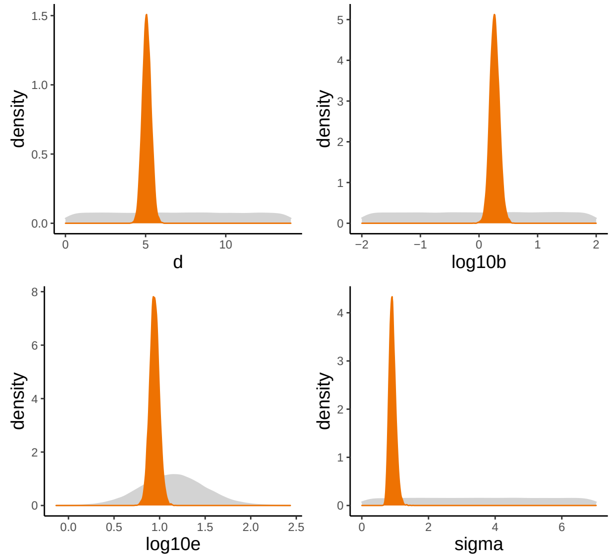

(c) Priors and posteriors

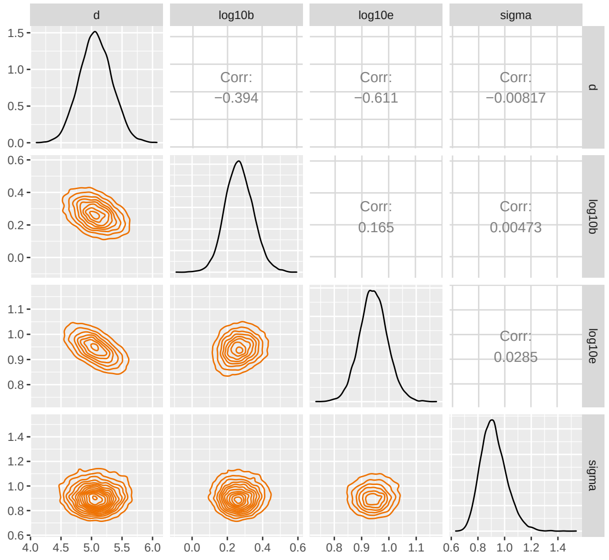

(d) Correlations between parameters

Figure 4: Dose-response curve (a), PPC (b), prior and posterior distributions (c) and correlations between parameters (d).

Data set: CUMSA_VV_weight

Table 5: Summary of parameter estimates for CUMSA_VV_weight data set

Parameter	median	Q2.5	Q97.5
b	0.989	0.801	1.213
d	8.268	7.685	8.874
e	14.147	11.163	18.087
sigma	0.993	0.834	1.207

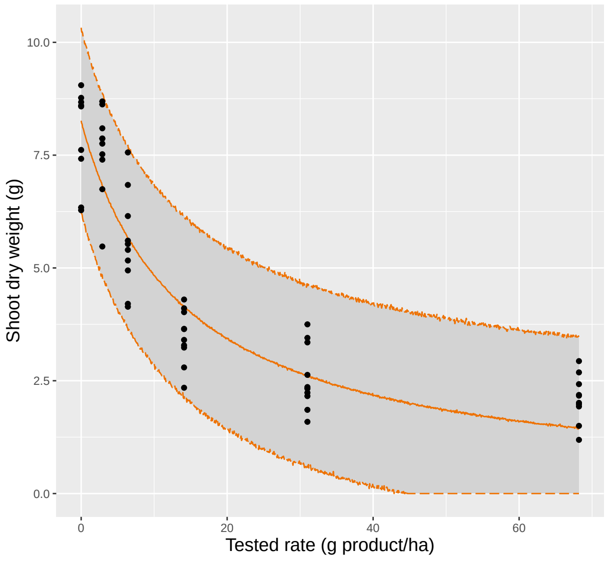

(a) Dose-response curve

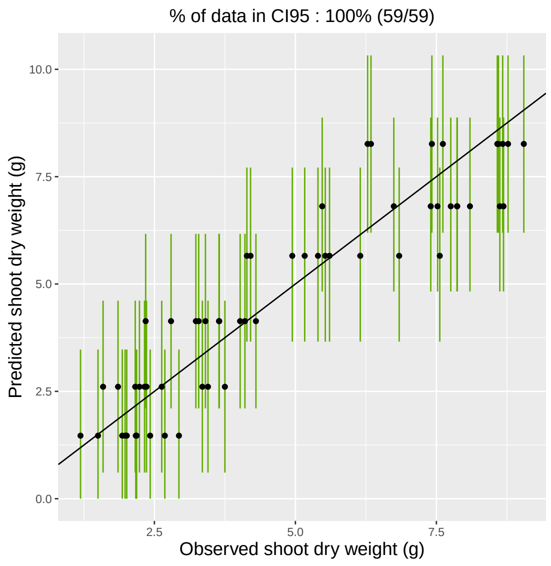

(b) Posterior predictive check (PPC)

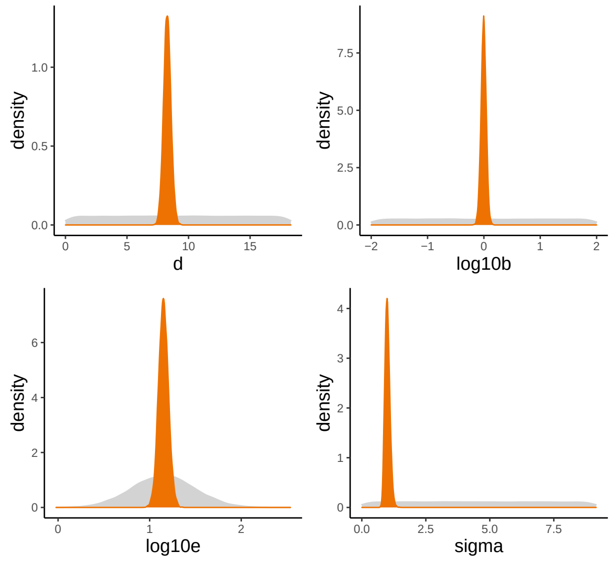

(c) Priors and posteriors

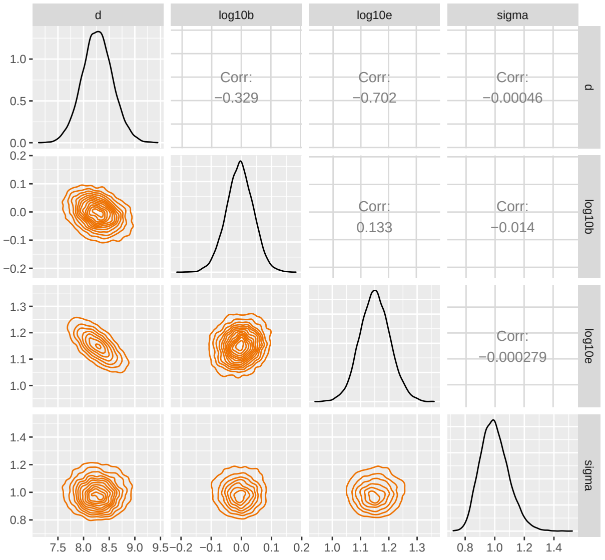

(d) Correlations between parameters

Figure 5: Dose-response curve (a), PPC (b), prior and posterior distributions (c) and correlations between parameters (d).

Data set: GLXMA_VV_weight

Table 6: Summary of parameter estimates for GLXMA_VV_weight data set

Parameter	median	Q2.5	Q97.5
b	1.432	1.219	1.703
d	5.129	4.870	5.400
e	14.249	12.358	16.367
sigma	0.470	0.395	0.573

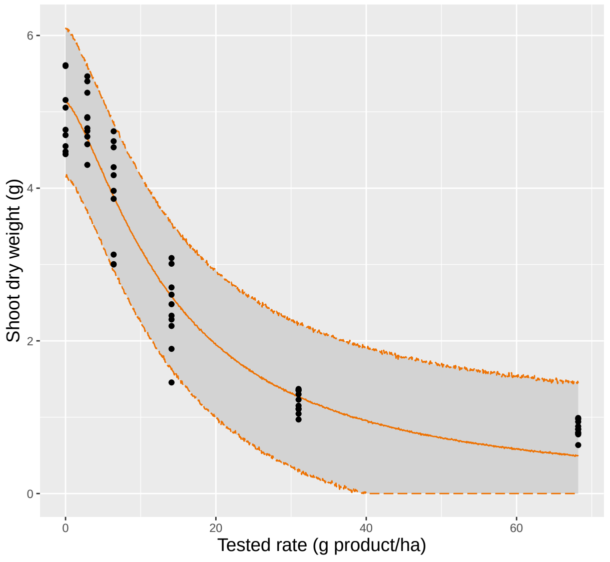

(a) Dose-response curve

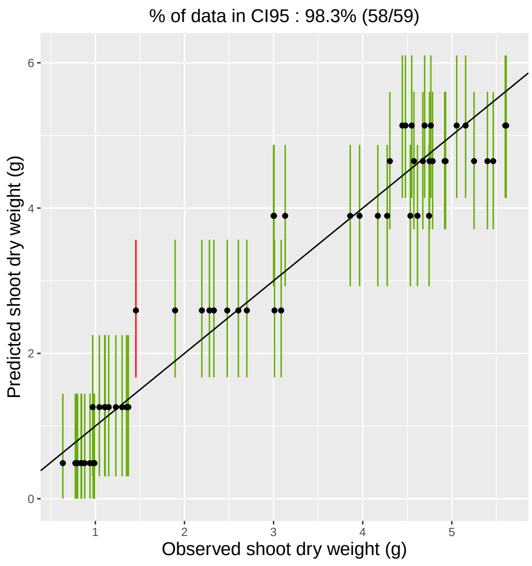

(b) Posterior predictive check (PPC)

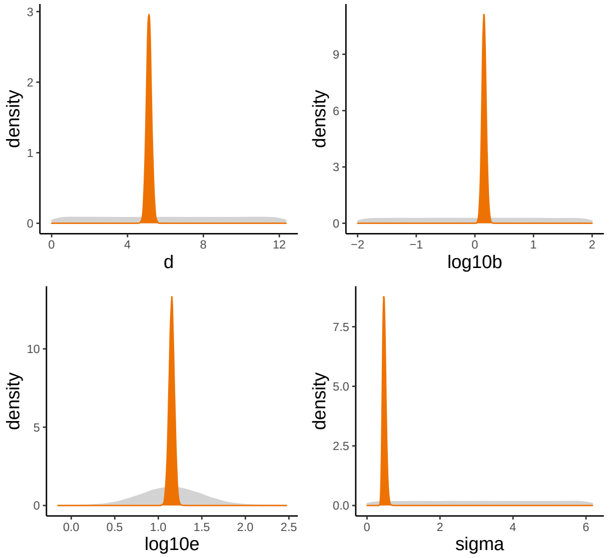

(c) Priors and posteriors

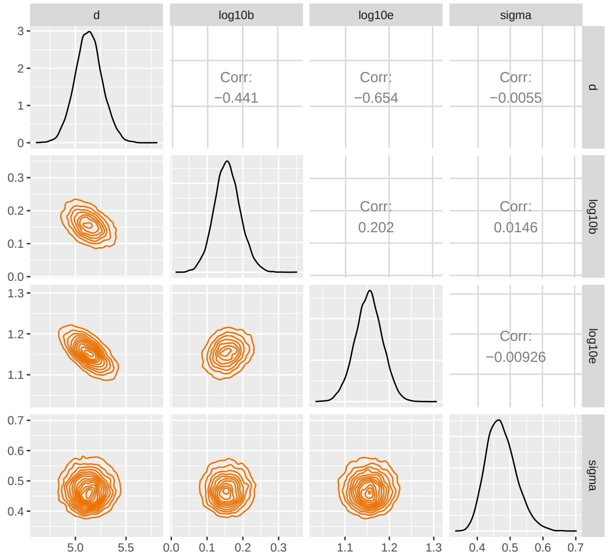

(d) Correlations between parameters

Figure 6: Dose-response curve (a), PPC (b), prior and posterior distributions (c) and correlations between parameters (d).

Data set: HELAN_VV_weight

Table 7: Summary of parameter estimates for HELAN_VV_weight data set

Parameter	median	Q2.5	Q97.5
b	5.860	3.602	46.854
d	2.651	2.562	2.751
e	12.793	11.729	13.931
sigma	0.217	0.175	0.278

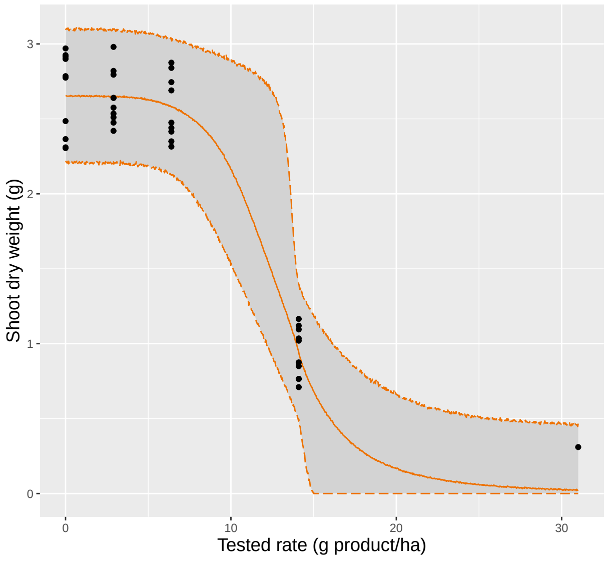

(a) Dose-response curve

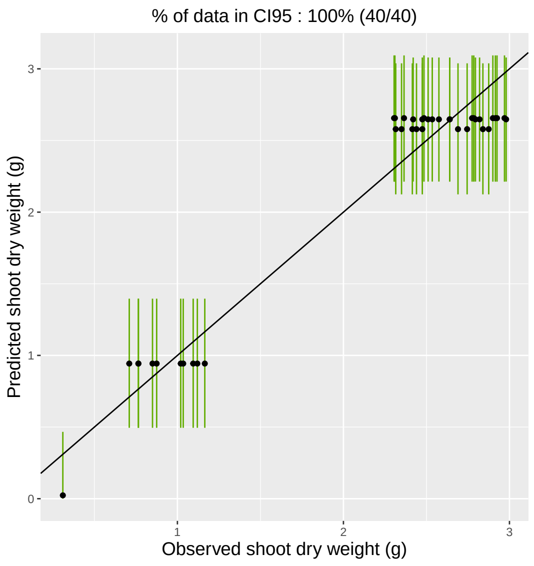

(b) Posterior predictive check (PPC)

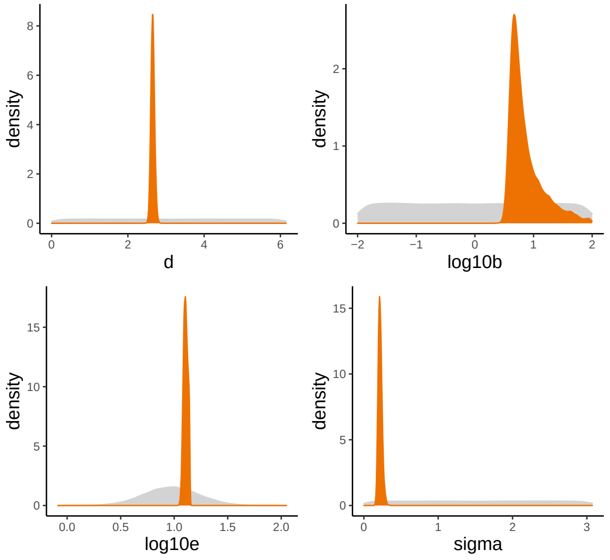

(c) Priors and posteriors

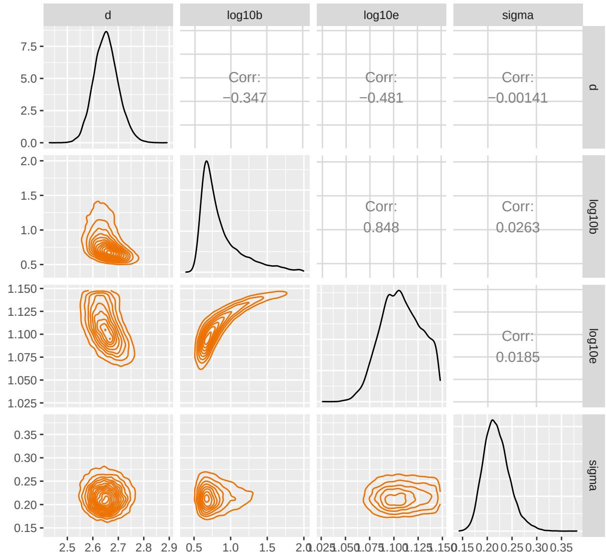

(d) Correlations between parameters

Figure 7: Dose-response curve (a), PPC (b), prior and posterior distributions (c) and correlations between parameters (d).

Data set: LYPES_VV_weight

Table 8: Summary of parameter estimates for LYPES_VV_weight data set

Parameter	median	Q2.5	Q97.5
b	1.394	1.165	1.686
d	4.492	4.218	4.767
e	10.757	9.141	12.670
sigma	0.476	0.400	0.583

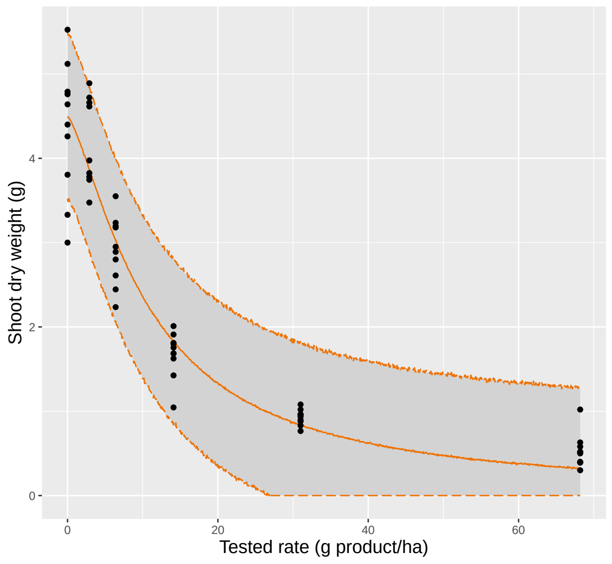

(a) Dose-response curve

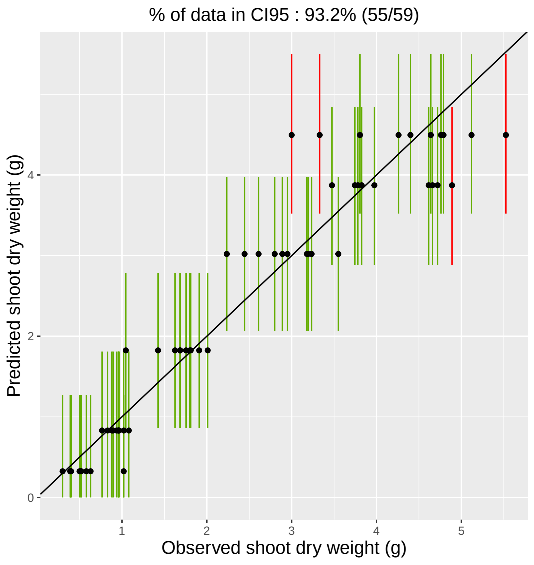

(b) Posterior predictive check (PPC)

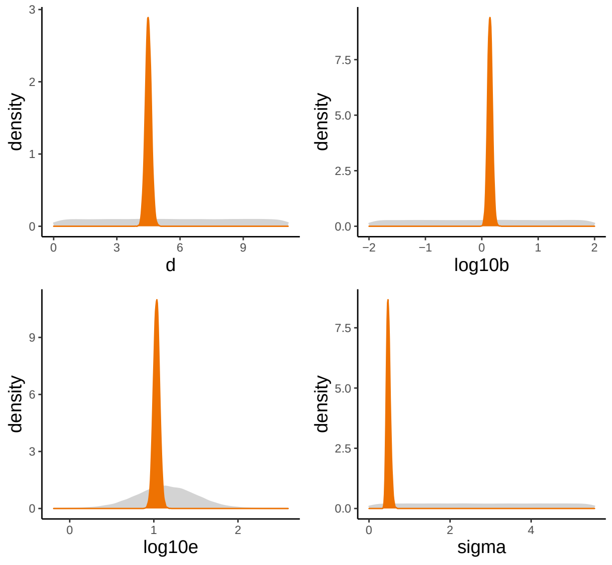

(c) Priors and posteriors

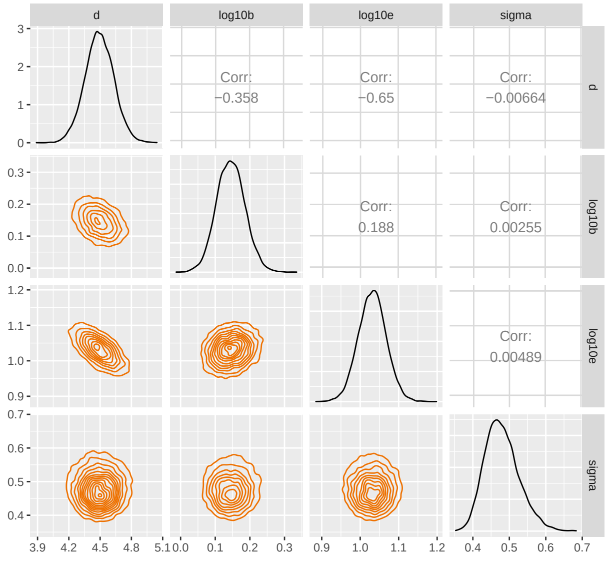

(d) Correlations between parameters

Figure 8: Dose-response curve (a), PPC (b), prior and posterior distributions (c) and correlations between parameters (d).

Data set: TRZAW_VV_weight

Table 9: Summary of parameter estimates for TRZAW_VV_weight data set

Parameter	median	Q2.5	Q97.5
b	2.985	2.185	4.139
d	1.293	1.233	1.353
e	248.882	219.795	281.520
sigma	0.121	0.094	0.165

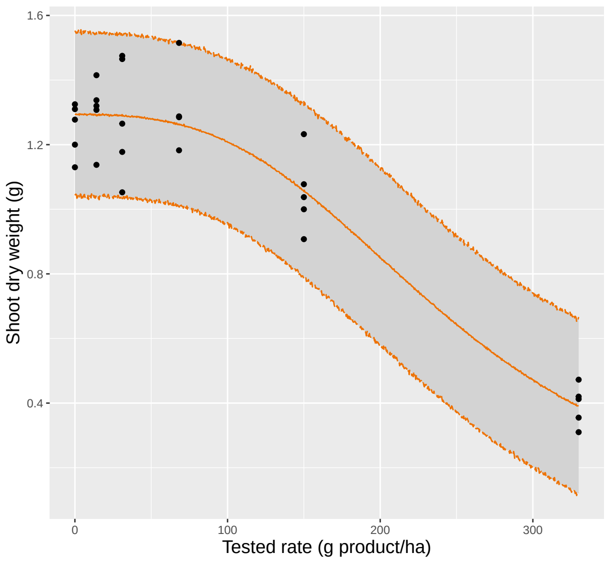

(a) Dose-response curve

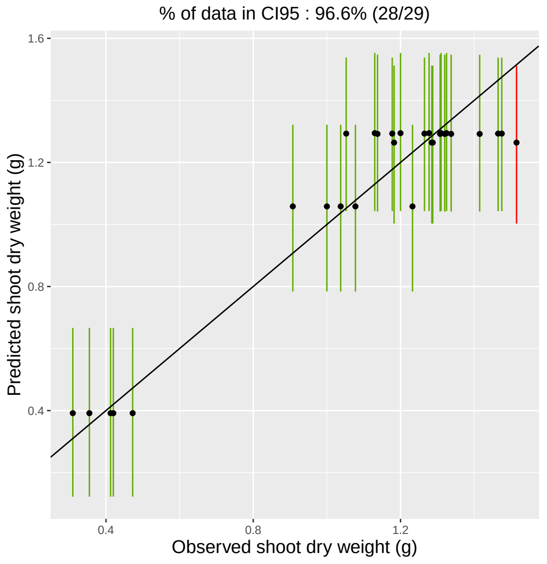

(b) Posterior predictive check (PPC)

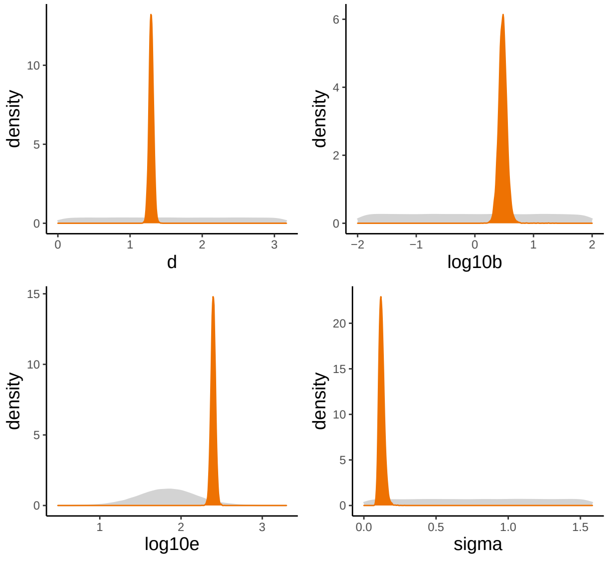

(c) Priors and posteriors

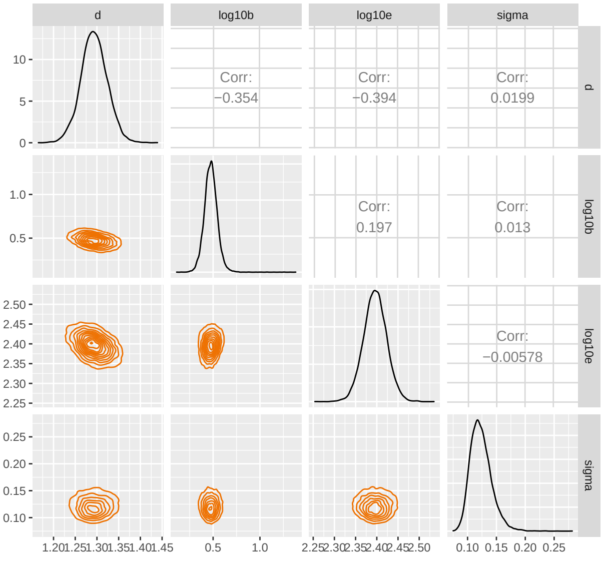

(d) Correlations between parameters

Figure 9: Dose-response curve (a), PPC (b), prior and posterior distributions (c) and correlations between parameters (d).

Data set: ZEAMA_VV_weight

Table 10: Summary of parameter estimates for ZEAMA_VV_weight data set

Parameter	median	Q2.5	Q97.5
b	3.503	2.595	4.869
d	7.719	7.367	8.077
e	213.231	189.844	240.396
sigma	1.040	0.875	1.269

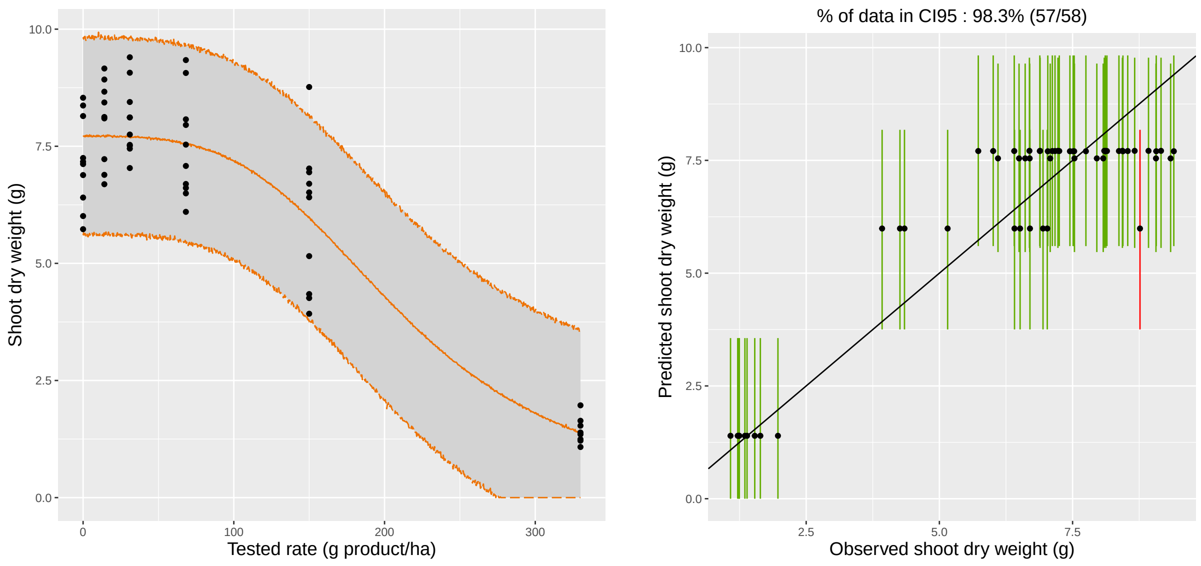

(a) Dose-response curve

(b) Posterior predictive check (PPC)

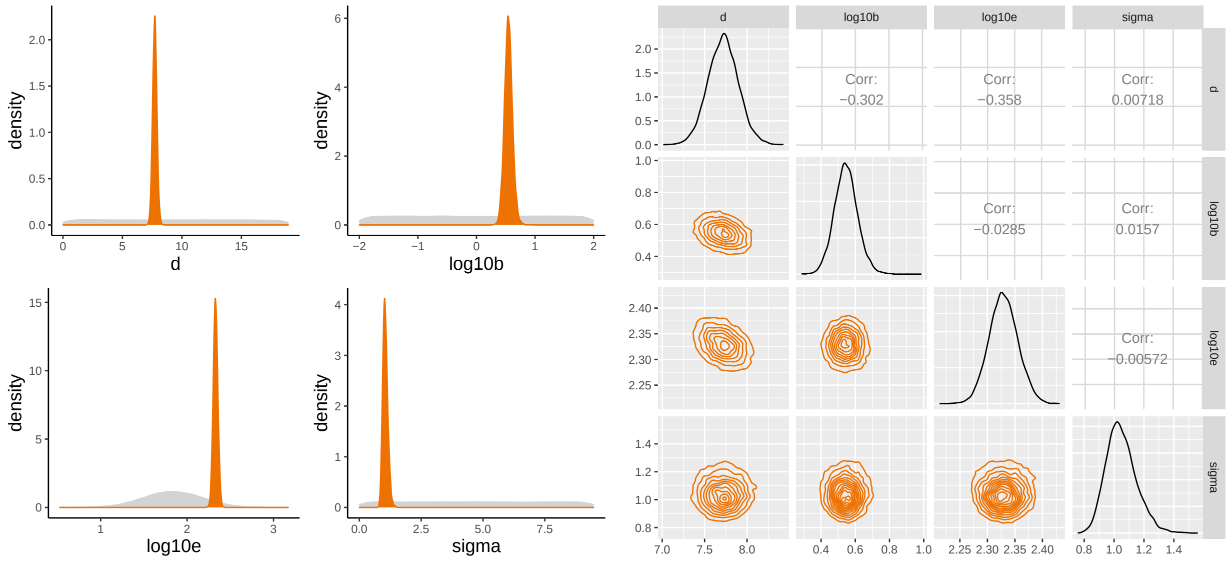

(c) Priors and posteriors

(d) Correlations between parameters

Figure 10: Dose-response curve (a), PPC (b), prior and posterior distributions (c) and correlations between parameters (d).
