# Supplementary material for: How to account for the uncertainty from standard toxicity tests in species sensitivity distributions: An example in non-target plants
Source: PLoS One. 2021 Jan 7;16(1):e0245071. doi: 10.1371/journal.pone.0245071 (PMC7790375; doi:10.1371/journal.pone.0245071)
Supplement: S1 Archive — It is a zip file containing seven folders (one folder per case study). Each folder contains five files report_xxx.pdf with detailed results of the dose-response analyses, one file corresponding to does-response analysis per endpoint. It also contains one file ER50_censoring.pdf for censored ER50 and one file SSD_analyses.pdf for results of SSD analyses. (ZIP) [file pone.0245071.s004.zip › S1_archive/Study6/SSD_analyses.pdf]

Species sensitivity distribution (SSD) analyses by fitting a log-normal distribution

Study 6

25 June 2020

Contact: sandrine.charles@univ-lyon1.fr

The document provides results on species sensitivity distribution (SSD) analyses for study 6. There are five endpoints for study 6: emergence, survival, shoot dry weight of the seedling emergence (SE) test, and survival and shoot dry weight of vegetative vigour (VV) test.

We used SSD to describe the toxicity variation of herbicides among a set of non-target terrestrial plants (NTTP). Based on this SSD, the hazardous rate at which five percent of the species are affected (HR_5) can be estimated. This document aims to compare SSD and HR_5 based on 50% effective rates (ER_{50}) generated in three different ways. We considered:

1. Point estimates (medians of posterior distributions of ER_{50}) as inputs for SSD analyses;
2. Interval-censored ER_{50} by 95% credible interval (CI95);
3. censored ER_{50} according to criterion 1 with a decision threshold T = 0.5

For each endpoint, a total of three SSD are produced:

1. SSD based on medians of posterior distributions of ER_{50} (figure title: median);
2. SSD based on CI95 of ER_{50} (figure title: CI95);
3. SSD based on censored ER_{50} according to criterion 1 with T = 0.5 (figure title: C1T0.5).

Meaning of symbols used in Tables 1-5:

- nb_ER_{50} (RC): number of ER_{50} (number of right-censored ER_{50});
- nb_distER_{50} (RC): number of distinct ER_{50} (number of distinct right-censored ER_{50});
- HR_5_est: HR_5 mean estimate;
- HR_5_median: median of HR_5;
- HR_5_LCoI95: lower bound of the 95% confidence interval (CoI95) of HR_5;
- HR_5_UCoI95: upper bound of the CoI95 of HR_5.

Meaning of legends, lines or curves in Figures 1-5:

- Nb. ER_{50} (RC): number of ER_{50} (number of right-censored ER_{50});
- Nb. distinct ER_{50} (RC): number of distinct ER_{50} (number of distinct right-censored ER_{50});
- HR_5 [CoI95]: HR_5 mean estimate [CoI95 of HR_5];
- Blue points: fractions of affected species (EPPO code) ordered by the median of ER_{50};
- Solid blue horizontal segment: interval-censored ER_{50} by the CI95 of the ER_{50};
- Dotted blue horizontal segment: right-censored ER_{50};
- Solid red curve: fitted SSD curve;
- Dotted red curves: CoI95 of the fitted SSD curve.

HR_5 results and SSD curves

HR_5 and SSD for the emergence endpiont of the SE test of study 6

Table 1: Number of (distinct) ER_{50} (right-censored) and HR_5 estimate for the emergence endpoint of the SE test of study 6 (tested rate unit: g product/ha)

criterion	nb_ER_{50} (RC)	nb_distER_{50} (RC)	HR_5_est	HR_5_median	HR_5_LCoI95	HR_5_UCoI95	CV
median	10 (0)	10 (0)	115.192	120.067	54.567	327.357	0.518
CI95	10 (0)	10 (0)	113.151	113.970	53.844	574.263	0.750
C1T0.5	10 (9)	3 (2)	86.468	86.505	47.411	3534.905	1.279

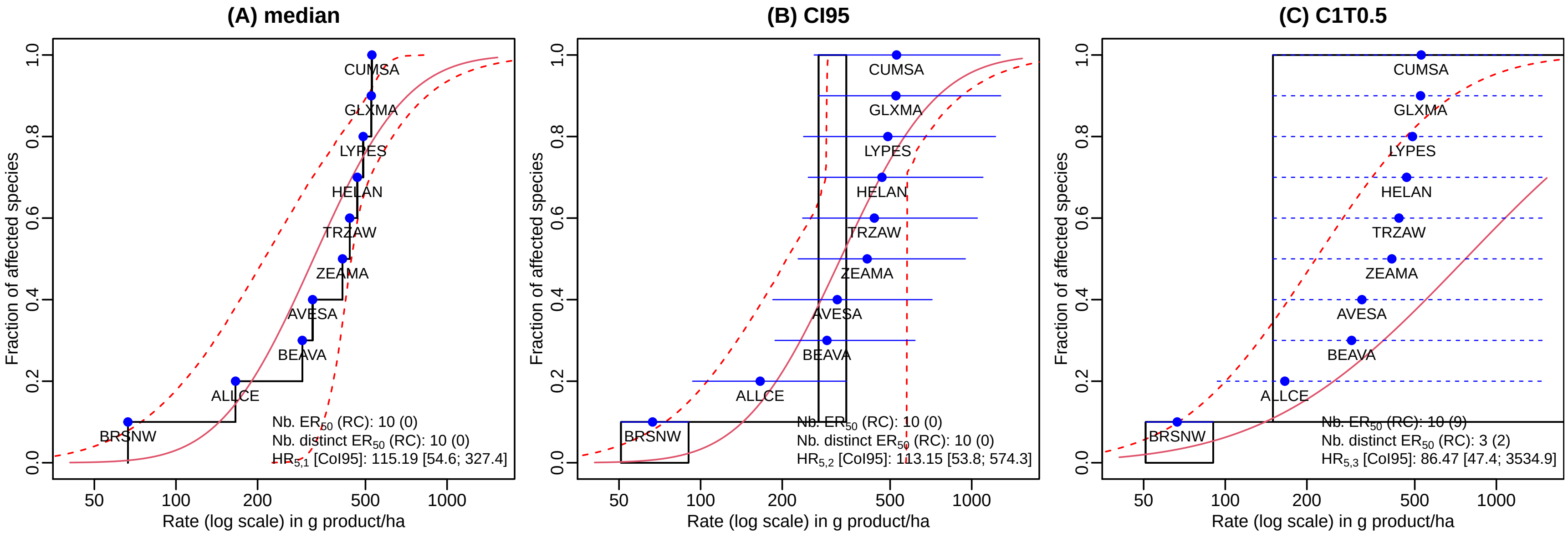

Figure 1: Example of three SSD based on respectively ER_{50} medians (A), CI95 of ER_{50} (B) and censored ER_{50} according to C1T0.5 (C) for the emergnece endpoint of the SE test of study 6.

HR_5 and SSD for the survival endpiont of the SE test of study 6

Table 2: Number of (distinct) ER_{50} (right-censored) and HR_5 estimate for the survival endpoint of the SE test of study 6 (tested rate unit: g product/ha)

criterion	nb_ER_{50} (RC)	nb_distER_{50} (RC)	HR_5_est	HR_5_median	HR_5_LCoI95	HR_5_UCoI95	CV
median	10 (0)	10 (0)	29.901	31.251	12.996	121.242	0.693
CI95	10 (0)	10 (0)	29.990	31.240	12.304	135.898	0.798
C1T0.5	10 (6)	6 (2)	21.325	22.787	8.864	133.913	2.145

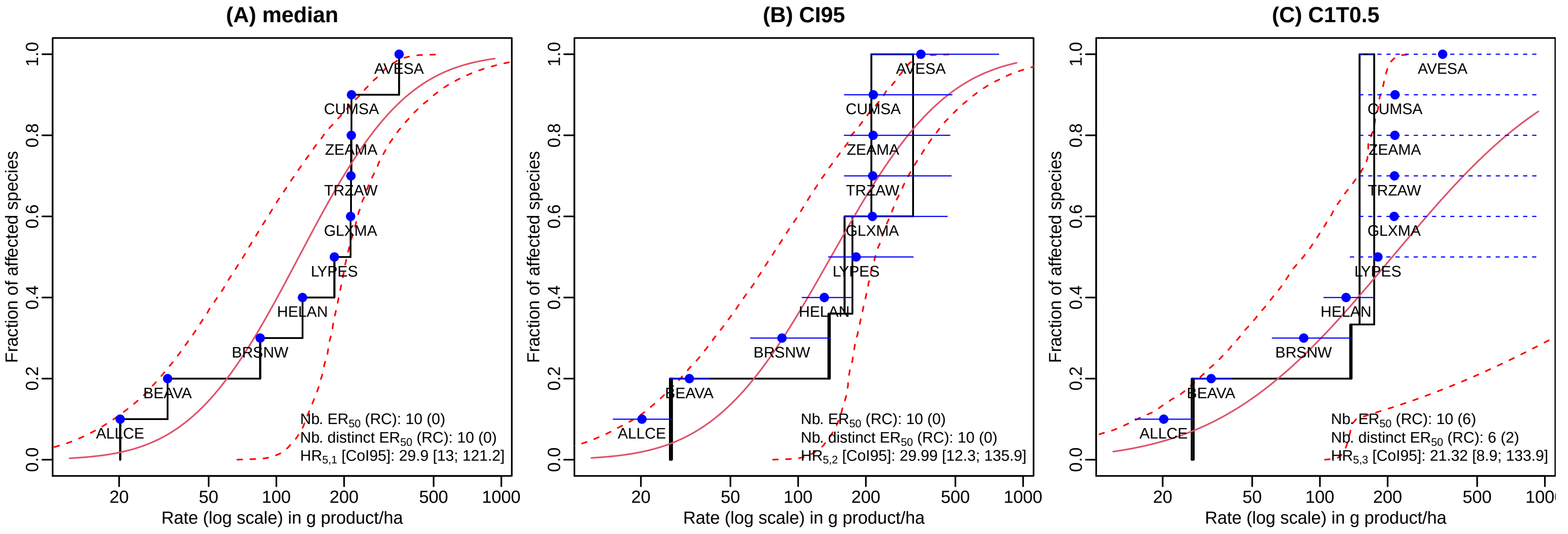

Figure 2: Example of three SSD based on respectively ER_{50} medians (A), CI95 of ER_{50} (B) and censored ER_{50} according to C1T0.5 (C) for the survival endpoint of the SE test of study 6.

HR_5 and SSD for the shoot dry weight endpiont of the SE test of study 6

Table 3: Number of (distinct) ER_{50} (right-censored) and HR_5 estimate for the shoot dry weight endpoint of the SE test of study 6 (tested rate unit: g product/ha)

criterion	nb_ER_{50} (RC)	nb_distER_{50} (RC)	HR_5_est	HR_5_median	HR_5_LCoI95	HR_5_UCoI95	CV
median	10 (0)	10 (0)	7.416	8.117	4.924	16.109	0.349
CI95	10 (0)	10 (0)	7.470	8.331	5.008	16.771	0.342
C1T0.5	10 (2)	10 (2)	6.499	7.075	3.960	14.159	0.355

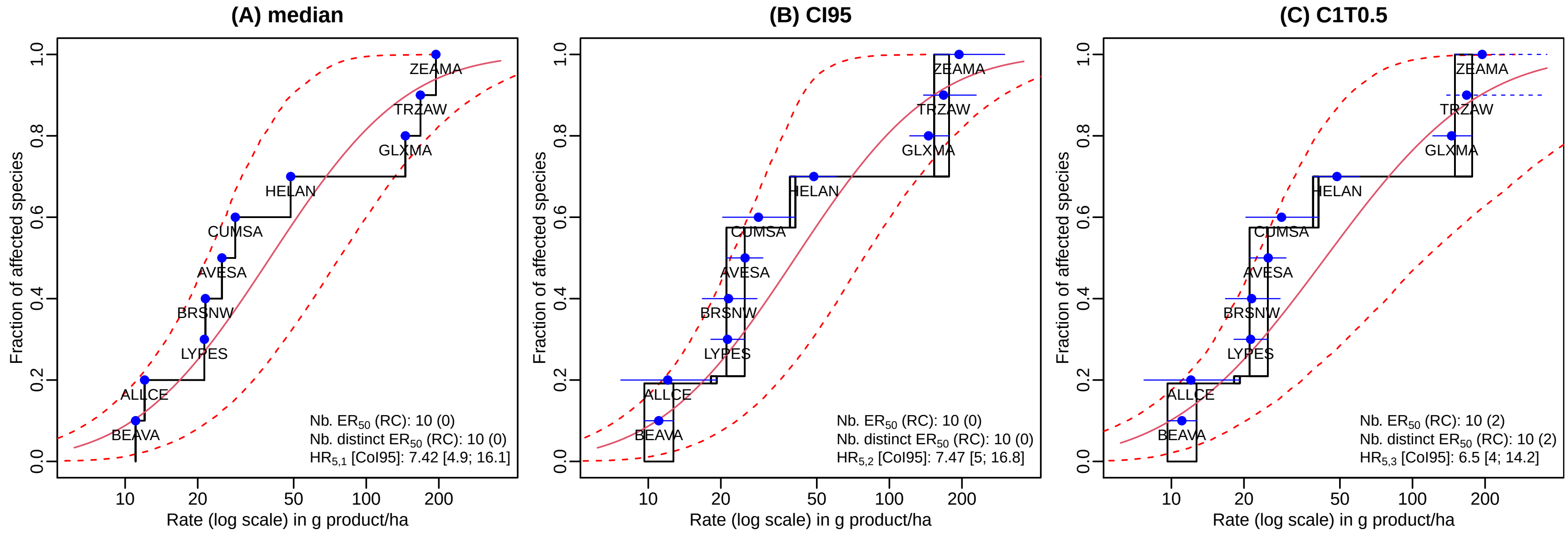

Figure 3: Example of three SSD based on respectively ER_{50} medians (A), CI95 of ER_{50} (B) and censored ER_{50} according to C1T0.5 (C) for the shoot dry weight endpoint of the SE test of study 6.

HR_5 and SSD for the survival endpiont of the VV test of study 6

Table 4: Number of (distinct) ER_{50} (right-censored) and HR_5 estimate for the survival endpoint of the VV test of study 6 (tested rate unit: g product/ha)

criterion	nb_ER_{50} (RC)	nb_distER_{50} (RC)	HR_5_est	HR_5_median	HR_5_LCoI95	HR_5_UCoI95	CV
median	10 (0)	10 (0)	22.503	24.721	14.426	43.684	0.294
CI95	10 (0)	10 (0)	22.535	24.944	13.187	50.846	0.365
C1T0.5	10 (6)	6 (2)	16.882	19.482	9.297	55.464	0.512

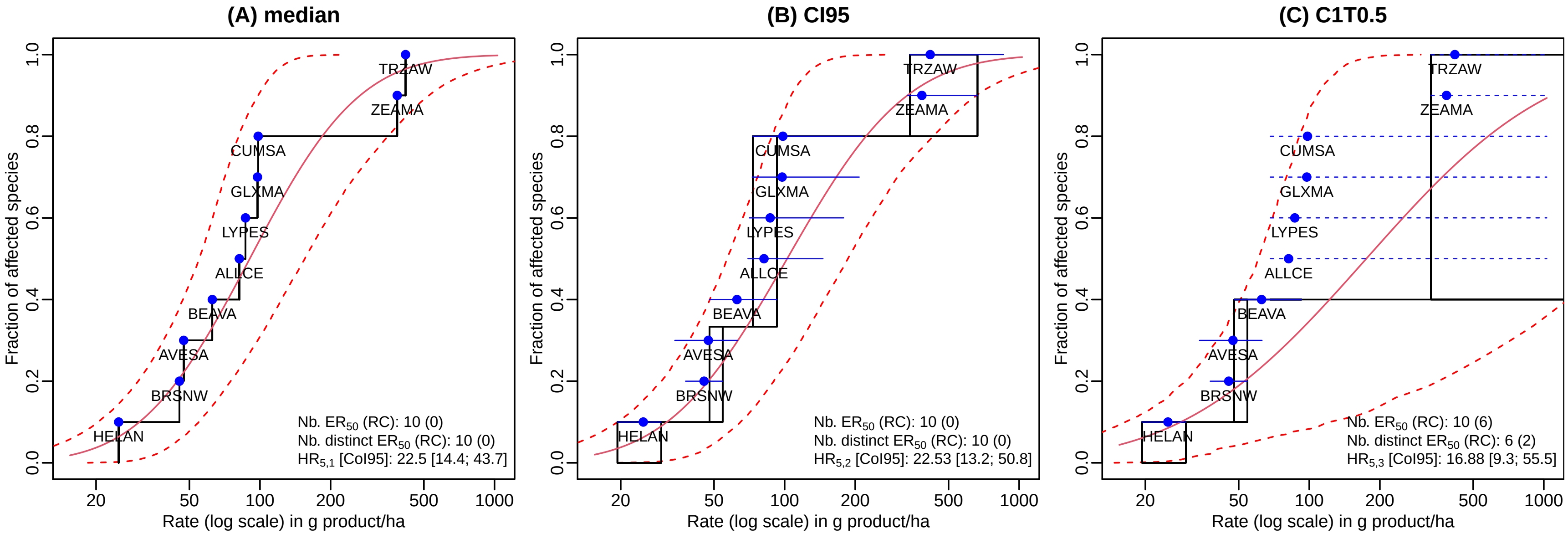

Figure 4: Example of three SSD based on respectively ER_{50} medians (A), CI95 of ER_{50} (B) and censored ER_{50} according to C1T0.5 (C) for the survival endpoint of the VV test of study 6.

HR_5 and SSD for the shoot dry weight endpiont of the VV test of study 6

Table 5: Number of (distinct) ER_{50} (right-censored) and HR_5 estimate for the shoot dry weight endpoint of the VV test of study 6 (tested rate unit: g product/ha)

criterion	nb_ER_{50} (RC)	nb_distER_{50} (RC)	HR_5_est	HR_5_median	HR_5_LCoI95	HR_5_UCoI95	CV
median	10 (0)	10 (0)	3.525	3.997	2.454	7.914	0.355
CI95	10 (0)	10 (0)	3.553	3.983	2.449	8.065	0.373
C1T0.5	10 (1)	10 (1)	3.487	3.948	2.414	8.764	0.408

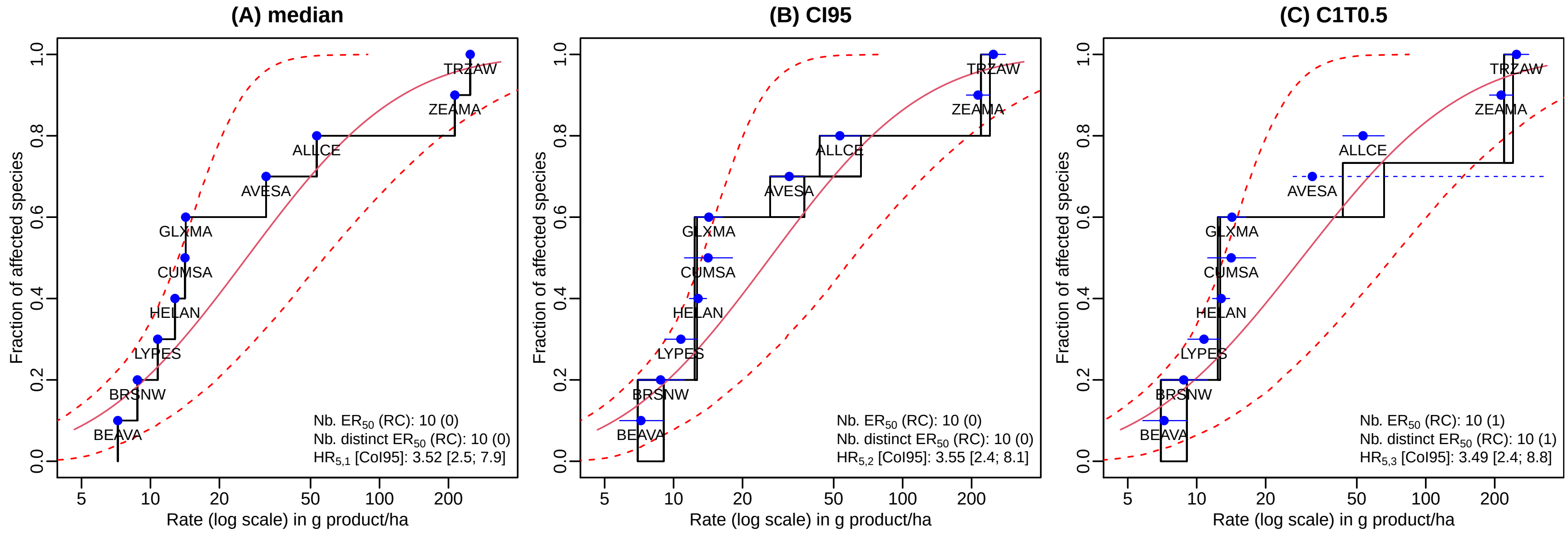

Figure 5: Example of three SSD based on respectively ER_{50} medians (A), CI95 of ER_{50} (B) and censored ER_{50} according to C1T0.5 (C) for the shoot dry weight endpoint of the VV test of study 6.
