# Supplementary material for: How to account for the uncertainty from standard toxicity tests in species sensitivity distributions: An example in non-target plants
Source: PLoS One. 2021 Jan 7;16(1):e0245071. doi: 10.1371/journal.pone.0245071 (PMC7790375; doi:10.1371/journal.pone.0245071)
Supplement: S1 Archive — It is a zip file containing seven folders (one folder per case study). Each folder contains five files report_xxx.pdf with detailed results of the dose-response analyses, one file corresponding to does-response analysis per endpoint. It also contains one file ER50_censoring.pdf for censored ER50 and one file SSD_analyses.pdf for results of SSD analyses. (ZIP) [file pone.0245071.s004.zip › S1_archive/Study7/ER50_censoring.pdf]

ER_{50} censoring after dose-response analyses

Study 7

25 June 2020

Contact: sandrine.charles@univ-lyon1.fr

This document provides the ER_{50} censoring after dose-response analyses for study 7, including four parts:

- Raw data description (test item rates);
- Summary on ER_{50} from a Bayesian dose-response fit;
- Censoring on ER_{50} according to two criteria;
  - Criterion 1 (C_1) based on the ratio of the probablities, that is the ratio of the probability that the ER_{50} lies within the range of the LCI95 and the maximum tested rate over the probability that the ER_{50} lies in the CI95;
  - Criterion 2 (C_2) based on the ratio of distances, that is the ratio of the distance between the LCI95 of ER_{50} and the maximum tested rate over the extend of the CI95 of ER_{50};
- Graphical presentation of censored ER_{50} per species per endpoint.

Meaning of symbols used in the document

- min_rate: minimum tested rate;
- max_rate: maximum tested rate;
- ER_{50}: 50% effective rate;
- CI95: 95% credible interval;
- ER_{50}_median: median of ER_{50};
- ER_{50}_LCI95: lower bound of the CI95 of ER_{50};
- ER_{50}_UCI95: upper bound of the CI95 of ER_{50};
- C_1: criterion based on the ratio of probabilities;
- C_2: criterion based on the ratio of distances;
- T: decision threshold;
- Censored ER_{50} [lower bound; upper bound]:
  - C1T0.0: censored ER_{50} according to C_1 with T = 0.0;
  - C1T0.2: censored ER_{50} according to C_1 with T = 0.2;

- C1T0.4: censored ER_{50} according to C_1 with T = 0.4;
- C1T0.5: censored ER_{50} according to C_1 with T = 0.5;
- C1T0.6: censored ER_{50} according to C_1 with T = 0.6;
- C1T0.8: censored ER_{50} according to C_1 with T = 0.8;
- C1T1.0: censored ER_{50} according to C_1 with T = 1.0;
- C2T0.0: censored ER_{50} according to C_2 with T = 0.0;
- C2T0.2: censored ER_{50} according to C_2 with T = 0.2;
- C2T0.4: censored ER_{50} according to C_2 with T = 0.4;
- C2T0.5: censored ER_{50} according to C_2 with T = 0.5;
- C2T0.6: censored ER_{50} according to C_2 with T = 0.6;
- C2T0.8: censored ER_{50} according to C_2 with T = 0.8;
- C2T1.0: censored ER_{50} according to C_2 with T = 1.0.

Seeding emergence (SE) test of study 7

Raw data description (test item rates) for the SE test

```
Number of species:  10
EPPO code:  ALLCE AVESA BEAVA BRSNW CUMSA GLXMA HELAN LYPES TRZAW ZEAMA
```

Table 1: Correspondance between species and common names according to the EPPO code

EPPO code	Species name	Common name
ALLCE	*Allium cepa*	Onion
AVESA	*Avena sativa*	Oat
BEAVA	*Beta vulgaris*	Sugar beet
BRSNW	*Brassica napus winter*	Oilseed rape
CUMSA	*Cucumis sativus*	Cucumber
GLXMA	*Glycine max*	Soybean
HELAN	*Helianthus annuus*	Sunflower
LYPES	*Solanum lycopersicum*	Tomato
TRZAW	*Triticum aestivum winter*	Winter wheat
ZEAMA	*Zea mays subs. Amylacea*	Corn

SE test - emergence endpoint

Table 2: Minimum and maximum tested rates, median, quantile 2.5% and quantile 97.5% of the ER_{50} posterior distribution - emergence endpoint of the SE test of study 7

species	min_rate	max_rate	ER_{50}_median	ER_{50}_LCI95	ER_{50}_UCI95
ALLCE	0.166	90.91	97.05953	45.11447	334.4929
AVESA	3.880	90.91	122.90510	94.92559	261.2516
BEAVA	0.802	90.91	445.00190	186.97293	1494.5358
BRSNW	3.880	90.91	95.13338	62.53236	181.6225
CUMSA	3.880	90.91	126.03630	95.62384	275.4094
GLXMA	3.880	90.91	265.74120	145.00580	634.7157
HELAN	3.880	90.91	264.95104	140.47192	626.0006
LYPES	3.880	90.91	128.14477	96.39124	291.5196
TRZAW	3.880	90.91	169.38203	113.75338	358.2504
ZEAMA	3.880	90.91	131.39009	97.14600	294.9957

Table 3: Censored ER_{50} according to criterion 1 and the seven decision thresholds - emergence endpoint of the SE test of study 7

species	C1T0.0	C1T0.2	C1T0.4	C1T0.5	C1T0.6	C1T0.8	C1T1.0
ALLCE	[45.11; 334.49]	[45.11; 334.49]	[45.11; 334.49]	[45.11; Inf)	[45.11; Inf)	[45.11; Inf)	[45.11; Inf)
AVESA	[90.91; Inf)	[90.91; Inf)	[90.91; Inf)	[90.91; Inf)	[90.91; Inf)	[90.91; Inf)	[90.91; Inf)
BEAVA	[90.91; Inf)	[90.91; Inf)	[90.91; Inf)	[90.91; Inf)	[90.91; Inf)	[90.91; Inf)	[90.91; Inf)
BRSNW	[62.53; 181.62]	[62.53; 181.62]	[62.53; 181.62]	[62.53; Inf)	[62.53; Inf)	[62.53; Inf)	[62.53; Inf)
CUMSA	[90.91; Inf)	[90.91; Inf)	[90.91; Inf)	[90.91; Inf)	[90.91; Inf)	[90.91; Inf)	[90.91; Inf)
GLXMA	[90.91; Inf)	[90.91; Inf)	[90.91; Inf)	[90.91; Inf)	[90.91; Inf)	[90.91; Inf)	[90.91; Inf)
HELAN	[90.91; Inf)	[90.91; Inf)	[90.91; Inf)	[90.91; Inf)	[90.91; Inf)	[90.91; Inf)	[90.91; Inf)
LYPES	[90.91; Inf)	[90.91; Inf)	[90.91; Inf)	[90.91; Inf)	[90.91; Inf)	[90.91; Inf)	[90.91; Inf)
TRZAW	[90.91; Inf)	[90.91; Inf)	[90.91; Inf)	[90.91; Inf)	[90.91; Inf)	[90.91; Inf)	[90.91; Inf)
ZEAMA	[90.91; Inf)	[90.91; Inf)	[90.91; Inf)	[90.91; Inf)	[90.91; Inf)	[90.91; Inf)	[90.91; Inf)

Table 4: Censored ER_{50} according to criterion 2 and the seven decision thresholds - emergence endpoint of the SE test of study 7

species	C2T0.0	C2T0.2	C2T0.4	C2T0.5	C2T0.6	C2T0.8	C2T1.0
ALLCE	[45.11; 334.49]	[45.11; Inf)	[45.11; Inf)	[45.11; Inf)	[45.11; Inf)	[45.11; Inf)	[45.11; Inf)
AVESA	[90.91; Inf)	[90.91; Inf)	[90.91; Inf)	[90.91; Inf)	[90.91; Inf)	[90.91; Inf)	[90.91; Inf)
BEAVA	[90.91; Inf)	[90.91; Inf)	[90.91; Inf)	[90.91; Inf)	[90.91; Inf)	[90.91; Inf)	[90.91; Inf)
BRSNW	[62.53; 181.62]	[62.53; 181.62]	[62.53; Inf)	[62.53; Inf)	[62.53; Inf)	[62.53; Inf)	[62.53; Inf)
CUMSA	[90.91; Inf)	[90.91; Inf)	[90.91; Inf)	[90.91; Inf)	[90.91; Inf)	[90.91; Inf)	[90.91; Inf)
GLXMA	[90.91; Inf)	[90.91; Inf)	[90.91; Inf)	[90.91; Inf)	[90.91; Inf)	[90.91; Inf)	[90.91; Inf)
HELAN	[90.91; Inf)	[90.91; Inf)	[90.91; Inf)	[90.91; Inf)	[90.91; Inf)	[90.91; Inf)	[90.91; Inf)
LYPES	[90.91; Inf)	[90.91; Inf)	[90.91; Inf)	[90.91; Inf)	[90.91; Inf)	[90.91; Inf)	[90.91; Inf)
TRZAW	[90.91; Inf)	[90.91; Inf)	[90.91; Inf)	[90.91; Inf)	[90.91; Inf)	[90.91; Inf)	[90.91; Inf)
ZEAMA	[90.91; Inf)	[90.91; Inf)	[90.91; Inf)	[90.91; Inf)	[90.91; Inf)	[90.91; Inf)	[90.91; Inf)

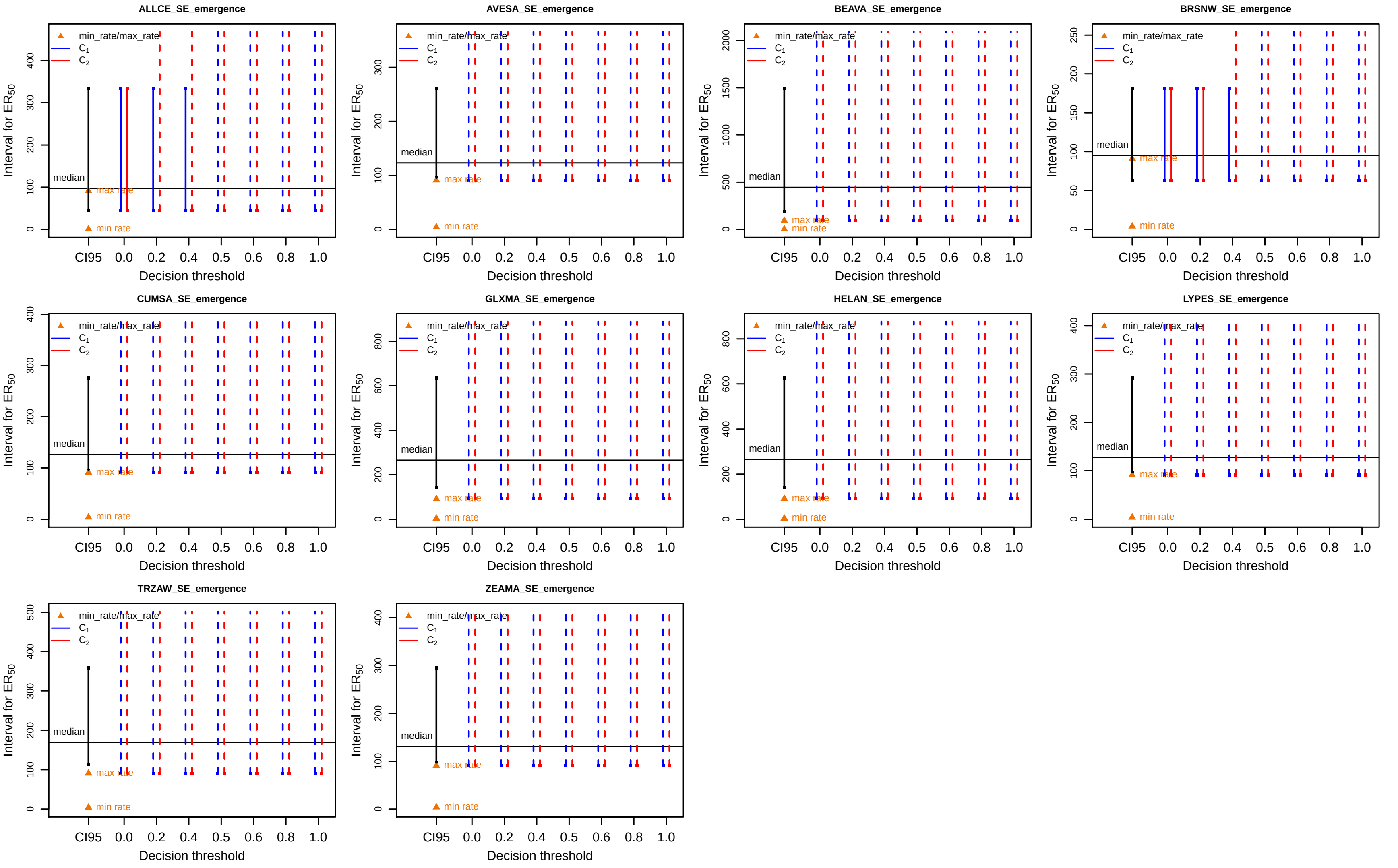

Figure 1: CI95 of ER_{50} and censored ER_{50} according to both criteria and the seven decision thresholds for all species - emergence endpoint of the SE test of study 7. The two orange triangles stand for the lowest and the highest tested rates. Vertical segments of three different colors (black, blue, red) represent the CI95 of ER_{50}, the censored ER_{50} according to C_1 and the censored ER_{50} according to C_2, respectively; solid vertical segments are for bounded intervals while dotted vertical segments stand for right-unbounded intervals; the black horizontal line represents the median of the ER_{50}.

SE test - survival endpoint

Table 5: Minimum and maximum tested rates, median, quantile 2.5% and quantile 97.5% of the ER_{50} posterior distribution - survival endpoint of the SE test of study 7

species	min_rate	max_rate	ER_{50}_median	ER_{50}_LCI95	ER_{50}_UCI95
ALLCE	0.166	90.91	2.689684	2.058483	3.46178
AVESA	3.880	90.91	97.441835	68.021285	172.49433
BEAVA	0.802	90.91	13.936858	11.068070	17.63860
BRSNW	3.880	90.91	56.653232	41.742047	83.63910
CUMSA	3.880	90.91	178.796740	107.109086	395.70705
GLXMA	3.880	90.91	187.844440	120.473427	419.63462
HELAN	3.880	90.91	78.272547	53.723540	130.92953
LYPES	3.880	90.91	88.004095	66.529442	133.26106
TRZAW	3.880	90.91	129.696489	97.384962	293.64758
ZEAMA	3.880	90.91	130.078055	97.205533	285.80160

Table 6: Censored ER_{50} according to criterion 1 and the seven decision thresholds - survival endpoint of the SE test of study 7

species	C1T0.0	C1T0.2	C1T0.4	C1T0.5	C1T0.6	C1T0.8	C1T1.0
ALLCE	[2.06; 3.46]	[2.06; 3.46]	[2.06; 3.46]	[2.06; 3.46]	[2.06; 3.46]	[2.06; 3.46]	[2.06; 3.46]
AVESA	[68.02; 172.49]	[68.02; 172.49]	[68.02; Inf)	[68.02; Inf)	[68.02; Inf)	[68.02; Inf)	[68.02; Inf)
BEAVA	[11.07; 17.64]	[11.07; 17.64]	[11.07; 17.64]	[11.07; 17.64]	[11.07; 17.64]	[11.07; 17.64]	[11.07; 17.64]
BRSNW	[41.74; 83.64]	[41.74; 83.64]	[41.74; 83.64]	[41.74; 83.64]	[41.74; 83.64]	[41.74; 83.64]	[41.74; 83.64]
CUMSA	[90.91; Inf)	[90.91; Inf)	[90.91; Inf)	[90.91; Inf)	[90.91; Inf)	[90.91; Inf)	[90.91; Inf)
GLXMA	[90.91; Inf)	[90.91; Inf)	[90.91; Inf)	[90.91; Inf)	[90.91; Inf)	[90.91; Inf)	[90.91; Inf)
HELAN	[53.72; 130.93]	[53.72; 130.93]	[53.72; 130.93]	[53.72; 130.93]	[53.72; 130.93]	[53.72; Inf)	[53.72; Inf)
LYPES	[66.53; 133.26]	[66.53; 133.26]	[66.53; 133.26]	[66.53; 133.26]	[66.53; Inf)	[66.53; Inf)	[66.53; Inf)
TRZAW	[90.91; Inf)	[90.91; Inf)	[90.91; Inf)	[90.91; Inf)	[90.91; Inf)	[90.91; Inf)	[90.91; Inf)
ZEAMA	[90.91; Inf)	[90.91; Inf)	[90.91; Inf)	[90.91; Inf)	[90.91; Inf)	[90.91; Inf)	[90.91; Inf)

Table 7: Censored ER_{50} according to criterion 2 and the seven decision thresholds - survival endpoint of the SE test of study 7

species	C2T0.0	C2T0.2	C2T0.4	C2T0.5	C2T0.6	C2T0.8	C2T1.0
ALLCE	[2.06; 3.46]	[2.06; 3.46]	[2.06; 3.46]	[2.06; 3.46]	[2.06; 3.46]	[2.06; 3.46]	[2.06; 3.46]
AVESA	[68.02; 172.49]	[68.02; 172.49]	[68.02; Inf)	[68.02; Inf)	[68.02; Inf)	[68.02; Inf)	[68.02; Inf)
BEAVA	[11.07; 17.64]	[11.07; 17.64]	[11.07; 17.64]	[11.07; 17.64]	[11.07; 17.64]	[11.07; 17.64]	[11.07; 17.64]
BRSNW	[41.74; 83.64]	[41.74; 83.64]	[41.74; 83.64]	[41.74; 83.64]	[41.74; 83.64]	[41.74; 83.64]	[41.74; 83.64]
CUMSA	[90.91; Inf)	[90.91; Inf)	[90.91; Inf)	[90.91; Inf)	[90.91; Inf)	[90.91; Inf)	[90.91; Inf)
GLXMA	[90.91; Inf)	[90.91; Inf)	[90.91; Inf)	[90.91; Inf)	[90.91; Inf)	[90.91; Inf)	[90.91; Inf)
HELAN	[53.72; 130.93]	[53.72; 130.93]	[53.72; 130.93]	[53.72; Inf)	[53.72; Inf)	[53.72; Inf)	[53.72; Inf)
LYPES	[66.53; 133.26]	[66.53; 133.26]	[66.53; Inf)	[66.53; Inf)	[66.53; Inf)	[66.53; Inf)	[66.53; Inf)
TRZAW	[90.91; Inf)	[90.91; Inf)	[90.91; Inf)	[90.91; Inf)	[90.91; Inf)	[90.91; Inf)	[90.91; Inf)
ZEAMA	[90.91; Inf)	[90.91; Inf)	[90.91; Inf)	[90.91; Inf)	[90.91; Inf)	[90.91; Inf)	[90.91; Inf)

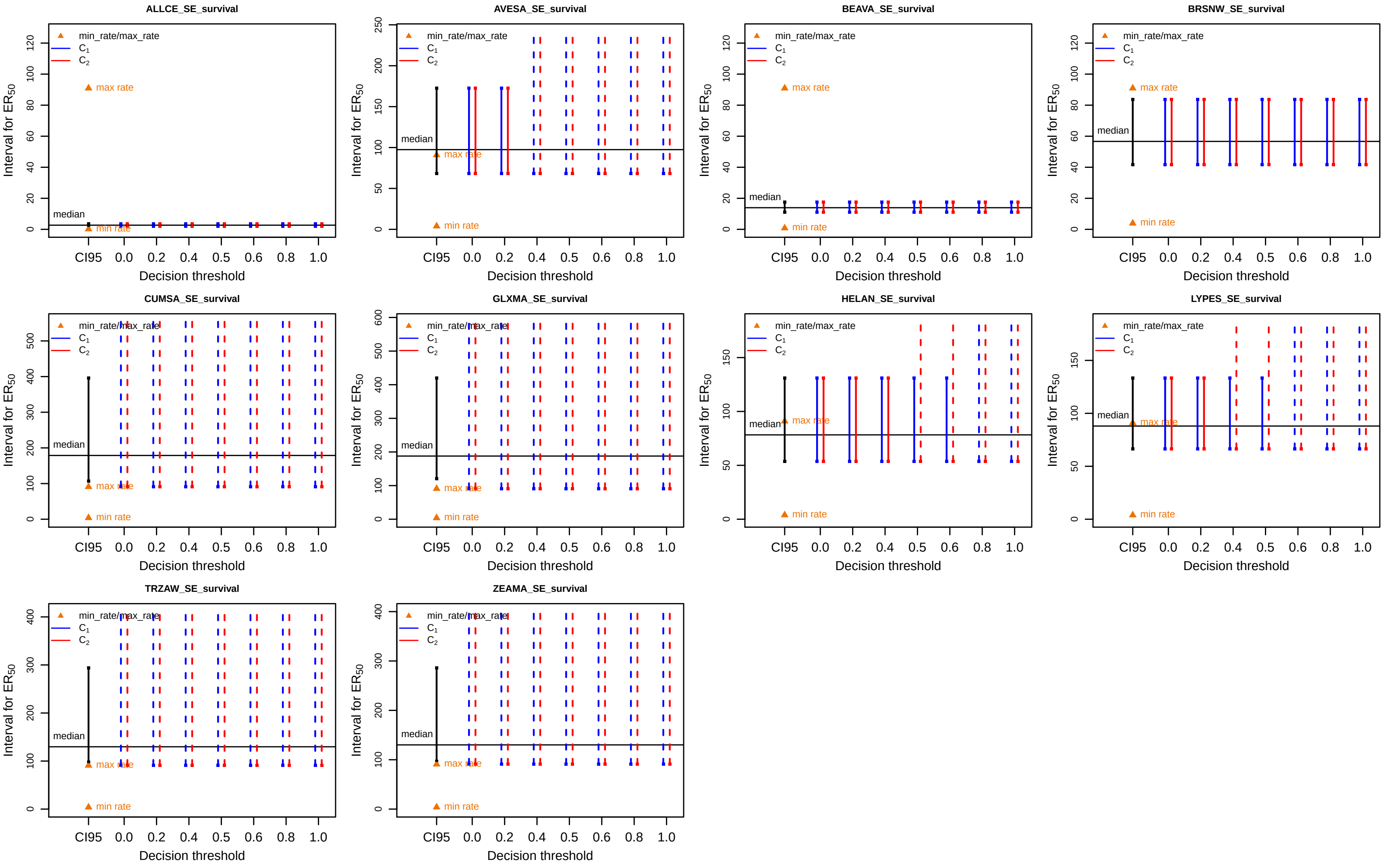

Figure 2: CI95 of ER_{50} and censored ER_{50} according to both criteria and the seven decision thresholds for all species - survival endpoint of the SE test of study 7. The two orange triangles stand for the lowest and the highest tested rates. Vertical segments of three different colors (black, blue, red) represent the CI95 of ER_{50}, the censored ER_{50} according to C_1 and the censored ER_{50} according to C_2, respectively; solid vertical segments are for bounded intervals while dotted vertical segments stand for right-unbounded intervals; the black horizontal line represents the median of the ER_{50}.

SE test - shoot dry weight endpoint

Table 8: Minimum and maximum tested rates, median, quantile 2.5% and quantile 97.5% of the ER_{50} posterior distribution - shoot dry weight endpoint of the SE test of study 7

species	min_rate	max_rate	ER_{50}_median	ER_{50}_LCI95	ER_{50}_UCI95
ALLCE	0.166	18.78	0.3620842	0.0997492	1.435263
AVESA	3.880	90.91	13.6036784	11.4052582	16.097591
BEAVA	0.802	41.32	3.9756174	2.5908858	5.886766
BRSNW	3.880	90.91	11.2371002	9.2720797	13.747939
CUMSA	3.880	90.91	9.2826393	7.0020399	12.336570
GLXMA	3.880	90.91	52.5459370	43.1427806	64.219082
HELAN	3.880	90.91	10.5857543	7.1429281	15.550129
LYPES	3.880	90.91	15.8588676	13.6473161	18.115954
TRZAW	3.880	90.91	110.8278310	81.1229476	164.913404
ZEAMA	3.880	90.91	37.5100755	29.2653647	48.304069

Table 9: Censored ER_{50} according to criterion 1 and the seven decision thresholds - shoot dry weight endpoint of the SE test of study 7

species	C1T0.0	C1T0.2	C1T0.4	C1T0.5	C1T0.6	C1T0.8	C1T1.0
ALLCE	[0.1; 1.44]	[0.1; 1.44]	[0.1; 1.44]	[0.1; 1.44]	[0.1; 1.44]	[0.1; 1.44]	[0.1; 1.44]
AVESA	[11.41; 16.1]	[11.41; 16.1]	[11.41; 16.1]	[11.41; 16.1]	[11.41; 16.1]	[11.41; 16.1]	[11.41; 16.1]
BEAVA	[2.59; 5.89]	[2.59; 5.89]	[2.59; 5.89]	[2.59; 5.89]	[2.59; 5.89]	[2.59; 5.89]	[2.59; 5.89]
BRSNW	[9.27; 13.75]	[9.27; 13.75]	[9.27; 13.75]	[9.27; 13.75]	[9.27; 13.75]	[9.27; 13.75]	[9.27; 13.75]
CUMSA	[7; 12.34]	[7; 12.34]	[7; 12.34]	[7; 12.34]	[7; 12.34]	[7; 12.34]	[7; 12.34]
GLXMA	[43.14; 64.22]	[43.14; 64.22]	[43.14; 64.22]	[43.14; 64.22]	[43.14; 64.22]	[43.14; 64.22]	[43.14; 64.22]
HELAN	[7.14; 15.55]	[7.14; 15.55]	[7.14; 15.55]	[7.14; 15.55]	[7.14; 15.55]	[7.14; 15.55]	[7.14; 15.55]
LYPES	[13.65; 18.12]	[13.65; 18.12]	[13.65; 18.12]	[13.65; 18.12]	[13.65; 18.12]	[13.65; 18.12]	[13.65; 18.12]
TRZAW	[81.12; 164.91]	[81.12; Inf)	[81.12; Inf)	[81.12; Inf)	[81.12; Inf)	[81.12; Inf)	[81.12; Inf)
ZEAMA	[29.27; 48.3]	[29.27; 48.3]	[29.27; 48.3]	[29.27; 48.3]	[29.27; 48.3]	[29.27; 48.3]	[29.27; 48.3]

Table 10: Censored ER_{50} according to criterion 2 and the seven decision thresholds - shoot dry weight endpoint of the SE test of study 7

species	C2T0.0	C2T0.2	C2T0.4	C2T0.5	C2T0.6	C2T0.8	C2T1.0
ALLCE	[0.1; 1.44]	[0.1; 1.44]	[0.1; 1.44]	[0.1; 1.44]	[0.1; 1.44]	[0.1; 1.44]	[0.1; 1.44]
AVESA	[11.41; 16.1]	[11.41; 16.1]	[11.41; 16.1]	[11.41; 16.1]	[11.41; 16.1]	[11.41; 16.1]	[11.41; 16.1]
BEAVA	[2.59; 5.89]	[2.59; 5.89]	[2.59; 5.89]	[2.59; 5.89]	[2.59; 5.89]	[2.59; 5.89]	[2.59; 5.89]
BRSNW	[9.27; 13.75]	[9.27; 13.75]	[9.27; 13.75]	[9.27; 13.75]	[9.27; 13.75]	[9.27; 13.75]	[9.27; 13.75]
CUMSA	[7; 12.34]	[7; 12.34]	[7; 12.34]	[7; 12.34]	[7; 12.34]	[7; 12.34]	[7; 12.34]
GLXMA	[43.14; 64.22]	[43.14; 64.22]	[43.14; 64.22]	[43.14; 64.22]	[43.14; 64.22]	[43.14; 64.22]	[43.14; 64.22]
HELAN	[7.14; 15.55]	[7.14; 15.55]	[7.14; 15.55]	[7.14; 15.55]	[7.14; 15.55]	[7.14; 15.55]	[7.14; 15.55]
LYPES	[13.65; 18.12]	[13.65; 18.12]	[13.65; 18.12]	[13.65; 18.12]	[13.65; 18.12]	[13.65; 18.12]	[13.65; 18.12]
TRZAW	[81.12; 164.91]	[81.12; Inf)	[81.12; Inf)	[81.12; Inf)	[81.12; Inf)	[81.12; Inf)	[81.12; Inf)
ZEAMA	[29.27; 48.3]	[29.27; 48.3]	[29.27; 48.3]	[29.27; 48.3]	[29.27; 48.3]	[29.27; 48.3]	[29.27; 48.3]

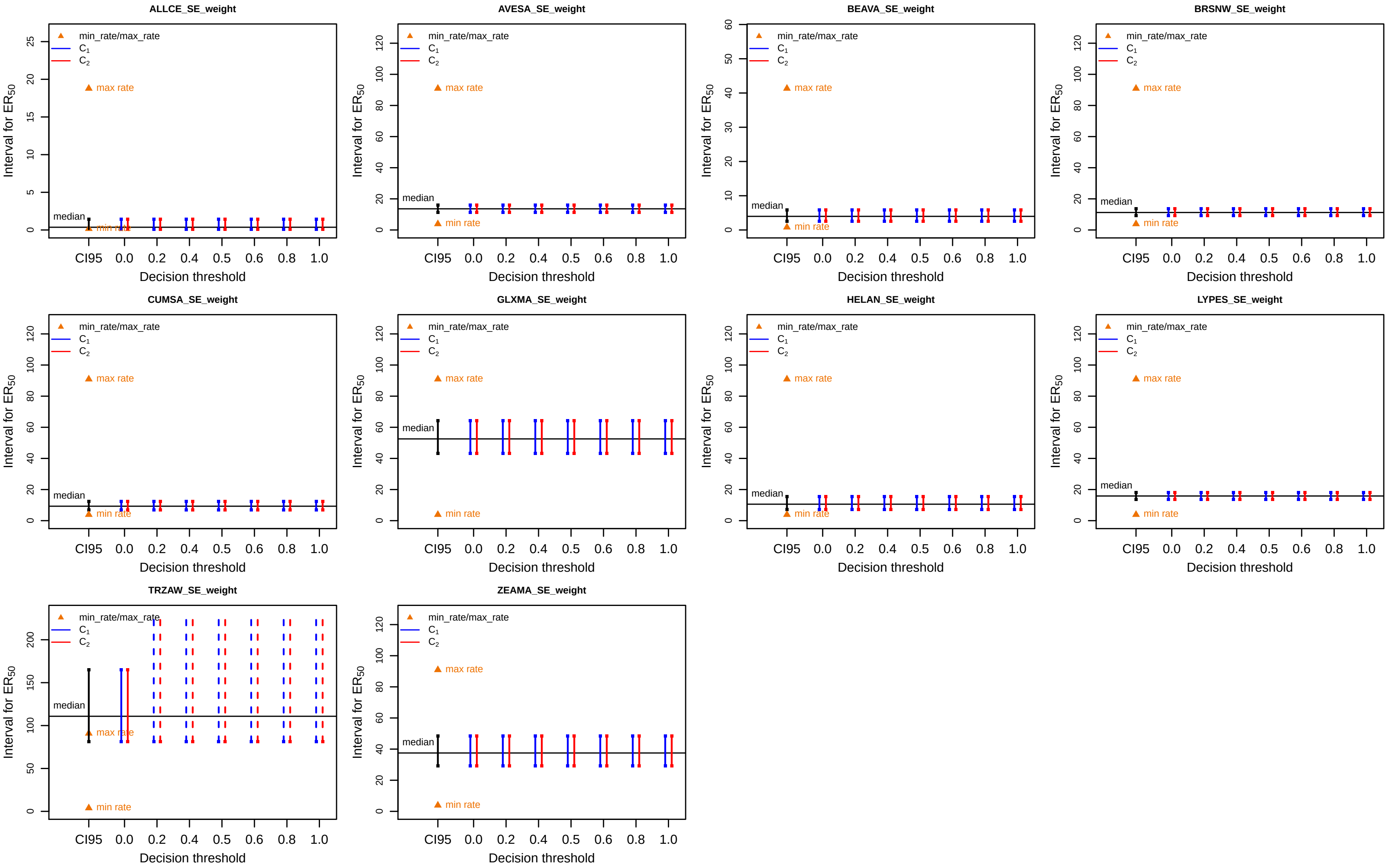

Figure 3: CI95 of ER_{50} and censored ER_{50} according to both criteria and the seven decision thresholds for all species - shoot dry weight endpoint of the SE test of study 7. The two orange triangles stand for the lowest and the highest tested rates. Vertical segments of three different colors (black, blue, red) represent the CI95 of ER_{50}, the censored ER_{50} according to C_1 and the censored ER_{50} according to C_2, respectively; solid vertical segments are for bounded intervals while dotted vertical segments stand for right-unbounded intervals; the black horizontal line represents the median of the ER_{50}.

Vegatative vigor (VV) test of study 7

Raw data description (test item rates) for VV test

```
Number of species:  10
EPPO code:  ALLCE AVESA BEAVA BRSNW CUMSA GLXMA HELAN LYPES TRZAW ZEAMA
```

Table 11: Corresponding species and common names to the EPPO code

EPPO code	Species name	Common name
ALLCE	*Allium cepa*	Onion
AVESA	*Avena sativa*	Oat
BEAVA	*Beta vulgaris*	Sugar beet
BRSNW	*Brassica napus winter*	Oilseed rape
CUMSA	*Cucumis sativus*	Cucumber
GLXMA	*Glycine max*	Soybean
HELAN	*Helianthus annuus*	Sunflower
LYPES	*Solanum lycopersicum*	Tomato
TRZAW	*Triticum aestivum winter*	Winter wheat
ZEAMA	*Zea mays subs. Amylacea*	Corn

Table 12: Tested rates per species (g product/ha) for the VV test of study 7

ALLCE	AVESA	BEAVA	BRSNW	CUMSA	GLXMA	HELAN	LYPES	TRZAW	ZEAMA
0.00	0.00	0.000	0.000	0.00	0.00	0.00	0.00	0.00	0.00
1.76	1.76	0.364	0.364	1.76	3.88	1.76	1.76	3.88	3.88
3.88	3.88	0.802	0.802	3.88	8.54	3.88	3.88	8.54	8.54
8.54	8.54	1.760	1.760	8.54	18.78	8.54	8.54	18.78	18.78
18.78	18.78	3.880	3.880	18.78	41.32	18.78	18.78	41.32	41.32
41.32	41.32	8.540	8.540	41.32	90.91	41.32	41.32	90.91	90.91

VV test - survival endpoint

Table 13: Minimum and maximum tested rates, median, quantile 2.5% and quantile 97.5% of the ER_{50} posterior distribution - survival endpoint of the VV test of study 7

species	min_rate	max_rate	ER_{50}_median	ER_{50}_LCI95	ER_{50}_UCI95
ALLCE	1.760	41.32	46.641646	41.601541	75.843785
AVESA	1.760	41.32	49.473009	42.230466	88.991649
BEAVA	0.364	8.54	7.085982	5.837452	8.589002
BRSNW	0.364	8.54	9.932272	8.673981	16.546308
CUMSA	1.760	41.32	38.936929	32.401060	42.706934
GLXMA	3.880	90.91	129.998101	97.333796	294.161230
HELAN	1.760	41.32	8.807486	8.055439	10.279506
LYPES	1.760	41.32	36.152426	28.797941	47.938910
TRZAW	3.880	90.91	129.113217	97.423971	283.965460
ZEAMA	3.880	90.91	130.044482	97.498618	281.766364

Table 14: Censored ER_{50} according to criterion 1 and the seven decision thresholds - survival endpoint of the VV test of study 7

species	C1T0.0	C1T0.2	C1T0.4	C1T0.5	C1T0.6	C1T0.8	C1T1.0
ALLCE	[41.32; Inf)	[41.32; Inf)	[41.32; Inf)	[41.32; Inf)	[41.32; Inf)	[41.32; Inf)	[41.32; Inf)
AVESA	[41.32; Inf)	[41.32; Inf)	[41.32; Inf)	[41.32; Inf)	[41.32; Inf)	[41.32; Inf)	[41.32; Inf)
BEAVA	[5.84; 8.59]	[5.84; 8.59]	[5.84; 8.59]	[5.84; 8.59]	[5.84; 8.59]	[5.84; 8.59]	[5.84; Inf)
BRSNW	[8.54; Inf)	[8.54; Inf)	[8.54; Inf)	[8.54; Inf)	[8.54; Inf)	[8.54; Inf)	[8.54; Inf)
CUMSA	[32.4; 42.71]	[32.4; 42.71]	[32.4; 42.71]	[32.4; 42.71]	[32.4; 42.71]	[32.4; 42.71]	[32.4; Inf)
GLXMA	[90.91; Inf)	[90.91; Inf)	[90.91; Inf)	[90.91; Inf)	[90.91; Inf)	[90.91; Inf)	[90.91; Inf)
HELAN	[8.06; 10.28]	[8.06; 10.28]	[8.06; 10.28]	[8.06; 10.28]	[8.06; 10.28]	[8.06; 10.28]	[8.06; 10.28]
LYPES	[28.8; 47.94]	[28.8; 47.94]	[28.8; 47.94]	[28.8; 47.94]	[28.8; 47.94]	[28.8; 47.94]	[28.8; Inf)
TRZAW	[90.91; Inf)	[90.91; Inf)	[90.91; Inf)	[90.91; Inf)	[90.91; Inf)	[90.91; Inf)	[90.91; Inf)
ZEAMA	[90.91; Inf)	[90.91; Inf)	[90.91; Inf)	[90.91; Inf)	[90.91; Inf)	[90.91; Inf)	[90.91; Inf)

Table 15: Censored ER_{50} according to criterion 2 and the seven decision thresholds - survival endpoint of the VV test of study 7

species	C2T0.0	C2T0.2	C2T0.4	C2T0.5	C2T0.6	C2T0.8	C2T1.0
ALLCE	[41.32; Inf)	[41.32; Inf)	[41.32; Inf)	[41.32; Inf)	[41.32; Inf)	[41.32; Inf)	[41.32; Inf)
AVESA	[41.32; Inf)	[41.32; Inf)	[41.32; Inf)	[41.32; Inf)	[41.32; Inf)	[41.32; Inf)	[41.32; Inf)
BEAVA	[5.84; 8.59]	[5.84; 8.59]	[5.84; 8.59]	[5.84; 8.59]	[5.84; 8.59]	[5.84; 8.59]	[5.84; Inf)
BRSNW	[8.54; Inf)	[8.54; Inf)	[8.54; Inf)	[8.54; Inf)	[8.54; Inf)	[8.54; Inf)	[8.54; Inf)
CUMSA	[32.4; 42.71]	[32.4; 42.71]	[32.4; 42.71]	[32.4; 42.71]	[32.4; 42.71]	[32.4; 42.71]	[32.4; Inf)
GLXMA	[90.91; Inf)	[90.91; Inf)	[90.91; Inf)	[90.91; Inf)	[90.91; Inf)	[90.91; Inf)	[90.91; Inf)
HELAN	[8.06; 10.28]	[8.06; 10.28]	[8.06; 10.28]	[8.06; 10.28]	[8.06; 10.28]	[8.06; 10.28]	[8.06; 10.28]
LYPES	[28.8; 47.94]	[28.8; 47.94]	[28.8; 47.94]	[28.8; 47.94]	[28.8; 47.94]	[28.8; Inf)	[28.8; Inf)
TRZAW	[90.91; Inf)	[90.91; Inf)	[90.91; Inf)	[90.91; Inf)	[90.91; Inf)	[90.91; Inf)	[90.91; Inf)
ZEAMA	[90.91; Inf)	[90.91; Inf)	[90.91; Inf)	[90.91; Inf)	[90.91; Inf)	[90.91; Inf)	[90.91; Inf)

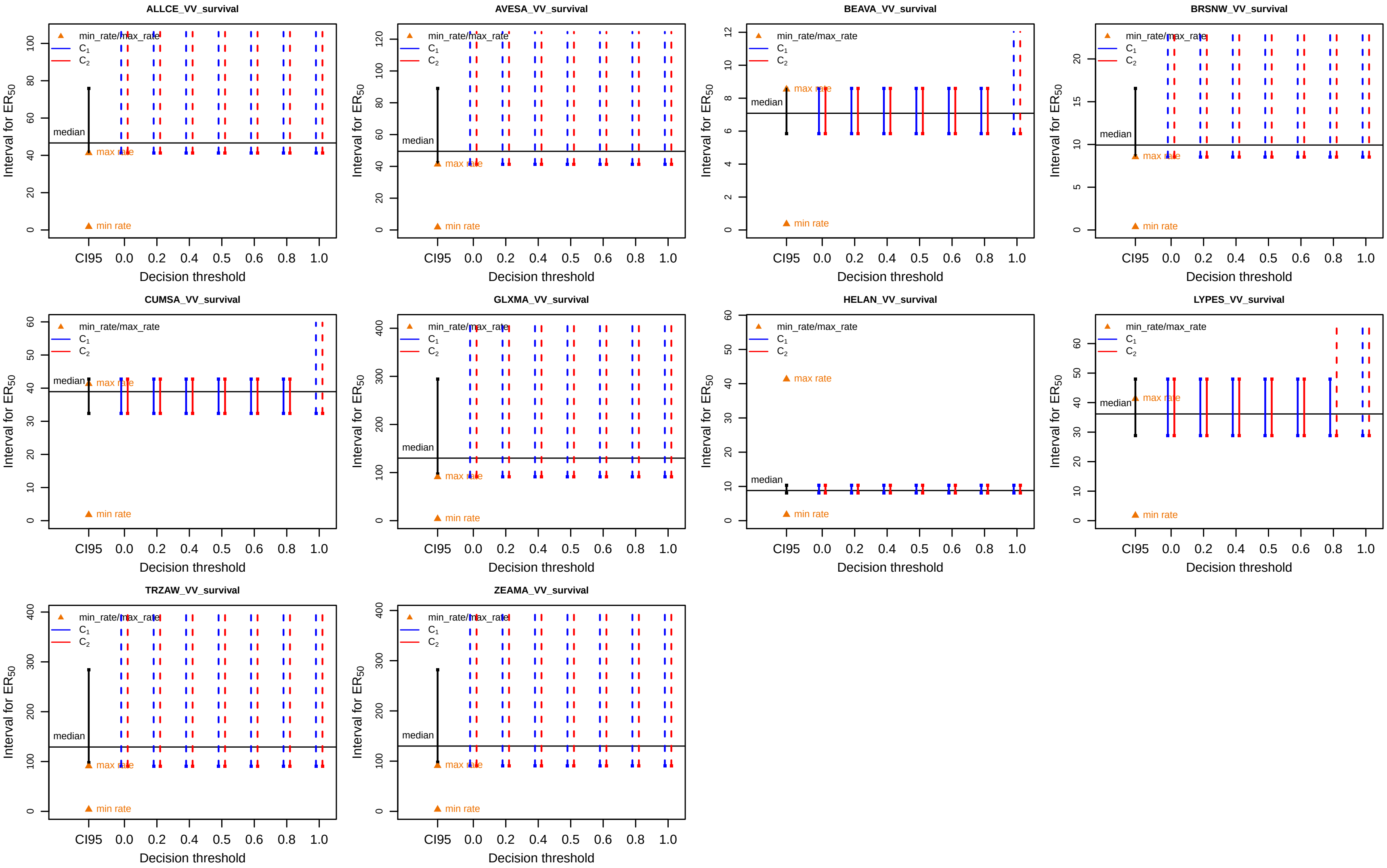

Figure 4: CI95 of ER_{50} and censored ER_{50} according to both criteria and the seven decision thresholds for all species - survival endpoint of the VV test of study 7. The two orange triangles stand for the lowest and the highest tested rates. Vertical segments of three different colors (black, blue, red) represent the CI95 of ER_{50}, the censored ER_{50} according to C_1 and the censored ER_{50} according to C_2, respectively; solid vertical segments are for bounded intervals while dotted vertical segments stand for right-unbounded intervals; the black horizontal line represents the median of the ER_{50}.

VV test - shoot dry weight endpoint

Table 16: Minimum and maximum tested rates, median, quantile 2.5% and quantile 97.5% of the ER_{50} posterior distribution - shoot dry weight endpoint of the VV test of study 7

species	min_rate	max_rate	ER_{50}_median	ER_{50}_LCI95	ER_{50}_UCI95
ALLCE	1.760	41.32	37.965213	27.928862	50.457253
AVESA	1.760	41.32	29.872184	23.640898	38.034025
BEAVA	0.364	8.54	1.756003	1.406141	2.215841
BRSNW	0.364	8.54	4.065580	3.600104	4.593755
CUMSA	1.760	41.32	9.378367	6.943601	12.905670
GLXMA	3.880	90.91	8.119111	6.372771	10.193543
HELAN	1.760	8.54	5.172458	4.318611	6.279537
LYPES	1.760	41.32	4.988694	4.388423	5.633699
TRZAW	3.880	90.91	123.306567	94.552554	290.279282
ZEAMA	3.880	90.91	94.766356	83.961649	112.980031

Table 17: Censored ER_{50} according to criterion 1 and the seven decision thresholds - shoot dry weight endpoint of the VV test of study 7

species	C1T0.0	C1T0.2	C1T0.4	C1T0.5	C1T0.6	C1T0.8	C1T1.0
ALLCE	[27.93; 50.46]	[27.93; 50.46]	[27.93; 50.46]	[27.93; 50.46]	[27.93; 50.46]	[27.93; Inf)	[27.93; Inf)
AVESA	[23.64; 38.03]	[23.64; 38.03]	[23.64; 38.03]	[23.64; 38.03]	[23.64; 38.03]	[23.64; 38.03]	[23.64; 38.03]
BEAVA	[1.41; 2.22]	[1.41; 2.22]	[1.41; 2.22]	[1.41; 2.22]	[1.41; 2.22]	[1.41; 2.22]	[1.41; 2.22]
BRSNW	[3.6; 4.59]	[3.6; 4.59]	[3.6; 4.59]	[3.6; 4.59]	[3.6; 4.59]	[3.6; 4.59]	[3.6; 4.59]
CUMSA	[6.94; 12.91]	[6.94; 12.91]	[6.94; 12.91]	[6.94; 12.91]	[6.94; 12.91]	[6.94; 12.91]	[6.94; 12.91]
GLXMA	[6.37; 10.19]	[6.37; 10.19]	[6.37; 10.19]	[6.37; 10.19]	[6.37; 10.19]	[6.37; 10.19]	[6.37; 10.19]
HELAN	[4.32; 6.28]	[4.32; 6.28]	[4.32; 6.28]	[4.32; 6.28]	[4.32; 6.28]	[4.32; 6.28]	[4.32; 6.28]
LYPES	[4.39; 5.63]	[4.39; 5.63]	[4.39; 5.63]	[4.39; 5.63]	[4.39; 5.63]	[4.39; 5.63]	[4.39; 5.63]
TRZAW	[90.91; Inf)	[90.91; Inf)	[90.91; Inf)	[90.91; Inf)	[90.91; Inf)	[90.91; Inf)	[90.91; Inf)
ZEAMA	[83.96; 112.98]	[83.96; 112.98]	[83.96; Inf)	[83.96; Inf)	[83.96; Inf)	[83.96; Inf)	[83.96; Inf)

Table 18: Censored ER_{50} according to criterion 2 and the seven decision thresholds - shoot dry weight endpoint of the VV test of study 7

species	C2T0.0	C2T0.2	C2T0.4	C2T0.5	C2T0.6	C2T0.8	C2T1.0
ALLCE	[27.93; 50.46]	[27.93; 50.46]	[27.93; 50.46]	[27.93; 50.46]	[27.93; Inf)	[27.93; Inf)	[27.93; Inf)
AVESA	[23.64; 38.03]	[23.64; 38.03]	[23.64; 38.03]	[23.64; 38.03]	[23.64; 38.03]	[23.64; 38.03]	[23.64; 38.03]
BEAVA	[1.41; 2.22]	[1.41; 2.22]	[1.41; 2.22]	[1.41; 2.22]	[1.41; 2.22]	[1.41; 2.22]	[1.41; 2.22]
BRSNW	[3.6; 4.59]	[3.6; 4.59]	[3.6; 4.59]	[3.6; 4.59]	[3.6; 4.59]	[3.6; 4.59]	[3.6; 4.59]
CUMSA	[6.94; 12.91]	[6.94; 12.91]	[6.94; 12.91]	[6.94; 12.91]	[6.94; 12.91]	[6.94; 12.91]	[6.94; 12.91]
GLXMA	[6.37; 10.19]	[6.37; 10.19]	[6.37; 10.19]	[6.37; 10.19]	[6.37; 10.19]	[6.37; 10.19]	[6.37; 10.19]
HELAN	[4.32; 6.28]	[4.32; 6.28]	[4.32; 6.28]	[4.32; 6.28]	[4.32; 6.28]	[4.32; 6.28]	[4.32; 6.28]
LYPES	[4.39; 5.63]	[4.39; 5.63]	[4.39; 5.63]	[4.39; 5.63]	[4.39; 5.63]	[4.39; 5.63]	[4.39; 5.63]
TRZAW	[90.91; Inf)	[90.91; Inf)	[90.91; Inf)	[90.91; Inf)	[90.91; Inf)	[90.91; Inf)	[90.91; Inf)
ZEAMA	[83.96; 112.98]	[83.96; 112.98]	[83.96; Inf)	[83.96; Inf)	[83.96; Inf)	[83.96; Inf)	[83.96; Inf)

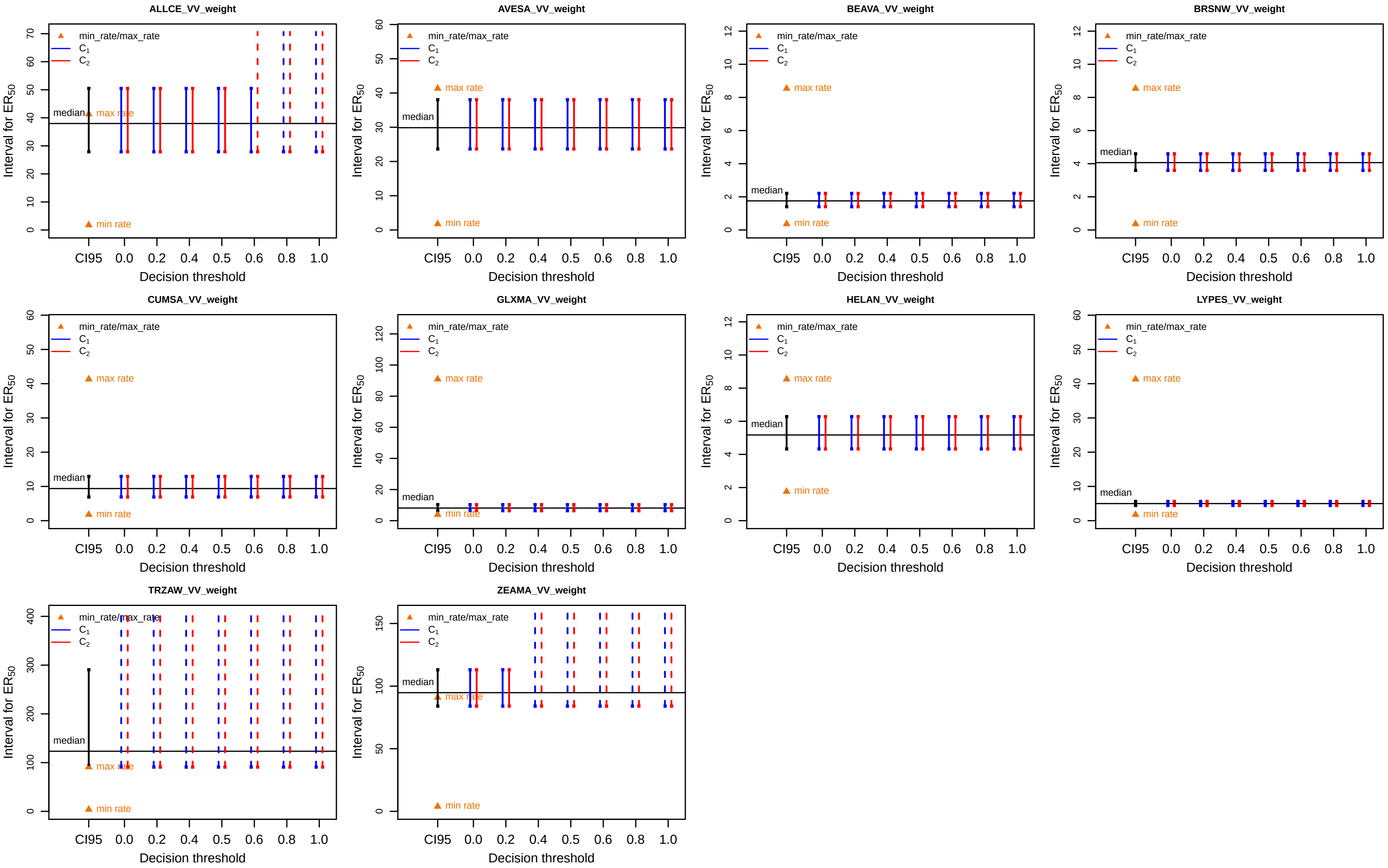

Figure 5: CI95 of ER_{50} and censored ER_{50} according to both criteria and the seven decision thresholds for all species - shoot dry weight endpoint of the VV test of study 7. The two orange triangles stand for the lowest and the highest tested rates. Vertical segments of three different colors (black, blue, red) represent the CI95 of ER_{50}, the censored ER_{50} according to C_1 and the censored ER_{50} according to C_2, respectively; solid vertical segments are for bounded intervals while dotted vertical segments stand for right-unbounded intervals; the black horizontal line represents the median of the ER_{50}.
