# Supplementary material for: How to account for the uncertainty from standard toxicity tests in species sensitivity distributions: An example in non-target plants
Source: PLoS One. 2021 Jan 7;16(1):e0245071. doi: 10.1371/journal.pone.0245071 (PMC7790375; doi:10.1371/journal.pone.0245071)
Supplement: S1 Archive — It is a zip file containing seven folders (one folder per case study). Each folder contains five files report_xxx.pdf with detailed results of the dose-response analyses, one file corresponding to does-response analysis per endpoint. It also contains one file ER50_censoring.pdf for censored ER50 and one file SSD_analyses.pdf for results of SSD analyses. (ZIP) [file pone.0245071.s004.zip › S1_archive/Study7/report_SE_emergence.pdf]

Dose-response analyses

Study 7

Seedling Emergence test - emergence endpoint

25 June 2020

Contact: sandrine.charles@univ-lyon1.fr

This is a report which provides results on all performed dose-response analyses for the emergence endpoint of the Seedling Emergence test for study 7.

Contents

Data set: ALLCE_SE_emergence . . . 2
Data set: AVESA_SE_emergence . . . 3
Data set: BEAVA_SE_emergence . . . 4
Data set: BRSNW_SE_emergence . . . 5
Data set: CUMSA_SE_emergence . . . 6
Data set: GLXMA_SE_emergence . . . 7
Data set: HELAN_SE_emergence . . . 8
Data set: LYPES_SE_emergence . . . 9
Data set: TRZAW_SE_emergence . . . 10
Data set: ZEAMA_SE_emergence . . . 11

Data set: ALLCE_SE_emergence

Table 1: Summary of parameter estimates for ALLCE_SE_emergence data set

Parameter	median	Q2.5	Q97.5
b	0.856	0.425	2.530
d	0.794	0.714	0.870
e	97.060	45.114	334.493

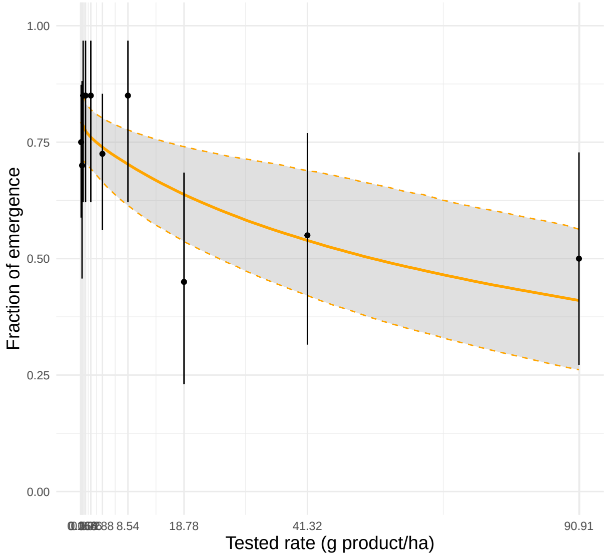

(a) Dose-response curve

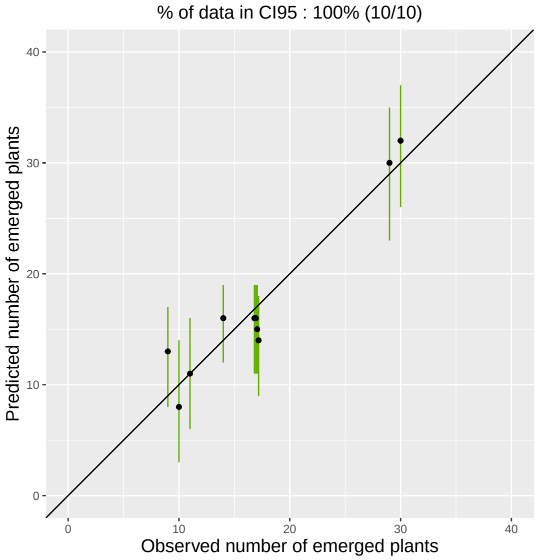

(b) Posterior predictive check (PPC)

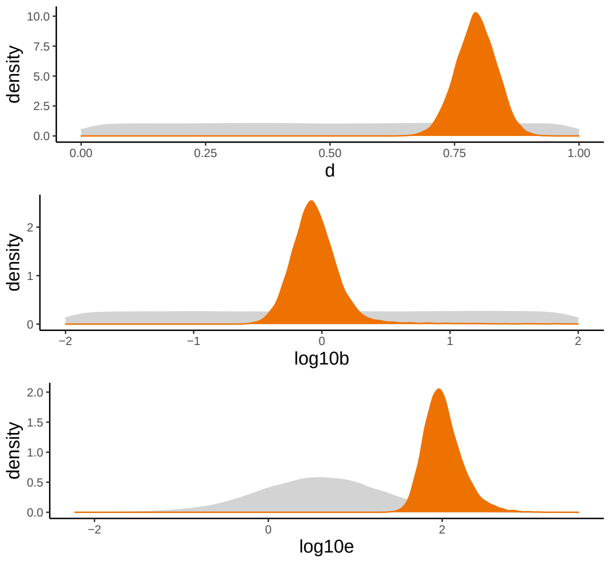

(c) Priors and posteriors

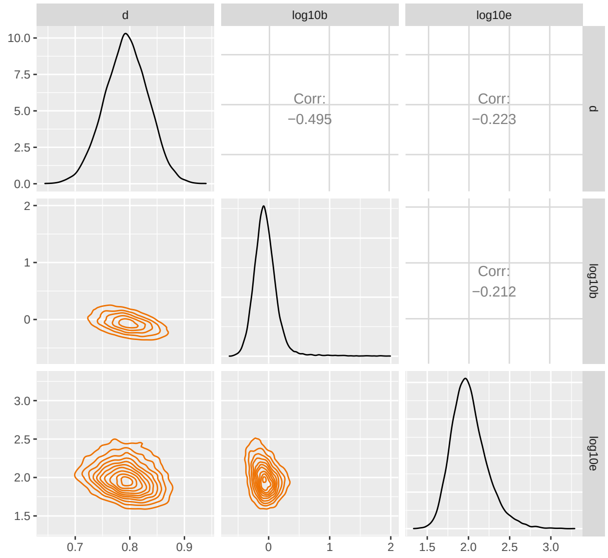

(d) Correlations between parameters

Figure 1: Dose-response curve (a), PPC (b), prior and posterior distributions (c) and correlations between parameters (d).

Data set: AVESA_SE_emergence

Table 2: Summary of parameter estimates for AVESA_SE_emergence data set

Parameter	median	Q2.5	Q97.5
b	22.370	3.199	93.202
d	0.950	0.899	0.981
e	122.905	94.926	261.252

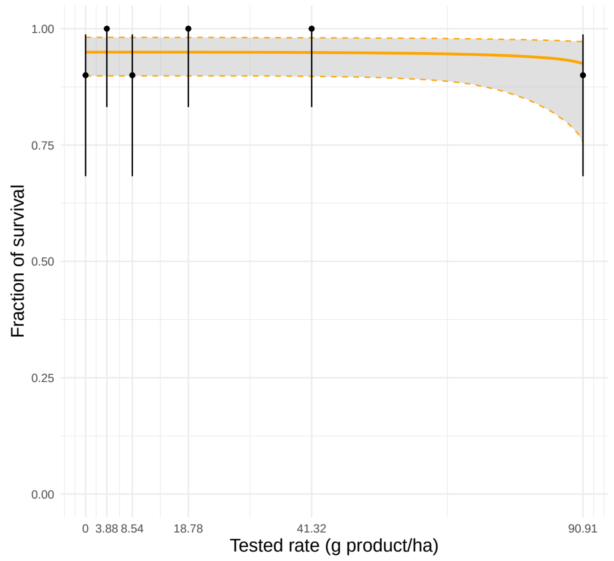

(a) Dose-response curve

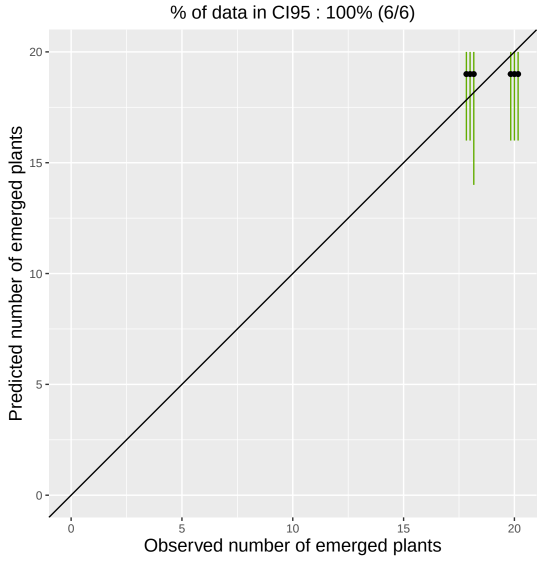

(b) Posterior predictive check (PPC)

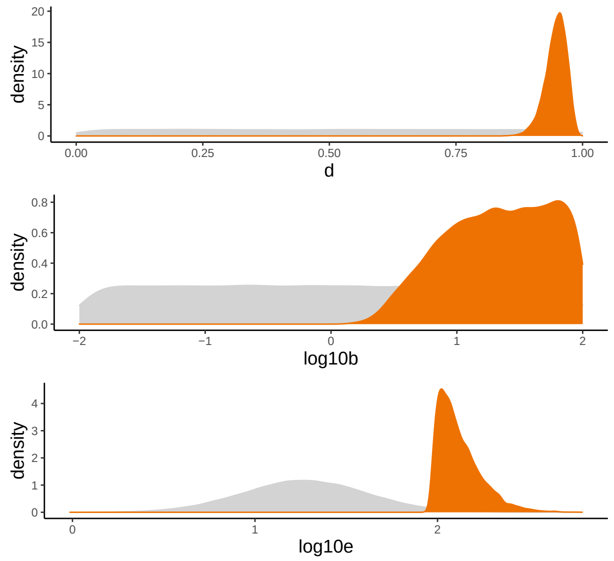

(c) Priors and posteriors

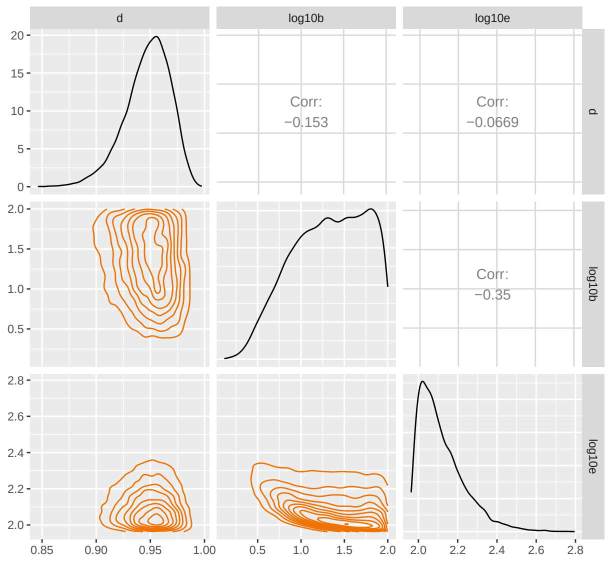

(d) Correlations between parameters

Figure 2: Dose-response curve (a), PPC (b), prior and posterior distributions (c) and correlations between parameters (d).

Data set: BEAVA_SE_emergence

Table 3: Summary of parameter estimates (parameter d is set to 1) for BEAVA_SE_emergence data set

Parameter	median	Q2.5	Q97.5
b	1.016	0.668	1.552
e	445.002	186.973	1494.536

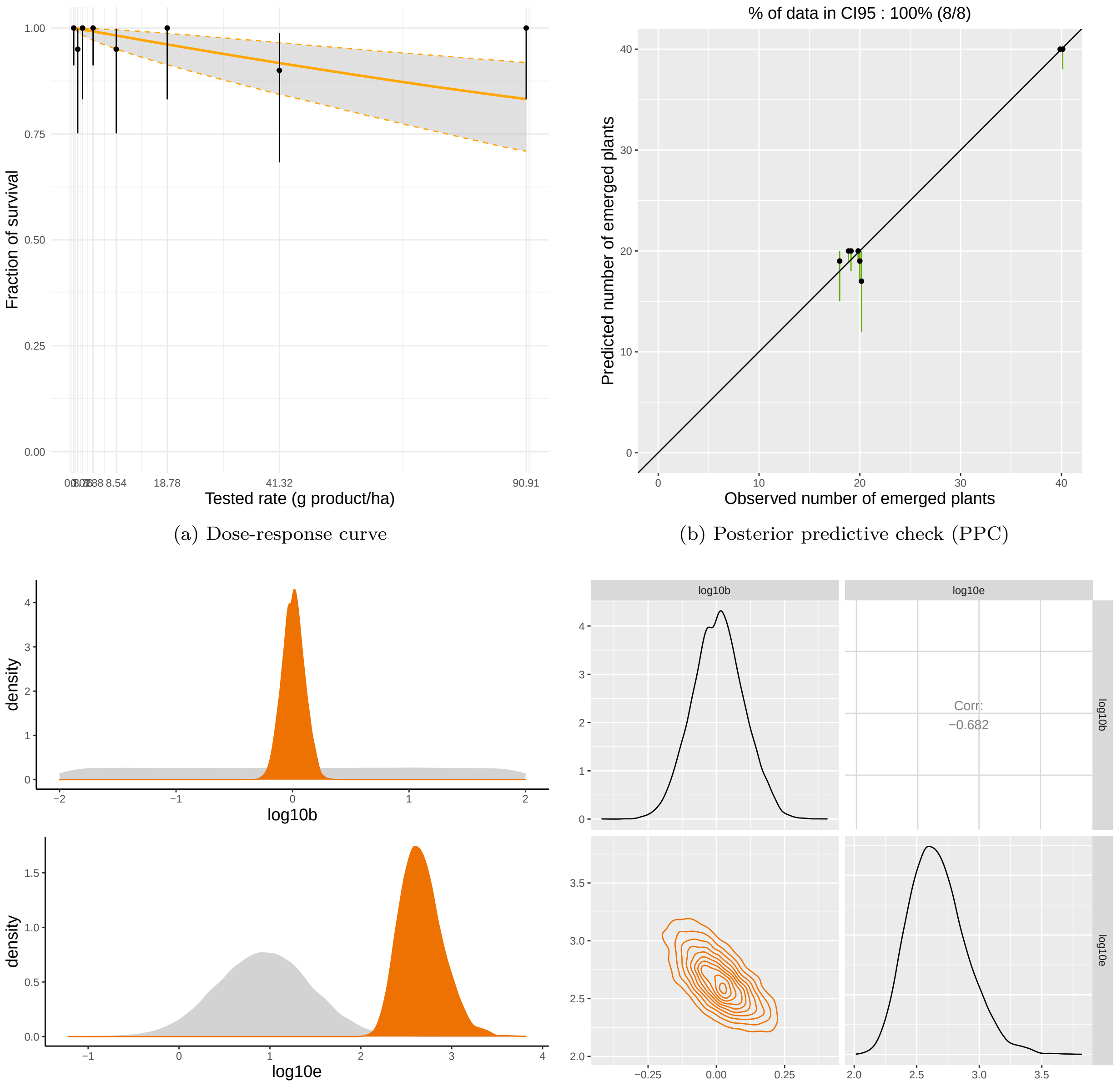

(a) Dose-response curve

(b) Posterior predictive check (PPC)

(c) Priors and posteriors

(d) Correlations between parameters

Figure 3: Dose-response curve (a), PPC (b), prior and posterior distributions (c) and correlations between parameters (d).

Data set: BRSNW_SE_emergence

Table 4: Summary of parameter estimates (parameter d is set to 1) for BRSNW_SE_emergence data set

Parameter	median	Q2.5	Q97.5
b	1.324	0.793	2.046
e	95.133	62.532	181.623

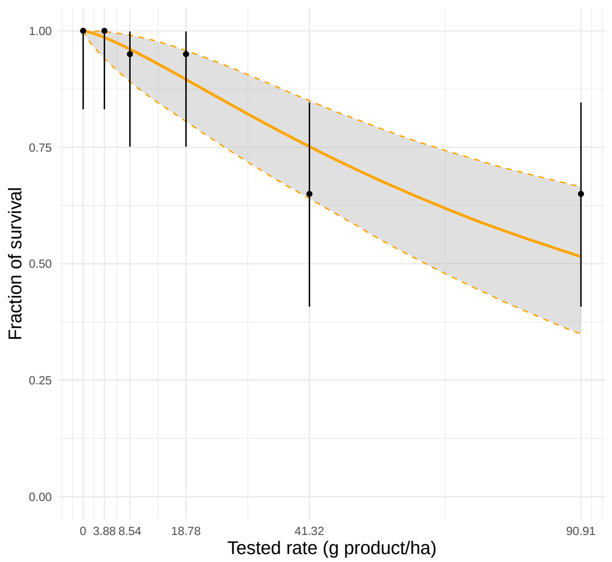

(a) Dose-response curve

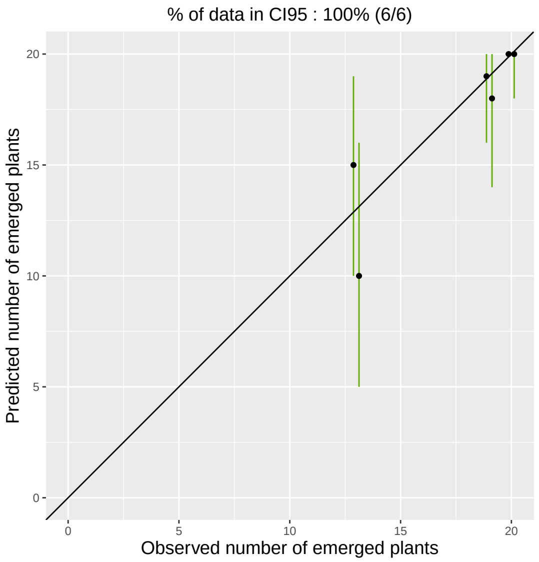

(b) Posterior predictive check (PPC)

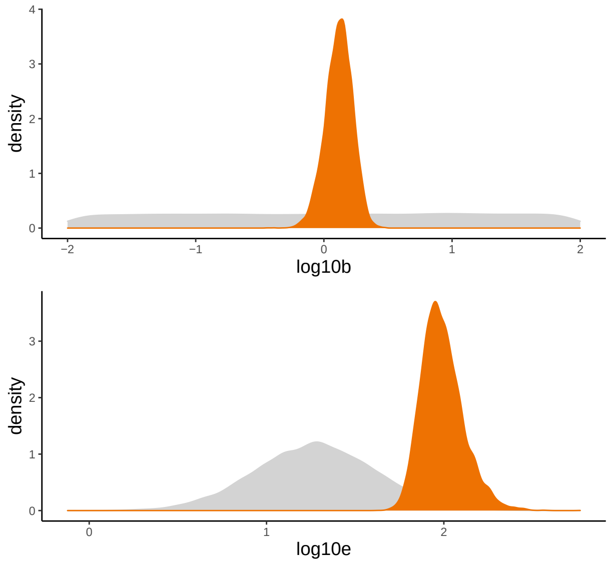

(c) Priors and posteriors

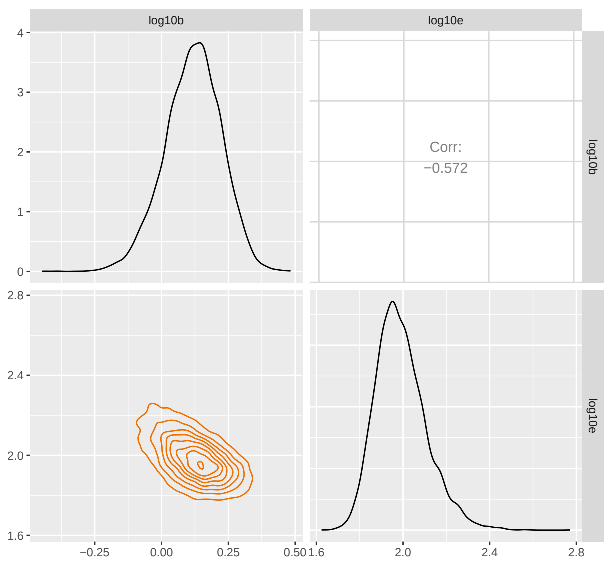

(d) Correlations between parameters

Figure 4: Dose-response curve (a), PPC (b), prior and posterior distributions (c) and correlations between parameters (d).

Data set: CUMSA_SE_emergence

Table 5: Summary of parameter estimates for CUMSA_SE_emergence data set

Parameter	median	Q2.5	Q97.5
b	26.439	3.214	93.735
d	0.923	0.865	0.963
e	126.036	95.624	275.409

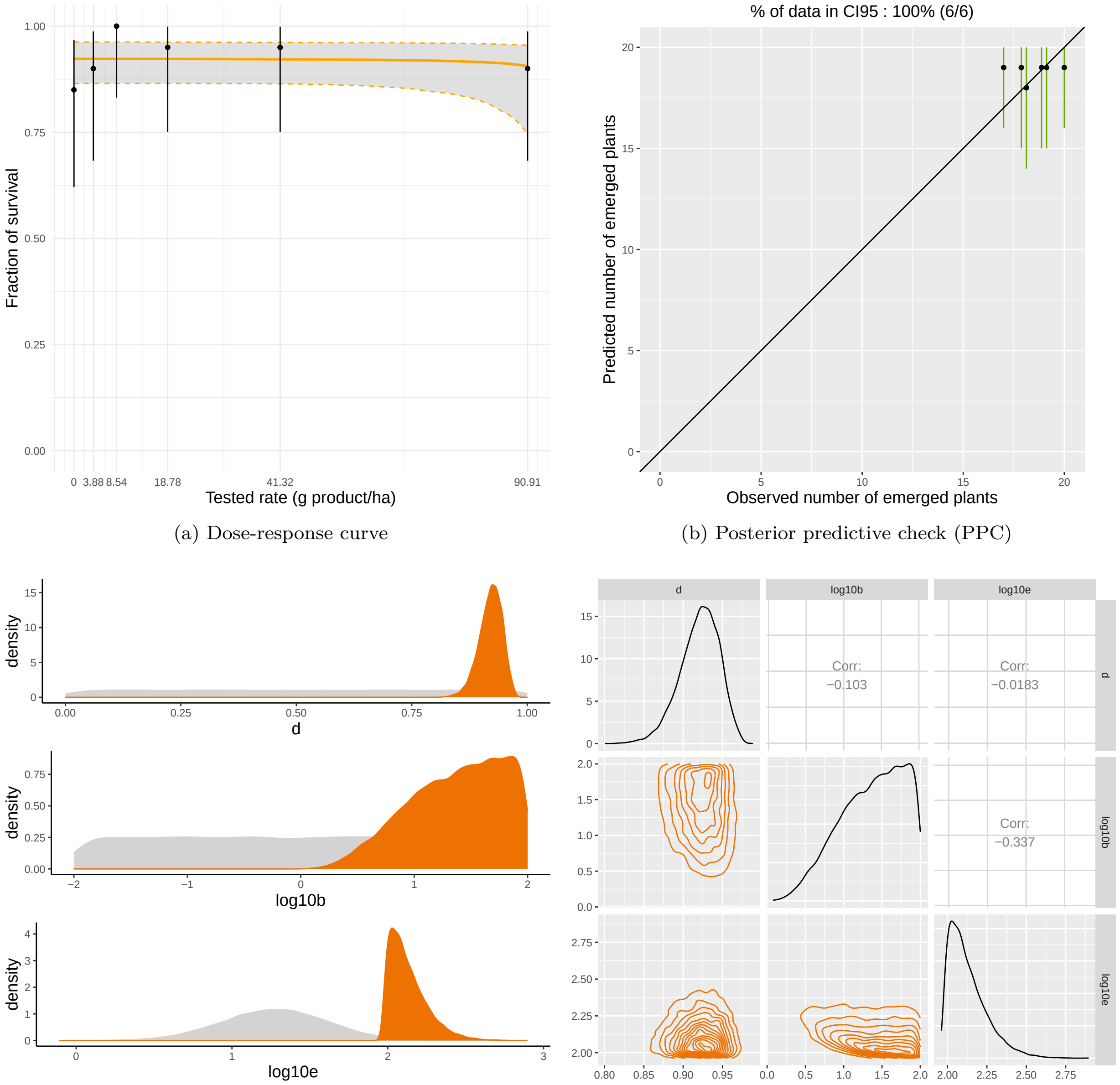

(a) Dose-response curve

(b) Posterior predictive check (PPC)

(c) Priors and posteriors

(d) Correlations between parameters

Figure 5: Dose-response curve (a), PPC (b), prior and posterior distributions (c) and correlations between parameters (d).

Data set: GLXMA_SE_emergence

Table 6: Summary of parameter estimates (parameter d is set to 1) for GLXMA_SE_emergence data set

Parameter	median	Q2.5	Q97.5
b	1.370	0.863	2.175
e	265.741	145.006	634.716

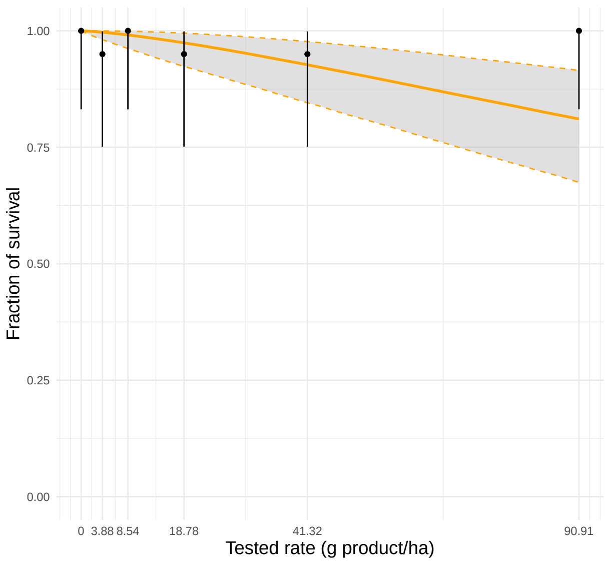

(a) Dose-response curve

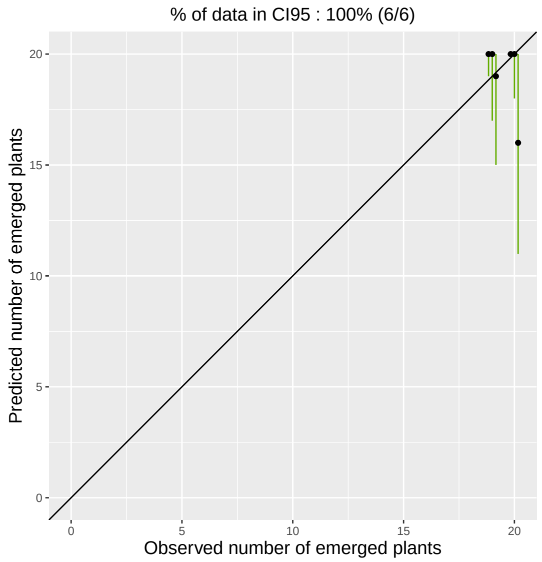

(b) Posterior predictive check (PPC)

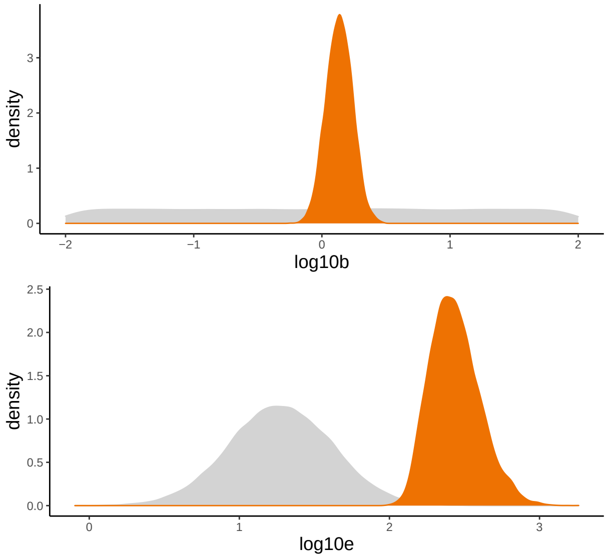

(c) Priors and posteriors

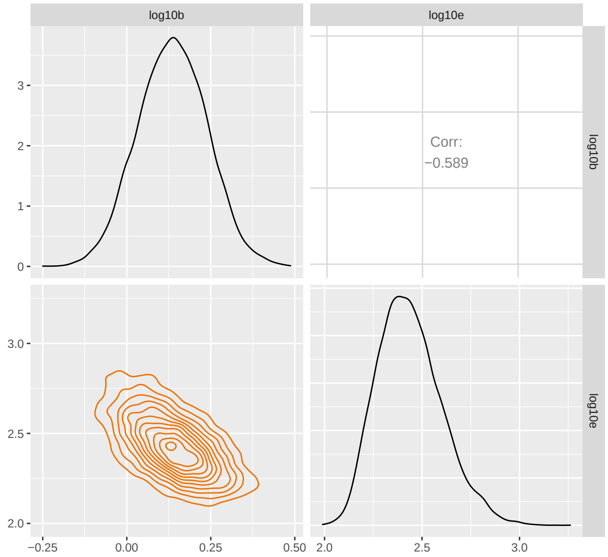

(d) Correlations between parameters

Figure 6: Dose-response curve (a), PPC (b), prior and posterior distributions (c) and correlations between parameters (d).

Data set: HELAN_SE_emergence

Table 7: Summary of parameter estimates (parameter d is set to 1) for HELAN_SE_emergence data set

Parameter	median	Q2.5	Q97.5
b	1.215	0.773	1.896
e	264.951	140.472	626.001

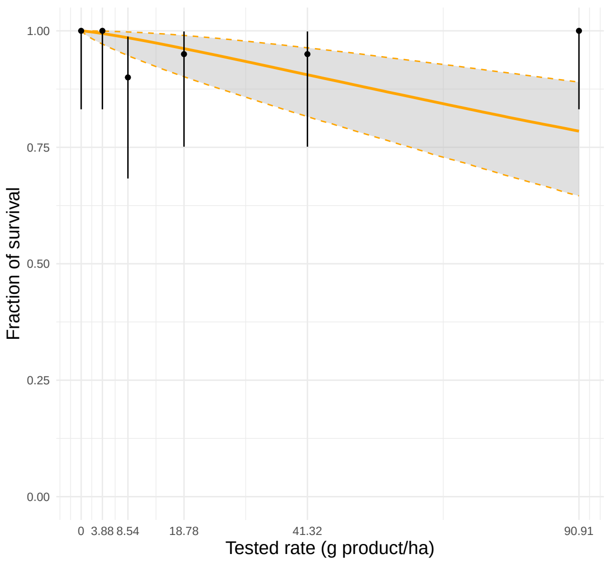

(a) Dose-response curve

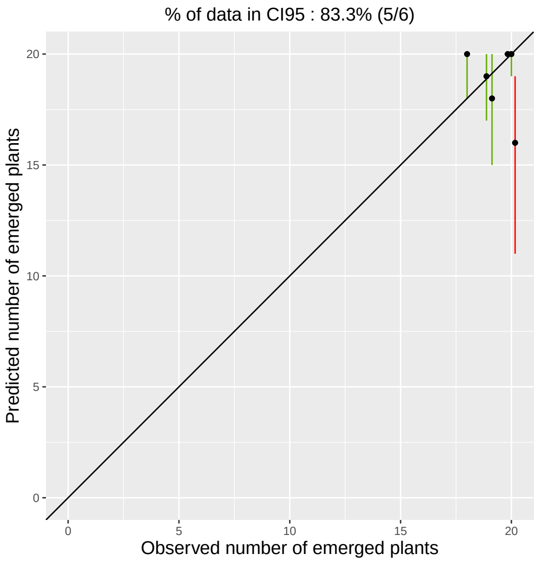

(b) Posterior predictive check (PPC)

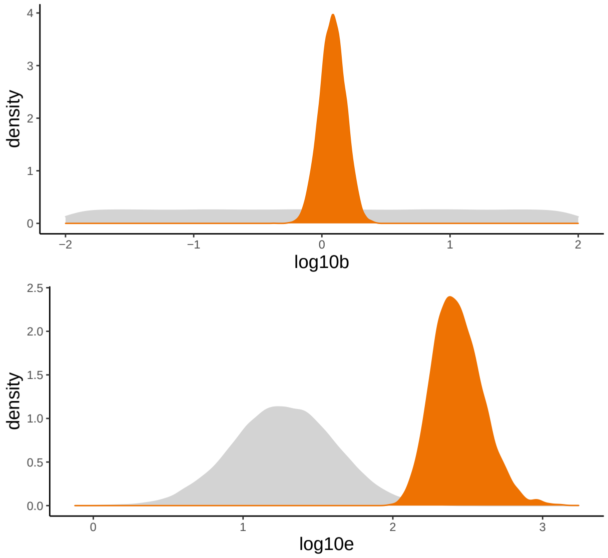

(c) Priors and posteriors

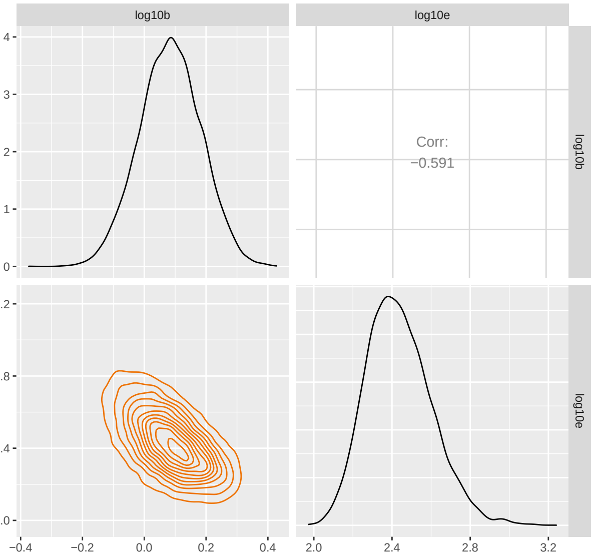

(d) Correlations between parameters

Figure 7: Dose-response curve (a), PPC (b), prior and posterior distributions (c) and correlations between parameters (d).

Data set: LYPES_SE_emergence

Table 8: Summary of parameter estimates for LYPES_SE_emergence data set

Parameter	median	Q2.5	Q97.5
b	29.699	3.190	94.289
d	0.946	0.896	0.978
e	128.145	96.391	291.520

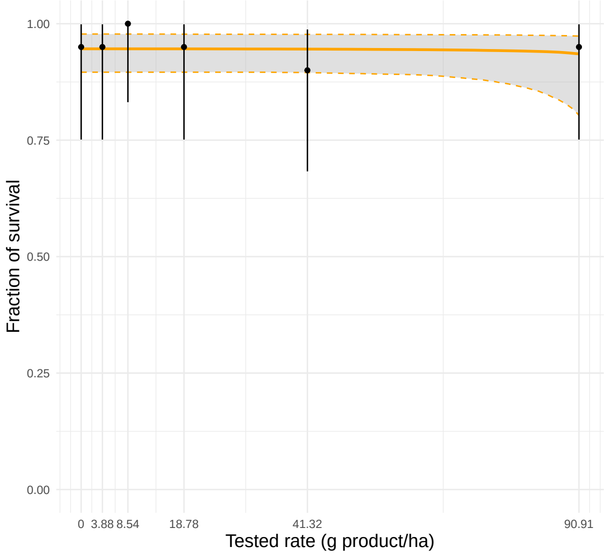

(a) Dose-response curve

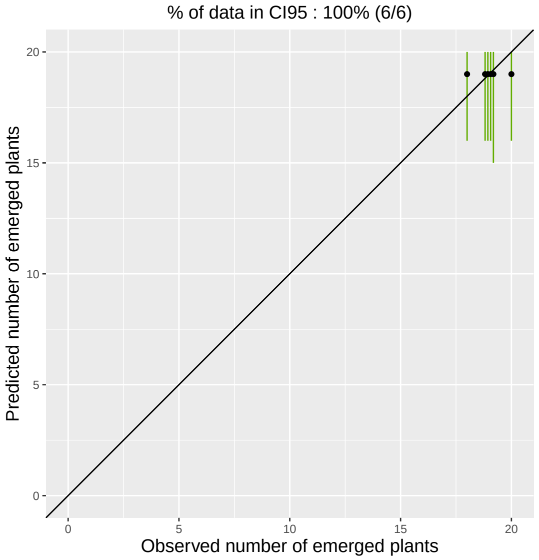

(b) Posterior predictive check (PPC)

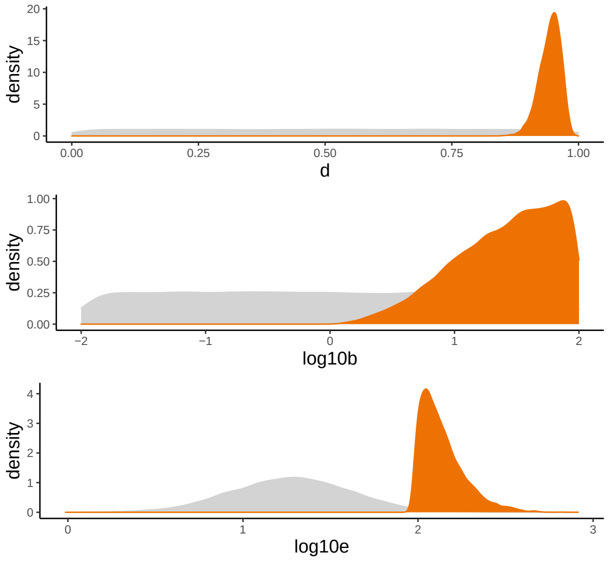

(c) Priors and posteriors

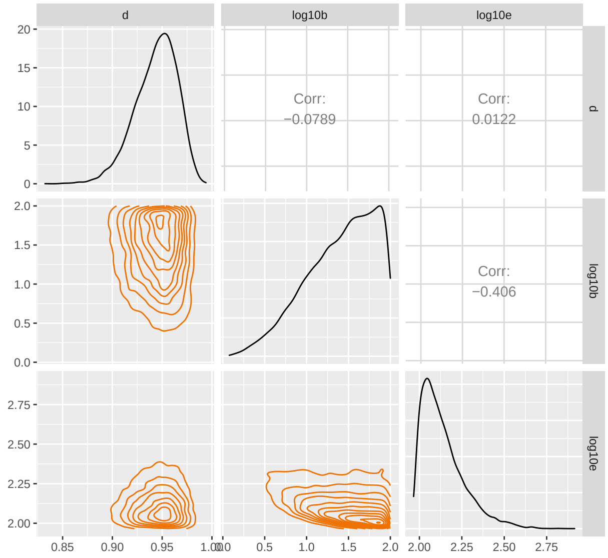

(d) Correlations between parameters

Figure 8: Dose-response curve (a), PPC (b), prior and posterior distributions (c) and correlations between parameters (d).

Data set: TRZAW_SE_emergence

Table 9: Summary of parameter estimates (parameter d is set to 1) for TRZAW_SE_emergence data set

Parameter	median	Q2.5	Q97.5
b	3.009	1.510	6.399
e	169.382	113.753	358.250

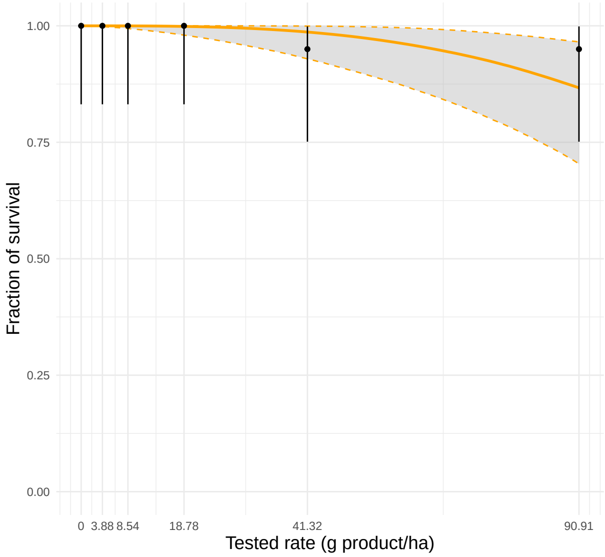

(a) Dose-response curve

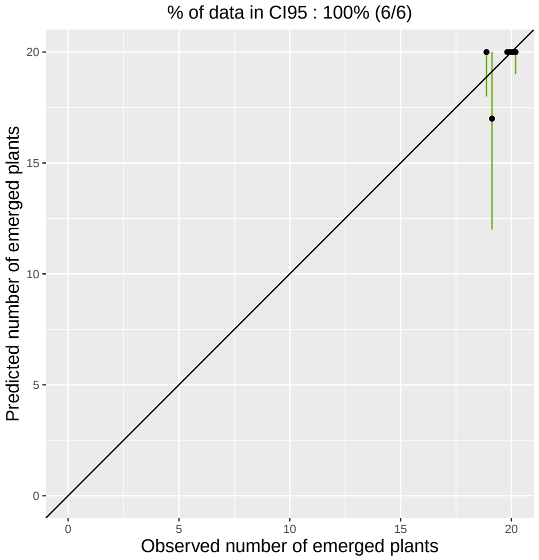

(b) Posterior predictive check (PPC)

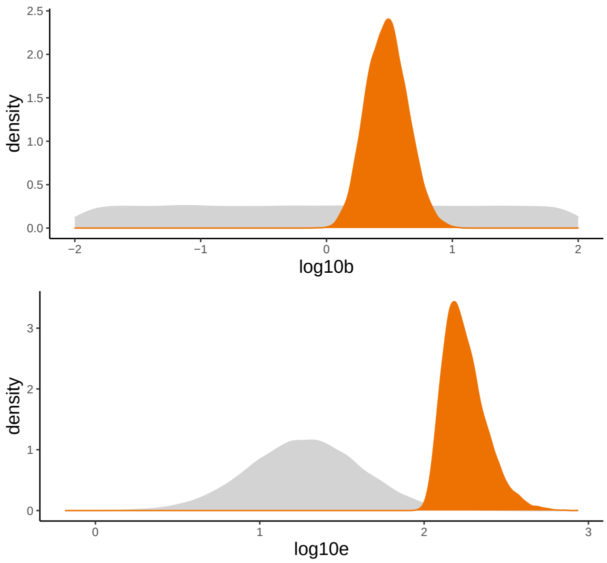

(c) Priors and posteriors

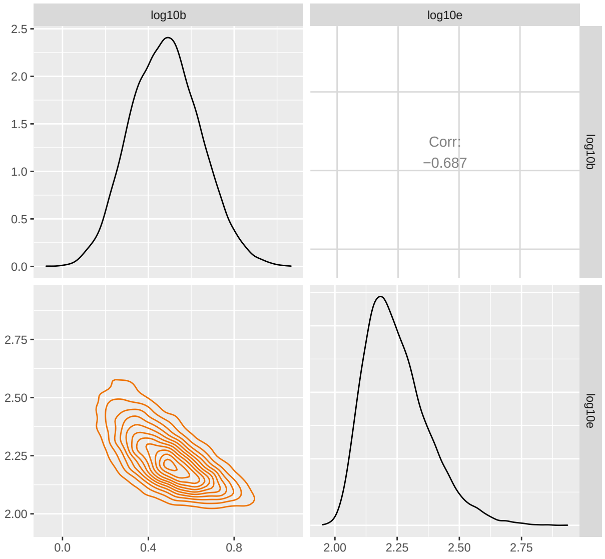

(d) Correlations between parameters

Figure 9: Dose-response curve (a), PPC (b), prior and posterior distributions (c) and correlations between parameters (d).

Data set: ZEAMA_SE_emergence

Table 10: Summary of parameter estimates for ZEAMA_SE_emergence data set

Parameter	median	Q2.5	Q97.5
b	34.013	4.981	95.444
d	0.970	0.929	0.991
e	131.390	97.146	294.996

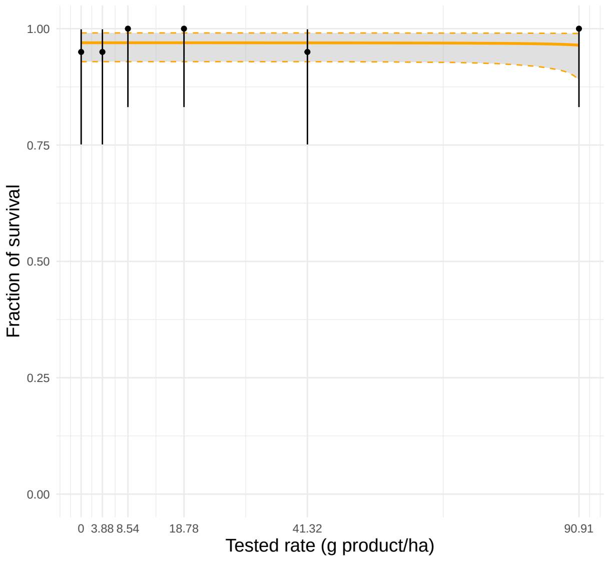

(a) Dose-response curve

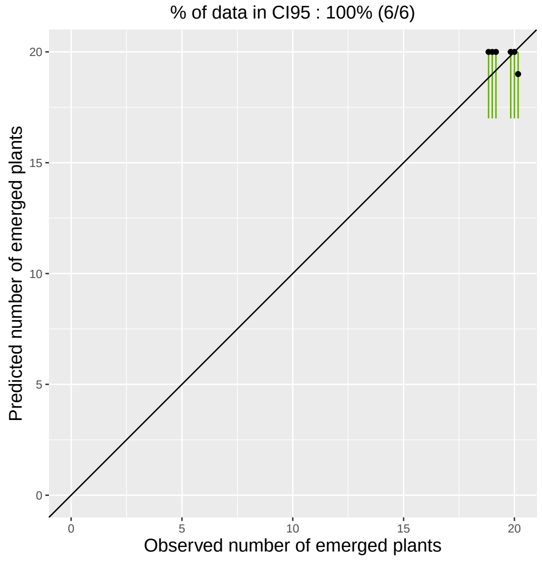

(b) Posterior predictive check (PPC)

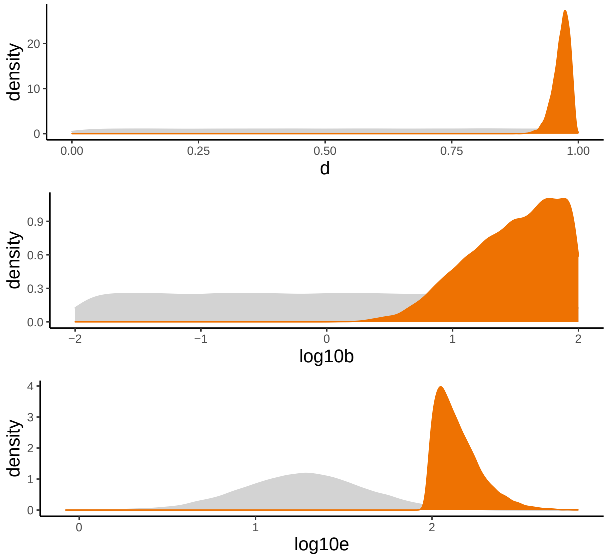

(c) Priors and posteriors

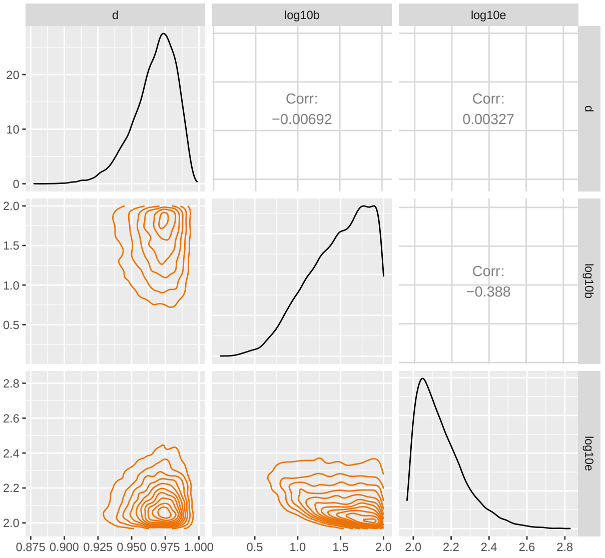

(d) Correlations between parameters

Figure 10: Dose-response curve (a), PPC (b), prior and posterior distributions (c) and correlations between parameters (d).
