# Supplementary material for: How to account for the uncertainty from standard toxicity tests in species sensitivity distributions: An example in non-target plants
Source: PLoS One. 2021 Jan 7;16(1):e0245071. doi: 10.1371/journal.pone.0245071 (PMC7790375; doi:10.1371/journal.pone.0245071)
Supplement: S1 Archive — It is a zip file containing seven folders (one folder per case study). Each folder contains five files report_xxx.pdf with detailed results of the dose-response analyses, one file corresponding to does-response analysis per endpoint. It also contains one file ER50_censoring.pdf for censored ER50 and one file SSD_analyses.pdf for results of SSD analyses. (ZIP) [file pone.0245071.s004.zip › S1_archive/Study7/report_SE_weight.pdf]

Dose-response analysis

Study 7

Seedling Emergence test - shoot dry SE_weight endpoint

25 June 2020

Contact: sandrine.charles@univ-lyon1.fr

This is a report which provides results on all performed dose-response analyses for the shoot dry SE_weight endpoint of the Seedling Emergence test for study 7.

Contents

Data set: ALLCE_SE_weight . . . 2
Data set: AVESA_SE_weight . . . 3
Data set: BEAVA_SE_weight . . . 4
Data set: BRSNW_SE_weight . . . 5
Data set: CUMSA_SE_weight . . . 6
Data set: GLXMA_SE_weight . . . 7
Data set: HELAN_SE_weight . . . 8
Data set: LYPES_SE_weight . . . 9
Data set: TRZAW_SE_weight . . . 10
Data set: ZEAMA_SE_weight . . . 11

Data set: ALLCE_SE_weight

Table 1: Summary of parameter estimates for ALLCE_SE_weight data set

Parameter	median	Q2.5	Q97.5
b	0.446	0.100	0.849
d	0.027	0.022	0.031
e	0.362	0.100	1.435
sigma	0.007	0.005	0.009

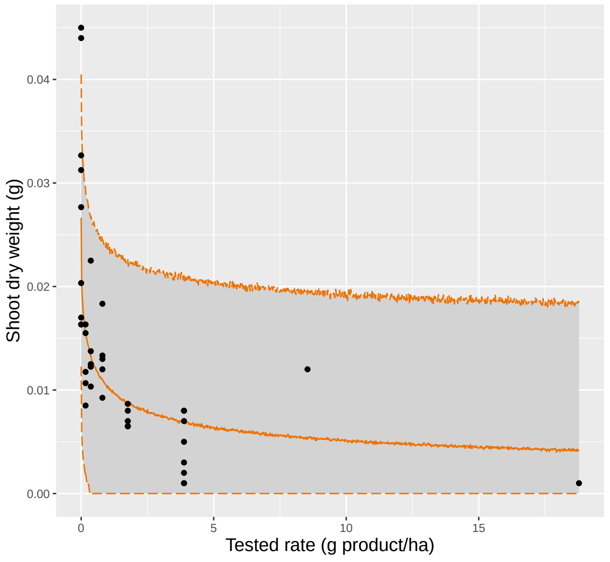

(a) Dose-response curve

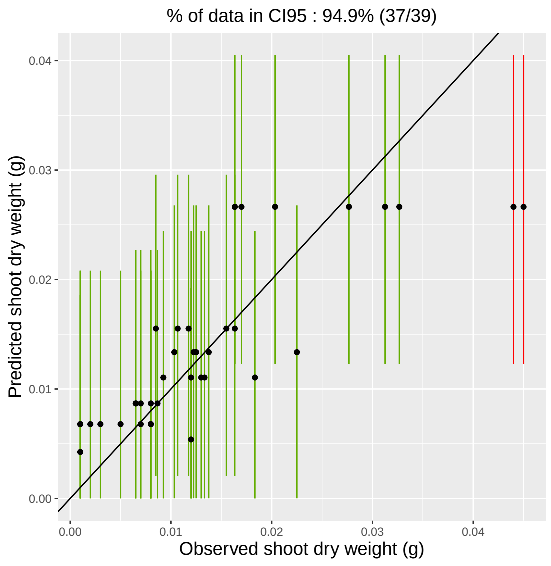

(b) Posterior predictive check (PPC)

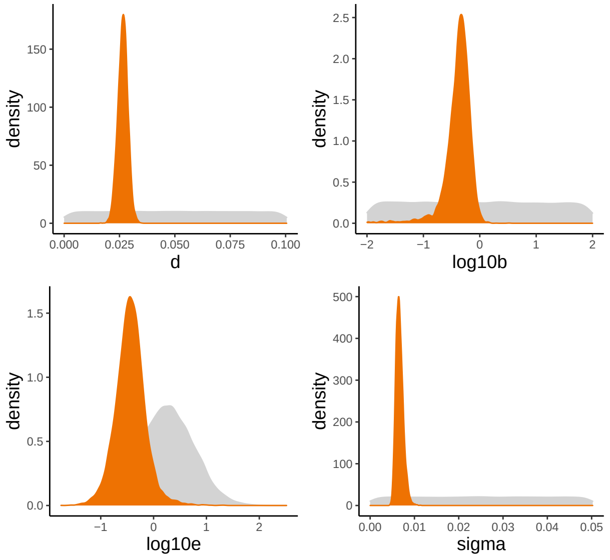

(c) Priors and posteriors

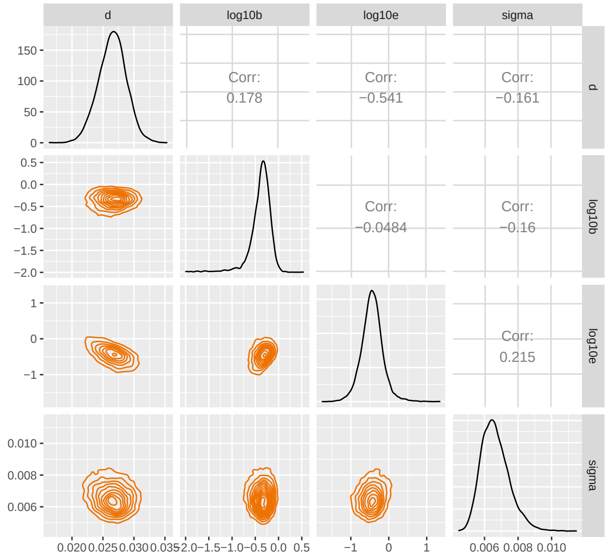

(d) Correlations between parameters

Figure 1: Dose-response curve (a), PPC (b), prior and posterior distributions (c) and correlations between parameters (d).

Data set: AVESA_SE_weight

Table 2: Summary of parameter estimates for AVESA_SE_weight data set

Parameter	median	Q2.5	Q97.5
b	2.026	1.525	2.845
d	0.491	0.453	0.534
e	13.604	11.405	16.098
sigma	0.046	0.036	0.063

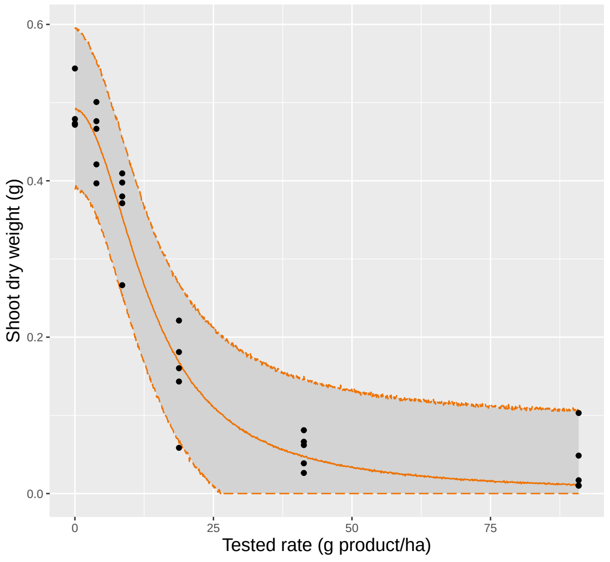

(a) Dose-response curve

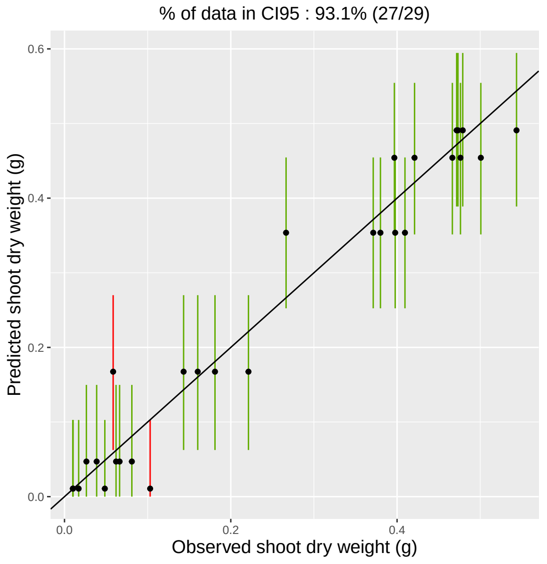

(b) Posterior predictive check (PPC)

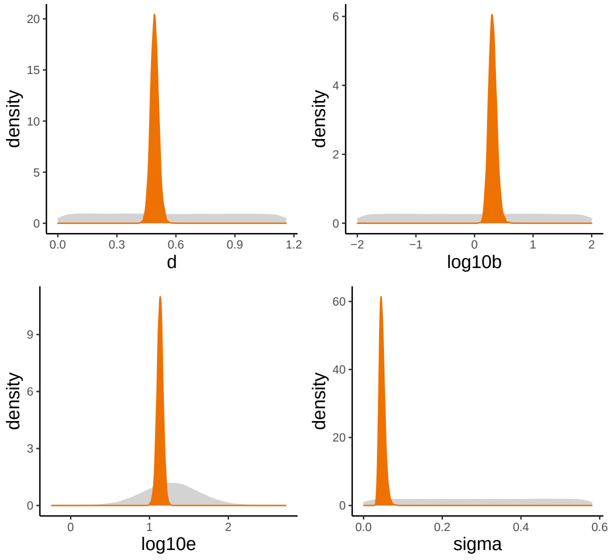

(c) Priors and posteriors

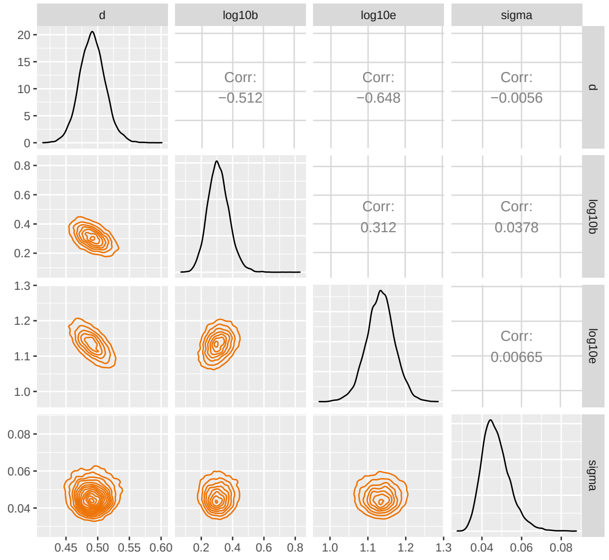

(d) Correlations between parameters

Figure 2: Dose-response curve (a), PPC (b), prior and posterior distributions (c) and correlations between parameters (d).

Data set: BEAVA_SE_weight

Table 3: Summary of parameter estimates for BEAVA_SE_weight data set

Parameter	median	Q2.5	Q97.5
b	1.013	0.589	1.767
d	0.419	0.372	0.467
e	3.976	2.591	5.887
sigma	0.100	0.085	0.119

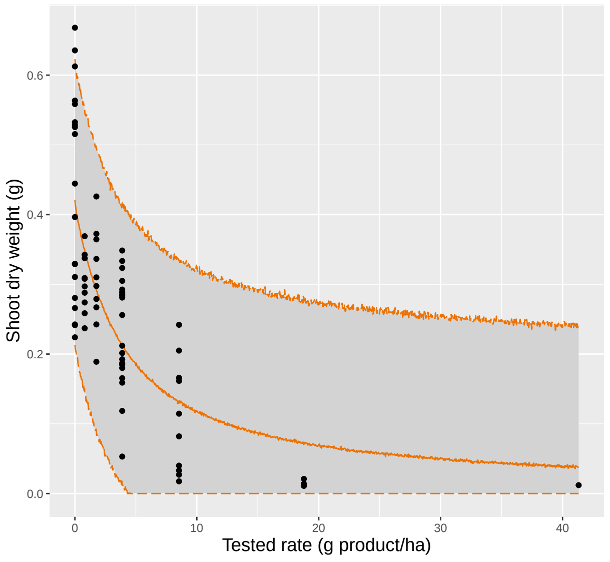

(a) Dose-response curve

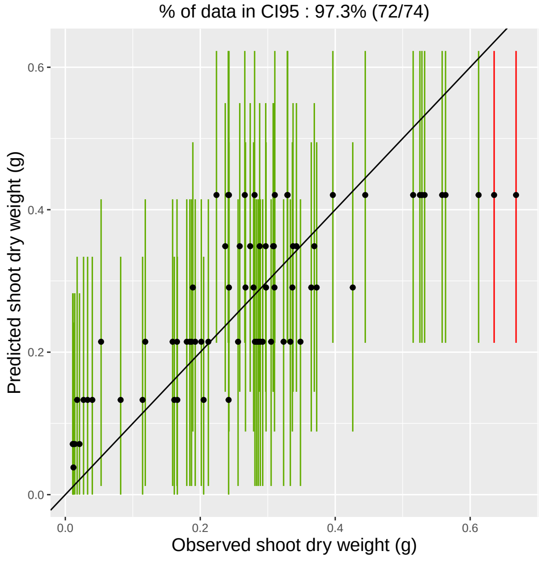

(b) Posterior predictive check (PPC)

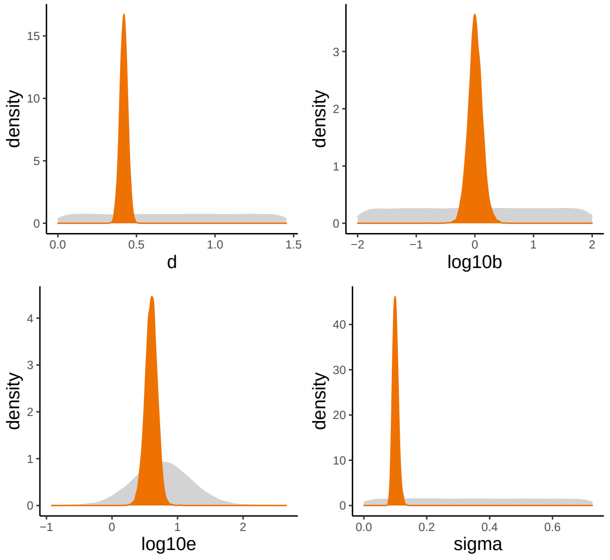

(c) Priors and posteriors

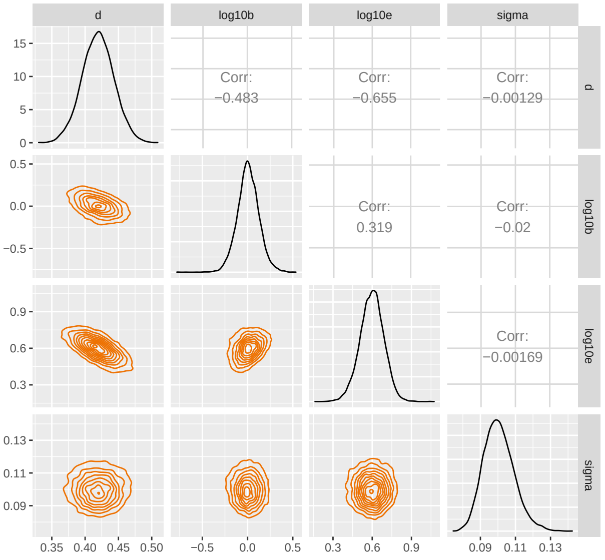

(d) Correlations between parameters

Figure 3: Dose-response curve (a), PPC (b), prior and posterior distributions (c) and correlations between parameters (d).

Data set: BRSNW_SE_weight

Table 4: Summary of parameter estimates for BRSNW_SE_weight data set

Parameter	median	Q2.5	Q97.5
b	3.293	2.110	8.143
d	0.974	0.878	1.077
e	11.237	9.272	13.748
sigma	0.186	0.153	0.234

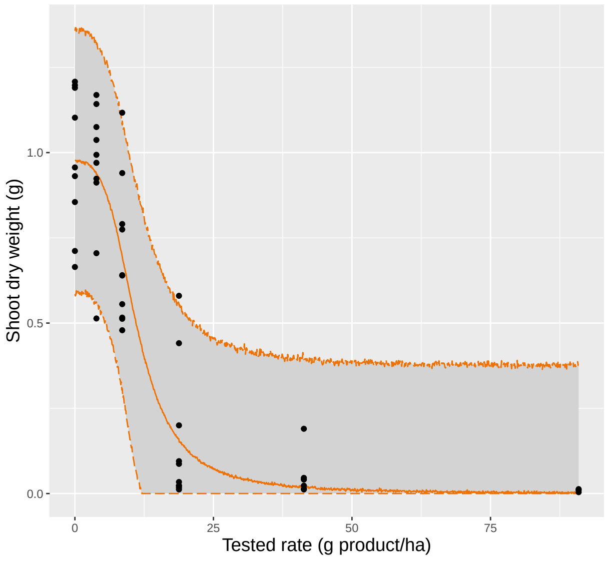

(a) Dose-response curve

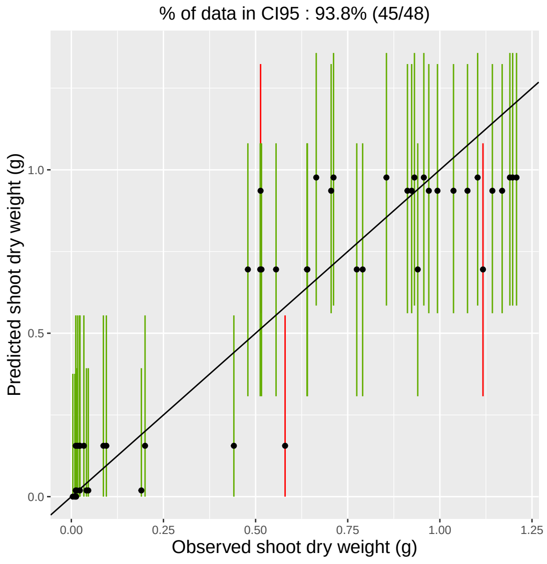

(b) Posterior predictive check (PPC)

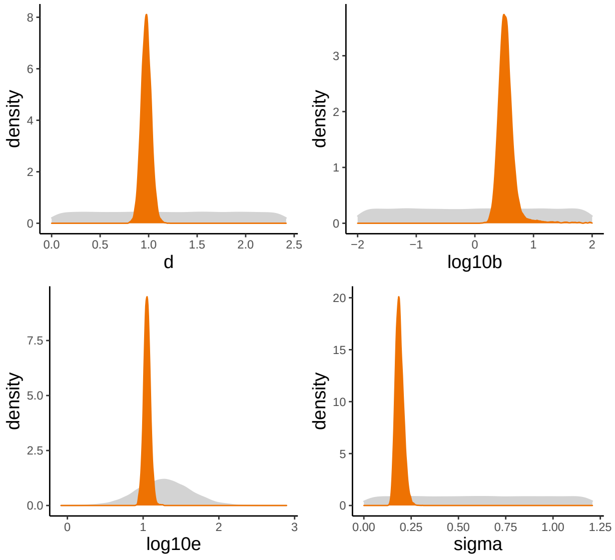

(c) Priors and posteriors

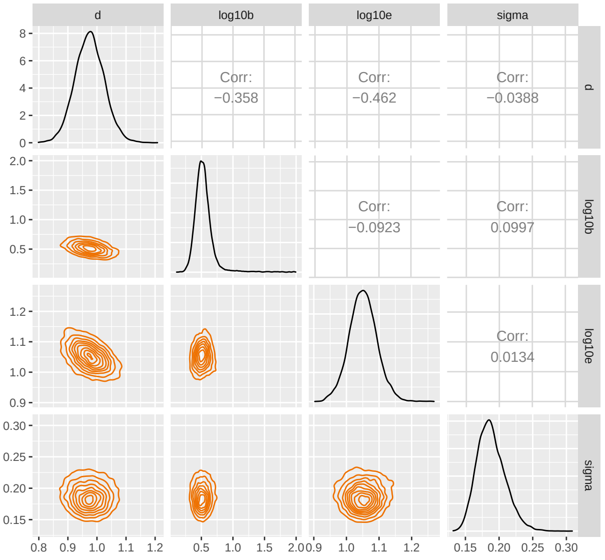

(d) Correlations between parameters

Figure 4: Dose-response curve (a), PPC (b), prior and posterior distributions (c) and correlations between parameters (d).

Data set: CUMSA_SE_weight

Table 5: Summary of parameter estimates for CUMSA_SE_weight data set

Parameter	median	Q2.5	Q97.5
b	1.244	0.932	1.674
d	1.489	1.332	1.647
e	9.283	7.002	12.337
sigma	0.245	0.205	0.300

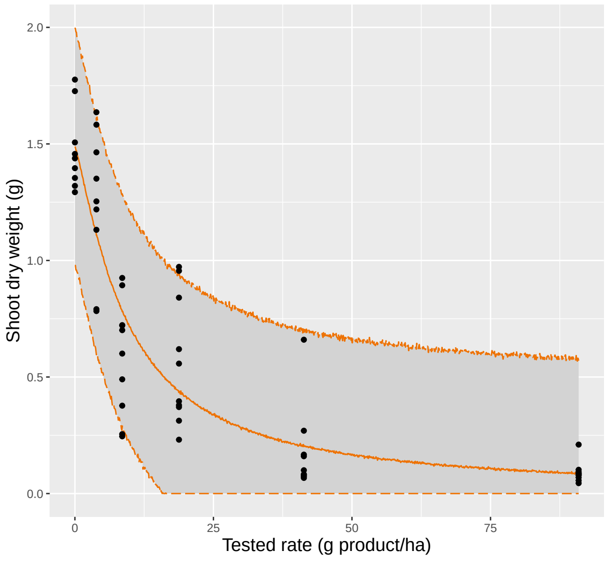

(a) Dose-response curve

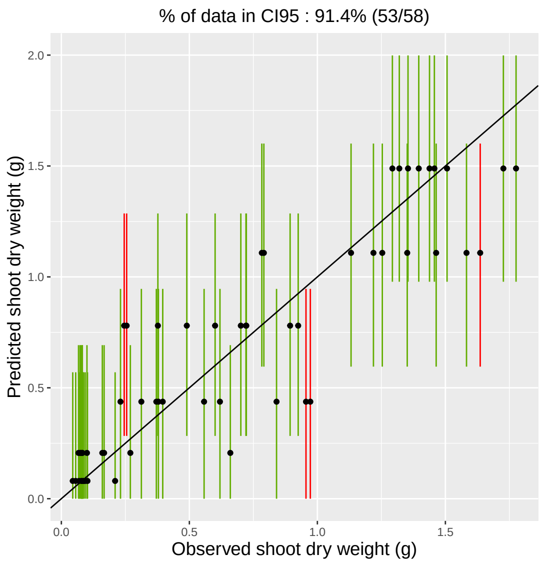

(b) Posterior predictive check (PPC)

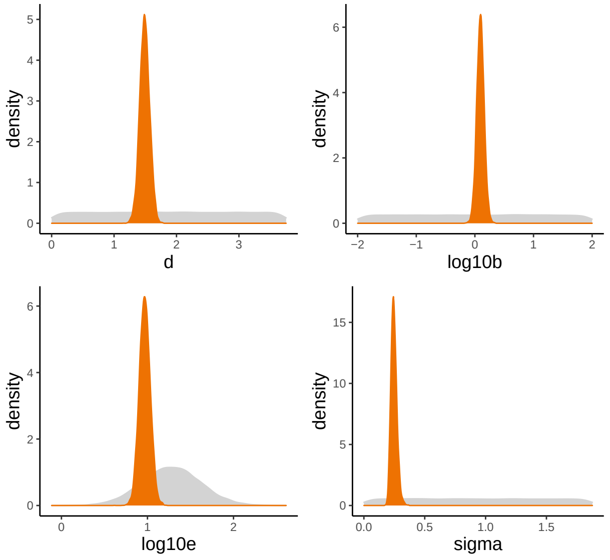

(c) Priors and posteriors

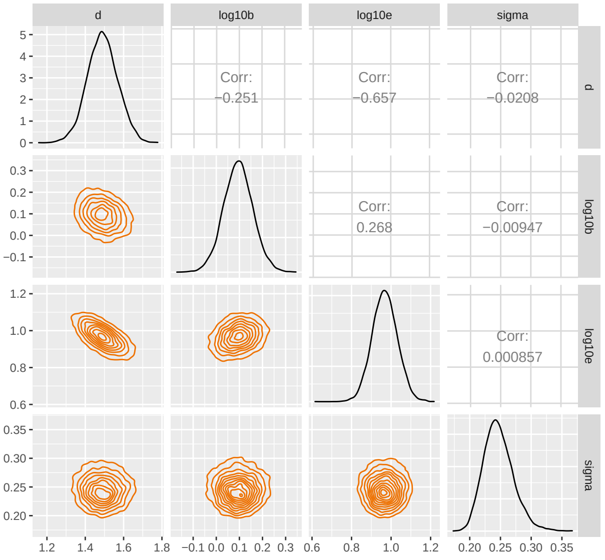

(d) Correlations between parameters

Figure 5: Dose-response curve (a), PPC (b), prior and posterior distributions (c) and correlations between parameters (d).

Data set: GLXMA_SE_weight

Table 6: Summary of parameter estimates for GLXMA_SE_weight data set

Parameter	median	Q2.5	Q97.5
b	2.016	1.391	2.895
d	1.134	1.060	1.213
e	52.546	43.143	64.219
sigma	0.198	0.166	0.242

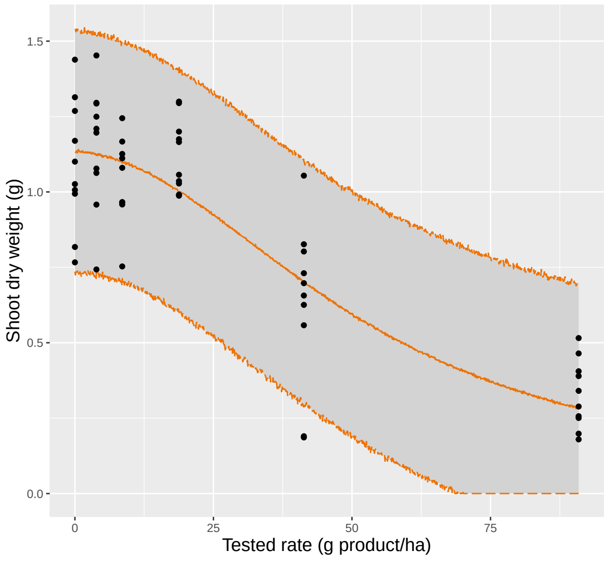

(a) Dose-response curve

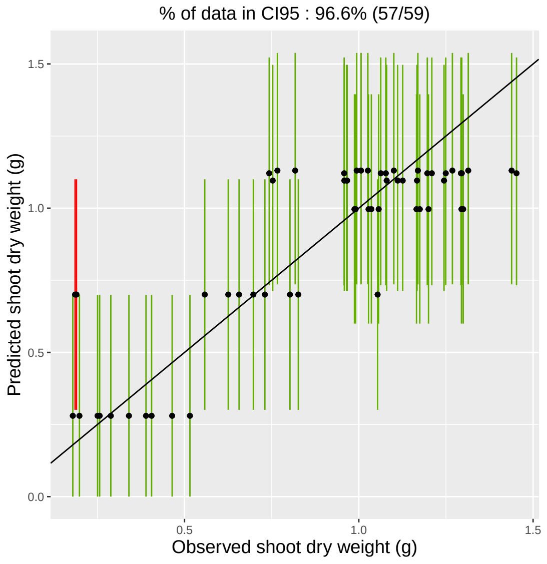

(b) Posterior predictive check (PPC)

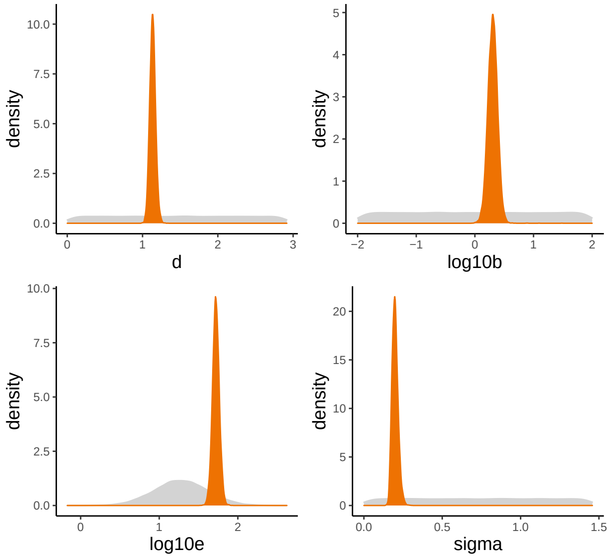

(c) Priors and posteriors

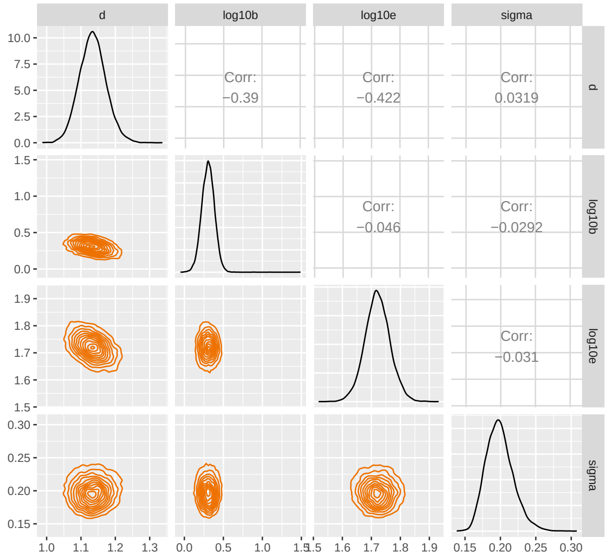

(d) Correlations between parameters

Figure 6: Dose-response curve (a), PPC (b), prior and posterior distributions (c) and correlations between parameters (d).

Data set: HELAN_SE_weight

Table 7: Summary of parameter estimates for HELAN_SE_weight data set

Parameter	median	Q2.5	Q97.5
b	1.319	0.828	2.299
d	0.501	0.431	0.573
e	10.586	7.143	15.550
sigma	0.114	0.095	0.141

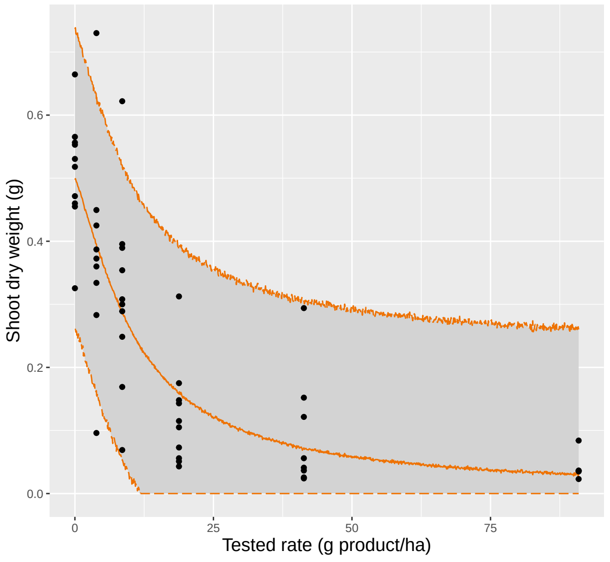

(a) Dose-response curve

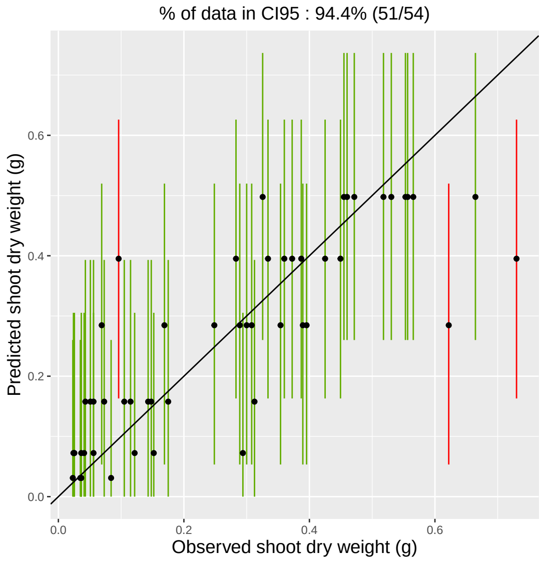

(b) Posterior predictive check (PPC)

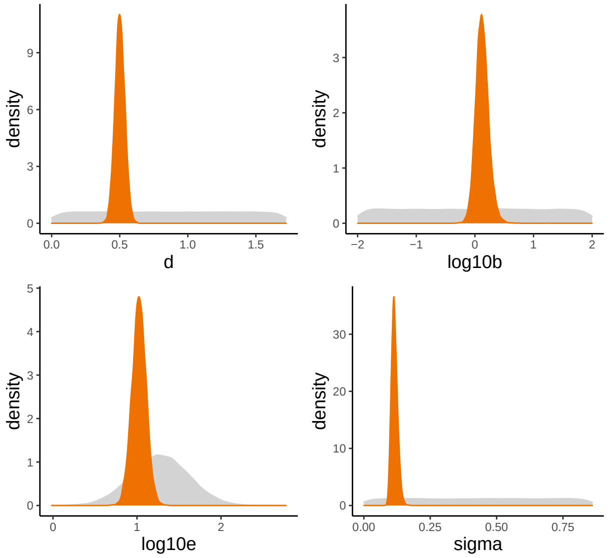

(c) Priors and posteriors

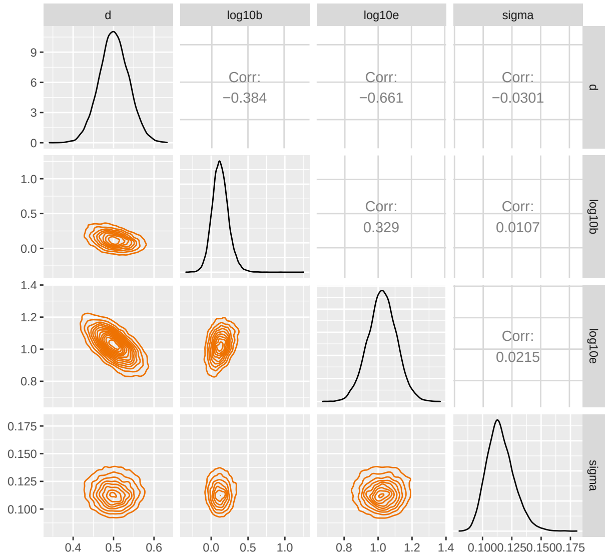

(d) Correlations between parameters

Figure 7: Dose-response curve (a), PPC (b), prior and posterior distributions (c) and correlations between parameters (d).

Data set: LYPES_SE_weight

Table 8: Summary of parameter estimates for LYPES_SE_weight data set

Parameter	median	Q2.5	Q97.5
b	2.270	1.731	3.195
d	1.101	1.029	1.179
e	15.859	13.647	18.116
sigma	0.133	0.111	0.163

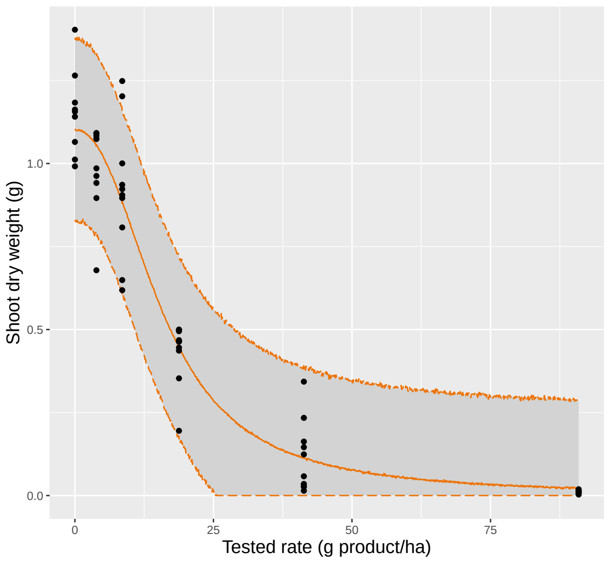

(a) Dose-response curve

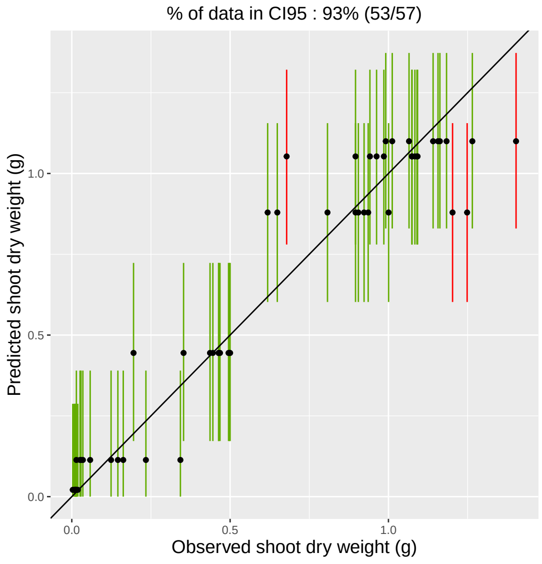

(b) Posterior predictive check (PPC)

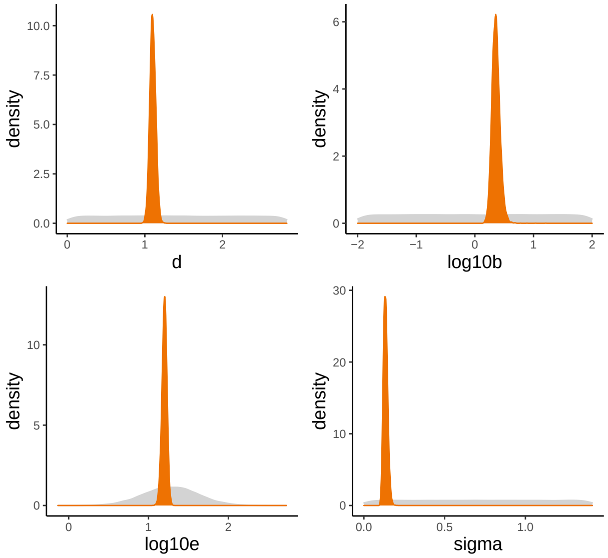

(c) Priors and posteriors

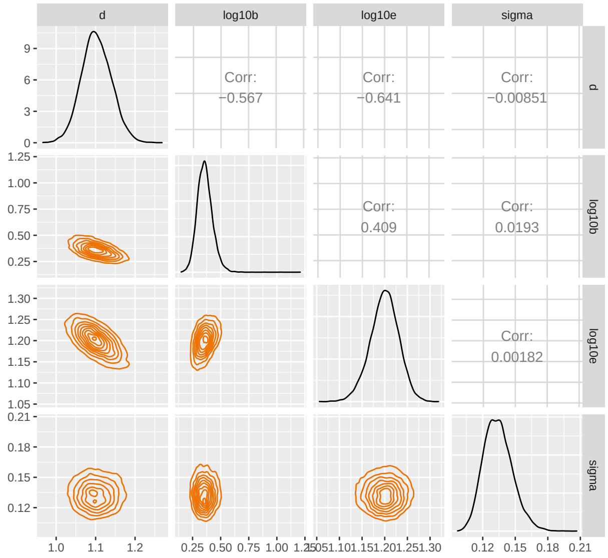

(d) Correlations between parameters

Figure 8: Dose-response curve (a), PPC (b), prior and posterior distributions (c) and correlations between parameters (d).

Data set: TRZAW_SE_weight

Table 9: Summary of parameter estimates for TRZAW_SE_weight data set

Parameter	median	Q2.5	Q97.5
b	0.921	0.609	1.450
d	0.397	0.369	0.431
e	110.828	81.123	164.913
sigma	0.034	0.027	0.047

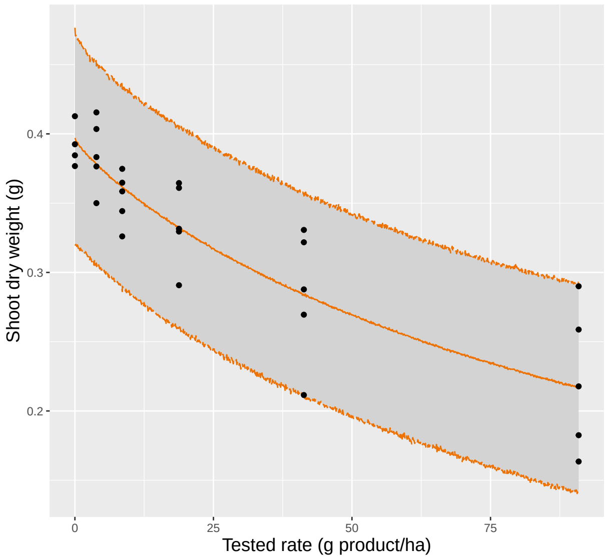

(a) Dose-response curve

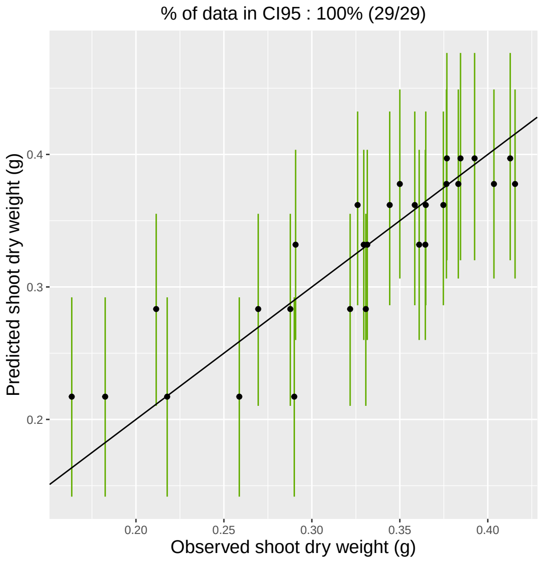

(b) Posterior predictive check (PPC)

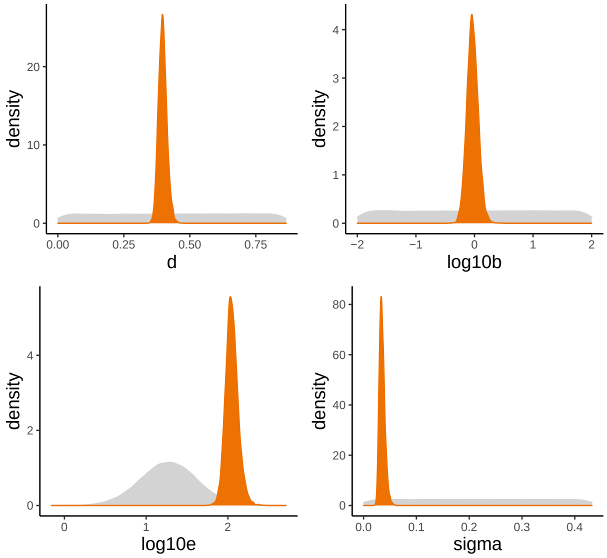

(c) Priors and posteriors

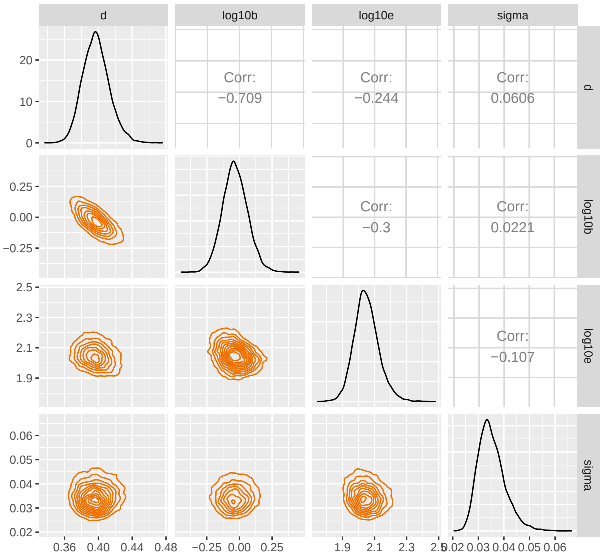

(d) Correlations between parameters

Figure 9: Dose-response curve (a), PPC (b), prior and posterior distributions (c) and correlations between parameters (d).

Data set: ZEAMA_SE_weight

Table 10: Summary of parameter estimates for ZEAMA_SE_weight data set

Parameter	median	Q2.5	Q97.5
b	1.454	1.054	2.039
d	2.509	2.312	2.725
e	37.510	29.265	48.304
sigma	0.425	0.357	0.520

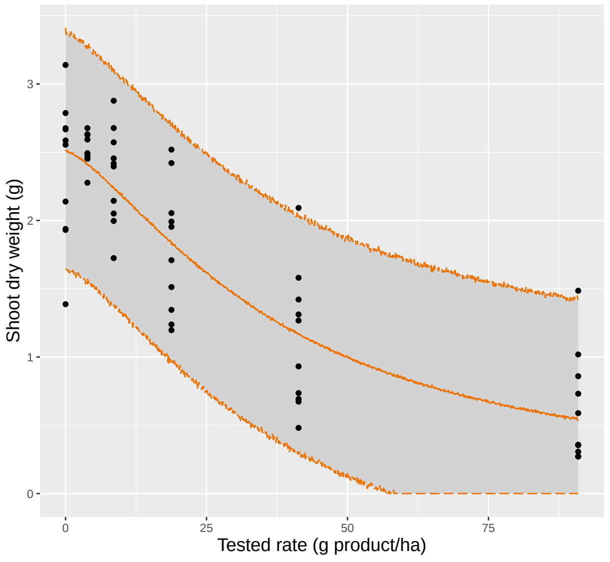

(a) Dose-response curve

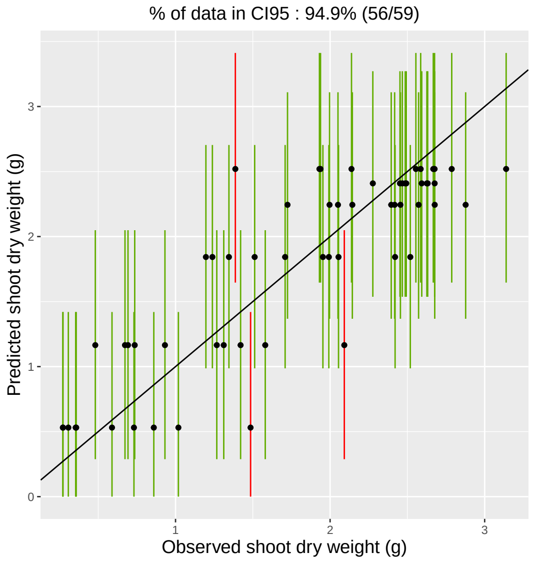

(b) Posterior predictive check (PPC)

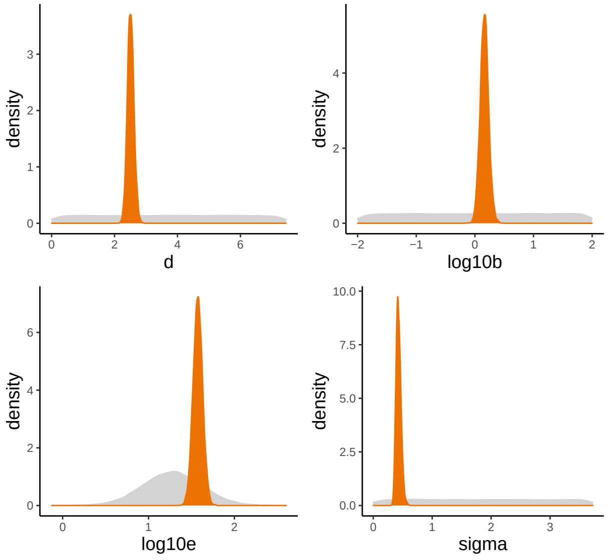

(c) Priors and posteriors

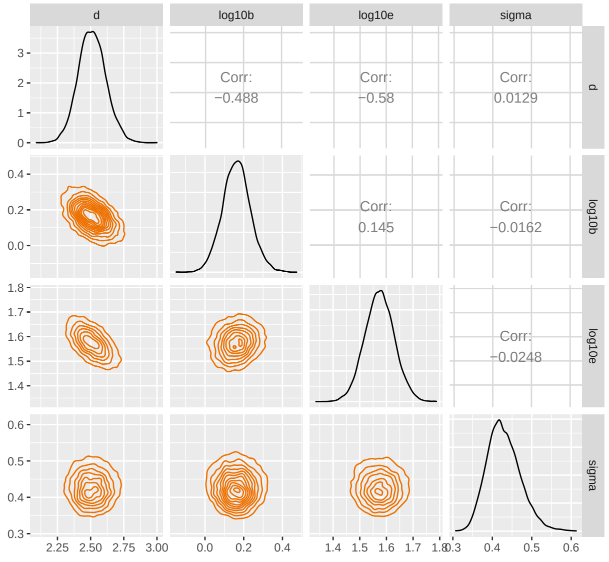

(d) Correlations between parameters

Figure 10: Dose-response curve (a), PPC (b), prior and posterior distributions (c) and correlations between parameters (d).
