# Supplementary material for: How to account for the uncertainty from standard toxicity tests in species sensitivity distributions: An example in non-target plants
Source: PLoS One. 2021 Jan 7;16(1):e0245071. doi: 10.1371/journal.pone.0245071 (PMC7790375; doi:10.1371/journal.pone.0245071)
Supplement: S1 Archive — It is a zip file containing seven folders (one folder per case study). Each folder contains five files report_xxx.pdf with detailed results of the dose-response analyses, one file corresponding to does-response analysis per endpoint. It also contains one file ER50_censoring.pdf for censored ER50 and one file SSD_analyses.pdf for results of SSD analyses. (ZIP) [file pone.0245071.s004.zip › S1_archive/Study7/report_VV_weight.pdf]

Dose-response analysis
Study 7
Vegetative Vigour test - shoot dry VV_weight endpoint

25 June 2020

Contact: sandrine.charles@univ-lyon1.fr

This is a report which provides results on all performed dose-response analyses for the shoot dry VV_weight endpoint of the Vegetative Vigour test for study 7.

Contents

Data set: ALLCE_VV_weight . . . 2
Data set: AVESA_VV_weight . . . 3
Data set: BEAVA_VV_weight . . . 4
Data set: BRSNW_VV_weight . . . 5
Data set: CUMSA_VV_weight . . . 6
Data set: GLXMA_VV_weight . . . 7
Data set: HELAN_VV_weight . . . 8
Data set: LYPES_VV_weight . . . 9
Data set: TRZAW_VV_weight . . . 10
Data set: ZEAMA_VV_weight . . . 11

Data set: ALLCE_VV_weight

Table 1: Summary of parameter estimates for ALLCE_VV_weight data set

Parameter	median	Q2.5	Q97.5
b	2.650	1.198	21.399
d	0.216	0.195	0.241
e	37.965	27.929	50.457
sigma	0.042	0.033	0.057

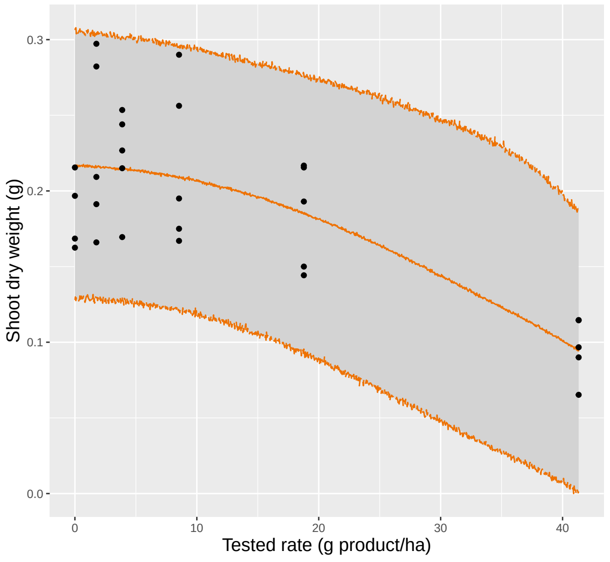

(a) Dose-response curve

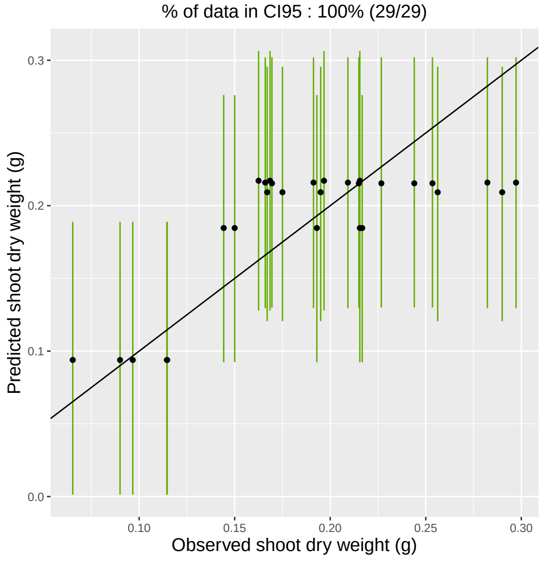

(b) Posterior predictive check (PPC)

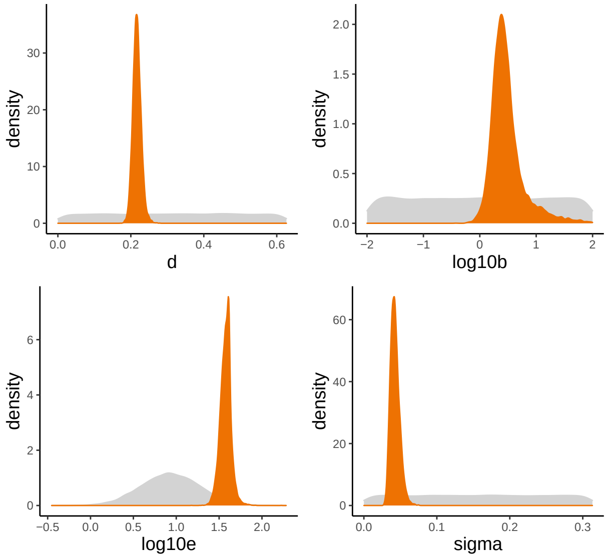

(c) Priors and posteriors

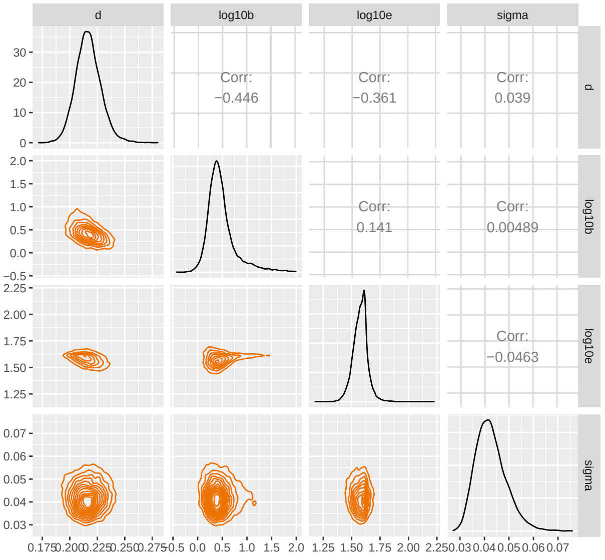

(d) Correlations between parameters

Figure 1: Dose-response curve (a), PPC (b), prior and posterior distributions (c) and correlations between parameters (d).

Data set: AVESA_VV_weight

Table 2: Summary of parameter estimates for AVESA_VV_weight data set

Parameter	median	Q2.5	Q97.5
b	3.202	1.922	9.033
d	1.706	1.573	1.842
e	29.872	23.641	38.034
sigma	0.274	0.211	0.376

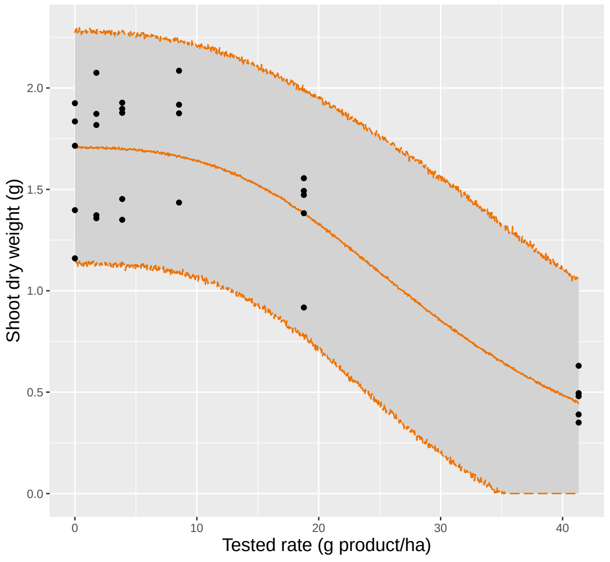

(a) Dose-response curve

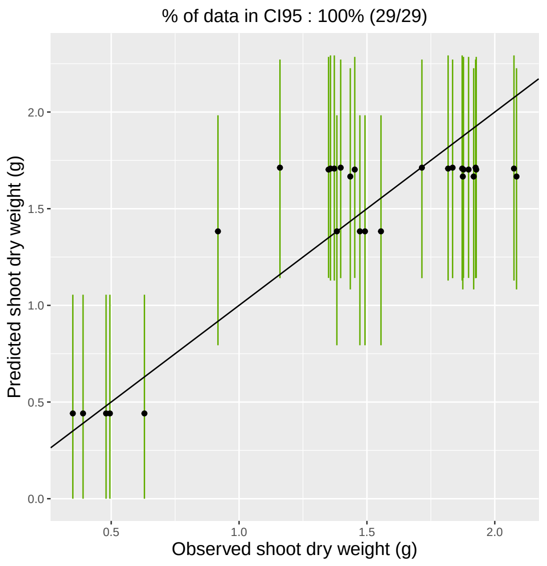

(b) Posterior predictive check (PPC)

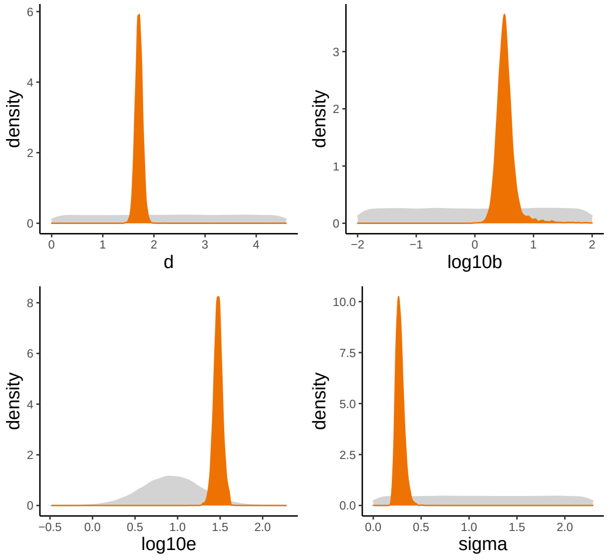

(c) Priors and posteriors

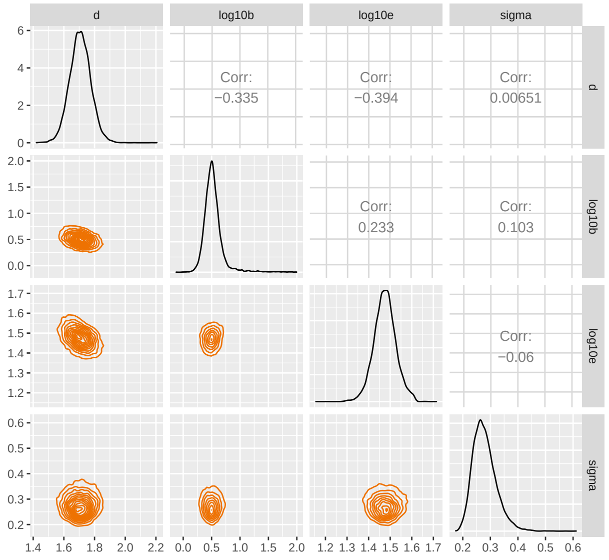

(d) Correlations between parameters

Figure 2: Dose-response curve (a), PPC (b), prior and posterior distributions (c) and correlations between parameters (d).

Data set: BEAVA_VV_weight

Table 3: Summary of parameter estimates for BEAVA_VV_weight data set

Parameter	median	Q2.5	Q97.5
b	1.865	1.316	2.827
d	2.255	2.052	2.472
e	1.756	1.406	2.216
sigma	0.412	0.344	0.510

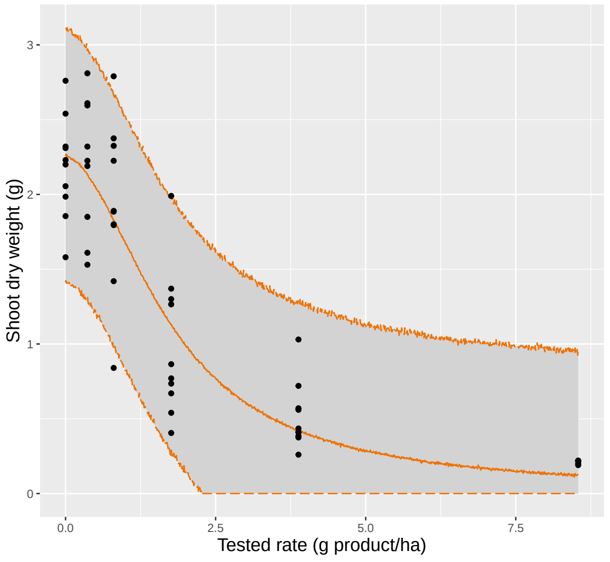

(a) Dose-response curve

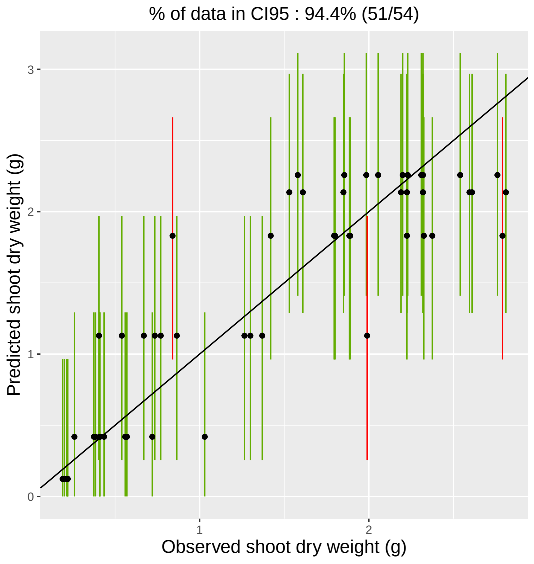

(b) Posterior predictive check (PPC)

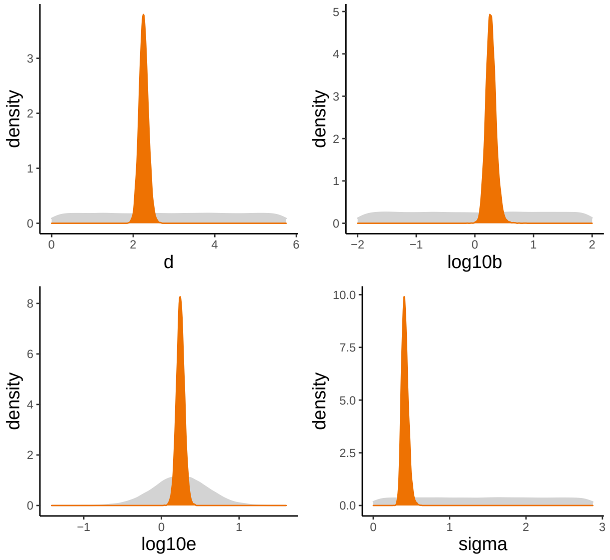

(c) Priors and posteriors

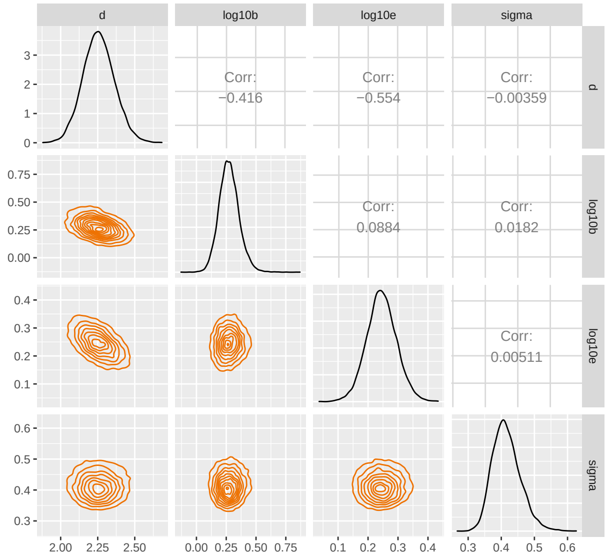

(d) Correlations between parameters

Figure 3: Dose-response curve (a), PPC (b), prior and posterior distributions (c) and correlations between parameters (d).

Data set: BRSNW_VV_weight

Table 4: Summary of parameter estimates for BRSNW_VV_weight data set

Parameter	median	Q2.5	Q97.5
b	3.021	2.167	7.829
d	4.810	4.557	5.070
e	4.066	3.600	4.594
sigma	0.692	0.582	0.846

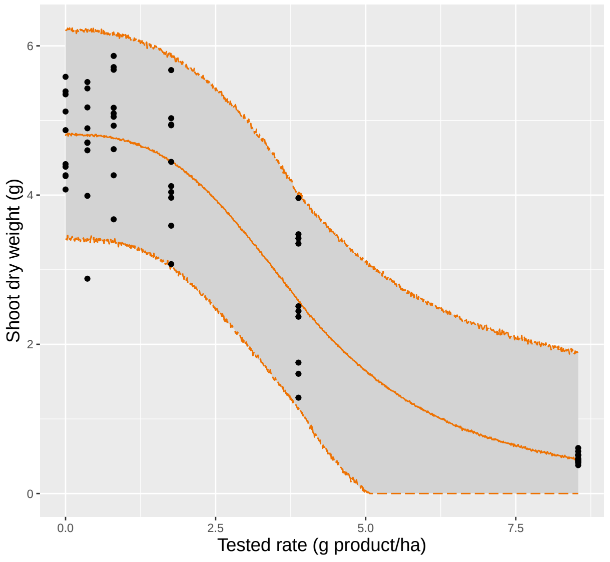

(a) Dose-response curve

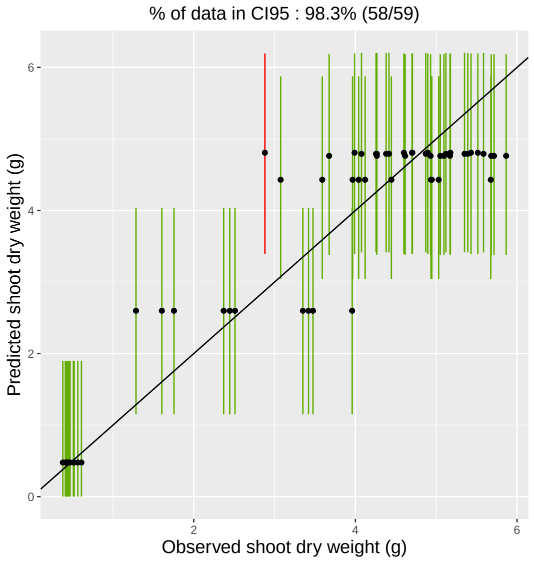

(b) Posterior predictive check (PPC)

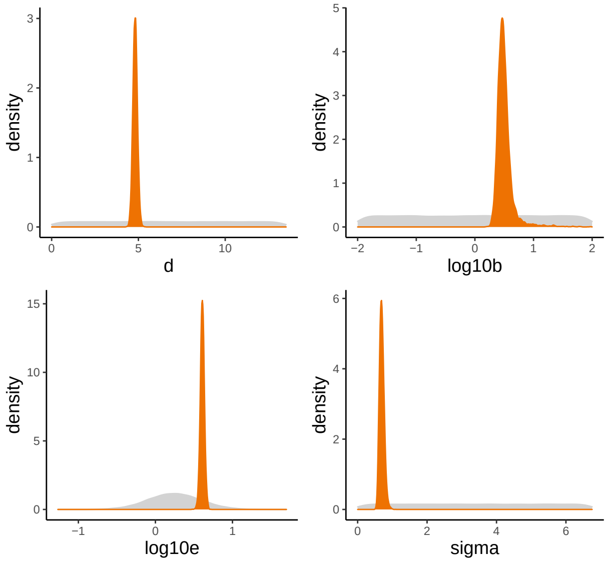

(c) Priors and posteriors

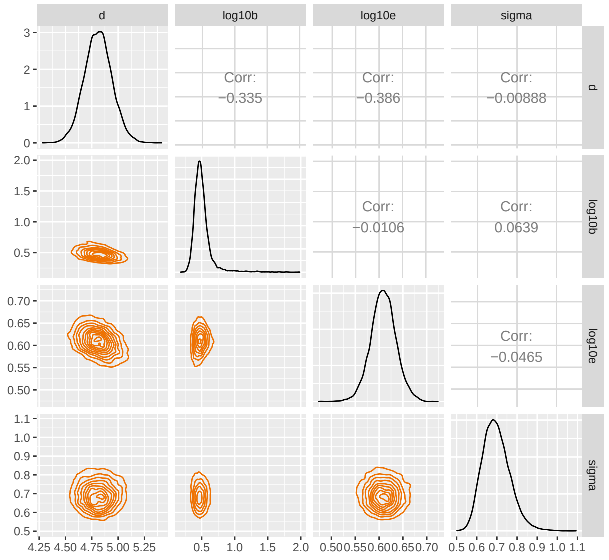

(d) Correlations between parameters

Figure 4: Dose-response curve (a), PPC (b), prior and posterior distributions (c) and correlations between parameters (d).

Data set: CUMSA_VV_weight

Table 5: Summary of parameter estimates for CUMSA_VV_weight data set

Parameter	median	Q2.5	Q97.5
b	0.822	0.632	1.052
d	9.891	9.105	10.687
e	9.378	6.944	12.906
sigma	1.250	1.047	1.541

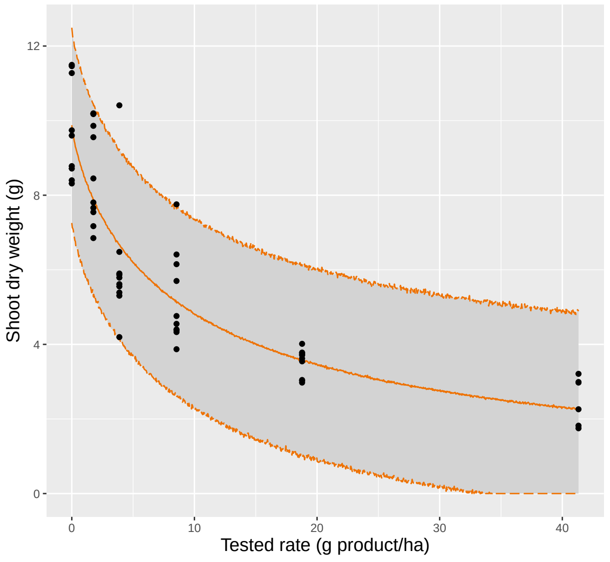

(a) Dose-response curve

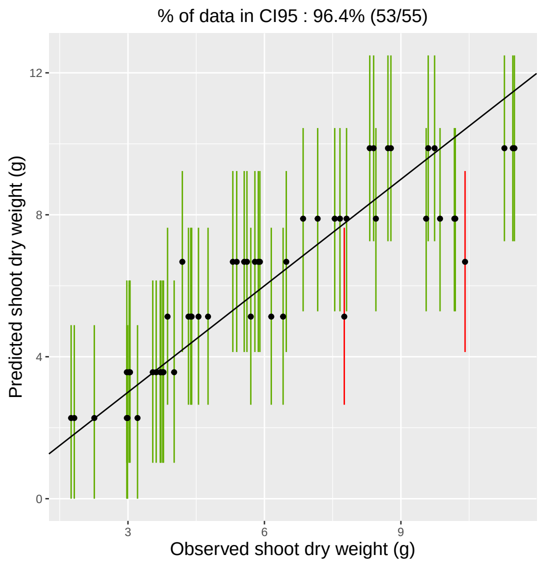

(b) Posterior predictive check (PPC)

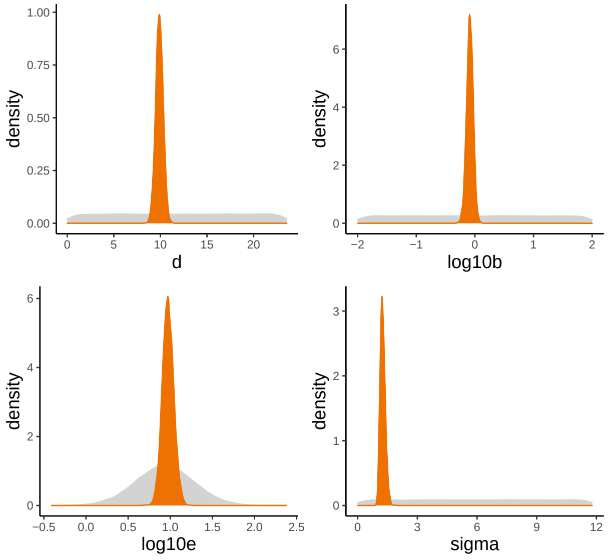

(c) Priors and posteriors

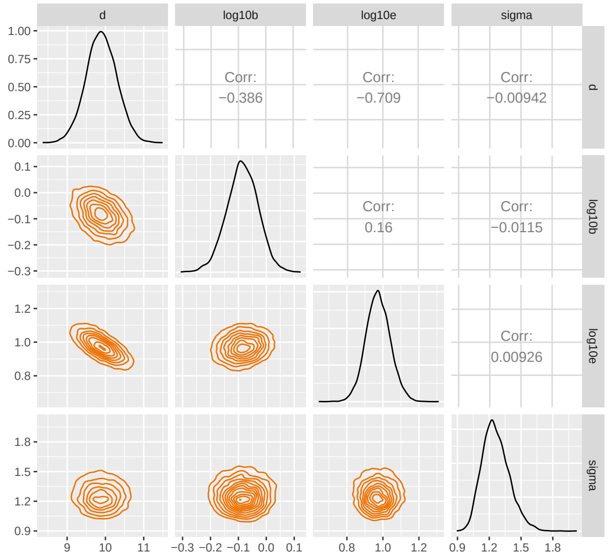

(d) Correlations between parameters

Figure 5: Dose-response curve (a), PPC (b), prior and posterior distributions (c) and correlations between parameters (d).

Data set: GLXMA_VV_weight

Table 6: Summary of parameter estimates for GLXMA_VV_weight data set

Parameter	median	Q2.5	Q97.5
b	0.902	0.730	1.102
d	3.071	2.863	3.280
e	8.119	6.373	10.194
sigma	0.315	0.264	0.383

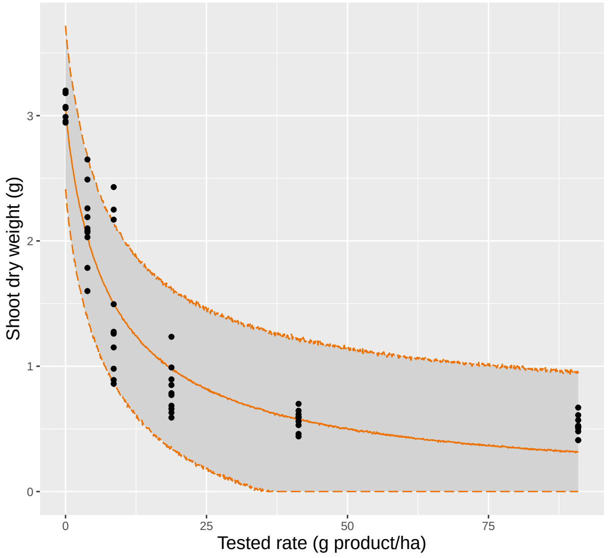

(a) Dose-response curve

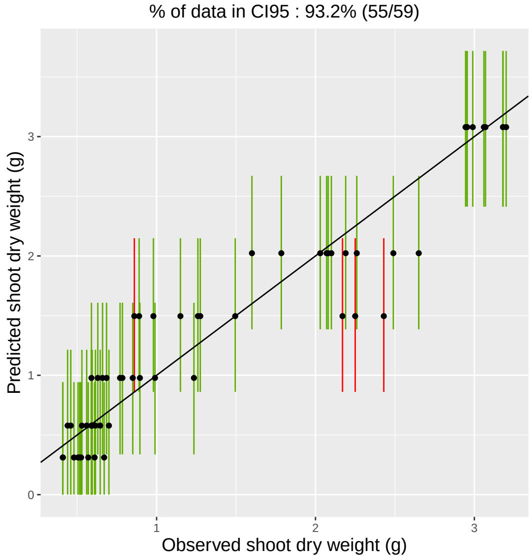

(b) Posterior predictive check (PPC)

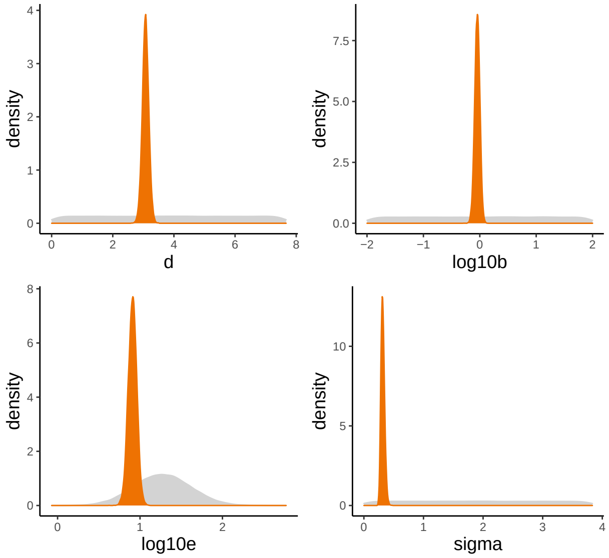

(c) Priors and posteriors

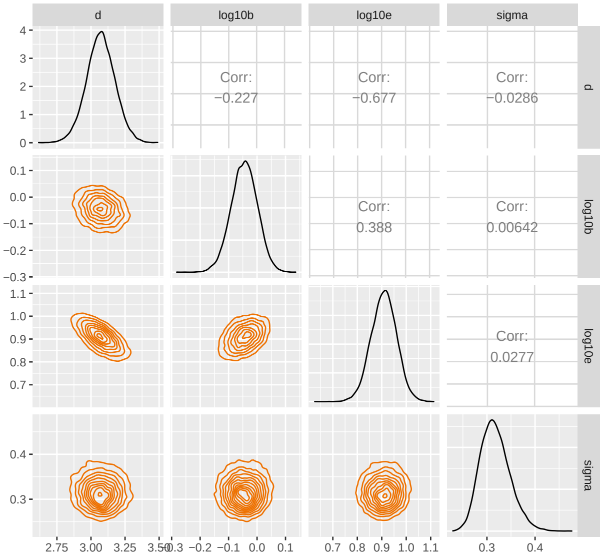

(d) Correlations between parameters

Figure 6: Dose-response curve (a), PPC (b), prior and posterior distributions (c) and correlations between parameters (d).

Data set: HELAN_VV_weight

Table 7: Summary of parameter estimates for HELAN_VV_weight data set

Parameter	median	Q2.5	Q97.5
b	2.634	1.756	4.070
d	1.742	1.585	1.907
e	5.172	4.319	6.280
sigma	0.299	0.238	0.389

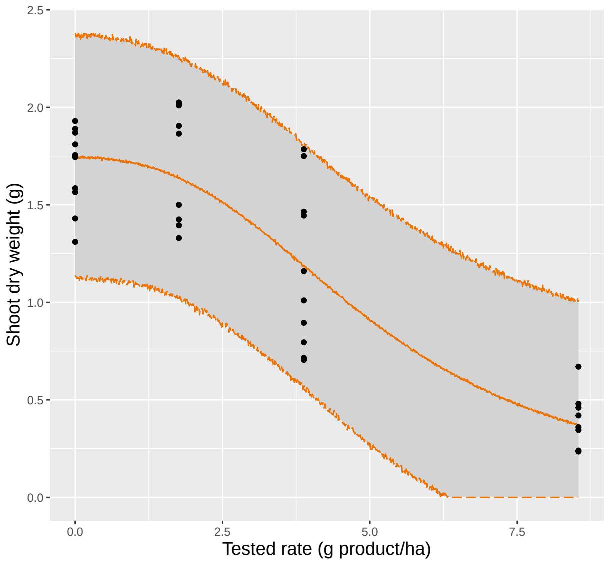

(a) Dose-response curve

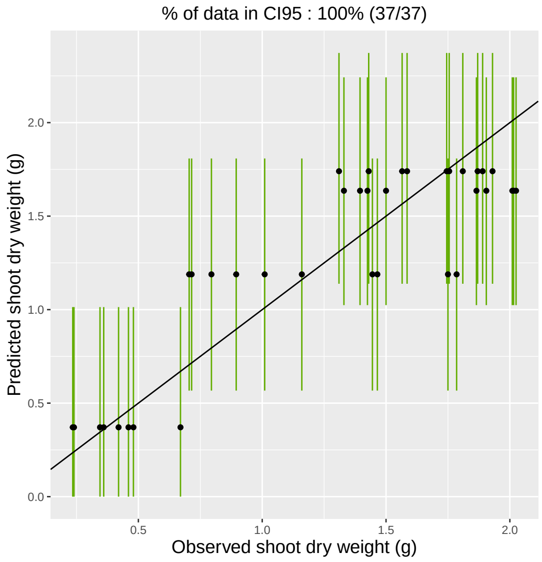

(b) Posterior predictive check (PPC)

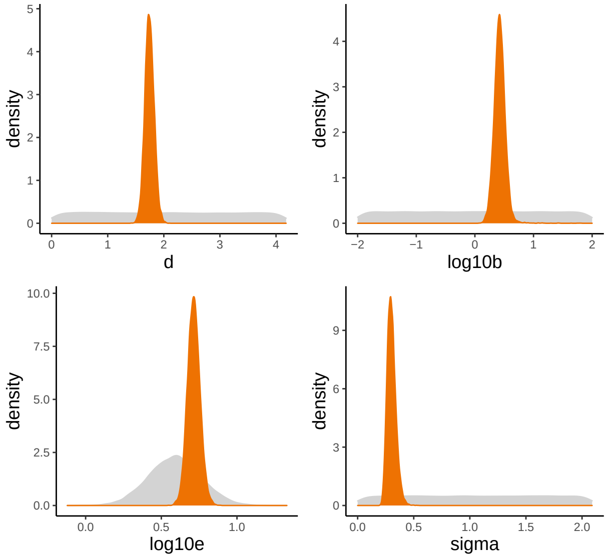

(c) Priors and posteriors

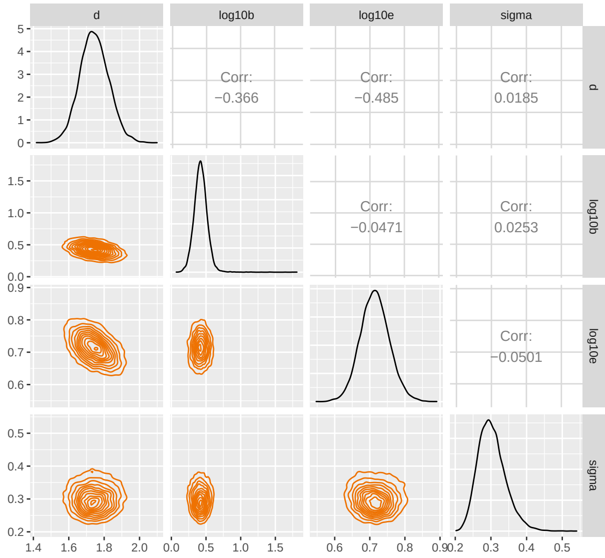

(d) Correlations between parameters

Figure 7: Dose-response curve (a), PPC (b), prior and posterior distributions (c) and correlations between parameters (d).

Data set: LYPES_VV_weight

Table 8: Summary of parameter estimates for LYPES_VV_weight data set

Parameter	median	Q2.5	Q97.5
b	2.078	1.676	2.642
d	4.098	3.850	4.358
e	4.989	4.388	5.634
sigma	0.412	0.344	0.508

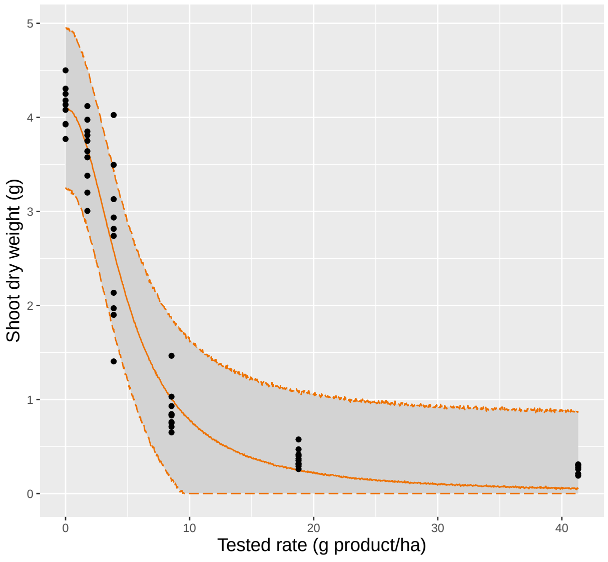

(a) Dose-response curve

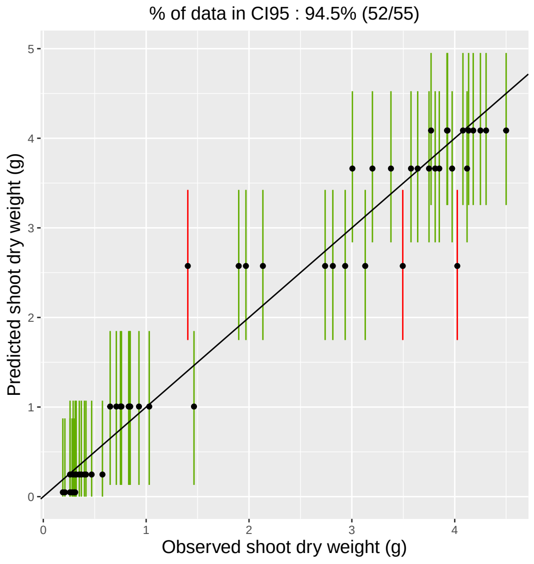

(b) Posterior predictive check (PPC)

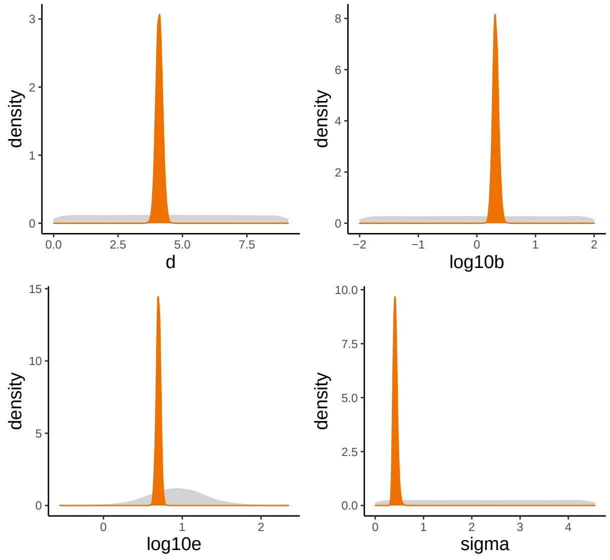

(c) Priors and posteriors

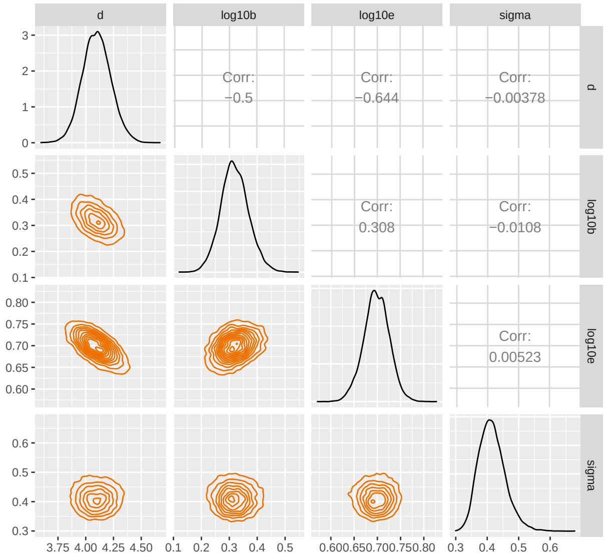

(d) Correlations between parameters

Figure 8: Dose-response curve (a), PPC (b), prior and posterior distributions (c) and correlations between parameters (d).

Data set: TRZAW_VV_weight

Table 9: Summary of parameter estimates for TRZAW_VV_weight data set

Parameter	median	Q2.5	Q97.5
b	14.210	2.099	89.264
d	0.624	0.604	0.645
e	123.307	94.553	290.279
sigma	0.047	0.036	0.063

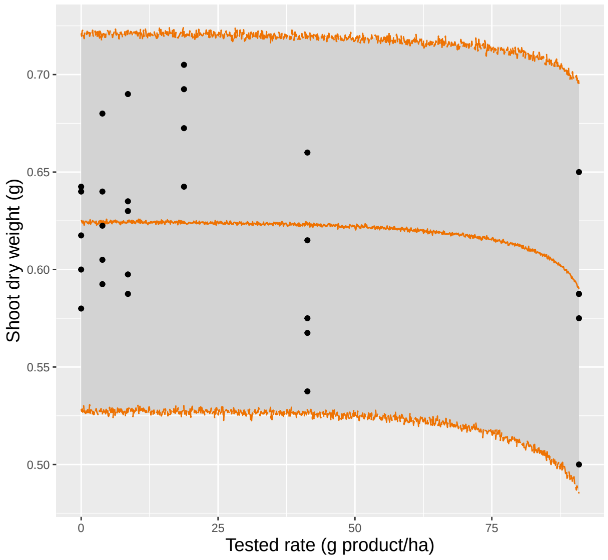

(a) Dose-response curve

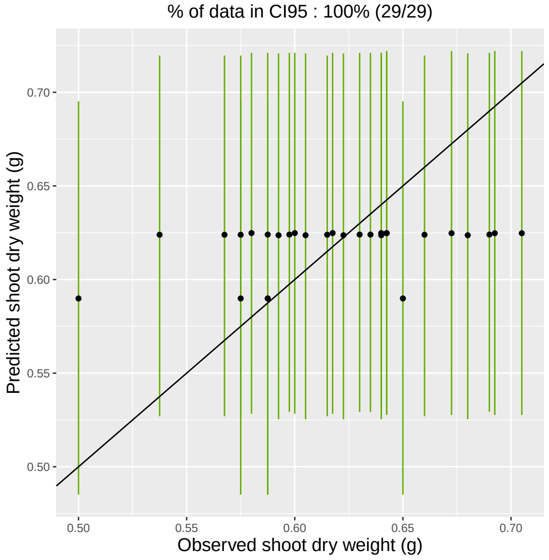

(b) Posterior predictive check (PPC)

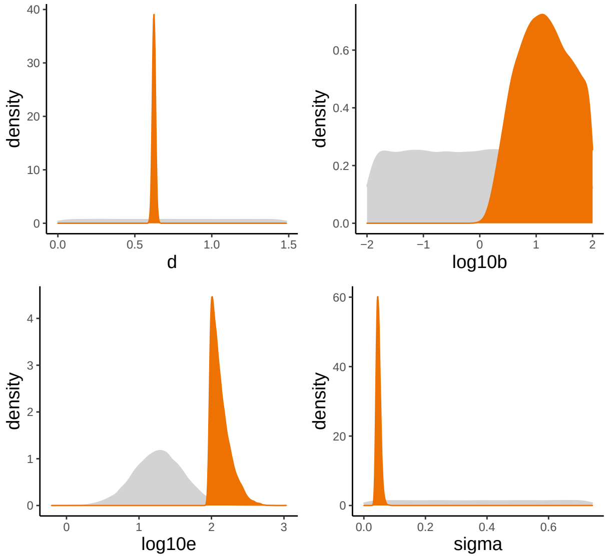

(c) Priors and posteriors

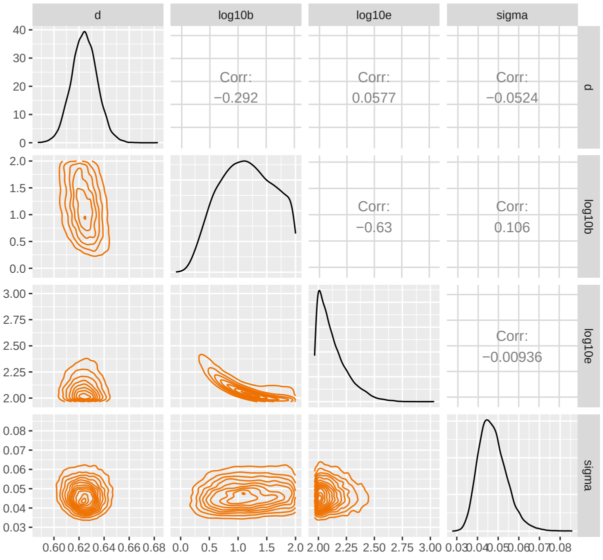

(d) Correlations between parameters

Figure 9: Dose-response curve (a), PPC (b), prior and posterior distributions (c) and correlations between parameters (d).

Data set: ZEAMA_VV_weight

Table 10: Summary of parameter estimates for ZEAMA_VV_weight data set

Parameter	median	Q2.5	Q97.5
b	2.644	1.659	12.564
d	4.669	4.453	4.900
e	94.766	83.962	112.980
sigma	0.634	0.528	0.777

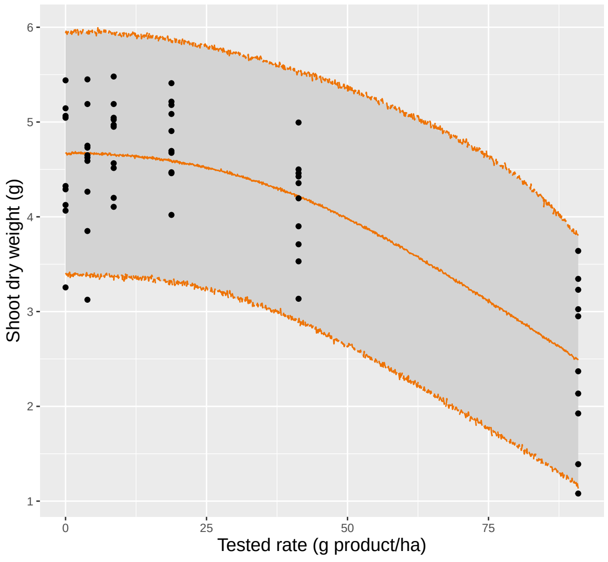

(a) Dose-response curve

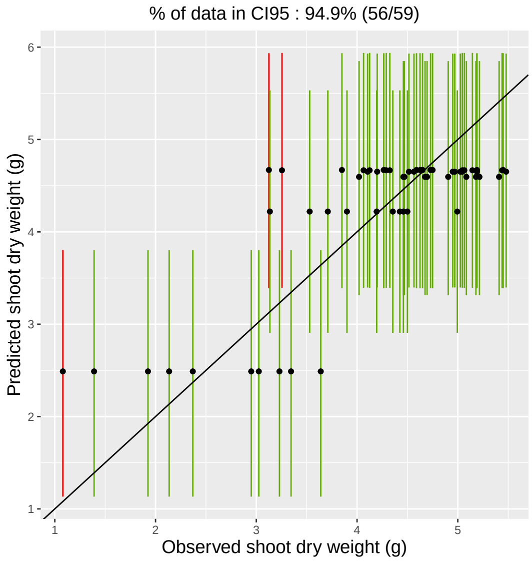

(b) Posterior predictive check (PPC)

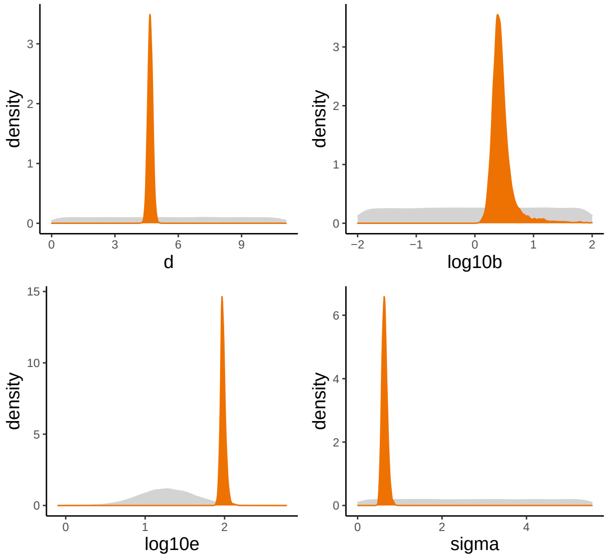

(c) Priors and posteriors

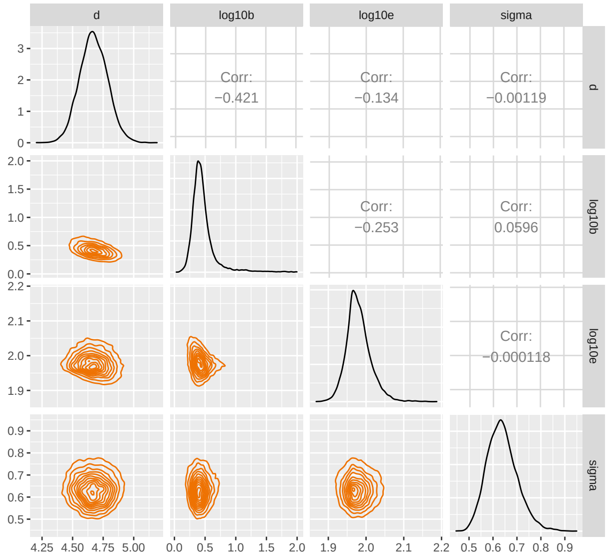

(d) Correlations between parameters

Figure 10: Dose-response curve (a), PPC (b), prior and posterior distributions (c) and correlations between parameters (d).
