# Supplementary material for: How to account for the uncertainty from standard toxicity tests in species sensitivity distributions: An example in non-target plants
Source: PLoS One. 2021 Jan 7;16(1):e0245071. doi: 10.1371/journal.pone.0245071 (PMC7790375; doi:10.1371/journal.pone.0245071)
Supplement: S1 Archive — It is a zip file containing seven folders (one folder per case study). Each folder contains five files report_xxx.pdf with detailed results of the dose-response analyses, one file corresponding to does-response analysis per endpoint. It also contains one file ER50_censoring.pdf for censored ER50 and one file SSD_analyses.pdf for results of SSD analyses. (ZIP) [file pone.0245071.s004.zip › S1_archive/Study7/SSD_analyses.pdf]

Species sensitivity distribution (SSD) analyses by fitting a log-normal distribution

Study 7

25 June 2020

Contact: sandrine.charles@univ-lyon1.fr

The document provides results on species sensitivity distribution (SSD) analyses for study 7. There are five endpoints for study 7: emergence, survival, shoot dry weight of the seedling emergence (SE) test, and survival and shoot dry weight of vegetative vigour (VV) test.

We used SSD to describe the toxicity variation of herbicides among a set of non-target terrestrial plants (NTTP). Based on this SSD, the hazardous rate at which five percent of the species are affected (HR_5) can be estimated. This document aims to compare SSD and HR_5 based on 50% effective rates (ER_{50}) generated in three different ways. We considered:

1. Point estimates (medians of posterior distributions of ER_{50}) as inputs for SSD analyses;
2. Interval-censored ER_{50} by 95% credible interval (CI95);
3. censored ER_{50} according to criterion 1 with a decision threshold T = 0.5

For each endpoint, a total of three SSD are produced:

1. SSD based on medians of posterior distributions of ER_{50} (figure title: median);
2. SSD based on CI95 of ER_{50} (figure title: CI95);
3. SSD based on censored ER_{50} according to criterion 1 with T = 0.5 (figure title: C1T0.5).

Meaning of symbols used in Tables 1-5:

- nb_ER_{50} (RC): number of ER_{50} (number of right-censored ER_{50});
- nb_distER_{50} (RC): number of distinct ER_{50} (number of distinct right-censored ER_{50});
- HR_5_est: HR_5 mean estimate;
- HR_5_median: median of HR_5;
- HR_5_LCoI95: lower bound of the 95% confidence interval (CoI95) of HR_5;
- HR_5_UCoI95: upper bound of the CoI95 of HR_5.

Meaning of legends, lines or curves in Figures 1-5:

- Nb. ER_{50} (RC): number of ER_{50} (number of right-censored ER_{50});
- Nb. distinct ER_{50} (RC): number of distinct ER_{50} (number of distinct right-censored ER_{50});
- HR_5 [CoI95]: HR_5 mean estimate [CoI95 of HR_5];
- Blue points: fractions of affected species (EPPO code) ordered by the median of ER_{50};
- Solid blue horizontal segment: interval-censored ER_{50} by the CI95 of the ER_{50};
- Dotted blue horizontal segment: right-censored ER_{50};
- Solid red curve: fitted SSD curve;
- Dotted red curves: CoI95 of the fitted SSD curve.

HR_5 results and SSD curves

HR_5 and SSD for the emergence endpiont of the SE test of study 7

Table 1: Number of (distinct) ER_{50} (right-censored) and HR_5 estimate for the emergence endpoint of the SE test of study 7 (tested rate unit: g product/ha)

criterion	nb_ER_{50} (RC)	nb_distER_{50} (RC)	HR_5_est	HR_5_median	HR_5_LCoI95	HR_5_UCoI95	CV
median	10 (0)	10 (0)	73.879	77.864	61.307	103.565	0.139
CI95	10 (0)	10 (0)	151.674	159.269	131.520	235.401	0.180
C1T0.5	10 (10)	3 (3)	302.396	302.396	302.396	302.396	0.000

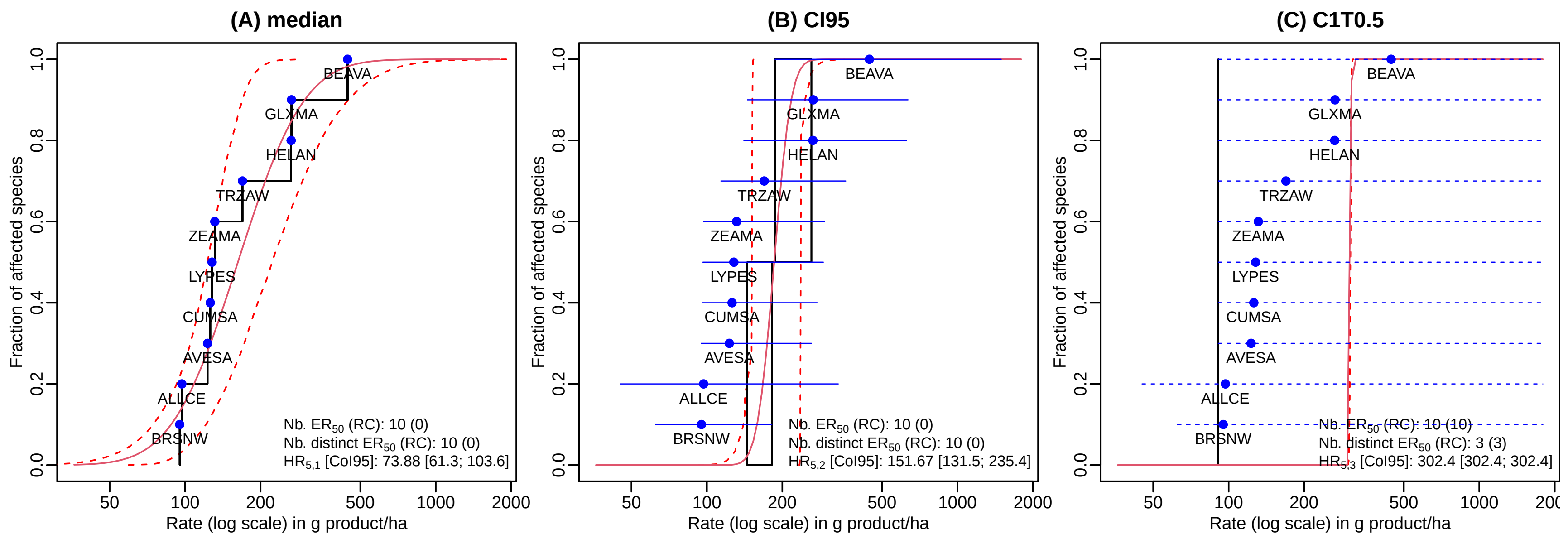

Figure 1: Example of three SSD based on respectively ER_{50} medians (A), CI95 of ER_{50} (B) and censored ER_{50} according to C1T0.5 (C) for the emergnece endpoint of the SE test of study 7.

HR_5 and SSD for the survival endpiont of the SE test of study 7

Table 2: Number of (distinct) ER_{50} (right-censored) and HR_5 estimate for the survival endpoint of the SE test of study 7 (tested rate unit: g product/ha)

criterion	nb_ER_{50} (RC)	nb_distER_{50} (RC)	HR_5_est	HR_5_median	HR_5_LCoI95	HR_5_UCoI95	CV
median	10 (0)	10 (0)	7.758	8.256	1.734	67.839	1.105
CI95	10 (0)	10 (0)	7.658	8.075	1.659	76.363	1.315
C1T0.5	10 (5)	7 (2)	5.275	5.927	0.992	80.099	1.727

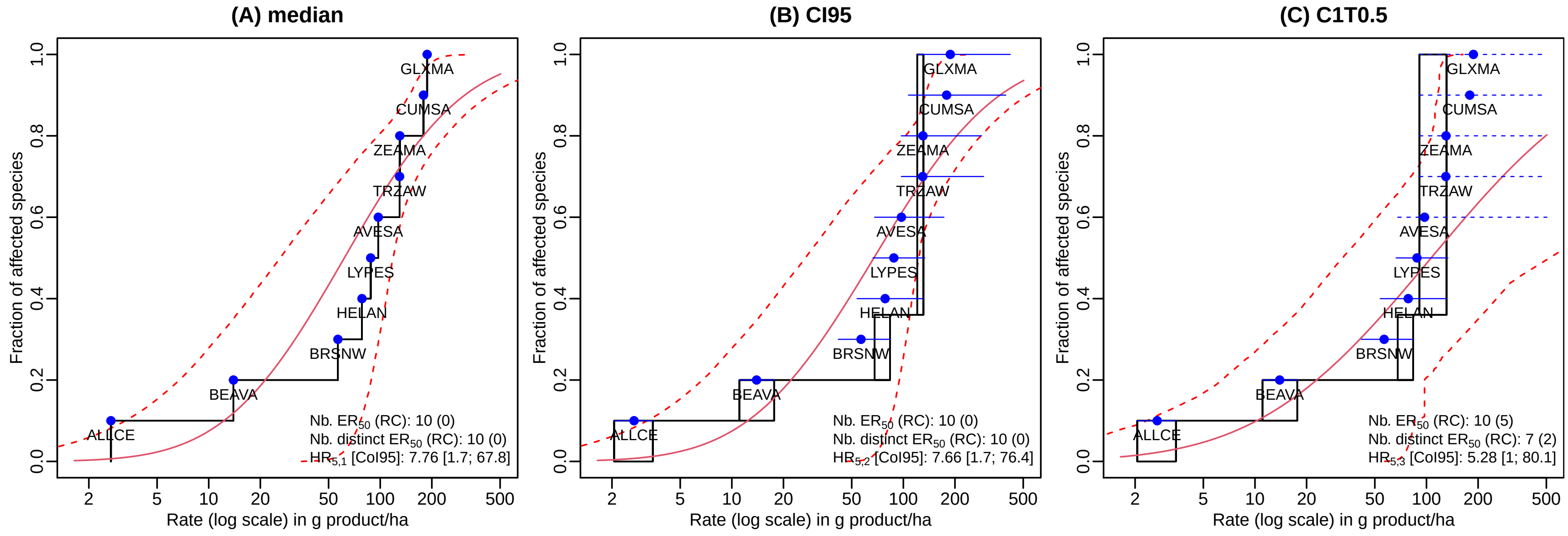

Figure 2: Example of three SSD based on respectively ER_{50} medians (A), CI95 of ER_{50} (B) and censored ER_{50} according to C1T0.5 (C) for the survival endpoint of the SE test of study 7.

HR_5 and SSD for the shoot dry weight endpiont of the SE test of study 7

Table 3: Number of (distinct) ER_{50} (right-censored) and HR_5 estimate for the shoot dry weight endpoint of the SE test of study 7 (tested rate unit: g product/ha)

criterion	nb_ER_{50} (RC)	nb_distER_{50} (RC)	HR_5_est	HR_5_median	HR_5_LCoI95	HR_5_UCoI95	CV
median	10 (0)	10 (0)	1.062	1.198	0.210	6.958	0.945
CI95	10 (0)	10 (0)	1.553	1.829	0.305	6.965	0.777
C1T0.5	10 (1)	10 (1)	1.401	1.707	0.240	6.885	0.809

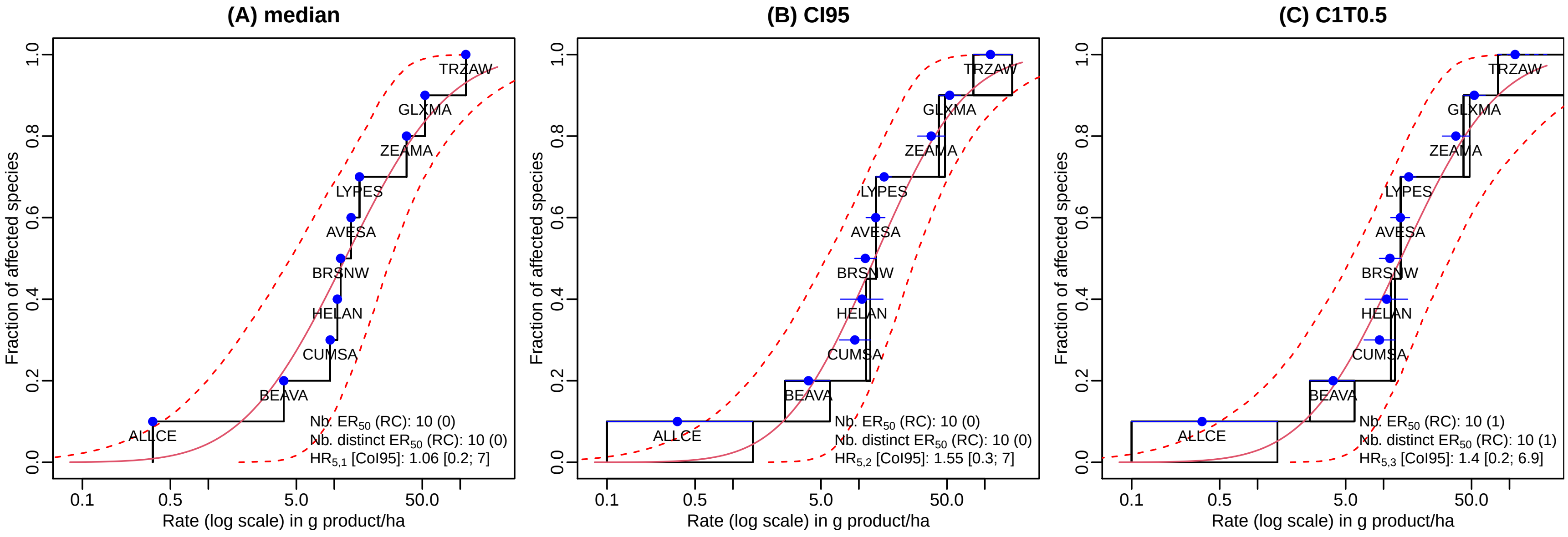

Figure 3: Example of three SSD based on respectively ER_{50} medians (A), CI95 of ER_{50} (B) and censored ER_{50} according to C1T0.5 (C) for the shoot dry weight endpoint of the SE test of study 7.

HR_5 and SSD for the survival endpiont of the VV test of study 7

Table 4: Number of (distinct) ER_{50} (right-censored) and HR_5 estimate for the survival endpoint of the VV test of study 7 (tested rate unit: g product/ha)

criterion	nb_ER_{50} (RC)	nb_distER_{50} (RC)	HR_5_est	HR_5_median	HR_5_LCoI95	HR_5_UCoI95	CV
median	10 (0)	10 (0)	6.341	6.587	3.258	22.568	0.597
CI95	10 (0)	10 (0)	6.516	6.903	3.280	25.679	0.650
C1T0.5	10 (6)	7 (3)	5.612	5.927	2.314	35.396	4.628

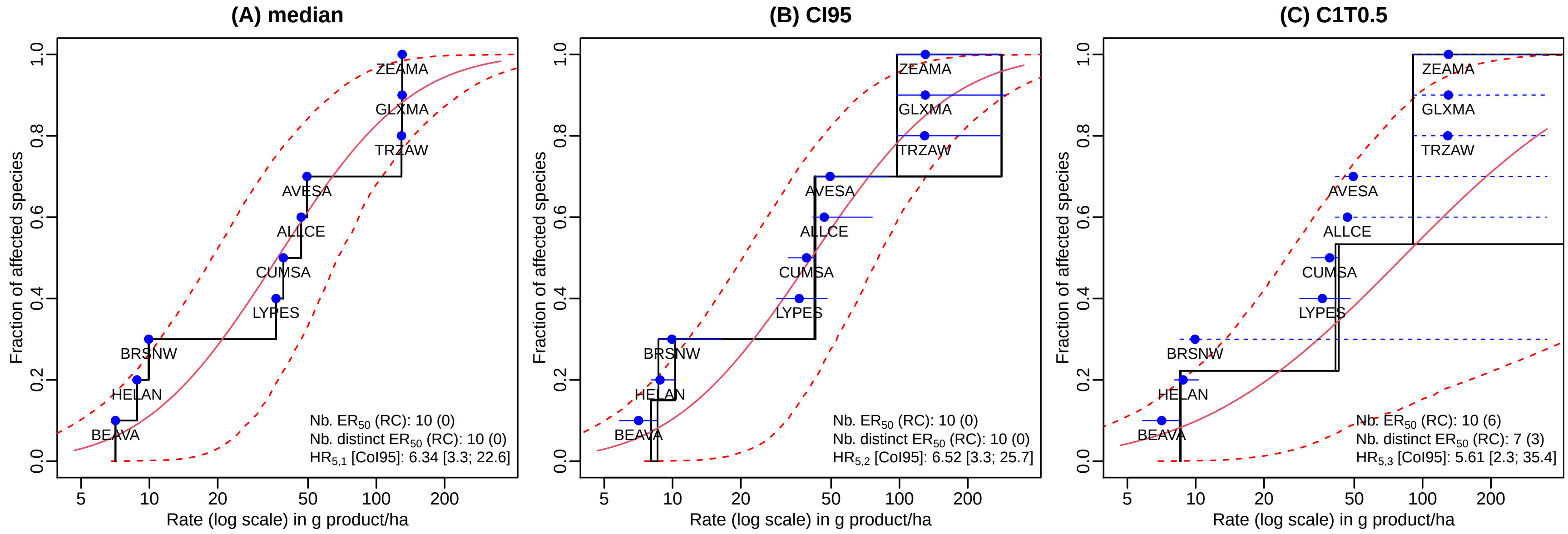

Figure 4: Example of three SSD based on respectively ER_{50} medians (A), CI95 of ER_{50} (B) and censored ER_{50} according to C1T0.5 (C) for the survival endpoint of the VV test of study 7.

HR_5 and SSD for the shoot dry weight endpiont of the VV test of study 7

Table 5: Number of (distinct) ER_{50} (right-censored) and HR_5 estimate for the shoot dry weight endpoint of the VV test of study 7 (tested rate unit: g product/ha)

criterion	nb_ER$_{50}$ (RC)	nb_distER$_{50}$ (RC)	HR$_5$_est	HR$_5$_median	HR$_5$_LCoI95	HR$_5$_UCoI95	CV
median	10 (0)	10 (0)	1.448	1.652	0.720	4.423	0.553
CI95	10 (0)	10 (0)	1.402	1.583	0.725	4.510	0.602
C1T0.5	10 (2)	10 (2)	1.152	1.299	0.496	3.481	0.572

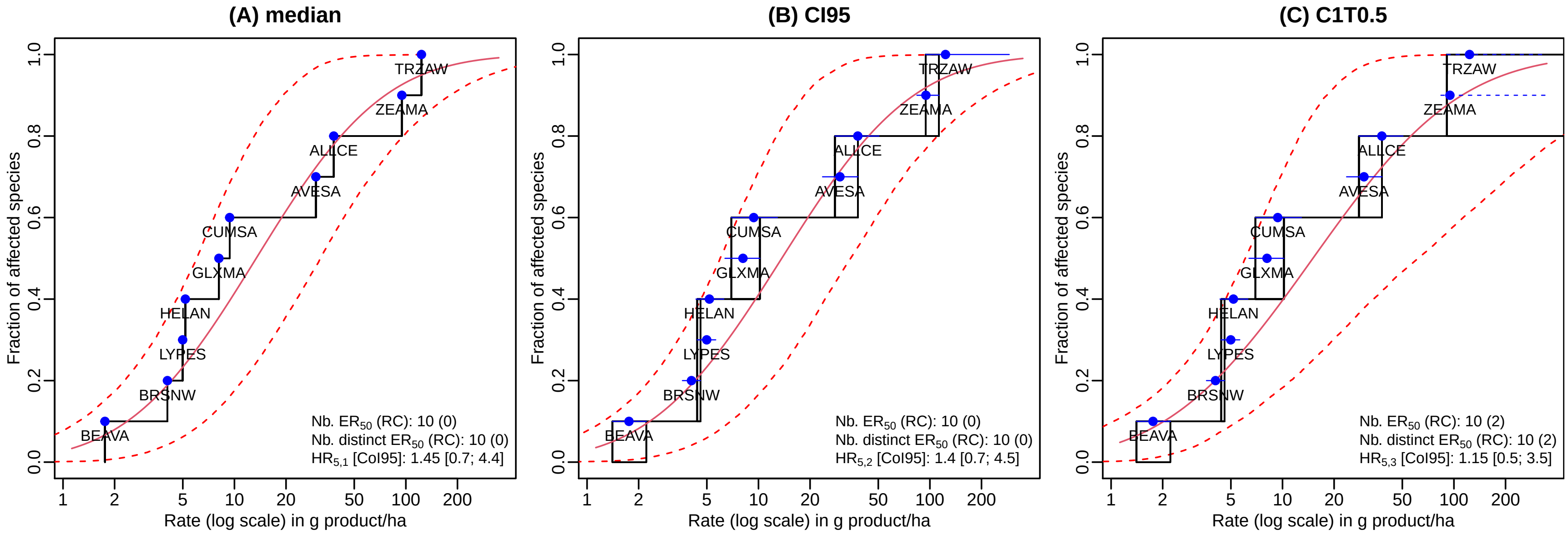

Figure 5: Example of three SSD based on respectively ER_{50} medians (A), CI95 of ER_{50} (B) and censored ER_{50} according to C1T0.5 (C) for the shoot dry weight endpoint of the VV test of study 7.
