# Supplementary material for: How to account for the uncertainty from standard toxicity tests in species sensitivity distributions: An example in non-target plants
Source: PLoS One. 2021 Jan 7;16(1):e0245071. doi: 10.1371/journal.pone.0245071 (PMC7790375; doi:10.1371/journal.pone.0245071)
Supplement: Attachment — Submitted filename: Response.pdf [file pone.0245071.s005.pdf]

LBBE
LABORATOIRE DE BIOMÉTRIE ET BIOLOGIE ÉVOLUTIVE

Laboratoire de Biométrie et Biologie Évolutive
UMR CNRS 5558
Université Claude Bernard Lyon 1
Bât Mendel – 43 bd du 11 novembre 1918
69622 Villeurbanne Cedex, FRANCE
Tél : +33(0) 4 72 44 81 42 – Fax : +33(0)4 72 43 13 88
http://lbbe.univ-lyon1.fr

Villeurbanne, December 11, 2020.

Object: revised manuscript [PONE-D-20-21130] - [EMID:9bb6ae7b4856d342]

Dear Editor,

We are pleased to submit a revised version of our manuscript [PONE-D-20-21130] entitled "How to account for the uncertainty from standard toxicity tests in species sensitivity distributions: an example in non-target plants". Our manuscript has been improved with very minor wording changes that we do not detail, by adding recent references to related works by other colleagues (the list of added references is provided at the end of this letter), and by rephrasing some parts according to reviewers' comments (see page 3).

As requested, you will find on the next page: (i) an updated version of the Funding Statement regarding the commercial affiliation of [VD]. According to these modifications, we also changed a bit the Author Contribution section on-line regarding [DW]'s salary support by our ECPA Funder; (ii)an updated version of our Competing Interests Statement. From page 3, we explain how our revised manuscript accounts for the reviewer 2's comments.

Best regards,

Sandrine CHARLES
Docteur en Sciences
Titulaire de l'Habilitation à Diriger des Recherches
Professeur à l'Université C. Bernard Lyon 1

Updated funding statement

The European Crop Protection Association (ECPA, https://www.ecpa.eu) financially supported this work. The funder provided support in the form of salaries for authors [DW], but did not have any additional role in the study design, data collection and analysis, decision to publish, or preparation of the manuscript. The specific roles of these authors are articulated in the 'author contributions' section. One of the author [VD] is employed by a commercial company (Bayer AG, Crop Science). This author collected and made available the raw data used for the modelling work and participated to the preparation of the manuscript but had no role in the study design, data analysis and data interpretation.

Updated Competing Interests Statement

One of the author [VD] is employed by a commercial company (Bayer AG, Crop Science). This author collected and made available the raw data used for the modelling work and participated to the preparation of the manuscript but had no role in the study design, data analysis and data interpretation. This does not alter our adherence to PLOS ONE policies on sharing data and materials.

Laboratoire de Biométrie et Biologie Évolutive (UMR CNRS 5558)
Université Claude Bernard Lyon 1
Bât Mendel – 43 bd du 11 novembre 1918
69622 Villeurbanne Cedex, FRANCE
Tél : +33(0) 4 72 44 81 42 – Fax : +33(0)4 72 43 13 88
http://lbbe.univ-lyon1.fr

LABORATOIRE DE BIOMÉTRIE ET BIOLOGIE ÉVOLUTIVE

Reply to reviewers

First of all, we would like to thank a lot the reviewers for their very positive comments on our submitted manuscript. This is particularly encouraging for us. The three points questioned by reviewer 2 have been addressed in the revised manuscript; changes are marked in red within file `Revised-Manuscript-with-Track-Changes.pdf`. Below, points questioned by reviewer 2 are got back in *italic* and blue, whereas our answers are coloured in black.

1. *What is the novelity of this study?*

   Surprisingly, reviewer 2 himself stated that we presented a novel methodology. So, in order to be more convincing on this aspect, the abstract was slightly modified, as well as the conclusion has been improved at its beginning.

   In essence, the novelty of our study is in: (i) the automatic censuring of ER 50 estimates as interval-input values for the further SSD analyses, based on our innovative criterion 1 (namely, a ratio of probabilities) and a decision threshold fixed to 0.5; (ii) systematically consider SSD input data as intervals, either 95% credible intervals (extracted from 2.5 and 97.5% quantiles of the posterior probability distribution of the previous dose-response analysis under a Bayesian framework), or unbounded intervals of the form [lower bound; $+\infty$) (*i.e.*, right-censored ER 50 estimates), with an automatic decision leading to the most appropriate choice of the lower bound (*i.e.*, the minimum between the lower bound of the 95% credible interval and the maximum tested rate in the experimental design).

2. *What is the difference/advancement in this methodology with the other ones present?*

   The main difference between our novel approach and the ones already existing is the systematic censoring of input data for SSD analyses. Even if already pointed out in the scientific literature that censoring need to be accounted for, any guidance document considers this yet. Our study intends to highlight the added-value of including data as intervals within SSD analyses, based on a case study on non-target terrestrial plants, as well as to convince that considering such intervals in a mathematically sound way is easy as user-friendly tools already exist. The main advancement we propose is thus the automatic building of the interval-censored data, an automatic procedure for which we provide ready-to-use tools.

3. *What is the usefulness of this methodology?*

   As now clearly stated in our conclusion, all our results confirm the usefulness of our integrated approach going from raw toxicity test data until the HR 5 (or HC 5) estimation, considering uncertainty propagation all along the data analysis process. Accounting for ER 50 (or EC 50) estimates as intervals clearly avoid to discard any inputs for SSD analyses, or to arbitrarily convert them to point values. This also avoid to increase uncertainty in the apical estimate of the HR 5 (or HC 5) by keeping as much ER 50 (or EC 50) inputs as possible whatever their associated type of interval (bounded or not). Please also note that the method we proposed in our paper is applicable to any taxon in ecotoxicology.

Laboratoire de Biométrie et Biologie Évolutive (UMR CNRS 5558)
Université Claude Bernard Lyon 1
Bât Mendel – 43 bd du 11 novembre 1918
69622 Villeurbanne Cedex, FRANCE
Tél : +33(0) 4 72 44 81 42 – Fax : +33(0)4 72 43 13 88
http://lbbe.univ-lyon1.fr

List of added references

1. Fox DR, Dam RA, Fisher R, Batley GE, Tillmanns AR, Thorley J, Schwarz CJ, Spry DJ, McTavish K. 2020. Recent developments in SSD Modeling. Environ. Toxicol. Chem.:etc.4925. doi:10.1002/etc.4925.v

2. Gottschalk F, Nowack B. 2013. A probabilistic method for species sensitivity distributions taking into account the inherent uncertainty and variability of effects to estimate environmental risk. Integr. Environ. Assess. Manag. 9:79–86.

3. Posthuma L, van Gils J, Zijp MC, van de Meent D, de Zwartd D. 2019. Species sensitivity distributions for use in environmental protection, assessment, and management of aquatic ecosystems for 12 386 chemicals. Environ. Toxicol. Chem. 38:703–711.

4. Spurgeon D, Lahive E, Robinson A, Short S, Kille P. 2020. Species Sensitivity to Toxic Substances : Evolution , Ecology and Applications. Fontiers Environ. Sci. 8:1–25.

5. Xu FL, Li YL, Wang Y, He W, Kong XZ, Qin N, Liu WX, Wu WJ, Jorgensen SE. 2015. Key issues for the development and application of the species sensitivity distribution (SSD) model for ecological risk assessment. Ecol. Indic. 54:227–237.

6. Zhao J, Zhang R. 2017. Species sensitivity distribution for pentachlorophenol to aquatic organisms based on interval ecotoxicological data. Ecotoxicol. Environ. Saf. 145:193–199.

Laboratoire de Biométrie et Biologie Évolutive (UMR CNRS 5558)
Université Claude Bernard Lyon 1
Bât Mendel – 43 bd du 11 novembre 1918
69622 Villeurbanne Cedex, FRANCE
Tél : +33(0) 4 72 44 81 42 – Fax : +33(0)4 72 43 13 88
http://lbbe.univ-lyon1.fr
